# SIRT6-Mediated Regulation of TFAM: A Central Mechanism Connecting Nuclear and Mitochondrial Transcriptional Processes and Mitophagy

**DOI:** 10.7150/ijbs.120007

**Published:** 2026-01-01

**Authors:** Meimei Jiang, Jiehan Li, Ning Ding, Guiyun Jia, Siming Wu, Nannan Liu, Ying Kang, Ge Zhang, Jiawei Wu, Lingling Zhang, Yingjie Zhang

**Affiliations:** 1 School of Biomedical Sciences, Hunan University, Changsha, 410082 Hunan, China.; 2 Department of Gastroenterology, the First Affiliated Hospital of Zhengzhou University, Zhengzhou, 450052 Henan, China.; 3 Department of Laboratory Medicine, the Third Xiangya Hospital, Central South University, Changsha, 410083 Hunan, China.

**Keywords:** SIRT6, TFAM, mitochondrial dysfunction, FoxA1, transcription regulation, mitophagy

## Abstract

Nuclear and mitochondrial transcriptional regulation represent distinct mechanisms of gene expression control, both of which have garnered significant scientific attention. However, the interplay between these two regulatory processes remains poorly understood and underexplored. Our research uncovers a novel link between nuclear and mitochondrial transcription by identifying SIRT6 as an upstream regulator of the mitochondrial transcription factor TFAM, acting both indirectly and directly. Mechanistically, SIRT6 deacetylates FoxA1 at the K267 site, blocks the binding of FoxA1 to the promoter region of TFAM, leading to reduced TFAM expression. In parallel, SIRT6 translocates to the mitochondria and directly deacetylates TFAM at the K154 site, suppressing its transcriptional activity. Furthermore, SIRT6 downregulates the expression level of mitochondrial genes and proteins, inducing mitochondrial dysfunction and mitophagy by targeting TFAM. Additionally, TFAM promotes the growth and metastasis of colon cancer *in vitro* and *in vivo*, while SIRT6 was inhibited. In conclusion, our findings provide compelling evidence that SIRT6 establishes a network linking nuclear and mitochondrial transcription through the regulation of TFAM, identifying TFAM as a potential therapeutic target for cancer.

## Introduction

Mitochondria are essential organelles in eukaryotic cells, consisting of the outer membrane, inner membrane (with cristae), intermembrane space, and matrix. Many proteins embedded in the inner membrane, including the mitochondrial respiratory chain complexes and various transport carriers, are localized there. From an energy conversion perspective, the inner membrane plays a crucial role. Enzymes involved in the citric acid cycle, fatty acid and pyruvate oxidation, as well as heme synthesis, are all located in the matrix. Mitochondria are central to cellular energy metabolism and play crucial roles in maintaining both cellular health and disease progression. When the mitochondrial membrane is damaged, the respiratory chain is inhibited, enzyme activity is reduced, or mitochondrial DNA is compromised, mitochondrial dysfunction can occur. Mitochondrial dysfunction can lead to alterations in mitochondrial morphology, reduced ATP synthesis, excessive reactive oxygen species (ROS) production, imbalanced dynamics, and mtDNA damage^1^. Mitochondrial dysfunction has been implicated in various common pathological conditions, including cardiovascular diseases (ischemic/reperfusion injury)^2^, ^3^, neurodegenerative diseases^4^, ^5^, aging^6^, ^7^, metabolic syndrome^8^, ^9^, and cancer^10^, ^11^.

Mitochondria possess their own genome and have independent mitochondrial transcription and translation mechanisms. Mitochondrial transcription is primarily driven by mitochondrial RNA polymerase (POLRMT), which directly interacts with promoter elements and requires mitochondrial transcription factor A (TFAM) or mitochondrial transcription factor B2 (TFB2M) to initiate transcription^12^. The nuclear transcription regulatory mechanism is another important control mechanism for gene expression within the cell. The transcriptional regulatory mechanisms in the nucleus are typically composed of various factors, including transcription factors, coactivators, corepressors, chromatin modifications, and so on working together. Recent studies have highlighted the potential for nuclear transcription factors to influence mitochondrial transcription. Mitochondrial function is primarily mediated by nuclear-encoded gene signals, which increase mitochondrial activity to meet cellular demands. This regulation depends on nuclear-encoded transcription factors, such as PCG1α, NRF1, along with other co-regulatory factors, to influence the expression of mtDNA-encoded genes^13^, ^14^. Emerging research suggests that nuclear transcription factors play a key role in regulating the expression of mtDNA genes^15^, ^16^. However, the exact mechanisms through which nuclear factors influence mtDNA transcription, and how changes in nuclear gene expression regulate mitochondrial function, remain poorly understood. Therefore, elucidating whether nuclear factors directly regulate mtDNA transcription in mitochondria presents a significant challenge.

SIRT6, a nuclear-localized deacetylase, has been shown to play an important role in DNA damage repair^17^, telomere maintenance^18^, aging^19^, metabolic homeostasis^20^, inflammation^21^, and tumorigenesis^21^. In relation to mitochondrial function, SIRT6 exerts multiple regulatory effects, influencing processes such as mitochondrial energy production, respiration, and ATP synthesis^22^. Additionally, SIRT6 is considered to have antioxidant properties, helping mitigate oxidative stress-induced damage to mitochondria and thus maintaining their normal function^23^. SIRT6 was first reported to play a significant role in the aging brain and neurodegeneration by modulating SIRT3 and SIRT4, thereby inhibiting the expression of mitochondrial-related genes, preventing mitochondrial dysfunction, and reducing ROS production^24^. SIRT6 reduces ROS production during damage^25^, and activates the AMPK pathway or the PGC1α/AKT axis^26^ to maintaining mitochondrial homeostasis, and enhancing mitochondrial biogenesis. Our previous research identified SIRT6 as a target gene of FOXO3a and suggested that it could serve as a potential therapeutic target for colorectal cancer (CRC)^27^. Furthermore, our previous study showed that SIRT6 promotes autophagy by competitively binding to PUMA in CRC^28^.

Mitochondrial transcription factor A (TFAM) is a key regulator involved in the activation of mitochondrial DNA transcription and regulation of mtDNA copy number. TFAM is encoded by nuclear genes and is translocated into the mitochondrial matrix, where it exerts its regulatory function, improving mitochondrial function. TFAM binds to mtDNA to form a DNA-protein complex^29^, ^30^, which helps maintain the stability of mitochondrial genes^31^ and regulates the expression of mitochondrial genes to sustain energy production^32^. Recent studies have implicated TFAM in tumor progression. Abnormal TFAM expression can lead to dysfunction of mitochondrial respiratory chain complexes and a reduction in oxidative phosphorylation efficiency. For instance, TFAM expression was upregulated in colitis-associated cancer tissues and contributed to cell growth. TFAM promoted the proliferation of both intestinal epithelial cells (IECs) and CRC cells by enhancing mitochondrial biogenesis and activity^33^. Additionally, TFAM was significantly downregulated in metastatic hepatocellular carcinoma (HCC) tissues and was associated with overall survival and tumor recurrence in HCC patients^34^. Intestinal-specific knockout of TFAM has been shown to prevent tumor formation in Apc-mutant mouse models of colon cancer^35^. Moreover, MCU-induced mitochondrial Ca^2+^ uptake promotes mitochondrial biogenesis by suppressing TFAM phosphorylation, thus contributing to CRC cell growth^36^.

While previous studies have demonstrated that SIRT6 influences mitochondrial function through the regulation of aging-related signaling pathways, the specific role of SIRT6 in mitochondrial function regulation and its impact on tumorigenesis remain underexplored in cancer models. Particularly, the direct or indirect regulation of TFAM by SIRT6 and how this regulation affects mitochondrial dysfunction and mitophagy remains an unresolved scientific question. Investigating these mechanisms will clarify the specific role of SIRT6 in CRC and provide potential targets for the development of novel therapeutic strategies. Recent studies suggest that mitochondrial dysfunction is a key factor in the treatment of CRC. By targeting mitochondria with drugs to inhibit mitochondrial energy supply, tumor progression can be suppressed through the induction of immune responses, or by triggering cell pyroptosis^3^, apoptosis^37^, ^38^, ferroptosis^39^, ^40^, and mitophagy^41^, ^42^. This study aims to investigate whether and how SIRT6 induces mitochondrial dysfunction through the regulation of TFAM, leading to mitophagy, and consequently inhibiting colorectal cancer growth.

## Materials and Methods

### Bioinformatics analysis

The SIRT6-Cor-Gene set was derived from the correlation of SIRT6 with genes from the TCGA-COAD and TCGA-READ databases using RStudio. Gene sets with an absolute correlation value greater than 0.4 (either positive or negative) were selected, and GO (Gene Ontology) and KEGG (Kyoto Encyclopedia of Genes and Genomes) analyses were then performed on these correlated genes. The GO analysis was retrieved from the Gene Ontology database (https://www.geneontology.org/) via R-studio. The KEGG analysis was retrieved from the KEGG PATHWAY Database (https://www.genome.jp/kegg/pathway.html) via R-studio. Survival analysis and correlation relationships were analyzed using the GEPIA database. Mitochondria databases were acquired from Human MitoCarta3.0 (https://www.broadinstitute.org/files/shared/metabolism/mitocarta/human.mitocarta3.0.html) and MitoProteome Human Mitochondrial Protein Database (http://mitoproteome.org/). The predicted transcription factors targeted to TFAM were obtained from PROMO (http://alggen.lsi.upc.es/cgi-bin/promo_v3/promo/promoinit.cgi?dirDB=TF_8.3), hTFtarget (https://guolab.wchscu.cn/hTFtarget/#!/), JASPAR (https://jaspar.elixir.no/) and KnockTF (http://www.licpathway.net/KnockTF/index.php). PPI was analyzed from online website STRING (https://string-db.org/). The acetylation sites were acquired from GPS-PAIL 2.0-Prediction of Acetylation on Internal Lysines (https://pail.biocuckoo.org/online.php) and PhosphoSitePlus (https://www.phosphosite.org/homeAction.action).

### Cell lines treatment

HCT116 (RRID: CVCL_0291) and HEK293T (RRID: CVCL_0063) cells were ordered from ATCC (CCL-247/ CRL-3216). Confirmed mycoplasma-free using the Mycoplasma PCR Detection Kit (Beyotime, Cat# P2078, Shanghai, China) prior to experiments. Cells cultured in DMEM with 10% fetal bovine serum at 37°C under 5% CO_2_ in a humidified incubator. For treatment, CCCP, FCCP, Oligomycin, Rotenone and Actinomycin A diluted with DMSO were added to the medium directly before detection. For transfection, cells were plated in a 6-well or 12-well plate 24 h before transfection, and the indicated plasmids were transfected using the Neofect DNA transfection reagent (Wuhan, China). Cell freezing medium used from NCM (Cat#C40100, Suzhou, China).

### Antibodies and reagents

Primary antibodies against TFAM, SIRT6, FoxA1, Tom20, Tim23, Lc3, P62, ND1, ND2, and so on. were purchased from Proteintech (Wuhan, China) and ZEN-BIO (Chengdu, China). The reagents CCCP, FCCP, Oligomycin, Rotenone and Actinomycin A were purchased from Selleck.

### Mitochondrial DNA content assessment

The genomic DNA was extracted using TIANamp Genomic DNA Kit (TIANGEN, Cat#Y2208, Beijing, China). After transfecting indicated plasmid, RT-qPCR detected the mtDNA. RT-qPCR detected the nuclear DNA. The mtDNA copy number was determined by the ratio of mtDNA to nDNA^43^.

### Cellular ATP measurements

After transfecting indicated plasmid, and the cellular ATP was extracted using ATP Content Assay Kit-Microplate Reader (Solarbio, Cat#BC0305, Beijing, China).

### Mitochondrial membrane potential (MMP) determination

A JC-1 kit (YEASEN, Cat# 40706ES60, Beijing, China) was used to detect the membrane potential of the treated groups, which were seeded in a 24-well plate and stained with JC-1 via incubation in the working solution for 20 min, and flow cytometry was used to observe the fluorescence intensity.

### Measurement of mitochondrial Reactive oxygen species (ROS)

A ROS kit (UE, Cat# R6033, Suzhou, China) was used to detect the changes in the levels and variations of reactive oxygen species (ROS) in the damaged cells. And images captured to observe the fluorescence intensity via EVOSTM M5000 (Thermo, America).

### Oxygen Consumption Rate (OCR) determination

Experimental design followed the protocol provided by Mitochondrial Oxygen Consumption Rate Assay Kit (Bestbio, Cat# BB-482112, Shanghai, China) via BioTek Synergy H1 (Agilent, America). First, cells were injected with 1μM oligomycin, which inhibits ATP synthesis and identifies the percentage of OCR devoted to ATP synthesis. The second compound, 1 μM FCCP, was used to calculate the maximum and spare respiratory capacity of cells. Finally, cells were exposed to a combination of 1 μM rotenone (a complex I inhibitor), and 1 μM antimycin A (a complex III inhibitor). This combination inhibits mitochondrial respiration and allows calculation of the mitochondrial and non-mitochondrial fractions contributing to respiration.

### Western blotting

Protein samples were extracted using RIPA buffer, and SDS-PAGE was performed on a 10% or 12% gel. The proteins were transferred to a PVDF membrane (Millipore) and sealed overnight at 4°C with 5% skim milk. The membrane was incubated with the primary antibody overnight at 4°C. Horseradish peroxidase (HRP)-conjugated anti-rabbit or anti-mouse secondary antibody was used. Bands were visualized using an ECL Plus Kit (GLPBIO, Cat# GK10008, America) via Odyssey Infrared Imaging System (JIA PENG, Shanghai, China).

### RT-qPCR

Total RNA was extracted from CRC cells using the TransZol (Transgen, Cat# ET101-01, Beijing, China). RNA quality was assessed using the NanoDrop ND-1000. The cDNA synthesis was performed using 1 μg of total RNA with the Hiscript III All-in-one RT SuperMix Perfect for qPCR Kit (Vazyme, Cat# R331-01, Nanjing, China). RT-qPCR was conducted using the ChamQ Universal SYBR qPCR Master Mix Kit (Vazyme, Cat# Q711-02, Nanjing, China). Primer sequences used for gene amplification were provided in Table 1.

### Chromatin Immunoprecipitation (ChIP)-qPCR

The chromatin immunoprecipitation (ChIP) experiment was conducted using the ChIP Assay Kit (Beyotime, Cat# P2078, Shanghai, China). The products for PCR detection were obtained after DNA purification using the universal DNA purification kit (Tiangen). The primer sequence of the four predicted binding sites for FoxA1 to interact with TFAM were shown in Table 2.

### Plasmid construction

The mutant plasmids for FoxA1 (K237Q/R, K240Q/R, K264Q/R, K267Q/R, K270Q/R) were generated using Myc-His-FoxA1 as the vector template, while TFAM (76Q/R, 154Q/R) mutants employed His-mCherry-TFAM as the template. Complementary primers containing the desired mutation sites were used for PCR amplification. Template plasmids were digested with DpnI enzyme to selectively retain the PCR-amplified mutant plasmids. The resulting PCR products were ligated into circular plasmids and transformed into *E. coli* DH5α competent cells for screening of successful transformants. The forward and reverse amplification primers for point mutations in the mutant plasmids used in this study are listed in the Table 3.

### Dual-luciferase reporter assays

The dual-luciferase reporter plasmid pmirGLO (pGLO) was used as the empty vector backbone. Full-length or truncated TFAM promoter sequences were inserted upstream of the firefly luciferase (Fluc) coding sequence to construct the required experimental plasmids. After transfecting indicated plasmid into HEK293Tcells, the cells were lysed using the Dual Luciferase Reporter Gene Assay Cell Lysis Buffer (Beyotime, Cat# RG132, Shanghai, China), and luminescence was detected using the Dual-Lumi™ Luciferase Assay Kit (Beyotime, Cat# RG088, Shanghai, China).When using sea kidney luciferase as an internal control, the RLU value obtained from the firefly luciferase assay is normalized by dividing it by the RLU value from the sea kidney luciferase assay.

### Electrophoretic mobility shift assay (EMSA)

His-tagged FoxA1 protein was purified *in vitro* and FAM-labeled BS2 probes, unlabeled BS2 probes, and unlabeled mutBS2 probes were designed by Sangon Biotech. The protein was mixed with the corresponding probes in Binding buffer (Tris 100 mM, KCl 500 mM, DTT 10 mM, pH 7.5) and incubated at 25°C for 45 minutes. The mixture was then subjected to electrophoresis on a 4% PAGE gel for observation.

### Co-immunoprecipitation (Co-IP)

Cells with different treatments were resuspended in lysis buffer, incubated, and precleared with Protein A/G Agarose and rabbit immunoglobulin G. Precleared lysates were incubated with the corresponding primary antibody or control immunoglobulin G overnight. The complexes were washed, eluted, and analyzed using SDS-PAGE. For mass spectrometry analysis, Co-IP eluates were subjected to in-gel tryptic digestion, and peptides were analyzed by LC-MS/MS (Novogene Co. Ltd).

### Immunofluorescence

Cells were plated on coverslips and fixed with 4% paraformaldehyde (PFA) for 30 minutes. The cells were then permeabilized with 0.1%-0.5% Triton X-100 for 5-10 minutes to allow antibodies to enter the cells. To block non-specific binding sites, the cells were incubated with PBS containing 5%-10% normal goat serum for 30-60 minutes. The primary antibody, specific to the target molecule, was added and incubated overnight at 4°C. The cells were washed several times with PBS. A fluorescence-labeled secondary antibody, matching the species of the primary antibody, was added and incubated for 1 hour. After washing with PBS, the cells were stained with DAPI for 10-15 minutes to label the nuclei. A mounting medium was applied to preserve fluorescence, and a coverslip was placed over the cells. Finally, the cells were observed under a fluorescence microscope, and images were captured to analyze the expression and localization of the target protein.

### Protein-protein docking

The structure of the key target protein (TFAM, SIRT6) was obtained from the PDB database (https://www.rcsb.org). The protein structure was processed using Pymol software to remove water molecules and extract the target protein structure. Protein-protein docking was performed using GRAMM (https://gramm.compbio.ku.edu/). The resulting chemical bond interactions were analyzed using LigPlot+ software, and the docking results were visualized using Pymol software^44^.

### Mitophagy assessment

Mitophagy was evaluated by counting GFP-Lc3 and Ds-Red-mito positive or mCherry-Tim23 colocalized with GFP-Lc3 merged dots in the presence of CRC cells with different treatment. After transfecting Ds-Red-mito, and immunofluorescence staining of Lamp1, or immunofluorescence staining of Lamp1 and Tim23, typical images were captured. The mitophagy flux marker, mito-Keima was used to detect the occurrence of mitophagy. Cell images were captured using a Zeiss LSM980 confocal microscope. Confocal image magnification: 100x.

### Transmission electronic microscopy (TEM)

For TEM detection, cell samples were trimmed into smaller pieces and fixed with 2% glutaraldehyde. Images were obtained using a TEM (JEOL Ltd., Tokyo, Japan). The TEM images were analyzed using Image J software. Intracellular double-membrane vesicles containing mitochondria were identified as mitochondrial-autophagosomes, which reflect the level of mitophagy activity in cell samples. The number of mitochondrial autophagosomes was calculated in an area larger than 1200 μm^2^ in six images per group.

### Cell viability assays and colony formation

After cells treated with different treatment, then, cells were seeded at a density of 3×10^3^ cells per well in 96-well plate. After incubation, 10 μL CCK-8 reagent (Solarbio, Beijing, China) was added to each well, followed by further incubation in an incubator. The absorbance at 450 nm was measured using a microplate reader. CRC cells were seeded at a density of 1×10^3^ cells per well in 6-well plate. After two weeks of incubation, the number of colonies was counted by staining with crystal violet.

### Scratch assay

Cells were seeded in 6-well plates and allowed to grow until they reached 95% confluence. One hours prior to scratching, the medium was changed to ensure that the cells were in a good condition. On the back of the 6-well plate, perpendicular lines intersecting the scratch were marked with a black marker to identify the position for photography. For scratching, a 10 μL pipette tip was used to draw a straight, uniform scratch. The wells were gently washed thrice with PBS to remove a large number of floating cells, as observed under a microscope. Serum-free medium was added and photographs were taken at 0 h, 24 h, and 48 h to record the experimental data.

### Edu assay

EDU assay was analyzed by using BeyoClick™ EdU Cell Proliferation Kit with Alexa Fluor 555 (Beyotime, Cat# C0075L, Shanghai, China). After cells within different treatment, and cells were cultured to an appropriate density, typically during the logarithmic phase of growth. Images were analyzed by Image J software.

### Human tissue analysis

Human CRC and matching normal colonic tissues were obtained from consented patients at the First Affiliated Hospital of Zhengzhou University. Tissues collected from the Surgical Pathology Laboratory after surgical resection were immediately processed and then analyzed by Western blot analysis, RT-qPCR, and IHC.

### Construction of xenograft tumor model

Four-week-old female nude mice were injected subcutaneously with 1
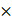
10^6^ HCT116 cells (shVector+oeVector, oeSIRT6, and shTFAM) into the flank of nude mice. The mice were then fed normally, with continuous regular monitoring of tumor size (volume formula = 0.5×length×width^2^). At the end of the experiment, the mice were euthanized by dislocation and the tumors were carefully dissected and used for further experiments.

### Immunohistochemistry staining

After fixing the tumor tissue samples in 4% paraformaldehyde for 24 h, they were dehydrated, embedded, and sectioned for slide preparation. Following antigen retrieval and blocking of endogenous peroxidase activity, the slides were incubated overnight with antibodies against related proteins, followed by incubation with secondary antibodies. After staining, dehydration, and mounting, the slides were observed under a microscope and the images obtained were collected and saved.

### Statistical analysis

Fluorescence colocalization analysis: A Pearson's correlation coefficient (r) ranging from -1.0 to 0.5 is considered indicative of no colocalization, while a Pearson's correlation coefficient (r) between 0.5 and 1.0 is regarded as evidence of colocalization. The data were graphically plotted using R package and GraphPad Prism version 8.02 software. The T-test and two-way ANOVA were used to analyze group differences. A value of P<0.05 was regarded as statistically significant for two-sided statistical tests.

## Results

### SIRT6 induced mitochondrial dysfunction

**Figure 1:**
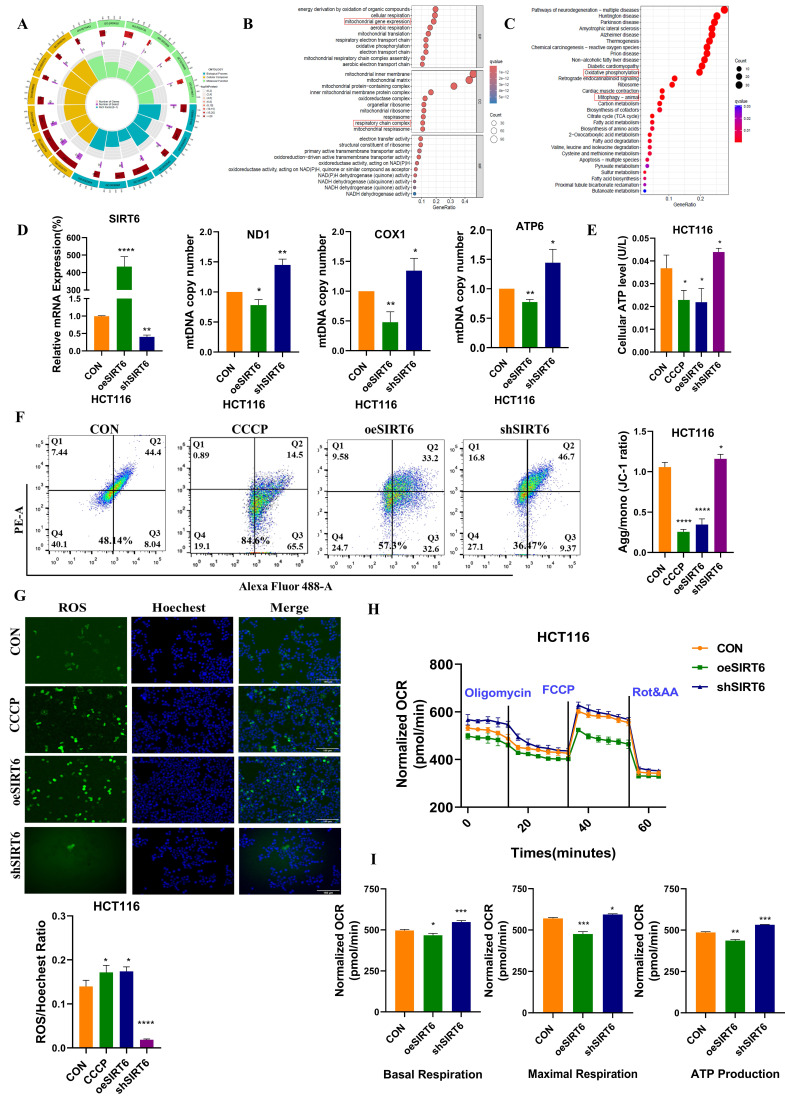
** Overexpression of SIRT6 induces mitochondrial damage.** (A-C) Bioinformatics analysis using Gene Ontology (GO) and Kyoto Encyclopedia of Genes and Genomes (KEGG) pathways was performed to predict the relationship between SIRT6 and mitochondrial function. (D) Mitochondrial DNA (mtDNA) copy number was measured by detecting ND1/COX1/ATP6 DNA levels in HCT116 cells. (E) Cellular ATP levels were determined in SIRT6-overexpressed and knockdown HCT116 cells. (F) Mitochondrial membrane potential was measured using the JC-1 probe in HCT116 cells. (G) Reactive oxygen species (ROS) levels were detected in HCT116 cells. Scale bar = 150 μm (H-I) Oxygen consumption rate (OCR) was measured in HCT116 cells. Basal respiration, maximal respiration, and ATP production were quantified from (H). Error bars represent mean ± SEM. NS, not significant; statistical significance: *P < 0.05, **P < 0.01, ***P < 0.001 vs control group. Data are representative of three independent experiments.

To explore the potential correlation between SIRT6 and mitochondrial function, enrichment analysis using bioinformatics methods on the GO database was performed. The results revealed that SIRT6 is associated with mitochondria tightly (Figure 1A). As shown in Figure 1B, SIRT6 was found to be involved in the regulation of mitochondrial gene expression and oxidative phosphorylation, potentially affecting the respiratory chain. These findings suggest that SIRT6 plays a critical role in regulating mitochondrial function. Furthermore, KEGG pathway enrichment analysis further revealed that SIRT6 is predominantly enriched in the mitophagy and oxidative phosphorylation pathways, indicating a strong correlation between SIRT6 and mitophagy. This implies that SIRT6 may play a significant role in the regulation of mitophagy, which is crucial for maintaining mitochondrial quality and function (Figure 1C).

Next, overexpression or knockdown of SIRT6 was performed in HCT116 cells, leading to significant changes in mtDNA copy numbers. Overexpression of SIRT6 resulted in a decrease in the DNA levels of ND1, COX1, and ATP6 on mtDNA, while SIRT6 knockdown led to an upregulation of these genes (Figure 1D). This indicates that SIRT6 induces mitochondrial DNA damage. Additionally, cellular ATP levels were measured, and the results showed that SIRT6 overexpression reduced cellular ATP production (Figure 1E). The mitochondrial membrane potential (MMP) was also assessed, revealing that overexpression of SIRT6 significantly induced mitochondrial depolarization, as evidenced by a reduction in membrane potential. This effect was consistent with the results observed in the positive control group treated with CCCP. Quantitative analysis further confirmed that SIRT6 induced mitochondrial depolarization to a similar extent as CCCP (Figure 1F). What's more, reactive oxygen species (ROS) levels were markedly elevated in the SIRT6 overexpression group compared to the control group, showing a same increase comparable to that in the CCCP-treated group. Quantitative analysis consistently demonstrated that SIRT6 induced oxidative stress (Figure 1G).

To further investigate the impact of SIRT6 on mitochondrial function, the oxygen consumption rate (OCR) was measured. Using oligomycin, the uncoupler FCCP, and electron transport inhibitors antimycin A and rotenone, the SIRT6 overexpression group exhibited reduced basal respiration, maximal respiration, and ATP production compared to the control group (Figure 1H-I).

Taken together, these findings confirmed that overexpression of SIRT6 leads to mitochondrial damage, resulting in mitochondrial dysfunction.

### SIRT6 inhibited TFAM expression through FoxA1

**Figure 2:**
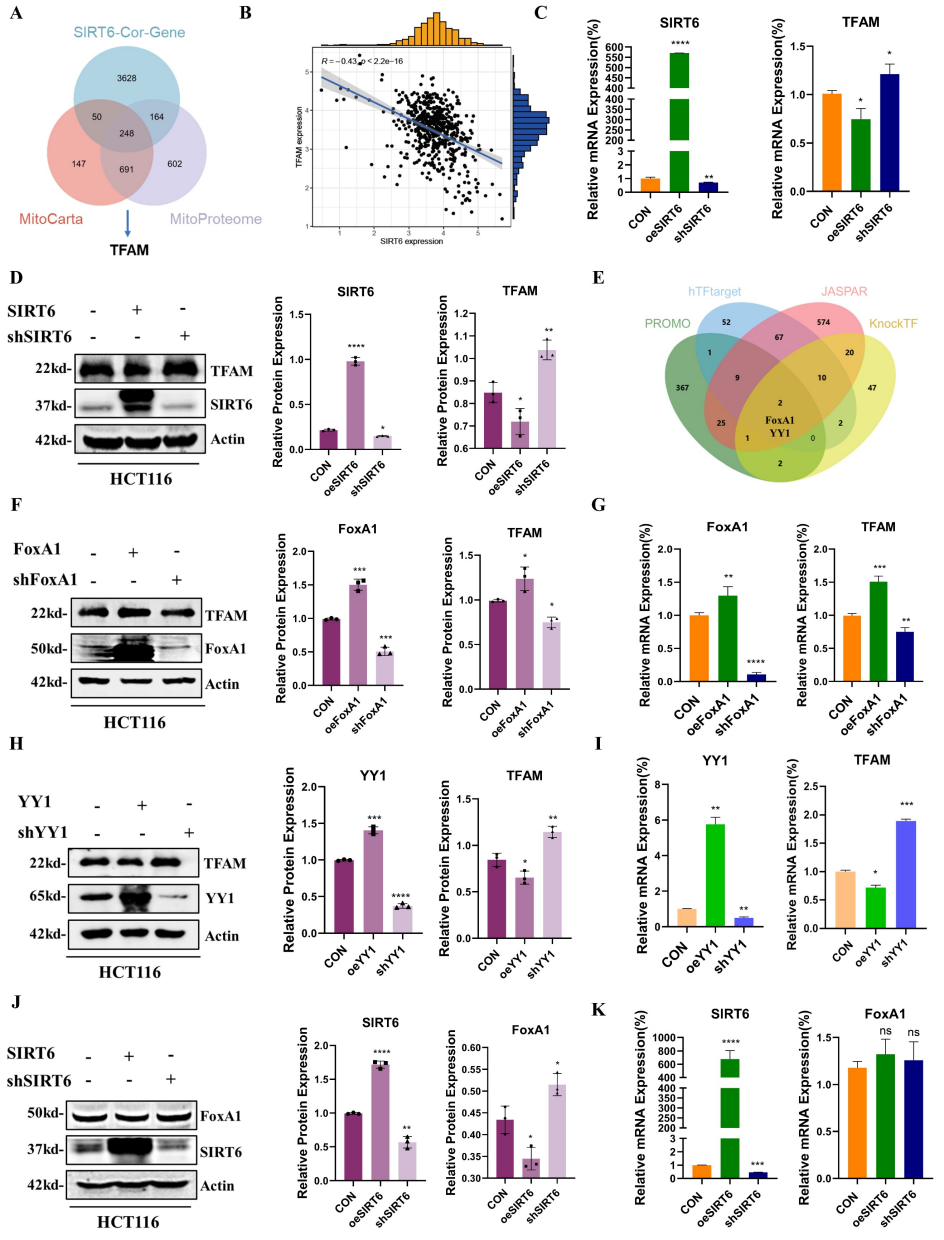
** SIRT6 inhibits the expression of TFAM.** (A) Intersection of SIRT6-Cor-Gene, MitoCarta, and Mitoproteome databases to identify potential relationships between SIRT6 and mitochondrial function. (B) Correlation between SIRT6 and TFAM expression was analyzed using RStudio. (C) RT-qPCR analysis of mRNA levels of TFAM and SIRT6. (D) Western blotting analysis of TFAM and SIRT6 protein levels in HCT116 cells with overexpression or knockdown of SIRT6. Quantification of TFAM and SIRT6 protein levels under different treatments. (E) Transcription factor targets of TFAM were identified through the intersection of five online databases. (F-G) Protein and mRNA levels of TFAM were assessed in FoxA1-overexpressed or FoxA1-knockdown HCT116 cells. (H-I) Protein and mRNA levels of TFAM were assessed in YY1-overexpressed or YY1-knockdown HCT116 cells. (J-K) Protein and mRNA levels of FoxA1 were determined in SIRT6-overexpressed or SIRT6-knockdown HCT116 cells. Error bars represent mean ± SEM. NS, not significant; statistical significance: *P < 0.05, **P < 0.01, ***P < 0.001 vs control group. Data are representative of three independent experiments.

To further investigate the molecular mechanism by which SIRT6 affects mitochondrial function, the mitochondrial protein-related databases MitoCarta and Mitoproteome with the SIRT6-Cor-Gene data was intersected. Among the intersecting factors, one key protein found was TFAM, a mitochondrial transcription factor closely associated with mitochondrial function. It was hypothesized that the mitochondrial dysfunction induced by SIRT6 is likely related to TFAM (Figure 2A and see Supplementary Table 1). Next, the correlation between SIRT6 and TFAM was predicted, which revealed a negative regulatory relationship between them (Figure 2B). SIRT6 affected TFAM mRNA levels, with overexpression of SIRT6 leading to a decrease in mRNA expression of TFAM (Figure 2C). Similarly, Western blotting confirmed that TFAM protein levels decreased when SIRT6 was overexpressed, while SIRT6 knockdown significantly upregulated the protein levels of TFAM (Figure 2D). These findings suggested that SIRT6 inhibits TFAM expression at both the protein and transcriptional levels.

The potential transcription factors targeting TFAM was acquired from the intersect datasets within four databases (PROMO, hTFtarget, JASPAR, and KnockTF) (Figure 2E and see Supplementary Table 2), which identified FoxA1 and YY1 as potential transcription factors for TFAM. Additionally, protein-protein interaction (PPI) analysis revealed a potential association between SIRT6 and both FoxA1 and YY1 (Figure S1A). Next, the impact of FoxA1 and YY1 on TFAM expression was investigated by overexpressing or knocking down these transcription factors in cells. Western blot analysis showed that FoxA1 significantly upregulated TFAM protein expression, and RT-qPCR results indicated that overexpression of FoxA1 also increased TFAM mRNA levels (Figure 2F-G). In contrast, overexpression of YY1 led to a reduction in both TFAM protein and mRNA levels (Figure 2H-I). To explore how SIRT6 regulates these transcription factors, the protein and mRNA levels of FoxA1 and YY1 was analyzed following SIRT6 overexpression or knockdown. The results indicated that SIRT6 downregulated FoxA1 protein levels without affecting its mRNA levels (Figure 2J-K). Additionally, as shown in Figure S1B-C, overexpression of SIRT6 reduced YY1 expression levels. Whether SIRT6 influences TFAM through the well-known transcription factor PGC-1α and Nrf1 (Figure S1D-E), the results demonstrated that SIRT6 might suppress the expression of PGC-1α and Nrf1, providing an additional mechanism through which SIRT6 might regulate TFAM.

Taken together, these findings suggested that SIRT6 likely induces mitochondrial dysfunction by inhibiting the expression of a transcription factor FoxA1, thereby suppressing its transcriptional activation of TFAM.

### FoxA1 is a direct transcriptional factor of TFAM

**Figure 3:**
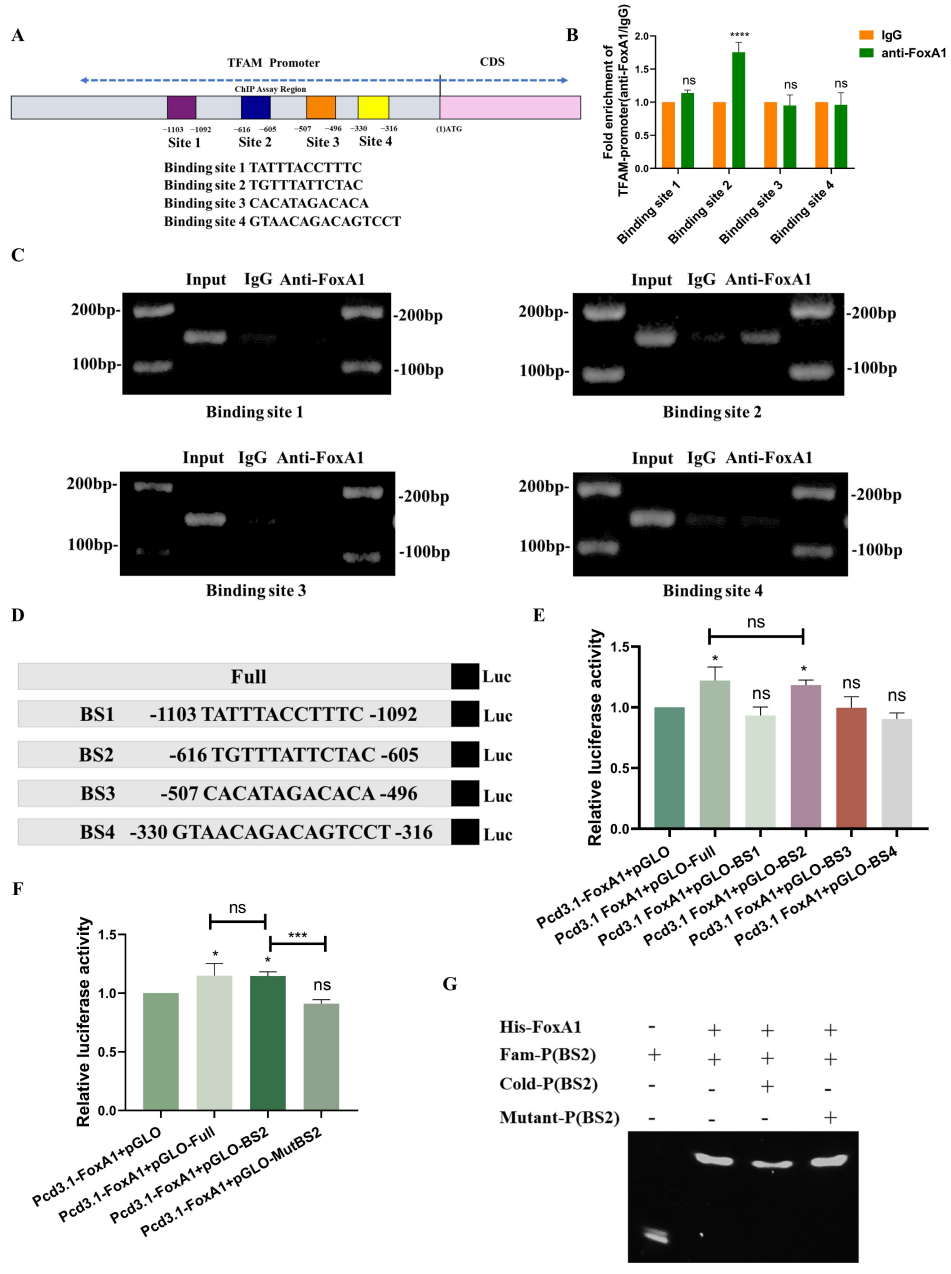
** FoxA1 binds to the TFAM promoter through BS2.** (A) Potential binding sites of FoxA1 on the TFAM promoter were predicted using hTFtarget and JASPAR databases. (B-C) Chromatin immunoprecipitation (ChIP) assays determined the binding of FoxA1 to the TFAM promoter, analyzed by RT-qPCR. (D-F) Dual-luciferase reporter assays were used to measure luciferase activity in cells with different treatments. (G) Electrophoretic mobility shift assay (EMSA) was performed to determine the interaction between Fam-BS2 and FoxA1. Error bars represent mean ± SEM. NS, not significant; statistical significance: *P < 0.05, **P < 0.01, ***P < 0.001 vs control group. Data are representative of three independent experiments.

To investigate how the transcription factor FoxA1 binds to the TFAM promoter and identify specific binding sites, four potential binding sites within the TFAM promoter were predicted: BS1 (TATTTACCTTTC), BS2 (TGTTTATTCTAC), BS3 (CACATAGACACA), and BS4 (GTAACAGACAGTCCT) (Figure S2A and 3A). Chromatin immunoprecipitation (ChIP) assays, as shown in Figures 3B-C, the results revealed that BS2 is the most likely binding region for FoxA1 on the TFAM promoter.

To further validate this finding, dual-luciferase reporter plasmids were constructed containing the full-length TFAM promoter as well as four constructs with individual potential binding sites. Dual-luciferase reporter assays showed that the bioluminescence of the plasmid containing BS2 was comparable to that of the full-length TFAM promoter construct, and significantly stronger than that of the other three binding site constructs (Figure 3D-E). To confirm the role of BS2, mutations were introduced into the BS2 sequence and constructed a dual-luciferase reporter plasmid containing the mutated BS2 (Mut BS2) (Figure S2B). The results of dual-luciferase reporter assays demonstrated that, after mutation of BS2, the bioluminescence signal was similar to that of the control group and significantly weaker than that of the other treatment groups (Figure 3F). Next, FoxA1 protein was purified *in vitro* and a BS2 double-stranded DNA fragment labeled with a FAM probe was designed, along with an unlabeled competitive probe and an unlabeled mutated probe. *In vitro* binding assays confirmed that FoxA1 specifically binds to the BS2 sequence (Figure 3G).

In summary, these findings confirmed that the transcription factor FoxA1 binds to the BS2 sequence within the TFAM promoter and activates its transcriptional regulation.

### SIRT6 directly deacetylated the K267 site of FoxA1 to inhibit its transcriptional activity on TFAM

**Figure 4:**
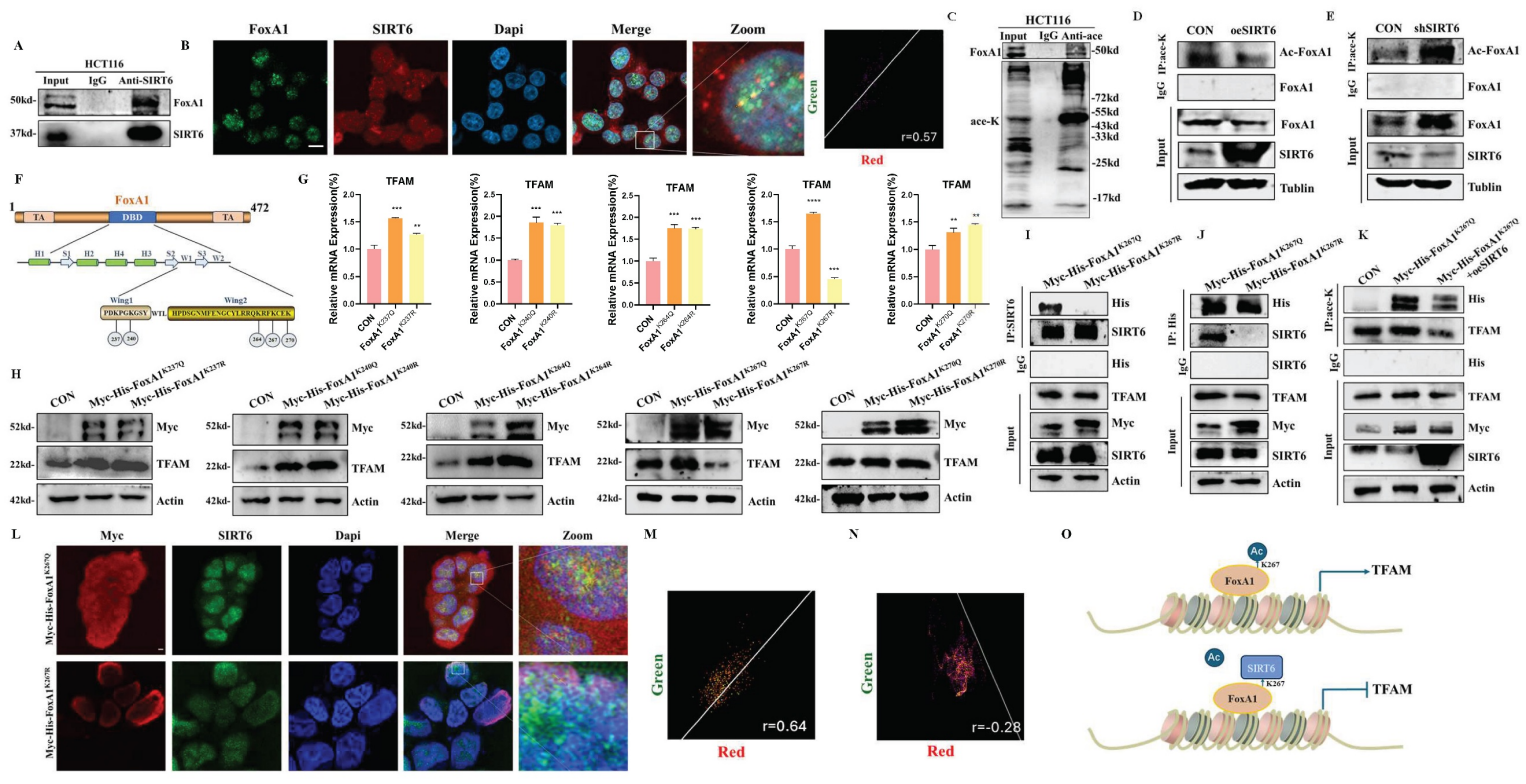
** SIRT6 deacetylates FoxA1 at the K267 site.** (A) Co-immunoprecipitation (Co-IP) analysis of SIRT6 binding to FoxA1 in HCT116 cells. (B) Immunofluorescence staining of SIRT6 and FoxA1 was performed, followed by confocal microscopy. Quantitative co-localization was analyzed using Coloc 2 in ImageJ software. Scale bar = 10 μm. (C) Co-IP analysis of acetylation levels of FoxA1 in HCT116 cells. (D-E) Co-IP analysis of acetylation levels of FoxA1 in SIRT6-overexpressed or knockdown HCT116 cells. (F) Schematics of prediction of potential acetylation sites on FoxA1. (G-H) After transfection of FoxA1 mutants (K237Q/R, K240Q/R, K264Q/R, K267Q/R, K270Q/R) into 293T cells, TFAM mRNA and protein levels were analyzed. (I-J) Co-IP analysis of SIRT6 binding to FoxA1^K267Q^ mutant in 293T cells. (K) IP analysis of acetylation levels of exogenous FoxA1^K267Q^ and combination of FoxA1^K267Q^ and SIRT6 in 293T cells. (L-N) Immunofluorescence staining of SIRT6 and Myc in FoxA1^K267Q^ and FoxA1^K267R^-transfected 293T cells, analyzed by confocal microscopy. Quantitative co-localization analysis was performed using Coloc 2 in ImageJ software. Scale bar = 10 μm. (O) Simplified mechanistic flowchart for Figure 4. Error bars represent mean ± SEM. NS, not significant; statistical significance: *P < 0.05, **P < 0.01, ***P < 0.001 vs control group. Data are representative of three independent experiments.

Based on the results described above, SIRT6 reduced FoxA1 protein levels without affecting its mRNA levels, suggesting that SIRT6 might participate in the post-translational modification of FoxA1. To explore this possibility, co-immunoprecipitation (Co-IP) experiments were conducted to examine whether SIRT6 interacts with FoxA1 (Figure 4A). Furthermore, FoxA1 was confirmed to be among the high-confidence interacting proteins of SIRT6 identified through co-immunoprecipitation (Co-IP) coupled with mass spectrometry (see Supplementary Table 3). The results demonstrated that SIRT6 actually binds to FoxA1, which was further confirmed by immunofluorescence (IF) experiments. The colocalization quantitative analysis showed a Pearson correlation coefficient (r) of 0.57, supporting the Co-IP experiments (Figure 4B and S3A). To investigate whether SIRT6 deacetylates FoxA1, whether FoxA1 undergoes acetylation, as shown in Figure 4C, immunoprecipitation (IP) experiments confirmed the presence of acetylation on FoxA1. When SIRT6 was overexpressed or knocked down in cells, pan-acetylation antibodies were used to pull down FoxA1. The results revealed that overexpression of SIRT6 reduced the acetylation levels of FoxA1, while SIRT6 knockdown increased FoxA1 acetylation (Figure 4D-E).

To identify the specific lysine residues on FoxA1 that were deacetylated by SIRT6, two online tools were utilized, GPS-PAIL·Prediction of Acetylation on Internal Lysines (https://pail.biocuckoo.org/online.php) and PhosphoSitePlus® (https://www.phosphosite.org/homeAction.action), to predict potential acetylation sites (Figure S3B-C). Based on these predictions, five lysine residues on FoxA1 were identified that are likely to undergo acetylation: K237, K240, K264, K267, and K270 (Figure 4F). Next, lysine (K) residues on FoxA1 were mutated to glutamine (Q) and arginine (R) to simulate the processes of acetylation and non-acetylation, respectively, and constructed plasmids for FoxA1 mutants at the five identified lysine sites. To determine which mutation, affects the expression levels of the target gene TFAM, FoxA1 mutant plasmids at different sites were overexpressed in 293T cells separately. The results revealed that when FoxA1 K267 was mutated to Q267, the mRNA and protein levels of TFAM increased, whereas it decreased when mutated to R267, confirming that K267 is a critical site for FoxA1's transcriptional regulation of TFAM (Figure 4G-4H and S3D).

To investigate whether SIRT6 interacts with FoxA1 through the K267 site and performs deacetylation, Myc-His-FoxA1^K267Q^ and Myc-His-FoxA1^K267R^ were overexpressed in 293T cells and Co-IP experiments performed pull-down and reverse pull-down experiments. The results showed that when the K267 site was acetylated, it could interact with SIRT6, however, upon deacetylation, the interaction was lost (Figure 4I-J). To further confirm that the K267 site is a target for SIRT6-mediated deacetylation, Myc-His-FoxA1^K267Q^ and GFP-SIRT6 were co-overexpressed, and external Co-IP experiments demonstrated that SIRT6 indeed deacetylates FoxA1 at the K267 site. Additionally, the acetylation level of TFAM was elevated following the overexpression of Myc-His-FoxA1^K267Q^, but decreased when both Myc-His-FoxA1^K267Q^ and SIRT6 were co-overexpressed (Figure 4K). Besides, to further confirm the interaction between Myc-His-FoxA1^K267Q^ and SIRT6, IF experiments revealed that Myc-His-FoxA1^K267Q^ and SIRT6 directly interact in the nucleus (Figure 4L-N and S3E-F), however, Myc-His-FoxA1^K267R^ was localized on the nuclear membrane and did not interact with SIRT6.

Based on these findings, SIRT6 was confirmed to deacetylate FoxA1 at the K267 site, which inhibits FoxA1-mediated transcriptional activation of its target gene TFAM, ultimately impacting mitochondrial function, as depicted in the molecular mechanism diagram in Figure 4O.

### SIRT6 down-regulated the expression of mitochondrial gene and protein by binding to TFAM

**Figure 5:**
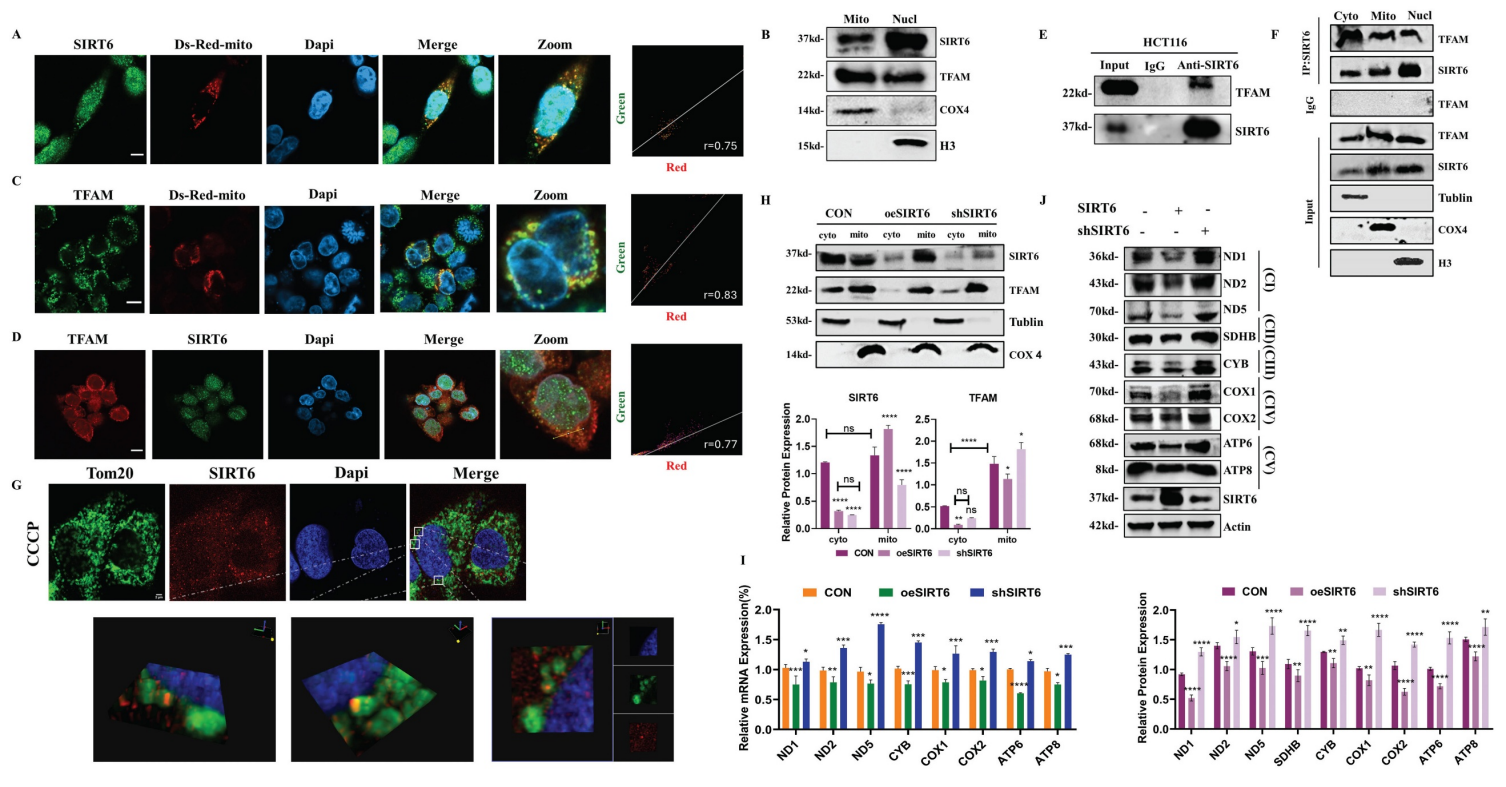
** SIRT6 translocates from the nucleus to mitochondria.** (A, C) Immunofluorescence staining of SIRT6/TFAM in HCT116 cells transfected with Ds-Red-mito plasmid, followed by confocal microscopy. Quantitative co-localization was performed using Coloc 2 in ImageJ software. Scale bar = 10 μm. (B) Western blotting of SIRT6 and TFAM protein levels after mitochondrial and nuclear separation in HCT116 cells. (D) Immunofluorescence staining of SIRT6 and TFAM in CRC cells, followed by confocal microscopy. Scale bar = 10 μm. (E) Co-IP experiments analyzing the interaction of SIRT6 and TFAM in HCT116 cells. (F) Immunoprecipitation (IP) analysis of acetylation levels of TFAM and SIRT6 following mitochondrial isolation. (G) Immunofluorescence staining of SIRT6 and Tom20 in CCCP-treated cells, followed by confocal microscopy. Scale bar = 2 μm. (H) Protein levels of SIRT6 and TFAM were measured in mitochondria- and cytoplasm-isolated samples from SIRT6-overexpressed or knockdown HCT116 cells. (I-J) mRNA and protein levels of mitochondrial proteins were detected in SIRT6-overexpressed or knockdown HCT116 cells. Error bars represent mean ± SEM. NS, not significant; statistical significance: *P < 0.05, **P < 0.01, ***P < 0.001 vs control group. Data are representative of three independent experiments.

Based on the above findings, SIRT6 likely suppressed TFAM expression by modulating FoxA1's transcriptional regulation of TFAM. However, this raises the question of whether SIRT6 also directly deacetylates TFAM, thereby affecting the transcriptional process of mitochondrial genes and, consequently, mitochondrial function. To investigate this hypothesis, HCT116 cells were transfected with Ds-Red-mito and performed immunofluorescence staining for SIRT6 to assess whether SIRT6 localized to the mitochondria. And, the colocalization quantitative analysis confirmed this, showing a Pearson correlation coefficient (r) of 0. 75, indicating that SIRT6 partially localizes to the mitochondria (Figure 5A and S4A). Furthermore, following separating mitochondrial and nuclear fractionation, Western blot analysis confirmed that SIRT6 predominantly resides in the nucleus but is also partially localized to mitochondria (Figure 5B). In contrast, TFAM localization was predominantly in the mitochondria, with a smaller fraction observed in the cytoplasm and nucleus, as determined by IF experiments (Figure 5B-C and S4B-C).

Additionally, using the STRING database, the PPI network between the SIRT family and TFAM was predicted, indicating a potential interaction between SIRT6 and TFAM (Figure S4D), which was supported by IF experiments, showing partial colocalization of SIRT6 and TFAM with a Pearson correlation coefficient (r) of 0.77 (Figure 5D and S4E). Additionally, Co-IP experiments further confirmed that SIRT6 and TFAM interact with each other (Figure 5E). Moreover, Co-IP followed by mass spectrometry analysis revealed TFAM as one of the high-confidence proteins interacting with SIRT6 (see Supplementary Table 3). To identify the specific subcellular location of this interaction, the cytosolic, mitochondrial, and nuclear fractions were separated and performed Co-IP experiments, which demonstrated that SIRT6 and TFAM interacted within the mitochondria (Figure 5F). Given that TFAM functions within the mitochondrial matrix, these findings suggest that SIRT6 may translocate from the cytoplasm to the mitochondrial matrix, where it binds to TFAM and potentially inhibits its biological function. To clearly verify whether SIRT6 can enter the mitochondrial matrix, cells were treated with CCCP to induce mitochondrial swelling. IF experiments obviously showed that SIRT6 colocalizes with the outer mitochondrial membrane and crosses it into the mitochondrial matrix (Figure 5G).

Next, by overexpressing or knocking down SIRT6, mitochondrial and cytoplasmic components were extracted separately and performed Western blot analysis, which showed that, most SIRT6 was localized in the cytoplasm under basal conditions in the control group. However, overexpression of SIRT6 promoted its translocation from the cytoplasm to the mitochondria, accompanied by a reduction in TFAM protein levels in both the mitochondrial and cytoplasmic fractions (Figure 5H). Furthermore, overexpression of SIRT6 led to a downregulation of mRNA levels of mitochondrial proteins encoded by mtDNA (Figure 5I). Western blot analysis of mitochondrial respiratory chain complex-associated proteins also revealed a significant downregulation of their expression after SIRT6 overexpression (Figure 5J).

In summary, these results suggested that SIRT6 translocates from the cytoplasm to the mitochondrial matrix, where it interacts with TFAM, which likely disrupts TFAM's transcriptional regulation of downstream mitochondrial genes, thereby impacting mitochondrial function.

### SIRT6 deacetylated the K154 site of TFAM to inhibit its transcriptional activity

**Figure 6:**
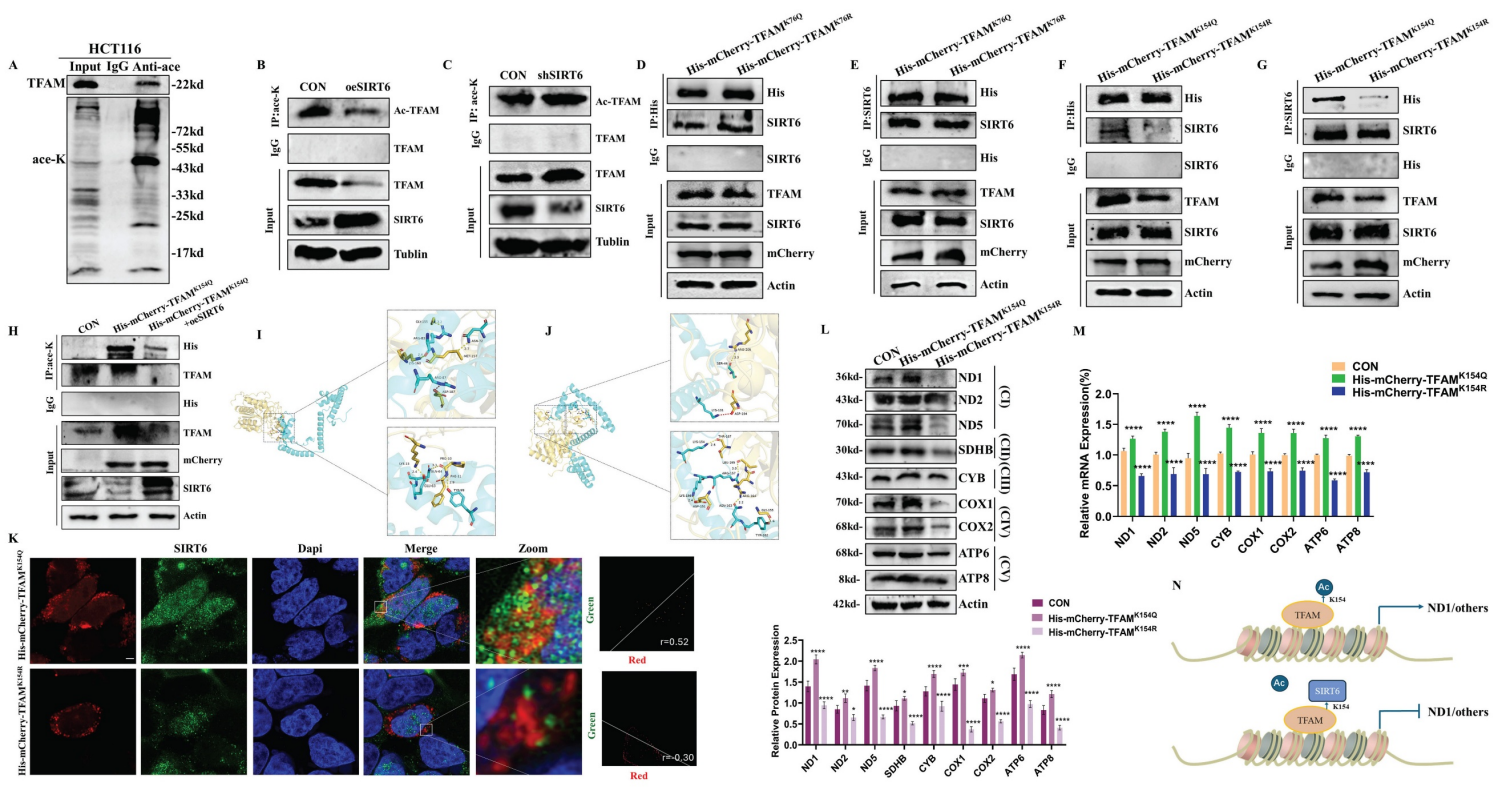
** SIRT6 deacetylates TFAM at the K154 site.** (A) Immunoprecipitation (IP) analysis of TFAM acetylation levels in HCT116 cells. (B-C) IP analysis of TFAM acetylation in HCT116 cells with overexpression or knockdown of SIRT6. (D-E) Co-immunoprecipitation (Co-IP) assays in 293T cells transfected with TFAM^K76Q^ and TFAM^K76R^, showing the interaction of SIRT6 with TFAM mutants (K76Q and K76R) using anti-SIRT6 or anti-His antibodies. (F-G) Co-IP analysis in 293T cells transfected with TFAM^K154Q^ and TFAM^K154R^ to examine the interaction between SIRT6 and TFAM^K154Q^ using anti-SIRT6 or anti-His antibodies. (H) IP analysis of acetylation levels of exogenous TFAM^K154Q^ and combination of TFAM^K154Q^ and SIRT6 in 293T cells. (I-J) Molecular docking of SIRT6 and TFAM demonstrates binding at the K154 site of TFAM. (K) Immunofluorescence co-localization analysis of SIRT6 and mCherry-tagged TFAM^K154Q^ or TFAM^K154R^ in 293T cells. Representative confocal microscopy images are shown. Scale bar = 10 μm. Quantitative analysis of co-localization was performed using Coloc 2 in ImageJ. (L-M) Protein and mRNA levels of mitochondrial proteins in 293T cells transfected with TFAM^K154Q^ and TFAM^K154R^. (N) A simplified mechanism illustrating the interaction between SIRT6 and TFAM at the K154 acetylation site. Error bars represent mean ± SEM. NS, not significant; statistical significance: *P < 0.05, **P < 0.01, ***P < 0.001 vs. control group. Data are representative of three independent experiments.

To investigate whether SIRT6 deacetylate TFAM, as shown in Figure 6A, IP experiments revealed the presence of acetylation on TFAM firstly. Additionally, the results demonstrated that overexpression of SIRT6 reduced the acetylation levels of TFAM, while the absence of SIRT6 led to increased acetylation (Figure 6B-C). To identify the acetylation sites on TFAM, online databases were used to predict potential acetylation sites. As shown in Figure S5A-B, the predicted acetylation sites were identified as K76 and K154. To determine whether SIRT6 interacts with TFAM at the K76 or K154 site and performs deacetylation, exogenous Co-IP experiments were conducted, His-mCherry-TFAM^K76Q^ and His-mCherry-TFAM^K76R^ were overexpressed in 293T cells and performed pull-down and reverse pull-down experiments. As shown in Figure 6D-E, mutations at the K76 site still kept the interaction between SIRT6 and His-tag-mutant. However, His-mCherry-TFAM^K154Q^ was able to interact with SIRT6, while His-mCherry-TFAM^K154R^ was not, which indicated that the K154 site mediates the interaction between SIRT6 and TFAM and serves as the target site for SIRT6-mediated deacetylation of TFAM (Figure 6F-G).

Next, to further confirm that SIRT6 can deacetylate the K154 site, His-mCherry-TFAM^K154Q^ and SIRT6 were co-transfected into cells and examined whether SIRT6 reduces the acetylation of His-mCherry-TFAM^K154Q^. IP experiments showed a significant reduction in acetylation levels of His-mCherry-TFAM^K154Q^ upon SIRT6 overexpression (Figure 6H). Additionally, molecular docking experiments were performed to visualize the interaction between SIRT6 and TFAM. The docking results revealed that the binding region of SIRT6 with TFAM includes the K154 site but excludes the K76 site, as shown in Figure 6I-J and S5C-D, which supported the conclusion that SIRT6 specifically targets the K154 site of TFAM for deacetylation.

Additionally, to further determine the colocalization of SIRT6 with His-mCherry-TFAM^K154Q^ and His-mCherry-TFAM^K154R^, IF experiments were conducted, which showed that SIRT6 could colocalize with His-mCherry-TFAM^K154Q^. In contrast, no colocalization was observed between SIRT6 and His-mCherry-TFAM^K154R^ (Figure 6K and S5E-F). To further confirm that deacetylation at the K154 site weakens TFAM's transcriptional regulation of mitochondrial genes and decreases their protein levels, 293T cells were transfected with His-mCherry-TFAM^K154Q^ and His-mCherry-TFAM^K154R^ separately. Immunoblotting and RT-qPCR experiments were performed to assess the protein and mRNA levels of mitochondrial respiratory chain complexes. As shown in Figure 6L-M, the results demonstrated that deacetylation of TFAM at the K154 site significantly reduced its transcriptional activation of downstream target genes. This finding underscores the critical role of K154 deacetylation in modulating TFAM's regulatory function on mitochondrial gene expression.

Taken together, these results confirmed that SIRT6 translocases into the mitochondrial matrix, where it interacts with TFAM and deacetylates its K154 residue. This modification suppresses TFAM's function as a mitochondrial transcription factor, thereby inhibiting its transcriptional activation of downstream mitochondrial genes. The molecular mechanism of this pathway is illustrated in Figure 6N.

### SIRT6 induced the progression of mitophagy

**Figure 7:**
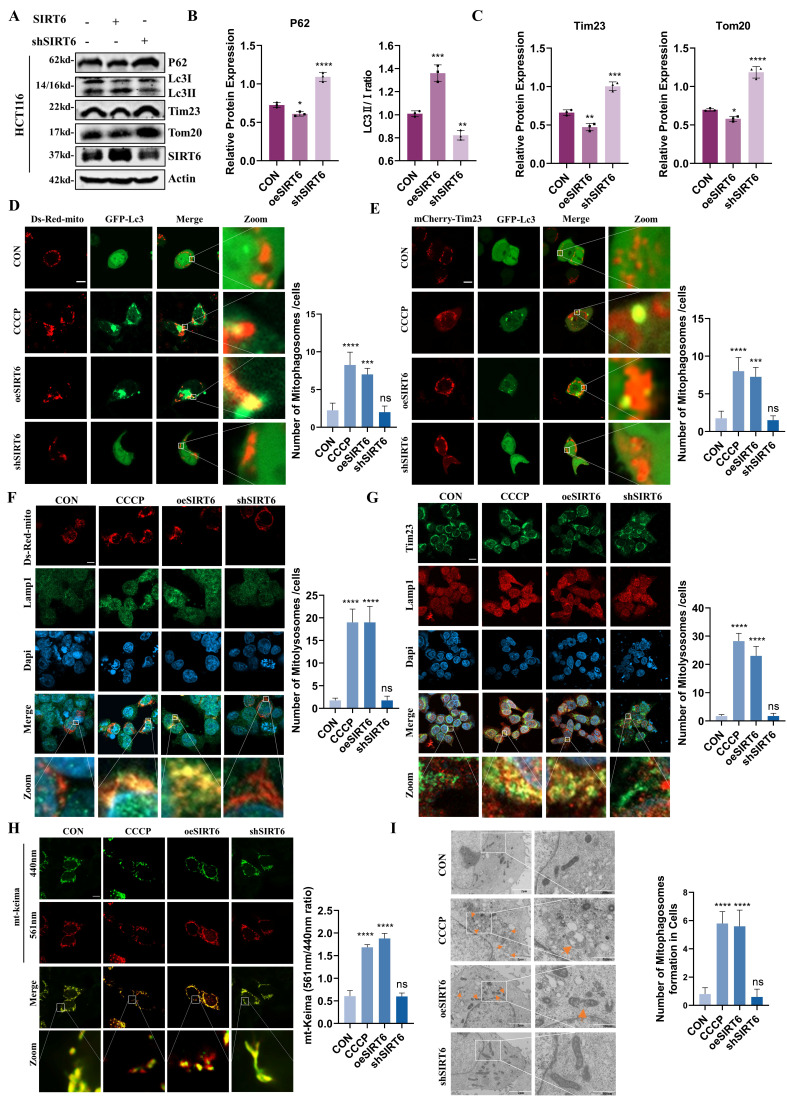
** SIRT6 activates mitophagy.** (A-C) Western blot analysis of mitophagy markers (P62, Lc3-II/I, Tim23, Tom20) in SIRT6-overexpressed or -knocked down HCT116 cells. (D-E) Confocal microscopy images showing mitophagosome formation after co-transfection of Ds-Red-mito/mCherry-Tim23 and GFP-Lc3 in HCT116 cells for 24 hours. Quantitative analysis of the number of yellow merged puncta is shown. Scale bar = 10 μm. (F-G) Confocal microscopy analysis of mitolysosomes formation after co-transfection of Ds-Red-mito and immunofluorescence staining for Lamp1 in CRC cells. Representative images and quantitative analysis of yellow dots are presented. Scale bar = 10 μm. (H) Mitophagy flux was monitored in living HCT116 cells using mito-Keima. Representative confocal images are shown. Scale bar = 10μm. Fluorescence ratio quantification is provided. (I) Electron microscopy images showing mitophagosome formation under different treatments. Scale bar = 2 μm / 500 nm Error bars represent mean ± SEM. NS, not significant; statistical significance: *P < 0.05, **P < 0.01, ***P < 0.001 vs. control group. Data are representative of three independent experiments.

Based on the above findings, it was established that SIRT6 impacts TFAM through two pathways: (1) by regulating the newly identified transcription factor FoxA1, which suppresses its transcriptional activation of TFAM; and (2) by directly binding to TFAM and deacetylating its K154 residue, thereby impairing its biological function and inhibiting the expression of mitochondrial respiratory chain complex proteins, which would made SIRT6 induce mitophagy as a compensatory pathway to eliminate damaged mitochondria. To test this hypothesis, SIRT6 was overexpressed or knocked down in HCT116 cells and performed Western blot analysis, which showed that overexpression of SIRT6 led to an upregulation of the ratio of Lc3II/Lc3I and a downregulation of P62, as well as the mitochondrial inner and outer membrane proteins Tim23 and Tom20 (Figure 7A-C). What's more, the role of FoxA1 and TFAM also was examined in modulating mitophagy, which showed that the ratio of Lc3II/Lc3I decreased, whereas P62, Tim23 and Tom20 increased in FoxA1 or TFAM overexpression group (Figure S6A-B). In addition, 293T cells were transfected with His-mCherry-TFAM^K154Q^ and His-mCherry-TFAM^K154R^ separately to investigate the impact of the K154 acetylation site on mitophagy. The results showed that that deacetylation at the K154 site promoted mitophagy. Besides, Live-cell imaging further demonstrated that the formation of mitophagosomes was enhanced in His-mCherry-TFAM^K154R^ overexpression group (Figure S6C-D).

To better understand the dynamics of mitophagy, Ds-Red-mito and GFP-Lc3 were co-transfected into cells and performed live-cell imaging to assess the colocalization of phagosomes with mitochondria, which revealed that, compared to the control group, the number of yellow puncta was significantly higher in the SIRT6 overexpression group and was similar to the CCCP treated group. In contrast, no colocalization between phagosomes and mitochondria was observed in the SIRT6 knockdown group (Figure 7D). Additionally, consistent results were observed in live-cell imaging using mCherry-Tim23 and GFP-Lc3 co-transfection (Figure 7E). To further verify the formation of mitolysosomes, cells were transfected with Ds-Red-mito and performed immunofluorescence staining for the lysosomal marker protein Lamp1, which showed that SIRT6 promoted the formation of mitolysosomes (Figure 7F). In addition, as depicted in Figure 7G, the immunofluorescence imaging results for Tim23 and Lamp1, also supported this observation. These findings indicated that SIRT6 facilitates the fusion of mitochondria with phagosomes or lysosomes, a critical step in mitophagy.

Furthermore, to investigate the impact of SIRT6 on mitophagy flux, the mitochondrial pH indicator mt-Keima was utilized for live-cell imaging, which showed that SIRT6 led to an increase in 561 nm red fluorescence excitation and a decrease in 440 nm green fluorescence excitation, which indicated an enhancement of mitophagy flux after SIRT6 overexpression (Figure 7H). Next, scanning electron microscopy was performed to assess mitochondrial morphology, which showed that mitochondria appear intact with no observable damage in the control and SIRT6 knockdown groups, but varying degrees of mitochondrial damage display and autophagosomes encapsulating damaged mitochondria or lysosomes engulfing mitochondria are observed in both the CCCP treatment group and the SIRT6 overexpression group (Figure 7I).

In summary, it was determined that SIRT6 induces mitophagy by impairing mitochondrial function through the suppression of TFAM expression and its biological activity, while, TFAM and FoxA1 played critical roles in maintaining mitochondrial function and inhibiting mitophagy.

### SIRT6 suppressed colorectal cancer cell growth and metastasis through targeting TFAM

**Figure 8:**
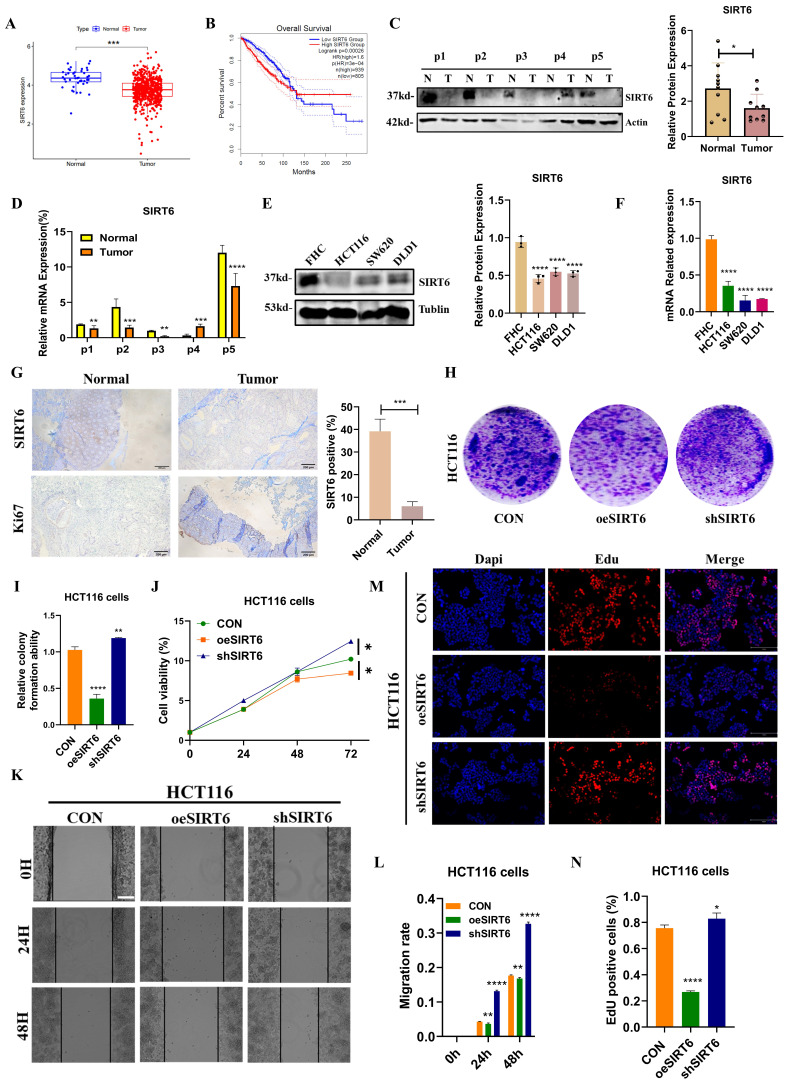
** SIRT6 inhibited the progression of CRC.** (A) Analyze the expression differences of SIRT6 in colorectal cancer tumors and adjacent tissues online using the GEPIA database. (B) Kaplan-Meier Survival Analysis of SIRT6 was acquired from the GEPIA database. (C-D) Western blotting analyzed the protein levels and mRNA levels of SIRT6 in the tumor and peritumoral tissue of 5 patients. (E-F) Western blotting analyzed the protein levels and mRNA levels of SIRT6 in human colon epithelial cells and colon cancer cells. (G) The IHC staining of SIRT6 was measured in tumor and peritumoral tissues. (H-I) The colony formation assay and quantitative analysis showed the HCT116 cells proliferation in overexpressed/knocked-down SIRT6 in HCT116 cells. (J) CCK8 showed the cell activity in overexpressed/knocked-down SIRT6 in HCT116 cells. (K-L) Scratch tests showed the cell activity in overexpressed/knocked-down SIRT6 in HCT116 cells. Scale bar = 300 μm. (M-N) Edu assay was measured cells proliferation in overexpressed/knocked-down SIRT6 in HCT116 cells. Scale bar = 150 μm. Error bars represent mean ± SEM. NS, not significant; statistical significance: * P < 0.05, ** P < 0.01, *** P < 0.001 vs control group. Data were representative of three independent experiments.

**Figure 9:**
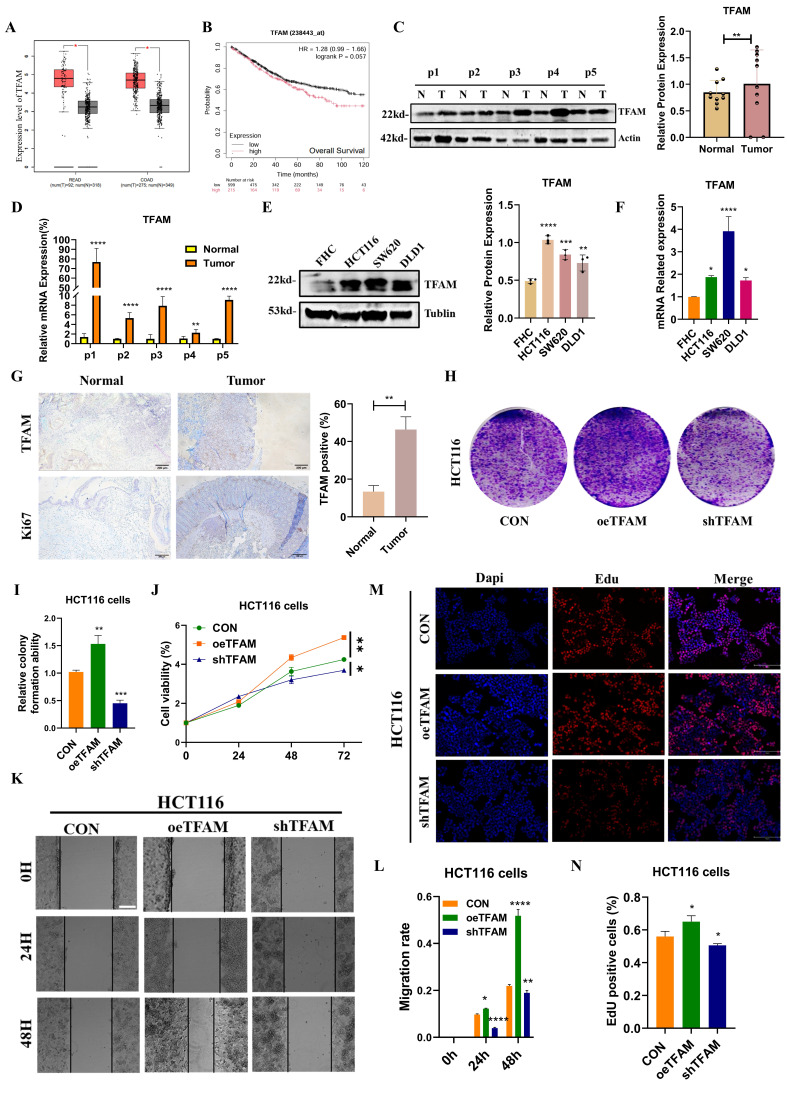
** TFAM activated the progression of CRC.** (A) Analyze the expression differences of TFAM in colorectal cancer tumors and adjacent tissues online using the GEPIA database. (B) Kaplan-Meier Survival Analysis of TFAM was acquired from the GEPIA database. (C-D) Western blotting analyzed the protein levels and mRNA levels of TFAM in the tumor and peritumoral tissue of 5 patients. (E-F) Western blotting analyzed the protein levels and mRNA levels of TFAM in human colon epithelial cells and colon cancer cells. (G) The IHC staining of TFAM was measured in tumor and peritumoral tissues. (H-I) The colony formation assay and quantitative analysis showed the HCT116 cells proliferation in overexpressed/knocked-down TFAM in HCT116 cells. (J) CCK8 showed the cell activity in overexpressed/knocked-down TFAM in HCT116 cells. (K-L) Scratch tests showed the cell activity in overexpressed/knocked-down TFAM in HCT116 cells. Scale bar = 300 μm (M-N) Edu assay was measured cells proliferation in overexpressed/knocked-down TFAM in HCT116 cells. Scale bar = 150 μm. Error bars represent mean ± SEM. NS, not significant; statistical significance: * P < 0.05, ** P < 0.01, *** P < 0.001 vs control group. Data were representative of three independent experiments.

Based on the findings described above, it could be elucidated the molecular mechanism by which SIRT6 regulates TFAM. To further explore the relationship between SIRT6 and TFAM in the context of colorectal cancer on TCGA and GEPIA databases, which revealed that SIRT6 expression is significantly lower in colorectal tumor tissues and is associated with a favorable prognosis (Figure 8A-B). But, TFAM showed the higher expression in tumor tissues compared to normal tissues and was linked to poor prognosis (Figure 9A-B). These findings were corroborated by the analysis of patient samples, which showed that both protein and mRNA levels of SIRT6 were markedly higher in adjacent non-tumor tissues compared to tumor tissues, but TFAM was significantly lower in adjacent non-tumor tissues than in tumor tissues (Figure 8C-D and 9C-D). Similar trends were observed in colorectal cancer cell lines (Figure 8E-F and 9E-F). Additionally, immunohistochemical analysis of patient tissue samples revealed more positive staining for SIRT6 in normal tissue sections, while TFAM exhibited more intense staining in tumor tissues, consistent with the quantitative analysis (Figure 8G and 9G).

To further assess the role of SIRT6 and TFAM in colorectal cancer cell proliferation and survival, the crystal violet assay confirmed these findings, showing that SIRT6 overexpression inhibits cell growth, while TFAM overexpression enhances it (Figure 8H-I and 9H-I). Similarly, CCK8 assays was performed, which indicated that SIRT6 inhibits the growth of HCT116 cells, whereas TFAM promotes their growth (Figure 8J and 9J). Further, SIRT6 and TFAM were overexpressed or knocked down in HCT116 cells, and scratch assays and EDU cell proliferation assays demonstrated that SIRT6 inhibits both the migration and proliferation of colorectal cancer cells, while TFAM promotes these processes (Figure 8K-N and 9K-N).

Taken together, it provided strong evidence that SIRT6 and TFAM play critical roles in the development and progression of colorectal cancer. It proposes that SIRT6 regulates TFAM, leading to mitochondrial dysfunction and the induction of mitophagy, which suppresses tumor progression and may provide a potential therapeutic strategy for targeting colorectal cancer.

### *In vivo* validation of SIRT6-mediated regulation of TFAM in colorectal cancer xenograft models

**Figure 10:**
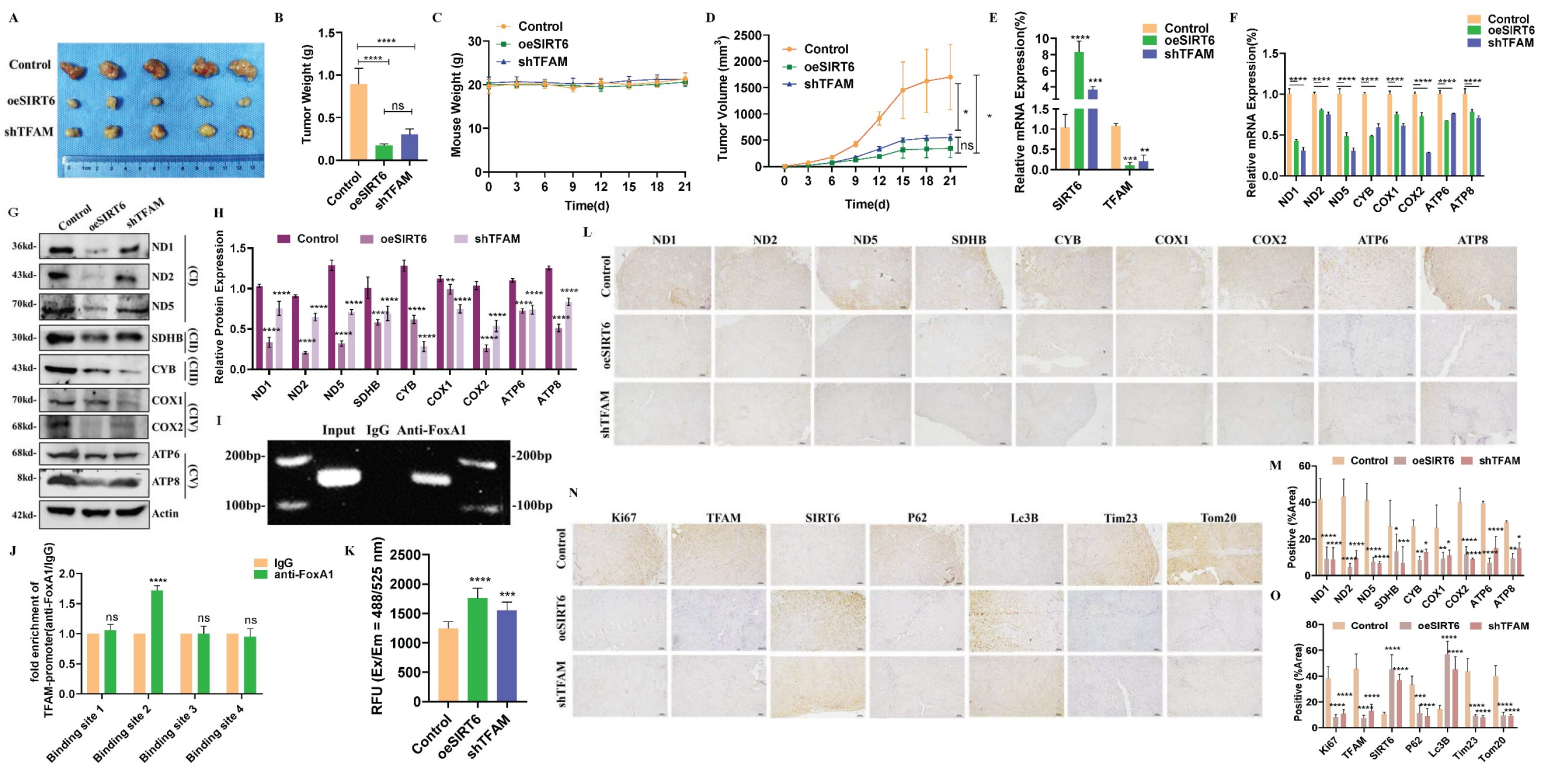
** Animal experiments were conducted to validate the findings.** (A) Nude mice were used to establish a xenograft model with control, oeSIRT6 and shTFAM tumor tissues. Representative images of tumors of oeSIRT6 and shTFAM group. (B-D) Tumor weight, mouse weight and tumor volume were measured. (E-F) RT-qPCR analyzed the mRNA levels of SIRT6, TFAM and mitochondrial protein in mice tumor tissues. (G-H) Western blotting analyzed the expression of mitochondrial protein of mice tumors. (I-J) Using ChIP assays determined the binding site of FoxA1 interact with TFAM promoter in mice tumor. ChIP-seq detected by RT-qPCR. (K) ROS was detected in mice tumor tissues. (L-M) Representative data from IHC staining of mitochondrial respiratory chain complexes. Scale bar = 100 μm. (N-O) Representative data from IHC staining of mitophagy marker proteins in mice tumor tissues. Scale bar = 100 μm. Error bars represent mean ± SEM. NS, not significant; statistical significance: * P < 0.05, ** P < 0.01, *** P < 0.001 vs control group. Data were representative of three independent experiments.

To validate the molecular mechanism by which SIRT6 regulates TFAM transcription and affects the expression of TFAM's downstream target genes *in vivo*, stable colorectal cancer cell lines with SIRT6 overexpression and TFAM knockdown were constructed, which were subcutaneously injected into nude mice to establish xenograft tumor models. After three weeks, tumors were excised and compared among the groups. Both the SIRT6 overexpression group and the TFAM knockdown group showed significantly smaller tumor volumes compared to the control group (Figure 10A).

Additionally, the weight of the tumors in the SIRT6 overexpression and TFAM knockdown groups were lighter than in the control group, while the body weights of the mice remained unchanged across all groups (Figure 10B-C). Tumor growth trend analysis further confirmed that SIRT6 overexpression and TFAM knockdown inhibited and slowed tumor growth (Figure 10D).

To verify the successful establishment of gene expression in the constructed cell lines within the tumors, RT-qPCR was performed, which confirmed that mRNA levels of SIRT6 were upregulated to varying degrees in both the SIRT6 overexpression and TFAM knockdown groups, while the mRNA levels of TFAM were downregulated (Figure 10E). Additionally, the mRNA levels of mitochondrial respiratory chain complex genes encoded by mtDNA were assessed, confirming that SIRT6 overexpression significantly downregulated these genes, with effects similar to those observed in the TFAM knockdown group (Figure 10F). Furthermore, the results of Western blotting indicated that the protein levels of mitochondrial respiratory chain complex genes were consistently decreased in both the SIRT6 overexpression and TFAM knockdown groups (Figure 10G-H).

ChIP experiments further demonstrated that FoxA1 binds to the promoter region of TFAM, specifically through the BS2 sequence, supporting the transcriptional regulation mechanism (Figure 10I-J). Moreover, ROS detection in tumor tissues revealed that both the SIRT6 overexpression and TFAM knockdown groups induced oxidative stress (Figure 10K) Immunohistochemistry (IHC) analysis of corresponding tumor tissue sections supported these findings, showing reduced positive staining for related proteins compared to the control group, consistent with the results in Figure 8G (Figure 10L-M). Subsequently, IHC analysis was performed to detect autophagy-related markers, P62 and Lc3B, and mitochondrial proteins, Tim23 and Tom20, demonstrating that SIRT6 overexpression and TFAM knockdown indeed induced the progression of mitophagy (Figure 10N-O). These findings were further supported by Western blotting results, which showed consistent evidence of mitophagy induction in Figure 10N (Figure S7A).

In conclusion, the results demonstrated that SIRT6 inhibits TFAM, leading to mitochondrial dysfunction and inducing mitophagy, ultimately suppressing colorectal cancer growth, which were validated *in vivo* using a mouse xenograft tumor model.

## Discussion

The human SIRT family, consisting of recognized members SIRT1-SIRT7, represents a group of highly conserved deacetylases involved in a wide range of biological processes and the pathogenesis of various diseases. These proteins are considered potential therapeutic targets for numerous diseases, including cancer, cardiovascular diseases, and respiratory disorders. Among these, SIRT6 has been extensively studied for its role in regulating the molecular mechanisms of aging^45^.

Early studies about SIRT6 have focused on the mechanisms of mitochondrial dysfunction related to aging, metabolism, and cardiovascular diseases. SIRT6 activation protects against doxorubicin-induced cardiotoxicity by enhancing mitochondrial biogenesis and mitophagy, while simultaneously promoting doxorubicin cytotoxicity in cardiomyocytes through metabolic remodeling toward mitochondrial respiration^46^. However, whether SIRT6 inhibits tumorigenesis through regulation of mitochondrial function remains unclear and lacks systematic investigation. Tumor growth is often accelerated due to the high mitochondrial energy demands of the tumor microenvironment. Therefore, it is crucial to explore whether the inhibition of CRC progression by SIRT6 is linked to its ability to disrupt mitochondrial function in CRC cells. However, few studies have investigated whether—and how—SIRT6 influences mitochondrial function in tumor systems. Based on this, the hypothesis was proposed that SIRT6 may inhibit the energy supply from mitochondria to tumors, thereby exerting therapeutic effects. To test this hypothesis, the study investigated whether SIRT6 suppresses the development and progression of CRC by inducing mitochondrial dysfunction.

Bioinformatics analyses, including GO and KEGG pathway analysis, suggested that SIRT6 is associated with processes related to the mitochondrial respiratory chain, oxidative phosphorylation, mitochondrial gene expression, and mitophagy. Indeed, after overexpression or knockdown of SIRT6, cellular ATP levels, mitochondrial membrane potential, ROS production, mtDNA copy number, and respiratory chain functionality were assessed, which consistently indicated that SIRT6 leads to mitochondrial dysfunction (Figure 1). This result starkly contrasts with the enhanced mitochondrial stability mediated by SIRT6 in cardiomyocytes, underscoring the essential role of SIRT6 in sustaining human health.

To explore how SIRT6 induces mitochondrial dysfunction, by intersecting mitochondrial-related genes from existing mitochondrial databases with SIRT6-correlated genes acquired from the CRC database, TFAM as a common target was identified. However, studies have highlighted those members of the SIRT family, including SIRT1 and SIRT3, can directly or indirectly regulate TFAM, resulting in downstream mitochondrial functional changes^47^, ^48^. Currently, no studies have been conducted on the relationship between SIRT6 and TFAM.

Western blotting results further confirmed that SIRT6 negatively regulates TFAM expression, consistent with the Pearson correlation coefficient obtained from bioinformatics analysis. Next, both the transcriptional and protein levels of TFAM also was observed (Figure 2A-D). Based on these findings, two potential mechanisms by which SIRT6 influences TFAM and induces mitochondrial dysfunction were proposed: (1) SIRT6 may regulate TFAM transcription by modulating transcription factors that target TFAM, and (2) SIRT6 may directly deacetylate TFAM, altering its function. Through these two pathways, SIRT6 could impair mitochondrial functionality.

By utilizing four transcription factor databases, potential transcription factors were identified that may regulate TFAM. The intersection of these results suggested that FoxA1 and YY1 could be key transcription factors regulating TFAM. After investigating the influence and regulation of SIRT6 on both of these factors, it found that SIRT6 likely modulates FoxA1, thereby influencing the expression of its target gene, TFAM (Figure 2E-K and S1). Interestingly, no previous studies have reported that FoxA1 directly binds to the TFAM promoter to transcriptionally activate TFAM. To explore this, potential binding sites on the TFAM promoter where FoxA1 could interact with were predicted. Based on these predictions, ChIP experiments, dual-luciferase reporter assays, and ESMA *in vitro* validation experiments were designed. Ultimately, our results confirmed that FoxA1 binds to the TFAM promoter at the BS2 (TGTTTATTCTAC), activating TFAM transcription (Figure 3). Furthermore, the results from the ChIP experiment in animal tissues (*in vivo*) also corroborated this conclusion (Figure 10I-J).

After clarifying the regulatory role of FoxA1 on TFAM, the next question was how SIRT6 regulates FoxA1. Co-IP and IP experiments were designed, and the results confirmed that SIRT6 interacts with FoxA1, and FoxA1 undergoes acetylation. Next, an online prediction tool was used to identify potential acetylation sites on FoxA1. Based on this prediction, SIRT6 could deacetylate FoxA1 at specific sites was determined, which showed that SIRT6-induced deacetylation of FoxA1 at the K267 site inhibited the transcriptional activation of TFAM (Figure 4).

In the second hypothesis, the hypothesis was proposed that SIRT6 may enter the mitochondria, bind to TFAM, and perform deacetylation modification, thereby affecting mitochondrial function. According to current mainstream views, SIRT1, SIRT6, and SIRT7 are primarily localized in the nucleus, while SIRT3, SIRT4, and SIRT5 are found in the mitochondria, and SIRT2 is distributed in the cytoplasm. Previous reports have shown that SIRT3, localized in the mitochondria, can bind to and deacetylate the K154 site of TFAM, inducing mitophagy^47^. However, no studies have reported that SIRT6 can bind to TFAM in the mitochondrial matrix or perform deacetylation modification. Therefore, an in-depth study of SIRT6 localization was conducted, transfecting mitochondria-targeting plasmids into cells and performing immunofluorescence for SIRT6, which revealed that SIRT6 is predominantly localized in the nucleus, partially in the cytoplasm, and to a lesser extent in the mitochondria. Further, after separating mitochondria, cytoplasm, and the nucleus, Western blotting experiments confirmed that SIRT6 is distributed throughout the cell. Co-IP and IF experiments also demonstrated that SIRT6 can bind to TFAM in the mitochondria (Figure 5A-F).

To better observe the entry of SIRT6 into the mitochondria, cells were treated with the mitophagy activator CCCP and performed immunofluorescence staining with Tom20 and SIRT6, which showed SIRT6 co-localized with the outer mitochondrial membrane, crossed the outer membrane, and entered the mitochondrial matrix (Figure 5G). These findings suggested that SIRT6 indeed enters the mitochondria. Furthermore, the overexpression of SIRT6 can downregulate the mitochondrial proteins targeted by TFAM's transcription, supporting the hypothesis that SIRT6 may deacetylate TFAM and inhibit its transcriptional activity (Figure 5H-J). Through online predictions, potential acetylation sites on TFAM were identified, specifically K76 and K154. Using Co-IP and IF experiments, the K154 site was confirmed that can be deacetylated by SIRT6 (Figure 6). This finding is consistent with previously published research that mentions the deacetylation of TFAM's K154 site by SIRT3 in acute kidney injury, which mean that K154 site takes a critical role in its transcriptional activity.

After SIRT6 induced mitochondrial dysfunction in CRC cells, the mitochondria are no longer able to properly supply energy to meet the tumor's needs. This led to the process where the mitochondria are engulfed by autophagosomes and lysosomes, resulting in the occurrence of mitophagy. This conclusion was further confirmed by using mitophagy indicator mt-Keima and electron microscopy (Figure 7). Additionally, this study unexpectedly found that knockdown of FoxA1 promoted mitophagy, albeit with modest changes at the protein level as assessed by Western blotting. Currently, no studies have been reported on the relationship between FoxA1 and mitophagy, warranting further investigation.

## Conclusion

In conclusion, our study reveals that SIRT6 plays a crucial role in regulating mitochondrial function and mitophagy in colorectal cancer. SIRT6 inhibits the transcriptional activity of TFAM by both deacetylating TFAM and regulating its expression through transcription factors like FoxA1. This deacetylation disrupts mitochondrial function, leading to energy deficiencies in tumor cells. Consequently, mitochondrial damage triggered mitophagy. These findings suggested that SIRT6's modulation of mitochondrial function can serve as a potential therapeutic target for CRC by inducing mitophagy and impairing the tumor's energy supply. Our research aims to clarify the core role of SIRT6 in metabolic regulation in colorectal cancer, uncover its anti-cancer mechanisms, identifying TFAM as a promising therapeutic target and provide theoretical support for developing targeted therapeutic strategies based on SIRT6.

## Supplementary Material

Supplementary figures and tables.

## Figures and Tables

**Table 1:** The sequences of primers used for polymerase chain reaction (PCR) amplification.

Gene	Forward primer	Reverse primer
TFAM	TGATTCACCGCAGGAAAAGC	CCTAACTGGTTTCCTGTGCCT
SIRT6	CCGACTTCAGGGGTCC	GCACATTCTTCCACAAACAT
FoxA1	GAAGACCGGCCAGCTAGAG	TTTGCACTGGGGGAAAGGTT
YY1	TCAGACAAGTCACGTCAGGC	CTCCATGTCACCTCCCAC
ND1	TGGCTCCTTTAACCTCTCCA	GGCGTATTCGATGTTGAAGC
ND2	ATTTCCTCACGCAAGCAACC	CCTTGGGTAACCTCTGGGAC
ND5	GCCCAATTAGGTCTCCACCC	GCAGGAATGCTAGGTGTGGT
CYB	CCTAGCAACACTCCACCTCC	TGTTAGGGACGGATCGGAGA
COX1	CCTACTCCTGCTCGCATCTG	AGAGGGGCGTTTGGTATTGG
COX2	AACGATCCCTCCCTTACCAT	TCGATTGTCAACGTCAAGGA
ATP6	GCGCCACCCTAGCAATATCA	AGGCTTGGATTAAGGCGACA
ATP8	ATGGCCCACCATAATTACCC	GCAATGAATGAAGCGAACAG

**Table 2:** Primer sequences for the predicted FoxA1 binding sites interacting with TFAM.

Gene	Forward primer	Reverse primer
BS1	AAAGGGAAGTGGTTATTACC	TTGGATAGCCGTAATTGTTAG
BS2	AACTAGCCAGTTTCCTCTG	TTCCCCAGAATTTAACAAGTTC
BS3	CTTGTTAAATTCTGGGGAACT	GCTCGGAGTTCAGAAATAG
BS4	GCTCCAGCCCTGGCTTGAA	CTCACCCCAACCCGGCGTT

**Table 3:** Primer sequences for site-directed mutagenesis.

Plasmid	Forward primer	Reverse primer
FoxA1^K237Q^	gcacgctccccggacCagccgggcaagggctcctactggacgctgc	agcccttgcccggctGgtccggggagcgtgccaccttgacgaagca
FoxA1^K237R^	cacgctccccggacaGgccgggcaagggctcctactggacgctgca	gagcccttgcccggcCtgtccggggagcgtgccaccttgacgaagc
FoxA1^K240Q^	ccggacaagccgggcCagggctcctactggacgctgcacccggact	tccagtaggagccctGgcccggcttgtccggggagcgtgccacctt
FoxA1^K240R^	cggacaagccgggcaGgggctcctactggacgctgcacccggactc	gtccagtaggagcccCtgcccggcttgtccggggagcgtgccacct
FoxA1^K264Q^	tacttgcgccgccagCagcgcttcaagtgcgagaagcagccgggggc	cgcacttgaagcgctGctggcggcgcaagtagcagccgttctcgaa
FoxA1^K264R^	acttgcgccgccagaGgcgcttcaagtgcgagaagcagccgggggc	tcgcacttgaagcgcCtctggcggcgcaagtagcagccgttctcga
FoxA1^K267Q^	cgccagaagcgcttcCagtgcgagaagcagccgggggccggcggcg	gctgcttctcgcactGgaagcgcttctggcggcgcaagtagcagcc
FoxA1^K267R^	gccagaagcgcttcaGgtgcgagaagcagccgggggccggcggcgg	ggctgcttctcgcacCtgaagcgcttctggcggcgcaagtagcagc
FoxA1^K270Q^	cgcttcaagtgcgagCagcagccgggggccggcggcgggggcggga	cggcccccggctgctGctcgcacttgaagcgcttctggcggcgcaa
FoxA1^K270R^	gcttcaagtgcgagaGgcagccgggggccggcggcgggggcgggag	ccggcccccggctgcCtctcgcacttgaagcgcttctggcggcgca
TFAM^K76Q^	agaacccagatgcaCaaactacagaactaattagaagaattgcc	ttagttctgtagtttGtgcatctgggttctgagctttaaatatg
TFAM^K76R^	agaacccagatgcaaGaactacagaactaattagaagaattgc	attagttctgtagttCttgcatctgggttctgagctttaaatatg
TFAM^K154Q^	ttaacactgcttggaCaaccaaaaagacctcgttcagcttataac	gaggtctttttggttGtccaagcagtgttaactcttttttttttg
TFAM^K154R^	taacactgcttggaaGaccaaaaagacctcgttcagcttataacg	cgaggtctttttggtCttccaagcagtgttaactctttttttttt

## Abbreviations

SIRT6: NAD-dependent protein deacylase sirtuin-6
TFAM: Transcription factor A, mitochondrial
FoxA1: Hepatocyte nuclear factor 3-alpha
mtDNA: Mitochondrial DNA
CCCP: Carbonyl cyanide 3-chlorophenylhydrazone
FCCP: Carbonyl cyanide 4-(trifluoromethoxy) phenylhydrazone
Tim23: Translocase of inner mitochondrial membrane 23 homolog
Tom20: Translocase of outer mitochondrial membrane 20 homolog
Lamp1: Lysosome-associated membrane protein 1

## References

[1] Cobo I, Tanaka TN, Chandra Mangalhara K, Lana A, Yeang C, Han C (2022). DNA methyltransferase 3 alpha and TET methylcytosine dioxygenase 2 restrain mitochondrial DNA-mediated interferon signaling in macrophages. Immunity.

[2] Li B, Liu F, Chen X, Chen T, Zhang J, Liu Y (2024). FARS2 Deficiency Causes Cardiomyopathy by Disrupting Mitochondrial Homeostasis and the Mitochondrial Quality Control System. Circulation.

[3] Zong Y, Li H, Liao P, Chen L, Pan Y, Zheng Y (2024). Mitochondrial dysfunction: mechanisms and advances in therapy. Signal transduction and targeted therapy.

[4] Zhang W, Liu D, Yuan M, Zhu LQ (2024). The mechanisms of mitochondrial abnormalities that contribute to sleep disorders and related neurodegenerative diseases. Ageing Res Rev.

[5] Al-Kuraishy HM, Jabir MS, Albuhadily AK, Al-Gareeb AI, Jawad SF, Swelum AA (2024). Role of ketogenic diet in neurodegenerative diseases focusing on Alzheimer diseases: The guardian angle. Ageing Res Rev.

[6] Suomalainen A, Nunnari J (2024). Mitochondria at the crossroads of health and disease. Cell.

[7] Headley CA, Gautam S, Olmo-Fontanez A, Garcia-Vilanova A, Dwivedi V, Akhter A (2024). Extracellular Delivery of Functional Mitochondria Rescues the Dysfunction of CD4(+) T Cells in Aging. Advanced science (Weinheim, Baden-Wurttemberg, Germany).

[8] Xiao X, Li R, Cui B, Lv C, Zhang Y, Zheng J (2024). Liver ACSM3 deficiency mediates metabolic syndrome via a lauric acid-HNF4α-p38 MAPK axis. Embo j.

[9] Chella Krishnan K, Vergnes L, Acín-Pérez R, Stiles L, Shum M, Ma L (2021). Sex-specific genetic regulation of adipose mitochondria and metabolic syndrome by Ndufv2. Nat Metab.

[10] Patel SG, Dominitz JA (2024). Screening for Colorectal Cancer. Annals of internal medicine.

[11] Lu T, Zhang Z, Bi Z, Lan T, Zeng H, Liu Y (2023). TFAM deficiency in dendritic cells leads to mitochondrial dysfunction and enhanced antitumor immunity through cGAS-STING pathway. J Immunother Cancer.

[12] Kuhl I, Kukat C, Ruzzenente B, Milenkovic D, Mourier A, Miranda M (2014). POLRMT does not transcribe nuclear genes. Nature.

[13] Peng B, Wang Y, Zhang H Mitonuclear Communication in Stem Cell Function. Cell proliferation. 2024: e13796.

[14] Rubalcava-Gracia D, Garcia-Villegas R, Larsson NG (2023). No role for nuclear transcription regulators in mammalian mitochondria?. Mol Cell.

[15] Chen Y, Tang Y, Luo S, Jia H, Xu Q, Chang R (2021). Nuclear factor erythroid 2-related factor 2 protects bovine mammary epithelial cells against free fatty acid-induced mitochondrial dysfunction in vitro. J Dairy Sci.

[16] Blesa JR, Prieto-Ruiz JA, Abraham BA, Harrison BL, Hegde AA, Hernández-Yago J (2008). NRF-1 is the major transcription factor regulating the expression of the human TOMM34 gene. Biochem Cell Biol.

[17] Zhang XY, Khan S, Jiang H, Antonyak MA, Chen X, Spiegelman NA (2016). Identifying the functional contribution of the defatty-acylase activity of SIRT6. Nature Chemical Biology.

[18] Mao ZY, Hine C, Tian X, Van Meter M, Au M, Vaidya A (2011). SIRT6 Promotes DNA Repair Under Stress by Activating PARP1. Science.

[19] Kawahara TLA, Michishita E, Adler AS, Damian M, Berber E, Lin M (2009). SIRT6 Links Histone H3 Lysine 9 Deacetylation to NF-κB-Dependent Gene Expression and Organismal Life Span. Cell.

[20] Sebastián C, Zwaans BMM, Silberman DM, Gymrek M, Goren A, Zhong L (2012). The Histone Deacetylase SIRT6 Is a Tumor Suppressor that Controls Cancer Metabolism. Cell.

[21] Jiang H, Khan S, Wang Y, Charron G, He B, Sebastian C (2013). SIRT6 regulates TNF-α secretion through hydrolysis of long-chain fatty acyl lysine. Nature.

[22] Peng KZ, Zeng CY, Gao YQ, Liu BL, Li LY, Xu K (2023). Overexpressed SIRT6 ameliorates doxorubicin-induced cardiotoxicity and potentiates the therapeutic efficacy through metabolic remodeling. Acta Pharmacol Sin B.

[23] Song MY, Han CY, Moon YJ, Lee JH, Bae EJ, Park BH (2022). Sirt6 reprograms myofibers to oxidative type through CREB-dependent Sox6 suppression. Nature communications.

[24] Smirnov D, Eremenko E, Stein D, Kaluski S, Jasinska W, Cosentino C (2023). SIRT6 is a key regulator of mitochondrial function in the brain. Cell Death Dis.

[25] Zhang S, Jiang S, Wang H, Di W, Deng C, Jin Z (2018). SIRT6 protects against hepatic ischemia/reperfusion injury by inhibiting apoptosis and autophagy related cell death. Free Radic Biol Med.

[26] Pulipaka S, Singuru G, Sahoo S, Shaikh A, Thennati R, Kotamraju S (2024). Therapeutic efficacies of mitochondria-targeted esculetin and metformin in the improvement of age-associated atherosclerosis via regulating AMPK activation. GeroScience.

[27] Zhang Y, Nie L, Xu K, Fu Y, Zhong J, Gu K (2019). SIRT6, a novel direct transcriptional target of FoxO3a, mediates colon cancer therapy. Theranostics.

[28] Wu Z, Hong L, Luo G, Lu S, Li Y, Wang J (2023). SIRT6 promotes autophagy through direct interaction with ULK1 and competitive binding to PUMA. Genes & diseases.

[29] Hao Z, Wu T, Cui X, Zhu P, Tan C, Dou X (2020). N(6)-Deoxyadenosine Methylation in Mammalian Mitochondrial DNA. Mol Cell.

[30] Kukat C, Davies KM, Wurm CA, Spahr H, Bonekamp NA, Kuhl I (2015). Cross-strand binding of TFAM to a single mtDNA molecule forms the mitochondrial nucleoid. Proc Natl Acad Sci U S A.

[31] Kasashima K, Sumitani M, Endo H (2012). Maintenance of mitochondrial genome distribution by mitochondrial AAA+ protein ClpX. Exp Cell Res.

[32] Cell Biology of the Mitochondrion Genetics. 2018; 208: 1673.

[33] Huang QC, Li JB, Xing JL, Li WW, Li HW, Ke X (2014). CD147 promotes reprogramming of glucose metabolism and cell proliferation in HCC cells by inhibiting the p53-dependent signaling pathway. Journal of Hepatology.

[34] Yang S, He X, Zhao J, Wang D, Guo S, Gao T (2021). Mitochondrial transcription factor A plays opposite roles in the initiation and progression of colitis-associated cancer. Cancer communications (London, England).

[35] Wen YA, Xiong X, Scott T, Li AT, Wang C, Weiss HL (2019). The mitochondrial retrograde signaling regulates Wnt signaling to promote tumorigenesis in colon cancer. Cell Death Differ.

[36] Liu Y, Jin M, Wang Y, Zhu J, Tan R, Zhao J (2020). MCU-induced mitochondrial calcium uptake promotes mitochondrial biogenesis and colorectal cancer growth. Signal transduction and targeted therapy.

[37] Song J, Ham J, Park W, Song G, Lim W (2024). Osthole impairs mitochondrial metabolism and the autophagic flux in colorectal cancer. Phytomedicine.

[38] Lu Y, Wei W, Li M, Chen D, Li W, Hu Q (2024). The USP11/Nrf2 positive feedback loop promotes colorectal cancer progression by inhibiting mitochondrial apoptosis. Cell Death Dis.

[39] Ren M, Liang S, Lin S, Huang R, Chen Y, Zhang Y (2024). Design, synthesis and biological evaluation of artesunate-Se derivatives as anticancer agents by inducing GPX4-mediated ferroptosis. Bioorganic chemistry.

[40] Hu H, Zhang F, Sheng Z, Tian S, Li G, Tang S (2024). Synthesis and mitochondria-localized iridium (III) complexes induce cell death through pyroptosis and ferroptosis pathways. Eur J Med Chem.

[41] Jiang Y, Gao S, Sun H, Wu X, Gu J, Wu H (2024). Targeting NEDD8 suppresses surgical stress-facilitated metastasis of colon cancer via restraining regulatory T cells. Cell Death Dis.

[42] Wei Y, Xiao G, Xu H, Sun X, Shi Y, Wang F (2023). Radiation resistance of cancer cells caused by mitochondrial dysfunction depends on SIRT3-mediated mitophagy. Febs j.

[43] Soltész B, Urbancsek R, Pös O, Hajas O, Forgács IN, Szilágyi E (2019). Quantification of peripheral whole blood, cell-free plasma and exosome encapsulated mitochondrial DNA copy numbers in patients with atrial fibrillation. J Biotechnol.

[44] Mokashi A, Bhatia NM (2024). Integrated Network Ethnopharmacology, Molecular Docking, and ADMET Analysis Strategy for Exploring the Anti-Breast Cancer Activity of Ayurvedic Botanicals Targeting the Progesterone Receptor. BIOI.

[45] Ramteke P, Watson B, Toci M, Tran VA, Johnston S, Tsingas M (2025). Sirt6 deficiency promotes senescence and age-associated intervertebral disc degeneration in mice. Bone Res.

[46] Peng K, Zeng C, Gao Y, Liu B, Li L, Xu K (2023). Overexpressed SIRT6 ameliorates doxorubicin-induced cardiotoxicity and potentiates the therapeutic efficacy through metabolic remodeling. Acta Pharm Sin B.

[47] Deng ZY, He M, Hu HB, Zhang WQ, Zhang YY, Ge Y (2023). Melatonin attenuates sepsis-induced acute kidney injury by promoting mitophagy through SIRT3-mediated TFAM deacetylation. Autophagy.

[48] Chen L, Tang J, Zuo X, Li B, Liu C, Hong S (2025). SIRT1 Alleviates Oxidative Stress-Induced Mitochondrial Dysfunction and Mitochondria-Associated Membrane Dysregulation in Stress Urinary Incontinence. Cell Prolif.

