# Supplementary material for: SIRT6-Mediated Regulation of TFAM: A Central Mechanism Connecting Nuclear and Mitochondrial Transcriptional Processes and Mitophagy
Source: Int J Biol Sci. 2026 Jan 1;22(1):178–200. doi: 10.7150/ijbs.120007 (PMC12681845; doi:10.7150/ijbs.120007)
Supplement: Supplementary file 1 — Supplementary figures and tables. [file ijbsv22p0178s1.pdf]

Supplemental Figure 1

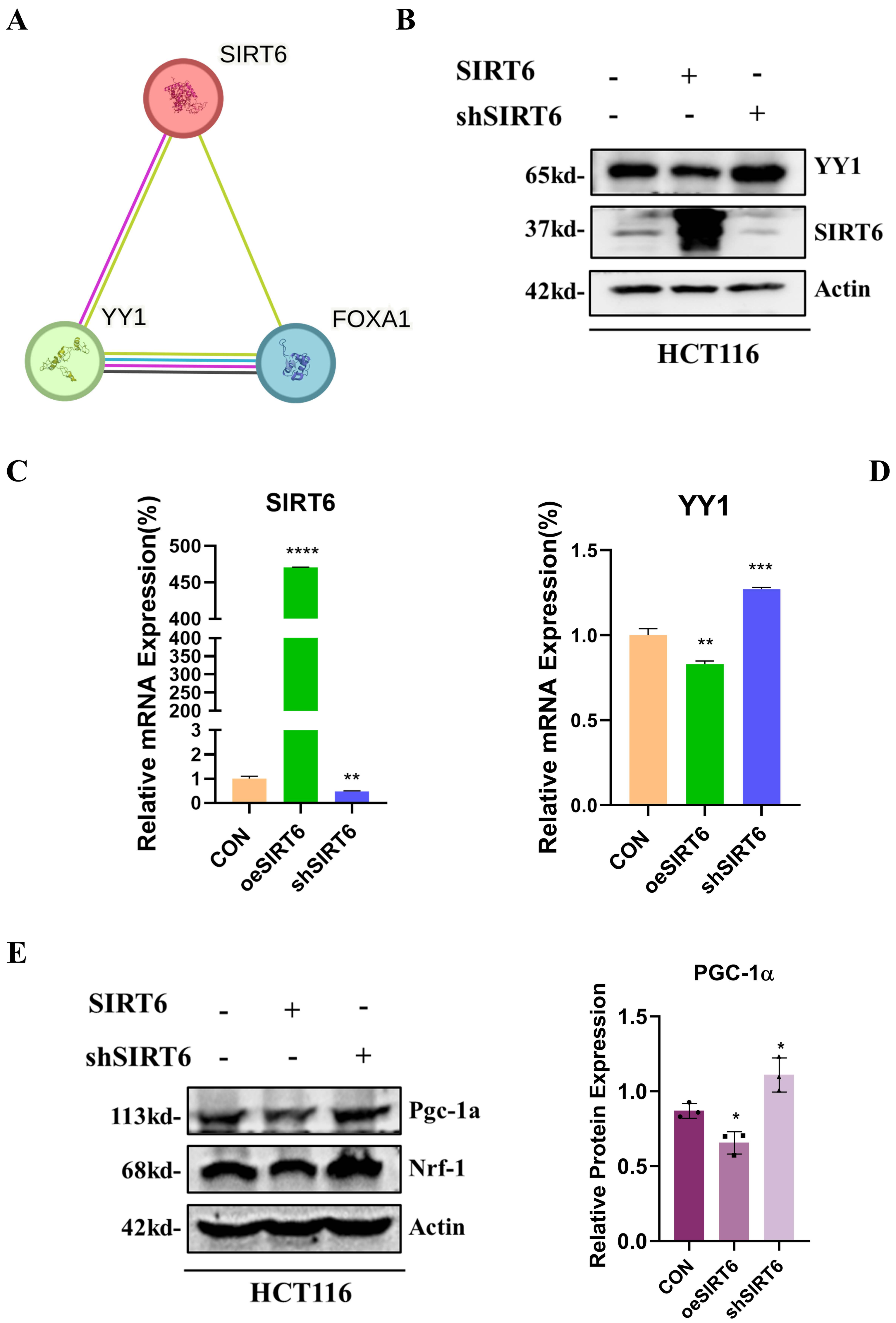

Supplemental Figure 2

A

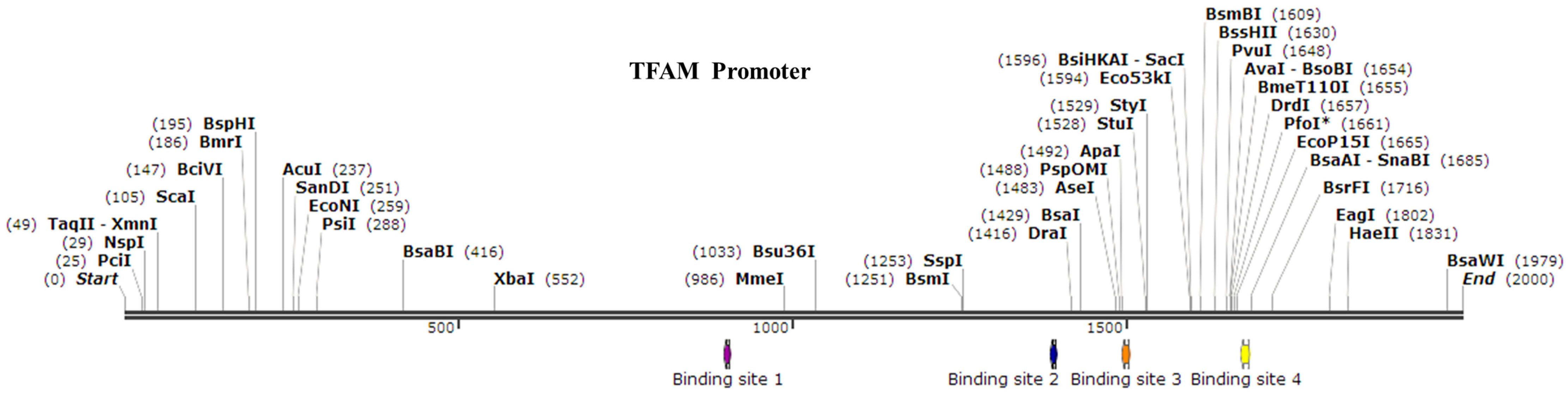

B

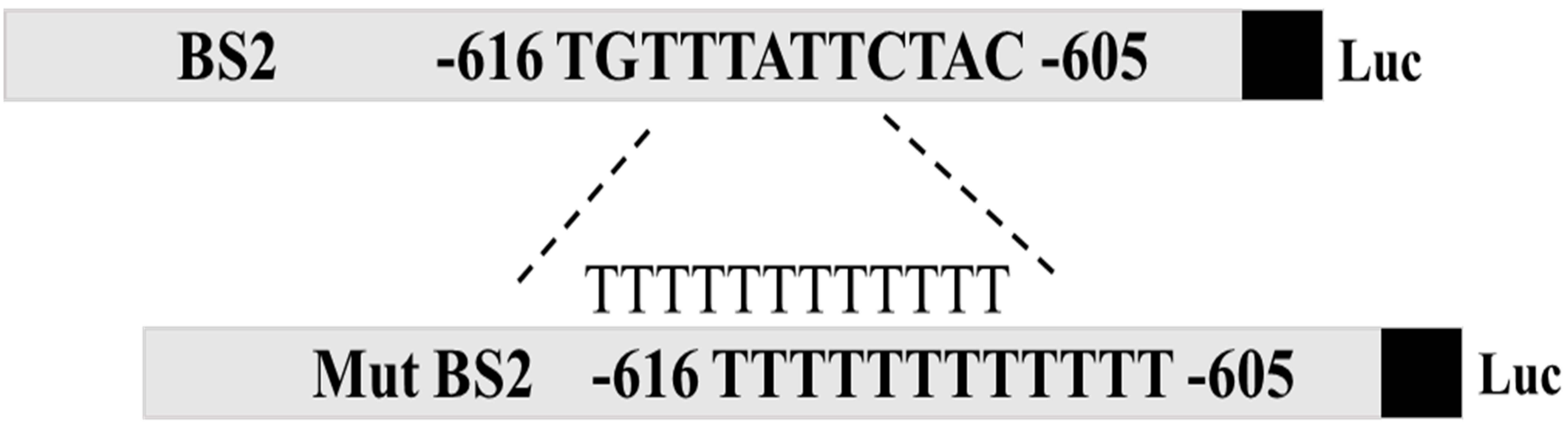

Supplemental Figure 3

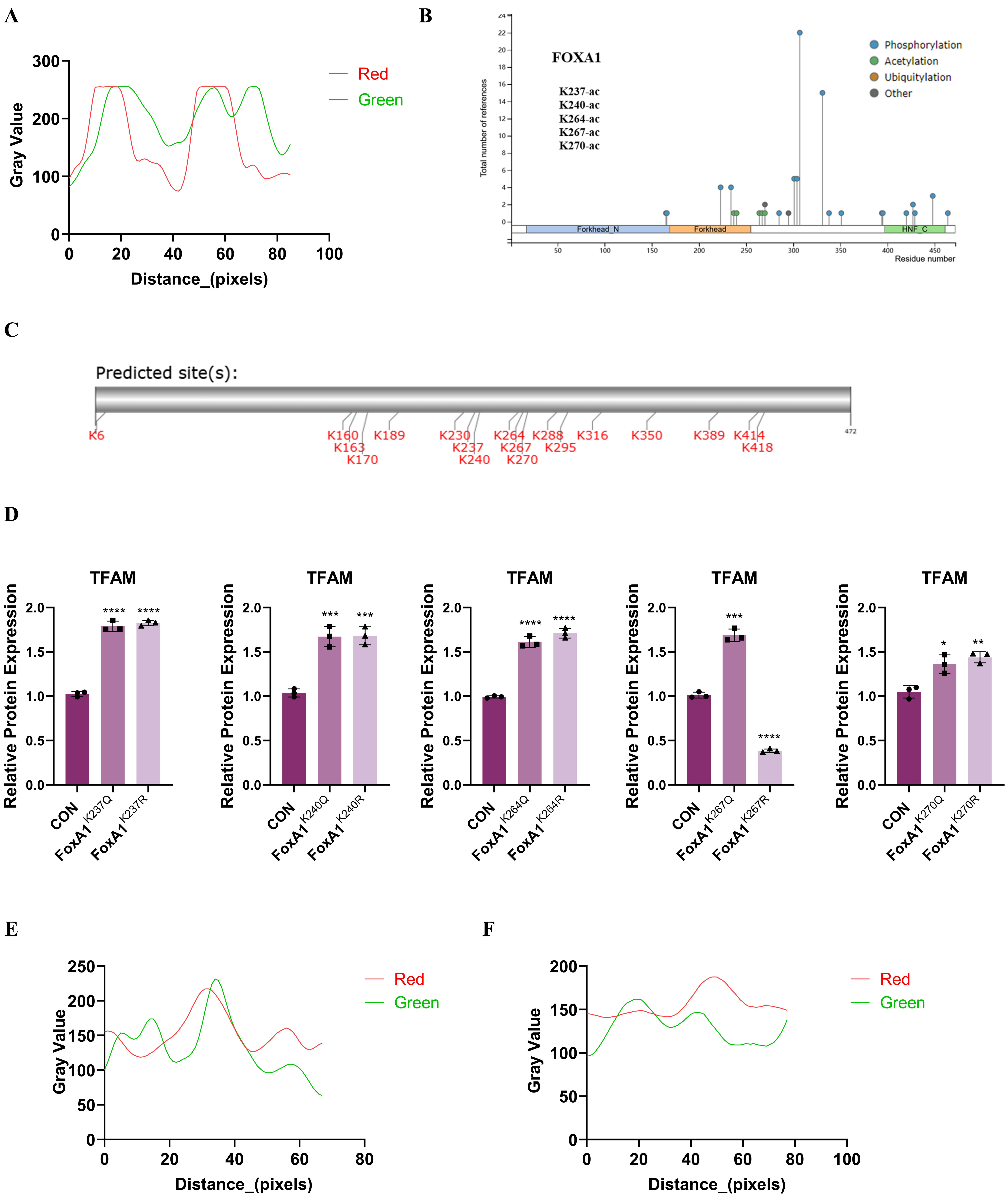

Supplemental Figure 4

A

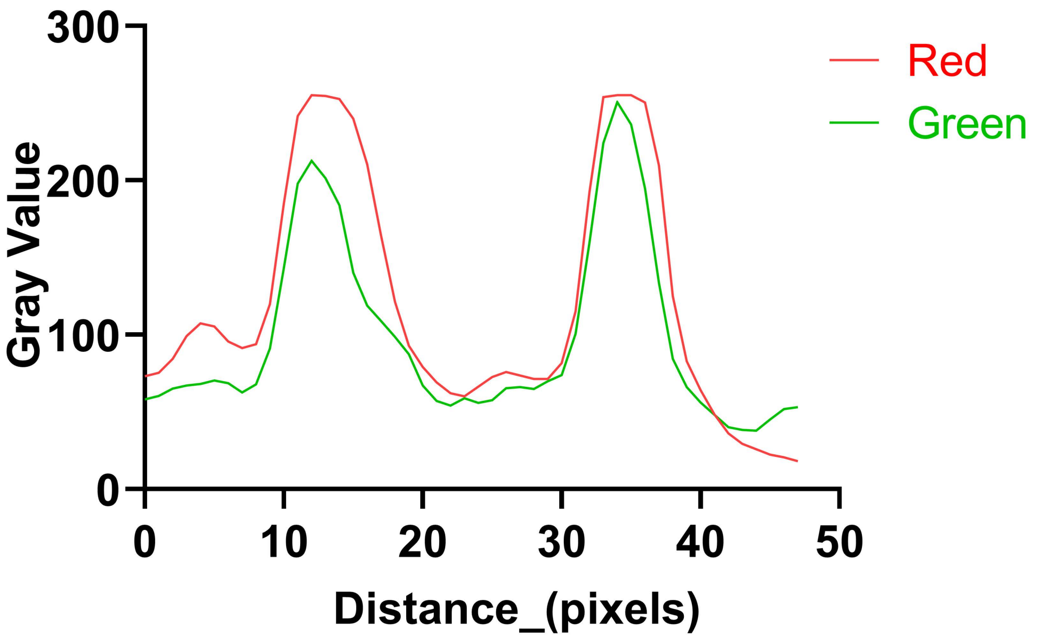

B

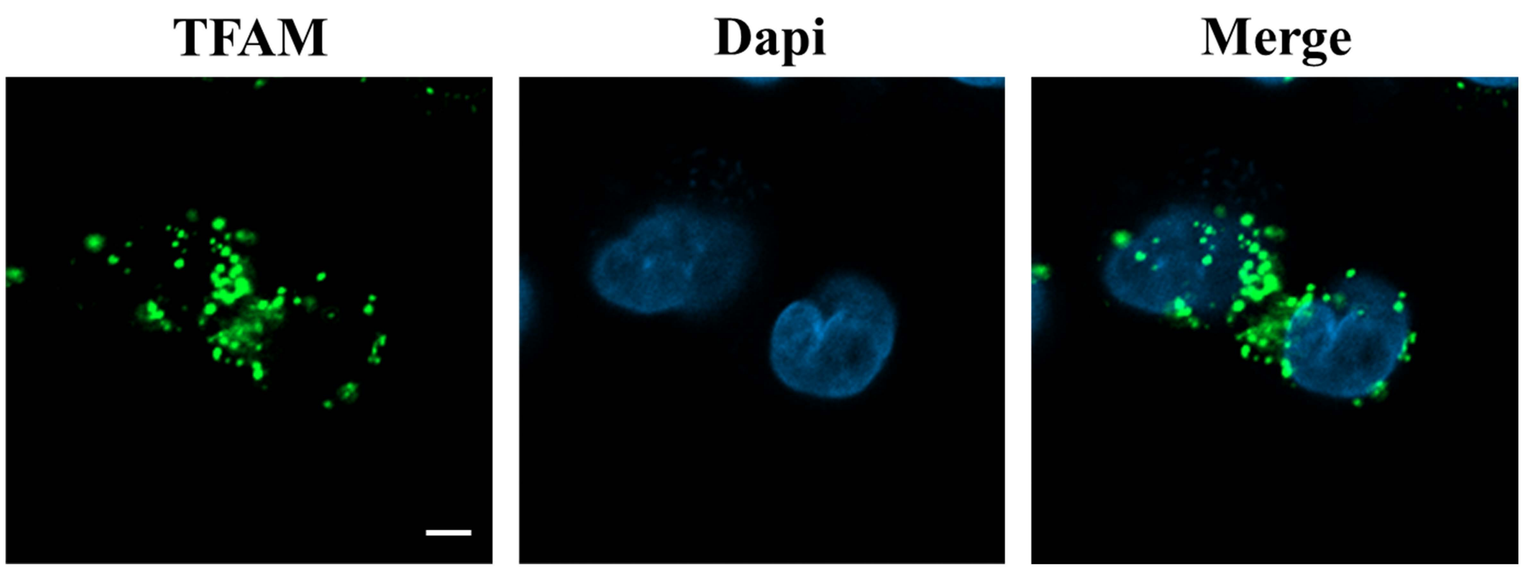

C

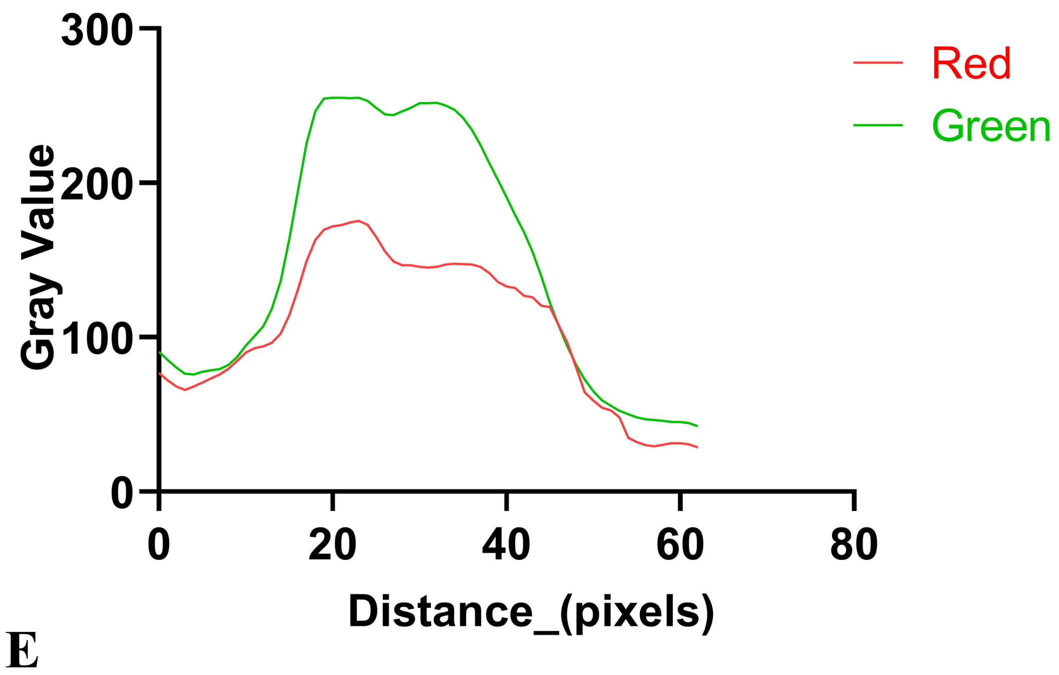

D

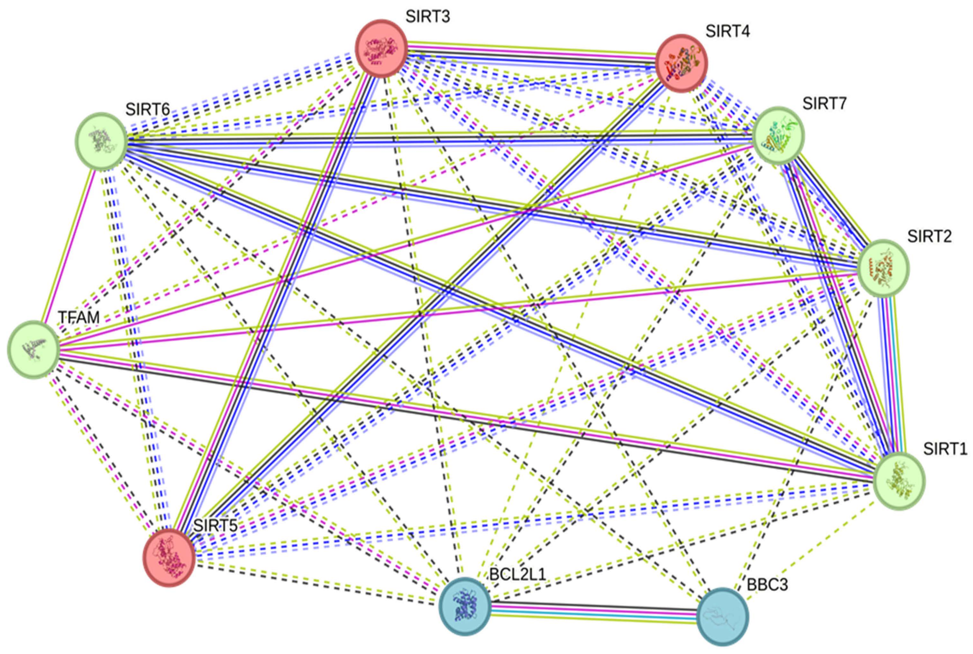

E

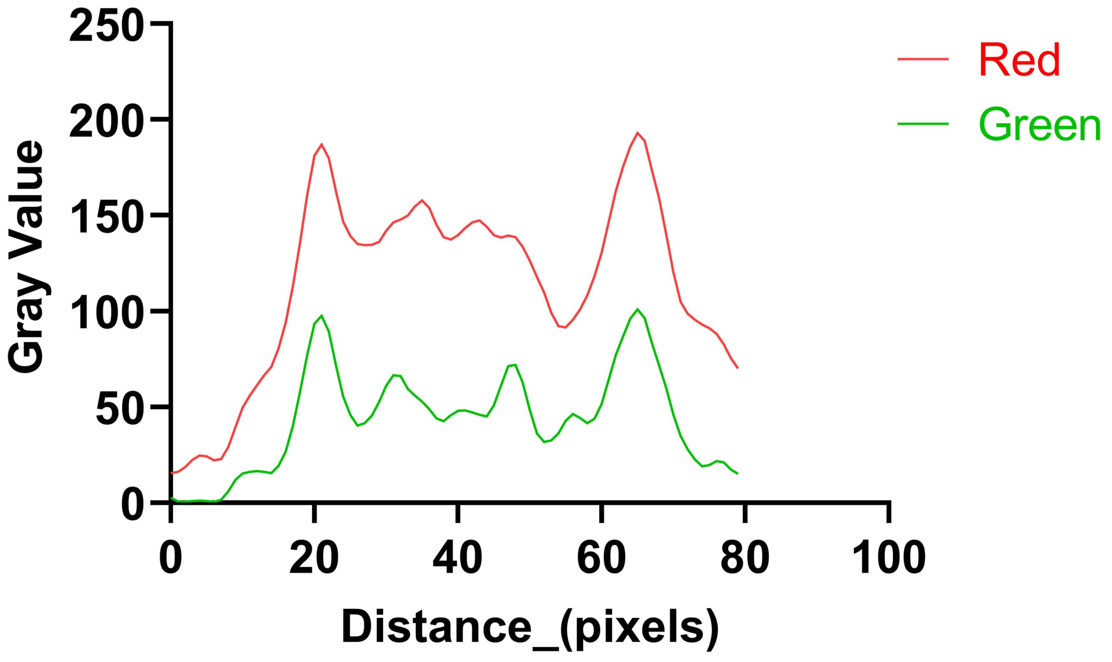

Supplemental Figure 5

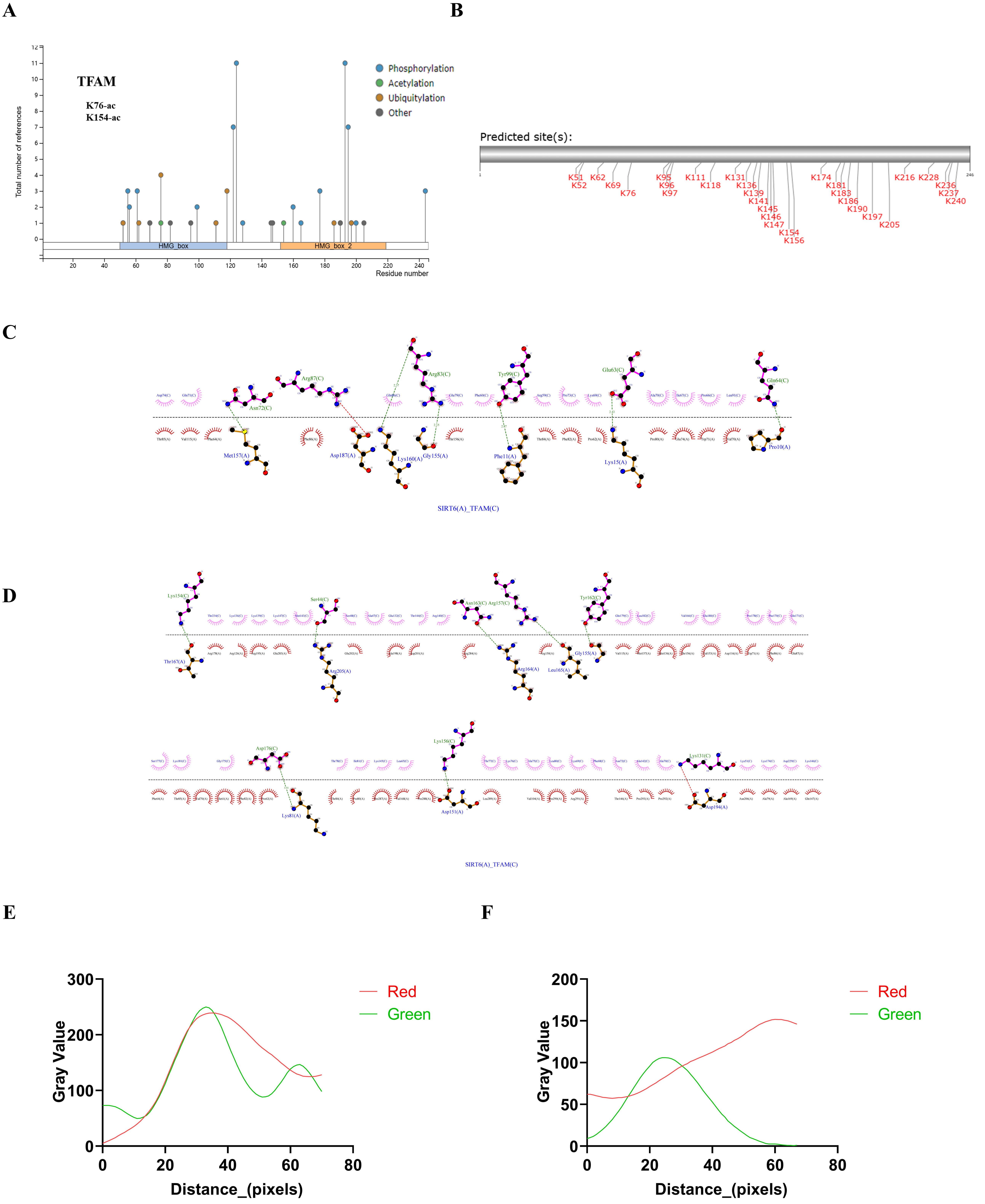

Supplemental Figure 6

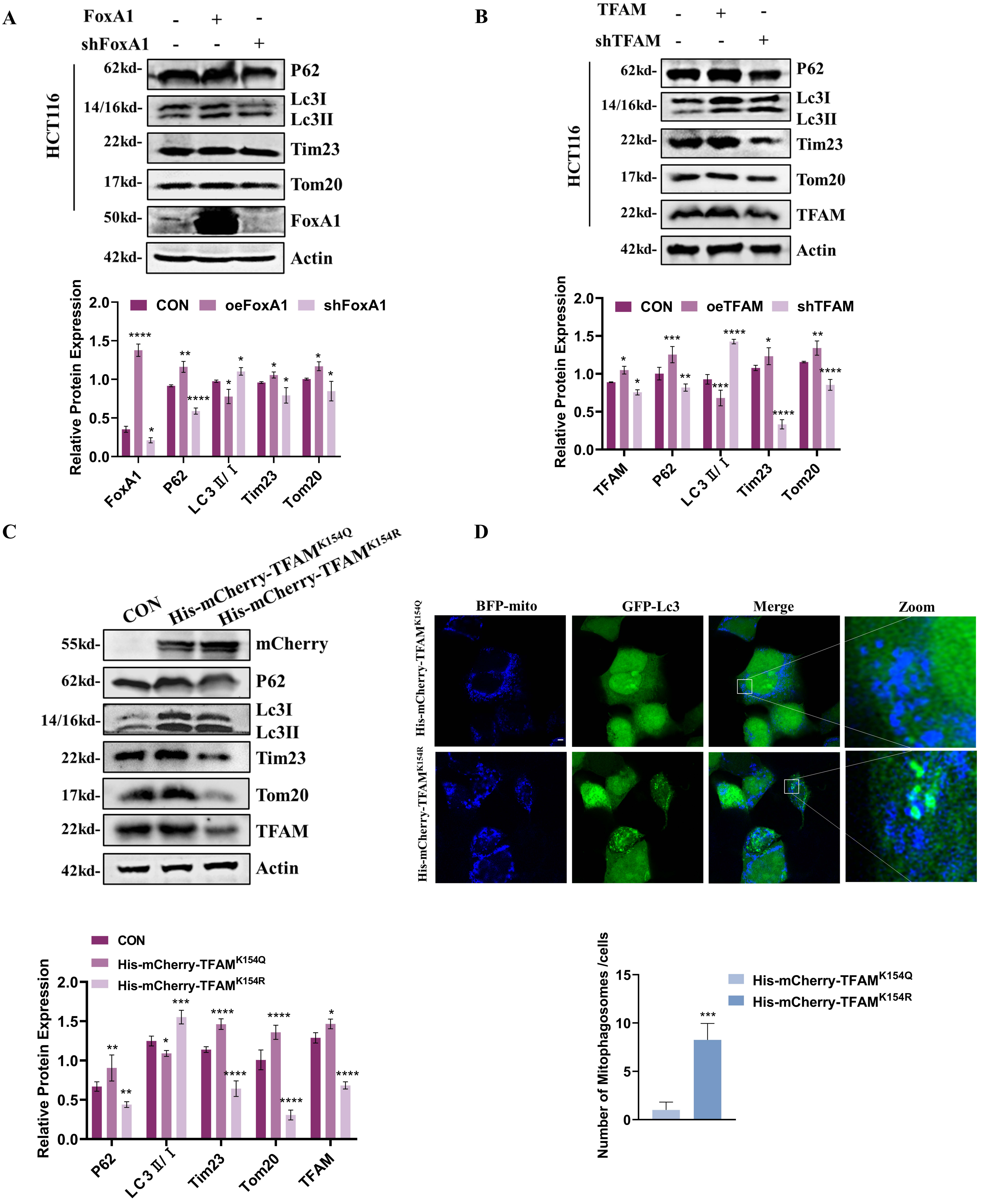

Supplemental Figure 7

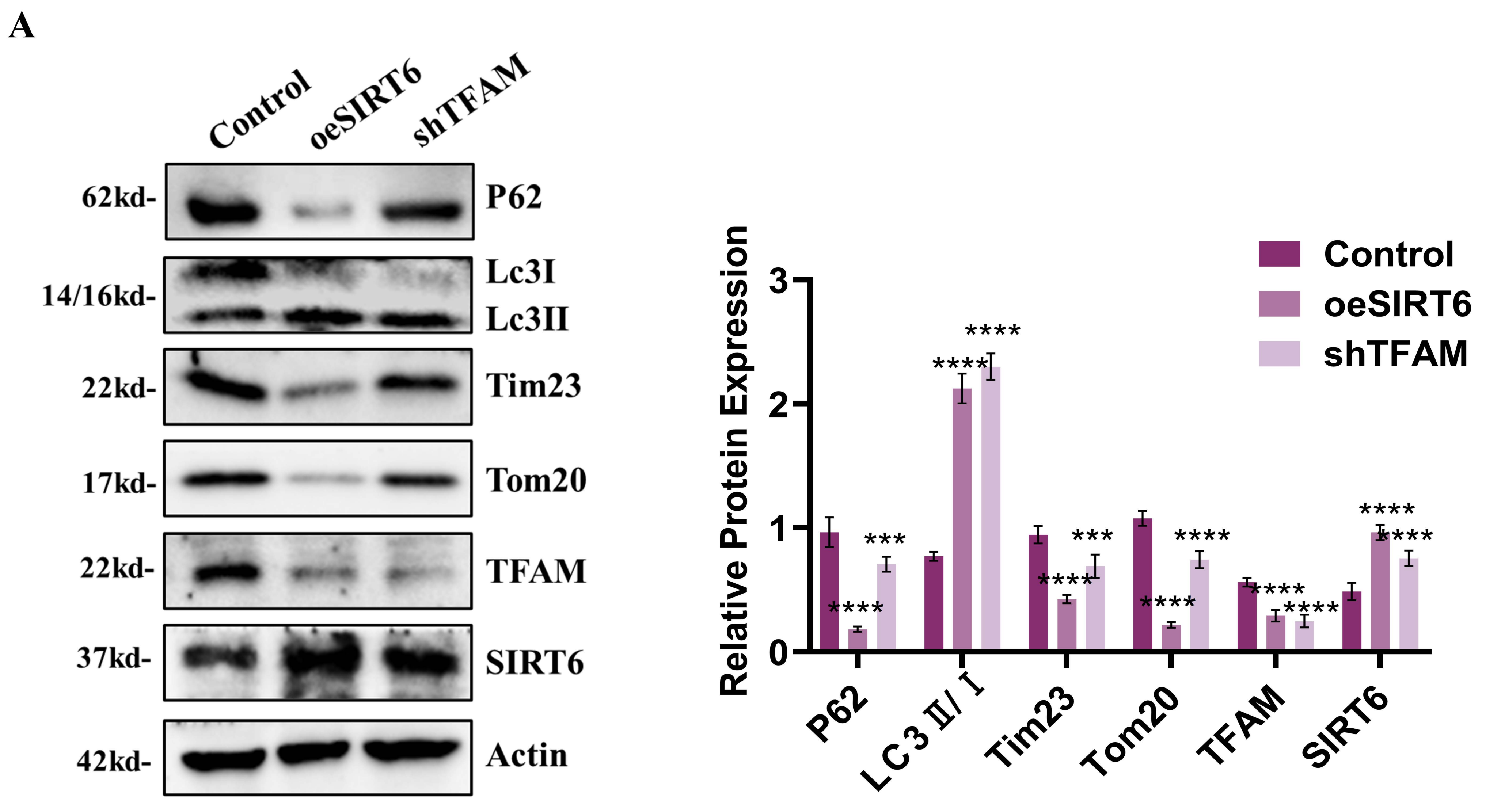

Supplementary Figure 1.

(A) Protein-protein interaction (PPI) prediction of SIRT6, FoxA1, and YY1 was performed using the *STRING* database. (B) Protein and mRNA levels of YY1 were detected in SIRT6-overexpressed or knockdown HCT116 cells. (C) RT-qPCR analysis of YY1 and SIRT6 mRNA levels. (D) PPI prediction of SIRT6, NRF1, and PGC-1α using STRING. (E) PGC-1α protein levels were measured. Error bars represent mean ± SEM. NS, not significant; statistical significance: *$P < 0.05$, **$P < 0.01$, ***$P < 0.001$ vs control group. Data are representative of three independent experiments.

Supplementary Figure 2.

(A) Potential binding sites of FoxA1 on the TFAM promoter were identified. (B) Mutation analysis of BS2 binding site.

Supplementary Figure 3.

(A) Quantitative co-localization analysis in Figure 4B using Plot Profile in ImageJ software. (B-C) Prediction of potential acetylation sites on FoxA1 by two online prediction tools. (D) Quantification of TFAM protein levels in Figure 4H. (E-F) Quantitative co-localization analysis in Figure 4L using Plot Profile in ImageJ software.

Supplementary Figure 4.

(A, C) Quantitative co-localization analysis in Figures 5A/C using Plot Profile in ImageJ software. (B) PPI prediction of SIRT family and TFAM from *STRING*. (D) Immunofluorescence staining of TFAM, followed by confocal microscopy. Scale bar = 10μm. (E) Quantitative co-localization analysis in Figure 5D using Plot Profile in ImageJ software.

Supplementary Figure 5.

(A-B) Prediction of potential acetylation sites on TFAM using two online prediction tools. (C-D) 2D molecular docking model of SIRT6 binding to TFAM. (E-F) Quantitative co-localization analysis from Figure 6K, using Plot Profile in ImageJ.

Supplementary Figure 6.

(A) Western blot analysis of mitophagy markers in FoxA1-overexpressed or -knocked down HCT116 cells. (B) Western blot analysis of mitophagy markers in TFAM-overexpressed or -knocked down HCT116 cells. (C) Western blot analysis of mitophagy

markers in 293T cells transfected with $TFAM^{K154Q}$ and $TFAM^{K154R}$. (D) Confocal microscopy analysis of the number of cyan merged puncta after co-transfection of BFP-mito, GFP-LC3, and $TFAM^{K154Q}$/$TFAM^{K154R}$ in HCT116 cells. Representative images and quantitative analysis of the number of cyan dots are shown. Scale bar = 10μm. Error bars represent mean ± SEM. NS, not significant; statistical significance: *P < 0.05, **P < 0.01, ***P < 0.001 vs. control group. Data are representative of three independent experiments.

Supplementary Figure 7.

(A) Western blot analysis of mitophagy marker proteins in tumor tissues from xenograft mouse models. Error bars represent mean ± SEM. NS, not significant; statistical significance: *P < 0.05, **P < 0.01, ***P < 0.001 vs. control group. Data are representative of three independent experiments.

Supplementary Table 1

	A	B	C	D	E	F	G	H	I	J
1	Only SIRT6-Cc	Only MitoCar	Only MitoPro	SIRT6-Cor-Ge	SIRT6-Cor-Ge	MitoCarta AN	SIRT6-Cor-Gene AND MitoCarta AND MitoProteome			
2	PNRC1	ATP5F1A	1-Mar	GTPBP6	EEFSEC	SDHB	AASS			
3	SERINC1	ATP5F1E	2-Mar	MICOS13	DNAJC5	COQ7	ABCB8			
4	RO60	ATP5F1B	5-Mar	METTL4	PTPN11	SDHA	ABHD11			
5	S100A6	SDHAF4	4-Sep	C2orf69	GMPPB	COQ5	ACAD9			
6	EHD1	ATP5PO	ABCA12	ACAA1	BCAT1	PDHA1	ACADS			
7	ADGRL2	ATP5ME	ABCA8	GUK1	CLN3	COX5A	ACADVL			
8	GCA	ATP5PF	ABCF2	VPS13D	TMEM160	ISCA2	ACO2			
9	RNASEH2A	GATD3A	ABCG1	FDX2	NELFE	PMPCB	ACOT7			
10	FAM171B	MICOS10	ABCG2	TIMM29	CTSA	UQCRFS1	ADCK1			
11	FOXN3	ATP5F1C	ABHD6	RAB5IF	SPNS1	PDHB	ADCK2			
12	FBL	COQ8A	ABL1	DMAC2	KRAS	UQCRC2	ADCK5			
13	SENP1	ATP5PB	ACN9	ETFRF1	PPP1CC	SDHD	AIFM2			
14	FAM89B	MT-ATP6	ACO1	C8orf82	ERCC6L2	MRPS35	AIFM3			
15	HBS1L	MT-CO2	ACOX1	METTL15	UBA1	MRPL53	AKAP10			
16	FZR1	FAHD2A	ACOX3	MIGA1	ATG12	PDK4	ALKBH7			
17	STUB1	MTARC2	ACSBG2	C16orf91	LRP5	MRPS27	APOOL			
18	IFNAR1	SQOR	ACSL5	RCC1L	MAPK3	CS	ARL2			
19	RNASE2	ATP5PD	ACSM6	RPUSD3	SRC	GRPEL1	ATAD1			
20	ESPN	ATP5MG	ACYP2	MARCHF5	ACBD3	DLAT	ATAD3A			
21	PLEKHG5	MOCS1	ADAP2	GRHPR	MAN2A1	LRPPRC	ATAD3B			
22	TANK	MMUT	ADCK3	AFG1L	PSMB3	DLST	AURKAIP1			
23	TP53I13	ATP5MC3	ADCK4	NDUFAF8	CYP1B1	PDHX	BAD			
24	ATXN7L3	NIPSNAP2	ADH5	METTL8	ATG9A	MPC2	BAX			
25	FPGT	RIDA	ADO	RECQL4	ATXN3	NDUFS1	BBC3			
26	STX7	MRPL58	ADPRHL2	NAXE	GSK3B	MRPL46	BCAT2			
27	GSDMD	MRM3	AGAP2	ATP5MC1	DIAPH2	SLC25A3	BCKDK			
28	NIBAN2	FMC1	AGR2	ATP5MC2	PFDN2	MRPS23	BCS1L			
29	GPR63	SDHAF3	AKAP8	MYG1	UACA	FH	BDH1			
30	DENND1B	PRELID3B	AKT1	ANTKMT	ZNF205	SUCLA2	BLOC1S1			
31	ARTN	ECHDC1	ALB	FABP1	ELK3	COQ3	BNIP3L			
32	PIGA	MTRES1	ALDOC	UQCC3	PPP2R3C	IARS2	C6orf136			
33	ZMYND19	KYAT3	ALKBH3	NSUN3	GSTP1	MRPS15	CA5B			
34	SLAIN2	MAIP1	ALOX12	PNPLA8	USP48	IDH3A	CASP8			
35	ARHGEF12	HAO2	AMBRA1	ANGEL2	MAP1S	COX11	CBR4			
36	PKN2	ATP5MPL	ANXA1	TRMT1	ZMIZ2	ETFDH	CHCHD10			
37	PRPF6	COX19	ANXA10	ATP5F1D	ZDHHC8	BCKDHA	CHCHD2			
38	NKIRAS1	HDHD5	AP2M1	ATP5IF1	INF2	UQCRH	CHCHD5			
39	CHTF18	COA8	APOA1BP	PREPL	STK11	LETMD1	CHCHD6			
40	GSS	ATP23	APOPT1	SPHK2	TTC3	COX15	CISD3			
41	SUPT5H	ATP5MF	ARGLU1	SELENOO	HIVEP1	AFG3L2	CLPP			
42	CWF19L2	NGRN	ARL2BP	TMEM205	ATG2A	HADHA	CLPX			
43	TMEM161A	MT-CO1	ARL6IP5	MIGA2	AIP	ETFA	COA1			
44	RC3H2	MTFR1L	ARMS2	ARF5	RPS6KB1	CPT2	COA3			
45	GYS1	SDR39U1	ARRB2	SLC30A9	CTU2	BCKDHB	COASY			
46	ITPRIPL2	PRELID3A	AS3MT	MCRIP2	IREB2	IDH3B	COMT			
47	GNB2	TOMM70	ASAH2	PRORP	ATG4C	LARS2	COMTD1			
48	GINM1	MT-ND2	ASB9	SMIM8	AGPS	LETM1	COQ4			
49	VCPKMT	MT-ND4	ASS1	PRDX2	NAIF1	AUH	COQ9			
50	B3GAT3	MT-ND5	ATCAY	DCXR	CAV2	SUCLG1	COX14			
51	SON	DELE1	ATG3	PUSL1	CTU1	NDUFV2	COX4I1			
52	PDE4D	STOM	ATG4A		MEF2A	COQ6	COX5B			
53	ZBTB25	METTL5	ATG5		PPP3CA	NDUFB8	COX6A1			
54	THAP9	DGLUCY	ATG7		PYCARD	DLD	COX6B1			
55	TMPO	MT-CYB	ATIC		TIMM23B	AIFM1	COX8A			
56	FLOT1	AKR1B10	ATP2A1		AGTPBP1	ECHDC3	CRY1			
57	GMEB2	PXMP4	ATP5A1		FAM32A	MRRF	CYB5R3			
58	UBALD1	CHPT1	ATP5B		ATF2	ACADM	CYC1			
59	RAD17	MT-ATP8	ATP5C1		CASP8AP2	IMMT	D2HGDH			
60	ANAPC2	HTATIP2	ATP5D		DDAH2	TIMM9	DBT			
61	SP1	AKR7A2	ATP5E		GPRC5C	SLC25A4	DHRS1			
62	TUBA4A	MT-CO3	ATP5EP2		VRK2	SAMM50	DHX30			
63	WDR83OS	DHRS7B	ATP5F1		PPP2R1A	SUPV3L1	DNA2			
64	REL	MTHFS	ATP5G1		DHX29	FECH	DNAJA3			
65	YIPF3	PNPO	ATP5G2		CLIC4	MTIF2	DNAJC4			
66	TP53BP1	PRDX6	ATP5G3		ATG4D	HIBCH	DNM1L			
67	PRICKLE3	GARS1	ATP5H		NUDT1	HSPA9	ECH1			
68	ZCCHC7	LYRM9	ATP5I		ATG4B	SURF1	ECHS1			

	A	B	C	D	E	F	G	H	I	J
69	PPP2R3A	NAXD	ATP5J		SFN	PRDX3	ECI1			
70	NFXL1	MT-ND3	ATP5J2		MAP3K1	GHITM	ECSIT			
71	ZDHHC12	ACCS	ATP5L		CSDE1	GUF1	ENDOG			
72	ZNF521	LYPLAL1	ATP5L2		MAP1LC3A	LYRM4	ERAL1			
73	TRAF2	DNAJC28	ATP5O		ARAF	MRPL16	ETFB			
74	KRIT1	NRDC	ATP5S		SH3GLB1	MRPL40	ETHE1			
75	KITLG	FAM210B	ATP5SL		DDX3X	SDHC	EXOG			
76	WDFY3	AHCYL1	ATP6		PEMT	NDUFB5	FASN			
77	PDZD3	NIPSNAP3A	ATP6V1E1		ABCE1	COQ10A	FASTK			
78	UBA52	MTARC1	ATP7B		TCIRG1	COX6C	FASTKD2			
79	SLC39A4	CBR3	ATP8		TRAF6	NDUFB9	FBXL4			
80	ZC3H11A	KARS1	ATPIF1		MPG	MTCH2	FIS1			
81	LARP1B	TMEM177	AVP		MAP2K2	NDUFA6	FKBP8			
82	LNPK	TOP3A	AZIN2		PALLD	SLC25A20	FLAD1			
83	H4C3	LDHAL6B	BAG1		SLC30A6	MRPL1	FPGS			
84	TERF1	MCUB	BAG5		CHPF	COX17	FTH1			
85	TULP4	TWNK	BBOX1		PLN	VDAC1	GADD45GIP1			
86	DGCR6L	PYCR2	BDH2		MEF2C	HADH	GCDH			
87	GRINA	TSTD1	BDNF		EMC8	ACADL	GFER			
88	FKBP7	OSBPL1A	BECN1		DHX57	ACAT1	GFM1			
89	KIAA1143	COQ8B	BFSP1		RNF144B	MALSU1	GLS			
90	CFL2	TSTD3	BHLHA15		TUSC2	NDUFS4	GLYCTK			
91	RBAK	EPHX2	BLID		RAB1B	C1QBP	GPAM			
92	CPTP	NUDT5	BLOC1S2		LMNA	PITRM1	GPD2			
93	ZBTB17	BOLA3	BMF		NR3C1	UQCC1	GPX1			
94	GNB1L	PLPBP	BNIP1		TMTC1	MRM1	GPX4			
95	MIA3	HINT1	BOP		MYH14	MECR	GRPEL2			
96	NRAS	MRM2	BRD8		ATP7A	MRPL44	GTPBP10			
97	HS2ST1	MGST3	BRI3BP		ATP6V1A	HSDL2	GTPBP3			
98	SYNCRIP	SMIM20	BRINP3		POP7	MRPS14	HAGH			
99	XAB2	MT-ND1	BTD		DNAJC27	ATPAF2	HDHD3			
100	RAB17	ACOT11	BZRAP1		CYBB	NDUFA9	HIGD2A			
101	YIPF6	NIPSNAP3B	C10orf10		BCAP31	COA6	HINT3			
102	PUM1	ACOD1	C12orf10		AKT3	ISCU	HSD17B10			
103	RPGR	DMAC2L	C14orf119		ACSL3	RTN4IP1	HTRA2			
104	MAP3K13	PRXL2A	C14orf159		RIPK3	OGDHL	IDE			
105	TRAPPC1	ATP5MD	C14orf2		ZFHX3	GFM2	IDH3G			
106	DYRK3	C15orf61	C15orf62		TBC1D15	NDUFAF4	ISCA1			
107	PHTF1	POLQ	C17orf89		CYBA	SLC25A35	ISOC2			
108	TBC1D17	CRYZ	C19orf12		ADAM12	TIMM8B	LACTB			
109	SAMD12	C15orf48	C19orf70		NT5C	IBA57	LONP1			
110	PLEKHH3	MT-ND4L	C21orf33		GCLM	PHB2	LYRM2			
111	COLGALT1	NOCT	C2orf47		POR	DAP3	MACROD1			
112	ARRDC3	NT5DC2	C6orf203		GTPBP8	CMC2	MCAT			
113	SCLT1	TRUB2	C7orf55		RRM2B	MRPS17	MDH2			
114	GNS	C3orf33	C9orf89		TNFRSF1A	ADHFE1	MFN1			
115	PTPRC	HPDL	CA5BP1		CLTC	NFS1	MIEF2			
116	KIAA1586	CDK5RAP1	CABS1		VPS25	NDUFAF5	MPST			
117	THAP6	PAICS	CAMK2A		APEX2	MRPL11	MPV17L2			
118	MED6	AGPAT4	CAPN10		MARK2	MRPL13	MRPL10			
119	CENPC	FAM185A	CAPRIN2		RILP	OXCT1	MRPL12			
120	CCDC61	SPATA20	CARKD		RAB8B	PDK2	MRPL14			
121	ARMC8	CCDC127	CASP1		PTEN	ALAS1	MRPL17			
122	ZNF184	SEPTIN4	CASP2		MAPK1	MRPL33	MRPL19			
123	NFATC3	DMAC1	CASP4		ATG2B	MTO1	MRPL2			
124	NAGLU	ATPSCKMT	CASP7		ACOT8	LIAS	MRPL23			
125	RBM12B	CKMT1B	CASQ1		TNRC18	NDUFB6	MRPL28			
126	SNRK	ATP5MF-PTCI	CCAR2		GSK3A	MTX2	MRPL34			
127	ZNF283	EXD2	CCBL2		PUF60	SUCLG2	MRPL36			
128	RB1	NBR1	CCDC109B		MLLT11	FDX1	MRPL38			
129	CCNY	FKBP10	CCDC136		PRKCD	MRPS28	MRPL4			
130	HECTD3	SETD9	CCK		CEBPA	VDAC2	MRPL41			
131	NAA35	CSKMT	CCT7		CLIC1	SLC25A42	MRPL42			
132	TXNDC16	RPIA	CD24		ACSL4	TOMM7	MRPL43			
133	MED15	MT-ND6	CD3EAP		TRAK2	GATB	MRPL54			
134	BLOC1S6	PRKN	CDK1		SHARPIN	CMC1	MRPL55			
135	C8orf37	STYXL1	CDK7		GRN	TSFM	MRPL57			
136	PRRG2	NSUN2	CDKN2A		PACS2	FXN	MRPS12			

	A	B	C	D	E	F	G	H	I	J
137	PSME4	ADCY10	CDS2		RRP15	NFU1	MRPS18A			
138	TAB1	ETFBKMT	CECR5		AFF4	YARS2	MRPS2			
139	SCAF11	RTL10	CERK		KIF1B	MRPL21	MRPS24			
140	CHD1	SPIRE1	CFAP45		ARSB	ALDH4A1	MRPS26			
141	DRD4	ARMCX2	CHAT		P4HA1	TOMM40L	MRPS34			
142	VAPA	ARMCX1	CHCHD2P9		ARMC1	NDUFAF1	MTCH1			
143	NOTCH2	POLB	CHMP2B		HIF1A	ACADSB	MTG2			
144	CDK10	SNAP29	CIAPIN1		SIRT1	MRPS9	MTHFD2L			
145	PCBP1	ARMCX6	CIDEA		TATDN3	MRPL15	MTPAP			
146	ZNF574	PIGBOS1	CISD2		VAMP8	MRPL24	MTRF1L			
147	MTF2	HTD2	CLN8		BRAF	HSPD1	MTX1			
148	STXBP5	RP11_469A15	CLU		RAB11B	NDUFS5	MTX3			
149	SPINT2		CNP		FIBP	AK3	NADK2			
150	GRK6		COL4A3BP		CKB	CYCS	NDUFA11			
151	KLHL23		COX1		MAP1B	MIPEP	NDUFA13			
152	ZNF576		COX2		SPATA5	LYRM7	NDUFA2			
153	NSMCE1		COX3		SIVA1	CRAT	NDUFA3			
154	STAG1		COX7A2P2		FYN	PCCB	NDUFA5			
155	RWDD4		CRYAB		SACS	MRPS7	NDUFA8			
156	CERT1		CRYM		CLUH	MRPL3	NDUFAF3			
157	DDHD1		CTPS2		PPP3R1	PRODH	NDUFB10			
158	DOHH		CTSB		TMEM102	PCCA	NDUFB11			
159	MPP6		CXADR		E2F1	MCCC1	NDUFB7			
160	OGFRL1		CXorf23		BSG	CLPB	NDUFS2			
161	SNAP23		CYB5A		CANX	MRPL49	NDUFS3			
162	TCF4		CYB5R1		HEATR1	COX7A2	NDUFS6			
163	NEMP2		CYB5R2		MAPK8	TMEM126A	NDUFS7			
164	REEP4		CYP17A1		RAI14	ECHDC2	NDUFS8			
165	GDAP2		CYP1A1		NAPG	HCCS	NDUFV1			
166	NAA25		CYP2D6			HIBADH	NIT1			
167	NUFIP2		CYP2E1			FARS2	NME3			
168	THOP1		CYTB			ABCB7	NME4			
169	KLHL17		DACT2			NDUFA7	NTHL1			
170	SRRT		DAO			TIMM17A	NUDT8			
171	LCLAT1		DAOA			ALDH9A1	OGDH			
172	SPPL2B		DCAF5			MRPS18C	OMA1			
173	INPP4A		DCPS			MARS2	OPA1			
174	TMEM123		DDAH1			ALDH6A1	OSGEPL1			
175	HEATR5A		DDIT4			FDXR	OXLD1			
176	TBL3		DDX23			GATC	OXR1			
177	DZIP1		DEGS1			ACAD8	PAM16			
178	LRFN4		DGAT2			ALDH2	PC			
179	BZW1		DHFRL1			PPIF	PCK2			
180	DDX54		DHX32			TIMM22	PDF			
181	ARF1		DISC1			IVD	PDK1			
182	SH3BGRL3		DNAJA1			L2HGDH	PHB			
183	RARA		DNM3			MRPL20	PICK1			
184	CISH		DPYSL2			SLC25A5	PMPCA			
185	DALRD3		DRG2			SLC25A12	PNKD			
186	DSE		DSP			MRPS21	POLDIP2			
187	RECQL		DUSP21			TOMM22	POLRMT			
188	TUBGCP2		DUSP26			ACAA2	PPM1K			
189	LEPR		DYNLL1			MRPL30	PPTC7			
190	DDR2		DYNLL2			NDUFB2	PRDX5			
191	PSMC3		ELK1			COX7C	PRELID1			
192	SWT1		ELN			CHCHD3	PRIMPOL			
193	TMEM238		EMC2			COQ10B	PTCD1			
194	R3HDM4		ENOSF1			ACSF3	PTCD2			
195	TASOR2		EPAS1			SLC25A13	PTGES2			
196	ZNF562		EPHA4			PDK3	PUS1			
197	CCNJ		ERBB4			ME3	PYCR1			
198	YAP1		ERN1			MRPL22	QTRT1			
199	TRPM4		ESR2			IDH2	RAB24			
200	FAM3A		ETNPPL			MRPL47	RHOT1			
201	PSMB10		EYA2			PPA2	RHOT2			
202	VPS13A		FADS1			MRPL9	RMDN1			
203	CD109		FAM110B			WARS2	ROMO1			
204	ARL5A		FAM65B			SLC25A19	SDHAF1			

	A	B	C	D	E	F	G	H	I	J
205	WIZ		FAM72A			SMDT1	SDHAF2			
206	CD320		FANCG			COX7A1	SDSL			
207	ECT2		FBXO7			MTG1	SLC25A1			
208	MEMO1		FDX1L			NDUFA12	SLC25A10			
209	PTPN2		FEN1			MRPS16	SLC25A11			
210	MGRN1		FEZ1			MTERF2	SLC25A22			
211	PNISR		FGR			NDUFA10	SLC25A24			
212	ZNHIT6		FITM2			NDUFC2	SLC25A28			
213	ERCC6		FKBP4			SLIRP	SLC25A30			
214	CHM		FLCN			TIMM21	SLC25A36			
215	JAG2		FLVCR1			DHTKD1	SLC25A39			
216	SIPA1		FNDC1			HSD17B8	SLC25A40			
217	PLP2		FOXO1			HINT2	SLC25A51			
218	KMT2E		FSIP2			MRPS5	SLC25A6			
219	DIPK2A		FTSJ2			SPRYD4	SOD2			
220	CARM1		G0S2			LIPT2	SPG7			
221	LLGL2		GABARAP			DECR1	SPR			
222	DESI2		GABARAPL1			SLC25A15	STOML2			
223	NAA50		GABARAPL2			NDUFV3	STX17			
224	PGLS		GABARAPL3			BPHL	SYNJ2BP			
225	ZNF672		GARS			MSRB2	TACO1			
226	TET2		GBAS			SLC25A25	TAZ			
227	UFL1		GBF1			ACAD10	TBRG4			
228	FAU		GCK			VWA8	TCAIM			
229	CLDN3		GCKR			CCDC90B	TFAM			
230	SDCCAG8		GCLC			DNAJC11	TIMM10			
231	DUSP8		GDF5OS			GLDC	TIMM13			
232	GET3		GGCT			MPC1L	TIMM17B			
233	ROGDI		GGNBP1			THEM4	TIMM44			
234	WDR47		GIMAP5			SSBP1	TIMM50			
235	DHFR2		GIMAP8			MRPL27	TMEM11			
236	ZNF436		GJA1			HSCB	TMEM65			
237	BABAM1		GK			MRPS10	TOMM40			
238	ERF		GK2			AK4	TRAP1			
239	CDON		GK3P			MRPL37	TRMT5			
240	NPRL2		GLRX			TXNRD2	TRNT1			
241	ARCN1		GLUL			ACSM5	TSPO			
242	DSTYK		GLYATL1			CISD1	TST			
243	GAN		GLYATL2			MRPS6	TUFM			
244	OSBPL3		GLYATL3			SCO1	TXN2			
245	MFSD4B		GM2A			GCAT	UQCR10			
246	SRRM1		GNB2L1			MTHFD1L	UQCR11			
247	MAF1		GNL3L			ECI2	UQCRC1			
248	CDC37		GNPAT			UQCRB	UQCRQ			
249	TTC33		GPD1			CCDC58	YME1L1			
250	ZNF445		GPER1			MCCC2				
251	OVOL1		GRAMD4			MCEE				
252	TJP3		GSDMC			OXNAD1				
253	RPL36		GZMB			DARS2				
254	ACTN4		H6PD			MRPL51				
255	PLEKHA8		HAAO			CLYBL				
256	MYCBP2		HAP1			MLYCD				
257	KCNQ1		HAX1			PYURF				
258	MCM7		HCFC1			MRPL32				
259	FGF7		HCLS1			SLC25A26				
260	AGPAT2		HDDC2			PRODH2				
261	ESRP2		HDGFRP2			NIT2				
262	NBAS		HEBP2			ME2				
263	MYO5A		HERC2			COX6A2				
264	UBE4A		HGF			TAMM41				
265	MRTFB		HK1			TIMM23				
266	FBXL3		HK2			RMND1				
267	TOR1AIP1		HK3			MFN2				
268	GDPD3		HLCS			DNLZ				
269	UBE2J1		HOXB9			CHCHD4				
270	PARVA		HRK			NDUFAB1				
271	CEP112		HRSP12			ACSM1				
272	IL17RC		HSD3B1			COA5				

	A	B	C	D	E	F	G	H	I	J
273	H2AC13		HSD3B2			COQ2				
274	FEM1B		HSH2D			PDSS2				
275	LSM10		HSP90AA1			EHHADH				
276	GXYLT1		HSP90AB1			ABCB10				
277	RAVER1		HSPA1A			CKMT2				
278	CDC5L		HSPA1L			MRPS25				
279	AHI1		HSPA2			COX16				
280	PPP6R3		HSPA4			FAM210A				
281	PLXDC2		HSPA5			ACOT13				
282	ZNF254		HSPB7			CPT1A				
283	UBXN6		HTT			DHRS4				
284	PLCB1		ICT1			PRELID2				
285	TMEM150A		IDH1			CARS2				
286	PRICKLE2		IER3			GLRX2				
287	GRWD1		IFI6			MRPS30				
288	LRCH3		IFIT2			SCO2				
289	WDR19		IFIT3			NARS2				
290	POGLUT3		IGF1			SUOX				
291	SDF4		ILF3			SARDH				
292	AZI2		IQCE			MPV17				
293	TMEM134		IRG1			ZADH2				
294	CYB561A3		ISL1			MRPL18				
295	PPP1R15B		JTB			ALDH1B1				
296	KRTAP5-1		JUN			CHCHD1				
297	FAM168A		KANK2			TIMM8A				
298	TAF1B		KARS			VDAC3				
299	PANK3		KCNJ11			MPC1				
300	SLC1A3		KCNJ8			SLC25A31				
301	EOLA1		KCTD14			SLC25A18				
302	ZNF770		KIAA0141			GSTK1				
303	CDC42BPG		KIAA0391			VARS2				
304	ZNF420		KIAA1279			HSPE1				
305	ZNF12		KIAA1683			TRIAP1				
306	CSNK1G1		KIF28P			PTCD3				
307	APPBP2		KLK6			COX10				
308	OSBPL8		KRT5			AKAP1				
309	SLC6A9		KYNU			AK2				
310	UBL4A		LACE1			TMEM70				
311	SUZ12		LDHA			NOA1				
312	HES4		LGALS3			MCUR1				
313	EDIL3		LIG1			TMEM14C				
314	DNAJC3		LIN28B			NDUFC1				
315	CADPS2		LIPF			MRPL35				
316	RBMS1		LOC102723830			PDSS1				
317	MCPH1		LPIN1			SLC25A16				
318	TYSND1		LRRC10			ACSM3				
319	MAST4		LRRC59			NDUFAF6				
320	LPGAT1		LRRC75A-AS1			UCP1				
321	FLYWCH1		LRRK1			SFXN1				
322	NFKBIB		LRRK2			GLRX5				
323	HMG20B		LYN			PTRH1				
324	DPH6		LYRM5			MRPS11				
325	BBOF1		MAATS1			XPNPEP3				
326	ARID4B		MAP1LC3B			PDP1				
327	ANKRD40		MAP1LC3B2			SLC25A21				
328	C19orf73		MAP1LC3C			SLC25A29				
329	DLG1		MAP2K1			SLC25A45				
330	HMBOX1		MAPK10			NDUFB3				
331	OSGIN1		MAPK12			REXO2				
332	NUP50		MAPK14			GATM				
333	NOC2L		MAPK8IP1			TIMM10B				
334	KLHL42		MAPK9			SARS2				
335	VPS4B		MAPT			CHDH				
336	FAM133B		MARS			EARS2				
337	TMEM181		MAT2B			MTFMT				
338	SRSF10		MDH1			HARS2				
339	SLFN5		ME1			NDUFA4				
340	RBM42		MECP2			NAGS				

	A	B	C	D	E	F	G	H	I	J
341	UBXN2B		MED7			HSD17B4				
342	SORBS3		METTL12			NDUFAF7				
343	ARHGEF6		METTL20			OAT				
344	GNA13		MGEA5			MRPL50				
345	THAP4		MINOS1			COX7B				
346	DMXL2		MINOS1-NBL1			RARS2				
347	CYBRD1		MLXIP			LACTB2				
348	BBS7		MMACHC			CHCHD7				
349	SLC22A18AS		MMP2			SHMT2				
350	LRP6		MOAP1			MTHFD2				
351	RYK		MOBP			CPS1				
352	IRAK1BP1		MPDU1			RDH13				
353	MSL2		MPO			TRMT2B				
354	LEMD3		MPP7			GSR				
355	THAP1		MSTO1			TEFM				
356	MOSPD2		MTCP1			ACSF2				
357	ZNF181		MTHFD1			TRMU				
358	RMI1		MTM1			MRPS22				
359	C5orf24		MTOR			LAP3				
360	TSKU		MTUS1			GLUD1				
361	PICALM		MUT			HMGCL				
362	JOSD2		MYCBP			FHIT				
363	CLIC2		MYH10			MRPS33				
364	ATAD2B		MYL10			NUBPL				
365	WDR1		MYO1C			MPV17L				
366	MIB1		MYOC			ALDH18A1				
367	G3BP2		MYOM2			OXSM				
368	MAPRE3		NARS			TARS2				
369	TMEM209		NCBP1			DNAJC15				
370	DPM2		ND1			HIGD1A				
371	CNPY3		ND2			SLC25A44				
372	BCAR1		ND3			OXA1L				
373	WAC		ND4			SLC25A46				
374	EFNA1		ND4L			RDH14				
375	TLR1		ND5			GSTZ1				
376	CCDC107		ND6			COX6B2				
377	TADA3		NDFIP2			GLOD4				
378	C1orf122		NDUFC2-KCTD14			CROT				
379	METTL25		NEFH			AMT				
380	GADD45G		NEFL			PDPR				
381	PSMA7		NFKB1			NLN				
382	TMEM120A		NGB			BOLA1				
383	XRCC1		NLRP5			ALAS2				
384	PPP1R14B		NME1-NME2			ALDH7A1				
385	NSDHL		NMT1			IMMP2L				
386	RABEP2		NOL3			COX4I2				
387	SLC16A5		NOL6			MRPL39				
388	BAZ1A		NOL7			AADAT				
389	DNAJB12		NOP14			FUNDC2				
390	CARD8		NOS1			LYPLA1				
391	PNPLA6		NOS3			PDHA2				
392	MYO1B		NOX4			ACOT2				
393	WASHC4		NPTX1			MMAB				
394	SMC2		NRD1			PPOX				
395	FZD3		NRF1			ABCD3				
396	COMMD2		NT5C3A			SIRT3				
397	RAB28		NVL			LDHD				
398	CLDN4		NXNL1			MRPL45				
399	PGP		OAS1			C12orf65				
400	MPP5		OAS2			IMMP1L				
401	USP47		OGT			POLG				
402	HYAL3		OLFM4			ATPAF1				
403	METRNL		OPRD1			ALDH1L2				
404	RASAL2		P2RY1			COX18				
405	TNKS		P2RY12			CPOX				
406	RSRC1		PACRG			NNT				
407	PSMD5		PAK7			DCAKD				
408	COMMD5		PARG			SFXN3				

	A	B	C	D	E	F	G	H	I	J
409	TMEM167A		PARK2			DIABLO				
410	PPP1R2		PECR			COX8C				
411	TMED9		PERP			PXMP2				
412	DTX3L		PEX5			AGXT2				
413	NUP155		PFDN4			GLS2				
414	EXOC5		PGR			APOO				
415	DPY19L4		PHYHIPL			ACAD11				
416	TMEM208		PHYKPL			C5orf63				
417	CFLAR		PI4K2A			HOGA1				
418	TNKS2		PI4KB			TTC19				
419	CDC14A		PID1			GCSH				
420	SYMPK		PIM2			SIRT5				
421	SLC12A9		PIN4			GPT2				
422	RXRA		PINX1			TOMM6				
423	PTDSS2		PKM			FAM162A				
424	C12orf4		PLA2G15			ABCB6				
425	THAP2		PLA2G2A			DMGDH				
426	DDX49		PLA2G4A			SLC25A14				
427	EXD3		PLA2G4B			FAHD1				
428	JUND		PLA2G6			MCU				
429	SLFN12		PLIN5			CYP27A1				
430	TBCCD1		PNPLA7			FASTKD1				
431	HNRNPR		POLD3			MRPS31				
432	RFX3		PON2			ALDH1L1				
433	RBM41		PPARGC1A			HADHB				
434	H2BC17		PPARGC1B			TRIT1				
435	SLC66A2		PPL			CRLS1				
436	TM9SF3		PPM1E			SLC25A32				
437	POLR2M		PPP1R15A			AARS2				
438	FER		PPP2CA			KMO				
439	RAB21		PPP2CB			SFXN5				
440	YDJC		PPP2R2B			ELAC2				
441	ZNF391		PPP3CC			CPT1B				
442	ARFRP1		PRDX1			UCP3				
443	TMEM245		PRKCA			SLC25A27				
444	PARP10		PRKCE			SLC25A23				
445	CLCC1		PRNP			HEMK1				
446	EFNA2		PROSC			LETM2				
447	RAB5C		PRR5L			MSRA				
448	HGS		PSAP			NMNAT3				
449	PDGFRA		PSEN1			SLC25A38				
450	TARBP2		PSEN2			ACLY				
451	CDC42		PSMD10			ABCD2				
452	FRY		PSTK			LIPT1				
453	RUFY2		PTRF			NUDT13				
454	TMX3		PTS			ACACA				
455	LPP		PVRL2			ABAT				
456	AP1G1		QARS			HMGCS2				
457	CARHSP1		QTRTD1			ALDH5A1				
458	NPRL3		RAB11A			CKMT1A				
459	PRSS3		RAB11FIP5			PISD				
460	KPNA5		RAB29			NDUFB4				
461	ZSCAN12		RAB32			FAM136A				
462	ZNF518A		RAB35			TOMM5				
463	CFAP410		RAB3A			NDUFA1				
464	RHBDF1		RAB3D			OPA3				
465	ZNF688		RAB40AL			PHYH				
466	ZNF791		RAD51			NDUFAF2				
467	VWA1		RAD51C			PARL				
468	SHLD2		RAF1			ALDH3A2				
469	EDRF1		RAI1			ACSL6				
470	CNOT6		RAP1GDS1			MRS2				
471	TIAM1		RARS			ABHD10				
472	SFT2D2		REEP1			NUDT2				
473	AGO4		RGS2			CYB5B				
474	NAPA		RHBDD1			GRSF1				
475	AQR		RIPK1			MRPS18B				
476	CLOCK		RMDN2			DNAJC30				

	A	B	C	D	E	F	G	H	I	J
477	FAM91A1		RNASEL			CAT				
478	PHTF2		RNF185			DGUOK				
479	CXXC1		RNF5			ACOT9				
480	CCDC66		RNMTL1			ACSS1				
481	ARHGAP24		RNR2			RFK				
482	ARMC5		RPL10A			STARD7				
483	ZDHHC21		RPL35A			TIMMDC1				
484	B3GALNT2		RPP14			SPATA19				
485	MACF1		RPS14			DUT				
486	DAXX		RPS3			MTRF1				
487	TMEM79		RPS6KA6			TMEM143				
488	MSR1		RSAD2			NT5M				
489	ADAMTS5		RUNX1T1			MICU2				
490	XRN1		SARM1			PCBD2				
491	HEXD		SARS			QRSL1				
492	ICE1		SCCPDH			NIPSNAP1				
493	GALNT1		SDS			ACSM2A				
494	MDM4		SECISBP2			SLC25A33				
495	GOLM2		SESN2			PARS2				
496	HDHD2		SGK1			MRPL52				
497	WDR13		SH3BP5			GOT2				
498	RLIM		SHC1			PTRH2				
499	MOSPD3		SHMT1			PGS1				
500	MUC20		SHOX2			SLC25A53				
501	C1QTNF3		SIAH3			MGST1				
502	CHAF1A		SLC16A1			MTFP1				
503	ELP2		SLC22A4			COA4				
504	MAP3K11		SLC25A17			NIF3L1				
505	BTBD2		SLC25A2			MAVS				
506	PPM1L		SLC27A2			METTL17				
507	CLTA		SLC27A3			ACACB				
508	TRIM15		SLC35B3			ARG2				
509	CFAP97		SLC35F6			CCDC51				
510	TWF2		SLC3A1			SUGCT				
511	SIGIRR		SLC44A1			TMEM126B				
512	ZNF433		SLC4A5			NSUN4				
513	TSC2		SLC8A3			ACSS3				
514	THOC6		SLC9A1			RBFA				
515	TSEN54		SLC9A6			METAP1D				
516	IRAK3		SLC9B2			FUNDC1				
517	ZNF224		SLIT3			ACSL1				
518	CEP192		SLMO1			COX20				
519	SAV1		SLMO2			HSDL1				
520	CDK6		SMCP			MTERF4				
521	GPR137B		SMIM4			NUDT19				
522	ZNF281		SNCA			HEBP1				
523	ELL		SNCB			PTPMT1				
524	EMC6		SNN			PGAM5				
525	BORCS6		SNPH			DTYMK				
526	CHD9		SORD			STAR				
527	TIGD5		SOX10			DNAJC19				
528	NAA10		SOX4			OTC				
529	ZFP1		SPARC			COX7A2L				
530	RPS28		SPATA18			MTERF3				
531	KIAA0232		SPG20			SFXN2				
532	PTOV1		SQRDL			PINK1				
533	CYB5R4		SRI			TXNRD1				
534	C3orf38		SRP19			THNSL1				
535	DAPK3		ST20			FDPS				
536	MGA		STAP1			IDI1				
537	MNAT1		STARD13			FTMT				
538	ZZZ3		STARD3			MTFR1				
539	PDS5B		STAT3			BCL2L13				
540	AP1S2		STXBP1			TRMT10C				
541	SPART		SYBU			TFB1M				
542	STK17B		SYNE2			MMADHC				
543	ATP6V0B		SYNJ2BP-COX16			LYRM1				
544	MYO7B		TAP1			PET100				

	A	B	C	D	E	F	G	H	I	J
545	CHORDC1		TAT			GDAP1				
546	NOP16		TCHP			MTIF3				
547	NUP153		TDH			UNG				
548	METTL26		TDRD7			GLYAT				
549	TEDC2		TERT			QDPR				
550	HIPK3		TFDP1			MRPS36				
551	UBE2M		TGM2			PDP2				
552	GNB4		TH			AGPAT5				
553	PRRX1		TMEM173			UCP2				
554	PLXNC1		TMEM8B			SLC25A37				
555	RHPN1		TNFSF10			SLC25A48				
556	LGALS4		TNNC1			SLC25A43				
557	CEP350		TOMM70A			SLC25A47				
558	AAAS		TP53			SLC25A41				
559	TNIP2		TP53AIP1			SLC25A34				
560	DNAJB9		TP73			MICU1				
561	WDR18		TPO			SOD1				
562	SIL1		TPP1			SERHL2				
563	RACK1		TRAF3			OCIAD2				
564	SURF6		TRAK1			RSAD1				
565	TKFC		TRIM31			PEX11B				
566	NABP1		TRIM39			TMEM186				
567	NF1		TSHZ3			PIF1				
568	TMOD3		TUSC3			ABCD1				
569	ZNF227		TXN			DBI				
570	ZNF25		TXNIP			PNPT1				
571	EML4		TYMP			TOP1MT				
572	RAB18		TYMS			RNASEH1				
573	TBL1XR1		UBB			ABCA9				
574	TMEM168		UBIAD1			SCP2				
575	VEZF1		UCHL1			NT5DC3				
576	TMEM214		URI1			CMC4				
577	ARMC7		UROS			LDHB				
578	NCOA2		USMG5			YBEY				
579	CD47		UXS1			MUL1				
580	SSBP4		UXT			TK2				
581	NCOA4		VAMP1			AGMAT				
582	ZFR		VARS			CMPK2				
583	CD302		VASN			BID				
584	ZMYM6		VAT1			MFF				
585	NAP1L1		VHL			NUDT9				
586	JMJD8		VHLL			AMACR				
587	SPPL2A		WDR45			NUDT6				
588	TMTC2		WDR45B			BAK1				
589	CLCN2		WIPI1			DHODH				
590	SH3BGRL		WIPI2			MAOB				
591	TP53INP1		WWOX			ACP6				
592	EIF4EBP3		XAF1			MRPL48				
593	APC		XRCC3			AGXT				
594	ACVR2B		YKT6			DDX28				
595	COPS6		YWHAB			MICU3				
596	TAF6L		YWHAE			BNIP3				
597	C21orf91		YWHAG			MUTYH				
598	NUP54		YWHAH			CYP11A1				
599	PAFAH1B2		YWHAQ			OCIAD1				
600	STRN4		YWHAZ			RPUSD4				
601	ANKRD54		ZBED8			NME6				
602	PIGQ		ZFYVE1			ACSM4				
603	RAB31		ZNF202			NLRX1				
604	DDHD2					FOXRED1				
605	GTF2H3					TFB2M				
606	UBXN4					MSRB3				
607	RABEP1					SFXN4				
608	CGGBP1					SLC25A52				
609	YTHDF3					OGG1				
610	KRT18					RMDN3				
611	INPPL1					PET117				
612	CAMK2D					MAOA				

	A	B	C	D	E	F	G	H	I	J
613	FNIP1					BCO2				
614	UBE2Q2					IFI27				
615	ROCK2					TMLHE				
616	TJP1					TOMM20				
617	EXOSC4					YRDC				
618	ARHGAP42					COA7				
619	KDSR					DHRS2				
620	GPR137					MMAA				
621	FAM50A					MIEF1				
622	METRN					LIG3				
623	AIMP2					FASTKD5				
624	UGCG					PARK7				
625	ZNF180					COX7B2				
626	FAM214A					SIRT4				
627	SDF2L1					UQCC2				
628	TBC1D10B					USP30				
629	KRT19					PDE12				
630	TMPPE					CYP24A1				
631	PATJ					PANK2				
632	EPB41L5					CA5A				
633	ZNF692					PRKACA				
634	AURKB					CPT1C				
635	MUS81					SND1				
636	CYP20A1					GLUD2				
637	CLDN7					PRDX4				
638	PELI1					MGARP				
639	SMC4					CYP11B2				
640	INO80B					PMAIP1				
641	GIT1					TDRKH				
642	PRKAR1B					SLC8B1				
643	APLF					CYP27B1				
644	CST3					POLG2				
645	CAB39					THEM5				
646	IL1RAP					BIK				
647	EIF3K					MTERF1				
648	H2AC21					MTFR2				
649	NUS1					TOMM20L				
650	ARHGAP45					OXCT2				
651	ARNTL2					ACSM2B				
652	UGGT1					MYO19				
653	PIK3CB					MGME1				
654	AKT1S1					EFHD1				
655	RBSN					APEX1				
656	TMEM101					SPTLC2				
657	YOD1					NEU4				
658	TMEM94					CASP9				
659	DCP2					AGK				
660	RITA1					MCL1				
661	APBB2					DUS2				
662	SNX8					PLD6				
663	ACTR3C					TOMM34				
664	XPR1					PLSCR3				
665	TMEM115					NDUFB1				
666	IFT81					BCL2L1				
667	ATP13A1					ARMC10				
668	USF3					THG1L				
669	CASKIN2					GOLPH3				
670	LSM11					FASTKD3				
671	FAM207A					CEP89				
672	ZBTB7B					GPAT2				
673	PLK1					BOK				
674	GTF3C3					CYP11B1				
675	NTMT1					BCL2				
676	SWAP70					BCL2A1				
677	FRK					BCL2L2				
678	ALDOA					CASP3				
679	CTTNBP2NL					DMPK				
680	KXD1					ALKBH1				

	A	B	C	D	E	F	G	H	I	J
681	SLC35A5					BCL2L10				
682	AP1B1					PABPC5				
683	KLHL24					PRSS35				
684	DDX51					SERAC1				
685	ZNF777					TRMT61B				
686	VPS28					PDE2A				
687	AZIN1					PLGRKT				
688	TMEM64					BCL2L11				
689	PLEKHG6					ARMCX3				
690	STK16					SPHKAP				
691	ASF1A					NAT8L				
692	UHRF2					MCCD1				
693	FAM20B									
694	RANBP2									
695	ARID5B									
696	NUDT16L1									
697	SBF1									
698	PRR12									
699	DNAAF5									
700	PRMT7									
701	PSMG3									
702	MDM1									
703	DPP3									
704	TKT									
705	SCAMP1									
706	FAM160B1									
707	THUMPD2									
708	KCTD17									
709	RAD1									
710	PPIL4									
711	PIAS2									
712	NEPRO									
713	SMIM22									
714	ZNF493									
715	ZNF566									
716	OAZ1									
717	PRAP1									
718	IRF2BP1									
719	INPP5E									
720	PBX4									
721	SNAPC3									
722	RNF13									
723	SLC38A10									
724	ALKBH4									
725	EIF5A2									
726	CHID1									
727	SART1									
728	MAST3									
729	NR2F6									
730	ZBTB48									
731	NOSIP									
732	CNPPD1									
733	CEP70									
734	FERMT2									
735	TMED10									
736	AP5Z1									
737	TM2D1									
738	CARD10									
739	SLC4A7									
740	WDFY1									
741	BPTF									
742	SEC24B									
743	DEAF1									
744	KRR1									
745	CMPK1									
746	EDC4									
747	TOR4A									
748	SLC39A5									

	A	B	C	D	E	F	G	H	I	J
749	LNPEP									
750	SEL1L									
751	CHMP4B									
752	FEZ2									
753	RPRD1A									
754	TEAD4									
755	YIPF2									
756	ZC3H3									
757	ANP32E									
758	TSHZ2									
759	NAPRT									
760	XIAP									
761	TMOD2									
762	TCP11L1									
763	MYCT1									
764	KCTD12									
765	ZNF808									
766	PNPLA2									
767	ADAM22									
768	FOXP4									
769	ENTPD6									
770	ANAPC10									
771	SPTY2D1									
772	MID1IP1									
773	DHRS3									
774	KCTD3									
775	ZNF267									
776	ST6GALNAC4									
777	SNX10									
778	EPS8L2									
779	RPP21									
780	DEDD2									
781	MFSD13A									
782	YIPF4									
783	ZNF316									
784	VTA1									
785	IGSF8									
786	FCHO2									
787	AGO3									
788	SIRT7									
789	FBXL17									
790	DPP7									
791	GCH1									
792	MED17									
793	AKAP17A									
794	CTDSPL2									
795	SCX									
796	PPP4C									
797	PRXL2B									
798	PRKCI									
799	CSNK1G3									
800	DDX6									
801	PURA									
802	ORC4									
803	PSMD3									
804	AOC1									
805	WBP2									
806	PRR22									
807	MZT2A									
808	PTBP2									
809	CYP7B1									
810	MEPCE									
811	PRDM2									
812	GPC6									
813	SHD									
814	MRC1									
815	SOS1									
816	KHSRP									

	A	B	C	D	E	F	G	H	I	J
817	RNF141									
818	ENPP4									
819	RAB6A									
820	RNF187									
821	ALKBH8									
822	GPR18									
823	MIDN									
824	EBLN2									
825	CPEB2									
826	SEC22A									
827	MALT1									
828	DVL1									
829	PACRGL									
830	POLR1H									
831	KLHL26									
832	PHRF1									
833	TESK1									
834	PMS1									
835	MBNL1									
836	SMG1									
837	ZMAT3									
838	FRS2									
839	DCP1A									
840	SNX17									
841	MAPK8IP3									
842	FAM204A									
843	KLHL11									
844	ZNF616									
845	MBLAC1									
846	POLI									
847	VPS9D1									
848	ASB6									
849	INTS2									
850	ANKRD50									
851	BTRC									
852	FANCM									
853	CC2D1A									
854	ZNF593									
855	FAM102B									
856	NEDD1									
857	IL32									
858	SLX4IP									
859	CNTRL									
860	NRP1									
861	RIT1									
862	MAZ									
863	USP37									
864	DOLK									
865	RPGRIP1L									
866	ARHGEF16									
867	PLEKHA1									
868	SHISA5									
869	AP3D1									
870	SCOC									
871	ZFP91									
872	ATG14									
873	WDR83									
874	APRT									
875	USP16									
876	ZNF197									
877	KMT2A									
878	ZNF484									
879	USP28									
880	UTP20									
881	AGFG1									
882	SNX27									
883	CSGALNACT2									
884	USE1									

	A	B	C	D	E	F	G	H	I	J
885	FCGR2A									
886	FAM110A									
887	SLC39A3									
888	CHURC1									
889	TMEM30A									
890	WDR43									
891	HIVEP2									
892	AAMP									
893	APAF1									
894	ZRANB3									
895	PSMD4									
896	AKAP13									
897	TGFBR3									
898	BARD1									
899	RSPH3									
900	RABGAP1									
901	LRWD1									
902	YIPF5									
903	RNF115									
904	SLC4A2									
905	ARSK									
906	PIDD1									
907	IFT80									
908	TAF2									
909	PSMC5									
910	MTMR9									
911	HERC4									
912	LARP4									
913	ZBTB37									
914	REV1									
915	RABL3									
916	MAP2K7									
917	RPS6KC1									
918	SLC35E4									
919	PEX10									
920	PDZK1IP1									
921	SDCBP									
922	SHROOM1									
923	B9D2									
924	EIF4E									
925	CCDC130									
926	JADE1									
927	VPS4A									
928	CCPG1									
929	RNF152									
930	ACTR1B									
931	DGAT1									
932	SMARCB1									
933	CEPT1									
934	ZBTB26									
935	ZNF865									
936	CCNT2									
937	PPP1R35									
938	SLC35F5									
939	TC2N									
940	RLF									
941	SNAPC1									
942	DST									
943	ERI1									
944	PPM1B									
945	SNX14									
946	FBRS									
947	UCKL1									
948	ZNF513									
949	INO80E									
950	MLH3									
951	LRSAM1									
952	SWSAP1									

	A	B	C	D	E	F	G	H	I	J
953	TIPARP									
954	PIK3R1									
955	SEPSECS									
956	E4F1									
957	PAFAH1B3									
958	MINK1									
959	VPS51									
960	MPHOSPH9									
961	SELENOI									
962	TTL									
963	ADAT3									
964	ACTG1									
965	JPT1									
966	DAGLA									
967	MARCHF9									
968	PPP1R37									
969	TNFSF4									
970	ELK4									
971	ZNF532									
972	PPDPF									
973	PKD2									
974	NELFA									
975	PAQR4									
976	ZGRF1									
977	DNTTIP1									
978	PUM2									
979	ETNK1									
980	MINDY3									
981	SLMAP									
982	DTWD2									
983	TICAM1									
984	BRI3									
985	GCFC2									
986	DRAP1									
987	TMEM147									
988	ZFX									
989	TRADD									
990	FILIP1L									
991	PLEC									
992	GPI									
993	ENPEP									
994	EXPH5									
995	GOLGB1									
996	FBXO38									
997	TTC39C									
998	ZEB1									
999	SNAPC4									
1000	LRRC57									
1001	ULK4									
1002	AMMECR1									
1003	KIDINS220									
1004	CDIN1									
1005	ZMYND11									
1006	RALGPS2									
1007	MPND									
1008	TRIP12									
1009	NBN									
1010	KEAP1									
1011	SMCHD1									
1012	HCFC2									
1013	HMGA1									
1014	RABGAP1L									
1015	ATRX									
1016	ACSS2									
1017	AJM1									
1018	ARPP19									
1019	C20orf27									
1020	PHPT1									

	A	B	C	D	E	F	G	H	I	J
1021	HECW2									
1022	CDYL2									
1023	REV3L									
1024	MYPOP									
1025	CCDC124									
1026	RAP1A									
1027	ARHGEF10L									
1028	HACE1									
1029	BLOC1S3									
1030	PSMD8									
1031	TIPRL									
1032	CDC42BPA									
1033	ZNF598									
1034	ITGB4									
1035	DHPS									
1036	SC5D									
1037	SOCS4									
1038	MVK									
1039	PDLIM5									
1040	ITCH									
1041	SURF2									
1042	DPP8									
1043	BOP1									
1044	ACTR2									
1045	ABRAXAS2									
1046	SLC2A8									
1047	AVPI1									
1048	ALG10									
1049	DIS3									
1050	SERTAD2									
1051	ZKSCAN3									
1052	FBXL20									
1053	SCRIB									
1054	MBD3									
1055	TP53I11									
1056	SF3B4									
1057	CEP295									
1058	FZD4									
1059	TADA1									
1060	ZMYM4									
1061	ARFGEF3									
1062	ANKRD9									
1063	USP33									
1064	EIF4B									
1065	GTF2F1									
1066	MGAT4B									
1067	ZBTB24									
1068	CYRIB									
1069	RPS21									
1070	TELO2									
1071	ERBIN									
1072	SLC2A4RG									
1073	ZNF124									
1074	PRDM10									
1075	RBMX									
1076	FANCB									
1077	INTS5									
1078	ATP2C1									
1079	YJU2									
1080	ACVR1									
1081	TMEM263									
1082	DOCK7									
1083	CD2BP2									
1084	GEN1									
1085	HELLS									
1086	SNX13									
1087	ZNF296									
1088	DR1									

	A	B	C	D	E	F	G	H	I	J
1089	SBF2									
1090	USP9X									
1091	EHBP1									
1092	KAT6A									
1093	BCAP29									
1094	HSPBP1									
1095	ZNF571									
1096	MBD5									
1097	PDXP									
1098	NOC3L									
1099	BACH1									
1100	IFITM3									
1101	NIN									
1102	GPR65									
1103	MED1									
1104	ZNF273									
1105	CYP2S1									
1106	ODF2L									
1107	EML2									
1108	STON1									
1109	LZTFL1									
1110	C20orf194									
1111	ABL2									
1112	ZMYM2									
1113	ATP6V0D1									
1114	ATP13A3									
1115	SEC22C									
1116	NBEAL1									
1117	RRAS									
1118	CUL4B									
1119	MBNL2									
1120	HIP1R									
1121	FIZ1									
1122	WIPF1									
1123	HCFC1R1									
1124	GMFB									
1125	HMG20A									
1126	ATF7									
1127	DOK1									
1128	NUDT3									
1129	AMD1									
1130	TRIM33									
1131	IHH									
1132	RBM27									
1133	ITPK1									
1134	PCDH1									
1135	LAMTOR1									
1136	CHERP									
1137	C18orf54									
1138	SPATA2L									
1139	VILL									
1140	TBK1									
1141	SPIN1									
1142	NUCB1									
1143	ENKD1									
1144	GIN1									
1145	RBM18									
1146	ANK3									
1147	SIKE1									
1148	ADGRF5									
1149	POLR2I									
1150	TAGLN2									
1151	PEX11G									
1152	CIP2A									
1153	SSH2									
1154	ZKSCAN1									
1155	COMMD4									
1156	RPAP2									

	A	B	C	D	E	F	G	H	I	J
1157	DGKE									
1158	WDR48									
1159	CCDC117									
1160	PPIP5K2									
1161	LRFN3									
1162	TGFBR1									
1163	ZFYVE27									
1164	FBXL12									
1165	KLHL15									
1166	C5orf22									
1167	SGSM3									
1168	KCTD5									
1169	NACC1									
1170	ZNF213									
1171	ZGPAT									
1172	SLC35A2									
1173	CDHR5									
1174	MMP15									
1175	MANEA									
1176	RAD23A									
1177	TBC1D8B									
1178	KRT8									
1179	FXYD3									
1180	BDP1									
1181	EPM2A									
1182	TSSC4									
1183	OGFR									
1184	FAM189B									
1185	RSRC2									
1186	C12orf29									
1187	POLD1									
1188	FBXL19									
1189	ZNF628									
1190	TNK2									
1191	ZNF766									
1192	GALE									
1193	DHX36									
1194	SEPTIN2									
1195	IRF3									
1196	PPP2R1B									
1197	MBTPS2									
1198	TCF3									
1199	INO80D									
1200	GTF2A1									
1201	U2SURP									
1202	PMVK									
1203	ATP2B4									
1204	PANX1									
1205	USP5									
1206	SPRTN									
1207	FAM3C									
1208	SFMBT2									
1209	RBCK1									
1210	LPAR2									
1211	TRMT10A									
1212	PRPF39									
1213	NEMF									
1214	PRKAB2									
1215	UBE2K									
1216	NRIP1									
1217	RFX1									
1218	GPBP1									
1219	TRIM5									
1220	RHBDL1									
1221	FN3K									
1222	SNX1									
1223	RTTN									
1224	KIN									

	A	B	C	D	E	F	G	H	I	J
1225	FMN1									
1226	ZNF35									
1227	RAPGEF2									
1228	H2AC14									
1229	TRIM3									
1230	AHCTF1									
1231	RHOG									
1232	SPINT1									
1233	ZNF784									
1234	CAPN1									
1235	SINHCAF									
1236	POLR2H									
1237	MBOAT7									
1238	EXOSC5									
1239	ALS2									
1240	TIAL1									
1241	NOL12									
1242	DNAL1									
1243	PLXNB2									
1244	GNG12									
1245	DZIP3									
1246	TMED1									
1247	PRPF31									
1248	ANAPC15									
1249	RBM4									
1250	CEP57L1									
1251	DCUN1D4									
1252	DCLRE1A									
1253	PHLDB2									
1254	SLC35B4									
1255	UGGT2									
1256	TLCD5									
1257	UBL7									
1258	SEPTIN9									
1259	PIK3CA									
1260	BICC1									
1261	ZNF10									
1262	SLC35C1									
1263	PAF1									
1264	TMED5									
1265	MOB1B									
1266	FUT11									
1267	KLHDC3									
1268	EXOC3									
1269	SCARB1									
1270	ZNF248									
1271	GPAA1									
1272	YAF2									
1273	ING3									
1274	ZNF449									
1275	EDF1									
1276	PPP1R14D									
1277	EIF2B4									
1278	WDHD1									
1279	RBM12									
1280	NCKIPSD									
1281	PPHLN1									
1282	MAPKAPK3									
1283	EMD									
1284	C1orf159									
1285	ATP13A2									
1286	ZC3HAV1									
1287	KLHL5									
1288	NDOR1									
1289	GCC2									
1290	PPP2R5C									
1291	SRSF11									
1292	EFCAB7									

	A	B	C	D	E	F	G	H	I	J
1293	FBXL15									
1294	MDFIC									
1295	B3GNT5									
1296	SLC30A7									
1297	AEBP2									
1298	PRR15									
1299	TBC1D9B									
1300	DYNC1LI2									
1301	PTBP1									
1302	ZNF451									
1303	ZNF567									
1304	ARSA									
1305	ZNF354A									
1306	CCDC15									
1307	LZTR1									
1308	VASP									
1309	ELMO3									
1310	UBR3									
1311	RAB23									
1312	HAUS6									
1313	ZBTB21									
1314	PAWR									
1315	CLCN7									
1316	CD2AP									
1317	KLC2									
1318	NEK4									
1319	LRRC58									
1320	MARCHF7									
1321	TBC1D19									
1322	RPAP3									
1323	ZC2HC1A									
1324	LIPA									
1325	ZNF280C									
1326	ASL									
1327	MLLT1									
1328	NINJ1									
1329	PTPRG									
1330	SH3GL1									
1331	MAN1A2									
1332	TONSL									
1333	SCARB2									
1334	ABHD13									
1335	STRN3									
1336	JCAD									
1337	KIF2A									
1338	KHK									
1339	ZNF787									
1340	TMED2									
1341	ARAP2									
1342	BNIP2									
1343	BPNT2									
1344	FAM83B									
1345	ZYX									
1346	ZFP30									
1347	ELF2									
1348	ASPH									
1349	ABHD18									
1350	ATXN2L									
1351	L3MBTL3									
1352	FAM149B1									
1353	P4HTM									
1354	ZNF268									
1355	GTF3C5									
1356	ZNF160									
1357	NKTR									
1358	ACER3									
1359	LMBR1									
1360	ITGB1									

	A	B	C	D	E	F	G	H	I	J
1361	IBTK									
1362	FBXO48									
1363	GNPTAB									
1364	PPP3CB									
1365	GPRIN3									
1366	LEPROT									
1367	NFYB									
1368	ERCC4									
1369	TENT2									
1370	ZNF341									
1371	PDE4DIP									
1372	NUCKS1									
1373	BLOC1S4									
1374	RNF38									
1375	POLR2J									
1376	TYW3									
1377	ATG101									
1378	FLT1									
1379	IMPACT									
1380	PLXNB1									
1381	RAB3IP									
1382	EIF5A									
1383	TSNAX									
1384	TK1									
1385	SNIP1									
1386	CRYBG3									
1387	EYA3									
1388	MS4A7									
1389	ATP6V1F									
1390	SPG11									
1391	IL1R1									
1392	H4C4									
1393	SLC38A6									
1394	ST14									
1395	STYX									
1396	ZNF84									
1397	ULK3									
1398	ATP10D									
1399	CHIC1									
1400	MDN1									
1401	KLHL2									
1402	RAB25									
1403	RANBP3									
1404	PIH1D1									
1405	MEN1									
1406	PAK4									
1407	CUL3									
1408	WSB1									
1409	SLC9A7									
1410	SESTD1									
1411	RAB11FIP2									
1412	DMWD									
1413	ZNF430									
1414	KCTD9									
1415	TMTC3									
1416	SLC52A3									
1417	MON2									
1418	ATE1									
1419	ZNF581									
1420	RANBP9									
1421	POLR2E									
1422	TMEM237									
1423	TASOR									
1424	HNRNPAB									
1425	EIF3G									
1426	NHP2									
1427	PUS7L									
1428	RUNX2									

	A	B	C	D	E	F	G	H	I	J
1429	C19orf53									
1430	CCDC50									
1431	ARHGAP21									
1432	RAB20									
1433	SCFD1									
1434	ETS1									
1435	DNAJC10									
1436	AP3M1									
1437	KRTCAP3									
1438	ANAPC11									
1439	EVI2A									
1440	TRIM23									
1441	BORCS8									
1442	CALU									
1443	ZNF641									
1444	SYDE2									
1445	SGCD									
1446	HYAL2									
1447	UBQLN1									
1448	API5									
1449	MTMR2									
1450	PRRC1									
1451	ZNF117									
1452	UNC45A									
1453	MAP2K4									
1454	VPS54									
1455	SLC39A10									
1456	MAP3K7									
1457	ANGPTL1									
1458	MOGAT3									
1459	IGIP									
1460	TRAPPC13									
1461	WDR36									
1462	CCDC24									
1463	TTBK2									
1464	CTSO									
1465	USP46									
1466	CYHR1									
1467	CDK16									
1468	INPP5J									
1469	MFSD12									
1470	NMD3									
1471	C5orf15									
1472	GLI3									
1473	MDM2									
1474	NEMP1									
1475	CIZ1									
1476	MFSD14A									
1477	MMS22L									
1478	ATXN7L1									
1479	ABCA1									
1480	ITPR2									
1481	PAQR3									
1482	SAPCD2									
1483	EEF1AKMT2									
1484	POU2F1									
1485	STAG2									
1486	PRPF40A									
1487	ELMOD2									
1488	SMNDC1									
1489	CNOT4									
1490	PDZD8									
1491	MIER1									
1492	MCRS1									
1493	FBXW7									
1494	DBNDD2									
1495	TMEM170B									
1496	CENPM									

	A	B	C	D	E	F	G	H	I	J
1497	KIFC2									
1498	ZDHHC1									
1499	NPHP3									
1500	ABI1									
1501	FUT10									
1502	DOCK10									
1503	PCNP									
1504	CACTIN									
1505	CPEB4									
1506	CCDC82									
1507	BLVRB									
1508	ZNF326									
1509	ZP3									
1510	VPS13C									
1511	EIF2AK3									
1512	SLC39A6									
1513	PHF20L1									
1514	CNKSR1									
1515	GPATCH11									
1516	NCOA1									
1517	EIF3B									
1518	STEAP4									
1519	ACY1									
1520	CERKL									
1521	LTBP4									
1522	RNF181									
1523	UBXN1									
1524	ERO1A									
1525	SEC13									
1526	THOC1									
1527	SMARCA5									
1528	NCKAP1									
1529	TLR5									
1530	TANC1									
1531	AASDHPPT									
1532	SMC5									
1533	ZNF292									
1534	AP1M2									
1535	MITF									
1536	TMEM182									
1537	ZC3H6									
1538	FANCL									
1539	ATM									
1540	TRPT1									
1541	POLD4									
1542	IGSF9									
1543	GAPDH									
1544	SCAMP4									
1545	ZBTB6									
1546	UBE2W									
1547	RNF126									
1548	P2RY2									
1549	HNRNPLL									
1550	ARIH1									
1551	CNN2									
1552	PARD6A									
1553	POLK									
1554	GAK									
1555	ADGRE5									
1556	ANO6									
1557	CDK5									
1558	CCDC12									
1559	CYB561D2									
1560	HS3ST3B1									
1561	MAP3K10									
1562	TMEM104									
1563	BICD1									
1564	DCTN4									

	A	B	C	D	E	F	G	H	I	J
1565	PSMB4									
1566	CFH									
1567	ABCF3									
1568	SRP9									
1569	PHACTR2									
1570	RNPEPL1									
1571	USF1									
1572	CNTLN									
1573	NRARP									
1574	RANGAP1									
1575	PRADC1									
1576	MAPK1IP1L									
1577	JMY									
1578	ZNF286A									
1579	FAM169A									
1580	EMC9									
1581	RESF1									
1582	CUL5									
1583	RBL2									
1584	OGA									
1585	PIM3									
1586	WDR26									
1587	PTER									
1588	TMEM135									
1589	ARFGAP2									
1590	PGAP6									
1591	AGL									
1592	RNF19A									
1593	GPM6B									
1594	WDR24									
1595	FBXL5									
1596	EXOC6									
1597	ZNF597									
1598	PPP1R21									
1599	CDC34									
1600	C1orf35									
1601	RASGEF1B									
1602	CACNA2D1									
1603	GIT2									
1604	DCUN1D1									
1605	NR2C1									
1606	NME7									
1607	ABRAXAS1									
1608	POC1B									
1609	ATL2									
1610	CHD2									
1611	UBR2									
1612	DHCR7									
1613	MAPK6									
1614	SELENOH									
1615	INVS									
1616	SSR1									
1617	PWWP2A									
1618	AUP1									
1619	DDX59									
1620	C9orf16									
1621	MLST8									
1622	EFCAB14									
1623	CNOT2									
1624	UBE3A									
1625	GOLGA4									
1626	CIAO3									
1627	ZNF703									
1628	TTC38									
1629	DDX60L									
1630	CSTF2T									
1631	FBXW9									
1632	FAAH									

	A	B	C	D	E	F	G	H	I	J
1633	PSMB6									
1634	EPG5									
1635	PITPNM1									
1636	VPS37C									
1637	TMEM184A									
1638	PRKD3									
1639	STK11IP									
1640	CBLB									
1641	ACTB									
1642	HMCN1									
1643	ADAM17									
1644	PTPN12									
1645	SKIL									
1646	EPS15									
1647	FHOD1									
1648	TMEM106B									
1649	NAGPA									
1650	ZNF26									
1651	AGO1									
1652	SNX29									
1653	HPS3									
1654	APOL6									
1655	DDX21									
1656	MORF4L1									
1657	ERO1B									
1658	SS18									
1659	PDIK1L									
1660	CAPNS1									
1661	DEF8									
1662	ZNF214									
1663	FAM83H									
1664	RALY									
1665	VCAN									
1666	LENG9									
1667	UNC93B1									
1668	DCAF15									
1669	ACD									
1670	NADK									
1671	TWF1									
1672	EDEM1									
1673	SASS6									
1674	TECR									
1675	TOM1									
1676	NAPEPLD									
1677	PRKCH									
1678	DHX15									
1679	AMOTL1									
1680	GAB1									
1681	MCM9									
1682	KATNAL1									
1683	H2AJ									
1684	ALG9									
1685	ZNF654									
1686	CCDC86									
1687	MKLN1									
1688	ATXN7									
1689	CNOT3									
1690	FADD									
1691	ERCC2									
1692	SNRNP25									
1693	TSEN34									
1694	PLEKHA3									
1695	GATD1									
1696	CLASRP									
1697	NRBF2									
1698	FEM1C									
1699	GJC1									
1700	RNASEK									

	A	B	C	D	E	F	G	H	I	J
1701	ZNF684									
1702	SPATA7									
1703	ZNF639									
1704	ZNF852									
1705	ZNF48									
1706	HMBS									
1707	PIK3C2A									
1708	EEA1									
1709	TOGARAM1									
1710	TNK1									
1711	RASGRP1									
1712	ZNF408									
1713	SMARCAD1									
1714	CCDC85B									
1715	FAT4									
1716	ZNF501									
1717	MYO15B									
1718	RAD51B									
1719	RFC1									
1720	POGLUT1									
1721	NSL1									
1722	KCTD20									
1723	CLPTM1L									
1724	ZNF717									
1725	TRIM38									
1726	SETD1A									
1727	SGSH									
1728	GXYLT2									
1729	MCTP1									
1730	STARD10									
1731	B3GNT3									
1732	PRXL2C									
1733	INAFM1									
1734	TMEM187									
1735	LRRC45									
1736	STX12									
1737	RFXANK									
1738	AGTRAP									
1739	SP100									
1740	PBX1									
1741	N4BP2									
1742	ZNF189									
1743	MSANTD4									
1744	ANKRD28									
1745	AKAP11									
1746	TBCB									
1747	SMARCA4									
1748	G3BP1									
1749	USP34									
1750	TRMT61A									
1751	NSUN5									
1752	ZRANB2									
1753	APPL1									
1754	LRRC8C									
1755	RAB30									
1756	NME2									
1757	ASXL2									
1758	AMDHD2									
1759	BAIAP2L2									
1760	KMT5C									
1761	POMK									
1762	PDLIM1									
1763	ZFAND4									
1764	STK25									
1765	LSS									
1766	TIA1									
1767	KLF3									
1768	PIGN									

	A	B	C	D	E	F	G	H	I	J
1769	USP49									
1770	TRIM37									
1771	PARP14									
1772	AGGF1									
1773	DDX41									
1774	FRA10AC1									
1775	SLC35C2									
1776	H2BC14									
1777	C7orf50									
1778	SPOUT1									
1779	TMEM219									
1780	ITPR1									
1781	NFAT5									
1782	RIC1									
1783	CHMP6									
1784	MEGF9									
1785	MAPK7									
1786	SNX16									
1787	MFSD10									
1788	ZBTB8A									
1789	DHX40									
1790	DENND5A									
1791	MXD3									
1792	CDK2AP2									
1793	APOBR									
1794	EXOC6B									
1795	SMARCA1									
1796	DUS1L									
1797	ZNF444									
1798	ZBTB7A									
1799	BMP2K									
1800	CD84									
1801	CPSF6									
1802	STK40									
1803	RNF223									
1804	RRNAD1									
1805	PUS10									
1806	CDK11B									
1807	ZNF527									
1808	REXO1									
1809	ATP6AP1									
1810	BCL7B									
1811	PAPOLG									
1812	RPL18									
1813	RANBP17									
1814	ADGRL4									
1815	MZT2B									
1816	PCBP4									
1817	CDV3									
1818	PSIP1									
1819	AP1S3									
1820	BCLAF1									
1821	LMAN2									
1822	SPSB3									
1823	TRAPPC6A									
1824	MMP24OS									
1825	DCAF17									
1826	LCMT1									
1827	HGH1									
1828	ZNF845									
1829	KLHL9									
1830	BAG6									
1831	ICE2									
1832	NAB1									
1833	TPR									
1834	FAM120B									
1835	RPL8									
1836	POMT1									

	A	B	C	D	E	F	G	H	I	J
1837	MROH6									
1838	SNAPC2									
1839	RCHY1									
1840	F12									
1841	GAPVD1									
1842	DTWD1									
1843	BRWD1									
1844	SEC62									
1845	OTUB1									
1846	PPP4R2									
1847	MIER2									
1848	SH3GLB2									
1849	ZBTB1									
1850	FKTN									
1851	ZSCAN30									
1852	CCDC22									
1853	NFIB									
1854	RPS9									
1855	DXO									
1856	CMTM6									
1857	MTMR10									
1858	ZBTB41									
1859	CCNQ									
1860	FAM114A2									
1861	ERI2									
1862	MARF1									
1863	MFSD3									
1864	GLI4									
1865	MCOLN1									
1866	PMM1									
1867	H1-10									
1868	LEMD2									
1869	R3HCC1L									
1870	HIPK2									
1871	MED28									
1872	HACD4									
1873	KIAA1958									
1874	SDC1									
1875	PDE5A									
1876	LZIC									
1877	INPP4B									
1878	ZWILCH									
1879	BTBD7									
1880	CENPE									
1881	FUBP1									
1882	RASA2									
1883	TAF1D									
1884	SLC8A1									
1885	ZNF91									
1886	ZNF837									
1887	RPUSD1									
1888	GIPC1									
1889	SLK									
1890	THEM6									
1891	DCAF10									
1892	FGD4									
1893	DYNC2I2									
1894	MYDGF									
1895	ADAP1									
1896	IFRD2									
1897	CCNI									
1898	SSNA1									
1899	RBFOX2									
1900	BOLA2B									
1901	PHKG2									
1902	POLD2									
1903	VCPIP1									
1904	SSPN									

	A	B	C	D	E	F	G	H	I	J
1905	METTL14									
1906	LTBR									
1907	PAXX									
1908	CPNE2									
1909	ZNF146									
1910	NXPE3									
1911	ENGASE									
1912	PLEKHA5									
1913	JMJD1C									
1914	PRR13									
1915	EOGT									
1916	ITFG1									
1917	PGGT1B									
1918	DUSP23									
1919	PRR5									
1920	NOB1									
1921	TUT7									
1922	RBM15									
1923	QKI									
1924	RRP9									
1925	VARS1									
1926	RAD9A									
1927	TRMT1L									
1928	PLOD3									
1929	TPRN									
1930	RHOC									
1931	CD81									
1932	MXD4									
1933	DYRK2									
1934	UBL5									
1935	ATXN7L3B									
1936	BRIP1									
1937	TSPAN15									
1938	PPP1CA									
1939	SERF2									
1940	CIB1									
1941	AIDA									
1942	MBNL3									
1943	ZFYVE16									
1944	HDAC11									
1945	MANBA									
1946	CBFB									
1947	CRTC1									
1948	LMF2									
1949	KIF21A									
1950	SMURF2									
1951	TTC14									
1952	STX6									
1953	PFN1									
1954	ELL2									
1955	PPM1G									
1956	SCAMP2									
1957	VEZT									
1958	PTPN4									
1959	FARSA									
1960	IL7R									
1961	PYCR3									
1962	UBA5									
1963	SETD2									
1964	RAB5A									
1965	COG5									
1966	MAP4K5									
1967	ZNF70									
1968	AKAP9									
1969	PRKG1									
1970	TRMT2A									
1971	ZNF720									
1972	KIF5B									

	A	B	C	D	E	F	G	H	I	J
1973	CCP110									
1974	BMI1									
1975	AP1M1									
1976	PDE1A									
1977	GSAP									
1978	HBP1									
1979	CRTC2									
1980	NAMPT									
1981	SMAD5									
1982	CCDC186									
1983	UHRF1BP1									
1984	SPATA6									
1985	MISP3									
1986	UBE3D									
1987	HPCAL1									
1988	ZNF510									
1989	EMCN									
1990	REXO4									
1991	ETAA1									
1992	INTS1									
1993	ELOF1									
1994	PPP2R5B									
1995	DTD2									
1996	SP4									
1997	CNEP1R1									
1998	ITGAV									
1999	SLC9A3R2									
2000	DENND11									
2001	BPGM									
2002	ZNF708									
2003	NBL1									
2004	BBS9									
2005	PRSS48									
2006	DOCK8									
2007	USO1									
2008	CLIP4									
2009	TOP2B									
2010	KNL1									
2011	CLK4									
2012	GPATCH2L									
2013	LPIN3									
2014	SEPTIN7									
2015	ROCK1									
2016	MEX3D									
2017	SERBP1									
2018	TAB3									
2019	MICAL1									
2020	SNRPD2									
2021	SNX6									
2022	PEAK1									
2023	DNAJC24									
2024	CCDC138									
2025	DNAJB14									
2026	PAK2									
2027	TRPM7									
2028	ZFAND6									
2029	ZNF579									
2030	GIGYF2									
2031	AFF1									
2032	GUCY1A1									
2033	TMEM167B									
2034	SRP72									
2035	CC2D2A									
2036	ZRANB1									
2037	SAMSN1									
2038	CTBS									
2039	ATP11B									
2040	UPF1									

	A	B	C	D	E	F	G	H	I	J
2041	CRLF3									
2042	C11orf68									
2043	SPATS2									
2044	ATXN1									
2045	TALDO1									
2046	CORO1B									
2047	FSD1L									
2048	ESRRA									
2049	SCAF1									
2050	ANKRD61									
2051	PPAN									
2052	CD151									
2053	TRA2B									
2054	DIP2B									
2055	DPH2									
2056	RNF11									
2057	RNF217									
2058	EMSY									
2059	WRN									
2060	ALYREF									
2061	KTN1									
2062	ZNF609									
2063	ARMT1									
2064	FTO									
2065	WAPL									
2066	POLR2L									
2067	TBCEL									
2068	ORAI1									
2069	DPH7									
2070	VPS8									
2071	VAC14									
2072	UBXN7									
2073	SASH1									
2074	ZNF619									
2075	ZFAND5									
2076	SCAI									
2077	TAB2									
2078	RNF25									
2079	TUBG1									
2080	RPLP2									
2081	ITGA1									
2082	RABAC1									
2083	AP1S1									
2084	ALG3									
2085	CREBL2									
2086	ARL5B									
2087	TRMT13									
2088	NADSYN1									
2089	DEPDC1									
2090	MROH1									
2091	ZNF611									
2092	RNF111									
2093	LSM4									
2094	C2orf42									
2095	CEP290									
2096	ZNF580									
2097	DHX37									
2098	FBXW2									
2099	CPLANE1									
2100	RHBDD2									
2101	MAN1B1									
2102	KIF20B									
2103	COG4									
2104	ZNF131									
2105	MON1A									
2106	DDX17									
2107	FGFR1OP2									
2108	ENTPD1									

	A	B	C	D	E	F	G	H	I	J
2109	FZD6									
2110	PPP4R3A									
2111	RBM43									
2112	MEX3C									
2113	HOMER1									
2114	MIS18BP1									
2115	GNA11									
2116	ZBTB14									
2117	VGLL3									
2118	TOPORS									
2119	ZNF322									
2120	KCNK5									
2121	STK3									
2122	NAA60									
2123	RBMXL1									
2124	KIAA0586									
2125	TUT1									
2126	MYSM1									
2127	HEATR5B									
2128	SLC35A4									
2129	AHR									
2130	ZNF699									
2131	GHDC									
2132	OSBPL5									
2133	STAM2									
2134	ATF7IP									
2135	TCEA1									
2136	PPP6R1									
2137	S100PBP									
2138	AP4E1									
2139	SAC3D1									
2140	SLC5A3									
2141	TRIM11									
2142	EHMT2									
2143	SREBF1									
2144	ARHGAP18									
2145	ADD3									
2146	PGM2L1									
2147	WWC2									
2148	ZNF33B									
2149	LPCAT4									
2150	HSPA13									
2151	GTF3C4									
2152	UVRAG									
2153	KLHL20									
2154	RING1									
2155	CSTB									
2156	DPP9									
2157	UBA3									
2158	SCAMP3									
2159	ARNT									
2160	TOR2A									
2161	PCGF1									
2162	XPO1									
2163	ELF3									
2164	ANAPC1									
2165	PRSS8									
2166	TLK1									
2167	BTAF1									
2168	SULT2B1									
2169	NAA38									
2170	PIK3C3									
2171	SMAD4									
2172	TAF1A									
2173	DYNC2LI1									
2174	SH2D3A									
2175	CCND3									
2176	DIS3L									

	A	B	C	D	E	F	G	H	I	J
2177	RHOQ									
2178	FNBP1L									
2179	SDE2									
2180	MSRB1									
2181	PPP1R16A									
2182	ENTPD7									
2183	GPBP1L1									
2184	DECR2									
2185	CCS									
2186	G6PC3									
2187	RPS19									
2188	PLCB3									
2189	CREB1									
2190	NECAB3									
2191	B3GNT8									
2192	UHRF1BP1L									
2193	THUMPD1									
2194	ADAM28									
2195	KIF27									
2196	IFITM1									
2197	MED21									
2198	MTAP									
2199	FBXW11									
2200	MED26									
2201	CCNE2									
2202	SLC29A2									
2203	GIMAP4									
2204	THAP7									
2205	DDX20									
2206	DGKZ									
2207	THRAP3									
2208	PSMB7									
2209	PBRM1									
2210	PREB									
2211	ANKRD13D									
2212	FNDC3B									
2213	CORO7									
2214	SVIP									
2215	DDA1									
2216	CARNMT1									
2217	ARPC1B									
2218	SLC27A4									
2219	CCNC									
2220	INPP5F									
2221	CDKN2D									
2222	EFR3A									
2223	PES1									
2224	SDAD1									
2225	NHLRC2									
2226	TMEM222									
2227	OSGIN2									
2228	CYBC1									
2229	ZNF17									
2230	USP24									
2231	HPS1									
2232	RECK									
2233	CAPN7									
2234	KIAA0895									
2235	C5orf52									
2236	TPRA1									
2237	SACM1L									
2238	PLPP2									
2239	DUS3L									
2240	MVP									
2241	NOC4L									
2242	ATMIN									
2243	AXIN1									
2244	OSBPL9									

	A	B	C	D	E	F	G	H	I	J
2245	PTBP3									
2246	TMEM125									
2247	MMGT1									
2248	ZBTB11									
2249	GTF2H1									
2250	SPOPL									
2251	MYO6									
2252	PCF11									
2253	DAAM1									
2254	ZFP14									
2255	ARHGAP31									
2256	B4GALT7									
2257	PFKL									
2258	ZNHIT2									
2259	CACUL1									
2260	TLE5									
2261	KIAA2013									
2262	ILVBL									
2263	RTN4R									
2264	RBBP4									
2265	PDCD1LG2									
2266	SMPD2									
2267	NUDT21									
2268	SYVN1									
2269	PRKD2									
2270	AP1AR									
2271	ATP11C									
2272	MINDY2									
2273	ZNF678									
2274	FLOT2									
2275	FAM76B									
2276	AP2S1									
2277	RARB									
2278	GFUS									
2279	PHETA1									
2280	PIP5K1C									
2281	ASPSCR1									
2282	EPC2									
2283	BICRAL									
2284	ATF1									
2285	SNRPA									
2286	HDGFL2									
2287	LENG1									
2288	ASCC3									
2289	USP31									
2290	GNAQ									
2291	TENT4B									
2292	PRR14									
2293	SECISBP2L									
2294	PHC3									
2295	LMAN1									
2296	C4orf48									
2297	PAXBP1									
2298	DGKH									
2299	TFPT									
2300	PCMTD1									
2301	ZC3H7A									
2302	RNASEH2C									
2303	DBNL									
2304	ZNF226									
2305	TNFAIP8									
2306	CRACR2B									
2307	PIGT									
2308	TMEM129									
2309	REX1BD									
2310	PPP1R13L									
2311	URM1									
2312	H4-16									

	A	B	C	D	E	F	G	H	I	J
2313	CACFD1									
2314	ERCC8									
2315	HP1BP3									
2316	KDM6A									
2317	CLIP1									
2318	BCL7C									
2319	ZNF697									
2320	RIC8B									
2321	SCYL2									
2322	TAF10									
2323	ZBTB45									
2324	MORC3									
2325	PCYOX1									
2326	MICALL2									
2327	PANK4									
2328	DPH1									
2329	GAS6									
2330	HSD11B2									
2331	SPSB2									
2332	SARNP									
2333	TIFA									
2334	PRKCSH									
2335	NLK									
2336	EPS8L3									
2337	SPIRE2									
2338	KATNBL1									
2339	AMN									
2340	BMPR1A									
2341	TCF12									
2342	ZNF136									
2343	TNFRSF14									
2344	CDC40									
2345	CIAO2B									
2346	UBLCP1									
2347	METTL21A									
2348	SEPTIN10									
2349	RAB12									
2350	NAA30									
2351	TCP11L2									
2352	JAK1									
2353	CLPTM1									
2354	NCLN									
2355	NSD1									
2356	RFX7									
2357	GTF2I									
2358	SLC26A6									
2359	DPM3									
2360	SMC6									
2361	SH2B2									
2362	SBNO2									
2363	SHKBP1									
2364	APEH									
2365	RFNG									
2366	HNF1A									
2367	ANGPT2									
2368	TMEM54									
2369	LINS1									
2370	PPIG									
2371	LIME1									
2372	ZNF780B									
2373	SNORC									
2374	TBC1D12									
2375	ERGIC3									
2376	DOCK6									
2377	TEPSIN									
2378	NUTF2									
2379	SF3A2									
2380	ZNF222									

	A	B	C	D	E	F	G	H	I	J
2381	BICDL2									
2382	GALK1									
2383	PPWD1									
2384	ACTR6									
2385	MST1R									
2386	ANOS1									
2387	PCIF1									
2388	MTMR12									
2389	PEX16									
2390	ARL8B									
2391	LYPLA2									
2392	ZNF664									
2393	B3GNT2									
2394	LPAR6									
2395	SLFN11									
2396	BTBD6									
2397	RBM48									
2398	BLZF1									
2399	PDE8B									
2400	GABPB1									
2401	BIRC2									
2402	RSF1									
2403	P2RY11									
2404	FAM172A									
2405	IPO13									
2406	VPS13B									
2407	CEP120									
2408	TMUB1									
2409	GPR180									
2410	RIMKLB									
2411	C18orf25									
2412	HAUS3									
2413	MOB2									
2414	CALCRL									
2415	IL17RE									
2416	RNF44									
2417	CILK1									
2418	CALD1									
2419	PAFAH1B1									
2420	AP5M1									
2421	CEP44									
2422	RICTOR									
2423	DRAM1									
2424	SENP7									
2425	CPEB3									
2426	SH2B1									
2427	KIAA1109									
2428	GPR35									
2429	ASB13									
2430	CLASP2									
2431	TMEM39A									
2432	RORA									
2433	LSM7									
2434	FBXO30									
2435	DIXDC1									
2436	FBXL6									
2437	SLC19A1									
2438	SYNRG									
2439	H2AX									
2440	RABL6									
2441	ZNF721									
2442	RPS6KA4									
2443	CEP128									
2444	ENAH									
2445	GABPA									
2446	RC3H1									
2447	CNST									
2448	RALGAPA1									

	A	B	C	D	E	F	G	H	I	J
2449	DMXL1									
2450	GK5									
2451	ODR4									
2452	LIN54									
2453	USP25									
2454	VPS41									
2455	SMIM29									
2456	MAP4K3									
2457	TBC1D5									
2458	ALPK1									
2459	ZNF860									
2460	ATP2B1									
2461	ZNF24									
2462	CDK14									
2463	TCAF1									
2464	EAF1									
2465	ZNF280D									
2466	TOR1AIP2									
2467	PURB									
2468	FBXL14									
2469	CLN6									
2470	SRBD1									
2471	RYBP									
2472	NR2C2AP									
2473	APH1A									
2474	CEP170B									
2475	PIEZO1									
2476	ERLEC1									
2477	SLC22A18									
2478	WDR90									
2479	DCK									
2480	FAM83E									
2481	ACBD4									
2482	ARID2									
2483	FAM126B									
2484	DCUN1D3									
2485	MISP									
2486	ZBTB34									
2487	TMEM175									
2488	MIF									
2489	CYLD									
2490	LIMS1									
2491	TRDMT1									
2492	SMPD3									
2493	PSKH1									
2494	WTAP									
2495	DYNLRB1									
2496	PRKAA1									
2497	UBA6									
2498	FAM98B									
2499	B3GALNT1									
2500	SLC30A1									
2501	CEP170									
2502	SLC2A13									
2503	ALG13									
2504	GMPPA									
2505	TMUB2									
2506	BICRA									
2507	ETV1									
2508	SOCS6									
2509	EVA1B									
2510	DENND6A									
2511	PCGF5									
2512	SETX									
2513	TRAM1									
2514	TSPYL1									
2515	DNAJC18									
2516	TOLLIP									

	A	B	C	D	E	F	G	H	I	J
2517	IL6ST									
2518	WNK1									
2519	LMNTD2									
2520	ERGIC2									
2521	CHML									
2522	MAK16									
2523	USP1									
2524	RASGRP3									
2525	RCE1									
2526	TMED7									
2527	TTLL5									
2528	FOXJ3									
2529	SRFBP1									
2530	TMEM161B									
2531	AASDH									
2532	SPATA2									
2533	ZNF81									
2534	ZNF624									
2535	ZNF644									
2536	MOSMO									
2537	SLC3A2									
2538	LAD1									
2539	ECM2									
2540	FA2H									
2541	HDAC10									
2542	TRIP10									
2543	RCAN3									
2544	CELF1									
2545	RBM7									
2546	RTKN2									
2547	ARFGAP1									
2548	PTPN22									
2549	UHMK1									
2550	PAPOLA									
2551	DDI2									
2552	ADAM15									
2553	SPCS3									
2554	HECTD1									
2555	PIAS4									
2556	STRN									
2557	RELA									
2558	NEK7									
2559	INHBA									
2560	ARPC5L									
2561	TMEM259									
2562	FAM135A									
2563	CDC42EP5									
2564	SSR4									
2565	ZNF714									
2566	MOB1A									
2567	TUT4									
2568	FAAP20									
2569	LTN1									
2570	SNRNP48									
2571	ZNF680									
2572	CCDC126									
2573	MLF2									
2574	MED16									
2575	FBXO46									
2576	DLC1									
2577	RPE									
2578	LCOR									
2579	UBE2D3									
2580	PDS5A									
2581	GBE1									
2582	SREK1									
2583	ACAP2									
2584	CLHC1									

	A	B	C	D	E	F	G	H	I	J
2585	SGTB									
2586	ALCAM									
2587	TM4SF5									
2588	STEAP2									
2589	DHRS11									
2590	LGALS8									
2591	PRMT9									
2592	HELZ									
2593	EVPL									
2594	POLL									
2595	PPT1									
2596	TMEM184C									
2597	NMRAL1									
2598	CLTB									
2599	SLC38A9									
2600	AP2A1									
2601	SLF2									
2602	ACY3									
2603	PPM1A									
2604	ABI3BP									
2605	NUP160									
2606	USP3									
2607	IKBIP									
2608	SLC37A4									
2609	CENPX									
2610	LANCL1									
2611	KDM5A									
2612	UBE2J2									
2613	KMT2C									
2614	EPC1									
2615	ZBTB33									
2616	ZNF148									
2617	SLC39A13									
2618	NCOA3									
2619	CEP131									
2620	SCRN2									
2621	SGCB									
2622	CLDND1									
2623	CCSER1									
2624	MCTP2									
2625	SIGMAR1									
2626	PPP1CB									
2627	U2AF2									
2628	RAP1B									
2629	WDFY2									
2630	C11orf58									
2631	DHX34									
2632	MFSD5									
2633	TAF9B									
2634	RELCH									
2635	LCORL									
2636	DYM									
2637	TTLL12									
2638	PRKRA									
2639	ZNF140									
2640	GORAB									
2641	IFIT5									
2642	MED24									
2643	MKNK2									
2644	ZSCAN20									
2645	BIRC6									
2646	C2CD2L									
2647	KDM3A									
2648	KIAA2026									
2649	QARS1									
2650	MSH3									
2651	PRRC2C									
2652	FNIP2									

	A	B	C	D	E	F	G	H	I	J
2653	H1-4									
2654	UFSP1									
2655	DUSP19									
2656	PTAR1									
2657	ATR									
2658	PPP5C									
2659	FBXO3									
2660	PCDHB13									
2661	PNKP									
2662	ZNF674									
2663	MYBL2									
2664	ZC3H14									
2665	DOK4									
2666	TAF1C									
2667	ZNF138									
2668	FBXO33									
2669	ITSN2									
2670	ZNF236									
2671	SSH3									
2672	UBN2									
2673	OTUD5									
2674	PHF10									
2675	IPP									
2676	THAP5									
2677	TRAPPC8									
2678	OTUD4									
2679	NFKBIL1									
2680	RNF208									
2681	RBMS2									
2682	SEC23IP									
2683	LHFPL2									
2684	SLC16A10									
2685	STK17A									
2686	RNPC3									
2687	BBS4									
2688	TNRC6B									
2689	NR1D2									
2690	SIN3A									
2691	KLF16									
2692	BAZ2B									
2693	SCAF8									
2694	SRPK2									
2695	UBR5									
2696	IQGAP1									
2697	ADAM10									
2698	USP53									
2699	RBBP8									
2700	AHCY									
2701	MEIS1									
2702	KAT6B									
2703	DTX2									
2704	SUCO									
2705	OSMR									
2706	NKAPD1									
2707	CBX5									
2708	IPMK									
2709	RAB3GAP2									
2710	ZNF587B									
2711	ZNF506									
2712	DENND4A									
2713	SOS2									
2714	SAMD8									
2715	CENPT									
2716	PPP1R12A									
2717	ZFP90									
2718	LYST									
2719	NPEPPS									
2720	ARID4A									

	A	B	C	D	E	F	G	H	I	J
2721	FAM199X									
2722	ZNF383									
2723	ANO9									
2724	BAP1									
2725	ZER1									
2726	ZNF45									
2727	FBXW4									
2728	ZMYM1									
2729	GAREM1									
2730	EPN1									
2731	RIN1									
2732	IZUMO4									
2733	TMEM141									
2734	RPS6KA1									
2735	FGL2									
2736	DNAJC13									
2737	PPAT									
2738	RNF169									
2739	FAM8A1									
2740	DCTN3									
2741	GSTCD									
2742	SIAH1									
2743	THADA									
2744	TWSG1									
2745	CBLC									
2746	TLR2									
2747	BBIP1									
2748	SETD7									
2749	WRAP73									
2750	ECE2									
2751	PHF6									
2752	C7orf26									
2753	GPT									
2754	CCSER2									
2755	GGA1									
2756	RTKN									
2757	RAB2B									
2758	IRAK1									
2759	ZNF780A									
2760	SRSF9									
2761	NAA15									
2762	ZNF92									
2763	ZNF417									
2764	BMPR2									
2765	ARL1									
2766	KAT2B									
2767	BYSL									
2768	TRABD									
2769	PIN1									
2770	FKBP14									
2771	TMEM82									
2772	GDI1									
2773	NREP									
2774	CSK									
2775	TACC1									
2776	SMPD4									
2777	LIN7C									
2778	ITGB8									
2779	KPNA6									
2780	CALM3									
2781	ZNF235									
2782	PEX13									
2783	ZNF107									
2784	RSBN1L									
2785	UBE2G1									
2786	SEC23A									
2787	CWC22									
2788	PRPF19									

	A	B	C	D	E	F	G	H	I	J
2789	RAPGEF6									
2790	REST									
2791	CARD19									
2792	CENPB									
2793	GPA33									
2794	SIK2									
2795	DYRK1A									
2796	SLF1									
2797	OMD									
2798	WDR89									
2799	BROX									
2800	MFSD8									
2801	ARHGDIA									
2802	STAT4									
2803	GOLT1B									
2804	TMEM52									
2805	RBBP5									
2806	DCLRE1C									
2807	ZDHHC17									
2808	PITPNB									
2809	LRBA									
2810	SGPP1									
2811	C6orf226									
2812	ASH1L									
2813	CPSF2									
2814	LAMTOR4									
2815	RPL28									
2816	FAM107B									
2817	SLC30A4									
2818	CDIPT									
2819	IER2									
2820	CEP97									
2821	EVI5L									
2822	PKMYT1									
2823	REEP3									
2824	ARL14EP									
2825	TMEM150B									
2826	VPS50									
2827	ZNF585A									
2828	GNPTG									
2829	C19orf25									
2830	UBAC1									
2831	ANKRD13C									
2832	SEC22B									
2833	CCNO									
2834	ANKRD13B									
2835	ZCCHC8									
2836	PLSCR4									
2837	PDCL									
2838	ZNF776									
2839	SENP6									
2840	TRIM2									
2841	AMN1									
2842	THAP12									
2843	USP32									
2844	ALG1									
2845	RNMT									
2846	ARL6IP4									
2847	NABP2									
2848	ANKRD44									
2849	HSD11B1L									
2850	SLC12A6									
2851	KPNA1									
2852	RBPJ									
2853	LYSMD3									
2854	MED13L									
2855	QSER1									
2856	WDR75									

	A	B	C	D	E	F	G	H	I	J
2857	ENTPD4									
2858	BOD1L1									
2859	TMEM53									
2860	DOCK11									
2861	OTULIN									
2862	FAM98C									
2863	MTR									
2864	WWP1									
2865	TUBE1									
2866	HSF2									
2867	MARCHF1									
2868	ETV2									
2869	TIAM2									
2870	TARS3									
2871	JAZF1									
2872	MEIS2									
2873	LAMC1									
2874	MTPN									
2875	CCDC91									
2876	C1QTNF12									
2877	JAK2									
2878	DYNLT3									
2879	NIBAN1									
2880	TSR3									
2881	TNFSF13B									
2882	SEMA3B									
2883	UEVLD									
2884	GMIP									
2885	KIAA0895L									
2886	MAP3K2									
2887	ZNF414									
2888	ZFPL1									
2889	COPS2									
2890	HMOX2									
2891	G2E3									
2892	SF3B1									
2893	KANSL1L									
2894	RMND5B									
2895	USP15									
2896	SPRED1									
2897	CDKL5									
2898	ZNF230									
2899	DDX56									
2900	OGN									
2901	THAP11									
2902	PRPF4B									
2903	DENR									
2904	UBXN2A									
2905	CDK19									
2906	NOXA1									
2907	TSC22D4									
2908	PLIN3									
2909	RBM25									
2910	RAB33B									
2911	CCSAP									
2912	CASD1									
2913	C2CD5									
2914	RPS2									
2915	ASB7									
2916	LIX1L									
2917	FAM126A									
2918	MARCHF6									
2919	VCL									
2920	RIC8A									
2921	TEDC1									
2922	FAM13B									
2923	ITM2C									
2924	MTDH									

	A	B	C	D	E	F	G	H	I	J
2925	OGFOD3									
2926	HEG1									
2927	GATAD2A									
2928	MSANTD3									
2929	RHBDD3									
2930	EFHD2									
2931	ETV5									
2932	YPEL2									
2933	H1-3									
2934	BTBD3									
2935	EXTL2									
2936	RPS6KA5									
2937	CAMSAP3									
2938	SLC33A1									
2939	PPP2R5E									
2940	CAPN15									
2941	TBCK									
2942	RPL18A									
2943	CAMSAP2									
2944	PSMC4									
2945	SMARCD2									
2946	SLC39A9									
2947	UTRN									
2948	VPS26A									
2949	CDC27									
2950	MCFD2									
2951	CTBP1									
2952	PIP5K1A									
2953	CLEC5A									
2954	P2RX7									
2955	JMJD4									
2956	CREBRF									
2957	IRF7									
2958	RCOR3									
2959	TMF1									
2960	FRMD6									
2961	RFLNA									
2962	LRIG2									
2963	CSPP1									
2964	ZNF335									
2965	ITGA4									
2966	PRKAR1A									
2967	ANXA11									
2968	ALDH16A1									
2969	FBXO11									
2970	G6PD									
2971	SCAP									
2972	FYTTD1									
2973	WDR74									
2974	RPS6KB2									
2975	ZNF407									
2976	CEP162									
2977	NUDT14									
2978	RDX									
2979	DCAF16									
2980	MFAP3									
2981	MYO9A									
2982	SAMD1									
2983	SNTB2									
2984	IPO7									
2985	RBM10									
2986	TYK2									
2987	PBX3									
2988	CBWD2									
2989	LAGE3									
2990	PSD3									
2991	SCAND1									
2992	BIN1									

	A	B	C	D	E	F	G	H	I	J
2993	YIF1A									
2994	CUTA									
2995	GPATCH2									
2996	SMIM30									
2997	ZNF615									
2998	DNPEP									
2999	CPSF1									
3000	ABHD14B									
3001	DGKQ									
3002	MOB4									
3003	PRR7									
3004	RELB									
3005	PIGF									
3006	SERINC2									
3007	WDR11									
3008	ATAD5									
3009	ZNF358									
3010	SH3BP1									
3011	MAP3K20									
3012	ABI2									
3013	FXR1									
3014	ASCC2									
3015	TSSK6									
3016	IPO8									
3017	TMX4									
3018	TMEM250									
3019	IMPDH1									
3020	TINAGL1									
3021	C16orf72									
3022	C11orf86									
3023	SREK1IP1									
3024	MED13									
3025	C14orf28									
3026	CAND1									
3027	NCK1									
3028	SCAPER									
3029	FBRSL1									
3030	E2F4									
3031	EPS8L1									
3032	SHTN1									
3033	ASAP1									
3034	PI15									
3035	CLEC7A									
3036	ZNF555									
3037	ARHGEF38									
3038	NEK1									
3039	ANAPC4									
3040	SLC10A7									
3041	HPS6									
3042	CCDC18									
3043	PTPN14									
3044	ZNF524									
3045	JUP									
3046	TMEM87B									
3047	FLYWCH2									
3048	TTC5									
3049	RUSC1									
3050	ZNF765									
3051	FNDC3A									
3052	ESS2									
3053	ZNF143									
3054	UBE2S									
3055	UBC									
3056	TMCC1									
3057	COPS8									
3058	GGCX									
3059	YIF1B									
3060	CEP78									

	A	B	C	D	E	F	G	H	I	J
3061	DDX18									
3062	NCAPH2									
3063	TLR8									
3064	ARHGAP15									
3065	LMBRD2									
3066	ASPM									
3067	BMT2									
3068	PDGFC									
3069	DNAJC21									
3070	DMTF1									
3071	MPDZ									
3072	SSR3									
3073	PPP1R7									
3074	SLC36A4									
3075	TCF25									
3076	SRGAP1									
3077	DNM2									
3078	CNOT6L									
3079	CPD									
3080	CHUK									
3081	MCC									
3082	NSD3									
3083	DNAJB4									
3084	RNGTT									
3085	PQBP1									
3086	INTS11									
3087	CYRIA									
3088	CMTR2									
3089	AP1G2									
3090	TMEM63B									
3091	TNPO1									
3092	RCN2									
3093	ZFAND2B									
3094	TBC1D23									
3095	GRK2									
3096	RNF138									
3097	FCSK									
3098	NAF1									
3099	MAEA									
3100	KDF1									
3101	TRIR									
3102	MIB2									
3103	SEC24A									
3104	DNPH1									
3105	ZNF219									
3106	TVP23B									
3107	KDM7A									
3108	VPS37A									
3109	EFNB1									
3110	HRCT1									
3111	KLHL28									
3112	PIP4K2A									
3113	TRIM13									
3114	STXBP3									
3115	SYNJ1									
3116	RBBP8NL									
3117	MAML2									
3118	SLC35E3									
3119	RAB3GAP1									
3120	GLMN									
3121	MED25									
3122	CDC37L1									
3123	HCN3									
3124	CRB3									
3125	ARHGAP5									
3126	TMX1									
3127	BCL3									
3128	FAM174C									

	A	B	C	D	E	F	G	H	I	J
3129	PCM1									
3130	SCYL3									
3131	VIRMA									
3132	ALG6									
3133	TTC39B									
3134	ZNF441									
3135	STAP2									
3136	DAZAP1									
3137	SUV39H1									
3138	CPNE3									
3139	CCNK									
3140	USP13									
3141	MYNN									
3142	RABGGTA									
3143	TYW5									
3144	ZNF431									
3145	BTBD1									
3146	SLC66A1									
3147	SERTAD1									
3148	KAT2A									
3149	STX4									
3150	HOOK2									
3151	ZDHHC20									
3152	WDR7									
3153	MAN1A1									
3154	SENP2									
3155	CCDC9									
3156	C10orf88									
3157	ZNF652									
3158	MTLN									
3159	HSPB1									
3160	ANKRD26									
3161	EBP									
3162	HPS5									
3163	ARPC4									
3164	GMCL1									
3165	THOC2									
3166	ZNF551									
3167	OAF									
3168	TMC4									
3169	EBF1									
3170	LRRC40									
3171	MIF4GD									
3172	CCNT1									
3173	HIPK1									
3174	VAMP4									
3175	ABCA7									
3176	RSPRY1									
3177	RNF167									
3178	STAU2									
3179	SRM									
3180	ZNF397									
3181	CEP57									
3182	RPL13									
3183	CBL									
3184	PRR14L									
3185	DOCK4									
3186	CARF									
3187	HSF1									
3188	ZNF37A									
3189	ZNF653									
3190	EML3									
3191	ZNF428									
3192	SBNO1									
3193	SGTA									
3194	FYB1									
3195	PIK3CG									
3196	EMC10									

	A	B	C	D	E	F	G	H	I	J
3197	SHQ1									
3198	NT5DC1									
3199	PTK6									
3200	LDB2									
3201	DDX39A									
3202	INTS6									
3203	TLK2									
3204	SGF29									
3205	REPIN1									
3206	MOGS									
3207	CCM2									
3208	CDK17									
3209	ZYG11B									
3210	KLHL8									
3211	FAM76A									
3212	ELOVL1									
3213	SMIM13									
3214	NAA80									
3215	INSIG2									
3216	UBR1									
3217	GPR34									
3218	MIIP									
3219	PTPA									
3220	POLR2B									
3221	JUNB									
3222	SNRNP27									
3223	CALHM5									
3224	NPEPL1									
3225	SEPTIN11									
3226	SSH1									
3227	DENND4C									
3228	CDX1									
3229	DNAJB2									
3230	PEPD									
3231	MFSD1									
3232	ITPKC									
3233	CTDNEP1									
3234	DNAJC16									
3235	TMA16									
3236	RRP1									
3237	GTPBP2									
3238	USP45									
3239	AKT2									
3240	ZKSCAN8									
3241	SPAG9									
3242	CEP63									
3243	CCDC137									
3244	AP2A2									
3245	PPP1R12C									
3246	NEXN									
3247	PPP2R3B									
3248	TEX10									
3249	PDE4B									
3250	QPCTL									
3251	PLEKHM3									
3252	FAR1									
3253	FRMD4B									
3254	IPO11									
3255	TMEM37									
3256	USP14									
3257	DPYD									
3258	PCNX4									
3259	RASSF7									
3260	ZDHHC24									
3261	OPLAH									
3262	TRAPPC2L									
3263	GPR108									
3264	TAOK1									

	A	B	C	D	E	F	G	H	I	J
3265	SGMS2									
3266	GASK1B									
3267	ALMS1									
3268	RPS15									
3269	CAPRIN1									
3270	TRAPPC11									
3271	FBXO28									
3272	C4orf46									
3273	MED23									
3274	TEX264									
3275	SCYL1									
3276	AP4S1									
3277	PHGR1									
3278	FAM161A									
3279	BRAT1									
3280	SAMD4A									
3281	UPF2									
3282	ATL3									
3283	EVI5									
3284	ESCO1									
3285	FAM234A									
3286	CNOT7									
3287	APH1B									
3288	HOOK3									
3289	YES1									
3290	DOP1A									
3291	ACVR2A									
3292	BTF3L4									
3293	HM13									
3294	FCHSD2									
3295	GMPS									
3296	USP8									
3297	KLF9									
3298	LATS1									
3299	ENTPD2									
3300	PCNX3									
3301	AFTPH									
3302	SLC10A3									
3303	UBALD2									
3304	CEP83									
3305	LIN7B									
3306	PGM3									
3307	ZNF460									
3308	TRAF7									
3309	WWTR1									
3310	TDG									
3311	STK38L									
3312	LFNG									
3313	RASSF8									
3314	JKAMP									
3315	ZFYVE9									
3316	ZNF800									
3317	MIS12									
3318	SNRPC									
3319	OCEL1									
3320	TTC22									
3321	ZNF675									
3322	STX10									
3323	PLEKHG1									
3324	PPP4R3B									
3325	NCBP2AS2									
3326	ARHGAP12									
3327	ZNF768									
3328	HELQ									
3329	ARMC6									
3330	CDT1									
3331	IMP4									
3332	TUBB4B									

	A	B	C	D	E	F	G	H	I	J
3333	NUDT22									
3334	FRMD8									
3335	SENP5									
3336	CCDC121									
3337	UCHL5									
3338	SF3B5									
3339	C19orf47									
3340	AGAP3									
3341	SRP54									
3342	TMED8									
3343	DGCR2									
3344	MAGI3									
3345	RNH1									
3346	C9orf72									
3347	RPLP1									
3348	SLC12A7									
3349	PGM2									
3350	KPNA4									
3351	NSMF									
3352	SMARCE1									
3353	CDC42EP3									
3354	SOCS5									
3355	PCDH17									
3356	PCYT2									
3357	ZFAND1									
3358	SAMD10									
3359	PHKB									
3360	ST8SIA4									
3361	PLEKHJ1									
3362	SNRPB									
3363	LRRC8B									
3364	RAB40C									
3365	PHIP									
3366	KIF14									
3367	FMNL2									
3368	ZNF638									
3369	UCK1									
3370	HELB									
3371	WDR41									
3372	TIGD2									
3373	EDEM3									
3374	LGALS9									
3375	LAMTOR2									
3376	CDKAL1									
3377	KAT7									
3378	POT1									
3379	OPHN1									
3380	H3C1									
3381	HACD2									
3382	CLEC2D									
3383	EEF2									
3384	PIKFYVE									
3385	SLC52A2									
3386	TANC2									
3387	ARHGAP29									
3388	CDK9									
3389	IDUA									
3390	C1orf210									
3391	TRIP11									
3392	ELOB									
3393	TMEM33									
3394	LGALS3BP									
3395	ARFIP1									
3396	ATF6									
3397	ANKRD17									
3398	ALG10B									
3399	MAFK									
3400	MVB12A									

	A	B	C	D	E	F	G	H	I	J
3401	ATN1									
3402	COPE									
3403	PPFIBP1									
3404	CLMP									
3405	PTPN23									
3406	SLC38A2									
3407	NPAT									
3408	DPY19L3									
3409	USP20									
3410	PKP3									
3411	OSTM1									
3412	KIF3A									
3413	CERS6									
3414	CHMP2A									
3415	SGO2									
3416	ZNF282									
3417	ZNF33A									
3418	MBTD1									
3419	PCNX1									
3420	OTUD6B									
3421	GNG2									
3422	TTC26									
3423	SENP8									
3424	HMGXB4									
3425	ST7L									
3426	SKIV2L									
3427	CDC73									
3428	ZNF782									
3429	CSRNP2									
3430	PJA2									
3431	BAG4									
3432	PIK3R4									
3433	NUBP2									
3434	C2orf49									
3435	ZNRD2									
3436	MVD									
3437	NR1H2									
3438	USF2									
3439	MSI2									
3440	RPS16									
3441	EXOC8									
3442	C1GALT1									
3443	RNF2									
3444	RNF186									
3445	CSNK1D									
3446	ZNHIT1									
3447	RAB4B									
3448	ARRDC1									
3449	ZBED6									
3450	ARL13B									
3451	NUP58									
3452	SYNGR2									
3453	INTU									
3454	ATP6V0C									
3455	PTPN6									
3456	TBC1D32									
3457	CCDC6									
3458	NOP53									
3459	CEP135									
3460	NPDC1									
3461	PCSK4									
3462	SHPRH									
3463	PARP11									
3464	ARHGAP27									
3465	SPAST									
3466	ANKIB1									
3467	CFL1									
3468	TAPT1									

	A	B	C	D	E	F	G	H	I	J
3469	BRWD3									
3470	WDR46									
3471	STXBP2									
3472	NR2C2									
3473	SUV39H2									
3474	RB1CC1									
3475	RUVBL2									
3476	KATNB1									
3477	CAPZA1									
3478	ZHX1									
3479	GNAI3									
3480	CDX2									
3481	PHF3									
3482	TFEB									
3483	ARMCX5-GPRASP2									
3484	ADRM1									
3485	CREB5									
3486	NECTIN2									
3487	TEAD1									
3488	SNRNP70									
3489	ASPN									
3490	ZNF100									
3491	BBX									
3492	ZNF805									
3493	LRRC14									
3494	HERC3									
3495	KNTC1									
3496	PGAP1									
3497	BCL2L12									
3498	MLLT10									
3499	ACAP3									
3500	TRIM28									
3501	LSR									
3502	C5orf51									
3503	C16orf87									
3504	TMEM67									
3505	DAB2IP									
3506	TRAPPC6B									
3507	BBS10									
3508	PALMD									
3509	FOXN2									
3510	PIGB									
3511	B4GALT2									
3512	FAAP100									
3513	PPFIA3									
3514	FAM234B									
3515	SLC9A3R1									
3516	TRIM59									
3517	CTSD									
3518	SP3									
3519	SMAD2									
3520	ADAM9									
3521	GTDC1									
3522	SSX2IP									
3523	RGL1									
3524	PHLDA2									
3525	NFIA									
3526	KMT5B									
3527	DICER1									
3528	ABHD17A									
3529	BET1L									
3530	CHMP1A									
3531	EME2									
3532	LMNB2									
3533	CAPN5									
3534	YTHDC2									
3535	TTC21B									
3536	SUDS3									

	A	B	C	D	E	F	G	H	I	J
3537	ZBTB44									
3538	EIF2S1									
3539	NR1D1									
3540	GPS1									
3541	CLUAP1									
3542	PDAP1									
3543	HELZ2									
3544	PEX14									
3545	ZNF724									
3546	CCHCR1									
3547	CCNG2									
3548	PIAS1									
3549	C6orf62									
3550	NGEF									
3551	SETBP1									
3552	CRBN									
3553	ST6GALNAC5									
3554	UBE2E1									
3555	TRAF4									
3556	PITX1									
3557	SLC1A5									
3558	WDR44									
3559	ARL6									
3560	SLC44A4									
3561	TFPI									
3562	BRMS1									
3563	NPL									
3564	ZNF106									
3565	SUGP1									
3566	FGD6									
3567	ANKRD12									
3568	KCTD13									
3569	ZFC3H1									
3570	UBTD2									
3571	ZNF507									
3572	OSBPL7									
3573	CCDC88A									
3574	RPS19BP1									
3575	TRPS1									
3576	LIN52									
3577	CSNK1G2									
3578	DOT1L									
3579	CTDSP1									
3580	PIGK									
3581	SGMS1									
3582	MED27									
3583	ZNF668									
3584	NELFB									
3585	KIF22									
3586	HRAS									
3587	TRIM44									
3588	GPKOW									
3589	HERC1									
3590	PRMT1									
3591	HOOK1									
3592	RUFY3									
3593	RIF1									
3594	BRMS1L									
3595	MCRIP1									
3596	CHSY1									
3597	PLCD3									
3598	NAT9									
3599	STAT6									
3600	EIF2AK2									
3601	PWWP2B									
3602	CREM									
3603	TMC6									
3604	RSBN1									

	A	B	C	D	E	F	G	H	I	J
3605	NIPBL									
3606	H2BC18									
3607	ZEB2									
3608	HAGHL									
3609	EXOC3L1									
3610	ZNF669									
3611	FBXW5									
3612	XPO4									
3613	RPP25L									
3614	IKZF5									
3615	ZNF260									
3616	GOPC									
3617	OLR1									
3618	C11orf24									
3619	GNPDA2									
3620	BRCA2									
3621	APBA3									
3622	SNX18									
3623	SMIM14									
3624	SAR1A									
3625	KRTCAP2									
3626	EIF6									
3627	LRP3									
3628	TRUB1									
3629	SHOC2									

Supplementary Table 2

hTFtarget	KnockTF	JASPAR	PROMO	hTFtarget|I	hTFtarget|J	hTFtarget|I	KnockTF|J	KnockTF|P	JASPAR|PR	hTFtarget|I	hTFtarget|J	KnockTF|J	hTFtarget|KnockTF|JASPAR|PROMO
BRD3	ARID1A	TEAD3	abaA	EP300	SPI1	TBP	NR3C1	FOXM1	FOXO4	GATA2	MAZ	STAT3	YY1
BRD4	ZNF43	TFAP2B	ABF1	BMI1	CTCF		ELK1	FOXO1	FOXP3	MYB	POU2F1		FOXA1
CBFB	POLR3A	BHLHE23	ABI4		BCL11A		GATA3		HOXA4	MYC	STAT1		
CDK7	POSTN	BHLHE22	Adf-1		BHLHE40		JUN		HOXB7	RELA	USF1		
CDK9	RBM17	CLOCK	ADR1		E2F1		XBP1		HOXB8	SP1	AR		
HDAC1	RBFOX2	FIGLA	AGL3		EGR1		HIF1A		HOXD10	TP53	FOXA2		
MED1	ZNF622	HES5	AIRE		ELF1		ESRRA		POU6F1	E2F6	POU5F1		
POLR2A	RBM39	HES7	ALF1B		ERG		TFAP4		HOXD8	E2F7	SRF		
RAD21	BCLAF1	HEY1	Alfin1		ETS1		MYCN		HOXD9	TP63	VDR		
RUNX1T1	U2AF2	MNT	aMEF-2		FLI1		KLF7		FOXI1	MITF			
RXRA	TARDBP	OLIG1	AML1a		GLIS1		TCF7L1		SRY				
STAG1	EWSR1	OLIG3	ANF		IRF4		TEAD2		HOXA5				
TAF1	HNRNPLL	SREBF2	ANT		KLF1		ATF4		CDX2				
NOTCH1	AGO2	TFE3	Antp		MAX		RELB		HSF1				
BRCA1	DROSHA	ATF7	AP-1		NRF1		ZNF213		POU2F2				
ARID3A	HNRNPUL	CEBPE	AP-2		PAX5		E2F2		USF2				
GTF3C2	PSIP1	CEBPG	AP-2alpha		REST		FOXK2		DBP				
HCFC1	KDM3A	CREB3L1	AP-2alphaA		RUNX1		PLAG1		PKNOX1				
KDM5B	ZNF165	NFE2	AP-3		RUNX3		IRF2		POU1F1				
KMT2A	EZH2	NRL	AP3:PI		RXRG		ZNF574		POU3F1				
PHF8	NFKBIZ	TEF	AREB6		SPIB				POU3F2				
PRAME	ARID3B	FOXB1	ARP-1		TCF12				ATF3				
RBBP5	CREBBP	FOXC2	AT-BP1		TCF3				HOXA9				
UBTF	ZFP36L1	FOXD2	AT-BP2		ZNF143				HOXA3				
SMC3	HNRNPU	FOXO6	ATHB-1		RBPJ				FOXD3				
ARNT	GLI1	RXRB	ATHB-5		ESR1								
SIN3A	DDX5	TP73	B		ZBTB17								
GTF2B	DNMT1	GMEB2	BR-C		CEBPD								
MED12	ILF2	MTF1	BTEB3		CTCFL								
SMC1A	STRAP	E2F8	BTEB4		E2F4								
TAF3	USP22	SOX21	C/EBP		GATA1								
BCOR	MAEL	SOX4	C/EBPalpha		IRF1								
SAP30	USP7	SOX8	C/EBPbeta		JUND								
TAF7	PURA	TFEC	C/EBPbeta(p20)		MXI1								
EZH1	CBX2	HOXD12	C/EBPdelta		NFYA								
JMJD6	TXNIP	BARX1	C/EBPgamma		THAP1								
NCOR1	SMARCA1	BSX	C1		ZBTB7A								
RNF2	CIZ1	CDX1	C1-I		ZNF263								
RYBP	FUBP1	DLX6	CAC-binding		ZNF384								
TBL1X	MARK2	DUXA	CAR:RXR-alpha		GABPA								
TRIM28	NKRF	EMX2	Cart-1		FOS								
RCOR1	NUP35	EVX1	CAT8		NR2F2								
SUZ12	CNOT7	EVX2	CCBF		PGR								
LMNB1	NPM1	GBX1	CD28RC		TCF7L2								
SIX5	HDGF	GBX2	Cdx-1		TFAP2C								
WDR5	ABT1	GSC2	Cdx-3		ELK4								
KDM4C	CCAR1	GSX1	c-Ets-1		TFAP2A								
SMARCA4		GSX2	c-Ets-2		E2F3								
ARRB1		HESX1	c-Fos		ETS2								
CBX8		HMBOX1	CIZ6-1		ETV7								
MRE11A		HOXA10	c-Jun		HOXA1								
PPARD/PPARG		HOXA2	c-Myb		TFDP1								
		HOXB13	c-Myc		FOXH1								
		HOXB2	COE1		FOXP1								
		HOXB3	COE2		OTX2								
		HOXC10	CP2		TCF4								
		HOXC12	CPC1		TEAD4								
		HOXC13	CRE-BP2		ETV1								
		HOXD11	c-Rel		ZNF76								
		HOXD13	CREMalpha		CREB1								
		ISL2	CREMtau		MAFF								
		ASCL1	CREMtau1		MAFK								
		BACH2	CREMtau2		KLF9								
		FOXK1	CRF		KLF5								
		GATA6	Croc		SOX2								
		GRHL2	Crx		BCL6								
		NEUROD1	CTF		ELK3								
		NR1H4	CUTL1										
		NR4A1	Cutl1										
		PBX2	DEAF-1										
		PBX3	DEF:GLO										
		SIX1	DEF:GLO:SQUA										
		SIX2	DI										
		SOX13	DP-1										
		TWIST1	DPBF-1										
		ZNF24	DPBF-2										
		ZNF282	DRF1.1										
		ZSCAN4	DRF1.3										
		FOS::JUN	dri										
		FOSB::JUN	DSXF										
		FOSL1::JUN	DSXM										
		FOSL2::JUN	DTF-1										
		JUN::JUNB	E12										
		FOS::JUNB	E2										
		FOSB::JUN	E2F										
		FOSL1::JUN	E2F-1										
		FOSL2::JUN	E2F-1:DP-1										
		JUNB	E2F-5										
		FOS::JUND	E47										
		FOSL1::JUN	E4F1										
		FOSL2::JUN	E74A										
		NR1H4::RX	EBF										
		NR4A2::RX	EFII										
		PPARA::RX	Egr-1										
		RARA::RXR	EIIaE-A										
		RORB	Elf-1										
		RORC	Elk-1										
		SOX15	EmBP-1a										
		IRF3	En-1										
		IRF5	ENKTF-1										
		ARGFX	ER-alpha										
		ARNT2	ER-beta										
		ATF6	ERR1										

ATOH1	ETF
ATOH7	Eve
BARX2	f(alpha)-f(epsilon)
BHLHA15	FACB
CDX4	FOXA4a
CREB3L4	FOXF1
DLX5	FOXI1a
DMRTA2	FOXJ1
DMRTC2	FOXJ2
DPRX	FOXN2
DRGX	FOXO3a
ELF2	Ftz
FERD3L	FXR
FOXE1	GA-BF
FOXN3	GABP
GLI3	GABP-alpha
HES6	GAGA
HNF4A	GAL4
HOXA6	GAMYB
HOXA7	GATA-1
HOXB4	GATA-2
HOXB6	GATA-3
HOXB9	GBF
HOXC4	GCF
HOXC8	GCM
HOXD4	GCMa
IKZF1	GCMb
IRF6	GCN4
KLF10	GCR1
KLF11	GR
KLF15	GR-alpha
KLF17	GR-beta
KLF2	Gt
KLF3	GT-1
KLF6	GT-1b
LHX1	Hb
LHX5	HBP-1
MAF	HBP-1a(1)
MAFA	HBP-1a(c14)
MSANTD3	HELIOS
MSGN1	HES-1
NFATC4	HFH-1
NFIC	Hlf
NFIX	HMG
NHLH2	HNF-1
NKX6-3	HNF-1A
NR1D1	HNF-1B
NR1D2	HNF-1C
NR1I2	HNF-3
NR1I3	HNF-3gamma
NR2C1	HNF-4alpha
NR2C2	HNF-4alpha1
NR2F1	HNF-4alpha2
NR2F6	HNF-6
NR5A1	HNF-6beta
NR6A1	HOX11
OSR1	HTF
OVOL1	Ik-4
OVOL2	INSAF
PAX3	IPF1
PITX2	IRF-1
PLAGL2	IRF-2
PPARD	IRF-3
RARB	IRF-7A
RARG	Isl-1
RFX7	JunB
SMAD5	JunD
SNAI1	LCR-F1
SNAI3	LEF-1
SOHLH2	LF-A1
SOX12	Lhx3a
SOX14	LIM1
SOX18	LVb-binding
SP9	LVc
TBX18	LyF-1
TBX3	LYS14
TBX6	MAC1
TCF21	MafG
TFAP2E	mat1-Mc
TGIF2LX	MATa1
TGIF2LY	MATalpha1
THAP11	MATalpha2
THRB	MBF1
TLX2	MBP-1
VEZF1	MCB1
ZBTB26	MCB2
ZBTB32	mec-3
ZBTB6	MED8
ZFP57	MEDEA
ZIC5	MEF1
ZKSCAN1	MEF-2C/delta8
ZNF135	MEF-2DAB
ZNF136	Meis-1a
ZNF140	Meis-1b
ZNF274	MF3
ZNF317	MIF-1
ZNF382	MIG1
ZNF460	MNB1a
ZNF528	MRF-2
ZNF682	Msx-1
ZNF684	mtTFA
ZNF75D	muEBP-C2
ZSCAN29	MYB2
BARHL1	MYBAS1

HES1	MYBST1
HOXB5	Myf-3
HOXD3	Myf-5
DMRTA1	MyoD
HOXB1	myogenin
PAX8	MZF-1
ZIM3	Ncx
ZNF257	NF-1
ZNF343	NF-1/L
ZNF454	NF-AT1
ZNF524	NF-AT2
ZNF610	NF-AT3
ZNF675	NF-AT4
ZNF707	NFdeltaE3A
ZNF75A	NF-E4
ZNF766	NFI/CTF
ZNF770	NF-kappaB
ZNF784	NF-kappaB(-like)
ZNF8	NF-kappaB1
ZNF816	NF-muNR
ZNF85	NF-X3
ZNF93	NF-Y
ZSCAN16	NHP-1
ZSCAN31	NIT2
PRDM9	Nkx2-1
ATF2	Nkx2-5
BACH1	Nkx6-2
BATF	NRF-1
EBF3	Nrf2:MafK
HAND2	octamer-binding
MEIS1	Oct-B1
MEIS2	Opaque-2
MYF5	OSH15
NEUROG2	p300
NFIB	p53
NFYC	Pax-2
NKX2-2	Pax-2a
OSR2	Pax-4a
PRDM4	Pax-5
ZBTB12	Pax-6
ZBTB14	Pax-8
ZFP42	Pax-9a
ZKSCAN5	Pax-9b
ZNF148	PBF
ZNF16	Pbx1b
ZNF341	PDR1
ZNF449	PDR3
ZNF652	PEA3
FOXA3	PEBP2
ZNF189	PEBP2alphaA1
ZNF331	PEND
ZNF417	PHR1
ZNF549	POU1F1a
ZNF677	POU1F1b
ZNF680	POU1F1c
ZNF708	POU2F1a
ZNF768	POU2F1b
BATF3	POU2F1c
SREBF1	POU2F2B
BNC2	POU2F2C
ELK1::HOX	POU4F1(I)
ELK1::HOX	POU5F1(Oct-5)
ELK1::SREB	PPAR-alpha
ERF::FIGLA	PPAR-alpha:RXR-alpha
ERF::FOXI1	PR
ERF::FOXO	PR-alpha
ERF::HOXB	PR-beta
ERF::NHLH	Prd
ERF::SREBF	PTF1
ETV2::DRG	PTF1-beta
ETV2::FIGL	Pu
ETV2::FOX	PU.1
ETV2::HOX	PUR
ETV5::DRG	PXR-1:RXR-alpha
ETV5::FIGL	R
ETV5::FOX	R1
ETV5::FOX	R2
ETV5::HOX	RAR-beta
FLI1::DRGX	RAR-beta2
FLI1::FOXI1	RAR-gamma
FOXJ2::ELF	RC2
FOXO1::EL	RCS1
FOXO1::EL	RelA
FOXO1::EL	RF2a
FOXO1::FL	RITA-1
HOXB2::EL	ROM1
HOXD12::E	ROM2
MGA::EVX1	ROX1
PATZ1	RP58
POU2F1::S	S8
SATB1	SBF-1
SMAD2	SGF-3
SP5	Smad3
TFAP4::ETV	Smad4
TFAP4::FLI1	Sn
TFCP2	Sox13
THRA	Sox2
TRPS1	Sp1
ZBED2	Sp3
ZFP14	SPF1
ZKSCAN3	Spz1
ZNF211	Staf
ZNF214	STAT1beta
ZNF320	STAT4

ZNF324	STAT5A
ZNF354A	STAT5B
ZNF416	STAT6
ZNF418	STE12
ZNF530	SWI4
ZNF582	SWI6
ZNF667	SXR:RXR-alpha
ZNF669	T3R-alpha
ZNF692	T3R-alpha1
ZNF701	T3R-beta1
FOXP4	T3R-beta2
NKX2-4	TAF
DLX1	T-Ag
ZNF281	Tal-1
NFIL3	TCF-1(P)
FOXF2	TCF-1A
FOXD1	TCF-2
FOXC1	TCF-3
FOXL1	TCF-4
HLF	TCF-4E
NHLH1	TF68
MEF2A	TFIIB
MZF1	TFIID
MAX::MYC	TFII-I
NFKB1	TGA1a
PPARG::RX	TGGCA-binding
PPARG	TGIF
PAX6	TGT3
PBX1	Tll
RORA	TMF
RREB1	TRM1
RXRA::VDR	Ttk
SOX9	Ubx
TEAD1	UME6
TAL1::TCF	unc-86
REL	USF
NR1H2::RX	USF-1
NFIC::TLX1	USF2b
NKX3-1	VBP
ZNF354C	v-Myb
HINFP	Vpr
EWSR1-FL	VSF-1
NFE2L2	WIZZ
NFATC2	WRKY2
HNF1B	WT1
INSM1	XPF-1
FEV	Xvent-1
FOXO3	YAP1
RARA::RXR	Yi
NR4A2	Zen-1
ESR2	Zen-2
ARNT::HIF	ZF5
SOX10	Zic1
BATF::JUN	Zic2
CEBPB	Zic3
DUX4	Zta
FOSL1	
FOSL2	
HNF4G	
MEF2C	
MAF::NFE2	
NFYB	
PRDM1	
RFX5	
RUNX2	
SP2	
STAT1::STAT2	
ZBTB33	
CEBPA	
EBF1	
GATA1::TAL1	
ZEB1	
FOXP2	
EHF	
RFX2	
DMRT3	
EMX1	
FOXG1	
LBX1	
NFATC3	
SHOX	
ALX3	
BARHL2	
BHLHE41	
CREB3	
ELF3	
ELF4	
ELF5	
EN1	
EN2	
ESX1	
ETV6	
GCM1	
GRHL1	
GSC	
HEY2	
HNF1A	
HOXA13	
HOXC11	
IRF8	
IRF9	
ISX	
JDP2	
KLF13	

LHX6
MAFG
MEF2B
MEOX1
MIXL1
MLX
MLXIPL
MSC
MSX1
MYF6
NEUROD2
NFIA
NKX2-3
NKX2-8
NKX6-1
NKX6-2
OLIG2
ONECUT1
PAX4
PAX7
POU4F2
SP4
SPDEF
SPIC
TBXT
TBX2
TBX20
TBX21
TFEB
ZBTB7B
ZBTB7C
ZIC1
ZIC3
ZBTB18
LBX2
LHX2
LHX9
LMX1A
LMX1B
MEOX2
MNX1
MSX2
NKX3-2
NOTO
OTX1
PDX1
PHOX2A
PITX3
PROP1
PRRX1
RAX2
RAX
RHOXF1
UNCX
VAX1
VAX2
VENTX
VSX1
VSX2
ESRRB
NR3C2
RARA
BCL6B
EGR2
EGR3
EGR4
GLI2
GLIS2
GLIS3
HIC2
KLF14
KLF16
SCRT1
SCRT2
SNAI2
SP3
SP8
YY2
ZBED1
ZIC4
ZNF410
ZNF740
CUX1
CUX2
ONECUT2
ONECUT3
ERF
ETV2
ETV3
ETV4
ETV5
GATA5
GCM2
LEF1
HSF2
HSF4
IRF7
MEF2D
MEIS3
MYBL1
MYBL2
NFKB2
PAX1
PAX9

PKNOX2
POU3F3
POU3F4
POU4F1
POU4F3
POU5F1B
POU6F2
PROX1
SMAD3
TGIF1
TGIF2
RFX3
RFX4
EOMES
MGA
TBR1
TBX15
TBX19
TBX1
TBX4
TBX5
KLF4
GFI1
HES2
HOXC9
NEUROG1
PITX1
PRRX2
TCFL5
GATA4
MYOD1
MYOG
MAFG::NFE2L1
NKX2-5
PHOX2B
POU2F3
RFX1
TCF7
CREM
FOXS1
ZNF184
PAX2
KLF12
EPAS1
IKZF2
ZBED4
ZBTB11
ZBTB24
ZNF157
ZNF175
ZNF35
ZNF547
ZNF558
ZSCAN21
FEZF2

Supplementary Table 3

Protein	AAs	MW(Da)	calc.PI	Descriptior	gene	PG.Genes	oteinDescri	pedSeque	B_1	1_PG.Csco	1_PG.QVal	1_uniqCou	overage [%]
P98168	799	84769.8	7.53	Zinc finger	ZXDA	ZXDA;ZXD	Zinc finger	1	40.54856	41.3156	4.95E-29	1	1.75219
Q86XI8	627	70072.3	8.66	Uncharacte	ZSWIM9	ZSWIM9	Uncharacte	3	94.99374	44.72672	1.73E-38	3	6.539075
A7E2V4	1837	197295.5	6.8	Zinc finger	ZSWIM8	ZSWIM8	Zinc finger	1	266.2535	46.02852	1.56E-42	1	0.653239
Q8IWY8	852	96718.3	6.98	Zinc finger	ZSCAN29	ZSCAN29	Zinc finger	1	28.41171	34.67653	1.97E-15	1	0.938967
Q16670	478	55253.4	8.12	Zinc finger	ZSCAN26	ZSCAN26	Zinc finger	2	164.5461	48.93187	2.2E-52	1	3.76569
Q15695	479	57643	9.9	U2 small n	ZRSR2P1	ZRSR2P1	U2 small n	5	367.8091	53.2707	7.07E-69	1	12.31733
O95218	330	37404	10.67	Zinc finger	ZRANB2	ZRANB2	Zinc finger	12	8564.655	50.31916	1.82E-57	12	30.60606
O60232	199	21474.1	4.88	Protein ZN	ZNRD2	ZNRD2	Protein ZN	2	148.4348	36.52356	1.41E-18	2	14.57286
Q9NWK9	470	53918	5.53	Box C/D sr	ZNHIT6	ZNHIT6	Box C/D sr	1	74.63808	41.68535	5.64E-30	1	2.765957
Q15649	155	17607.1	5.36	Zinc finger	ZNHIT3	ZNHIT3	Zinc finger	2	192.515	49.52187	1.62E-54	2	12.25806
Q9UHR6	403	42883.1	5.86	Zinc finger	ZNHIT2	ZNHIT2	Zinc finger	2	30.26922	31.27914	6.42E-11	2	6.203474
Q8IUF1	395	44033.7	4.52	Zinc-regul	ZNG1B	ZNG1B;ZN	Zinc-regul	8	333.1733	50.09516	1.25E-56	2	25.31646
P0CJ78	1059	111076.2	9.45	Zinc finger	ZNF865	ZNF865	Zinc finger	7	103.7871	42.67796	1.31E-32	7	7.932011
Q96IR2	970	113132	9.76	Zinc finger	ZNF845	ZNF845	Zinc finger	12	380.7907	51.03092	3.73E-60	4	17.01031
Q0VGE8	651	75725	9.72	Zinc finger	ZNF816	ZNF816	Zinc finger	1	211.2208	49.89064	7.17E-56		1.382488
Q5CZA5	627	71142.9	7.72	Zinc finger	ZNF805	ZNF805	Zinc finger	1	127.9732	41.48058	1.89E-29	1	1.275917
Q2TB10	664	75235.2	10.07	Zinc finger	ZNF800	ZNF800	Zinc finger	11	796.8495	54.10332	3.14E-72	11	18.22289
Q3KP31	576	66871.2	9.59	Zinc finger	ZNF791	ZNF791	Zinc finger	1	274.4599	50.80557	2.7E-59	1	1.5625
Q6DD87	382	40428	8	Zinc finger	ZNF787	ZNF787	Zinc finger	2	136.844	52.9573	1.26E-67	2	5.759162
O75290	641	74530.8	8.75	Zinc finger	ZNF780A	ZNF780A	Zinc finger	4	291.0524	46.55088	3.18E-44	3	5.928237
Q9ULD5	831	93761.6	6.6	Zinc finger	ZNF777	ZNF777	Zinc finger	1	37.90925	20.69375	8.9E-06	1	1.925391
Q68DI1	518	59612.6	8.38	Zinc finger	ZNF776	ZNF776	Zinc finger	2	75.70987	45.9219	3.4E-42	2	4.440154
Q7L3S4	317	35701.8	8.88	Zinc finger	ZNF771	ZNF771	Zinc finger	1	106.8652	34.12009	1.4E-14	1	3.154574
Q6IQ21	691	80006.6	10.13	Zinc finger	ZNF770	ZNF770	Zinc finger	2	51.64497	38.6781	7.23E-23	2	3.762663
Q9H5H4	540	60228.3	8.01	Zinc finger	ZNF768	ZNF768	Zinc finger	11	935.8881	53.04708	5.53E-68	11	20.37037
Q8NDX6	193	21856.8	9.61	Zinc finger	ZNF740	ZNF740	Zinc finger	3	41.99305	43.52341	5.83E-35	3	27.97927
Q9Y462	761	86244.6	6.87	Zinc finger	ZNF711	ZNF711	Zinc finger	2	158.7814	51.24977	5.48E-61	2	4.073587
Q9UC06	446	50801.9	8.37	Zinc finger	ZNF70	ZNF70	Zinc finger	4	93.48239	49.46166	2.69E-54	4	11.65919
Q5TEC3	545	60461	7.29	Zinc finger	ZNF697	ZNF697	Zinc finger	2	109.5095	50.98328	5.68E-60	2	3.486239
Q5VV52	315	35809.5	7.84	Zinc finger	ZNF691	ZNF691	Zinc finger	3	54.35492	37.19675	7.6E-20	3	12.69841
Q96CS4	500	56906.3	10.28	Zinc finger	ZNF689	ZNF689	Zinc finger	2	44.7347	38.8014	3.92E-23	2	3.4
Q8N1G0	1237	129527.5	8.24	Zinc finger	ZNF687	ZNF687	Zinc finger	7	183.5002	43.03163	1.4E-33	7	7.194826
Q499Z4	452	50223.7	10.47	Zinc finger	ZNF672	ZNF672	Zinc finger	1	204.562	43.32983	2.06E-34	1	2.212389
Q96K58	619	67903.5	9.03	Zinc finger	ZNF668	ZNF668	Zinc finger	2	226.7863	49.40231	4.43E-54	2	3.55412
Q8N3J9	261	30283.6	8.79	Zinc finger	ZNF664	ZNF664	Zinc finger	1	56.92702	47.14924	3.31E-46	1	9.961686
Q6ZN08	573	65873.5	9.84	Putative zi	ZNF66	ZNF66	Putative zi	1	1725.923	48.44548	1.18E-50	1	1.570681
Q96CK0	615	67234.2	6.98	Zinc finger	ZNF653	ZNF653	Zinc finger	1	222.874	48.3089	3.55E-50	1	1.626016
Q9Y2D9	606	69743.8	8.8	Zinc finger	ZNF652	ZNF652	Zinc finger	3	87.43935	29.21688	5.7E-09	3	5.940594
O15015	1832	200823.6	7.21	Zinc finger	ZNF646	ZNF646	Zinc finger	3	60.21593	46.16695	5.61E-43	3	1.637555
Q9H582	1327	149564.2	8.25	Zinc finger	ZNF644	ZNF644	Zinc finger	6	198.007	52.12338	2.37E-64	6	6.028636
Q14966	1978	220622.9	6.31	Zinc finger	ZNF638	ZNF638	Zinc finger	34	1017.319	54.50719	7.3E-74	34	20.6269
Q9UEG4	869	96619.7	7.97	Zinc finger	ZNF629	ZNF629	Zinc finger	6	127.8528	49.8204	1.3E-55	6	8.860759
Q7L945	461	52852.6	9.13	Zinc finger	ZNF627	ZNF627	Zinc finger	1	114.1145	47.15253	3.23E-46	1	1.952278
Q969S3	477	54271.7	6.06	Cytoplasm	ZNF622	ZNF622	Cytoplasm	10	732.5491	52.27657	5.91E-65	10	26.83438
Q08AN1	781	90272.8	10.09	Zinc finger	ZNF616	ZNF616	Zinc finger	3	139.0872	47.54186	1.57E-47	3	3.713188
Q6ZNG1	722	83123.2	9.64	Zinc finger	ZNF600	ZNF600	Zinc finger	1	57.81722	29.54593	3.05E-09	1	1.800554
Q86UK7	904	98636.1	8.46	E3 ubiquiti	ZNF598	ZNF598	E3 ubiquiti	5	136.7691	50.03559	2.08E-56	5	7.632743
Q96LX8	424	48075.8	6.76	Zinc finger	ZNF597	ZNF597	Zinc finger	2	116.7146	31.21413	7.54E-11	2	5.188679
Q96JF6	807	93906.5	8.82	Zinc finger	ZNF594	ZNF594	Zinc finger	1	61.48576	54.107	3.04E-72	1	2.230483
O00488	134	15199	10.38	Zinc finger	ZNF593	ZNF593	Zinc finger	3	169.1125	49.65677	5.17E-55	3	32.08955
Q92610	1267	137527	7.88	Zinc finger	ZNF592	ZNF592	Zinc finger	3	200.8492	49.87554	8.16E-56	3	3.551697
Q8IVC4	421	48271.4	8.9	Zinc finger	ZNF584	ZNF584	Zinc finger	1	114.1303	42.00294	8.38E-31	1	2.137767
Q8NAF0	562	60508.6	8.76	Zinc finger	ZNF579	ZNF579	Zinc finger	3	102.8554	44.99927	2.57E-39	3	6.049822
Q68EA5	555	64427.3	8.88	Zinc finger	ZNF57	ZNF57	Zinc finger	3	343.7289	45.86677	5.09E-42	3	5.225225
Q8TBZ8	553	63734.9	8.77	Zinc finger	ZNF564	ZNF564	Zinc finger	1	240.8228	33.8525	3.46E-14	1	3.254973
Q8N988	423	48626.6	9.21	Zinc finger	ZNF557	ZNF557	Zinc finger	6	190.3855	44.88542	5.72E-39	6	16.54846
Q9H707	407	46198	8.59	Zinc finger	ZNF552	ZNF552	Zinc finger	4	102.6518	43.73718	1.43E-35	3	12.53071
Q6NX49	715	81741.2	7.88	Zinc finger	ZNF544	ZNF544	Zinc finger	1	70.47469	20.26626	1.04E-05	1	1.118881
Q8TF50	670	73621.2	6.46	Zinc finger	ZNF526	ZNF526	Zinc finger	5	224.3351	41.42122	2.68E-29	5	6.567164
Q96C55	264	28709.2	8.88	Zinc finger	ZNF524	ZNF524	Zinc finger	1	91.34785	51.72772	8.06E-63	1	3.787879
Q9C0D4	1074	119530.4	10.03	Zinc finger	ZNF518B	ZNF518B	Zinc finger	7	233.6355	51.56298	3.44E-62	7	8.286778
Q96ME7	567	64681.7	10.38	Zinc finger	ZNF512	ZNF512	Zinc finger	17	3145.56	52.85843	3.13E-67	17	40.03527
Q8TF39	744	85097.5	8.68	Zinc finger	ZNF483	ZNF483	Zinc finger	5	113.8957	39.09545	8.93E-24	5	6.451613
Q96MX3	618	67819.2	9.86	Zinc finger	ZNF48	ZNF48	Zinc finger	2	155.5204	53.878	2.62E-71	2	3.236246
Q96JM2	2506	284685.8	7.6	Zinc finger	ZNF462	ZNF462	Zinc finger	7	223.6607	45.53801	5.54E-41	7	2.992817
Q14592	562	63664.6	7	Zinc finger	ZNF460	ZNF460	Zinc finger	2	64.7476	45.1168	1.12E-39	2	4.270463
Q9Y4E5	1061	121483.5	6.76	E3 SUMO-	ZNF451	ZNF451	E3 SUMO-	8	323.449	51.47758	7.3E-62	8	8.482564
Q9NWS9	450	48956.7	6.03	Zinc finger	ZNF446	ZNF446	Zinc finger	1	208.8164	42.01741	7.68E-31	1	2.222222
P59923	1031	118962	9.88	Zinc finger	ZNF445	ZNF445	Zinc finger	3	162.5947	43.80019	9.39E-36	3	3.685742
Q9Y2A4	671	77515.7	9.38	Zinc finger	ZNF443	ZNF443	Zinc finger	5	212.0599	50.50217	3.8E-58		14.7541
Q9C0F3	470	54276.7	7.18	Zinc finger	ZNF436	ZNF436	Zinc finger	2	54.2765	45.96074	2.56E-42	2	4.680851
Q8TF32	576	67216.4	8.94	Zinc finger	ZNF431	ZNF431	Zinc finger	4	155.0478	46.68155	1.19E-44		8.854167
Q96B54	188	20480.4	3.84	Zinc finger	ZNF428	ZNF428	Zinc finger	3	1548.273	46.79667	4.95E-45	3	20.74468
Q8TAU3	575	65732.9	9.29	Zinc finger	ZNF417	ZNF417;ZN	Zinc finger	2	106.9143	34.72765	1.64E-15	2	3.478261
Q8NF99	534	61138.5	7.28	Zinc finger	ZNF397	ZNF397	Zinc finger	1	50.96628	32.80291	9.67E-13	1	2.996255
Q569K4	471	50406.4	10.61	Zinc finger	ZNF385B	ZNF385B	Zinc finger	5	219.7349	49.49111	2.1E-54	4	13.3758
Q96PM9	386	40453.3	10.52	Zinc finger	ZNF385A	ZNF385A	Zinc finger	1	35.89117	42.3649	9.18E-32	1	4.145078
Q8TF68	577	63218.7	9.19	Zinc finger	ZNF384	ZNF384	Zinc finger	2	84.24871	45.66176	2.26E-41	2	7.45234
O60765	605	69236.3	10.12	Zinc finger	ZNF354A	ZNF354A	Zinc finger	5	299.6127	47.91043	8.58E-49	5	8.264463
Q9UL40	294	32932.4	9.48	Zinc finger	ZNF346	ZNF346	Zinc finger	7	499.3878	46.67978	1.2E-44	7	30.61224
Q9Y3M9	751	86874.4	10.24	Zinc finger	ZNF337	ZNF337	Zinc finger	7	98.96514	43.98065	2.82E-36	7	10.253
Q9H4Z2	1342	144891.5	6.39	Zinc finger	ZNF335	ZNF335	Zinc finger	3	59.88375	41.8004	2.84E-30	3	2.757079
Q9Y3S2	320	36201	6.09	Zinc finger	ZNF330	ZNF330	Zinc finger	2	105.033	53.97058	1.11E-71	2	6.25
Q5BKZ1	582	65653.6	4.81	DBIRD con	ZNF326	ZNF326	DBIRD con	18	1791.02	57.07912	2.19E-84	18	37.97251
O75467	553	61103.5	10.05	Zinc finger	ZNF324	ZNF324	Zinc finger	2	137.2914	30.12138	9.41E-10	1	6.871609
A2RRD8	509	59326.1	9.1	Zinc finger	ZNF320	ZNF320	Zinc finger	1	64.66067	39.68515	4.2E-25	1	1.571709
P17041	273	31029	9.73	Zinc finger	ZNF32	ZNF32	Zinc finger	1	33.80934	30.9356	1.49E-10	1	2.930403
Q9P2F9	582	65546.4	8.41	Zinc finger	ZNF319	ZNF319	Zinc finger	3	104.3481	49.30898	9.69E-54	3	5.670103
Q96PQ6	595	67958.7	9.21	Zinc finger	ZNF317	ZNF317	Zinc finger	5	197.6311	42.77386	7.17E-33	4	8.235294
A6NFI3	1004	108436.3	5	Zinc finger	ZNF316	ZNF316	Zinc finger	5	229.4398	46.2369	3.34E-43	5	6.2749
O60281	2723	304813.5	7.41	Zinc finger	ZNF292	ZNF292	Zinc finger	4	115.6305	50.68238	7.88E-59	4	1.799486
Q9HBT8	521	60174.2	8.39	Zinc finger	ZNF286A	ZNF286A	Zinc finger	2	68.35095	31.04755	1.14E-10		3.838772

Q9Y2X9	895	96914.1	8.66	Zinc finger	ZNF281	ZNF281	Zinc finger	3	106.8277	44.63639	3.24E-38	3	3.240223
Q6N043	979	109284.1	8.26	Zinc finger	ZNF280D	ZNF280D	Zinc finger	5	65.12526	48.0995	1.9E-49	5	6.843718
Q8ND82	737	83095.3	9.52	Zinc finger	ZNF280C	ZNF280C	Zinc finger	16	906.1724	52.71981	1.09E-66	14	27.13704
P17035	718	83658	9.31	Zinc finger	ZNF28	ZNF28	Zinc finger	4	123.4914	44.74075	1.57E-38	1	5.710306
Q8N554	614	67218.4	8.6	Zinc finger	ZNF276	ZNF276	Zinc finger	1	15.56923	21.92107	5.56E-06	1	1.954397
Q9NSD4	429	48442.5	9.38	Zinc finger	ZNF275	ZNF275	Zinc finger	2	117.8493	42.71223	1.06E-32	2	3.962704
Q14586	743	87375.3	9.1	Zinc finger	ZNF267	ZNF267	Zinc finger	4	87.93212	48.21435	7.58E-50	3	5.921938
O43296	627	70586.3	7.51	Zinc finger	ZNF264	ZNF264	Zinc finger	3	139.5414	44.83724	8.01E-39	3	5.263158
P15622	560	63467.6	8.33	Zinc finger	ZNF250	ZNF250	Zinc finger	7	296.0161	49.25323	1.54E-53	7	13.21429
P17028	368	42155.1	6.17	Zinc finger	ZNF24	ZNF24	Zinc finger	3	215.4963	48.01998	3.59E-49	3	8.695652
Q86WZ6	799	92032.6	9.01	Zinc finger	ZNF227	ZNF227	Zinc finger	1	140.7351	31.53765	3.32E-11	1	1.126408
P17026	224	25915.3	10.72	Zinc finger	ZNF22	ZNF22	Zinc finger	4	277.0003	51.70136	1.02E-62	4	20.98214
O43670	478	50750.3	9.49	BUB3-inte	ZNF207	ZNF207	BUB3-inte	3	1221.91	46.36897	1.24E-43	3	6.903766
O95125	648	74719.9	5.88	Zinc finger	ZNF202	ZNF202	Zinc finger	1	29.3995	24.60015	1.44E-06	1	2.006173
O75820	626	72975.2	8.57	Zinc finger	ZNF189	ZNF189	Zinc finger	8	189.6576	53.01467	7.43E-68	8	13.41853
O15231	689	73524.7	7	Zinc finger	ZNF185	ZNF185	Zinc finger	9	91.77857	47.3318	8.09E-47	9	19.1582
Q99676	751	86173.8	8.12	Zinc finger	ZNF184	ZNF184	Zinc finger	8	234.8004	52.90479	2.04E-67	7	11.85087
P17025	639	73645.2	8.81	Zinc finger	ZNF182	ZNF182	Zinc finger	6	210.486	45.67141	2.11E-41	1	7.511737
Q15697	407	46454.5	10.18	Zinc finger	ZNF174	ZNF174	Zinc finger	1	64.41281	46.47172	5.76E-44	1	2.702703
Q9HCG1	818	94111.6	9.69	Zinc finger	ZNF160	ZNF160	Zinc finger	1	241.0807	49.73482	2.68E-55	1	1.222494
P17020	682	76471.1	8	Zinc finger	ZNF16	ZNF16	Zinc finger	3	223.0106	51.33519	2.57E-61	2	4.252199
Q9UQR1	794	88975.6	6.46	Zinc finger	ZNF148	ZNF148	Zinc finger	2	38.56948	38.46851	2.02E-22	2	3.904282
Q15072	292	33307.7	9.1	Zinc finger	ZNF146	ZNF146	Zinc finger	6	148.4414	52.74277	8.89E-67	6	20.54795
P52746	1687	187878.3	7.93	Zinc finger	ZNF142	ZNF142	Zinc finger	1	34.0182	43.24948	3.46E-34	1	0.770599
P17017	642	75352.9	9.51	Zinc finger	ZNF14	ZNF14	Zinc finger	9	457.8145	55.06348	3.56E-76	9	14.64174
P58317	390	44694.2	8.09	Zinc finger	ZNF121	ZNF121	Zinc finger	1	115.683	36.99839	1.83E-19	1	5.384615
P17014	697	81201.2	7.87	Zinc finger	ZNF12	ZNF12	Zinc finger	5	158.4573	44.8359	8.09E-39	5	7.173601
Q9ULU4	1186	131691.4	7.2	MYND-typ	ZMYND8	ZMYND8	MYND-typ	6	184.8543	50.66123	9.44E-59	6	7.082631
Q15326	602	70962.5	8.65	Zinc finger	ZMYND11	ZMYND11	Zinc finger	7	387.1407	53.25262	8.35E-69	7	13.62126
Q5VZL5	1548	172786.6	6.82	Zinc finger	ZMYM4	ZMYM4	Zinc finger	25	710.6443	51.37472	1.83E-61	24	20.93023
Q14202	1370	152377.7	6.28	Zinc finger	ZMYM3	ZMYM3	Zinc finger	21	621.0696	51.17091	1.09E-60	21	16.56934
Q5SVZ6	1142	128715.4	7.46	Zinc finger	ZMYM1	ZMYM1	Zinc finger	4	119.2633	52.78473	6.11E-67	4	4.640981
O75844	475	54812.4	7.58	CAAX prer	ZMPSTE24	ZMPSTE24	CAAX prer	2	25.57082	45.0834	1.42E-39	2	5.684211
Q9UDW3	170	19970.6	8.74	Zinc finger	ZMAT5	ZMAT5	Zinc finger	2	135.8121	52.63415	2.35E-66	2	12.94118
Q96NC0	199	23611.8	9.55	Zinc finger	ZMAT2	ZMAT2	Zinc finger	2	97.99364	35.43904	1.13E-16	2	10.55276
Q15776	578	65815.4	7.41	Zinc finger	ZKSCAN8	ZKSCAN8	Zinc finger	4	113.1269	37.0744	1.31E-19	4	9.16955
Q969J2	545	61578.9	7.64	Zinc finger	ZKSCAN4	ZKSCAN4	Zinc finger	1	18.5149	26.21565	3.87E-07	1	2.752294
P17029	563	63629.5	7.05	Zinc finger	ZKSCAN1	ZKSCAN1	Zinc finger	3	109.399	45.06975	1.56E-39	3	6.749556
O95409	532	55005.6	8.51	Zinc finger	ZIC2	ZIC2	Zinc finger	2	226.1849	45.85821	5.41E-42	1	3.571429
Q9UKY1	873	98096.8	5.91	Zinc finger	ZHX1	ZHX1	Zinc finger	7	221.0437	50.60047	1.61E-58	7	8.934708
Q8N5A5	531	57358.1	5.1	Zinc finger	ZGPAT	ZGPAT	Zinc finger	12	263.4472	52.65905	1.88E-66	12	29.75518
Q9BQ24	234	26505.4	8.49	Zinc finger	ZFYVE21	ZFYVE21	Zinc finger	1	19.24693	42.27261	1.62E-31	1	4.700855
Q7Z3T8	1539	168901.8	4.42	Zinc finger	ZFYVE16	ZFYVE16	Zinc finger	1	151.3688	47.04314	7.51E-46	1	1.039636
P17010	805	90521.3	6.09	Zinc finger	ZFX	ZFX	Zinc finger	8	448.4224	53.11492	2.97E-68	1	11.5528
Q96KR1	1074	117011.2	9.38	Zinc finger	ZFR	ZFR	Zinc finger	24	1397.565	52.23953	8.22E-65	24	29.70205
O95159	310	34113.5	8.13	Zinc finger	ZFPL1	ZFPL1	Zinc finger	2	142.7401	37.96811	2.22E-21	2	5.483871
Q96JP5	570	63444.5	7.38	E3 ubiquiti	ZFP91	ZFP91	E3 ubiquiti	9	632.7987	52.587	3.6E-66	8	15.26316
Q8TF47	636	73030.8	8.1	Zinc finger	ZFP90	ZFP90	Zinc finger	6	99.71099	49.48233	2.26E-54	6	11.00629
Q9NTW7	645	72216.3	8.48	Zinc finger	ZFP64	ZFP64	Zinc finger	1	80.95124	46.8442	3.45E-45	1	3.100775
Q8NB50	900	102510.5	9.32	Zinc finger	ZFP62	ZFP62	Zinc finger	13	168.8541	51.66828	1.36E-62	13	17.44444
Q07352	338	36313.1	8.03	mRNA dec	ZFP36L1	ZFP36L1	mRNA dec	1	49.66408	40.93626	4.4E-28	1	4.733728
Q9C0B5	715	77544	9.28	Palmitoyltr	ZDHHC5	ZDHHC5	Palmitoyltr	3	97.83769	49.02006	1.06E-52	3	5.734266
Q9NYG2	299	34170.2	8.24	Palmitoyltr	ZDHHC3	ZDHHC3	Palmitoyltr	1	111.0707	49.74049	1.06E-51	1	5.351171
Q8TBF4	217	24591.5	8.83	Zinc finger	ZCRB1	ZCRB1	Zinc finger	3	111.7627	50.48207	4.51E-58	3	15.6682
Q6NZY4	707	78576.8	4.51	Zinc finger	ZCCHC8	ZCCHC8	Zinc finger	12	433.7714	52.46629	1.06E-65	12	24.1867
Q8N3Z6	543	63051.9	7.35	Zinc finger	ZCCHC7	ZCCHC7	Zinc finger	3	23.27515	40.10177	4.53E-26	3	7.734807
Q9H5U6	513	59009.3	8.86	rRNA N6-a	ZCCHC4	ZCCHC4	rRNA N6-a	4	398.0875	52.24197	8.06E-65	4	8.382066
Q9NUD5	403	43547	8.64	Zinc finger	ZCCHC3	ZCCHC3	Zinc finger	3	56.31762	43.1784	5.46E-34	3	7.940447
Q8N2G6	241	26954.4	8.84	Zinc finger	ZCCHC24	ZCCHC24	Zinc finger	1	75.83457	45.60551	3.4E-41	1	4.149378
Q9C0B9	1178	125934.9	7.01	Zinc finger	ZCCHC2	ZCCHC2	Zinc finger	3	230.6278	48.72174	1.24E-51	3	2.886248
Q9NP64	241	27569.4	10.41	Zinc finger	ZCCHC17	ZCCHC17	Zinc finger	7	476.5887	50.72275	5.56E-59	7	32.36515
Q8TBK6	192	20966.4	8.84	Zinc finger	ZCCHC10	ZCCHC10	Zinc finger	2	1286.39	50.44284	6.31E-58	2	14.58333
Q86WB0	502	55260.9	5.29	Zinc finger	ZC3HC1	ZC3HC1	Zinc finger	6	294.2947	48.86633	3.78E-52	6	15.53785
Q7Z2W4	902	101430.5	8.47	Zinc finger	ZC3HAV1	ZC3HAV1	Zinc finger	34	5634.871	56.83654	1.82E-83	33	42.23947
Q8N5P1	291	33575.6	8.4	Zinc finger	ZC3H8	ZC3H8	Zinc finger	3	132.4592	46.36633	1.27E-43	3	9.621993
P61129	1189	131669	7.68	Zinc finger	ZC3H6	ZC3H6	Zinc finger	8	414.0274	48.13041	1.48E-49	8	7.989907
Q9UPT8	1303	140255.5	6.19	Zinc finger	ZC3H4	ZC3H4	Zinc finger	9	933.6104	47.24219	1.62E-46	9	11.20491
Q86VM9	953	106377.4	8.67	Zinc finger	ZC3H18	ZC3H18	Zinc finger	10	533.0258	51.18943	9.31E-61	10	13.22141
Q8WU90	426	48602.2	4.95	Zinc finger	ZC3H15	ZC3H15	Zinc finger	5	238.9602	51.08306	2.36E-60	5	14.78873
Q6PJT7	736	82875	7.34	Zinc finger	ZC3H14	ZC3H14	Zinc finger	7	132.7547	50.05225	1.81E-56	7	12.5
Q5T200	1668	196633.4	10.06	Zinc finger	ZC3H13	ZC3H13	Zinc finger	16	556.6678	55.70753	7.87E-79	16	10.49161
O75152	810	89130.4	8.56	Zinc finger	ZC3H11A	ZC3H11A	Zinc finger	12	285.9637	54.01449	7.28E-72	10	22.09877
Q96GY0	325	35091.8	10.48	Zinc finger	ZC2HC1A	ZC2HC1A	Zinc finger	4	207.225	51.05183	3.11E-60	4	12.92308
O15156	539	58026.4	5.72	Zinc finger	ZBTB7B	ZBTB7B	Zinc finger	1	170.2671	45.79327	8.7E-42	1	4.823748
O95365	584	61438.5	4.79	Zinc finger	ZBTB7A	ZBTB7A	Zinc finger	1	186.9729	45.50713	6.92E-41	1	1.883562
P10074	688	77053.4	8.22	Telomere z	ZBTB48	ZBTB48	Telomere z	4	80.27132	37.58754	1.3E-20	4	7.412791
Q5SVQ8	909	105191	7.98	Zinc finger	ZBTB41	ZBTB41	Zinc finger	11	339.5082	50.1799	6.04E-57	11	14.85149
Q9P1Z0	1013	105112.6	8.45	Zinc finger	ZBTB4	ZBTB4	Zinc finger	9	427.7145	51.92081	1.45E-63	8	12.14215
Q9ULJ3	1066	118869.3	8.38	Zinc finger	ZBTB21	ZBTB21	Zinc finger	1	72.58517	35.84923	2.26E-17	1	1.125704
Q9HC78	741	81082.8	6.48	Zinc finger	ZBTB20	ZBTB20	Zinc finger	5	248.3293	50.94918	7.66E-60	5	9.041835
O95625	1053	119382.9	8.82	Zinc finger	ZBTB11	ZBTB11	Zinc finger	13	354.9604	49.55802	1.2E-54	13	14.24501
Q96DT7	871	94893.4	4.79	Zinc finger	ZBTB10	ZBTB10	Zinc finger	5	254.2707	55.29492	3.8E-77	5	6.19977
O75132	1171	130320.7	6.82	Zinc finger	ZBED4	ZBED4	Zinc finger	1	81.64136	26.0459	4.56E-07	1	1.537148
O15391	372	41347.1	6.11	Transcripti	YY2	YY2	Transcripti	1	847.9553	36.14749	6.75E-18	1	4.032258
P25490	414	44712.4	6.22	Transcripti	YY1	YY1	Transcripti	7	1011.294	51.49733	6.1E-62	4	23.18841
P63104	245	27744.8	4.43	14-3-3 pro	YWHAZ	YWHAZ	14-3-3 pro	9	3161.8	49.16382	3.24E-53	9	38.77551
P27348	245	27763.9	4.41	14-3-3 pro	YWHAQ	YWHAQ	14-3-3 pro	12	1389.669	52.52434	6.33E-66	10	38.77551
Q04917	246	28218.4	4.47	14-3-3 pro	YWHAH	YWHAH	14-3-3 pro	7	638.0662	53.28675	6.13E-69	7	26.82927
P61981	247	28302.3	4.52	14-3-3 pro	YWHAG	YWHAG	14-3-3 pro	8	979.5109	50.47138	4.94E-58	5	31.98381
P62258	255	29173.6	4.36	14-3-3 pro	YWHAE	YWHAE	14-3-3 pro	9	558.1107	50.58232	1.89E-58	9	34.90196
P31946	246	28082.2	4.47	14-3-3 pro	YWHAB	YWHAB	14-3-3 pro	2	590.3799	49.56573	1.12E-54	2	9.349593
Q9Y5A9	579	62333.8	9.16	YTH doma	YTHDF2	YTHDF2	YTH doma	6	148.9958	50.89989	1.18E-59	6	11.9171
Q9BYJ9	559	60873.4	9.15	YTH doma	YTHDF1	YTHDF1	YTH doma	9	405.824	53.76242	7.84E-71	4	18.42576

Q9H6S0	1430	160246.9	8.52	3-5 RNA h	YTHDC2	YTHDC2	3'-5' RNA	45	1845.4	54.49954	7.8E-74	45	37.83217
Q96MU7	727	84699.7	6.13	YTH doma	YTHDC1	YTHDC1	YTH doma	15	2309.289	51.28049	4.19E-61	15	20.49519
Q86U90	279	29327.5	8.68	Threonylca	YRDC	YRDC	Threonylca	1	141.5177	46.07445	1.11E-42	1	4.659498
P62699	121	13841.5	7.39	Protein yip	YPEL5	YPEL5	Protein yip	3	241.7633	49.47728	2.36E-54	3	29.75207
Q96TA2	773	86454.5	9.2	ATP-deper	YME1L1	YME1L1	ATP-deper	2	237.7403	43.73879	1.41E-35	2	3.234153
P49750	2146	241643.6	6.51	YLP motif-	YLPM1	YLPM1	YLP motif-	50	1540.561	53.62228	2.91E-70	50	25.86207
O15498	198	22417.4	6.93	Synaptobr	YKT6	YKT6	Synaptobr	1	70.70751	24.92231	1.15E-06	1	8.585859
P13994	396	44801.9	8.38	Probable s	YJU2B	YJU2B	Probable s	1	59.3011	44.07227	1.52E-36	1	4.292929
Q9BW85	323	37085.5	5.65	Splicing fa	YJU2	YJU2	Splicing fa	1	65.62678	26.24512	3.76E-07	1	3.095975
Q96EC8	236	26255.6	5.28	Protein YIF	YIPF6	YIPF6	Protein YIF	2	74.35322	48.11528	1.67E-49	2	11.86441
Q969M3	257	27989	3.96	Protein YIF	YIPF5	YIPF5	Protein YIF	2	333.2438	51.27401	4.43E-61	2	8.171206
Q9BSR8	244	27082.4	4.25	Protein YIF	YIPF4	YIPF4	Protein YIF	2	96.25703	45.7862	9.16E-42	2	7.377049
Q5BJH7	314	34435.1	9.42	Protein YIF	YIF1B	YIF1B	Protein YIF	1	92.35187	28.88144	1.04E-08	1	7.961783
O95070	293	32010.9	9.19	Protein YIF	YIF1A	YIF1A	Protein YIF	2	28.22787	32.72196	1.23E-12	2	9.215017
P07947	543	60800.8	6.71	Tyrosine-p	YES1	YES1	Tyrosine-p	8	689.1329	51.53717	4.31E-62	4	18.60037
O95619	227	26499.3	8.81	YEATS dor	YEATS4	YEATS4	YEATS dor	2	87.5412	49.40979	4.16E-54	2	11.45374
P16989	372	40089.7	10.18	Y-box-bin	YBX3	YBX3	Y-box-bin	6	1301	47.60221	9.78E-48	2	22.84946
P67809	324	35924.1	10.31	Y-box-bin	YBX1	YBX1	Y-box-bin	3	1610.937	50.52901	3.01E-58	3	14.81481
P58557	167	19298	7.64	Endoribon	YBEY	YBEY	Endoribon	2	726.4099	46.76002	6.53E-45	2	16.16766
Q9Y2Z4	477	53198.6	9.34	Tyrosine--	YARS2	YARS2	Tyrosine--	6	233.2172	42.13612	3.73E-31	6	15.30398
P54577	528	59143	7.05	Tyrosine--	YARS1	YARS1	Tyrosine--	12	377.7046	56.62	1.23E-82	12	26.32576
Q9H0D6	950	108581.5	7.52	5-3 exorib	XRN2	XRN2	5'-3' exoril	30	4991.506	52.48841	8.71E-66	30	39.68421
Q8IZH2	1706	194106	7.22	5-3 exorib	XRN1	XRN1	5'-3' exoril	17	486.7299	51.66327	1.41E-62	17	11.19578
P12956	609	69842.4	6.61	X-ray repa	XRCC6	XRCC6	X-ray repa	32	7196.333	50.61635	1.41E-58	32	50.57471
P13010	732	82703.8	5.58	X-ray repa	XRCC5	XRCC5	X-ray repa	28	5723.655	53.10605	3.21E-68	28	36.06557
Q13426	336	38286.3	4.63	DNA repai	XRCC4	XRCC4	DNA repai	1	359.5933	44.95873	3.42E-39	1	2.380952
P18887	633	69496.9	6.19	DNA repai	XRCC1	XRCC1	DNA repai	22	8166.517	51.22154	7.01E-61	22	37.1248
Q9UBH6	696	81534.4	8.58	Solute carr	XPR1	XPR1	Solute carr	1	112.6836	33.49722	1.11E-13	1	1.58046
O43592	962	109963.2	5.05	Exportin-T	XPOT	XPOT	Exportin-T	2	185.4117	48.23609	6.38E-50	2	2.494802
Q9UIA9	1087	123906.1	6.27	Exportin-7	XPO7	XPO7	Exportin-7	2	89.40403	46.30434	2.01E-43	2	2.207912
Q9HAV4	1204	136309.9	5.55	Exportin-5	XPO5	XPO5	Exportin-5	7	99.3721	53.30196	5.34E-69	7	8.056478
Q9C0E2	1151	130138.1	4.69	Exportin-4	XPO4	XPO4	Exportin-4	3	48.55421	52.67728	1.6E-66	3	3.214596
O14980	1071	123385	5.98	Exportin-1	XPO1	XPO1	Exportin-1	18	274.4182	53.40157	2.14E-69	18	19.88796
Q9NQH7	507	57033.1	6.83	Xaa-Pro ar	XPNPEP3	XPNPEP3	Xaa-Pro ar	2	246.573	43.92247	4.16E-36	2	5.325444
Q9NQW7	623	69917.2	5.41	Xaa-Pro ar	XPNPEP1	XPNPEP1	Xaa-Pro ar	1	54.83009	38.76983	4.59E-23	1	1.444623
Q01831	940	105951.8	9.3	DNA repai	XPC	XPC	DNA repai	22	3153.808	51.70464	9.87E-63	22	27.23404
Q9HCS7	855	100008.7	6.13	Pre-mRNA	XAB2	XAB2	Pre-mRNA	21	543.2495	53.3181	4.61E-69	21	29.59064
O00308	870	98911.4	7.13	NEDD4-lik	WWP2	WWP2	NEDD4-lik	5	164.5462	54.36877	2.75E-73	5	6.436782
Q9H0M0	922	105201	5.71	NEDD4-lik	WWP1	WWP1	NEDD4-lik	7	266.5026	44.78913	1.12E-38	4	6.941432
Q15007	396	44243.4	4.84	Pre-mRNA	WTAP	WTAP	Pre-mRNA	8	533.1798	52.55131	4.98E-66	8	24.74747
Q96S55	665	72132.2	6.01	ATPase WI	WRNIP1	WRNIP1	ATPase WI	9	320.0595	57.48682	4.49E-86	9	16.54135
Q14191	1432	162459.4	6.29	Bifunctiona	WRN	WRN	Bifunctiona	4	146.1878	46.6208	1.88E-44	4	3.840782
Q9BUR4	548	59308.8	4.2	Telomeras	WRAP53	WRAP53	Telomeras	2	178.7505	44.24144	4.84E-37	2	4.927007
Q9UBV4	365	40689.6	8.67	Protein Wr	WNT16	WNT16	Protein Wr	1	64.48062	38.35124	3.57E-22	1	2.191781
Q5T9L3	541	62252.8	7.39	Protein wn	WLS	WLS	Protein wn	1	123.1799	39.74867	3.01E-25	1	1.2939
O95785	1651	178672.5	6.84	Protein Wi	WIZ	WIZ	Protein Wi	9	240.8062	54.42718	1.58E-73	9	5.814658
Q96FK6	387	43214.3	6.05	WD repeat	WDR89	WDR89	WD repeat	7	696.4772	53.55362	5.31E-70	7	23.25581
Q9BRX9	315	34342.4	5.25	WD repeat	WDR83	WDR83	WD repeat	3	82.26807	42.23765	2.01E-31	3	13.96825
Q6UXN9	313	35078.7	7.79	WD repeat	WDR82	WDR82	WD repeat	8	674.3453	52.11071	2.65E-64	8	27.15655
Q9BQA1	342	36724	4.81	Methyloso	WDR77	WDR77	Methyloso	7	954.6063	51.16238	1.18E-60	7	28.94737
Q9H967	626	69768.6	9.69	WD repeat	WDR76	WDR76	WD repeat	17	3316.868	54.66092	1.66E-74	17	30.35144
Q8IWA0	830	94497.9	5.81	WD repeat	WDR75	WDR75	WD repeat	7	225.5317	45.02667	2.12E-39	7	11.68675
Q6RFH5	385	42440.9	8.42	WD repeat	WDR74	WDR74	WD repeat	6	387.4401	51.57466	3.1E-62	6	17.92208
Q9NW82	654	73200.6	6.26	WD repeat	WDR70	WDR70	WD repeat	9	457.4023	51.78236	4.94E-63	9	16.36086
Q9Y4E6	1490	163808.6	6.91	WD repeat	WDR7	WDR7	WD repeat	1	37.53564	35.72883	3.65E-17	1	0.805369
Q9NNW5	1121	121723.6	6.87	tRNA (34-:	WDR6	WDR6	tRNA (34-:	20	951.243	54.29234	5.55E-73	20	21.32025
Q86VZ2	330	36337.7	7.67	WD repeat	WDR5B	WDR5B	WD repeat	1	1316.45	43.14374	6.82E-34	1	4.242424
Q9H6Y2	383	42069.6	4.52	WD repeat	WDR55	WDR55	WD repeat	10	694.6072	53.98231	9.94E-72	10	29.24282
P61964	334	36588.1	8.4	WD repeat	WDR5	WDR5	WD repeat	11	1530.44	51.14962	1.32E-60	10	47.90419
Q8TAF3	677	76209.9	7.04	WD repeat	WDR48	WDR48	WD repeat	14	388.6039	53.95566	1.28E-71	14	22.15657
O15213	610	68070.6	10.29	WD repeat	WDR46	WDR46	WD repeat	2	262.0752	51.5537	3.73E-62	2	4.098361
Q5JSH3	913	101365.4	5.1	WD repeat	WDR44	WDR44	WD repeat	10	204.5477	53.75983	8E-71	10	13.80066
Q15061	677	74890.1	5.28	WD repeat	WDR43	WDR43	WD repeat	5	624.4948	55.38135	1.77E-77	5	10.93058
Q8NI36	951	105321.1	7.57	WD repeat	WDR36	WDR36	WD repeat	14	550.0863	51.13444	1.51E-60	14	17.13985
Q9C0J8	1336	145890.9	9.71	pre-mRNA	WDR33	WDR33	pre-mRNA	13	805.7299	50.96227	6.84E-60	13	10.85329
Q9UNX4	943	106097.9	6.61	WD repeat	WDR3	WDR3	WD repeat	4	181.4333	49.04743	8.46E-53	4	4.241782
Q9H7D7	661	72123.4	6.1	WD repeat	WDR26	WDR26	WD repeat	12	2277.432	50.19453	5.32E-57	12	22.23903
Q64LD2	544	60160.6	9.25	WD repeat	WDR25	WDR25	WD repeat	3	66.98835	49.2438	1.67E-53	3	5.882353
Q8TBZ3	569	62892.6	8.08	WD repeat	WDR20	WDR20	WD repeat	6	158.345	47.65397	6.51E-48	6	12.12654
Q9BV38	432	47404.8	6.69	WD repeat	WDR18	WDR18	WD repeat	9	1029.34	52.76568	7.27E-67	9	27.77778
Q9GZL7	423	47707.5	5.74	Ribosome	WDR12	WDR12	Ribosome	9	1117.284	49.8107	1.41E-55	9	24.8227
Q9BZH6	1224	136683.5	6.91	WD repeat	WDR11	WDR11	WD repeat	27	1632.91	53.09599	3.53E-68	27	26.96078
O75083	606	66193.1	6.64	WD repeat	WDR1	WDR1	WD repeat	6	280.661	53.48222	1.01E-69	6	16.83168
Q8IWB7	410	46323.4	7.35	WD repeat	WDFY1	WDFY1	WD repeat	3	114.5388	47.83919	1.51E-48	3	9.02439
O75554	376	42506.6	5.25	WW doma	WBP4	WBP4	WW doma	3	176.2308	46.09262	9.7E-43	3	9.574468
Q969T9	261	28086.8	5.73	WW doma	WBP2	WBP2	WW doma	1	109.1732	43.45334	9.21E-35	1	3.831418
Q9Y2W2	641	69997	8.96	WW doma	WBP11	WBP11	WW doma	4	282.7235	48.21747	7.4E-50	4	6.708268
O00401	505	54826.5	8.05	Actin nucle	WASL	WASL	Actin nucle	1	375.2723	31.20353	7.74E-11	1	1.584158
Q9Y6W5	498	54283.5	5.16	Actin-binc	WASF2	WASF2	Actin-binc	2	31.8534	36.4932	1.6E-18	1	5.220884
P23381	471	53164.9	6.15	Tryptopha	WARS1	WARS1	Tryptopha	4	94.39346	48.22059	7.22E-50	4	8.917197
Q7Z5K2	1190	132944.6	5.09	Wings apa	WAPL	WAPL	Wings apa	2	86.62596	52.09293	3.11E-64	2	3.193277
A3KMH1	1905	214822.7	7.42	von Willeb	VWA8	VWA8	von Willeb	16	304.9324	52.57914	3.87E-66	16	9.76378
Q8N398	1242	131666.2	6.42	von Willeb	VWA5B2	VWA5B2	von Willeb	1	81.01797	30.60328	3.24E-10	1	0.966184
O00534	786	86488.5	6.55	von Willeb	VWA5A	VWA5A	von Willeb	2	165.7605	47.38435	5.38E-47	2	3.053435
P04004	478	54305.1	5.54	Vitronectir	VTN	VTN	Vitronectir	1	182.1348	51.31703	3.02E-61	1	3.138075
Q9UEU0	232	26688.2	9.54	Vesicle trai	VTI1B	VTI1B	Vesicle trai	1	34.36609	40.74383	1.31E-27	1	5.172414
Q9NP79	307	33878.9	6.24	Vacuolar p	VTA1	VTA1	Vacuolar p	3	338.3381	45.91826	3.49E-42	3	10.74919
Q8IV63	474	52880.8	9.39	Serine/thre	VRK3	VRK3	Serine/thre	5	640.6696	48.89501	2.98E-52	5	16.03376
Q86Y07	508	58140.1	9.16	Serine/thre	VRK2	VRK2	Serine/thre	5	154.729	46.81379	4.35E-45	5	12.99213
Q99986	396	45475.7	9.29	Serine/thre	VRK1	VRK1	Serine/thre	5	628.9294	45.08871	1.37E-39	5	15.90909
Q15906	364	40594	6.43	Vacuolar p	VPS72	VPS72	Vacuolar p	4	174.6805	38.70237	6.42E-23	4	10.98901
Q9UID3	782	86041.4	6.42	Vacuolar p	VPS51	VPS51	Vacuolar p	2	119.535	40.50535	4.95E-27	2	3.196931
O75351	444	49301.6	7.26	Vacuolar p	VPS4B	VPS4B	Vacuolar p	7	694.709	48.7301	1.16E-51	6	19.81982

Q9UN37	437	48897.5	7.93	Vacuolar p	VPS4A	VPS4A	Vacuolar p	3	86.10874	44.38681	1.8E-37	3	10.52632
Q9NRW7	570	65076.4	8.41	Vacuolar p	VPS45	VPS45	Vacuolar p	1	145.3727	41.58853	1E-29	1	2.105263
P49754	854	98565.5	5.68	Vacuolar p	VPS41	VPS41	Vacuolar p	3	130.2261	47.24396	1.6E-46	3	3.981265
Q96JC1	886	101808.2	6.99	Vam6/Vps	VPS39	VPS39	Vam6/Vps	2	56.08056	44.71248	1.91E-38	2	3.498871
A5D8V6	355	38658.6	4.89	Vacuolar p	VPS37C	VPS37C	Vacuolar p	1	153.8225	50.58463	1.85E-58	1	3.380282
Q9H9H4	285	31306.9	7.5	Vacuolar p	VPS37B	VPS37B	Vacuolar p	1	24.6554	16.87389	3.36E-05	1	3.859649
Q7Z3J2	963	109561.5	7.22	VPS35 end	VPS35L	VPS35L	VPS35 end	2	79.89967	46.21858	3.82E-43	2	2.180685
Q96QK1	796	91706.2	5.17	Vacuolar p	VPS35	VPS35	Vacuolar p	10	393.5156	46.18073	5.06E-43	10	14.19598
Q96AX1	596	67610.2	6.95	Vacuolar p	VPS33A	VPS33A	Vacuolar p	4	97.84274	50.38851	1.01E-57	4	6.711409
Q9UBQ0	182	20505.6	6.79	Vacuolar p	VPS29	VPS29	Vacuolar p	2	134.2322	41.08613	1.87E-28	2	12.63736
Q9UK41	221	25424.9	5.2	Vacuolar p	VPS28	VPS28	Vacuolar p	3	89.68858	52.45459	1.18E-65	3	17.64706
O14972	297	33010.2	7.76	Vacuolar p	VPS26C	VPS26C	Vacuolar p	1	25.42756	25.87471	5.34E-07	1	4.040404
O75436	327	38169.7	6.53	Vacuolar p	VPS26A	VPS26A	Vacuolar p	5	310.7921	49.24772	1.61E-53	4	17.43119
Q9BRG1	176	20747.6	6.28	Vacuolar p	VPS25	VPS25	Vacuolar p	1	26.1648	26.90818	1.83E-07	1	5.681818
Q9H269	839	94692.7	6.76	Vacuolar p	VPS16	VPS16	Vacuolar p	5	239.3741	51.71224	9.22E-63	5	6.555423
Q709C8	3753	422386.3	6.81	Intermemb	VPS13C	VPS13C	Intermemb	1	160.6673	32.32558	3.9E-12	1	0.213163
Q8N0U8	176	19835.4	9.35	Vitamin K	VKORC1L1	VKORC1L1	Vitamin K	1	70.93233	29.31631	4.74E-09	1	6.25
Q9BQB6	163	18234.3	9.58	Vitamin K	VKORC1	VKORC1	Vitamin K	1	215.3027	51.07868	2.45E-60	1	7.97546
Q69YN4	1812	202022.9	4.64	Protein viri	VIRMA	VIRMA	Protein viri	27	416.0857	51.09133	2.2E-60	27	17.38411
O15240	615	67257.1	4.45	Neurosecr	VGF	VGF	Neurosecr	2	154.6283	38.25338	5.73E-22	2	3.902439
Q14119	521	56930.9	10.12	Vascular e	VEZF1	VEZF1	Vascular e	3	70.13622	44.55964	5.5E-38	3	7.677543
Q9Y277	283	30658.5	8.9	Voltage-d	VDAC3	VDAC3	Voltage-d	9	2700.452	49.06282	7.45E-53	9	42.04947
P45880	294	31566.3	7.65	Voltage-d	VDAC2	VDAC2	Voltage-d	11	6417.454	50.18047	6.01E-57	11	44.89796
P21796	283	30772.4	8.89	Non-selec	VDAC1	VDAC1	Non-selec	11	4061.042	51.1672	1.13E-60	11	44.52297
P55072	806	89320.9	4.89	Transitiona	VCP	VCP	Transitiona	21	920.0779	56.07282	2.11E-80	21	35.23573
P18206	1134	123798.1	5.33	Vinculin	VCL	VCL	Vinculin	6	163.3266	55.36893	1.97E-77	6	7.848325
P61758	197	22625.7	7.19	Prefoldin s	VBP1	VBP1	Prefoldin s	1	192.3187	41.41164	2.83E-29	1	5.076142
P52735	878	101288.2	7.08	Guanine n	VAV2	VAV2	Guanine n	4	104.2039	45.94515	2.86E-42	4	6.378132
Q99536	393	41920	6.25	Synaptic v	VAT1	VAT1	Synaptic v	12	1208.708	54.32887	3.99E-73	12	49.10941
P50552	380	39829.5	9.41	Vasodilato	VASP	VASP	Vasodilato	1	224.343	48.27227	4.77E-50	1	2.631579
P26640	1264	140474.8	7.63	Valine--tR	VARS1	VARS1	Valine--tR	32	1253.117	51.16989	1.1E-60	32	28.79747
O95292	243	27228.2	7.39	Vesicle-as	VAPB	VAPB	Vesicle-as	4	165.276	50.89388	1.25E-59	4	22.22222
Q9P0L0	249	27893	8.93	Vesicle-as	VAPA	VAPA	Vesicle-as	7	2084.537	49.54217	1.37E-54	6	29.31727
Q8TAA9	524	59974.4	9.08	Vang-like	VANGL1	VANGL1	Vang-like	17	2358.771	51.74865	6.66E-63	17	38.74046
P51809	220	24934.8	8.79	Vesicle-as	VAMP7	VAMP7	Vesicle-as	2	77.23574	43.74502	1.35E-35	2	8.181818
P63027	116	12662.6	8.48	Vesicle-as	VAMP2	VAMP2;VA	Vesicle-as	1	89.91528	50.95849	7.06E-60	1	14.65517
Q08AM6	782	87971.7	6.03	Protein VA	VAC14	VAC14	Protein VA	2	80.22159	44.55461	5.69E-38	2	3.580563
Q9UBK9	157	18245.9	7.76	Protein UX	UXT	UXT	Protein UX	1	96.68323	34.88212	9.29E-16	1	7.006369
P46939	3433	394463.1	4.98	Utrophin	UTRN	UTRN	Utrophin	16	174.6593	50.33284	1.62E-57	16	5.388873
Q9NYH9	597	70193.2	7.31	U3 small n	UTP6	UTP6	U3 small n	4	65.9625	37.93328	2.61E-21	4	7.035176
Q969X6	686	76889.6	9.12	U3 small n	UTP4	UTP4	U3 small n	5	385.718	49.90245	6.5E-56	5	10.20408
Q9NQZ2	479	54557.5	5.28	Something	UTP3	UTP3	Something	7	468.4028	51.19823	8.61E-61	7	17.119
Q68CQ4	756	87054.4	5.72	U3 small n	UTP25	UTP25	U3 small n	24	982.0124	52.76181	7.54E-67	24	37.16931
Q9BRU9	249	28402.1	10.79	rRNA-proc	UTP23	UTP23	rRNA-proc	4	670.6111	51.02168	4.06E-60	4	18.4739
O75691	2785	318382.3	7.4	Small subu	UTP20	UTP20	Small subu	3	73.35945	44.56204	5.42E-38	3	1.149013
Q9Y5J1	556	62003.1	9.09	U3 small n	UTP18	UTP18	U3 small n	10	959.371	53.19799	1.36E-68	10	18.34532
Q8TED0	518	58414.7	9.52	U3 small n	UTP15	UTP15	U3 small n	5	163.803	49.46295	2.66E-54	5	11.96911
Q9BVJ6	771	87977.2	8.12	U3 small n	UTP14A	UTP14A	U3 small n	7	344.9068	47.16248	2.99E-46	6	11.93256
Q9Y3A2	253	30446.4	10.86	Probable U	UTP11	UTP11	Probable U	2	234.6902	36.02734	1.1E-17	2	7.509881
Q93008	2554	290460.1	5.59	Ubiquitin c	USP9X	USP9X	Ubiquitin c	15	204.5221	54.09726	3.32E-72	9	5.990603
P40818	1118	127522.2	8.75	Ubiquitin c	USP8	USP8	Ubiquitin c	3	166.176	52.28613	5.43E-65	3	3.041145
Q93009	1102	128301.3	5.24	Ubiquitin c	USP7	USP7	Ubiquitin c	34	1202.305	53.45253	1.33E-69	34	33.39383
P45974	858	95785.4	4.65	Ubiquitin c	USP5	USP5	Ubiquitin c	7	163.1172	50.36823	1.2E-57	7	12.23776
Q86UV5	1035	119031.4	5.92	Ubiquitin c	USP48	USP48	Ubiquitin c	3	175.0116	53.43392	1.59E-69	3	2.705314
P62068	366	42442.1	6.82	Ubiquitin c	USP46	USP46	Ubiquitin c	6	317.1976	45.14643	9.11E-40	4	16.93989
Q9H9J4	1324	145390.8	8.81	Ubiquitin c	USP42	USP42	Ubiquitin c	5	323.1104	47.24555	1.58E-46	5	5.513595
Q9NVE5	1235	140128.4	5.4	Ubiquitin c	USP40	USP40	Ubiquitin c	10	350.6016	44.53815	6.37E-38	10	10.04049
Q53GS9	565	65380	9.26	Ubiquitin c	USP39	USP39	Ubiquitin c	15	2253.181	50.29487	2.24E-57	15	34.15929
Q70CQ4	1352	146650	9.65	Ubiquitin c	USP31	USP31	Ubiquitin c	1	264.7945	43.63595	2.78E-35	1	0.961538
Q9Y6I4	520	58896.6	8.23	Ubiquitin c	USP3	USP3	Ubiquitin c	1	90.5192	31.44047	4.26E-11	1	2.884615
Q9Y5T5	823	93569.6	6.93	Ubiquitin c	USP16	USP16	Ubiquitin c	5	189.5374	52.89562	2.22E-67	5	7.533414
Q9Y4E8	981	112418.1	4.84	Ubiquitin c	USP15	USP15	Ubiquitin c	6	154.6772	45.57083	4.37E-41	6	8.868502
P54578	494	56068.7	4.94	Ubiquitin c	USP14	USP14	Ubiquitin c	3	277.4377	49.68856	3.96E-55	3	7.08502
O75317	370	42857.4	6.58	Ubiquitin c	USP12	USP12	Ubiquitin c	1	44.30563	31.67015	2.35E-11	1	3.513514
P51784	963	109815.8	5.14	Ubiquitin c	USP11	USP11	Ubiquitin c	10	389.9869	52.43298	1.45E-65	9	10.48806
Q14694	798	87133.1	4.96	Ubiquitin c	USP10	USP10	Ubiquitin c	9	745.1406	49.18607	2.7E-53	9	15.28822
O60763	962	107894.4	4.56	General ve	USO1	USO1	General ve	10	355.6808	54.67172	1.51E-74	10	13.30561
O14949	82	9906.3	10.5	Cytochrom	UQCRQ	UQCRQ	Cytochrom	2	190.1701	49.37599	5.52E-54	2	25.60976
P07919	91	10738.7	4.1	Cytochrom	UQCRH	UQCRH	Cytochrom	1	207.2619	48.00673	3.99E-49	1	19.78022
P47985	274	29667.7	8.46	Cytochrom	UQCRFS1	UQCRFS1	Cytochrom	4	281.7194	44.29226	3.42E-37	1	16.05839
P22695	453	48442.6	8.92	Cytochrom	UQCRC2	UQCRC2	Cytochrom	13	1016.84	51.36078	2.06E-61	13	35.54084
P31930	480	52645.3	6.32	Cytochrom	UQCRC1	UQCRC1	Cytochrom	7	423.8647	52.50233	7.7E-66	7	21.45833
P14927	111	13530.3	9.23	Cytochrom	UQCRB	UQCRB	Cytochrom	1	206.9976	44.53635	6.45E-38	1	9.009009
Q9BZI7	483	57761.6	10.14	Regulator	UPF3B	UPF3B	Regulator	3	152.0456	43.32288	2.15E-34	3	6.004141
Q9HAU5	1272	147808.8	5.42	Regulator	UPF2	UPF2	Regulator	2	50.61771	42.88796	3.49E-33	2	1.650943
Q92900	1129	124344.4	6.58	Regulator	UPF1	UPF1	Regulator	10	446.5219	49.28924	1.14E-53	10	12.04606
Q9C0B0	810	88083.7	6.85	RING finge	UNK	UNK	RING finge	1	81.20501	49.63459	6.24E-55	1	4.320988
P13051	313	34645.3	9.85	Uracil-DN	UNG	UNG	Uracil-DN	3	88.48937	50.80882	2.63E-59	3	12.77955
Q9H3U1	944	103075.9	5.9	Protein un	UNC45A	UNC45A	Protein un	19	618.8236	54.22379	1.02E-72	19	26.58898
A6NIH7	251	28136.3	5.41	Protein un	UNC119B	UNC119B;l	Protein un	1	172.2581	45.31233	2.8E-40	1	3.585657
P11172	480	52221.1	7.26	Uridine 5-	UMPS	UMPS	Uridine 5'-	4	171.5435	47.20479	2.16E-46	4	11.04167
Q9BZM4	244	27949	8.13	UL16-binc	ULBP3	ULBP3	UL16-binc	4	209.9006	46.6389	1.64E-44	2	12.70492
Q9BZM5	246	27367.6	7.34	UL16-binc	ULBP2	ULBP2	UL16-binc	5	395.0291	49.46021	2.72E-54	2	20.3252
Q9BZM6	244	27996.4	7.48	UL16-binc	ULBP1	ULBP1	UL16-binc	1	103.4945	47.83342	1.58E-48	1	4.098361
Q96PU4	802	89984.3	8.27	E3 ubiquiti	UHRF2	UHRF2	E3 ubiquiti	8	1157.2	53.02501	6.76E-68	7	12.46883
Q96T88	793	89813.2	7.59	E3 ubiquiti	UHRF1	UHRF1	E3 ubiquiti	22	1872.167	51.3454	2.36E-61	22	34.67844
Q8TAS1	419	46545.8	5.77	Serine/thre	UHMK1	UHMK1	Serine/thre	1	46.49421	40.30943	1.46E-26	1	2.625298
Q16851	508	56939.8	8.4	UTP--gluc	UGP2	UGP2	UTP--gluc	2	155.5289	34.9641	6.86E-16	2	4.133858
Q9NYU1	1516	174733.7	6.88	UDP-gluco	UGGT2	UGGT2	UDP-gluco	8	176.2028	42.28461	1.51E-31	8	5.079156
Q9NYU2	1555	177188.1	5.32	UDP-gluco	UGGT1	UGGT1	UDP-gluco	18	333.6769	52.72461	1.05E-66	17	13.63344
O60701	494	55023.5	7.13	UDP-gluco	UGDH	UGDH	UDP-gluco	6	172.5497	48.48594	8.48E-51	6	14.37247
Q9NUQ7	469	53260.9	7.01	Ufm1-spec	UFSP2	UFSP2	Ufm1-spec	5	251.7434	45.06534	1.61E-39	5	13.64606
P61960	85	9117.5	9.91	Ubiquitin-	UFM1	UFM1	Ubiquitin-	1	81.00099	43.23854	3.71E-34	1	17.64706

O94874	794	89594.4	6.77	E3 UFM1-	UFL1	UFL1	E3 UFM1-	5	219.8752	50.98159	5.76E-60	5	7.052897
Q92890	307	34500.1	6.68	Ubiquitin r	UFD1	UFD1	Ubiquitin r	2	178.3066	46.78763	5.3E-45	2	8.143322
Q9BZX2	261	29298.9	6.68	Uridine-cy	UCK2	UCK2	Uridine-cy	2	106.6252	46.41742	8.65E-44	2	14.94253
Q9Y5K5	329	37606.6	5	Ubiquitin c	UCHL5	UCHL5	Ubiquitin c	5	337.8665	55.391	1.64E-77	5	19.14894
O94888	489	54861.9	4.82	UBX doma	UBXN7	UBXN7	UBX doma	2	141.9169	41.90715	1.5E-30	2	4.498978
Q92575	508	56776.9	6.26	UBX doma	UBXN4	UBXN4	UBX doma	1	90.67017	43.34097	1.92E-34	1	2.755906
Q04323	297	33325	4.93	UBX doma	UBXN1	UBXN1	UBX doma	1	110.6885	26.02526	4.65E-07	1	2.356902
P17480	764	89405.3	5.52	Nucleolar t	UBTF	UBTF	Nucleolar t	25	4168.225	50.60494	1.55E-58	25	36.51832
Q8WUN7	234	26189.3	5.4	Ubiquitin c	UBTD2	UBTD2;UBT	Ubiquitin c	2	79.88158	49.90612	6.29E-56	2	10.25641
O95071	2799	309349.2	5.63	E3 ubiquiti	UBR5	UBR5	E3 ubiquiti	5	460.2929	39.31884	2.84E-24	5	2.500893
Q5T4S7	5183	573835.3	5.91	E3 ubiquiti	UBR4	UBR4	E3 ubiquiti	13	184.5511	47.62172	8.39E-48	13	2.913371
Q8IWV8	1755	200536.4	6.19	E3 ubiquiti	UBR2	UBR2	E3 ubiquiti	8	208.598	48.78771	7.21E-52	8	5.584046
Q8IWV7	1749	200208.8	5.91	E3 ubiquiti	UBR1	UBR1	E3 ubiquiti	4	164.3492	52.69574	1.36E-66	4	2.801601
Q9NRR5	601	63852.5	4.88	Ubiquilin-4	UBQLN4	UBQLN4;U	Ubiquilin-4	1	397.6447	41.85447	2.05E-30	1	2.66223
Q8WVY7	318	36804.4	6.39	Ubiquitin-	UBLCP1	UBLCP1	Ubiquitin-	4	220.1207	55.85919	1.77E-79	4	12.89308
P11441	157	17776.3	9.12	Ubiquitin-	UBL4A	UBL4A	Ubiquitin-	2	144.9775	43.20337	4.65E-34	2	12.73885
Q14139	1066	122559.3	4.87	Ubiquitin c	UBE4A	UBE4A	Ubiquitin c	3	107.1618	46.3051	2E-43	3	3.283302
Q15386	1083	123921.3	6.69	Ubiquitin-	UBE3C	UBE3C	Ubiquitin-	9	173.8138	50.86597	1.6E-59	9	8.217913
Q13404	147	16494.9	8.18	Ubiquitin-	UBE2V1	UBE2V1	Ubiquitin-	2	304.2928	52.05339	4.42E-64	1	12.92517
Q16763	222	23845.1	8.67	Ubiquitin-	UBE2S	UBE2S	Ubiquitin-	2	98.33211	52.60428	3.09E-66	2	11.71171
Q712K3	238	27165.7	4.02	Ubiquitin-	UBE2R2	UBE2R2	Ubiquitin-	2	191.947	43.43429	1.04E-34	2	9.243697
Q9C0C9	1292	141292.3	4.76	(E3-indepe	UBE2O	UBE2O	(E3-indepe	12	271.3002	46.61512	1.96E-44	12	11.30031
P61088	152	17137.6	6.53	Ubiquitin-	UBE2N	UBE2N	Ubiquitin-	6	291.2243	50.01378	2.5E-56	1	30.26316
P61081	183	20899.8	7.84	NEDD8-co	UBE2M	UBE2M	NEDD8-co	5	2927.835	48.19988	8.51E-50	5	30.60109
P68036	154	17861.4	8.79	Ubiquitin-	UBE2L3	UBE2L3	Ubiquitin-	5	307.662	46.63344	1.71E-44	2	47.4026
P61086	200	22406.4	5.1	Ubiquitin-	UBE2K	UBE2K	Ubiquitin-	2	150.8124	50.27625	2.64E-57	2	11.5
P63279	158	18006.7	8.9	SUMO-cor	UBE2I	UBE2I	SUMO-cor	3	578.7014	45.8827	4.53E-42	3	14.55696
P62256	183	20655	4.29	Ubiquitin-	UBE2H	UBE2H	Ubiquitin-	3	134.4225	45.63473	2.75E-41	3	21.85792
P51965	193	21403.9	8.71	Ubiquitin-	UBE2E1	UBE2E1;UE	Ubiquitin-	2	194.7356	39.57225	7.61E-25	2	9.84456
P61077	147	16687	7.95	Ubiquitin-	UBE2D3	UBE2D3;U	Ubiquitin-	1	1409.774	51.50219	5.85E-62	1	7.482993
P49459	152	17315.3	4.79	Ubiquitin-	UBE2A	UBE2A	Ubiquitin-	1	160.5798	44.49901	8.33E-38	1	11.18421
Q8TF42	649	72695.3	6.92	Ubiquitin-	UBASH3B	UBASH3B	Ubiquitin-	11	460.9275	51.88709	1.95E-63	11	19.56857
Q5T6F2	1119	117114.6	7.36	Ubiquitin-	UBAP2	UBAP2	Ubiquitin-	1	48.56137	43.53888	5.27E-35	1	1.340483
A0AVT1	1052	117968.9	6.05	Ubiquitin-	UBA6	UBA6	Ubiquitin-	4	100.6321	49.13181	4.22E-53	4	5.608365
P62987	128	14728.2	10.51	Ubiquitin-	UBA52	UBA52	Ubiquitin-	1	16.00887	34.02667	1.92E-14	1	7.8125
Q8TBC4	463	51851.9	5.12	NEDD8-ac	UBA3	UBA3	NEDD8-ac	4	97.62851	48.13853	1.39E-49	4	9.719222
Q9UBT2	640	71222.9	4.91	SUMO-act	UBA2	UBA2	SUMO-act	3	90.69593	48.83307	4.96E-52	3	6.09375
P22314	1058	117848.1	5.5	Ubiquitin-	UBA1	UBA1	Ubiquitin-	14	705.6102	54.29919	5.23E-73	14	17.48582
Q16222	522	58768.7	6.29	UDP-N-ac	UAP1	UAP1;UAP	UDP-N-ac	1	178.332	38.66124	7.86E-23	1	2.490421
Q9BZF9	1416	162504.2	7.02	Uveal auto	UACA	UACA	Uveal auto	15	266.7888	48.40004	1.7E-50	15	12.14689
O15042	1029	118290.8	8.82	U2 snRNP-	U2SURP	U2SURP	U2 snRNP-	27	3892.979	52.63905	2.24E-66	27	29.34888
P26368	475	53500.6	9.49	Splicing fa	U2AF2	U2AF2	Splicing fa	11	12880.93	46.68892	1.12E-44	11	34.10526
Q01081	240	27871.9	8.95	Splicing fa	U2AF1	U2AF1	Splicing fa	8	8293.082	50.14906	7.85E-57	3	24.16667
Q6IPR3	259	29793.9	7.77	tRNA wybu	TYW3	TYW3	tRNA wybu	1	39.21003	34.98532	6.35E-16	1	6.949807
Q16881	649	70905.6	7.42	Thioredoxi	TXNRD1	TXNRD1	Thioredoxi	2	46.82051	46.60187	2.17E-44	2	5.084746
O43396	289	32251.1	4.58	Thioredoxi	TXNL1	TXNL1	Thioredoxi	3	142.2105	52.24267	8.02E-65	3	12.11073
Q8NBS9	432	47628.5	5.86	Thioredoxi	TXNDC5	TXNDC5	Thioredoxi	9	522.4475	47.39328	5.02E-47	9	24.76852
Q9BRA2	123	13940.7	5.22	Thioredoxi	TXNDC17	TXNDC17	Thioredoxi	1	51.29064	31.01075	1.24E-10	1	11.38211
P10599	105	11737.4	4.55	Thioredoxi	TXN	TXN	Thioredoxi	3	2096.987	47.09911	4.88E-46	3	31.42857
Q96RR1	684	77153.5	9.34	Twinkle mt	TWNK	TWNK	Twinkle mt	10	764.672	50.27343	2.7E-57	10	18.12865
Q6IBS0	349	39547.7	6.85	Twinfilin-2	TWF2	TWF2	Twinfilin-2	3	287.006	41.87847	1.78E-30	3	15.75931
Q12792	350	40282.4	6.96	Twinfilin-1	TWF1	TWF1	Twinfilin-1	1	85.72672	37.54023	1.61E-20	1	3.714286
Q5VYS8	1495	171227.8	6.81	Terminal u	TUT7	TUT7	Terminal u	24	720.6896	52.22918	9.59E-65	22	18.92977
P49411	455	49874.6	7.68	Elongation	TUFM	TUFM	Elongation	20	10833.96	53.23075	1.02E-68	20	49.67033
Q9BSJ2	902	102533	6.84	Gamma-tu	TUBGCP2	TUBGCP2	Gamma-tu	1	244.5637	40.92088	4.8E-28	1	1.330377
P23258	451	51169.5	6.06	Tubulin ga	TUBG1	TUBG1	Tubulin ga	4	267.2735	48.76607	8.62E-52	1	9.534368
Q3ZCM7	444	49775.7	4.52	Tubulin be	TUBB8	TUBB8	Tubulin be	1	204.5987	39.9869	8.42E-26	1	7.432432
Q9BUF5	446	49856.8	4.51	Tubulin be	TUBB6	TUBB6	Tubulin be	10	2097.659	50.69279	7.2E-59	5	31.39013
P68371	445	49830.7	4.52	Tubulin be	TUBB4B	TUBB4B	Tubulin be	21	13986.02	51.43427	1.07E-61	1	57.52809
P04350	444	49585.5	4.51	Tubulin be	TUBB4A	TUBB4A	Tubulin be	2	95.69154	56.40554	9.37E-82	2	6.756757
Q13509	450	50432.4	4.57	Tubulin be	TUBB3	TUBB3	Tubulin be	4	255.5938	50.05633	1.74E-56	4	18.66667
Q9BVA1	445	49952.8	4.52	Tubulin be	TUBB2B	TUBB2B	Tubulin be	1	130.3789	27.40175	9.94E-08	1	2.696629
Q13885	445	49906.7	4.52	Tubulin be	TUBB2A	TUBB2A	Tubulin be	5	6763.463	47.73247	3.51E-48	1	15.2809
P07437	444	49670.5	4.52	Tubulin be	TUBB	TUBB	Tubulin be	3	4494.067	46.04928	1.34E-42	3	12.16216
P68366	448	49924	4.69	Tubulin alp	TUBA4A	TUBA4A	Tubulin alp	3	651.5988	49.89742	6.78E-56	3	7.589286
P0DPH7	450	49959.1	4.74	Tubulin alp	TUBA3C	TUBA3C;TU	Tubulin alp	2	809.4442	44.94336	3.81E-39		6
Q9BQE3	449	49894.9	4.73	Tubulin alp	TUBA1C	TUBA1C	Tubulin alp	1	214.2455	50.48148	4.53E-58	1	3.786192
P68363	451	50151.2	4.7	Tubulin alp	TUBA1B	TUBA1B	Tubulin alp	15	6003.777	50.28058	2.54E-57		37.25055
Q9C0H2	523	57544.1	5.05	Protein tw	TTYH3	TTYH3	Protein tw	1	121.2072	47.13854	3.6E-46	1	2.676864
P02766	147	15886.9	5.58	Transthyre	TTR	TTR	Transthyre	2	189.0018	50.77568	3.5E-59	2	24.4898
Q14166	644	74403	5.25	Tubulin--t	TTLL12	TTLL12	Tubulin--t	5	191.9426	43.22159	4.14E-34	5	10.09317
O43156	1089	122068.2	5.85	TELO2-int	TTI1	TTI1	TELO2-int	2	108.9973	52.28141	5.66E-65	2	3.030303
Q15361	905	103050.3	10	Transcripti	TTF1	TTF1	Transcripti	7	99.82981	41.54079	1.33E-29	7	8.39779
Q8N5M4	171	20012.5	9.18	Tetratricop	TTC9C	TTC9C	Tetratricop	1	88.02686	35.25424	2.31E-16	1	8.77193
Q8N584	583	65869.1	6.98	Tetratricop	TTC39C	TTC39C	Tetratricop	1	31.36319	17.48039	2.75E-05	1	1.543739
Q6P3X3	843	96631.5	5.29	Tetratricop	TTC27	TTC27	Tetratricop	3	68.70241	26.7142	2.29E-07	3	4.270463
Q6DKK2	380	42456.2	5.53	Tetratricop	TTC19	TTC19	Tetratricop	1	72.08645	31.18166	8.17E-11	1	3.157895
Q96N46	770	88317.8	8.93	Tetratricop	TTC14	TTC14	Tetratricop	3	145.9197	48.10718	1.78E-49	3	4.675325
Q8NBP0	860	96812.2	7.01	Tetratricop	TTC13	TTC13	Tetratricop	1	83.85078	44.23351	5.11E-37	1	1.744186
Q99614	292	33525.8	4.49	Tetratricop	TTC1	TTC1	Tetratricop	1	168.2544	53.89702	2.21E-71	1	3.424658
Q16762	297	33428.7	7.28	Thiosulfate	TST	TST	Thiosulfate	1	53.59856	43.44196	9.92E-35	1	4.040404
Q9Y5U2	329	34325.2	4.85	U5 small n	TSSC4	TSSC4	U5 small n	2	94.0455	42.80051	6.06E-33	2	6.382979
Q9UJK0	312	33595.5	6.87	18S rRNA a	TSR3	TSR3	18S rRNA a	1	317.6741	42.57472	2.5E-32	1	3.525641
Q2NL82	804	91809.5	7.44	Pre-rRNA-	TSR1	TSR1	Pre-rRNA-	22	4345.089	51.79965	4.26E-63	22	35.44776
Q9H2G4	693	79434.4	4.19	Testis-spe	TSPYL2	TSPYL2	Testis-spe	2	487.0703	50.6329	1.21E-58	2	2.886003
Q9H0U9	437	49192	5.15	Testis-spe	TSPYL1	TSPYL1	Testis-spe	6	491.8614	48.92904	2.25E-52	6	18.30664
P30536	169	18827.8	9.58	Translocat	TSPO	TSPO	Translocat	2	83.56732	45.20075	6.19E-40	2	18.3432
O95858	294	33164.6	5.1	Tetraspani	TSPAN15	TSPAN15	Tetraspani	1	78.40691	49.30508	1E-53	1	4.081633
Q15631	228	26182.7	6.41	Translin	TSN	TSN	Translin	1	91.82475	40.86821	6.47E-28	1	5.701754
Q99816	390	43943.9	6.39	Tumor sus	TSG101	TSG101	Tumor sus	5	296.7959	54.60818	2.78E-74	5	15.38462
P43897	325	35390.4	8.56	Elongation	TSFM	TSFM	Elongation	1	206.4629	45.41361	1.36E-40	1	5.230769
Q7Z6J9	526	58818.8	8	tRNA-splic	TSEN54	TSEN54	tRNA-splic	2	181.9806	45.14338	9.3E-40	2	5.13308
Q9BSV6	310	33651.6	8.69	tRNA-splic	TSEN34	TSEN34	tRNA-splic	3	267.319	48.56966	4.3E-51	3	11.6129

Q5SRN2	563	61625.6	9.89	Testis-exp	TSBP1	TSBP1	Testis-exp	1	34.15624	30.63695	3E-10	1	1.243339
O95900	331	36694.2	9.36	Pseudouri	TRUB2	TRUB2	Pseudouri	3	109.3595	50.54416	2.64E-58	3	11.17825
Q8WWH5	349	37252.4	8.45	Pseudouri	TRUB1	TRUB1	Pseudouri	2	170.5492	41.86288	1.95E-30	2	6.590258
Q86TN4	253	27741.7	10.65	tRNA 2-pl	TRPT1	TRPT1	tRNA 2'-pl	5	138.8893	51.89404	1.84E-63	5	30.83004
Q8TD43	1214	134299.6	8.2	Transient r	TRPM4	TRPM4	Transient r	6	378.4027	52.21861	9.87E-65	6	5.68369
Q96Q11	434	50127.1	8.3	CCA tRNA	TRNT1	TRNT1	CCA tRNA	1	60.97267	47.67794	5.39E-48	1	2.304147
O75648	421	47744.3	8.12	Mitochonc	TRMU	TRMU	Mitochonc	1	32.05286	43.07975	1.03E-33	1	3.800475
Q9BVS5	477	52964.9	6.61	tRNA (ade	TRMT61B	TRMT61B	tRNA (ade	2	110.451	48.42065	1.44E-50	2	6.708595
Q8IZ69	625	68725	7.99	tRNA (urac	TRMT2A	TRMT2A	tRNA (urac	3	114.6085	43.61277	3.24E-35	3	6.08
Q7Z2T5	733	81746.6	7.94	TRMT1-lik	TRMT1L	TRMT1L	TRMT1-lik	3	50.48874	42.52081	3.49E-32	3	5.3206
Q9UI30	125	14199.3	4.98	Multifuncti	TRMT112	TRMT112	Multifuncti	2	188.4998	47.08355	5.5E-46	2	21.6
Q7Z4G4	463	53420.6	7.88	tRNA (gua	TRMT11	TRMT11	tRNA (gua	3	92.05885	45.08128	1.44E-39	3	9.50324
Q7L0Y3	403	47346.6	9.93	tRNA meth	TRMT10C	TRMT10C	tRNA meth	16	1600.596	53.45852	1.26E-69	16	45.16129
Q8TBZ6	339	39718.5	7.74	tRNA meth	TRMT10A	TRMT10A	tRNA meth	2	86.31272	46.3577	1.35E-43	2	7.374631
Q9NXH9	659	72233.3	7.67	tRNA (gua	TRMT1	TRMT1	tRNA (gua	5	134.7535	44.49502	8.55E-38	5	9.408194
Q9BQ61	176	18419.3	10.12	Telomeras	TRIR	TRIR	Telomeras	1	143.3117	38.43448	2.39E-22	1	6.818182
Q15650	581	66145.8	7.93	Activating	TRIP4	TRIP4	Activating	19	1701.632	52.02038	5.95E-64	19	32.87435
Q14669	1992	220431.5	8.64	E3 ubiquiti	TRIP12	TRIP12	E3 ubiquiti	33	825.09	54.85202	2.65E-75	33	20.03012
Q15642	601	68351.7	5.45	Cdc42-inte	TRIP10	TRIP10	Cdc42-inte	2	116.2543	41.69664	5.27E-30	2	3.826955
Q9H2D6	2365	261372.9	8.55	TRIO and F	TRIOBP	TRIOBP	TRIO and F	10	467.5419	47.87076	1.18E-48	10	4.312896
O75962	3097	346897.2	6.32	Triple func	TRIO	TRIO	Triple func	5	90.41171	49.02823	9.9E-53	5	1.808201
Q6ZMU5	477	52730.1	6.45	Tripartite r	TRIM72	TRIM72	Tripartite r	17	1742.849	51.44334	9.89E-62	17	49.47589
Q9BRZ2	755	81486.9	7.78	E3 ubiquiti	TRIM56	TRIM56	E3 ubiquiti	13	730.9998	51.22972	6.53E-61	13	23.44371
Q96LD4	638	69531.3	6.4	E3 ubiquiti	TRIM47	TRIM47	E3 ubiquiti	5	326.036	38.31309	4.3E-22	5	9.404389
Q8WV44	630	71669.4	4.72	E3 ubiquiti	TRIM41	TRIM41	E3 ubiquiti	12	559.6284	51.12721	1.61E-60	12	23.1746
Q9C037	500	57460.5	8.18	E3 ubiquiti	TRIM4	TRIM4	E3 ubiquiti	4	130.5782	48.18715	9.41E-50	4	10.6
O00635	465	53415.5	7.02	E3 ubiquiti	TRIM38	TRIM38	E3 ubiquiti	2	90.61906	47.96989	5.35E-49	2	4.516129
Q13049	653	71987.9	6.98	E3 ubiquiti	TRIM32	TRIM32	E3 ubiquiti	6	207.5072	43.75771	1.25E-35	6	9.954058
O75382	744	80829	7.87	Tripartite r	TRIM3	TRIM3	Tripartite r	6	231.7664	47.39654	4.89E-47	5	9.811828
Q13263	835	88548.8	5.55	Transcripti	TRIM28	TRIM28	Transcripti	16	2218.578	52.65281	1.99E-66	16	22.99401
P14373	513	58489.2	6.17	Zinc finger	TRIM27	TRIM27	Zinc finger	5	355.1788	56.72437	4.86E-83	5	11.50097
Q12899	539	62165.4	4.69	Tripartite r	TRIM26	TRIM26	Tripartite r	21	2216.794	52.65038	2.03E-66	21	41.18738
Q14258	630	70972.8	8.15	E3 ubiquiti	TRIM25	TRIM25	E3 ubiquiti	15	730.6516	51.14464	1.38E-60	15	29.36508
P36406	574	64066.1	6.35	E3 ubiquiti	TRIM23	TRIM23	E3 ubiquiti	1	203.3276	36.68729	7.02E-19	1	1.74216
P19474	475	54169.3	6.35	E3 ubiquiti	TRIM21	TRIM21	E3 ubiquiti	21	3636.17	54.7153	9.98E-75	21	50.73684
Q9NSU2	314	33211.8	8.01	Three-prin	TREX1	TREX1	Three-prin	2	80.18236	46.4126	8.96E-44	2	10.50955
Q96Q05	1148	128528.7	6.6	Trafficking	TRAPPC9	TRAPPC9	Trafficking	2	68.85355	29.91399	1.45E-09	2	2.351916
O43617	180	20273.9	4.58	Trafficking	TRAPPC3	TRAPPC3	Trafficking	3	125.0254	42.24501	1.92E-31	3	17.77778
Q9Y5R8	145	16831.4	9.47	Trafficking	TRAPPC1	TRAPPC1	Trafficking	1	161.6172	46.0985	9.3E-43	1	6.896552
Q12931	704	80109.2	8.4	Heat shock	TRAP1	TRAP1	Heat shock	19	2363.309	52.08042	3.46E-64	19	32.67045
Q15629	374	43071.2	10.09	Translocati	TRAM1	TRAM1	Translocati	1	123.2401	41.56727	1.13E-29	1	2.941176
Q6Q0C0	670	74608.5	7.15	E3 ubiquiti	TRAF7	TRAF7	E3 ubiquiti	3	74.40556	40.58037	3.26E-27	3	4.477612
Q9BUZ4	470	53542.2	8.19	TNF recept	TRAF4	TRAF4	TNF recept	5	119.3211	52.23413	8.6E-65	5	15.10638
Q13114	568	64489.6	7.96	TNF recept	TRAF3	TRAF3	TNF recept	6	296.3822	49.82951	1.21E-55	6	14.26056
Q12933	501	55858.8	7.56	TNF recept	TRAF2	TRAF2	TNF recept	4	111.5157	50.75543	4.18E-59	4	9.580838
Q15628	312	34246.6	6.17	Tumor nec	TRADD	TRADD	Tumor nec	2	61.21133	47.61196	9.06E-48	2	10.89744
Q9H4I3	376	42320.8	8.13	TraB doma	TRABD	TRABD	TraB doma	3	141.953	46.89488	2.34E-45	3	9.840426
P62995	288	33665.3	11.73	Transform	TRA2B	TRA2B	Transform	8	3367.865	49.37454	5.59E-54	8	31.25
Q13595	282	32688.3	11.75	Transform	TRA2A	TRA2A	Transform	8	3244.957	48.94035	2.05E-52	8	25.88652
Q9ULW0	747	85652.5	9.88	Targeting	TPX2	TPX2	Targeting	17	600.9911	49.07275	6.86E-53	17	27.0415
P13693	172	19595.2	4.57	Translation	TPT1	TPT1	Translation	1	185.7282	47.02042	8.94E-46	1	7.55814
P12270	2363	267291.1	4.68	Nucleopro	TPR	TPR	Nucleopro	8	157.9416	47.20005	2.24E-46	8	4.358866
P29144	1249	138348.7	6.27	Tripeptidyl	TPP2	TPP2	Tripeptidyl	7	131.1258	51.56437	3.4E-62	7	7.285829
O14773	563	61247.5	6.46	Tripeptidyl	TPP1	TPP1	Tripeptidyl	1	22.21414	17.53355	2.7E-05	1	2.486679
P67936	248	28521.5	4.36	Tropomyo	TPM4	TPM4	Tropomyo	6	273.4001	51.44437	9.81E-62	6	23.79032
P06753	285	32949.6	4.38	Tropomyo	TPM3	TPM3	Tropomyo	5	2944.572	48.53787	5.56E-51	5	16.14035
P07951	284	32850.4	4.36	Tropomyo	TPM2	TPM2	Tropomyo	7	3509.465	48.60394	3.25E-51	1	21.47887
P09493	284	32708.2	4.39	Tropomyo	TPM1	TPM1	Tropomyo	3	228.6723	49.40302	4.4E-54	3	9.859155
P60174	249	26669.3	6.91	Triosephos	TPI1	TPI1	Triosephos	8	248.0804	48.91079	2.61E-52	8	42.16867
O43399	206	22237.5	5.01	Tumor pro	TPD52L2	TPD52L2	Tumor pro	6	282.1275	54.0435	5.52E-72	6	37.86408
P55327	224	24326.8	4.49	Tumor pro	TPD52	TPD52	Tumor pro	2	132.0448	38.74536	5.19E-23	2	12.94643
Q8IXH6	220	23979.6	6.78	Tumor pro	TP53INP2	TP53INP2	Tumor pro	1	181.0329	45.97572	2.29E-42	1	5.454545
Q13625	1128	125615.6	5.93	Apoptosis-	TP53BP2	TP53BP2	Apoptosis-	5	304.4957	48.84044	4.67E-52	5	5.939716
Q12888	1972	213572.6	4.34	TP53-bind	TP53BP1	TP53BP1	TP53-bind	7	364.0954	50.87169	1.52E-59	7	4.716024
O94842	621	66194.4	4.68	TOX high r	TOX4	TOX4	TOX high r	3	212.875	49.7399	2.56E-55	2	5.958132
Q9NXH8	423	46913.5	10.42	Torsin-4A	TOR4A	TOR4A	Torsin-4A	8	277.5857	47.56579	1.3E-47	8	24.8227
Q5JU69	321	35713.9	8.05	Torsin-2A	TOR2A	TOR2A	Torsin-2A	1	122.6092	40.547	3.93E-27	1	4.049844
O14657	336	37978.5	8.75	Torsin-1B	TOR1B	TOR1B	Torsin-1B	2	117.8161	47.69772	4.61E-48	2	7.440476
Q8NFQ8	470	51262.9	4.59	Torsin-1A-	TOR1AIP2	TOR1AIP2	Torsin-1A-	2	124.1189	47.49797	2.21E-47	2	5.531915
Q5JTV8	583	66247.9	8.41	Torsin-1A-	TOR1AIP1	TOR1AIP1	Torsin-1A-	6	406.9807	53.77409	7.03E-71	6	13.20755
Q9NS56	1045	119196.4	9.99	E3 ubiquiti	TOPORS	TOPORS	E3 ubiquiti	3	95.56547	45.63886	2.67E-41	3	2.77512
O95985	862	96661.2	8.17	DNA topoi	TOP3B	TOP3B	DNA topoi	10	257.0185	49.66013	5.02E-55	9	13.34107
Q13472	1001	112371.5	8.41	DNA topoi	TOP3A	TOP3A	DNA topoi	2	70.67496	45.04328	1.88E-39	2	2.597403
Q02880	1626	183265.8	8.16	DNA topoi	TOP2B	TOP2B	DNA topoi	51	4613.847	53.13834	2.4E-68	41	34.37884
P11388	1531	174383.9	9.17	DNA topoi	TOP2A	TOP2A	DNA topoi	41	2841.036	51.97151	9.25E-64	41	25.93076
Q969P6	601	69871.4	9.94	DNA topoi	TOP1MT	TOP1MT	DNA topoi	4	52.41071	46.55602	3.05E-44	4	7.986689
P11387	765	90725.2	9.95	DNA topoi	TOP1	TOP1	DNA topoi	46	37979.09	50.85493	1.75E-59	41	51.24183
Q96HA7	1378	150926.8	6.39	Tonsoku-li	TONSL	TONSL	Tonsoku-li	7	218.0828	48.16829	1.09E-49	7	6.458636
O94826	608	67454.2	7.14	Mitochonc	TOMM70	TOMM70	Mitochonc	6	113.8018	45.18337	7E-40	6	16.77632
Q96B49	74	8001.9	4.47	Mitochonc	TOMM6	TOMM6	Mitochonc	1	195.3601	50.80595	2.7E-59	1	18.91892
O96008	361	37892.9	7.29	Mitochonc	TOMM40	TOMM40	Mitochonc	9	857.8325	49.3481	6.98E-54	9	31.57895
Q15785	309	34559	9.35	Mitochonc	TOMM34	TOMM34	Mitochonc	2	123.6989	49.5414	1.37E-54	2	7.119741
Q9NS69	142	15521.5	3.99	Mitochonc	TOMM22	TOMM22	Mitochonc	3	154.9424	48.37881	2.02E-50	3	30.98592
Q15388	145	16297.7	8.85	Mitochonc	TOMM20	TOMM20	Mitochonc	2	389.1744	49.77722	1.88E-55	2	22.75862
Q6ZVM7	507	55555.8	4.41	TOM1-like	TOM1L2	TOM1L2	TOM1-like	3	131.9426	44.5909	4.44E-38	3	6.706114
O60784	492	53817.9	4.32	Target of M	TOM1	TOM1	Target of M	2	184.6208	52.98827	9.49E-68	2	6.097561
Q9H0E2	274	30281.7	5.76	Toll-intera	TOLLIP	TOLLIP	Toll-intera	3	71.15604	42.65226	1.54E-32	3	16.42336
Q96GM8	510	56547.3	7.19	Target of E	TOE1	TOE1	Target of E	7	409.9549	45.3522	2.11E-40	7	13.92157
O15417	2968	314515.7	9.04	Trinucleoti	TNRC18	TNRC18	Trinucleoti	7	163.6831	54.22862	9.82E-73	7	3.234501
Q9Y5L0	923	104202.5	5.3	Transporti	TNPO3	TNPO3	Transporti	9	189.7827	50.69579	7.02E-59	9	12.7844
Q92973	898	102354	4.59	Transporti	TNPO1	TNPO1	Transporti	11	387.3992	47.93187	7.25E-49	8	13.58575
Q9H2K2	1166	126916.9	7.2	Poly [ADP-	TNKS2	TNKS2	Poly [ADP-	5	165.6272	55.67759	1.03E-78	5	5.403087
Q9C0C2	1729	181794	4.49	182 kDa ta	TNKS1BP1	TNKS1BP1	182 kDa ta	10	163.1533	52.93061	1.61E-67	10	8.444187

O95271	1327	142038.2	7.05	Poly [ADP-	TNKS	TNKS	Poly [ADP-	19	468.1929	51.49951	5.99E-62	12	17.10625
Q9UKE5	1360	154942.1	7.16	TRAF2 anc	TNIK	TNIK	TRAF2 anc	13	549.3892	53.82073	4.46E-71	8	12.05882
O95407	300	32679.2	8.27	Tumor nec	TNFRSF6B	TNFRSF6B	Tumor nec	6	215.6288	49.25371	1.53E-53	6	22
O14763	440	47877.9	5.25	Tumor nec	TNFRSF10I	TNFRSF10I	Tumor nec	1	124.3055	43.67704	2.12E-35	1	3.409091
Q03169	654	72660.5	6.42	Tumor nec	TNFAIP2	TNFAIP2	Tumor nec	3	93.58204	44.61126	3.86E-38	3	5.810398
Q13829	316	36203.9	8.13	BTB/POZ c	TNFAIP1	TNFAIP1	BTB/POZ c	4	129.8893	49.18918	2.63E-53	3	17.72152
Q9Y320	296	34037.3	8.91	Thioredoxi	TMX2	TMX2	Thioredoxi	2	163.9489	40.57777	3.31E-27	2	7.77027
Q9H3N1	280	31790.9	4.63	Thioredoxi	TMX1	TMX1	Thioredoxi	5	365.2833	51.37675	1.8E-61	5	18.92857
Q9BVT8	246	26261	5.53	Transmem	TMUB1	TMUB1	Transmem	1	154.9756	34.1963	1.08E-14	1	4.065041
Q6ZXV5	915	104007.7	9.2	Protein O-	TMTC3	TMTC3	Protein O-	3	94.16263	45.86776	5.05E-42	3	4.153005
Q6ZT21	453	49452	6.92	Transmem	TMPPE	TMPPE	Transmem	1	745.3859	28.25309	2.94E-08	1	2.207506
P42166	694	75491.3	7.74	Lamina-as	TMPO	TMPO	Lamina-as	14	1532.114	51.7904	4.6E-63	7	25.64841
P42167	454	50669.8	9.97	Lamina-as	TMPO	TMPO	Lamina-as	5	666.4262	50.07874	1.44E-56	5	13.43612
Q9NYL9	352	39594.4	4.83	Tropomod	TMOD3	TMOD3	Tropomod	13	1250.375	50.90356	1.15E-59	13	44.03409
Q9NZR1	351	39594.7	4.93	Tropomod	TMOD2	TMOD2	Tropomod	2	270.512	50.12873	9.35E-57	1	6.837607
P28289	359	40568.8	4.74	Tropomod	TMOD1	TMOD1	Tropomod	2	61.36815	42.27872	1.56E-31	2	7.520891
Q9NVH6	421	49517.2	7.79	Trimethylly	TMLHE	TMLHE	Trimethylly	1	9.558078	30.35717	5.63E-10	1	1.900238
Q9BUB7	260	28969	9.17	Transmem	TMEM70	TMEM70	Transmem	1	59.21276	37.95459	2.37E-21	1	4.615385
Q6PI78	240	25497.5	8.76	Transmem	TMEM65	TMEM65	Transmem	1	53.56076	37.10354	1.15E-19	1	4.583333
Q5T3F8	832	94957.3	7.42	CSC1-like	TMEM63B	TMEM63B	CSC1-like	1	44.3405	40.34798	1.18E-26	1	1.5625
Q9BTV4	400	44875.1	8.41	Transmem	TMEM43	TMEM43	Transmem	12	460.4786	51.59082	2.69E-62	12	32.5
Q8WWA1	233	25494.6	5.32	Transmem	TMEM40	TMEM40	Transmem	3	516.9268	49.49591	2.02E-54	3	17.16738
Q9H6F2	299	33259.6	8.41	Trimeric in	TMEM38A	TMEM38A	Trimeric in	2	509.8797	34.643	2.22E-15	2	6.688963
P57088	247	27977.8	9.99	Transmem	TMEM33	TMEM33	Transmem	6	665.9954	48.0489	2.84E-49	6	27.12551
Q9H330	879	97356.2	9.17	Transmem	TMEM245	TMEM245	Transmem	1	94.55782	37.33722	4.05E-20	1	1.478953
Q6NUQ4	689	77149.8	9.52	Transmem	TMEM214	TMEM214	Transmem	2	131.5996	43.17377	5.63E-34	2	3.193033
Q6UW68	189	21197.6	8.75	Transmem	TMEM205	TMEM205	Transmem	2	122.9514	45.77825	9.7E-42	2	15.87302
Q5SNT2	666	72234.8	9.45	Transmem	TMEM201	TMEM201	Transmem	3	117.758	48.21481	7.56E-50	3	4.504505
Q8IY95	271	30922	8.08	Transmem	TMEM192	TMEM192	Transmem	1	105.2946	46.93388	1.74E-45	1	5.535055
Q9NVA4	438	50141.2	6.23	Transmem	TMEM184(	TMEM184(	Transmem	1	114.7454	44.45058	1.16E-37	1	2.968037
Q7Z7N9	219	23550	7.92	Transmem	TMEM179I	TMEM179I	Transmem	1	66.11635	35.62244	5.55E-17	1	7.305936
Q3YBM2	270	29055.6	8.58	Transmem	TMEM176I	TMEM176I	Transmem	1	50.38076	21.38563	6.88E-06	1	4.074074
Q9HC07	324	34905.4	7.05	Putative di	TMEM165	TMEM165	Putative di	1	101.5927	46.15094	6.31E-43	1	6.17284
Q9NX00	188	19657.3	8.23	Transmem	TMEM160	TMEM160	Transmem	1	176.5767	47.8481	1.41E-48	1	6.914894
Q8IUX1	230	25943.2	9.08	Complex I	TMEM126I	TMEM126I	Complex I	1	32.83889	37.5298	1.69E-20	1	6.086957
Q8N131	208	21530.9	9.07	Porimin	TMEM123	TMEM123	Porimin	1	41.52703	29.1429	6.54E-09	1	4.326923
Q9BXJ8	343	40610	9.24	Ion channe	TMEM120/	TMEM120/	Ion channe	1	24.72034	25.27398	8.87E-07	1	2.623907
P17152	192	21540.5	7.4	Transmem	TMEM11	TMEM11	Transmem	2	205.2835	42.17876	2.88E-31	2	12.5
Q9BVC6	243	26209.6	11.09	Voltage-g	TMEM109	TMEM109	Voltage-g	1	875.3142	47.38413	5.39E-47	1	4.938272
Q9NUM4	274	31127.2	7.01	Transmem	TMEM106I	TMEM106I	Transmem	2	122.4724	45.45728	9.93E-41	2	8.394161
Q9BVK6	235	27277.2	8.23	Transmem	TMED9	TMED9	Transmem	3	536.0826	51.5134	5.3E-62	3	12.76596
Q9Y3B3	224	25171.4	6.9	Transmem	TMED7	TMED7	Transmem	5	205.8658	53.48941	9.5E-70	5	29.01786
Q9Y3A6	229	26004.6	4.46	Transmem	TMED5	TMED5	Transmem	3	93.71917	55.99715	4.49E-80	3	13.53712
Q7Z7H5	227	25942.7	8.48	Transmem	TMED4	TMED4	Transmem	4	244.8232	46.33715	1.58E-43	4	20.26432
Q9Y3Q3	217	24776.8	5.33	Transmem	TMED3	TMED3	Transmem	1	40.58798	43.47248	8.13E-35	1	4.608295
Q15363	201	22761.1	4.83	Transmem	TMED2	TMED2	Transmem	1	41.60525	45.89706	4.08E-42	1	5.970149
P49755	219	24975.7	7.55	Transmem	TMED10	TMED10	Transmem	5	832.2994	49.18329	2.76E-53	5	31.96347
Q13445	227	25205.6	4.11	Transmem	TMED1	TMED1	Transmem	1	69.8093	36.42621	2.12E-18	1	3.964758
Q9UM00	239	27078.8	10.93	Calcium lo	TMCO1	TMCO1	Calcium lo	1	119.4135	46.57455	2.66E-44	1	5.857741
Q9ULS5	477	53784.6	8.74	Transmem	TMCC3	TMCC3	Transmem	1	51.66381	46.09795	9.34E-43	1	1.886792
Q969X1	311	34607.2	7.81	Protein life	TMBIM1	TMBIM1	Protein life	2	122.2593	47.72381	3.75E-48	2	8.360129
Q96EY4	203	23864.1	9.87	Translatior	TMA16	TMA16	Translatior	11	1298.967	45.86055	5.32E-42	11	40.39409
Q92544	642	74518.1	6.51	Transmem	TM9SF4	TM9SF4	Transmem	1	128.6804	17.73428	2.52E-05	1	1.401869
Q9HD45	589	67887.7	7.23	Transmem	TM9SF3	TM9SF3	Transmem	3	197.9745	49.70168	3.54E-55	3	5.602716
Q99805	663	75774.9	7.47	Transmem	TM9SF2	TM9SF2	Transmem	3	172.8732	45.99943	1.92E-42	3	4.826546
O15321	606	68860.1	7.18	Transmem	TM9SF1	TM9SF1	Transmem	1	113.4265	36.88488	3E-19	1	1.815182
O76062	418	46405.3	9.04	Delta(14)-:	TM7SF2	TM7SF2	Delta(14)-:	2	103.6372	35.04406	5.1E-16	2	5.502392
Q9Y490	2541	269764.5	5.94	Talin-1	TLN1	TLN1	Talin-1	14	223.2356	50.48552	4.38E-58	12	7.792208
Q86UE8	772	87660.3	8.58	Serine/thre	TLK2	TLK2;TLK1	Serine/thre	1	54.92234	47.48159	2.51E-47	1	1.165803
P29401	623	67876.9	7.73	Transketol	TKT	TKT	Transketol	11	463.4586	49.35906	6.37E-54	11	23.27448
Q3LXA3	575	58946.5	7.56	Triokinase/	TKFC	TKFC	Triokinase/	4	107.813	48.15867	1.18E-49	4	9.043478
O00142	265	31004.5	8.58	Thymidine	TK2	TK2	Thymidine	4	162.6364	50.50228	3.8E-58	4	15.09434
P04183	234	25468.5	8.61	Thymidine	TK1	TK1	Thymidine	4	147.3008	38.42637	2.48E-22	4	22.64957
O95049	919	101396	6.73	Tight junct	TJP3	TJP3	Tight junct	19	803.7401	50.23543	3.75E-57	19	23.50381
Q9UDY2	1190	133956.8	7.43	Tight junct	TJP2	TJP2	Tight junct	17	391.0954	54.23286	9.49E-73	17	17.31092
Q07157	1748	195457.4	6.68	Tight junct	TJP1	TJP1	Tight junct	47	1494.373	51.03884	3.49E-60	47	32.32265
O75663	272	31443.7	5.78	TIP41-like	TIPRL	TIPRL	TIP41-like	4	241.6421	45.98361	2.16E-42	4	16.54412
Q9BSI4	451	50023	8.03	TERF1-inte	TINF2	TINF2	TERF1-inte	1	161.3725	54.79596	4.52E-75	1	2.882483
Q9GZM7	467	52387.1	6.98	Tubulointe	TINAGL1	TINAGL1	Tubulointe	7	380.0049	46.63779	1.65E-44	7	21.62741
P35625	211	24144.8	8.86	Metallopro	TIMP3	TIMP3	Metallopro	1	51.18916	45.66669	2.18E-41	1	5.687204
Q3ZCQ8	353	39645.8	8.55	Mitochonc	TIMM50	TIMM50	Mitochonc	8	771.9039	49.12741	4.38E-53	8	27.76204
O43615	452	51355.3	8.53	Mitochonc	TIMM44	TIMM44	Mitochonc	11	394.5356	52.72258	1.07E-66	11	26.54867
Q9BVV7	248	28202.2	10.17	Mitochonc	TIMM21	TIMM21	Mitochonc	3	174.6969	42.37142	8.83E-32	3	11.69355
Q99595	171	18023.5	8.04	Mitochonc	TIMM17A	TIMM17A	Mitochonc	1	245.8465	28.86818	1.07E-08	1	5.263158
Q9UNS1	1208	138657.1	5.08	Protein tim	TIMELESS	TIMELESS	Protein tim	8	153.0087	46.23109	3.48E-43	8	8.360927
Q4W5G0	525	59622.8	9.47	Tigger trar	TIGD2	TIGD2	Tigger trar	10	703.6404	55.35209	2.25E-77	10	23.61905
Q9NQ88	270	30062.3	7.79	Fructose-2	TIGAR	TIGAR	Fructose-2	1	61.23102	47.07217	6E-46	1	4.074074
Q13009	1591	177506.5	6.58	Rho guanii	TIAM1	TIAM1	Rho guanii	4	107.9394	46.19844	4.44E-43	4	3.205531
Q01085	375	41590.6	7.85	Nucleolysii	TIAL1	TIAL1	Nucleolysii	3	287.1723	45.9607	2.56E-42	1	8.266667
P31483	386	42963.1	7.85	Cytotoxic (	TIA1	TIA1	Cytotoxic (	1	45.52236	38.45122	2.2E-22	1	2.590674
Q9P016	225	25697.1	9.83	Thymocyte	THYN1	THYN1	Thymocyte	8	793.4403	48.04944	2.83E-49	8	38.22222
Q9BV44	507	57002.3	6.3	tRNA (gua	THUMPD3	THUMPD3	tRNA (gua	4	310.0559	48.50444	7.31E-51	4	7.889546
Q9NXG2	353	39315.2	8.02	THUMP dc	THUMPD1	THUMPD1	THUMP dc	4	209.1413	52.03233	5.34E-64	4	12.46459
Q6ZMP0	1018	112449.3	7.66	Thrombos	THSD4	THSD4	Thrombos	5	96.95822	45.99097	2.05E-42	5	7.170923
Q9Y2W1	955	108665	10.82	Thyroid hc	THRAP3	THRAP3	Thyroid hc	30	8268.066	53.90289	2.09E-71	29	29.21466
P52888	689	78839.1	5.93	Thimet olic	THOP1	THOP1	Thimet olic	3	103.5331	41.53718	1.35E-29	3	4.063861
Q6I9Y2	204	23742.7	5.43	THO comp	THOC7	THOC7	THO comp	2	52.8622	35.30788	1.88E-16	2	10.78431
Q86W42	341	37534.6	7.48	THO comp	THOC6	THOC6	THO comp	5	337.5039	48.9669	1.65E-52	5	17.59531
Q13769	683	78507.2	6.87	THO comp	THOC5	THOC5	THO comp	5	485.2818	47.25104	1.51E-46	5	9.224012
Q96J01	351	38771.3	6	THO comp	THOC3	THOC3	THO comp	5	134.8391	46.4806	5.39E-44	5	21.36752
Q8NI27	1593	182773.1	8.63	THO comp	THOC2	THOC2	THO comp	9	904.1697	46.80555	4.63E-45	9	6.277464
Q96FV9	657	75665.7	4.63	THO comp	THOC1	THOC1	THO comp	14	269.7233	47.82134	1.74E-48	14	25.57078
Q8IYQ7	743	83069.5	7.13	Threonine	THNSL1	THNSL1	Threonine	7	153.0566	47.51769	1.89E-47	7	12.65141

Q9NWX6	298	34830.6	8.12	Probable t	THG1L	THG1L	Probable t	2	188.7944	56.30406	2.41E-81	2	8.724832
P07996	1170	129381.7	4.51	Thrombos	THBS1	THBS1	Thrombos	5	297.2221	51.58704	2.78E-62	5	5.042735
Q8WY91	577	62889.3	9.84	Peroxynitri	THAP4	THAP4	Peroxynitri	2	107.0634	47.79208	2.19E-48	2	5.199307
Q96EK4	314	34454.8	9.3	THAP dom	THAP11	THAP11	THAP dom	1	142.5417	48.11181	1.72E-49	1	2.547771
Q96RS0	853	96619	4.57	Trimethylg	TGS1	TGS1	Trimethylg	9	357.816	49.10941	5.07E-53	9	10.31653
Q08188	693	76631.3	5.6	Protein-gl	TGM3	TGM3	Protein-gl	2	64.72666	44.14057	9.59E-37	2	3.463203
P21980	687	77328.2	4.86	Protein-gl	TGM2	TGM2	Protein-gl	13	776.8983	51.58978	2.72E-62	12	20.08734
Q03167	851	93498.6	5.49	Transformi	TGFBR3	TGFBR3	Transformi	2	138.9873	48.79618	6.73E-52	2	3.290247
P02786	760	84870.7	6.58	Transferrin	TFRC	TFRC	Transferrin	22	764.7648	53.28252	6.37E-69	22	37.63158
P0C1Z6	253	28278.1	4.88	TCF3 fusio	TFPT	TFPT	TCF3 fusio	2	254.1693	51.26434	4.82E-61	2	9.881423
Q9UBB9	837	96819.2	5.38	Tuftelin-in	TFIP11	TFIP11	Tuftelin-in	25	1605.745	54.96119	9.21E-76	25	33.57228
Q9NZI6	479	54626.3	7.01	Transcripti	TFCP2L1	TFCP2L1	Transcripti	1	330.3949	41.08425	1.89E-28	1	2.922756
Q12800	502	57255.3	5.61	Alpha-glol	TFCP2	TFCP2	Alpha-glol	3	226.9314	53.98298	9.92E-72	3	7.768924
Q9H5Q4	396	45348.4	9.63	Dimethylac	TFB2M	TFB2M	Dimethylac	4	92.75385	46.69251	1.09E-44	4	12.87879
Q8WVM0	346	39542.6	9.74	Dimethylac	TFB1M	TFB1M	Dimethylac	8	348.5262	49.6638	4.87E-55	8	26.01156
Q92754	450	49176.3	7.89	Transcripti	TFAP2C	TFAP2C	Transcripti	3	203.2713	45.22583	5.18E-40	2	10.88889
P05549	437	48061.7	8.12	Transcripti	TFAP2A	TFAP2A	Transcripti	1	53.3201	45.14868	8.96E-40		2.974828
Q00059	246	29096.4	10.32	Transcripti	**TFAM**	**TFAM**	Transcripti	11	917.062	51.76644	5.67E-63	11	38.61789
P02787	698	77049.2	7.13	Serotransfe	TF	TF	Serotransfe	18	342.1247	53.24655	8.84E-69	18	29.65616
Q9NXF1	929	105673.1	9.86	Testis-exp	TEX10	TEX10	Testis-exp	15	322.1254	49.71633	3.12E-55	15	20.34446
Q9UGI8	421	47996.1	7.72	Testin	TES	TES	Testin	11	867.5156	53.28346	6.32E-69	11	23.27791
Q9NYB0	399	44259.5	4.36	Telomeric	TERF2IP	TERF2IP	Telomeric	5	154.5422	53.26348	7.54E-69	5	16.54135
Q15554	542	59593	10.01	Telomeric	TERF2	TERF2	Telomeric	5	270.9406	51.43598	1.06E-61	5	11.99262
P54274	439	50245.1	6.31	Telomeric	TERF1	TERF1	Telomeric	14	375.0074	52.62379	2.58E-66	14	34.85194
Q8NDF8	572	63266.5	9.35	Terminal n	TENT4B	TENT4B	Terminal n	10	464.5307	51.59307	2.64E-62	10	22.55245
Q5XG87	792	84649.2	9.95	Terminal n	TENT4A	TENT4A	Terminal n	3	120.0251	46.77682	5.75E-45	3	4.924242
Q6PIY7	484	56027.2	9.82	Poly(A) RN	TENT2	TENT2	Poly(A) RN	7	329.492	47.09858	4.9E-46	7	16.32231
Q9P273	2699	300948	6.37	Teneurin-:	TENM3	TENM3	Teneurin-:	1	327.543	43.42114	1.14E-34	1	0.444609
Q96QE5	360	41675.8	9.8	Transcripti	TEFM	TEFM	Transcripti	2	112.9552	51.67089	1.33E-62	2	5.833333
Q86SX3	495	54230.5	8.09	Tubulin ep	TEDC1	TEDC1	Tubulin ep	2	89.49429	50.77129	3.64E-59	2	5.454545
Q9NZ01	308	36034.1	9.79	Very-long-	TECR	TECR	Very-long	8	811.1455	52.08311	3.38E-64	8	22.4026
P42680	631	73580.7	8.58	Tyrosine-p	TEC	TEC	Tyrosine-p	6	90.63545	43.13505	7.21E-34	6	11.25198
P28347	426	47945.2	8.27	Transcripti	TEAD1	TEAD1	Transcripti	1	278.4981	50.8019	2.79E-59	1	2.347418
Q9H7E2	651	73184.3	9.73	Tudor don	TDRD3	TDRD3	Tudor don	3	58.02261	43.12786	7.55E-34	3	6.144393
Q9NUW8	608	68419.9	7.72	Tyrosyl-DI	TDP1	TDP1	Tyrosyl-DI	11	433.4406	48.37196	2.13E-50	11	18.09211
P17987	556	60342.9	5.96	T-complex	TCP1	TCP1	T-complex	19	2005.73	55.2063	8.97E-77	19	38.1295
Q13428	1488	152104.7	9.79	Treacle pro	TCOF1	TCOF1	Treacle pro	26	2977.322	51.76294	5.86E-63	26	18.61559
Q13488	830	92967.2	7.13	V-type pro	TCIRG1	TCIRG1	V-type pro	2	67.11871	46.38503	1.1E-43	2	3.012048
P36402	384	41641.3	6.8	Transcripti	TCF7	TCF7;TCF7	Transcripti	1	650.2699	45.03014	2.07E-39	1	2.864583
Q9BQ70	676	76665.9	6.31	Ribosome	TCF25	TCF25	Ribosome	7	239.0175	39.92382	1.18E-25	7	10.50296
Q9UGU0	1960	211770	9.42	Transcripti	TCF20	TCF20	Transcripti	13	390.205	52.31821	4.05E-65	13	9.234694
O14776	1098	123900.1	9.15	Transcripti	TCERG1	TCERG1	Transcripti	11	506.668	48.70041	1.48E-51	10	10.10929
P23193	301	33969.3	8.55	Transcripti	TCEA1	TCEA1	Transcripti	4	244.5238	50.61937	1.37E-58	2	14.61794
A6NFQ2	919	100905.3	7.17	TRPM8 cha	TCAF2	TCAF2	TRPM8 cha	3	195.6762	51.31778	3E-61	3	3.373232
Q9Y4C2	921	102125	6.51	TRPM8 cha	TCAF1	TCAF1	TRPM8 cha	2	82.47619	36.75623	5.22E-19	2	2.171553
Q969Z0	631	70737.3	7.45	FAST kinas	TBRG4	TBRG4	FAST kinas	8	163.6943	49.42883	3.55E-54	8	17.43265
Q3YBR2	411	44945.2	8.65	Transformi	TBRG1	TBRG1	Transformi	1	266.9387	38.97843	1.62E-23	1	3.649635
Q12788	808	89033.9	6.9	Transducir	TBL3	TBL3	Transducir	14	236.3494	51.69284	1.09E-62	14	22.64851
Q9Y4P3	447	49797.4	10.02	Transducir	TBL2	TBL2	Transducir	13	484.691	52.83637	3.83E-67	13	37.36018
Q9UHD2	729	83641.5	6.77	Serine/thre	TBK1	TBK1	Serine/thre	3	87.48637	49.26485	1.4E-53	3	4.663923
Q15813	527	59345.7	6.73	Tubulin-sp	TBCE	TBCE	Tubulin-sp	4	97.3084	51.52363	4.86E-62	4	8.349146
Q9BTW9	1192	132598.5	6.11	Tubulin-sp	TBCD	TBCD	Tubulin-sp	4	188.1784	46.80977	4.49E-45	4	3.52349
Q99426	244	27325.3	4.78	Tubulin-fo	TBCB	TBCB	Tubulin-fo	4	167.0479	50.30312	2.09E-57	4	16.80328
Q66K14	1250	140523.4	4.9	TBC1 dom	TBC1D9B	TBC1D9B	TBC1 dom	3	86.84238	40.1056	4.44E-26	3	2.96
O60343	1298	146562	7	TBC1 dom	TBC1D4	TBC1D4	TBC1 dom	4	170.2834	41.61671	8.47E-30	4	3.620955
Q9ULP9	559	62918.9	7.39	TBC1 dom	TBC1D24	TBC1D24	TBC1 dom	1	247.8595	46.95378	1.49E-45	1	2.683363
Q8WUA7	517	59120.4	5.84	TBC1 dom	TBC1D22A	TBC1D22A	TBC1 dom	1	94.77402	36.50711	1.51E-18	1	2.901354
Q8TC07	691	79490	5.37	TBC1 dom	TBC1D15	TBC1D15	TBC1 dom	2	56.67705	26.16329	4.07E-07	2	3.617945
Q5VWN6	2430	268840.2	5.72	Protein TA	TASOR2	TASOR2	Protein TA	14	311.3152	48.91994	2.42E-52	14	7.283951
Q9UK61	1670	189030	5.58	Protein TA	TASOR	TASOR	Protein TA	7	207.3073	49.03727	9.19E-53	7	4.491018
Q9BW92	718	81035.3	7.31	Threonine-	TARS2	TARS2	Threonine-	4	318.2321	49.51201	1.76E-54	4	6.545961
P26639	723	83434.5	6.64	Threonine-	TARS1	TARS1	Threonine-	17	490.8773	54.26817	6.86E-73	16	26.14108
Q13148	414	44739.6	6.07	TAR DNA-	TARDBP	TARDBP	TAR DNA-	5	384.2276	49.70877	3.33E-55	5	14.25121
Q15633	366	39038.9	6.51	RISC-loadi	TARBP2	TARBP2	RISC-loadi	4	175.8831	47.62043	8.47E-48	4	12.56831
O15533	448	47570.2	6.99	Tapasin	TAPBP	TAPBP	Tapasin	2	153.8684	43.51527	6.15E-35	2	5.133929
Q03519	686	75663.2	8.1	Antigen pe	TAP2	TAP2;ABCI	Antigen pe	1	68.12144	36.38435	2.53E-18	1	1.166181
Q03518	748	80963.9	7.34	Antigen pe	TAP1	TAP1	Antigen pe	2	47.72365	30.54075	3.73E-10	2	3.743316
Q9H2K8	898	105405.2	7.31	Serine/thre	TAOK3	TAOK3	Serine/thre	1	348.4803	45.65093	2.45E-41	1	0.890869
Q9UL54	1235	138250.2	7.27	Serine/thre	TAOK2	TAOK2	Serine/thre	5	273.7043	43.47232	8.14E-35	5	4.453441
Q7L7X3	1001	116069.6	7.71	Serine/thre	TAOK1	TAOK1	Serine/thre	5	202.502	44.84167	7.77E-39	3	5.594406
Q96BW9	452	51066.4	8.01	Phosphatic	TAMM41	TAMM41	Phosphatic	3	47.75739	51.99899	7.24E-64	3	8.628319
P37837	337	37539.7	6.8	Transaldol	TALDO1	TALDO1	Transaldol	4	212.1134	54.15765	1.9E-72	4	13.64985
P37802	199	22391.4	8.45	Transgelin	TAGLN2	TAGLN2	Transgelin	1	62.31964	45.02608	2.13E-39	1	6.030151
Q16594	264	28973.5	9	Transcripti	TAF9	TAF9	Transcripti	8	529.4794	51.29843	3.57E-61	3	28.78788
Q7Z7C8	310	34262.1	6.41	Transcripti	TAF8	TAF8	Transcripti	7	370.1721	51.6875	1.15E-62	7	30.96774
Q15545	349	40258.7	4.83	Transcripti	TAF7	TAF7	Transcripti	7	259.0731	46.65845	1.41E-44	7	20.63037
Q9Y6J9	622	67813.7	9.19	TAF6-like I	TAF6L	TAF6L	TAF6-like I	2	392.5436	44.6862	2.29E-38	2	3.858521
P49848	677	72667.8	8.8	Transcripti	TAF6	TAF6	Transcripti	22	1220.432	52.57372	4.06E-66	22	38.25702
Q15542	800	86829.5	5.36	Transcripti	TAF5	TAF5	Transcripti	13	628.7878	53.57719	4.3E-70	13	22.625
Q92750	862	91089.7	10.23	Transcripti	TAF4B	TAF4B	Transcripti	3	147.1224	39.72654	3.38E-25	3	4.640371
O00268	1085	110113.3	10.64	Transcripti	TAF4	TAF4	Transcripti	12	805.0352	54.33748	3.69E-73	12	12.07373
Q5VWG9	929	103580.9	9.68	Transcripti	TAF3	TAF3	Transcripti	12	522.0836	53.47412	1.09E-69	12	14.96233
Q6P1X5	1199	136970.3	8.27	Transcripti	TAF2	TAF2	Transcripti	35	4217.398	51.50221	5.85E-62	35	34.52877
Q15572	869	95212.6	8.69	TATA box-	TAF1C	TAF1C	TATA box-	3	87.78418	48.52131	6.37E-51	3	6.329114
Q92804	592	61830	8.16	TATA-binc	TAF15	TAF15	TATA-binc	6	1192.895	48.62463	2.74E-51	6	8.614865
Q16514	161	17924.3	8.23	Transcripti	TAF12	TAF12	Transcripti	2	342.8812	44.49799	8.38E-38	2	11.80124
Q15544	211	23306.9	4.51	Transcripti	TAF11	TAF11	Transcripti	1	331.7757	48.87783	3.43E-52	1	5.21327
Q12962	218	21711	6.52	Transcripti	TAF10	TAF10	Transcripti	1	151.8448	37.82694	4.3E-21	1	6.422018
P21675	1893	214711.7	4.69	Transcripti	TAF1	TAF1	Transcripti	21	419.4664	54.64531	1.94E-74	11	11.99155
Q9BSH4	297	32476.8	8.24	Translatior	TACO1	TACO1	Translatior	1	35.04485	17.23716	2.98E-05	1	4.040404
Q15750	504	54643.7	5.21	TGF-beta-	TAB1	TAB1	TGF-beta-	1	161.1006	27.85845	5.3E-08	1	2.18254
Q96C24	671	76023	9.42	Synaptota	SYTL4	SYTL4	Synaptota	1	166.7453	22.63497	4.1E-06	1	1.639344
Q4VX76	610	68559.1	9.78	Synaptota	SYTL3	SYTL3	Synaptota	2	76.89806	45.59763	3.6E-41	2	4.262295

O43581	403	45501	9.74	Synaptotag	SYT7	SYT7	Synaptotag	8	584.1781	52.12589	2.32E-64	8	22.33251
Q9BQG1	590	63303.2	6.6	Synaptotag	SYT3	SYT3	Synaptotag	2	34.94876	20.38086	9.96E-06	2	3.050847
P21579	422	47572.8	8.33	Synaptotag	SYT1	SYT1	Synaptotag	9	288.5933	52.1995	1.18E-64	6	24.17062
Q16563	259	28565	8.55	Synaptoph	SYPL1	SYPL1	Synaptoph	1	999.1761	41.19085	1.02E-28	1	4.247104
P57105	145	15928	6.25	Synaptojar	SYNJ2BP	SYNJ2BP	Synaptojar	1	53.50249	38.36346	3.37E-22	1	9.655172
O43760	224	24809.8	4.53	Synaptogy	SYNGR2	SYNGR2	Synaptogy	3	214.8518	47.08447	5.46E-46	3	12.5
Q6ZMZ3	975	112215.5	6.15	Nesprin-3	SYNE3	SYNE3	Nesprin-3	4	69.21836	50.02965	2.19E-56	4	4.615385
O60506	623	69602.3	8.88	Heterogen	SYNCRIP	SYNCRIP	Heterogen	22	8929.193	50.20599	4.84E-57	22	38.52327
O95926	243	28722.1	8.81	Pre-mRNA	SYF2	SYF2	Pre-mRNA	3	290.916	47.52699	1.76E-47	3	15.22634
Q9NX95	663	72387.5	6.19	Syntabulin	SYBU	SYBU	Syntabulin	2	217.6692	47.82753	1.66E-48	1	2.865762
O95425	2214	247744.2	6.96	Supervillin	SVIL	SVIL	Supervillin	7	199.6306	50.39266	9.73E-58	7	4.245709
Q15022	739	83054.3	9.13	Polycomb	SUZ12	SUZ12	Polycomb	11	855.5864	51.54303	4.09E-62	11	16.77943
Q9H5I1	410	46682	8.27	Histone-ly	SUV39H2	SUV39H2	Histone-ly	8	543.4409	48.19227	9.04E-50	8	22.68293
O43463	412	47907.1	8.07	Histone-ly	SUV39H1	SUV39H1	Histone-ly	3	104.9905	40.79296	9.91E-28	3	8.495146
Q9UGT4	822	90206.9	6.24	Sushi dom	SUSD2	SUSD2	Sushi dom	8	298.4989	52.51185	7.06E-66	8	14.11192
O75683	361	41450	11.35	Surfeit locu	SURF6	SURF6	Surfeit locu	11	1035.185	51.26148	4.95E-61	11	27.70083
O15260	269	30393.7	7.93	Surfeit locu	SURF4	SURF4	Surfeit locu	3	256.531	53.01862	7.17E-68	3	11.89591
Q15527	256	29617.3	9.64	Surfeit locu	SURF2	SURF2	Surfeit locu	5	1036.08	46.34073	1.53E-43	5	21.09375
Q15526	300	33331.1	10.06	Surfeit locu	SURF1	SURF1	Surfeit locu	3	156.2664	45.34995	2.14E-40	3	12.33333
Q8IYB8	786	87990.3	8.07	ATP-depe	SUPV3L1	SUPV3L1	ATP-depe	4	158.8821	40.25488	1.97E-26	4	6.870229
Q7KZ85	1726	199071.1	4.54	Transcripti	SUPT6H	SUPT6H	Transcripti	48	3041.859	54.18924	1.4E-72	48	30.88065
O00267	1087	120998.9	4.69	Transcripti	SUPT5H	SUPT5H	Transcripti	23	963.8273	52.04472	4.78E-64	23	25.023
P63272	117	13193.1	8.2	Transcripti	SUPT4H1	SUPT4H1	Transcripti	2	499.8238	48.38818	1.87E-50	2	21.36752
Q9Y5B9	1047	119912.9	5.36	FACT comp	SUPT16H	SUPT16H	FACT comp	67	54934.15	51.78236	4.94E-63	67	53.48615
Q9UH99	717	80310.2	6.71	SUN doma	SUN2	SUN2	SUN doma	15	658.0636	51.56555	3.36E-62	14	25.94142
O94901	785	87109	6.93	SUN doma	SUN1	SUN1	SUN doma	17	525.889	53.75925	8.02E-71	17	28.53503
P55854	103	11637	5.18	Small ubiq	SUMO3	SUMO3;SU	Small ubiq	1	152.6113	49.262	1.43E-53	1	11.65049
P0DMM9	295	34196	5.88	Sulfotransf	SULT1A3	SULT1A3;S	Sulfotransf	1	60.01218	25.57963	6.92E-07	1	3.050847
Q8IWU5	870	100454	9.54	Extracellul	SULF2	SULF2	Extracellul	1	98.01564	49.73517	2.67E-55	1	1.494253
Q9Y2Z0	365	41023.8	4.8	Protein SG	SUGT1	SUGT1	Protein SG	8	314.9792	49.79403	1.63E-55	8	26.57534
Q8IX01	1082	120205.9	7.3	SURP and	SUGP2	SUGP2	SURP and	23	1712.691	52.01512	6.24E-64	23	25.23105
Q8IWZ8	645	72470.4	7.77	SURP and	SUGP1	SUGP1	SURP and	14	774.3412	56.12744	1.26E-80	14	24.18605
Q9H7L9	328	38135.8	5.33	Sin3 histor	SUDS3	SUDS3	Sin3 histor	2	583.48	45.74458	1.24E-41	2	5.792683
Q96I99	432	46510.2	6.18	Succinate-	SUCLG2	SUCLG2	Succinate-	8	260.2393	51.78016	5.03E-63	8	20.37037
P53597	346	36249.5	9.04	Succinate-	SUCLG1	SUCLG1	Succinate-	4	552.2313	49.0832	6.3E-53	4	17.63006
Q9P2R7	463	50316.9	7.5	Succinate-	SUCLA2	SUCLA2	Succinate-	8	274.8568	50.0897	1.31E-56	8	17.06263
P53999	127	14395.2	10.32	Activated I	SUB1	SUB1	Activated I	8	5153.491	56.58909	1.52E-82	8	46.45669
O00186	592	67763.6	7.88	Syntaxin-b	STXBP3	STXBP3	Syntaxin-b	1	50.32343	41.47724	1.93E-29	1	1.52027
Q15833	593	66451.8	6.52	Syntaxin-b	STXBP2	STXBP2	Syntaxin-b	5	114.6035	48.68527	1.67E-51	5	12.64755
P61764	594	67568	6.96	Syntaxin-b	STXBP1	STXBP1	Syntaxin-b	1	129.9771	24.15738	1.89E-06	1	1.346801
O15400	261	29815.3	5.24	Syntaxin-7	STX7	STX7	Syntaxin-7	6	202.8608	51.12115	1.69E-60	6	30.2682
O43752	255	29175.7	4.57	Syntaxin-6	STX6	STX6	Syntaxin-6	2	69.74065	47.76002	2.82E-48	2	9.411765
Q12846	297	34179.7	6.2	Syntaxin-4	STX4	STX4	Syntaxin-4	1	63.13227	41.11127	1.61E-28	1	3.703704
Q86Y82	276	31641.6	5.28	Syntaxin-1	STX12	STX12	Syntaxin-1	2	86.15273	47.26675	1.34E-46	2	11.5942
O60499	249	28113.9	4.53	Syntaxin-1	STX10	STX10	Syntaxin-1	1	45.21679	44.63089	3.37E-38	1	4.417671
Q9UNE7	303	34855.9	5.68	E3 ubiquiti	STUB1	STUB1	E3 ubiquiti	4	183.281	52.1583	1.73E-64	4	16.83168
Q8TCJ2	826	93673.5	9.17	Dolichyl-d	STT3B	STT3B	Dolichyl-d	6	414.9573	50.85012	1.83E-59	6	7.990315
P46977	705	80528.8	8.17	Dolichyl-d	STT3A	STT3A	Dolichyl-d	9	994.0019	49.91539	5.82E-56	9	11.48936
Q13033	797	87208.6	5	Striatin-3	STRN3	STRN3	Striatin-3	2	50.93044	48.42333	1.41E-50	2	3.136763
O43815	780	86131.3	4.91	Striatin	STRN	STRN	Striatin	2	79.3823	36.40074	2.36E-18	2	3.846154
Q96SI9	672	73651.9	9	Spermatid	STRBP	STRBP	Spermatid	8	386.591	50.93445	8.72E-60	8	11.30952
Q9Y3F4	350	38438	4.75	Serine-thr	STRAP	STRAP	Serine-thr	12	489.6426	51.6775	1.25E-62	12	40.85714
Q8WXE9	905	101164.2	5.05	Stonin-2	STON2	STON2	Stonin-2	1	1560.776	41.81562	2.59E-30	1	0.994475
Q9UJZ1	356	38533.6	7.5	Stomatin-l	STOML2	STOML2	Stomatin-l	8	1732.546	49.77165	1.97E-55	8	32.58427
P27105	288	31730.4	8.07	Stomatin	STOM	STOM	Stomatin	1	257.3544	37.44731	2.46E-20	1	4.166667
Q9H668	368	42118.6	5.98	CST compl	STN1	STN1	CST compl	6	212.7717	51.34083	2.45E-61	6	18.20652
Q9UEW8	545	59473.5	6.21	STE20/SPS	STK39	STK39	STE20/SPS	7	676.9834	49.84101	1.1E-55	7	14.86239
Q13188	491	56300.7	4.88	Serine/thre	STK3	STK3	Serine/thre	7	259.5903	52.48337	9.13E-66	4	16.49695
O00506	426	48111.3	6.73	Serine/thre	STK25	STK25	Serine/thre	1	128.752	43.33257	2.02E-34		3.051643
Q9Y6E0	443	49307.5	5.41	Serine/thre	STK24	STK24	Serine/thre	8	295.6912	46.17255	5.38E-43	4	18.73589
P31948	543	62638.7	6.78	Stress-indu	STIP1	STIP1	Stress-indu	13	491.7844	51.83094	3.23E-63	13	23.57274
Q86WV6	379	42192.1	7.06	Stimulator	STING1	STING1	Stimulator	1	221.2081	45.65641	2.35E-41	1	2.37467
Q13586	685	77422.8	6.65	Stromal int	STIM1	STIM1	Stromal int	2	74.69717	40.97322	3.56E-28	1	3.649635
Q9H5V9	222	25624.4	9	STING ER e	STEEP1	STEEP1	STING ER e	4	393.2578	45.23731	4.78E-40	4	22.52252
Q9NUL3	570	62608.1	10.24	Double-str	STAU2	STAU2	Double-str	18	1863.675	55.13029	1.85E-76	18	37.01754
O95793	577	63181.9	10.01	Double-str	STAU1	STAU1	Double-str	21	2549.79	51.73942	7.23E-63	21	39.16811
P40763	770	88067.2	6.23	Signal tran	STAT3	STAT3	Signal tran	11	375.2302	47.77236	2.56E-48	11	20.90909
P52630	851	97915.4	5.18	Signal tran	STAT2	STAT2	Signal tran	1	21.80722	36.16156	6.37E-18	1	1.292597
P42224	750	87334.2	5.92	Signal tran	STAT1	STAT1	Signal tran	22	652.069	57.51108	3.86E-86	22	33.33333
Q14849	445	50501.6	8.55	StAR-relat	STARD3	STARD3	StAR-relat	1	43.02076	33.48577	1.15E-13	1	1.797753
Q9Y5Y6	855	94769	6.53	Suppresso	ST14	ST14	Suppresso	5	152.5588	51.08725	2.28E-60	5	8.654971
P50502	369	41331.4	4.92	Hsc70-inte	ST13	ST13;ST13I	Hsc70-inte	1	203.3172	47.02027	8.95E-46	1	3.523035
Q9NP77	194	22574.3	4.98	RNA polyn	SSU72	SSU72	RNA polyn	4	178.7572	55.29619	3.78E-77	4	22.68041
Q08945	709	81074.2	6.85	FACT comp	SSRP1	SSRP1	FACT comp	33	79858.17	50.48109	4.55E-58	33	50.21157
P51571	173	18998.4	6.09	Translocor	SSR4	SSR4	Translocor	5	439.4816	50.31245	1.93E-57	5	36.41618
Q9UNL2	185	21080.3	10.17	Translocor	SSR3	SSR3	Translocor	1	546.5236	47.53709	1.63E-47	1	7.567568
P43307	286	32235.1	4.11	Translocor	SSR1	SSR1	Translocor	2	729.0792	45.28944	3.3E-40	2	9.090909
Q8TE77	659	72995	4.97	Protein ph	SSH3	SSH3	Protein ph	3	220.8474	46.32077	1.78E-43	3	4.704097
Q8WYL5	1049	115509.8	6.19	Protein ph	SSH1	SSH1	Protein ph	3	79.13069	45.63136	2.82E-41	3	3.050524
Q04837	148	17259.6	10.04	Single-stra	SSBP1	SSBP1	Single-stra	5	1244.822	55.72183	6.88E-79	5	40.54054
P05455	408	46836.8	7.14	Lupus La p	SSB	SSB	Lupus La p	15	774.6969	52.28384	5.55E-65	15	42.89216
Q13242	221	25542	8.85	Serine/arg	SRSF9	SRSF9	Serine/arg	12	27537.45	49.39248	4.81E-54	12	45.70136
Q9BRL6	282	32287.1	12.22	Serine/arg	SRSF8	SRSF8	Serine/arg	1	43.07868	50.76389	3.88E-59	1	6.028369
Q16629	238	27366.2	12.33	Serine/arg	SRSF7	SRSF7	Serine/arg	9	33821.04	48.44235	1.21E-50	9	35.29412
Q13247	344	39586.3	11.94	Serine/arg	SRSF6	SRSF6	Serine/arg	10	21533.6	52.52205	6.46E-66	7	28.19767
Q13243	272	31263.4	12.1	Serine/arg	SRSF5	SRSF5	Serine/arg	5	3623.609	50.14802	7.92E-57	5	21.32353
Q08170	494	56677.7	12.04	Serine/arg	SRSF4	SRSF4	Serine/arg	9	1590.605	50.02727	2.23E-56	9	14.37247
P84103	164	19329.4	12.15	Serine/arg	SRSF3	SRSF3	Serine/arg	7	47021.02	48.71161	1.35E-51	6	38.41463
Q01130	221	25476	12.36	Serine/arg	SRSF2	SRSF2	Serine/arg	2	442.4947	51.09227	2.18E-60	2	14.93213
Q05519	484	53541.6	11.24	Serine/arg	SRSF11	SRSF11	Serine/arg	8	2442.637	50.82766	2.23E-59	8	18.18182
O75494	262	31300.2	11.79	Serine/arg	SRSF10	SRSF10	Serine/arg	8	12434.93	50.31076	1.96E-57	5	20.61069
Q07955	248	27744.3	10.77	Serine/arg	SRSF1	SRSF1	Serine/arg	19	65757.41	49.92877	5.19E-56	18	49.19355
Q9BXP5	876	100665.6	5.76	Serrate RN	SRRT	SRRT	Serrate RN	23	1868.005	50.77161	3.63E-59	23	26.48402

Q9UQ35	2752	299611	12.56	Serine/arg	SRRM2	SRRM2	Serine/arg	50	29071.01	52.11148	2.63E-64	50	24.78198
Q8IYB3	904	102334	12.35	Serine/arg	SRRM1	SRRM1	Serine/arg	16	1266.367	49.39509	4.71E-54	16	20.35398
P78539	464	51571.4	8.77	Sushi repe	SRPX	SRPX	Sushi repe	3	268.2744	51.45038	9.3E-62	3	7.112069
Q9Y5M8	271	29701.9	9.47	Signal recc	SRPRB	SRPRB	Signal recc	8	414.2285	50.12577	9.6E-57	8	38.00738
P08240	638	69810.6	9.46	Signal recc	SRPRA	SRPRA	Signal recc	7	164.5208	48.23734	6.32E-50	7	13.47962
P78362	688	77526	4.61	SRSF prote	SRPK2	SRPK2	SRSF prote	13	1893.866	50.48594	4.37E-58	10	21.65698
Q96SB4	655	74324.3	6.09	SRSF prote	SRPK1	SRPK1	SRSF prote	12	1953.92	52.47063	1.02E-65	12	22.44275
P49458	86	10111.7	8.21	Signal recc	SRP9	SRP9	Signal recc	4	243.0976	54.26814	6.86E-73	4	45.34884
O76094	671	74605.6	9.8	Signal recc	SRP72	SRP72	Signal recc	25	2425.906	50.56618	2.17E-58	25	40.68554
Q9UHB9	627	70729	8.8	Signal recc	SRP68	SRP68	Signal recc	26	584.6394	51.69696	1.05E-62	26	46.0925
P61011	504	55704.4	9.19	Signal recc	SRP54	SRP54	Signal recc	3	158.5281	46.5811	2.53E-44	3	5.555556
P09132	144	16155.6	10.47	Signal recc	SRP19	SRP19	Signal recc	4	135.5807	49.92429	5.4E-56	4	45.83333
P37108	136	14569.8	10.75	Signal recc	SRP14	SRP14	Signal recc	7	413.0376	50.59458	1.7E-58	7	41.91176
P19623	302	33824.5	5.17	Spermidin	SRM	SRM	Spermidin	3	440.5388	45.78137	9.48E-42	3	10.2649
P30626	198	21676.2	5.21	Sorcin	SRI	SRI	Sorcin	2	174.386	31.46736	3.98E-11	2	11.61616
O75044	1071	120869.5	6.68	SLIT-ROBC	SRGAP2	SRGAP2	SLIT-ROBC	44	9967.818	51.19731	8.68E-61	24	45.845
Q7Z6B7	1085	124263.2	6.81	SLIT-ROBC	SRGAP1	SRGAP1	SLIT-ROBC	4	88.98981	38.50988	1.65E-22	2	4.700461
Q8N9Q2	155	18176.6	10.59	Protein SR	SREK1IP1	SREK1IP1	Protein SR	1	86.9791	42.39615	7.57E-32	1	7.741935
Q8WXA9	508	59379.8	11.14	Splicing re	SREK1	SREK1	Splicing re	7	383.2738	48.32377	3.15E-50	7	20.66929
Q6ZRS2	3230	343550.9	5.8	Helicase SI	SRCAP	SRCAP	Helicase SI	7	199.1227	43.68773	1.98E-35	7	2.167183
P12931	536	59834.3	7.47	Proto-onc	SRC	SRC	Proto-onc	1	68.79121	42.46376	4.98E-32	1	2.61194
Q8N5C6	995	111774.7	9	S1 RNA-bi	SRBD1	SRBD1	S1 RNA-bi	30	712.9996	49.87657	8.1E-56	30	37.58794
Q13501	440	47686.7	4.88	Sequestos	SQSTM1	SQSTM1	Sequestos	4	173.0153	53.30936	4.98E-69	4	14.77273
Q9Y6N5	450	49960.3	9.51	Sulfide:qui	SQOR	SQOR	Sulfide:qui	11	476.503	52.96608	1.17E-67	11	31.33333
Q14534	574	63922.5	8.87	Squalene r	SQLE	SQLE	Squalene r	2	44.18487	36.12244	7.48E-18	2	4.703833
O15270	562	62923.8	7.85	Serine palr	SPTLC2	SPTLC2	Serine palr	4	119.8135	51.87744	2.13E-63	4	9.964413
O15269	473	52743.4	5.87	Serine palr	SPTLC1	SPTLC1	Serine palr	5	237.9043	53.29851	5.5E-69	5	12.26216
O15020	2390	271322.5	6.01	Spectrin b	SPTBN2	SPTBN2	Spectrin b	9	88.07638	43.53171	5.52E-35	9	5.313808
Q01082	2364	274607	5.26	Spectrin b	SPTBN1	SPTBN1	Spectrin b	76	1634.016	56.96886	5.54E-84	69	38.91709
Q13813	2472	284537	5.01	Spectrin al	SPTAN1	SPTAN1	Spectrin al	102	1812.496	52.4276	1.51E-65	102	50
Q5W111	196	21665.7	6.7	SPRY dom	SPRYD7	SPRYD7	SPRY dom	2	140.7801	49.34545	7.13E-54	2	9.693878
Q9C004	299	32541	7.92	Protein spi	SPRY4	SPRY4	Protein spi	2	221.4191	43.8968	4.94E-36	2	11.03679
P35270	261	28048.1	8.19	Sepiapterii	SPR	SPR	Sepiapterii	2	74.60306	26.95404	1.74E-07	2	9.578544
Q8TCT8	520	58142.6	8.4	Signal pep	SPPL2A	SPPL2A	Signal pep	2	165.6389	44.99176	2.71E-39	2	4.423077
Q5T280	376	42008.4	7.48	Putative m	SPOUT1	SPOUT1	Putative m	10	774.7509	51.14529	1.37E-60	10	33.24468
Q08629	439	49123.8	5.98	Testican-1	SPOCK1	SPOCK1	Testican-1	1	110.4755	26.49872	2.9E-07	1	2.050114
O43291	252	28227.9	8.36	Kunitz-typ	SPINT2	SPINT2	Kunitz-typ	1	172.4706	28.45069	2.15E-08	1	4.761905
O43278	529	58397.5	6.23	Kunitz-typ	SPINT1	SPINT1	Kunitz-typ	6	308.5756	51.22935	6.54E-61	6	11.90926
Q56A73	249	28659.7	7.82	Spindlin-4	SPIN4	SPIN4	Spindlin-4	1	385.5215	46.51724	4.09E-44	1	4.016064
Q99865	258	29188	7.27	Spindlin-2	SPIN2A	SPIN2A;SP	Spindlin-2	1	119.7726	38.48373	1.88E-22	1	4.651163
Q9Y657	262	29600.4	6.96	Spindlin-1	SPIN1	SPIN1	Spindlin-1	6	1086.862	44.63987	3.16E-38	6	29.00763
Q9UQ90	795	88234	9.07	Mitochonc	SPG7	SPG7	Mitochonc	4	137.2123	43.21568	4.3E-34	4	7.295597
Q96T58	3664	402244.7	7.69	Msx2-inter	SPEN	SPEN	Msx2-inter	32	1177.35	49.87183	8.41E-56	32	9.224891
Q69YQ0	1117	124542.7	5.49	Cytospin-/	SPECC1L	SPECC1L	Cytospin-/	21	647.3801	47.85971	1.28E-48	21	22.02328
Q5M775	1068	118583.9	6.68	Cytospin-E	SPECC1	SPECC1	Cytospin-E	12	256.2039	53.04364	5.69E-68	12	14.32584
P61009	180	20313.2	8.99	Signal pep	SPCS3	SPCS3	Signal pep	3	210.9912	53.73907	9.78E-71	3	17.77778
Q15005	226	25002.5	8.68	Signal pep	SPCS2	SPCS2	Signal pep	4	262.8748	50.17766	6.15E-57	4	20.35398
Q9Y6A9	169	18298	8.92	Signal pep	SPCS1	SPCS1	Signal pep	1	84.87432	41.89137	1.64E-30	1	7.100592
Q9NUQ6	558	61728.6	10.36	SPATS2-lik	SPATS2L	SPATS2L	SPATS2-lik	8	142.5032	54.03842	5.79E-72	8	17.56272
Q86XZ4	545	59544.2	9.35	Spermatog	SPATS2	SPATS2	Spermatog	13	298.7166	50.48072	4.56E-58	13	26.78899
Q8TB22	786	87898	7.46	Spermatog	SPATA20	SPATA20	Spermatog	4	332.1151	45.15178	8.77E-40	4	6.361323
Q8IZ16	206	23861.8	11.12	Sperm acr	SPACDR	SPACDR	Sperm acr	1	99.30084	37.93467	2.6E-21	1	4.854369
Q02086	613	64899.7	10.62	Transcripti	SP2	SP2	Transcripti	1	46.00664	27.61756	7.45E-08	1	2.446982
Q9H930	580	67004.8	8.61	Nuclear bc	SP140L	SP140L	Nuclear bc	9	236.4821	55.33042	2.73E-77	3	16.72414
P23497	879	100415.9	8.36	Nuclear au	SP100	SP100	Nuclear au	9	220.6693	52.65081	2.02E-66	9	9.442548
P08047	785	80692.7	7.36	Transcripti	SP1	SP1	Transcripti	2	104.1125	40.55565	3.74E-27	2	2.165605
Q00796	357	38324.2	8.06	Sorbitol de	SORD	SORD	Sorbitol de	2	250.7013	39.97033	9.2E-26	2	8.963585
O60504	671	75340.5	9.87	Vinexin	SORBS3	SORBS3	Vinexin	6	239.1768	46.07251	1.12E-42	6	10.58122
Q9BX66	1292	142511.3	6.83	Sorbin anc	SORBS1	SORBS1	Sorbin anc	3	116.0103	41.53061	1.41E-29	3	3.250774
P18583	2426	263827.3	5.35	Protein SO	SON	SON	Protein SO	23	2341.752	52.58827	3.57E-66	23	11.04699
Q8WXH5	440	50622.6	7.07	Suppresso	SOCS4	SOCS4	Suppresso	1	233.5139	24.09902	1.95E-06	1	2.5
P35610	550	64734	9.18	Sterol O-a	SOAT1	SOAT1	Sterol O-a	1	103.5688	39.40037	1.87E-24	1	1.272727
Q9Y5X1	595	66591.2	5.26	Sorting ne:	SNX9	SNX9	Sorting ne:	13	1472.418	51.88889	1.92E-63	13	25.88235
Q9UNH7	406	46648.3	6.05	Sorting ne:	SNX6	SNX6	Sorting ne:	2	58.48705	34.97478	6.6E-16	2	4.926108
Q9Y5X3	404	46816.1	6.75	Sorting ne:	SNX5	SNX5	Sorting ne:	3	158.122	51.27323	4.46E-61	3	9.653465
Q96L92	541	61264.9	6.47	Sorting ne:	SNX27	SNX27	Sorting ne:	6	519.3644	53.33322	4E-69	6	12.19963
Q9Y343	169	19817.7	7.27	Sorting ne:	SNX24	SNX24	Sorting ne:	1	80.21558	33.49934	1.11E-13	1	5.325444
O60749	519	58470.5	4.77	Sorting ne:	SNX2	SNX2	Sorting ne:	3	157.997	49.72371	2.94E-55	2	6.743738
Q96RF0	628	68893.9	5.4	Sorting ne:	SNX18	SNX18	Sorting ne:	5	230.4891	54.61259	2.68E-74	5	10.98726
Q13596	522	59069	4.8	Sorting ne:	SNX1	SNX1	Sorting ne:	4	134.3312	42.33412	1.11E-31	4	9.770115
Q13573	536	61494	10.12	SNW dom.	SNW1	SNW1	SNW dom.	13	1401.759	54.0356	5.91E-72	13	28.54478
P55769	128	14173.4	8.64	NHP2-like	SNU13	SNU13	NHP2-like	7	870.8671	49.42407	3.7E-54	7	46.875
Q13425	540	57949	9.07	Beta-2-syr	SNTB2	SNTB2	Beta-2-syr	10	815.5093	49.05756	7.79E-53	10	22.77778
Q13884	538	58060.6	8.91	Beta-1-syr	SNTB1	SNTB1	Beta-1-syr	4	53.13734	43.18146	5.36E-34	4	9.665428
A8MWD9	76	8544	9.38	Putative sr	SNRPGP15	SNRPGP15	Putative sr	2	919.2198	48.60168	3.31E-51	2	17.10526
P62306	86	9725.2	4.33	Small nucl	SNRPF	SNRPF	Small nucl	5	2773.868	46.04588	1.37E-42	5	50
P62304	92	10803.6	9.86	Small nucl	SNRPE	SNRPE	Small nucl	3	5146.293	49.41444	4E-54	3	38.04348
P62318	126	13916.2	10.98	Small nucl	SNRPD3	SNRPD3	Small nucl	4	17611.7	49.54837	1.3E-54	4	47.61905
P62316	118	13526.8	10.66	Small nucl	SNRPD2	SNRPD2	Small nucl	5	10648.72	47.72938	3.59E-48	5	39.83051
P62314	119	13281.5	12.09	Small nucl	SNRPD1	SNRPD1	Small nucl	5	7783.636	49.24563	1.64E-53	5	54.62185
P09234	159	17393.6	10.14	U1 small n	SNRPC	SNRPC	U1 small n	2	517.0433	50.37353	1.15E-57	2	18.86792
P08579	225	25486.2	10.34	U2 small n	SNRPB2	SNRPB2	U2 small n	4	370.0228	51.37215	1.87E-61	4	25.77778
P14678	240	24609.9	11.75	Small nucl	SNRPB	SNRPB;SNI	Small nucl	8	6817.664	51.2434	5.79E-61	8	23.33333
P09661	255	28415.3	9	U2 small n	SNRPA1	SNRPA1	U2 small n	9	1656.728	50.37996	1.09E-57	9	34.11765
P09012	282	31279.4	10.5	U1 small n	SNRPA	SNRPA	U1 small n	3	3690.885	39.082	9.56E-24	3	12.76596
P08621	437	51556.2	10.47	U1 small n	SNRNP70	SNRNP70	U1 small n	16	27294.2	49.89438	6.96E-56	16	35.69794
Q6IEG0	339	39965.1	6.95	U11/U12 s	SNRNP48	SNRNP48	U11/U12 s	8	372.6258	50.49826	3.93E-58	8	29.20354
Q96DI7	357	39310.2	8.21	U5 small n	SNRNP40	SNRNP40	U5 small n	16	3874.425	51.89644	1.81E-63	16	56.30252
Q16560	246	29450	10.45	U11/U12 s	SNRNP35	SNRNP35	U11/U12 s	2	175.3511	41.67788	5.89E-30	2	9.349593
Q8WVK2	155	18860	12.13	U4/U6.U5	SNRNP27	SNRNP27	U4/U6.U5	3	594.2201	47.71442	4.04E-48	3	17.41935
Q9BV90	132	15269.7	8.2	U11/U12 s	SNRNP25	SNRNP25	U11/U12 s	2	62.84345	45.0432	1.88E-39	2	12.12121
O75643	2136	244505.5	5.95	U5 small n	SNRNP200	SNRNP200	U5 small n	95	9392.754	54.54571	5.05E-74	95	49.20412
Q8TAD8	396	45777.2	10.59	Smad nucl	SNIP1	SNIP1	Smad nucl	8	1507.802	49.77802	1.86E-55	8	22.9798

Q7KZF4	910	101996.1	7.17	Staphylocc	SND1	SND1	Staphylocc	29	2168.33	51.01181	4.42E-60	29	36.59341
Q5SXM2	1469	159431.3	8.42	snRNA-act	SNAPC4	SNAPC4	snRNA-act	1	176.2483	49.32253	8.66E-54	1	0.748809
O00161	211	23353.9	4.62	Synaptoso	SNAP23	SNAP23	Synaptoso	4	213.5312	47.58812	1.09E-47	4	26.06635
Q9H7B4	428	49096.7	7.27	Histone-ly	SMYD3	SMYD3	Histone-ly	2	117.0592	46.94962	1.54E-45	2	4.906542
Q9HAU4	748	86195.2	8.02	E3 ubiquiti	SMURF2	SMURF2	E3 ubiquiti	21	915.0606	51.74432	6.92E-63	15	29.94652
Q9HCE7	757	86113	7.02	E3 ubiquiti	SMURF1	SMURF1	E3 ubiquiti	2	440.4083	53.23823	9.53E-69	2	2.642008
Q2TAY7	513	57543.4	7.19	WD40 repe	SMU1	SMU1	WD40 repe	15	3183.092	49.62848	6.57E-55	15	38.79142
Q92485	455	50813.4	5.38	Acid sphin	SMPDL3B	SMPDL3B	Acid sphin	5	676.0194	52.53595	5.71E-66	5	17.36264
Q9NXE4	866	97809	8.35	Sphingom	SMPD4	SMPD4	Sphingom	2	60.09153	35.613	5.76E-17	2	3.002309
O75940	238	26711	7.34	Survival of	SMNDC1	SMNDC1	Survival of	2	533.8841	47.2929	1.09E-46	2	9.243697
Q16637	294	31848.4	6.51	Survival m	SMN1	SMN1	Survival m	4	398.1443	54.85728	2.53E-75	4	14.96599
Q8N5G0	67	7701.9	10.35	Small integ	SMIM20	SMIM20	Small integ	1	266.0022	51.36173	2.05E-61	1	20.89552
Q8ND04	991	109682.7	7.72	Nonsense-	SMG8	SMG8	Nonsense-	1	66.84176	35.26382	2.22E-16	1	1.10999
Q9H4I9	107	11441	7.54	Essential M	SMDT1	SMDT1	Essential M	1	83.2402	40.58589	3.17E-27	1	9.345794
A6NHR9	2005	226371.7	7.32	Structural	SMCHD1	SMCHD1	Structural	53	1240.417	52.52196	6.46E-66	53	32.21945
Q96SB8	1091	126324.5	6.99	Structural	SMC6	SMC6	Structural	10	139.937	53.50795	8.08E-70	10	10.17415
Q8IY18	1101	128805.6	8.54	Structural	SMC5	SMC5	Structural	10	449.6407	52.52866	6.09E-66	10	8.991826
Q9UQE7	1217	141540.7	7.18	Structural	SMC3	SMC3	Structural	17	395.9607	50.0498	1.84E-56	17	16.43385
O95347	1197	135655.1	8.78	Structural	SMC2	SMC2	Structural	4	96.38787	38.22858	6.45E-22	4	4.845447
Q14683	1233	143231.9	7.75	Structural	SMC1A	SMC1A	Structural	18	663.6289	50.47042	4.97E-58	18	15.65288
Q969G3	411	46649.1	4.54	SWI/SNF-r	SMARCE1	SMARCE1	SWI/SNF-r	3	147.8486	51.0621	2.84E-60	3	10.21898
Q92925	531	58920.5	10.22	SWI/SNF-r	SMARCD2	SMARCD2	SWI/SNF-r	3	197.7832	50.53202	2.93E-58	3	7.156309
Q96GM5	515	58232.1	9.72	SWI/SNF-r	SMARCD1	SMARCD1	SWI/SNF-r	1	341.5677	45.27922	3.55E-40	1	2.135922
Q8TAQ2	1214	132878.5	5.45	SWI/SNF c	SMARCC2	SMARCC2	SWI/SNF c	5	148.5995	49.54078	1.38E-54	5	5.107084
Q92922	1105	122866.3	5.53	SWI/SNF c	SMARCC1	SMARCC1	SWI/SNF c	11	748.659	50.99201	5.26E-60	5	11.22172
Q12824	385	44140.8	6.13	SWI/SNF-r	SMARCB1	SMARCB1	SWI/SNF-r	4	465.7561	51.10865	1.89E-60	4	12.20779
Q9NZC9	954	105936.9	9.55	SWI/SNF-r	SMARCAL1	SMARCAL1	SWI/SNF-r	5	102.5575	50.39653	9.41E-58	5	5.765199
Q9H4L7	1026	117401.3	5.24	SWI/SNF-r	SMARCAD	SMARCAD	SWI/SNF-r	2	70.24848	48.06277	2.55E-49	2	2.14425
O60264	1052	121904.3	8.25	SWI/SNF-r	SMARCA5	SMARCA5	SWI/SNF-r	59	7141.549	51.29524	3.67E-61	40	48.95437
P51532	1647	184644.3	8.02	Transcripti	SMARCA4	SMARCA4	Transcripti	10	431.3742	52.08869	3.21E-64	10	7.650273
P51531	1590	181278.2	7.2	Probable g	SMARCA2	SMARCA2	Probable g	19	220.4239	51.40475	1.4E-61	7	14.33962
P28370	1054	122603.6	8.25	Probable g	SMARCA1	SMARCA1	Probable g	33	1243.837	55.38983	1.64E-77	33	29.79127
P84022	425	48080.4	7.16	Mothers ag	SMAD3	SMAD3	Mothers ag	3	296.8892	50.33892	1.54E-57	3	7.529412
Q15796	467	52305.8	6.56	Mothers ag	SMAD2	SMAD2	Mothers ag	5	327.1204	51.13726	1.47E-60	1	12.63383
Q15797	465	52259.7	7.33	Mothers ag	SMAD1	SMAD1;SM	Mothers ag	1	90.44743	32.49258	2.42E-12	1	2.795699
Q9NSI2	230	25456	11.65	Ribosome	SLX9	SLX9	Ribosome	3	74.04996	49.12296	4.54E-53	3	16.08696
O95391	586	68386.3	7.14	Pre-mRNA	SLU7	SLU7	Pre-mRNA	6	325.4489	50.06125	1.67E-56	6	13.13993
Q9NWH9	1034	117147.4	8.05	SAFB-like t	SLTM	SLTM	SAFB-like t	23	1282.781	52.47244	1.01E-65	23	23.98453
Q9H2G2	1235	142694	4.8	STE20-like	SLK	SLK	STE20-like	2	175.9211	49.68476	4.09E-55	2	1.700405
O75093	1534	167924.4	6.54	Slit homol	SLIT1	SLIT1	Slit homol	1	44.6697	32.1508	6.39E-12	1	0.521512
Q9GZT3	109	12348.9	11	SRA stem-	SLIRP	SLIRP	SRA stem-	1	158.8251	45.88661	4.4E-42	1	12.84404
Q08AF3	891	101053.8	8.29	Schlafen fa	SLFN5	SLFN5	Schlafen fa	8	570.493	53.69437	1.47E-70	7	8.754209
Q96BD0	722	77192.5	7.85	Solute carr	SLCO4A1	SLCO4A1	Solute carr	1	297.4909	37.79998	4.87E-21	1	1.800554
Q01650	507	55009.6	7.79	Large neut	SLC7A5	SLC7A5	Large neut	2	269.224	49.30247	1.02E-53	2	6.311637
Q9Y6M7	1214	136042.6	6.7	Sodium bi	SLC4A7	SLC4A7	Sodium bi	24	3734.564	53.31464	4.76E-69	21	22.07578
Q9BWU0	742	82889.8	4.75	Kanadapti	SLC4A1AP	SLC4A1AP	Kanadapti	1	97.74777	43.95179	3.42E-36	1	1.212938
Q6U841	1118	125945.1	6.47	Sodium-dr	SLC4A10	SLC4A10	Sodium-dr	1	308.0051	47.74477	3.19E-48	1	0.894454
Q8IWA5	706	80122.7	8.66	Choline tra	SLC44A2	SLC44A2	Choline tra	2	74.631	37.2692	5.5E-20	2	2.832861
Q8WWI5	657	73301.3	8.69	Choline tra	SLC44A1	SLC44A1	Choline tra	1	170.286	33.3499	1.78E-13	1	2.130898
P08195	630	67993.3	4.63	Amino acid	SLC3A2	SLC3A2	Amino acid	20	1073.826	52.2354	8.52E-65	20	29.68254
Q13433	755	85046.2	6.95	Zinc transp	SLC39A6	SLC39A6	Zinc transp	4	317.1405	54.50759	7.3E-74	4	5.960265
Q8N1S5	342	35395.7	5.31	Zinc transp	SLC39A11	SLC39A11	Zinc transp	2	292.9431	41.54395	1.3E-29	2	11.40351
Q9ULF5	831	94131.5	6.75	Zinc transp	SLC39A10	SLC39A10	Zinc transp	8	695.5048	52.34264	3.26E-65	8	10.34898
Q96QD8	506	56025.4	8.12	Sodium-co	SLC38A2	SLC38A2	Sodium-co	1	51.9358	47.7146	4.03E-48	1	3.162055
Q9HBR0	1119	119761.5	5.48	Solute carr	SLC38A10	SLC38A10	Solute carr	1	169.4247	47.32932	8.24E-47	1	1.340483
Q8WV83	523	58886.5	9.67	Solute carr	SLC35F5	SLC35F5	Solute carr	1	268.3868	26.06998	4.46E-07	1	2.10325
Q96K37	410	44772.1	10.17	Solute carr	SLC35E1	SLC35E1	Solute carr	4	48.44608	50.87152	1.52E-59	4	14.14634
Q8TB61	432	47514.3	9.4	Adenosine	SLC35B2	SLC35B2	Adenosine	4	164.5939	52.8911	2.3E-67	4	9.953704
O00400	549	60908.4	7.37	Acetyl-coe	SLC33A1	SLC33A1	Acetyl-coe	1	23.65497	40.51437	4.71E-27	1	2.367942
Q8NEW0	376	41625.6	6.94	Zinc transp	SLC30A7	SLC30A7	Zinc transp	2	171.4445	42.57914	2.43E-32	2	6.648936
P11169	496	53923.8	7.24	Solute carr	SLC2A3	SLC2A3;SL	Solute carr	3	79.46508	43.679	2.09E-35	3	8.064516
P11166	492	54083.3	8.91	Solute carr	SLC2A1	SLC2A1	Solute carr	4	780.4661	46.51991	4.01E-44	4	10.36585
Q9Y2P4	619	70111	8.66	Long-chai	SLC27A6	SLC27A6	Long-chai	5	125.4112	48.21658	7.45E-50	5	9.854604
Q6P1M0	643	72063.6	8.58	Long-chai	SLC27A4	SLC27A4	Long-chai	4	392.2303	50.68298	7.85E-59	4	7.77605
O14975	620	70311.7	8.65	Long-chai	SLC27A2	SLC27A2	Long-chai	3	75.0034	49.14338	3.84E-53	3	6.774194
Q6PCB7	646	71107.5	8.63	Long-chai	SLC27A1	SLC27A1	Long-chai	3	192.0064	31.47942	3.86E-11	3	5.108359
P12236	298	32866	10.26	ADP/ATP t	SLC25A6	SLC25A6	ADP/ATP t	3	219.1879	53.60715	3.29E-70	3	12.41611
P05141	298	32852	10.18	ADP/ATP t	SLC25A5	SLC25A5	ADP/ATP t	13	6597.481	51.20225	8.32E-61	7	35.57047
P12235	298	33064.3	10.27	ADP/ATP t	SLC25A4	SLC25A4	ADP/ATP t	7	1016.952	48.73488	1.12E-51	4	22.81879
Q00325	362	40094.5	9.79	Solute carr	SLC25A3	SLC25A3	Solute carr	8	2475.092	48.01409	3.76E-49	8	22.37569
Q8N8R3	303	32061.9	8.89	Mitochonc	SLC25A29	SLC25A29	Mitochonc	2	133.1748	46.47513	5.61E-44	2	8.580858
Q6NUK1	477	53354.1	6.21	Mitochonc	SLC25A24	SLC25A24	Mitochonc	3	192.4939	46.49542	4.82E-44	3	6.918239
Q9H936	323	34469.8	9.67	Mitochonc	SLC25A22	SLC25A22	Mitochonc	6	356.5663	50.29918	2.16E-57	4	24.4582
O43772	301	32943.5	9.84	Mitochonc	SLC25A20	SLC25A20	Mitochonc	1	86.53343	23.86075	2.24E-06	1	2.990033
Q9HC21	320	35510.9	9.99	Mitochonc	SLC25A19	SLC25A19	Mitochonc	1	262.7802	45.95799	2.61E-42	1	4.6875
Q9UJS0	675	74174.9	8.87	Electrogen	SLC25A13	SLC25A13	Electrogen	3	146.5444	38.89808	2.42E-23	3	4.888889
O75746	678	74761.2	8.56	Electrogen	SLC25A12	SLC25A12	Electrogen	12	370.9484	51.1045	1.96E-60	7	19.46903
Q02978	314	34061.4	10.4	Mitochonc	SLC25A11	SLC25A11	Mitochonc	10	643.6682	49.72219	2.98E-55	10	40.76433
Q9UBX3	287	31282.2	9.91	Mitochonc	SLC25A10	SLC25A10	Mitochonc	6	469.2824	51.81634	3.67E-63	6	26.82927
P53007	311	34012.5	10.44	Tricarboxy	SLC25A1	SLC25A1	Tricarboxy	8	1551.37	50.45303	5.78E-58	8	30.54662
Q96BI1	424	44845	9.93	Solute carr	SLC22A18	SLC22A18	Solute carr	2	345.6381	49.18263	2.77E-53	2	5.660377
Q8WUM9	679	73699	7.08	Sodium-de	SLC20A1	SLC20A1	Sodium-de	1	189.9467	42.18541	2.76E-31	1	1.472754
Q15758	541	56597.6	5.14	Neutral am	SLC1A5	SLC1A5	Neutral am	6	572.757	48.79564	6.76E-52	6	12.01479
O15427	465	49468.9	8.03	Monocarbo	SLC16A3	SLC16A3	Monocarbo	4	232.318	53.36623	2.98E-69	4	10.75269
P53985	500	53943.7	8.82	Monocarbo	SLC16A1	SLC16A1	Monocarbo	5	219.8355	50.2012	5.02E-57	5	13
Q9UP95	1085	120648.7	6.41	Solute carr	SLC12A4	SLC12A4	Solute carr	1	75.17669	31.0012	1.27E-10	1	1.198157
P55011	1212	131445.8	6.36	Solute carr	SLC12A2	SLC12A2	Solute carr	9	200.3176	48.40118	1.68E-50	9	12.0462
Q14493	270	31285.4	7.54	Histone RN	SLBP	SLBP	Histone RN	4	306.7063	51.79868	4.29E-63	4	13.7037
P63208	163	18657.9	4.15	S-phase ki	SKP1	SKP1	S-phase ki	5	313.0814	51.47306	7.59E-62	5	32.51534
Q9GZS3	305	33580.3	5.12	Superkiller	SKIC8	SKIC8	Superkiller	7	563.4026	51.50331	5.8E-62	7	25.57377
Q6PGP7	1564	175484.6	7.57	Superkiller	SKIC3	SKIC3	Superkiller	3	77.16519	31.06107	1.1E-10	3	1.982097
P12755	728	80004.1	7.67	Ski oncoge	SKI	SKI	Ski oncoge	1	92.41476	29.25164	5.35E-09	1	2.335165
Q9NRC8	400	44897.9	10.3	NAD-depe	SIRT7	SIRT7	NAD-depe	7	216.5372	48.37585	2.06E-50	7	24.25

Q8N6T7	355	39118.5	9.67	NAD-depe	SIRT6	SIRT6	NAD-depe	15	4211.658	52.14019	2.04E-64	15	47.04225
Q9H106	197	21686.8	9.87	Signal-reg	SIRPD	SIRPD	Signal-reg	1	365.5851	24.22814	1.81E-06	1	9.64467
O60292	1781	194608.1	8.45	Signal-ind	SIPA1L3	SIPA1L3	Signal-ind	6	125.6997	48.49711	7.75E-51	5	3.705783
O43166	1804	200026.6	8.31	Signal-ind	SIPA1L1	SIPA1L1	Signal-ind	4	146.1358	45.37225	1.83E-40	4	2.605322
Q96FS4	1042	112147.9	6.58	Signal-ind	SIPA1	SIPA1	Signal-ind	4	75.94242	34.90592	8.51E-16	4	5.566219
Q9NP50	221	24852.1	9.96	SIN3-HDA	SINHCAF	SINHCAF	SIN3-HDA	5	90.99867	48.87919	3.39E-52	5	23.07692
O75182	1162	133065.4	6.92	Paired amp	SIN3B	SIN3B	Paired amp	5	128.325	52.74959	8.39E-67	5	4.216867
Q96ST3	1273	145174.2	7.27	Paired amp	SIN3A	SIN3A	Paired amp	9	317.5138	46.45557	6.5E-44	9	7.069914
Q99720	223	25127.5	5.87	Sigma non	SIGMAR1	SIGMAR1	Sigma non	1	114.1993	38.25293	5.74E-22	1	5.829596
A0MZ66	631	71639.3	5.01	Shootin-1	SHTN1	SHTN1	Shootin-1	4	117.501	52.16074	1.69E-64	4	7.448494
Q6PI26	577	65124.5	4.42	Protein SH	SHQ1	SHQ1	Protein SH	3	98.80379	51.05196	3.1E-60	3	7.62565
Q9UQ13	582	64887.3	8.68	Leucine-ric	SHOC2	SHOC2	Leucine-ric	4	116.1577	46.1852	4.9E-43	4	10.13746
P34897	504	55992.4	8.67	Serine hyd	SHMT2	SHMT2	Serine hyd	9	452.7309	52.20546	1.12E-64	9	24.20635
A1X283	911	101578.3	9.06	SH3 and P.	SH3PXD2B	SH3PXD2B	SH3 and P.	2	113.0972	38.9876	1.54E-23	2	2.854007
Q9NR46	395	43973.4	5.78	Endophilin	SH3GLB2	SH3GLB2	Endophilin	1	88.1931	28.39525	2.35E-08	1	3.291139
Q99961	368	41489.6	5.08	Endophilin	SH3GL1	SH3GL1	Endophilin	4	266.1621	51.8743	2.18E-63	3	15.76087
Q7L8J4	393	43498.9	5.55	SH3 doma	SH3BP5L	SH3BP5L	SH3 doma	1	45.72889	19.41736	1.4E-05	1	2.035623
Q9P0V3	963	107494.9	7.77	SH3 doma	SH3BP4	SH3BP4	SH3 doma	5	234.9578	48.9389	2.07E-52	5	6.542056
Q96EQ0	304	33429.1	4.54	Small gluta	SGTB	SGTB	Small gluta	11	12081.6	49.2735	1.3E-53	11	33.55263
O43765	313	34062.8	4.51	Small gluta	SGTA	SGTA	Small gluta	2	48.41948	44.09245	1.33E-36	2	7.98722
O95470	568	63523.3	9.57	Sphingosir	SGPL1	SGPL1	Sphingosir	6	323.8161	52.52092	6.52E-66	6	13.90845
Q6P4A7	337	37998	9.55	Sideroflexi	SFXN4	SFXN4	Sideroflexi	2	152.6994	55.547	3.7E-78	2	7.121662
Q9BWM7	321	35503.1	9.38	Sideroflexi	SFXN3	SFXN3	Sideroflexi	6	457.534	50.84529	1.9E-59	6	23.67601
Q9H9B4	322	35619.1	9.41	Sideroflexi	SFXN1	SFXN1	Sideroflexi	7	679.5264	51.79922	4.27E-63	7	27.01863
Q12872	951	104820.4	8.22	Splicing fa	SFSWAP	SFSWAP	Splicing fa	15	698.6991	48.89075	3.09E-52	15	16.50894
P23246	707	76149.2	9.95	Splicing fa	SFPQ	SFPQ	Splicing fa	25	13350.28	50.81456	2.5E-59	25	47.24187
P31947	248	27773.8	4.39	14-3-3 pro	SFN	SFN	14-3-3 pro	7	553.1642	48.48315	8.68E-51	7	24.19355
Q9UHJ3	866	98140.6	6.12	Scm-like w	SFMBT1	SFMBT1	Scm-like w	2	135.8755	21.05274	7.8E-06	2	2.655889
Q9Y3B4	125	14584.8	9.76	Splicing fa	SF3B6	SF3B6	Splicing fa	5	1202.231	53.73198	1.05E-70	5	48
Q9BWJ5	86	10135.3	6.34	Splicing fa	SF3B5	SF3B5	Splicing fa	2	258.912	51.36978	1.91E-61	2	27.90698
Q15427	424	44385.5	8.86	Splicing fa	SF3B4	SF3B4	Splicing fa	7	789.7019	47.8382	1.52E-48	7	24.5283
Q15393	1217	135576.2	4.91	Splicing fa	SF3B3	SF3B3	Splicing fa	37	11317.56	53.47272	1.1E-69	37	36.23665
Q13435	895	100226.9	5.37	Splicing fa	SF3B2	SF3B2	Splicing fa	33	4920.825	53.89356	2.28E-71	33	39.77654
O75533	1304	145829.1	7.1	Splicing fa	SF3B1	SF3B1	Splicing fa	49	9565.599	54.97102	8.52E-76	49	46.31902
Q12874	501	58848.4	5.05	Splicing fa	SF3A3	SF3A3	Splicing fa	9	1009.833	52.31848	4.05E-65	9	22.35529
Q15428	464	49255.4	10.21	Splicing fa	SF3A2	SF3A2	Splicing fa	9	558.3293	49.56098	1.17E-54	9	23.92241
Q15459	793	88885.6	4.88	Splicing fa	SF3A1	SF3A1	Splicing fa	25	2313.522	52.06128	4.13E-64	25	36.44388
Q15637	639	68329.5	9.41	Splicing fa	SF1	SF1	Splicing fa	8	1332.519	49.76161	2.14E-55	8	17.84038
Q7Z333	2677	302877.2	7.17	Probable h	SETX	SETX	Probable h	12	216.7769	49.78585	1.75E-55	12	4.856182
Q53H47	684	78033.3	7.15	Histone-ly	SETMAR	SETMAR	Histone-ly	1	139.2613	40.76381	1.17E-27	1	1.754386
Q15047	1291	143155.6	5.84	Histone-ly	SETDB1	SETDB1	Histone-ly	1	26.55388	30.31885	6.13E-10	1	1.936483
Q8WTS6	366	40720.6	4.25	Histone-ly	SETD7	SETD7	Histone-ly	2	101.6016	33.20148	2.85E-13	2	6.284153
Q9BYW2	2564	287594.2	6.02	Histone-ly	SETD2	SETD2	Histone-ly	2	82.16478	46.67999	1.2E-44	2	1.131045
Q9UPS6	1966	212800.7	4.59	Histone-ly	SETD1B	SETD1B	Histone-ly	1	165.2744	34.77667	1.37E-15	1	0.457782
O15047	1707	186032.2	4.79	Histone-ly	SETD1A	SETD1A	Histone-ly	21	658.5839	52.74821	8.47E-67	20	17.57469
Q01105	290	33488.6	3.95	Protein SE	SET	SET	Protein SE	5	2771.575	48.55483	4.85E-51	2	15.86207
P50454	418	46440.1	9.12	Serpin H1	SERPINH1	SERPINH1	Serpin H1	10	408.5998	49.99099	3.05E-56	10	33.01435
P07093	398	44001.8	9.9	Glia-derive	SERPINE2	SERPINE2	Glia-derive	4	160.1334	53.09313	3.6E-68	4	12.56281
P50453	376	42403.2	5.67	Serpin B9	SERPINB9	SERPINB9	Serpin B9	10	1498.915	49.1381	4.01E-53	9	28.98936
P35237	376	42621.6	4.93	Serpin B6	SERPINB6	SERPINB6	Serpin B6	6	249.1219	48.91886	2.44E-52	5	18.35106
P36952	375	42099.9	5.91	Serpin B5	SERPINB5	SERPINB5	Serpin B5	3	265.6063	48.70986	1.37E-51	3	9.6
P29508	390	44564.3	6.81	Serpin B3	SERPINB3	SERPINB3	Serpin B3	1	271.1388	44.33873	2.49E-37	1	2.564103
Q96P63	405	46276	5.22	Serpin B12	SERPINB12	SERPINB12	Serpin B12	2	370.7869	52.3755	2.42E-65	2	5.925926
P30740	379	42741.4	6.19	Leukocyte	SERPINB1	SERPINB1	Leukocyte	10	1024.205	52.35083	3.02E-65	10	28.75989
P01009	418	46736.2	5.31	Alpha-1-a	SERPINA1	SERPINA1	Alpha-1-a	7	264.0736	49.15819	3.39E-53	7	22.2488
Q8NC51	408	44965.2	9.22	SERPINE1	SERBP1	SERBP1	SERPINE1	20	3110.743	51.94618	1.16E-63	20	47.79412
Q9UHD8	586	65400.9	9.46	Septin-9	SEPTIN9	SEPTIN9	Septin-9	11	301.9121	51.10708	1.92E-60	11	22.69625
Q16181	437	50679.7	8.99	Septin-7	SEPTIN7	SEPTIN7	Septin-7	4	178.3292	49.16225	3.28E-53	3	11.21281
Q15019	361	41487.2	6.58	Septin-2	SEPTIN2	SEPTIN2	Septin-2	6	161.517	56.72449	4.86E-83	6	22.43767
Q9NVA2	429	49398	6.8	Septin-11	SEPTIN11	SEPTIN11	Septin-11	2	55.82986	39.30304	3.09E-24	1	5.594406
Q9P0V9	454	52592.7	6.79	Septin-10	SEPTIN10	SEPTIN10	Septin-10	2	88.79857	52.54131	5.44E-66	2	4.405286
Q9BQF6	1050	119656.7	6.58	Sentrin-sp	SENP7	SENP7	Sentrin-sp	9	496.3687	50.94247	8.11E-60	9	11.04762
Q9H4L4	574	65009.3	8.7	Sentrin-sp	SENP3	SENP3	Sentrin-sp	7	274.7275	53.33957	3.79E-69	7	16.55052
Q9HC62	589	67854.1	10	Sentrin-sp	SENP2	SENP2	Sentrin-sp	1	149.9506	47.16769	2.88E-46	1	2.546689
O75326	666	74823.2	7.66	Semaphori	SEMA7A	SEMA7A	Semaphori	7	213.7081	43.8479	6.84E-36	7	12.76276
Q13275	785	88380.6	8.32	Semaphori	SEMA3F	SEMA3F	Semaphori	1	83.00742	33.19026	2.95E-13	1	1.910828
Q99985	751	85206.4	8.83	Semaphori	SEMA3C	SEMA3C	Semaphori	10	781.1854	50.32583	1.72E-57	10	15.31292
Q13214	749	83120.8	9.05	Semaphori	SEMA3B	SEMA3B	Semaphori	3	76.15344	40.10831	4.37E-26	3	4.405874
Q14563	771	88888.7	7.43	Semaphori	SEMA3A	SEMA3A	Semaphori	8	291.4304	49.9899	3.07E-56	8	11.67315
P62341	195	22324	8.8	Thioredoxi	SELENOT	SELENOT	Thioredoxi	2	61.80185	33.26129	2.36E-13	2	11.79487
Q9BVL4	669	73488.5	5.82	Protein ad	SELENOO	SELENOO	Protein ad	1	87.3037	49.78394	1.77E-55	1	2.989537
O60613	165	18091.6	4.67	Selenoprot	SELENOF	SELENOF	Selenoprot	3	118.8382	45.81115	7.64E-42	3	20.60606
Q9UBV2	794	88754.1	5.06	Protein sel	SEL1L	SEL1L	Protein sel	5	182.4719	53.86572	2.95E-71	5	9.697733
Q96EE3	360	39648.3	8.18	Nucleopor	SEH1L	SEH1L	Nucleopor	4	337.9285	45.58428	3.96E-41	4	11.94444
Q96T21	854	95461.2	8.27	Selenocyst	SECISBP2	SECISBP2	Selenocyst	2	98.6405	39.13401	7.34E-24	2	2.576112
Q9UGP8	760	87996.3	4.96	Translocati	SEC63	SEC63	Translocati	6	142.7772	50.33133	1.64E-57	6	10.39474
Q99442	399	45861.5	7.14	Translocati	SEC62	SEC62	Translocati	1	60.21273	46.40743	9.32E-44	1	3.007519
P60059	68	7741.3	10.78	Protein tra	SEC61G	SEC61G	Protein tra	1	233.4407	34.09238	1.54E-14	1	17.64706
P60468	96	9974.4	12.08	Protein tra	SEC61B	SEC61B	Protein tra	3	311.0698	47.42802	3.82E-47	3	37.5
P61619	476	52264.2	8.17	Protein tra	SEC61A1	SEC61A1	Protein tra	6	606.3082	46.9748	1.27E-45	5	16.80672
O94979	1220	133013.6	6.89	Protein tra	SEC31A	SEC31A	Protein tra	3	107.9472	40.46223	6.29E-27	3	3.360656
O94855	1032	113008.8	7.26	Protein tra	SEC24D	SEC24D	Protein tra	1	80.96157	35.74263	3.45E-17	1	1.453488
P53992	1094	118323.8	7.07	Protein tra	SEC24C	SEC24C	Protein tra	8	319.2574	51.38277	1.7E-61	8	11.06033
O95487	1268	137416.4	6.65	Protein tra	SEC24B	SEC24B	Protein tra	15	288.5962	50.78963	3.1E-59	15	13.64353
O95486	1093	119748.4	7.72	Protein tra	SEC24A	SEC24A	Protein tra	6	101.5723	48.88967	3.11E-52	6	8.142726
Q9Y6Y8	1000	111075.6	5.24	SEC23-inte	SEC23IP	SEC23IP	SEC23-inte	9	391.3394	53.66692	1.89E-70	9	10.7
Q15437	767	86478.5	6.88	Protein tra	SEC23B	SEC23B	Protein tra	13	588.5132	52.02373	5.77E-64	10	20.59974
Q15436	765	86160.1	7.07	Protein tra	SEC23A	SEC23A	Protein tra	7	162.5974	52.54222	5.41E-66	7	10.45752
O75396	215	24740.3	8.74	Vesicle-tra	SEC22B	SEC22B	Vesicle-tra	7	429.3775	48.46845	9.78E-51	7	38.13953
Q96JE7	1060	116603.6	6	Protein tra	SEC16B	SEC16B	Protein tra	1	114.7514	35.12718	3.73E-16	1	0.943396
O15027	2357	251891.5	5.63	Protein tra	SEC16A	SEC16A	Protein tra	5	145.8549	46.59267	2.32E-44	5	2.545609
P55735	322	35540.3	5.16	Protein SE	SEC13	SEC13	Protein SE	5	333.5579	49.81823	1.33E-55	5	22.67081
P67812	179	20625.2	9.97	Signal pep	SEC11A	SEC11A	Signal pep	1	4006.805	32.81413	9.35E-13	1	5.027933

Q96GA7	329	34674	6.88	Serine deh	SDSL	SDSL	Serine deh	3	100.4223	48.21381	7.61E-50	3	15.80547
P21912	280	31629.4	8.92	Succinate	SDHB	SDHB	Succinate	7	720.4878	52.05902	4.21E-64	7	25.71429
P31040	664	72691	7.41	Succinate	SDHA	SDHA	Succinate	12	597.4099	53.68128	1.66E-70	12	23.04217
Q9BRK5	362	41806.5	4.49	45 kDa cal	SDF4	SDF4	45 kDa cal	2	101.0562	52.47388	9.96E-66	2	6.077348
Q9HCN8	221	23598.2	7.04	Stromal ce	SDF2L1	SDF2L1	Stromal ce	1	329.83	53.94008	1.47E-71	1	5.882353
O00560	298	32444.1	7.63	Syntenin-1	SDCBP	SDCBP	Syntenin-1	6	1181.752	50.16675	6.75E-57	6	34.22819
P31431	198	21641.3	4.13	Syndecan-	SDC4	SDC4	Syndecan-	9	7034.182	51.13157	1.55E-60	9	42.92929
O75056	442	45496.5	4.33	Syndecan-	SDC3	SDC3	Syndecan-	1	169.9443	47.80444	1.98E-48	1	4.977376
P18827	310	32461.4	4.26	Syndecan-	SDC1	SDC1	Syndecan-	4	701.9829	47.34737	7.17E-47	4	15.48387
Q9NVU7	687	79870.7	9.84	Protein SD	SDAD1	SDAD1	Protein SD	9	1002.057	49.0752	6.73E-53	9	13.9738
Q6P3W7	929	103708	8.38	SCY1-like	SCYL2	SCYL2	SCY1-like	5	167.8529	45.1139	1.15E-39	5	6.781485
Q96KG9	808	89630.6	6.23	N-termina	SCYL1	SCYL1	N-termina	1	107.6888	38.0326	1.64E-21	1	1.361386
Q14160	1630	174913	4.72	Protein scr	SCRIB	SCRIB	Protein scr	15	350.4165	51.2858	3.99E-61	14	13.06748
Q9HB40	452	50830.3	5.53	Retinoid-ir	SCPEP1	SCPEP1	Retinoid-ir	1	65.32947	44.08005	1.44E-36	1	2.433628
P22307	547	58993.2	6.88	Sterol carri	SCP2	SCP2	Sterol carri	1	199.514	40.67196	1.96E-27	1	2.193784
O43819	266	29809.7	9.07	Protein SC	SCO2	SCO2	Protein SC	1	131.7909	28.85659	1.09E-08	1	3.759398
O75880	301	33813.7	9.22	Protein SC	SCO1	SCO1	Protein SC	1	85.93842	35.95979	1.45E-17	1	6.644518
Q9UQR0	700	77256.2	8.72	Sex comb	SCML2	SCML2	Sex comb	8	302.6165	49.31645	9.11E-54	8	14.14286
Q96GD3	660	73353.3	9.71	Polycomb	SCMH1	SCMH1	Polycomb	5	280.176	56.54498	2.29E-82	5	10.45455
Q8WVM8	642	72379.2	6.17	Sec1 famil	SCFD1	SCFD1	Sec1 famil	4	109.0883	50.95735	7.13E-60	4	9.345794
Q86SK9	330	37610	10.02	Stearoyl-C	SCD5	SCD5	Stearoyl-C	1	88.04845	50.51394	3.43E-58	1	4.242424
O00767	359	41522.3	9.28	Stearoyl-C	SCD	SCD	Stearoyl-C	3	447.7657	52.48883	8.69E-66	3	13.92758
Q14108	478	54289.7	4.76	Lysosome	SCARB2	SCARB2	Lysosome	4	246.1357	46.7825	5.51E-45	4	8.577406
Q8WTV0	552	60877.4	8.31	Scavenger	SCARB1	SCARB1	Scavenger	2	153.4991	39.17236	6.04E-24	2	3.623188
Q969E2	229	25727.7	8.98	Secretory	SCAMP4	SCAMP4	Secretory	1	79.42886	45.06807	1.58E-39	1	4.803493
O14828	347	38286.6	7.73	Secretory	SCAMP3	SCAMP3	Secretory	4	150.1585	49.17654	2.92E-53	4	15.27378
O15126	338	37920.1	7.47	Secretory	SCAMP1	SCAMP1	Secretory	2	223.7816	43.3373	1.96E-34	2	10.65089
Q8N9R8	606	70398.9	8.82	Protein SC	SCAI	SCAI	Protein SC	5	212.5003	48.97137	1.59E-52	5	8.415842
Q9UPN6	1271	140518.5	8.52	SR-related	SCAF8	SCAF8	SR-related	3	113.3897	51.53626	4.34E-62	2	3.06845
O95104	1147	125867.8	10.16	SR-related	SCAF4	SCAF4	SR-related	4	243.36	48.12466	1.55E-49	4	4.359198
Q99590	1463	164651	8.55	Protein SC	SCAF11	SCAF11	Protein SC	18	1099.58	51.88994	1.91E-63	18	13.53383
Q9H7N4	1312	139268.2	9.84	Splicing fa	SCAF1	SCAF1	Splicing fa	10	325.3547	50.40857	8.48E-58	9	8.841463
O95248	1868	208441.5	6.89	Myotubula	SBF1	SBF1	Myotubula	7	92.43812	48.38591	1.9E-50	6	4.389722
Q9Y3A5	250	28763.3	9.15	Ribosome	SBDS	SBDS	Ribosome	4	105.1834	33.94605	2.53E-14	4	15.6
Q9H4B6	383	44633.7	9.37	Protein sal	SAV1	SAV1	Protein sal	2	76.61322	36.76308	5.07E-19	2	5.483029
Q15020	963	109933.5	5.25	Squamous	SART3	SART3	Squamous	31	1049.052	54.23645	9.19E-73	31	39.04465
O43290	800	90254.1	5.92	U4/U6.U5	SART1	SART1	U4/U6.U5	37	1970.645	56.5956	1.48E-82	37	46.625
Q9NP81	518	58282.1	8.22	Serine--tR	SARS2	SARS2	Serine--tR	17	933.7538	50.69924	6.82E-59	17	47.6834
P49591	514	58776.8	6.38	Serine--tR	SARS1	SARS1	Serine--tR	7	177.7697	56.84303	1.78E-83	7	17.70428
P82979	210	23670.6	6.33	SAP doma	SARNP	SARNP	SAP doma	2	182.5428	51.59548	2.58E-62	2	9.52381
Q96BY9	339	36974.9	7.64	Store-oper	SARAF	SARAF	Store-oper	2	73.77802	39.95635	9.91E-26	2	6.19469
Q9NR31	198	22366.6	6.68	Small COP	SAR1A	SAR1A	Small COP	4	425.4665	46.38841	1.07E-43	2	25.75758
Q9HAJ7	183	20876.4	9.88	Histone de	SAP30L	SAP30L	Histone de	1	122.5764	34.63145	2.31E-15	1	4.918033
Q9UHR5	308	33870.1	4.46	SAP30-bin	SAP30BP	SAP30BP	SAP30-bin	7	1148.419	52.37649	2.4E-65	7	24.67532
O75446	220	23306	9.6	Histone de	SAP30	SAP30	Histone de	1	77.10144	47.23344	1.73E-46	1	4.090909
O00422	153	17561	9.86	Histone de	SAP18	SAP18	Histone de	9	549.6069	50.78174	3.32E-59	9	58.16993
Q9H0E3	1048	110323	10.45	Histone de	SAP130	SAP130	Histone de	3	89.27875	43.37116	1.57E-34	3	3.625954
Q9Y512	469	51975.8	6.9	Sorting an	SAMM50	SAMM50	Sorting an	9	1037.619	55.22033	7.97E-77	9	21.96162
Q9Y3Z3	626	72199.9	7.14	Deoxynucl	SAMHD1	SAMHD1	Deoxynucl	27	713.5793	52.22766	9.12E-65	27	49.68051
Q6SPF0	538	56051.2	7.67	Sterile alpl	SAMD1	SAMD1	Sterile alpl	1	75.44855	44.17027	7.84E-37	1	3.717472
Q14151	953	107472.6	6.07	Scaffold at	SAFB2	SAFB2	Scaffold at	3	183.3006	41.41507	2.77E-29	3	3.672613
Q15424	915	102640.7	5.12	Scaffold at	SAFB	SAFB	Scaffold at	26	5196.186	52.33619	3.45E-65	17	26.88525
Q9UBE0	346	38449.5	4.93	SUMO-act	SAE1	SAE1	SUMO-act	5	196.53	47.33038	8.18E-47	5	19.07514
Q9NTJ5	587	66966.4	7.13	Phosphatic	SACM1L	SACM1L	Phosphatic	5	157.7352	47.10822	4.55E-46	5	8.006814
Q96ER3	474	53557.5	4.12	Protein SA	SAAL1	SAAL1	Protein SA	2	212.1654	41.27892	6.13E-29	2	4.43038
P06702	114	13242	6.08	Protein S1	S100A9	S100A9	Protein S1	3	134.3728	44.69323	2.19E-38	3	29.82456
P05109	93	10834.4	7.07	Protein S1	S100A8	S100A8	Protein S1	2	326.0416	49.2788	1.24E-53	2	23.65591
P31151	101	11470.9	6.78	Protein S1	S100A7	S100A7	Protein S1	2	58.07743	47.82193	1.73E-48	2	30.69307
P26447	101	11728.4	5.92	Protein S1	S100A4	S100A4	Protein S1	3	516.7281	47.4574	3.04E-47	3	28.71287
Q96FQ6	103	11801.3	6.79	Protein S1	S100A16	S100A16	Protein S1	5	427.832	49.10933	5.07E-53	5	55.33981
Q9HCY8	104	11662	4.93	Protein S1	S100A14	S100A14	Protein S1	4	406.0574	53.50275	8.46E-70	4	44.23077
Q99584	98	11471.1	5.96	Protein S1	S100A13	S100A13	Protein S1	3	678.5368	48.71832	1.28E-51	3	32.65306
P31949	105	11740.3	7.26	Protein S1	S100A11	S100A11	Protein S1	4	573.1741	47.66508	5.96E-48	4	37.14286
P60903	97	11203.1	7.51	Protein S1	S100A10	S100A10	Protein S1	3	428.0006	45.23484	4.86E-40	3	38.14433
Q9Y230	463	51156.1	5.32	RuvB-like 2	RUVBL2	RUVBL2	RuvB-like 2	17	4334.279	50.64895	1.05E-58	17	39.30886
Q9Y265	456	50227.6	6.37	RuvB-like 1	RUVBL1	RUVBL1	RuvB-like 1	17	2193.078	52.51992	6.56E-66	17	44.95614
Q96GQ5	468	51017.4	6.93	RUS family	RUSF1	RUSF1	RUS family	1	112.2035	30.66049	2.84E-10	1	2.350427
Q13761	415	44355.2	9.77	Runt-relat	RUNX3	RUNX3	Runt-relat	2	165.0603	45.66119	2.27E-41	1	6.746988
Q01196	453	48736.2	9.62	Runt-relat	RUNX1	RUNX1	Runt-relat	3	237.9889	41.86842	1.89E-30	3	9.271523
Q96T51	708	79817.2	5.52	RUN and F	RUFY1	RUFY1	RUN and F	1	86.09122	37.22222	6.78E-20	1	1.553672
Q9Y224	244	28067.8	6.64	RNA trans	RTRAF	RTRAF	RNA trans	11	2199.75	51.94654	1.16E-63	11	55.32787
Q86UN3	420	46105	7.66	Reticulon-	RTN4RL2	RTN4RL2	Reticulon-	3	137.0282	42.70403	1.11E-32	3	7.619048
Q9BZR6	473	50707.4	8.95	Reticulon-	RTN4R	RTN4R	Reticulon-	1	53.96483	25.48189	7.51E-07	1	2.748414
Q9NQC3	1192	129929.8	4.14	Reticulon-	RTN4	RTN4	Reticulon-	5	5422.616	49.13133	4.24E-53	5	7.634228
O95197	1032	112609.9	4.58	Reticulon-	RTN3	RTN3	Reticulon-	1	122.9009	47.9113	8.53E-49	1	1.065891
Q9BST9	563	62666.8	7.47	Rhotekin	RTKN	RTKN	Rhotekin	1	136.7336	48.5385	5.54E-51	1	2.664298
Q92541	710	80312.8	8.49	RNA polyn	RTF1	RTF1	RNA polyn	12	1076.649	48.23993	6.19E-50	12	19.01408
Q9Y3I0	505	55209.9	7.23	RNA-splici	RTCB	RTCB	RNA-splici	24	2510.748	52.56688	4.32E-66	24	47.52475
O00442	366	39336.4	7.94	RNA 3'-ter	RTCA	RTCA	RNA 3'-ter	5	233.7589	52.2362	8.47E-65	5	17.48634
Q7L4I2	434	50559.2	11.88	Arginine/s	RSRC2	RSRC2	Arginine/s	5	342.2644	49.46271	2.67E-54	5	12.4424
Q96IZ7	334	38676.6	11.68	Serine/Arg	RSRC1	RSRC1	Serine/Arg	4	914.9802	46.19655	4.5E-43	4	16.76647
Q96DX4	576	64179.5	5.26	RING finge	RSPRY1	RSPRY1	RING finge	2	80.74022	38.81417	3.68E-23	2	3.472222
Q9UHA3	163	19621	10.7	Probable r	RSL24D1	RSL24D1	Probable r	3	433.2749	45.13401	9.94E-40	3	17.17791
O76021	490	54972	10.89	Ribosomal	RSL1D1	RSL1D1	Ribosomal	16	3014.229	51.86766	2.32E-63	16	34.69388
Q96T23	1441	163818.9	4.65	Remodelin	RSF1	RSF1	Remodelin	38	2134.185	53.58563	4E-70	38	25.19084
Q6PCB5	846	94868.8	9.2	Lysine-spe	RSBN1L	RSBN1L	Lysine-spe	23	732.2316	52.26098	6.78E-65	22	32.2695
Q5VWQ0	802	90071.6	8.77	Lysine-spe	RSBN1	RSBN1	Lysine-spe	19	3252.063	51.00686	4.62E-60	19	28.6783
Q9HA92	442	48713.1	7.84	Radical S-a	RSAD1	RSAD1	Radical S-a	4	85.22482	36.05468	9.86E-18	4	10.85973
Q15050	365	41193.1	11.4	Ribosome	RRS1	RRS1	Ribosome	10	1449.166	50.372	1.16E-57	10	32.87671
O43818	475	51840.2	7.92	U3 small n	RRP9	RRP9	U3 small n	8	388.9461	51.79053	4.6E-63	8	20.63158
O43159	456	50714.3	10.06	Ribosomal	RRP8	RRP8	Ribosomal	9	573.5071	48.34628	2.62E-50	9	25.2193
Q9Y3A4	280	32333.8	10.14	Ribosomal	RRP7A	RRP7A	Ribosomal	4	63.23478	45.75314	1.17E-41	4	22.5
Q96EU6	259	29822.9	10.87	Ribosomal	RRP36	RRP36	Ribosomal	1	75.30978	38.14294	9.72E-22	1	5.791506

Q14684	758	84426.9	10.49	Ribosomal	RRP1B	RRP1B	Ribosomal	13	547.4216	53.39433	2.28E-69	13	20.84433
Q9Y3B9	282	31484	5.15	RRP15-like	RRP15	RRP15	RRP15-like	8	2067.21	48.26199	5.17E-50	8	28.36879
Q5JTH9	1297	143700.7	9.02	RRP12-like	RRP12	RRP12	RRP12-like	26	1094.559	53.27413	6.85E-69	26	23.97841
P56182	461	52838.8	9.96	Ribosomal	RRP1	RRP1	Ribosomal	5	408.9753	50.57785	1.96E-58	5	12.14751
Q7LG56	351	40736.1	4.61	Ribonuclec	RRM2B	RRM2B	Ribonuclec	3	90.79402	51.19914	8.55E-61	3	7.977208
P23921	792	90069.4	7.16	Ribonuclec	RRM1	RRM1	Ribonuclec	4	51.3219	44.65165	2.92E-38	4	6.565657
Q92766	1687	181418.4	6.98	Ras-respo	RREB1	RREB1	Ras-respo	3	43.63941	35.07926	4.47E-16	3	2.074689
Q9P2E9	1410	152455.8	9.2	Ribosome-	RRBP1	RRBP1	Ribosome-	21	368.2798	52.30989	4.37E-65	20	17.23404
P62070	204	23399.4	5.81	Ras-relate	RRAS2	RRAS2	Ras-relate	3	86.00074	52.93592	1.54E-67	3	18.62745
P10301	218	23480.3	6.93	Ras-relate	RRAS	RRAS	Ras-relate	2	293.0331	51.12576	1.63E-60	1	11.92661
Q9HB90	399	44223.5	4.72	Ras-relate	RRAGC	RRAGC;RR	Ras-relate	1	126.3854	44.08178	1.43E-36	1	2.255639
Q5VZM2	374	43250	6.13	Ras-relate	RRAGB	RRAGB;RR	Ras-relate	1	92.81759	48.54795	5.13E-51	1	2.673797
Q96CM3	377	42204.9	10.53	Pseudouric	RPUSD4	RPUSD4	Pseudouric	8	312.2988	46.69307	1.09E-44	8	21.22016
Q6P087	351	38460.4	10.95	Mitochonc	RPUSD3	RPUSD3	Mitochonc	6	250.4626	48.62496	2.74E-51	6	20.22792
Q8N122	1335	149036.2	6.87	Regulatory	RPTOR	RPTOR	Regulatory	6	117.7929	47.20913	2.09E-46	6	5.842697
P08865	295	32853.8	4.51	Small ribos	RPSA	RPSA	Small ribos	9	6600.242	48.31595	3.35E-50	2	29.83051
P46781	194	22591.2	11.32	Small ribos	RPS9	RPS9	Small ribos	15	28929.25	51.7515	6.49E-63	15	50.51546
P62241	208	24205	10.97	Small ribos	RPS8	RPS8	Small ribos	14	57263.64	50.20515	4.87E-57	14	59.13462
P62081	194	22126.7	10.86	Small ribos	RPS7	RPS7	Small ribos	8	6516.833	50.43335	6.84E-58	8	29.89691
P23443	525	59139.1	6.64	Ribosomal	RPS6KB1	RPS6KB1	Ribosomal	4	344.946	44.54923	5.91E-38	4	11.2381
O75582	802	89864.7	7.1	Ribosomal	RPS6KA5	RPS6KA5	Ribosomal	6	188.8589	43.72265	1.57E-35	6	7.98005
O75676	772	85605	8.4	Ribosomal	RPS6KA4	RPS6KA4	Ribosomal	9	177.3003	50.51983	3.26E-58	9	15.80311
P51812	740	83735.3	6.88	Ribosomal	RPS6KA3	RPS6KA3	Ribosomal	10	576.3718	50.4217	7.58E-58	6	16.89189
Q15418	735	82722.4	7.94	Ribosomal	RPS6KA1	RPS6KA1	Ribosomal	4	117.1635	44.12371	1.07E-36	4	8.29932
P62753	249	28680.4	11.52	Small ribos	RPS6	RPS6	Small ribos	17	68355.31	49.8557	9.66E-56	17	46.18474
P46782	204	22876.2	10.3	Small ribos	RPS5	RPS5	Small ribos	11	13060.71	55.14206	1.68E-76	11	43.13725
P62701	263	29597.5	10.85	Small ribos	RPS4X	RPS4X	Small ribos	15	23815.1	53.84252	3.66E-71	7	53.9924
P61247	264	29944.8	10.44	Small ribos	RPS3A	RPS3A	Small ribos	18	8221.535	51.94575	1.16E-63	18	62.87879
P23396	243	26688.1	10.26	Small ribos	RPS3	RPS3	Small ribos	17	65041.36	51.03277	3.68E-60	17	66.66667
P62273	56	6676.7	10.64	Small ribos	RPS29	RPS29	Small ribos	2	215.6435	48.24657	5.86E-50	2	33.92857
P62857	69	7841	11.33	Small ribos	RPS28	RPS28	Small ribos	3	2207.973	46.445	7.03E-44	3	46.37681
Q71UM5	84	9477.1	9.99	Ribosomal	RPS27L	RPS27L	Ribosomal	3	2373.211	50.30823	2E-57	1	38.09524
P62979	156	17964.8	10.25	Ubiquitin-	RPS27A	RPS27A	Ubiquitin-	9	16329.96	49.58307	9.66E-55	5	57.69231
P42677	84	9461.1	9.99	Small ribos	RPS27	RPS27	Small ribos	2	1770.17	45.29822	3.1E-40	2	14.28571
P62854	115	13015.3	11.61	Small ribos	RPS26	RPS26	Small ribos	4	10474.29	45.39332	1.57E-40	1	39.13043
P62851	125	13742	10.85	Small ribos	RPS25	RPS25	Small ribos	5	34098.76	46.23558	3.37E-43	5	25.6
P62847	133	15423.1	11.48	Small ribos	RPS24	RPS24	Small ribos	5	4132.722	47.18216	2.57E-46	5	35.33835
P62266	143	15807.5	11.23	Small ribos	RPS23	RPS23	Small ribos	5	16222.21	50.39034	9.92E-58	5	32.16783
P63220	83	9111.3	8.75	Small ribos	RPS21	RPS21	Small ribos	4	922.2476	51.17588	1.05E-60	4	44.57831
P60866	119	13372.6	10.71	Small ribos	RPS20	RPS20	Small ribos	4	3767.254	47.48787	2.39E-47	4	23.52941
P15880	293	31324.2	10.9	Small ribos	RPS2	RPS2	Small ribos	15	29396.41	51.21782	7.24E-61	15	50.17065
Q86WX3	136	15433.7	11.43	Active regu	RPS19BP1	RPS19BP1	Active regu	4	547.6656	47.01951	8.99E-46	4	34.55882
P39019	145	16060.4	10.99	Small ribos	RPS19	RPS19	Small ribos	9	12709.35	50.50795	3.61E-58	9	44.13793
P62269	152	17718.6	11.61	Small ribos	RPS18	RPS18	Small ribos	13	13851.02	50.99281	5.23E-60	13	63.15789
P08708	135	15550	10.47	Small ribos	RPS17	RPS17	Small ribos	12	7950.037	49.38752	5.02E-54	12	65.92593
P62249	146	16445.2	10.81	Small ribos	RPS16	RPS16	Small ribos	12	37772.17	48.55154	4.99E-51	12	58.90411
P62244	130	14839.4	10.87	Small ribos	RPS15A	RPS15A	Small ribos	7	8021.254	50.69545	7.04E-59	7	55.38462
P62841	145	17040	11.04	Small ribos	RPS15	RPS15	Small ribos	6	1603.127	49.16296	3.26E-53	6	62.75862
P62263	151	16272.6	10.79	Small ribos	RPS14	RPS14	Small ribos	9	25092.78	50.16555	6.81E-57	9	41.0596
P62277	151	17222.1	11.19	Small ribos	RPS13	RPS13	Small ribos	10	12054.12	50.36037	1.28E-57	10	50.99338
P25398	132	14514.8	7.27	Small ribos	RPS12	RPS12	Small ribos	9	14510.57	53.90403	2.08E-71	9	71.21212
P62280	158	18430.6	10.96	Small ribos	RPS11	RPS11	Small ribos	13	12858.25	48.31653	3.34E-50	13	58.22785
P46783	165	18897.7	10.74	Small ribos	RPS10	RPS10	Small ribos	10	6808.66	52.22353	9.46E-65	6	58.78788
Q5VT52	1461	156018	7.44	Regulation	RPRD2	RPRD2	Regulation	3	59.82185	45.57618	4.2E-41	3	3.216975
Q9NQG5	326	36899.4	5.78	Regulation	RPRD1B	RPRD1B	Regulation	6	705.7102	50.19127	5.47E-57	6	21.77914
Q96P16	312	35719.3	7.64	Regulation	RPRD1A	RPRD1A	Regulation	10	691.9148	51.54318	4.09E-62	8	42.94872
O75818	363	41833.6	6.67	Ribonuclea	RPP40	RPP40	Ribonuclea	5	198.3635	54.12314	2.61E-72	5	16.25344
P78345	283	31834	10.69	Ribonuclea	RPP38	RPP38	Ribonuclea	8	425.1998	49.78068	1.82E-55	8	28.97527
P78346	268	29320.8	9.23	Ribonuclea	RPP30	RPP30	Ribonuclea	11	1196.061	50.16953	6.59E-57	11	48.13433
Q8N5L8	163	17631	10.91	Ribonuclea	RPP25L	RPP25L	Ribonuclea	3	336.788	45.33952	2.31E-40	3	18.40491
Q9H633	154	17569.9	9.79	Ribonuclea	RPP21	RPP21	Ribonuclea	2	65.20404	36.02663	1.1E-17	2	11.68831
P04844	631	69283.3	5.46	Dolichyl-d	RPN2	RPN2	Dolichyl-d	11	951.1147	48.79187	6.97E-52	11	26.78288
P04843	607	68568.8	6.34	Dolichyl-d	RPN1	RPN1	Dolichyl-d	21	2158.463	54.00674	7.84E-72	21	38.3855
P05387	115	11664.8	4.14	Large ribos	RPLP2	RPLP2	Large ribos	7	14384.79	49.28333	1.2E-53	7	73.91304
P05386	114	11513.8	3.95	Large ribos	RPLP1	RPLP1	Large ribos	1	4375.092	52.26455	6.56E-65	1	14.03509
P05388	317	34273.2	5.77	Large ribos	RPLP0	RPLP0	Large ribos	11	23056.41	52.1722	1.52E-64	3	40.06309
P32969	192	21863.3	10.62	Large ribos	RPL9	RPL9	Large ribos	9	8037.633	52.54841	5.12E-66	9	41.14583
P62917	257	28024.5	11.64	Large ribos	RPL8	RPL8	Large ribos	16	49568.77	47.70534	4.34E-48	16	57.97665
Q6DKI1	255	29669	11.24	Ribosomal	RPL7L1	RPL7L1	Ribosomal	7	810.4099	48.67965	1.75E-51	7	28.62745
P62424	266	29995.4	11.32	Large ribos	RPL7A	RPL7A	Large ribos	23	27192.74	51.44117	1.01E-61	23	60.90226
P18124	248	29225.6	11.31	Large ribos	RPL7	RPL7	Large ribos	19	77408.06	50.40522	8.73E-58	19	50.80645
Q02878	288	32727.7	11.29	Large ribos	RPL6	RPL6	Large ribos	19	41230.6	50.50325	3.77E-58	19	50.69444
P46777	297	34362.4	10.21	Large ribos	RPL5	RPL5	Large ribos	17	20860.61	53.61128	3.17E-70	17	44.78114
P36578	427	47696.9	11.7	Large ribos	RPL4	RPL4	Large ribos	32	71487.23	50.22815	4E-57	32	55.03513
P62891	51	6406.7	13.06	Large ribos	RPL39	RPL39;RPL	Large ribos	1	174.1886	40.78407	1.04E-27	1	19.60784
P63173	70	8217.8	10.82	Large ribos	RPL38	RPL38	Large ribos	6	869.5333	47.51569	1.92E-47	6	52.85714
P61513	92	10275.2	11.11	Large ribos	RPL37A	RPL37A	Large ribos	6	2846.403	51.1905	9.22E-61	5	52.17391
Q969Q0	106	12468.8	11.35	Ribosomal	RPL36AL	RPL36AL	Ribosomal	1	4902.157	43.15894	6.19E-34	1	9.433962
P83881	106	12440.8	11.29	Large ribos	RPL36A	RPL36A	Large ribos	5	1390.691	48.85494	4.15E-52	1	41.50943
Q9Y3U8	105	12253.6	12.13	Large ribos	RPL36	RPL36	Large ribos	5	35641.07	47.47961	2.55E-47	5	37.14286
P18077	110	12537.6	11.64	Large ribos	RPL35A	RPL35A	Large ribos	8	24106.27	49.51519	1.71E-54	8	41.81818
P42766	123	14551.4	11.7	Large ribos	RPL35	RPL35	Large ribos	8	32056.07	50.01988	2.38E-56	8	52.03252
P49207	117	13292.9	12.02	Large ribos	RPL34	RPL34	Large ribos	7	16701.52	46.05925	1.24E-42	7	28.20513
P62910	135	15859.7	11.9	Large ribos	RPL32	RPL32	Large ribos	11	10291.35	50.32177	1.78E-57	11	50.37037
P62899	125	14462.8	11.22	Large ribos	RPL31	RPL31	Large ribos	6	9790.2	50.44001	6.46E-58	6	43.2
P62888	115	12784	10.13	Large ribos	RPL30	RPL30	Large ribos	7	32444.26	49.46044	2.72E-54	7	44.34783
P39023	403	46108.7	10.89	Large ribos	RPL3	RPL3	Large ribos	24	17974.28	53.94638	1.4E-71	22	48.63524
P47914	159	17751.9	12.22	Large ribos	RPL29	RPL29	Large ribos	3	6382.305	51.9182	1.48E-63	3	15.09434
P46779	137	15747.4	12.52	Large ribos	RPL28	RPL28	Large ribos	8	8567.94	50.29842	2.18E-57	8	42.33577
P46776	148	16561.4	11.64	Large ribos	RPL27A	RPL27A	Large ribos	5	28696.28	48.50694	7.16E-51	5	31.08108
P61353	136	15797.6	11.23	Large ribos	RPL27	RPL27	Large ribos	12	52027.64	50.943	8.09E-60	12	58.82353
Q9UNX3	145	17256.2	11.24	Ribosomal	RPL26L1	RPL26L1	Ribosomal	2	622.0182	44.43176	1.32E-37	2	11.72414
P61254	145	17258.2	11.24	Large ribos	RPL26	RPL26	Large ribos	13	9295.169	51.15261	1.29E-60	3	49.65517

P83731	157	17778.8	11.87	Large ribos	RPL24	RPL24	Large ribos	7	9483.251	49.667	4.75E-55	7	37.57962
P62750	156	17694.9	11.16	Large ribos	RPL23A	RPL23A	Large ribos	14	39434.09	51.40319	1.42E-61	14	52.5641
P62829	140	14865.3	11.2	Large ribos	RPL23	RPL23	Large ribos	7	1072.082	53.15707	2.01E-68	7	47.85714
Q6P5R6	122	14606.4	9.95	Ribosomal	RPL22L1	RPL22L1	Ribosomal	2	1332.917	48.75275	9.62E-52	2	19.67213
P35268	128	14786.9	9.75	Large ribos	RPL22	RPL22	Large ribos	4	9008.98	47.86315	1.25E-48	4	21.09375
P46778	160	18564.8	11.17	Large ribos	RPL21	RPL21	Large ribos	7	11130.25	52.30351	4.62E-65	7	36.875
P84098	196	23465.8	12.03	Large ribos	RPL19	RPL19	Large ribos	9	4614.47	50.45625	5.62E-58	9	36.73469
Q02543	176	20762.2	11.33	Large ribos	RPL18A	RPL18A	Large ribos	10	26654.84	51.63732	1.78E-62	10	43.18182
Q07020	188	21634.3	12.24	Large ribos	RPL18	RPL18	Large ribos	10	43842.42	51.02741	3.85E-60	10	45.21277
P18621	184	21396.9	10.86	Large ribos	RPL17	RPL17	Large ribos	11	23094.22	51.80572	4.04E-63	11	45.65217
P61313	204	24145.9	12.13	Large ribos	RPL15	RPL15	Large ribos	13	29298.59	48.77158	8.25E-52	13	45.58824
P50914	215	23431.7	11.65	Large ribos	RPL14	RPL14	Large ribos	5	23863.62	50.52834	3.03E-58	5	24.65116
P40429	203	23577.1	11.57	Large ribos	RPL13A	RPL13A	Large ribos	12	29293.12	49.94593	4.49E-56	6	40.8867
P26373	211	24261.3	12.18	Large ribos	RPL13	RPL13	Large ribos	17	63939.67	49.30275	1.02E-53	17	56.87204
P30050	165	17818.4	10.11	Large ribos	RPL12	RPL12	Large ribos	5	6065.129	51.51785	5.11E-62	5	43.0303
P62913	178	20252.2	10.14	Large ribos	RPL11	RPL11	Large ribos	10	23431.64	51.93946	1.23E-63	10	58.42697
P62906	217	24831.1	10.62	Large ribos	RPL10A	RPL10A	Large ribos	11	2920.575	49.96117	3.94E-56	11	41.01382
P27635	214	24576.7	10.75	Large ribos	RPL10	RPL10	Large ribos	12	15414.28	49.54063	1.38E-54	8	51.86916
P49247	311	33268.7	8.72	Ribose-5-p	RPIA	RPIA	Ribose-5-p	2	126.0831	45.86668	5.09E-42	2	5.466238
Q9H7B2	306	35582.6	10.67	Ribosome	RPF2	RPF2	Ribosome	8	194.2811	52.718	1.11E-66	8	30.71895
Q9H9Y2	349	40110.6	10.68	Ribosome	RPF1	RPF1	Ribosome	7	625.9701	49.94076	4.68E-56	7	20.63037
Q9H6T3	665	75717.9	6.81	RNA polyn	RPAP3	RPAP3	RNA polyn	5	103.3342	49.67913	4.29E-55	5	11.2782
P35244	121	13568.6	4.71	Replicatior	RPA3	RPA3	Replicatior	1	170.0523	46.04251	1.4E-42	1	9.917355
P27694	616	68137.7	7.24	Replicatior	RPA1	RPA1	Replicatior	4	229.5139	50.85647	1.73E-59	4	8.441558
Q8TA86	221	26106.5	10.52	Retinitis pi	RP9	RP9	Retinitis pi	2	183.9858	41.2587	6.89E-29	2	10.85973
O75695	350	39640.7	4.73	Protein XR	RP2	RP2	Protein XR	2	43.65909	46.34975	1.43E-43	2	3.428571
O75116	1388	160898.6	5.84	Rho-assoc	ROCK2	ROCK2	Rho-assoc	15	253.0965	49.75597	2.24E-55	12	11.95965
Q13464	1354	158173.5	5.67	Rho-assoc	ROCK1	ROCK1	Rho-assoc	2	169.7171	36.29519	3.67E-18	2	1.698671
P10155	538	60670.1	8.12	RNA-bind	RO60	RO60	RNA-bind	16	1424.866	54.04615	5.4E-72	16	35.68773
Q15287	305	34207.8	12.36	RNA-bind	RNPS1	RNPS1	RNA-bind	5	5330.644	48.93583	2.13E-52	5	23.27869
Q9H4A4	650	72595.1	5.52	Aminopep	RNPEP	RNPEP	Aminopep	3	93.48663	34.19915	1.07E-14	3	4.615385
Q96LT9	517	58574.6	7.82	RNA-bind	RNPC3	RNPC3	RNA-bind	11	357.4706	49.37953	5.36E-54	11	25.53191
O43148	476	54843.7	6.54	mRNA cap	RNMT	RNMT	mRNA cap	7	380.1949	49.05497	7.95E-53	7	17.43697
P13489	461	49972.8	4.44	Ribonuclea	RNH1	RNH1	Ribonuclea	13	1279.423	53.56805	4.68E-70	13	41.86551
O60942	597	68556.2	8.23	mRNA-cap	RNGTT	RNGTT	mRNA-cap	10	270.1788	53.53801	6.13E-70	10	18.42546
Q9Y252	685	78090.6	9.04	E3 ubiquiti	RNF6	RNF6	E3 ubiquiti	5	189.1192	50.70585	6.45E-59	5	9.489051
Q9H4P4	317	35904.9	6.08	E3 ubiquiti	RNF41	RNF41	E3 ubiquiti	1	82.09895	31.70866	2.12E-11	1	4.100946
O75150	1001	113677.5	6.19	E3 ubiquiti	RNF40	RNF40	E3 ubiquiti	6	137.2052	44.35605	2.22E-37	6	7.992008
P78317	190	21318.7	7.05	E3 ubiquiti	RNF4	RNF4	E3 ubiquiti	1	45.19643	19.68475	1.27E-05	1	8.947368
Q96EP0	1072	119650.8	6.54	E3 ubiquiti	RNF31	RNF31	E3 ubiquiti	2	135.8681	32.08886	8.71E-12	2	2.798507
Q5VTB9	566	62764.6	5.9	E3 ubiquiti	RNF220	RNF220	E3 ubiquiti	2	132.9761	48.15793	1.19E-49	2	4.240283
Q63HN8	5207	591402	6.46	E3 ubiquiti	RNF213	RNF213	E3 ubiquiti	6	118.3951	49.31151	9.49E-54	6	1.555598
Q5VTR2	975	113661.5	5.7	E3 ubiquiti	RNF20	RNF20	E3 ubiquiti	5	105.4153	39.02675	1.27E-23	4	6.25641
Q99496	336	37655	6.84	E3 ubiquiti	RNF2	RNF2	E3 ubiquiti	11	2076.418	49.9451	4.52E-56	10	41.07143
Q8NCN4	708	77193.5	9.54	E3 ubiquiti	RNF169	RNF169	E3 ubiquiti	7	367.7949	50.4557	5.65E-58	7	14.40678
Q9BV68	311	33861.2	5.17	E3 ubiquiti	RNF126	RNF126	E3 ubiquiti	3	147.4815	51.63645	1.8E-62	3	11.25402
Q9Y508	228	25694.1	7.28	E3 ubiquiti	RNF114	RNF114	E3 ubiquiti	4	303.3294	49.17907	2.86E-53	4	17.98246
O15541	343	38786.9	5.4	E3 ubiquiti	RNF113A	RNF113A	E3 ubiquiti	1	51.7296	43.25822	3.27E-34	1	2.915452
Q9Y3C5	154	17443.6	4.4	RING finge	RNF11	RNF11	RING finge	1	71.3056	36.04171	1.04E-17	1	5.844156
Q8N5U6	811	89926.2	6.92	E3 ubiquiti	RNF10	RNF10	E3 ubiquiti	4	77.52045	42.59213	2.24E-32	4	6.288533
O00584	256	29480.6	7.09	Ribonuclea	RNASET2	RNASET2	Ribonuclea	1	42.599	39.58448	7.14E-25	1	3.90625
O60930	286	32064	9.6	Ribonuclea	RNASEH1	RNASEH1	Ribonuclea	6	685.0807	51.8299	3.26E-63	6	30.06993
Q9H871	391	43992.2	5.97	E3 ubiquiti	RMND5A	RMND5A	E3 ubiquiti	2	75.91618	52.9162	1.84E-67	2	5.882353
Q9NWS8	449	51603.4	9	Required f	RMND1	RMND1	Required f	1	143.5392	39.14877	6.81E-24	1	2.004454
Q96TC7	470	52117.7	4.75	Regulator	RMDN3	RMDN3	Regulator	4	201.9354	49.96589	3.78E-56	4	12.76596
Q96DB5	314	35807.6	8.73	Regulator	RMDN1	RMDN1	Regulator	2	306.8576	46.02491	1.6E-42	2	6.050955
Q8N999	325	37489.8	7.07	RNA ligase	RLIG1	RLIG1	RNA ligase	4	232.1133	43.10532	8.73E-34	4	14.76923
Q13129	1914	217951.1	6.76	Zinc finger	RLF	RLF	Zinc finger	22	398.8665	56.26752	3.32E-81	22	14.05434
Q13546	671	75930.3	6.29	Receptor-i	RIPK1	RIPK1	Receptor-i	3	73.07364	41.31063	5.1E-29	3	7.153502
Q8IUF8	465	52799.8	6.68	Ribosomal	RIOX2	RIOX2	Ribosomal	5	177.7185	49.02153	1.05E-52	5	13.11828
Q9H6W3	641	71085	6.42	Ribosomal	RIOX1	RIOX1	Ribosomal	6	432.3345	51.46093	8.47E-62	6	14.19657
O14730	519	59092.6	5.53	Serine/thre	RIOK3	RIOK3	Serine/thre	2	215.6618	46.10886	8.62E-43	2	3.468208
Q9BVS4	552	63282.6	5.78	Serine/thre	RIOK2	RIOK2	Serine/thre	11	493.9808	53.22871	1.03E-68	11	26.99275
Q9BRS2	568	65582.5	6.08	Serine/thre	RIOK1	RIOK1	Serine/thre	7	606.3682	47.14773	3.35E-46	7	11.61972
Q6NUQ1	792	90630.7	5.12	RAD50-int	RINT1	RINT1	RAD50-int	1	65.26507	31.296	6.15E-11	1	1.515152
Q06587	406	42429	5.34	E3 ubiquiti	RING1	RING1	E3 ubiquiti	9	2688.761	49.9098	6.1E-56	9	25.36946
Q13671	783	84098	8.08	Ras and Ra	RIN1	RIN1	Ras and Ra	2	153.1185	41.13101	1.44E-28	2	2.93742
Q6R327	1708	192215.4	7.5	Rapamycir	RICTOR	RICTOR	Rapamycir	11	1069.053	48.62926	2.65E-51	11	7.259953
Q9NPQ8	531	59708.9	4.98	Synembryr	RIC8A	RIC8A;RIC8	Synembryr	1	83.71901	36.36065	2.79E-18	1	2.636535
Q8IUC4	686	76992.6	6.79	Rhophilin-	RHPN2	RHPN2	Rhophilin-	2	103.9454	47.03791	7.82E-46	2	3.35277
Q8IXI1	618	68117.2	5.69	Mitochonc	RHOT2	RHOT2	Mitochonc	4	278.188	47.68959	4.91E-48	3	10.19417
Q8IXI2	618	70783.5	6.21	Mitochonc	RHOT1	RHOT1	Mitochonc	4	195.9805	46.74	7.61E-45	4	6.472492
P84095	191	21308.3	8.21	Rho-relate	RHOG	RHOG	Rho-relate	3	255.1859	54.60088	2.98E-74	3	21.98953
Q9HBH0	211	23625.2	8.82	Rho-relate	RHOF	RHOF	Rho-relate	6	321.1418	56.10299	1.56E-80	6	34.12322
P08134	193	22006.2	6.52	Rho-relate	RHOC	RHOC	Rho-relate	6	939.3165	47.3545	6.79E-47	1	28.49741
O94955	611	69412.8	7.66	Rho-relate	RHOBTB3	RHOBTB3	Rho-relate	1	101.2534	31.8676	1.39E-11	1	1.636661
P61586	193	21767.9	5.89	Transformi	RHOA	RHOA	Transformi	3	129.7763	54.29746	5.3E-73	3	12.43523
Q15382	184	20497.3	5.73	GTP-bindi	RHEB	RHEB	GTP-bindi	4	221.9508	38.6165	9.81E-23	4	16.30435
O75783	438	48313.7	7.97	Rhomboid	RHBDL1	RHBDL1	Rhomboid	1	1761.007	33.91127	2.84E-14	1	5.936073
Q6PJF5	856	96685.2	8.98	Inactive rh	RHBDF2	RHBDF2	Inactive rh	1	123.5286	37.86946	3.53E-21	1	1.051402
P49796	1198	132334.4	6.21	Regulator	RGS3	RGS3	Regulator	1	102.7266	35.73303	3.59E-17	1	0.751252
Q92546	391	42454.9	5.84	RAB6A-GE	RGP1	RGP1	RAB6A-GE	2	80.70709	40.20514	2.58E-26	2	4.603581
P48378	723	79986.6	6.77	DNA-bind	RFX2	RFX2;RFX3	DNA-bind	1	108.921	37.91275	2.88E-21	1	0.968188
P40937	340	38496.1	7.22	Replicatior	RFC5	RFC5	Replicatior	13	1566.546	51.39872	1.47E-61	13	47.94118
P35249	363	39681.4	8.13	Replicatior	RFC4	RFC4	Replicatior	16	2203.577	49.82681	1.23E-55	16	46.00551
P40938	356	40555.9	8.42	Replicatior	RFC3	RFC3	Replicatior	15	1626.929	51.28883	3.89E-61	15	49.4382
P35250	354	39156.9	6.4	Replicatior	RFC2	RFC2	Replicatior	11	2301.613	51.03833	3.5E-60	11	44.0678
P35251	1148	128253.5	9.99	Replicatior	RFC1	RFC1	Replicatior	35	1501.986	54.97418	8.31E-76	35	35.27875
Q9GZR2	422	46671.5	10.5	RNA exonu	REXO4	REXO4	RNA exonu	7	181.6409	47.43383	3.65E-47	7	17.53555
Q9Y3B8	237	26832.5	6.88	Oligoribon	REXO2	REXO2	Oligoribon	1	122.9724	33.75666	4.76E-14	1	4.219409
Q8N1G1	1221	131508.1	9.28	RNA exonu	REXO1	REXO1	RNA exonu	1	115.531	47.59998	9.95E-48	1	1.064701
Q6NUM9	610	66818.9	8.39	All-trans-r	RETSAT	RETSAT	All-trans-r	4	88.496	46.43367	7.65E-44	4	8.688525
O15258	196	22957.8	9.91	Protein RE	RER1	RER1	Protein RE	3	219.6473	49.35341	6.68E-54	3	19.38776

Q96D71	796	86661.4	5.44	RalBP1-as:	REPS1	REPS1	RalBP1-as:	1	86.19791	22.94475	3.56E-06	1	1.633166
Q04206	551	60218.5	5.38	Transcripti	RELA	RELA	Transcripti	2	91.87883	41.06243	2.14E-28	2	3.811252
Q96HR9	211	23418	8.75	Receptor e	REEP6	REEP6	Receptor e	2	339.9021	46.13849	6.92E-43	2	10.90047
Q00765	189	21492.9	8.29	Receptor e	REEP5	REEP5	Receptor e	5	158.4294	43.06081	1.16E-33	5	20.63492
O94762	991	108857.4	8.69	ATP-deper	RECQL5	RECQL5	ATP-deper	3	95.52943	48.45315	1.11E-50	3	3.229062
O94761	1208	133065.9	8.12	ATP-deper	RECQL4	RECQL4	ATP-deper	1	32.63118	28.47844	2.05E-08	1	1.324503
P46063	649	73456.8	7.97	ATP-deper	RECQL	RECQL	ATP-deper	12	989.5164	49.89217	7.09E-56	12	21.41757
Q9HBH5	336	36864.4	9	Retinol del	RDH14	RDH14	Retinol del	1	157.2288	46.53565	3.56E-44	1	3.869048
Q8NBN7	331	35931.8	8.24	Retinol del	RDH13	RDH13	Retinol del	2	124.556	48.3939	1.78E-50	2	6.94864
Q8TC12	318	35386	9.06	Retinol del	RDH11	RDH11	Retinol del	3	242.6515	52.1176	2.5E-64	3	12.89308
Q8IZV5	341	38087.2	7.44	Retinol del	RDH10	RDH10	Retinol del	1	14.33489	19.60841	1.31E-05	1	2.346041
Q9P2K3	495	55581.1	8.56	REST corep	RCOR3	RCOR3	REST corep	1	88.04005	39.68201	4.27E-25	1	2.828283
Q9UKL0	485	53327	7.03	REST corep	RCOR1	RCOR1	REST corep	6	317.3657	50.85888	1.7E-59	3	14.43299
Q14257	317	36876.1	4.01	Reticulocal	RCN2	RCN2	Reticulocal	5	692.6339	49.76165	2.14E-55	5	19.87382
Q9Y2P8	373	40842.2	9.67	RNA 3-ter	RCL1	RCL1	RNA 3'-ter	12	2260.945	50.79572	2.94E-59	12	34.58445
Q9Y256	329	35832.3	8.01	CAAX prer	RCE1	RCE1	CAAX prer	1	130.1264	48.15654	1.2E-49	1	3.951368
Q9P258	522	56084.1	9	Protein RC	RCC2	RCC2	Protein RC	19	4677.206	50.6173	1.39E-58	19	47.12644
Q96I51	464	49897.4	8.36	RCC1-like	RCC1L	RCC1L	RCC1-like	4	90.42693	52.38226	2.28E-65	4	10.99138
P18754	421	44968.9	7.57	Regulator	RCC1	RCC1	Regulator	13	2970.771	49.25116	1.57E-53	13	41.5677
P62877	108	12273.9	6.98	E3 ubiquiti	RBX1	RBX1	E3 ubiquiti	2	307.5641	53.36205	3.09E-69	2	24.07407
Q06330	500	55636.7	7.2	Recombini	RBPJ	RBPJ	Recombini	9	506.7299	48.77138	8.26E-52	8	21.8
P09455	135	15850.1	4.74	Retinol-bir	RBP1	RBP1	Retinol-bir	4	185.6436	50.58603	1.83E-58	4	31.11111
Q96E39	390	42141.1	10.21	RNA bindi	RBMXL1	RBMXL1	RNA bindi	4	610.1626	48.65965	2.06E-51	4	9.230769
Q9Y388	322	37335.3	10.51	RNA-bind	RBMX2	RBMX2	RNA-bind	6	805.0443	52.43735	1E-65	6	19.25466
P38159	391	42331.4	10.39	RNA-bind	RBMX	RBMX	RNA-bind	12	36367.34	50.20502	4.87E-57	2	26.59847
Q15434	407	43958.4	9.39	RNA-bind	RBMS2	RBMS2	RNA-bind	3	142.4251	49.66666	4.76E-55	3	10.56511
P29558	406	44505.1	9.06	RNA-bind	RBMS1	RBMS1;RBI	RNA-bind	1	279.23	41.46734	2.04E-29	1	3.694581
Q9Y5S9	174	19888.9	5.43	RNA-bind	RBM8A	RBM8A	RNA-bind	4	1198.468	47.01704	9.16E-46	4	29.31034
Q9Y580	266	30503.3	9.98	RNA-bind	RBM7	RBM7	RNA-bind	5	510.4303	47.67824	5.38E-48	5	17.29323
P78332	1123	128643	6.23	RNA-bind	RBM6	RBM6	RNA-bind	27	721.6368	51.01833	4.17E-60	27	25.02226
P52756	815	92153	6.17	RNA-bind	RBM5	RBM5	RNA-bind	17	1674.107	49.27906	1.24E-53	14	21.59509
Q9BQ04	359	40149.3	6.74	RNA-bind	RBM4B	RBM4B;RB	RNA-bind	10	317.8017	52.72586	1.04E-66	10	27.0195
A0AV96	593	64098.6	7.76	RNA-bind	RBM47	RBM47	RNA-bind	5	188.9297	53.87839	2.62E-71	4	10.28668
Q8IUH3	476	53502.1	7.19	RNA-bind	RBM45	RBM45	RNA-bind	6	146.1322	49.29374	1.1E-53	6	13.86555
Q9BTD8	480	50413.5	10.31	RNA-bind	RBM42	RBM42	RNA-bind	3	213.5569	50.47701	4.71E-58	3	7.5
Q96IZ5	413	47099.5	9.08	RNA-bind	RBM41	RBM41	RNA-bind	1	65.01539	36.34094	3.03E-18	1	3.874092
Q14498	530	59379	10.75	RNA-bind	RBM39	RBM39	RNA-bind	14	4995.223	51.07869	2.45E-60	12	30.56604
P42696	430	48564.5	10.85	RNA-bind	RBM34	RBM34	RNA-bind	12	738.5417	55.56797	3.01E-78	12	26.27907
Q96EV2	1170	129985.3	6.93	RNA-bind	RBM33	RBM33	RNA-bind	6	191.4454	52.92516	1.69E-67	6	7.863248
P98179	157	17170.3	9.13	RNA-bind	RBM3	RBM3	RNA-bind	1	117.1021	43.60477	3.42E-35	1	6.369427
Q9NW13	759	85737.1	9.91	RNA-bind	RBM28	RBM28	RNA-bind	30	2165.623	52.39025	2.12E-65	30	39.13043
Q9P2N5	1060	118717.1	9.71	RNA-bind	RBM27	RBM27	RNA-bind	10	707.836	48.38335	1.94E-50	10	12.35849
Q5T8P6	1007	113596.3	9.7	RNA-bind	RBM26	RBM26	RNA-bind	13	295.4502	52.08021	3.47E-64	12	15.29295
P49756	843	100184.5	6.17	RNA-bind	RBM25	RBM25	RNA-bind	27	1610.234	52.79355	5.67E-67	27	23.72479
Q86U06	439	48730.2	10.4	Probable F	RBM23	RBM23	Probable F	2	92.7505	44.09066	1.34E-36	2	6.605923
Q9NW64	420	46895.2	8.67	Pre-mRNA	RBM22	RBM22	Pre-mRNA	9	660.8422	51.08276	2.37E-60	9	22.61905
Q9Y4C8	960	107331	6.5	Probable F	RBM19	RBM19	Probable F	19	628.0613	53.66976	1.85E-70	19	25.9375
Q96I25	401	44961.2	5.69	Splicing fa	RBM17	RBM17	Splicing fa	12	2385.569	50.35484	1.34E-57	12	31.9202
Q8NDT2	890	97204.5	10.35	Putative RI	RBM15B	RBM15B	Putative RI	12	270.0472	49.55135	1.27E-54	11	17.19101
Q96T37	977	107187.4	10.65	RNA-bind	RBM15	RBM15	RNA-bind	28	2081.364	51.4531	9.08E-62	28	34.28864
Q96PK6	669	69490.9	9.92	RNA-bind	RBM14	RBM14	RNA-bind	16	1591.296	52.65929	1.88E-66	16	26.4574
Q8IXT5	1001	118101.7	6.79	RNA-bind	RBM12B	RBM12B	RNA-bind	38	4356.66	53.61887	2.98E-70	38	43.35664
Q9NTZ6	932	97394.9	8.98	RNA-bind	RBM12	RBM12	RNA-bind	3	110.2173	43.93362	3.86E-36	3	3.004292
P98175	930	103531.9	5.8	RNA-bind	RBM10	RBM10	RNA-bind	16	2781.049	53.27712	6.67E-69	16	20
O43251	390	41373.5	7.31	RNA bindi	RBFOX2	RBFOX2;RI	RNA bindi	4	190.0288	55.86648	1.67E-79	2	10.25641
Q8N0V3	343	38359	7.95	Putative ril	RBFA	RBFA	Putative ril	2	349.1941	51.65962	1.46E-62	2	6.413994
Q16576	425	47819.7	4.68	Histone-bi	RBBP7	RBBP7	Histone-bi	3	293.4581	52.15712	1.74E-64	3	8
Q7Z6E9	1792	201562.7	10.27	E3 ubiquiti	RBBP6	RBBP6	E3 ubiquiti	25	866.3536	50.2425	3.53E-57	25	17.29911
Q15291	538	59152.1	4.71	Retinoblas	RBBP5	RBBP5	Retinoblas	9	463.0038	48.05788	2.65E-49	9	21.56134
Q09028	425	47655.3	4.5	Histone-bi	RBBP4	RBBP4	Histone-bi	11	3529.137	50.7365	4.93E-59	7	36.94118
Q9NYW8	714	82994.3	7.34	RB-associa	RBAK	RBAK	RB-associa	6	210.111	48.05444	2.72E-49	5	7.142857
P06400	928	106158.3	8.04	Retinoblas	RB1	RB1	Retinoblas	5	271.4952	47.0472	7.28E-46	5	7.758621
P50749	326	37789.9	9.17	Ras associa	RASSF2	RASSF2	Ras associa	3	64.6775	41.47741	1.93E-29	2	11.34969
P54136	660	75378.3	6.65	Arginine--	RARS1	RARS1	Arginine--	25	904.2499	51.55203	3.78E-62	25	39.84848
P61225	183	20504.2	4.44	Ras-relate	RAP2B	RAP2B	Ras-relate	2	479.7128	49.42611	3.63E-54	1	12.02186
P61224	184	20824.7	5.39	Ras-relate	RAP1B	RAP1B	Ras-relate	9	1355.479	51.87643	2.14E-63		46.19565
P62834	184	20987.1	6.55	Ras-relate	RAP1A	RAP1A	Ras-relate	2	261.7397	51.41692	1.25E-61	2	15.21739
P46060	587	63541.3	4.33	Ran GTPas	RANGAP1	RANGAP1	Ran GTPas	14	529.4852	48.59901	3.38E-51	14	30.15332
Q96S59	729	77846.7	6.78	Ran-bindi	RANBP9	RANBP9	Ran-bindi	6	554.0511	51.25447	5.26E-61	6	8.641975
O60518	1105	124712.3	4.63	Ran-bindi	RANBP6	RANBP6	Ran-bindi	1	98.44956	45.58831	3.85E-41	1	1.176471
P49792	3224	358196.4	6.1	E3 SUMO-	RANBP2	RANBP2	E3 SUMO-	20	352.2711	54.3662	2.8E-73	10	8.746898
Q6VN20	620	67256.2	6.76	Ran-bindi	RANBP10	RANBP10	Ran-bindi	11	712.5226	52.68102	1.55E-66	9	22.58065
P43487	201	23309.9	4.96	Ran-specif	RANBP1	RANBP1	Ran-specif	4	461.206	49.73169	2.75E-55	4	26.86567
P62826	216	24423	7.59	GTP-bindi	RAN	RAN	GTP-bindi	9	5076.355	49.0345	9.4E-53	9	38.88889
Q9UKM9	306	32462.9	9.66	RNA-bind	RALY	RALY	RNA-bind	9	6344.378	51.6633	1.41E-62	9	27.77778
P11234	206	23408.4	6.56	Ras-relate	RALB	RALB	Ras-relate	3	202.649	51.89212	1.87E-63	2	16.01942
P11233	206	23566.6	7.19	Ras-relate	RALA	RALA	Ras-relate	3	175.9585	49.29387	1.1E-53	3	14.07767
Q9P0K7	980	110040.4	6.14	Ankycorbir	RAI14	RAI14	Ankycorbir	7	158.6534	48.26842	4.91E-50	7	8.469388
Q7Z5J4	1906	203350.3	9	Retinoic ac	RAI1	RAI1	Retinoic ac	4	216.1992	48.23497	6.43E-50	4	3.147954
P78406	368	40967.8	7.9	mRNA exp	RAE1	RAE1	mRNA exp	6	148.219	44.05426	1.72E-36	6	20.92391
Q92878	1312	153891.1	6.87	DNA repai	RAD50	RAD50	DNA repai	2	96.12907	45.96787	2.42E-42	2	1.981707
P54727	409	43170.9	4.49	UV excisio	RAD23B	RAD23B	UV excisio	8	931.4534	54.83814	2.99E-75	7	22.00489
O60216	631	71688.9	4.28	Double-str	RAD21	RAD21	Double-str	8	138.5503	46.57988	2.55E-44	8	17.43265
Q9NS91	495	56222.1	7.63	E3 ubiquiti	RAD18	RAD18	E3 ubiquiti	1	131.4248	38.34253	3.73E-22	1	2.626263
O75943	681	77054	7.07	Cell cycle c	RAD17	RAD17	Cell cycle c	3	125.3682	45.08739	1.38E-39	3	5.580029
P63244	317	35076.5	7.76	Small ribos	RACK1	RACK1	Small ribos	13	5212.77	50.41896	7.76E-58	13	48.8959
Q9H0H5	632	71025.9	9.26	Rac GTPas	RACGAP1	RACGAP1	Rac GTPas	6	97.15987	49.0187	1.07E-52	6	12.81646
P15153	192	21428.6	7.7	Ras-relate	RAC2	RAC2	Ras-relate	1	29.33901	27.61576	7.46E-08	1	4.6875
P63000	192	21449.9	8.65	Ras-relate	RAC1	RAC1	Ras-relate	4	624.4382	48.9902	1.36E-52	1	22.91667
Q5HYI8	236	26422.6	7.13	Rab-like p	RABL3	RABL3	Rab-like p	2	282.0057	53.41365	1.91E-69	2	9.322034
P53611	331	36924	4.66	Geranylger	RABGGTB	RABGGTB	Geranylger	1	153.0687	41.4102	2.85E-29	1	3.323263
Q92696	567	65071.1	5.41	Geranylger	RABGGTA	RABGGTA	Geranylger	3	34.79235	29.66284	2.42E-09	3	5.996473
Q5R372	815	92512.1	4.96	Rab GTPas	RABGAP1L	RABGAP1L	Rab GTPas	1	24.84504	36.94588	2.3E-19	1	2.08589

Q9Y3P9	1069	121735.8	4.9	Rab GTPas	RABGAP1	RABGAP1	Rab GTPas	8	176.0637	47.80314	2.01E-48	7	7.67072
P51151	201	22837.4	5.1	Ras-relate	RAB9A	RAB9A	Ras-relate	3	256.9392	48.45605	1.08E-50	2	19.40299
P51149	207	23489.5	6.58	Ras-relate	RAB7A	RAB7A	Ras-relate	9	1719.073	52.37815	2.37E-65	9	57.48792
Q9NRW1	208	23461.5	5.14	Ras-relate	RAB6B	RAB6B	Ras-relate	1	29.87487	36.74363	5.51E-19	1	6.730769
P20340	208	23592.6	5.16	Ras-relate	RAB6A	RAB6A	Ras-relate	9	744.6289	51.03359	3.65E-60	4	47.11538
P51148	216	23482.4	8.61	Ras-relate	RAB5C	RAB5C	Ras-relate	2	382.9763	46.82603	3.97E-45	2	11.11111
P61020	215	23706.6	8.3	Ras-relate	RAB5B	RAB5B	Ras-relate	2	672.6907	44.64775	3E-38	2	12.09302
P20339	215	23658.5	8.31	Ras-relate	RAB5A	RAB5A	Ras-relate	7	1601.683	51.35086	2.25E-61	4	39.06977
P20338	218	24389.4	5.88	Ras-relate	RAB4A	RAB4A;RA	Ras-relate	1	76.09792	25.02869	1.07E-06	1	6.422018
Q9H2M9	1393	155983.3	5.34	Rab3 GTPa	RAB3GAP2	RAB3GAP2	Rab3 GTPa	5	89.72973	41.10739	1.65E-28	5	3.4458
P20337	219	24757.7	4.61	Ras-relate	RAB3B	RAB3B;RAI	Ras-relate	2	353.3147	47.71659	3.97E-48	1	11.87215
Q15286	201	23025.1	8.46	Ras-relate	RAB35	RAB35	Ras-relate	7	813.7745	52.51841	6.65E-66	7	30.84577
Q13636	195	21699.8	7.08	Ras-relate	RAB31	RAB31	Ras-relate	2	88.58232	43.11472	8.22E-34	1	12.30769
P61019	212	23545.4	6.51	Ras-relate	RAB2A	RAB2A	Ras-relate	10	614.1348	51.10049	2.03E-60	3	53.77358
O14966	203	23155.1	7.24	Ras-relate	RAB29	RAB29	Ras-relate	3	143.704	50.57241	2.06E-58	2	16.25616
P57735	213	23495.4	5.78	Ras-relate	RAB25	RAB25	Ras-relate	2	149.5806	50.58112	1.91E-58	2	10.32864
Q9ULC3	237	26659.1	6.54	Ras-relate	RAB23	RAB23	Ras-relate	1	89.50661	48.95088	1.88E-52	1	6.751055
Q9UL25	225	24347.4	8.05	Ras-relate	RAB21	RAB21	Ras-relate	5	354.6103	49.30121	1.03E-53	5	24.44444
Q9H0U4	201	22171	5.37	Ras-relate	RAB1B	RAB1B	Ras-relate	1	24.33165	44.34925	2.32E-37	1	7.462687
P62820	205	22677.6	6	Ras-relate	RAB1A	RAB1A	Ras-relate	6	3659.146	45.7823	9.42E-42	2	31.21951
Q9NP72	206	22976.9	4.86	Ras-relate	RAB18	RAB18	Ras-relate	5	694.2924	50.20384	4.92E-57	5	28.15534
P59190	212	24390.5	5.43	Ras-relate	RAB15	RAB15	Ras-relate	1	1084.108	43.03652	1.35E-33	1	5.188679
P61106	215	23896.8	6.13	Ras-relate	RAB14	RAB14	Ras-relate	7	477.0487	48.7637	8.79E-52	7	34.88372
P51153	203	22773.9	9.73	Ras-relate	RAB13	RAB13	Ras-relate	2	253.1091	52.01061	6.51E-64	2	12.31527
Q6IQ22	244	27248.1	8.58	Ras-relate	RAB12	RAB12	Ras-relate	4	145.1946	48.93327	2.17E-52	4	20.4918
Q9BXF6	653	70414.4	9.8	Rab11 fam	RAB11FIP5	RAB11FIP5	Rab11 fam	4	63.16132	47.46774	2.8E-47	4	10.56662
O75154	756	82439	4.16	Rab11 fam	RAB11FIP3	RAB11FIP3	Rab11 fam	1	64.20099	18.996	1.62E-05	1	1.322751
Q15907	218	24488.3	5.73	Ras-relate	RAB11B	RAB11B	Ras-relate	7	1006.797	51.26526	4.79E-61	1	35.3211
P61026	200	22540.7	8.61	Ras-relate	RAB10	RAB10	Ras-relate	3	312.8528	47.8168	1.8E-48	3	16.5
Q9Y3T6	440	49091.1	5.32	R3H and c	R3HCC1	R3HCC1	R3H and c	2	221.1491	47.85959	1.29E-48	2	4.318182
Q9H974	415	46712.4	6.81	Queuine tI	QTRT2	QTRT2	Queuine tI	2	73.7609	44.75737	1.4E-38	2	4.819277
Q9NXS2	382	42923.6	10.25	Glutaminyl	QPCTL	QPCTL	Glutaminyl	1	391.8674	45.70178	1.69E-41	1	2.879581
P47897	775	87798	7.16	Glutamine	QARS1	QARS1	Glutamine	24	907.701	51.36357	2.01E-61	24	37.54839
P06737	847	97147.8	7.17	Glycogen p	PYGL	PYGL	Glycogen p	6	173.5622	52.00079	7.13E-64	6	8.854782
P11216	843	96695.2	6.85	Glycogen p	PYGB	PYGB	Glycogen p	23	790.5331	53.24249	9.16E-69	18	34.1637
Q53H96	274	28663	7.78	Pyrroline-5	PYCR3	PYCR3	Pyrroline-5	2	110.9877	43.23181	3.88E-34	2	12.40876
Q96C36	320	33636.8	7.87	Pyrroline-5	PYCR2	PYCR2	Pyrroline-5	4	158.5971	45.27587	3.63E-40	4	21.875
P32322	319	33360.3	7.69	Pyrroline-5	PYCR1	PYCR1	Pyrroline-5	12	1383.285	50.66442	9.2E-59	11	45.14107
P49023	591	64504.8	6.13	Paxillin	PXN	PXN	Paxillin	1	10.23785	51.11599	1.77E-60	1	2.876481
Q96N64	755	81959.6	9.54	PWWP dor	PWWP2A	PWWP2A	PWWP dor	5	126.4547	47.03529	7.97E-46	5	7.94702
Q15269	919	102451.2	6.06	Periodic tr	PWP2	PWP2	Periodic tr	3	294.2907	48.52641	6.11E-51	3	3.699674
Q13610	501	55827.3	4.36	Periodic tr	PWP1	PWP1	Periodic tr	16	2979.24	51.87482	2.17E-63	16	47.50499
Q8N0Z8	303	33232.5	10.15	tRNA pseu	PUSL1	PUSL1	tRNA pseu	6	583.0541	50.36137	1.27E-57	6	24.42244
Q96PZ0	661	75034.9	6.31	Pseudouri	PUS7	PUS7	Pseudouri	9	173.6067	50.20124	5.02E-57	9	16.03631
Q9Y606	427	47469.7	8.54	Pseudouri	PUS1	PUS1	Pseudouri	7	665.9191	53.9325	1.58E-71	7	18.73536
Q96QR8	312	33240.6	5.06	Transcripti	PURB	PURB	Transcripti	3	158.6759	50.37875	1.1E-57	3	9.935897
Q00577	322	34910.6	6.39	Transcripti	PURA	PURA	Transcripti	3	807.17	48.21043	7.82E-50	3	12.73292
Q15397	648	73583.9	10.3	Pumilio ho	PUM3	PUM3	Pumilio ho	4	172.2254	47.96805	5.43E-49	4	6.481481
Q14671	1186	126472.4	6.83	Pumilio ho	PUM1	PUM1	Pumilio ho	3	71.74648	33.27022	2.3E-13	2	2.866779
Q9UHX1	559	59875	4.95	Poly(U)-bi	PUF60	PUF60	Poly(U)-bi	14	4470.478	50.01598	2.45E-56	14	25.93918
Q6GMV3	140	15805.3	9.52	Putative pe	PTRHD1	PTRHD1	Putative pe	8	806.7537	55.33742	2.57E-77	8	71.42857
Q9Y3E5	179	19193.4	8.97	Peptidyl-tI	PTRH2	PTRH2	Peptidyl-tI	2	133.6151	53.11969	2.85E-68	2	15.64246
Q86Y79	214	22936.4	11.14	Peptidyl-tI	PTRH1	PTRH1	Peptidyl-tI	5	418.5462	47.11435	4.34E-46	5	33.17757
Q92729	1446	162422.1	6.91	Receptor-t	PTPRU	PTPRU	Receptor-t	5	94.9104	50.0392	2.02E-56	5	4.356846
Q15262	1439	162100.9	5.75	Receptor-t	PTPRK	PTPRK	Receptor-t	5	228.027	49.87392	8.27E-56	4	4.725504
P23470	1445	162002.3	6.38	Receptor-t	PTPRG	PTPRG	Receptor-t	1	58.08711	29.95375	1.34E-09	1	1.107266
P10586	1907	212877.4	6.23	Receptor-t	PTPRF	PTPRF	Receptor-t	5	60.24866	52.62191	2.62E-66	3	3.828002
P23469	700	80641.2	7.02	Receptor-t	PTPRE	PTPRE	Receptor-t	2	177.2143	35.57767	6.61E-17	2	2.857143
P18433	802	90718.4	6.62	Receptor-t	PTPRA	PTPRA	Receptor-t	2	85.83904	49.31174	9.48E-54	2	3.117207
P29350	595	67560.8	7.88	Tyrosine-p	PTPN6	PTPN6	Tyrosine-p	7	191.595	47.95055	6.25E-49	7	14.28571
P26045	913	103989.1	7.04	Tyrosine-p	PTPN3	PTPN3	Tyrosine-p	6	110.3463	50.2387	3.65E-57	6	8.214677
Q9H3S7	1636	178971.9	6.91	Tyrosine-p	PTPN23	PTPN23	Tyrosine-p	4	117.2678	48.5396	5.49E-51	4	3.850856
P17706	415	48472.9	8.44	Tyrosine-p	PTPN2	PTPN2	Tyrosine-p	3	137.4008	42.1028	4.57E-31	3	7.46988
Q06124	593	68010.5	7.31	Tyrosine-p	PTPN11	PTPN11	Tyrosine-p	3	304.485	42.44078	5.75E-32	3	4.890388
P18031	435	49966.4	6.21	Tyrosine-p	PTPN1	PTPN1	Tyrosine-p	10	648.1247	49.00256	1.23E-52	10	28.73563
Q8WUK0	201	22843.4	10.24	Phosphatic	PTPMT1	PTPMT1	Phosphatic	3	58.82368	43.8745	5.73E-36	3	18.90547
Q93096	173	19814.9	9.25	Protein tyr	PTP4A1	PTP4A1	Protein tyr	1	45.96693	37.36519	3.57E-20	1	7.514451
Q13308	1070	118390.7	7.09	Inactive ty	PTK7	PTK7	Inactive ty	11	310.5067	51.09837	2.07E-60	11	16.26168
Q05397	1052	119232	6.61	Focal adhe	PTK2	PTK2	Focal adhe	5	208.1321	49.42322	3.72E-54	5	5.703422
Q8N4Q0	377	40140.1	8.32	Prostaglan	PTGR3	PTGR3	Prostaglan	3	141.244	41.15648	1.24E-28	3	10.34483
Q14914	329	35869.6	8.53	Prostaglan	PTGR1	PTGR1	Prostaglan	1	95.28627	35.42711	1.19E-16	1	6.990881
Q9P2B2	879	98555.6	6.58	Prostaglan	PTGFRN	PTGFRN	Prostaglan	4	111.454	42.95781	2.24E-33	4	5.005688
Q15185	160	18697.2	4.11	Prostaglan	PTGES3	PTGES3	Prostaglan	5	1882.23	51.3451	2.36E-61	5	32.5
Q9H7Z7	377	41942.8	9.5	Prostaglan	PTGES2	PTGES2	Prostaglan	5	250.5642	45.36997	1.86E-40	5	15.38462
O14684	152	17102.1	9.77	Prostaglan	PTGES	PTGES	Prostaglan	1	136.5841	40.45762	6.45E-27	1	6.578947
Q96BW5	349	39017.6	6.51	Phosphotr	PTER	PTER	Phosphotr	2	110.0756	46.60074	2.18E-44	2	6.303725
P48651	473	55527.2	8.52	Phosphatic	PTDSS1	PTDSS1	Phosphatic	4	250.1899	54.27102	6.78E-73	4	10.35941
Q96EY7	689	78548.9	6.38	Small ribos	PTCD3	PTCD3	Small ribos	20	6765.293	51.87648	2.14E-63	20	36.42961
O75127	700	78855	8.78	Pentatrico	PTCD1	PTCD1	Pentatrico	2	131.7122	40.50076	5.08E-27	2	2.714286
O95758	552	59689	9.46	Polypyrimi	PTBP3	PTBP3	Polypyrimi	7	478.0556	55.77521	4.01E-79	5	12.13768
Q9UKA9	531	57490.3	9.05	Polypyrimi	PTBP2	PTBP2	Polypyrimi	1	762.6258	39.4106	1.77E-24	1	1.506591
P26599	557	59632.6	9.67	Polypyrimi	PTBP1	PTBP1	Polypyrimi	10	2183.58	49.91676	5.75E-56	10	24.41652
Q8WXF1	523	58743.2	6.63	Paraspeckl	PSPC1	PSPC1	Paraspeckl	8	907.8454	57.23557	5.27E-85	8	19.12046
Q9BT73	122	13104.4	8.07	Proteasom	PSMG3	PSMG3	Proteasom	1	53.59884	39.34616	2.47E-24	1	12.29508
Q92530	271	29816.5	5.53	Proteasom	PSMF1	PSMF1	Proteasom	1	177.8435	42.14954	3.44E-31	1	4.428044
P61289	254	29505.8	5.76	Proteasom	PSME3	PSME3	Proteasom	3	168.5774	49.93619	4.87E-56	3	14.96063
Q9UL46	239	27401.4	5.47	Proteasom	PSME2	PSME2	Proteasom	6	175.5954	51.05194	3.1E-60	6	35.98326
Q06323	249	28722.9	5.83	Proteasom	PSME1	PSME1	Proteasom	9	336.1714	47.826	1.68E-48	9	31.72691
P48556	350	39611.5	10.19	26S protea	PSMD8	PSMD8	26S protea	6	285.9255	52.40407	1.87E-65	6	18.85714
P51665	324	37025.2	6.75	26S protea	PSMD7	PSMD7	26S protea	9	320.8364	49.55839	1.19E-54	9	34.25926
Q15008	389	45530.9	5.29	26S protea	PSMD6	PSMD6	26S protea	16	526.9242	52.05719	4.28E-64	16	36.24679
Q16401	504	56195.1	5.16	26S protea	PSMD5	PSMD5	26S protea	8	396.3232	51.48	7.14E-62	8	19.84127
P55036	377	40736.3	4.42	26S protea	PSMD4	PSMD4	26S protea	3	151.3101	48.45983	1.05E-50	3	8.753316

O43242	534	60977	8.75	26S protea	PSMD3	PSMD3	26S protea	18	323.2524	49.60455	8.06E-55	18	40.07491
Q13200	908	100198.8	4.84	26S protea	PSMD2	PSMD2	26S protea	21	373.5069	53.62129	2.93E-70	21	30.837
O00487	310	34576.9	6.51	26S protea	PSMD14	PSMD14	26S protea	2	820.4596	43.44418	9.78E-35	2	7.419355
Q9UNM6	376	42945.2	5.61	26S protea	PSMD13	PSMD13	26S protea	11	489.036	50.26245	2.97E-57	11	33.51064
O00232	456	52903.9	7.76	26S protea	PSMD12	PSMD12	26S protea	7	150.3577	51.67797	1.24E-62	7	17.98246
O00231	422	47463.2	6.42	26S protea	PSMD11	PSMD11	26S protea	13	1035.397	53.19175	1.45E-68	13	33.41232
O75832	226	24427.6	6.03	26S protea	PSMD10	PSMD10	26S protea	2	186.4995	44.26156	4.22E-37	2	7.522124
Q99460	953	105835.3	5.04	26S protea	PSMD1	PSMD1	26S protea	13	531.4231	51.36544	1.98E-61	13	17.20881
P62333	389	44172.6	7.56	26S protea	PSMC6	PSMC6	26S protea	5	149.5774	51.23576	6.19E-61	5	15.68123
P62195	406	45625.7	7.66	26S protea	PSMC5	PSMC5	26S protea	10	262.0992	51.89607	1.81E-63	10	32.26601
P43686	418	47365.8	4.84	26S protea	PSMC4	PSMC4	26S protea	9	462.4039	49.70278	3.51E-55	9	24.88038
P17980	439	49203.1	4.87	26S protea	PSMC3	PSMC3	26S protea	4	524.3468	46.33223	1.63E-43	4	10.25057
P35998	433	48633.4	5.65	26S protea	PSMC2	PSMC2	26S protea	12	955.3998	54.08286	3.79E-72	12	30.71594
P62191	440	49184.1	6.11	26S protea	PSMC1	PSMC1	26S protea	12	440.9989	53.00927	7.82E-68	12	26.59091
P28074	263	28480	6.94	Proteasom	PSMB5	PSMB5	Proteasom	3	56.81946	38.00135	1.9E-21	3	12.92776
P49721	201	22836	7.04	Proteasom	PSMB2	PSMB2	Proteasom	2	229.0122	46.96651	1.35E-45	2	9.452736
P20618	241	26489.1	8.29	Proteasom	PSMB1	PSMB1	Proteasom	2	210.6067	47.92474	7.67E-49	2	9.958506
O14818	248	27886.6	8.73	Proteasom	PSMA7	PSMA7	Proteasom	6	449.6097	50.85537	1.75E-59	3	33.06452
P60900	246	27399.2	6.74	Proteasom	PSMA6	PSMA6	Proteasom	4	181.1358	46.08203	1.05E-42	4	18.29268
P28066	241	26410.8	4.45	Proteasom	PSMA5	PSMA5	Proteasom	5	204.823	45.8845	4.47E-42	5	31.53527
P25789	261	29483.6	7.86	Proteasom	PSMA4	PSMA4	Proteasom	1	111.0087	44.69628	2.14E-38	1	3.831418
P25788	255	28433	4.97	Proteasom	PSMA3	PSMA3	Proteasom	3	123.978	51.95327	1.09E-63	3	12.15686
P25787	234	25898.4	7.55	Proteasom	PSMA2	PSMA2	Proteasom	2	103.7378	39.99056	8.25E-26	2	9.401709
P25786	263	29555.3	6.6	Proteasom	PSMA1	PSMA1	Proteasom	4	144.2556	46.75565	6.75E-45	4	19.01141
O75475	530	60103	9.86	PC4 and SI	PSIP1	PSIP1	PC4 and SI	12	1063.692	51.12189	1.68E-60	12	22.64151
P49768	467	52667.3	4.97	Presenilin-	PSEN1	PSEN1	Presenilin-	1	50.90107	28.48959	2.02E-08	1	4.068522
Q9Y617	370	40422.4	7.77	Phosphose	PSAT1	PSAT1	Phosphose	8	288.0359	49.29771	1.06E-53	8	28.91892
P07602	524	58112.1	4.82	Prosaposir	PSAP	PSAP	Prosaposir	8	499.2176	47.64233	7.13E-48	8	16.98473
Q16651	343	36431.1	5.72	Prostasin	PRSS8	PRSS8	Prostasin	1	20.2075	34.1563	1.24E-14	1	3.498542
P54821	245	27296.2	9.91	Paired mes	PRRX1	PRRX1;PRF	Paired mes	1	170.8376	47.59167	1.06E-47	1	5.306122
Q9Y520	2896	316908.9	9.73	Protein PR	PRRC2C	PRRC2C	Protein PR	16	459.6758	52.95811	1.25E-67	15	6.146409
Q5JSZ5	2229	242964.8	8.52	Protein PR	PRRC2B	PRRC2B	Protein PR	5	232.4229	50.95627	7.19E-60	5	2.69179
P48634	2157	228861.2	10	Protein PR	PRRC2A	PRRC2A	Protein PR	16	645.4849	56.20923	5.99E-81	16	11.21929
Q96M27	445	46700.8	5.64	Protein PR	PRRC1	PRRC1	Protein PR	2	162.0725	50.2048	4.88E-57	2	6.292135
Q9BWN1	585	64327.7	10.99	Proline-ric	PRR14	PRR14	Proline-ric	4	262.431	47.49855	2.2E-47	3	10.25641
Q96HE9	360	40084.6	10.87	Proline-ric	PRR11	PRR11	Proline-ric	1	116.7792	33.15001	3.34E-13	1	4.444444
Q14558	356	39393.3	7.22	Phosphoril	PRPSAP1	PRPSAP1	Phosphoril	1	109.6752	37.04149	1.51E-19	1	6.179775
P11908	318	34768.8	6.6	Ribose-ph	PRPS2	PRPS2	Ribose-ph	5	228.4759	47.98535	4.73E-49	2	22.95597
P60891	318	34833.9	6.98	Ribose-ph	PRPS1	PRPS1	Ribose-ph	2	77.72965	47.03334	8.09E-46	2	7.232704
Q6P2Q9	2335	273598.4	9.14	Pre-mRNA	PRPF8	PRPF8	Pre-mRNA	87	3945.932	53.45518	1.3E-69	87	39.65739
O94906	941	106924	8.4	Pre-mRNA	PRPF6	PRPF6	Pre-mRNA	39	4224.385	51.3584	2.11E-61	39	44.95218
Q6NWY9	871	99357.2	6.81	Pre-mRNA	PRPF40B	PRPF40B	Pre-mRNA	3	4241.693	43.09202	9.5E-34	3	3.444317
O75400	957	108804.2	7.64	Pre-mRNA	PRPF40A	PRPF40A	Pre-mRNA	23	6513.186	51.91549	1.52E-63	23	22.15256
O43172	522	58448.7	7.44	U4/U6 sma	PRPF4	PRPF4	U4/U6 sma	18	1456.83	49.92012	5.59E-56	18	38.69732
Q5VTL8	546	64467.4	11.22	Pre-mRNA	PRPF38B	PRPF38B	Pre-mRNA	11	2104.516	53.31338	4.81E-69	11	22.71062
Q8NAV1	312	37476.1	10.51	Pre-mRNA	PRPF38A	PRPF38A	Pre-mRNA	8	3118.685	50.5735	2.04E-58	8	18.58974
Q8WWY3	499	55455.6	5.51	U4/U6 sma	PRPF31	PRPF31	U4/U6 sma	15	583.6809	53.30141	5.36E-69	15	38.67735
O43395	683	77528.4	10.22	U4/U6 sma	PRPF3	PRPF3	U4/U6 sma	15	1255.385	55.44256	1E-77	15	25.32943
Q9UMS4	504	55180.3	6.6	Pre-mRNA	PRPF19	PRPF19	Pre-mRNA	11	1862.443	49.94257	4.62E-56	11	29.16667
Q99633	342	39859.4	8.4	Pre-mRNA	PRPF18	PRPF18	Pre-mRNA	1	168.4282	49.26983	1.34E-53	1	2.339181
Q13523	1007	116985.7	10.96	Serine/thre	PRP4K	PRP4K	Serine/thre	23	2032.268	50.44341	6.29E-58	23	24.32969
O15091	583	67314.8	9.02	Mitochonc	PRORP	PRORP	Mitochonc	4	88.78851	54.1068	3.04E-72	4	8.576329
Q9UNN8	238	26671.2	7.2	Endothelia	PROCR	PROCR	Endothelia	4	184.0405	48.39619	1.75E-50	4	20.58824
P04156	253	27661.2	9.23	Major prio	PRNP	PRNP	Major prio	3	260.1599	51.22293	6.92E-61	3	12.64822
O14744	637	72683.2	6.24	Protein arg	PRMT5	PRMT5	Protein arg	13	1911.28	49.10013	5.47E-53	13	25.27473
O60678	531	59902.7	5	Protein arg	PRMT3	PRMT3	Protein arg	1	37.80773	41.45941	2.14E-29	1	1.883239
Q99873	371	42461.3	4.99	Protein arg	PRMT1	PRMT1	Protein arg	16	1082.484	52.63861	2.25E-66	14	47.43935
O75569	313	34403.9	8.56	Interferon-	PRKRA	PRKRA	Interferon-	11	1782.207	51.93396	1.29E-63	11	38.33866
P78527	4128	469084.2	7.12	DNA-depe	PRKDC	PRKDC	DNA-depe	105	3126.167	56.79752	2.63E-83	105	30.69283
Q9BZL6	878	96721.3	6.84	Serine/thre	PRKD2	PRKD2	Serine/thre	13	375.5013	50.26041	3.02E-57	10	19.1344
P14314	528	59424.9	4.05	Glucosidas	PRKCSH	PRKCSH	Glucosidas	7	132.8519	52.43264	1.45E-65	7	14.20455
P41743	596	68261.9	5.68	Protein kin	PRKCI	PRKCI	Protein kin	3	389.2129	47.71865	3.91E-48	3	8.221477
P05129	697	78447.2	7.49	Protein kin	PRKCG	PRKCG	Protein kin	10	546.6628	50.1521	7.65E-57	10	19.22525
Q05655	676	77504.4	7.81	Protein kin	PRKCD	PRKCD	Protein kin	6	201.0152	44.33183	2.61E-37	5	10.65089
P17252	672	76749.4	7.05	Protein kin	PRKCA	PRKCA	Protein kin	12	345.3159	52.10876	2.69E-64	9	21.72619
P13861	404	45518	4.7	cAMP-dep	PRKAR2A	PRKAR2A	cAMP-dep	2	111.3367	36.15237	6.61E-18	1	6.683168
P10644	381	42981.3	5	cAMP-dep	PRKAR1A	PRKAR1A	cAMP-dep	5	577.2919	51.10659	1.93E-60	5	16.0105
P54619	331	37579.1	6.91	5-AMP-ac	PRKAG1	PRKAG1	5'-AMP-ac	5	196.4012	50.11823	1.02E-56	4	17.52266
P17612	351	40589.4	9.22	cAMP-dep	PRKACA	PRKACA	cAMP-dep	2	246.6039	42.24911	1.87E-31	1	5.982906
Q13131	559	64008.6	8.21	5-AMP-ac	PRKAA1	PRKAA1	5'-AMP-ac	6	471.4802	46.72171	8.74E-45	6	10.19678
P49643	509	58805.4	8.02	DNA prima	PRIM2	PRIM2	DNA prima	4	92.394	47.98862	4.61E-49	4	10.21611
O43900	615	68608.4	7.91	Prickle pla	PRICKLE3	PRICKLE3	Prickle pla	2	121.4111	51.63378	1.84E-62	2	5.203252
Q4J6C6	727	83926.3	6.34	Prolyl endo	PREPL	PREPL	Prolyl endo	1	92.87863	47.37773	5.66E-47	1	2.61348
P48147	710	80698.9	5.68	Prolyl endo	PREP	PREP	Prolyl endo	3	158.4153	49.55196	1.26E-54	3	5.070423
Q9HCU5	417	45467.8	7.95	Guanine n	PREB	PREB	Guanine n	4	325.2491	53.41188	1.94E-69	4	17.26619
P30041	224	25034.7	6.29	Peroxiredo	PRDX6	PRDX6	Peroxiredo	10	1797.443	50.44326	6.29E-58	10	44.64286
P30044	214	22086.2	8.97	Peroxiredo	PRDX5	PRDX5	Peroxiredo	5	569.7817	46.36341	1.29E-43	5	28.03738
Q13162	271	30539.6	6.24	Peroxiredo	PRDX4	PRDX4	Peroxiredo	4	393.5232	47.471	2.73E-47	4	19.9262
P30048	256	27692.4	7.88	Thioredoxi	PRDX3	PRDX3	Thioredoxi	6	711.623	51.28684	3.96E-61	6	37.10938
P32119	198	21891.7	5.75	Peroxiredo	PRDX2	PRDX2	Peroxiredo	5	850.4537	49.74991	2.35E-55	5	23.23232
Q06830	199	22110.2	8.31	Peroxiredo	PRDX1	PRDX1	Peroxiredo	13	17882.94	49.75714	2.22E-55	12	57.78894
O43663	620	71606	6.66	Protein reg	PRC1	PRC1	Protein reg	2	85.57019	39.45273	1.42E-24	2	2.419355
O60831	178	19257.6	9.39	PRA1 fami	PRAF2	PRAF2	PRA1 fami	2	175.335	46.0677	1.17E-42	2	12.35955
O60828	265	30471.9	6.26	Polyglutan	PQBP1	PQBP1	Polyglutan	2	410.2782	47.54842	1.49E-47	2	9.056604
Q96BP3	646	73574.4	7.16	Peptidylpr	PPWD1	PPWD1	Peptidylpr	12	157.6867	48.6594	2.07E-51	12	21.67183
Q8NI37	304	32645.4	4.77	Protein ph	PPTC7	PPTC7	Protein ph	3	32.78819	47.72135	1.29E-46	3	14.14474
P50897	306	34193.2	6.5	Palmitoyl-	PPT1	PPT1	Palmitoyl-	1	165.4093	40.15908	3.32E-26	1	4.901961
Q5H9R7	873	97668.1	4.23	Serine/thre	PPP6R3	PPP6R3	Serine/thre	6	192.215	41.14832	1.3E-28	6	8.247423
Q9UPN7	881	96722.8	4.18	Serine/thre	PPP6R1	PPP6R1	Serine/thre	2	97.18114	44.43785	1.26E-37	2	2.383655
O00743	305	35144	5.4	Serine/thre	PPP6C	PPP6C	Serine/thre	2	226.3065	51.64438	1.67E-62	2	8.52459
P53041	499	56878.2	6.21	Serine/thre	PPP5C	PPP5C	Serine/thre	4	92.23076	47.74259	3.24E-48	4	9.218437
Q5MIZ7	849	97457.3	4.6	Serine/thre	PPP4R3B	PPP4R3B;F	Serine/thre	1	68.85875	38.96697	1.71E-23	1	1.648999
P60510	307	35079.6	4.68	Serine/thre	PPP4C	PPP4C	Serine/thre	1	45.86674	42.5836	2.36E-32	1	3.583062

P63098	170	19299.8	4.4	Calcineurir	PPP3R1	PPP3R1	Calcineurir	2	118.7058	42.23185	2.08E-31	2	14.11765
Q08209	521	58687.3	5.66	Protein ph	PPP3CA	PPP3CA	Protein ph	8	810.312	48.64666	2.29E-51	5	18.04223
Q13362	524	61060.2	6.87	Serine/thre	PPP2R5C	PPP2R5C;F	Serine/thre	2	89.55664	44.81846	9.14E-39	1	4.389313
P63151	447	51691.6	6.11	Serine/thre	PPP2R2A	PPP2R2A	Serine/thre	9	1036.352	51.92565	1.39E-63	5	24.16107
P30153	589	65307.8	4.75	Serine/thre	PPP2R1A	PPP2R1A	Serine/thre	15	456.2608	55.11451	2.14E-76	13	31.23939
P62714	309	35574.8	5.08	Serine/thre	PPP2CB	PPP2CB	Serine/thre	6	244.3168	51.09719	2.09E-60	1	29.77346
P67775	309	35593.9	5.22	Serine/thre	PPP2CA	PPP2CA	Serine/thre	1	137.6154	47.64978	6.72E-48	1	4.20712
Q96SB3	817	89333.5	4.62	Neurabin-	PPP1R9B	PPP1R9B	Neurabin-	17	1016.201	51.96304	9.97E-64	15	26.19339
Q9ULJ8	1098	123341.2	4.73	Neurabin-	PPP1R9A	PPP1R9A	Neurabin-	3	84.12999	52.73474	9.6E-67	3	3.734062
Q12972	351	38478.5	7.42	Nuclear inl	PPP1R8	PPP1R8	Nuclear inl	2	94.14957	55.35906	2.16E-77	2	7.977208
Q6NYC8	613	67942	5.09	Phostensir	PPP1R18	PPP1R18	Phostensir	1	231.5309	47.01954	8.99E-46	1	2.773246
Q8TAE6	165	17842.7	4.81	Protein ph	PPP1R14C	PPP1R14C	Protein ph	1	110.2498	44.89789	5.24E-39	1	10.30303
Q8WUF5	828	89090.1	6.8	RelA-assoc	PPP1R13L	PPP1R13L	RelA-assoc	3	175.1623	48.46659	9.92E-51	3	3.019324
O14974	1030	115279.6	5.06	Protein ph	PPP1R12A	PPP1R12A	Protein ph	25	909.5669	51.83547	3.11E-63	24	27.18447
Q96QC0	940	99057.4	9.73	Serine/thre	PPP1R10	PPP1R10	Serine/thre	17	388.6002	52.6632	1.82E-66	17	22.12766
P36873	323	36983.4	6.5	Serine/thre	PPP1CC	PPP1CC	Serine/thre	4	741.6347	51.0117	4.42E-60	4	11.14551
P62140	327	37186.5	6.06	Serine/thre	PPP1CB	PPP1CB	Serine/thre	6	1558.034	48.32438	3.13E-50	6	20.18349
P62136	330	37511.7	6.25	Serine/thre	PPP1CA	PPP1CA	Serine/thre	19	1844.974	51.62308	2.02E-62	5	57.57576
Q9Y570	386	42315.1	5.84	Protein ph	PPME1	PPME1	Protein ph	7	1961.263	50.07731	1.46E-56	7	25.12953
O15355	546	59271.1	3.99	Protein ph	PPM1G	PPM1G	Protein ph	11	517.3101	52.65803	1.9E-66	11	24.72527
O15297	605	66674.6	9.27	Protein ph	PPM1D	PPM1D	Protein ph	2	145.5015	47.2975	1.06E-46	2	3.636364
O60437	1756	204745.3	5.3	Periplakin	PPL	PPL	Periplakin	39	438.35	52.35151	3E-65	39	24.37358
Q8WUA2	492	57224.6	5.71	Peptidyl-p	PPIL4	PPIL4	Peptidyl-p	11	882.9031	49.1517	3.58E-53	11	25
Q9H2H8	161	18154.5	6.78	Peptidyl-p	PPIL3	PPIL3	Peptidyl-p	3	181.861	47.88117	1.08E-48	3	22.36025
Q13356	520	58823.1	9.1	RING-type	PPIL2	PPIL2	RING-type	2	52.21387	39.96042	9.7E-26	2	4.230769
Q9Y3C6	166	18236.7	8.22	Peptidyl-p	PPIL1	PPIL1	Peptidyl-p	3	268.2314	52.10974	2.67E-64	3	21.08434
O43447	177	19208	8.22	Peptidyl-p	PPIH	PPIH	Peptidyl-p	6	348.7501	51.81572	3.69E-63	6	42.37288
Q13427	754	88616.5	11.01	Peptidyl-p	PPIG	PPIG	Peptidyl-p	14	2256.324	51.64259	1.7E-62	14	17.90451
Q9UNP9	301	33430.6	5.29	Peptidyl-p	PPIE	PPIE	Peptidyl-p	5	408.0325	49.03663	9.24E-53	5	17.27575
Q08752	370	40763.3	7.24	Peptidyl-p	PPID	PPID	Peptidyl-p	7	187.5043	51.49566	6.19E-62	7	19.72973
P23284	216	23742.4	10.07	Peptidyl-p	PPIB	PPIB	Peptidyl-p	8	860.451	51.40028	1.45E-61	8	41.2037
P62937	165	18012.4	7.97	Peptidyl-p	PPIA	PPIA	Peptidyl-p	9	7112.108	51.58068	2.8E-62	8	47.27273
Q8NEY8	458	52736.7	9.53	Periphilin-	PPHLN1	PPHLN1	Periphilin-	10	351.2656	48.65461	2.15E-51	10	22.27074
Q86W92	1011	114023.1	5.22	Liprin-beta	PPFIBP1	PPFIBP1;PF	Liprin-beta	1	368.0092	46.27121	2.58E-43	1	1.186944
Q13136	1202	135777.5	6.23	Liprin-alpł	PPFIA1	PPFIA1	Liprin-alpł	4	231.8751	48.59761	3.42E-51	4	4.326123
Q06203	517	57398.5	6.74	Amidopho	PPAT	PPAT	Amidopho	8	627.3742	52.64497	2.13E-66	8	17.79497
Q9NQ55	473	53193.6	10.82	Suppresso	PPAN	PPAN	Suppresso	8	539.0451	49.11266	4.94E-53	8	19.45032
Q9H2U2	334	37919.9	7.45	Inorganic p	PPA2	PPA2	Inorganic p	2	147.6277	56.87189	1.45E-83	2	6.886228
Q15181	289	32659.8	5.64	Inorganic p	PPA1	PPA1	Inorganic p	4	252.6166	49.66693	4.75E-55	4	19.37716
P0CG39	1038	117389.1	5.83	POTE anky	POTEJ	POTEJ	POTE anky	1	115.1814	41.19885	9.75E-29	1	2.2158
Q9NUX5	634	71441.4	6.72	Protection	POT1	POT1	Protection	4	118.773	46.53837	3.49E-44	4	8.044164
P16435	677	76689.1	5.28	NADPH--c	POR	POR	NADPH--c	12	487.2357	47.73494	3.44E-48	12	21.1226
O75817	140	15650.7	9.24	Ribonuclea	POP7	POP7	Ribonuclea	2	469.3736	46.62016	1.89E-44	2	24.28571
Q969H6	163	18819.4	7.31	Ribonuclea	POP5	POP5	Ribonuclea	2	128.5599	52.23212	8.75E-65	2	14.72393
O95707	220	25424.5	10.74	Ribonuclea	POP4	POP4	Ribonuclea	5	537.498	52.78589	6.06E-67	5	19.09091
Q99575	1024	114707.8	9.72	Ribonuclea	POP1	POP1	Ribonuclea	30	2575.622	51.28412	4.05E-61	30	36.13281
Q15165	354	39380.5	5.35	Serum par	PON2	PON2	Serum par	1	355.276	48.64904	2.25E-51	1	2.542373
Q8NAT1	580	66614.8	8.72	Protein O-	POMGNT2	POMGNT2	Protein O-	1	69.21405	29.4587	3.61E-09	1	1.551724
A8CG34	1229	125090.4	11.06	Nuclear en	POM121C	POM121C	Nuclear en	3	67.75589	44.72865	1.71E-38	1	3.417413
O00411	1230	138619.3	9.25	DNA-direc	POLRMT	POLRMT	DNA-direc	26	919.462	50.33522	1.59E-57	26	25.69106
O15318	223	25914.3	4.26	DNA-direc	POLR3G	POLR3G	DNA-direc	1	66.25394	40.0141	7.27E-26	1	6.278027
P05423	398	44395.5	6.97	DNA-direc	POLR3D	POLR3D	DNA-direc	1	60.06265	17.32126	2.9E-05	1	2.261307
O14802	1390	155640.2	8.62	DNA-direc	POLR3A	POLR3A	DNA-direc	3	43.35561	48.5023	7.43E-51	3	2.733813
P62875	67	7645	7.92	DNA-direc	POLR2L	POLR2L	DNA-direc	1	245.88	39.86611	1.61E-25	1	13.43284
P53803	58	7004.1	9.34	DNA-direc	POLR2K	POLR2K	DNA-direc	1	85.15687	26.18922	3.97E-07	1	31.03448
P52435	117	13293.2	5.68	DNA-direc	POLR2J	POLR2J;PC	DNA-direc	4	436.5038	52.23895	8.26E-65	1	30.76923
P36954	125	14523.1	4.79	DNA-direc	POLR2I	POLR2I	DNA-direc	4	166.2939	49.06801	7.14E-53	4	40.8
P52434	150	17143.1	4.27	DNA-direc	POLR2H	POLR2H	DNA-direc	6	548.1655	53.66011	2.02E-70	6	49.33333
P62487	172	19294.2	5.23	DNA-direc	POLR2G	POLR2G	DNA-direc	4	650.9247	49.68019	4.25E-55	4	25.5814
P19388	210	24551.1	5.76	DNA-direc	POLR2E	POLR2E	DNA-direc	6	563.04	52.60121	3.17E-66	6	36.66667
O15514	142	16311.1	4.46	DNA-direc	POLR2D	POLR2D	DNA-direc	2	431.2417	41.33961	4.31E-29	2	16.90141
P19387	275	31440.9	4.54	DNA-direc	POLR2C	POLR2C	DNA-direc	6	819.0165	52.75962	7.67E-67	6	28.72727
P30876	1174	133895.4	6.87	DNA-direc	POLR2B	POLR2B	DNA-direc	37	1703.47	53.50396	8.38E-70	37	35.51959
P24928	1970	217174.2	7.38	DNA-direc	POLR2A	POLR2A	DNA-direc	46	1999.204	55.794	3.39E-79	46	26.4467
O15446	510	54985.3	8.91	DNA-direc	POLR1G	POLR1G	DNA-direc	2	171.3788	42.10309	4.57E-31	2	5.098039
Q3B726	338	37432	6.98	DNA-direc	POLR1F	POLR1F	DNA-direc	2	104.7196	52.48309	9.14E-66	2	5.91716
O15160	346	39249.4	5.18	DNA-direc	POLR1C	POLR1C	DNA-direc	7	353.0507	48.19271	9.01E-50	7	25.72254
Q9H9Y6	1135	128228.4	7.88	DNA-direc	POLR1B	POLR1B	DNA-direc	3	91.70942	34.45213	4.39E-15	3	3.436123
O95602	1720	194809.6	7.03	DNA-direc	POLR1A	POLR1A	DNA-direc	4	167.0854	41.41886	2.71E-29	4	2.616279
Q9NP87	494	54815.1	8.5	DNA-direc	POLM	POLM	DNA-direc	2	79.85464	46.04281	1.4E-42	2	4.8583
Q9UHN1	485	54910.7	8.48	DNA polyr	POLG2	POLG2	DNA polyr	3	125.4949	44.5125	7.59E-38	3	6.804124
P54098	1239	139561.1	6.89	DNA polyr	POLG	POLG	DNA polyr	6	140.8685	40.70377	1.64E-27	6	6.2954
Q9BY77	421	46088.9	10.72	Polymeras	POLDIP3	POLDIP3	Polymeras	9	389.4246	49.09436	5.74E-53	9	28.50356
Q9Y2S7	368	42033	8.81	Polymeras	POLDIP2	POLDIP2	Polymeras	4	196.4176	40.01622	7.19E-26	4	11.41304
P49005	469	51289	5.28	DNA polyr	POLD2	POLD2	DNA polyr	2	97.41818	42.19178	2.66E-31	2	5.117271
P28340	1107	123629.9	7.03	DNA polyr	POLD1	POLD1	DNA polyr	3	178.5374	45.76325	1.08E-41	3	2.710027
P06746	335	38177.3	9.41	DNA polyr	POLB	POLB	DNA polyr	16	4029.847	49.80634	1.47E-55	16	44.47761
Q7Z3K3	1410	155342.6	7.42	Pogo trans	POGZ	POGZ	Pogo trans	29	3839.004	53.47023	1.12E-69	29	25.8156
Q9P215	609	69443.4	4.78	Pogo trans	POGK	POGK	Pogo trans	1	35.75553	26.36061	3.34E-07	1	1.313629
O00592	558	58634.7	5.18	Podocalyxi	PODXL	PODXL	Podocalyxi	4	176.6303	51.85824	2.52E-63	4	7.168459
Q8IY17	1375	150952.4	7.77	Patatin-lik	PNPLA6	PNPLA6	Patatin-lik	14	711.722	52.57911	3.87E-66	14	12.58182
P00491	289	32117.7	6.95	Purine nuc	PNP	PNP	Purine nuc	1	95.01217	23.09901	3.31E-06	1	3.460208
Q9NRX1	252	27924	10.45	RNA-bind	PNO1	PNO1	RNA-bind	5	612.8365	48.66657	1.95E-51	5	27.77778
Q9H307	717	81627.2	7.15	Pinin	PNN	PNN	Pinin	33	18352.42	52.44394	1.3E-65	33	42.53835
Q96T60	521	57075.9	8.61	Bifunctiona	PNKP	PNKP	Bifunctiona	26	7101.083	54.14709	2.09E-72	26	50.47985
Q8N490	385	42875.4	9.37	Probable h	PNKD	PNKD	Probable h	1	39.35247	37.97758	2.13E-21	1	3.896104
Q8TF01	805	92576.2	10.75	Arginine/s	PNISR	PNISR	Arginine/s	11	848.4188	50.85057	1.82E-59	11	11.80124
Q13670	270	28554.3	6.7	Putative pc	PMS2P11	PMS2P11	Putative pc	1	50.24373	40.18813	2.84E-26	1	5.185185
O75439	489	54365.6	6.82	Mitochonc	PMPCB	PMPCB	Mitochonc	7	165.6787	49.48197	2.27E-54	7	17.99591
Q10713	525	58252.4	6.91	Mitochonc	PMPCA	PMPCA	Mitochonc	2	118.6895	42.45869	5.14E-32	2	3.619048
P29590	882	97549.5	6.13	Protein PM	PML	PML	Protein PM	7	261.0423	46.01593	1.7E-42	7	8.730159
Q8IYS1	436	47775.7	5.7	Xaa-Arg d	PM20D2	PM20D2	Xaa-Arg d	4	114.958	38.03296	1.64E-21	4	10.77982
O15031	1838	205125.6	6.17	Plexin-B2	PLXNB2	PLXNB2	Plexin-B2	2	82.12003	40.92991	4.56E-28	2	1.142546

Q9NRY6	295	31648.1	6.63	Phospholip	PLSCR3	PLSCR3	Phospholip	1	78.91882	47.67801	5.38E-48	1	4.067797
O15162	318	35048.8	4.57	Phospholip	PLSCR1	PLSCR1	Phospholip	2	37.715	47.1733	2.75E-46	2	6.289308
P13797	630	70810.4	5.28	Plastin-3	PLS3	PLS3	Plastin-3	10	223.2866	52.02427	5.75E-64	4	17.30159
Q14651	629	70252.7	5.06	Plastin-1	PLS1	PLS1	Plastin-1	2	67.6432	43.52287	5.85E-35	2	5.564388
O43660	514	57193.5	9.61	Pleiotropic	PLRG1	PLRG1	Pleiotropic	10	188.5376	47.61137	9.1E-48	10	26.07004
O14495	311	35115.6	9.4	Phospholip	PLPP3	PLPP3	Phospholip	1	222.4413	40.33765	1.25E-26	1	3.536977
O43688	288	32573.4	8.44	Phospholip	PLPP2	PLPP2	Phospholip	3	224.8382	48.18786	9.36E-50	3	12.15278
Q04941	152	16690.5	7.3	Proteolipic	PLP2	PLP2	Proteolipic	2	600.7427	46.70779	9.72E-45	2	18.42105
O60568	738	84784.5	5.95	Multifuncti	PLOD3	PLOD3	Multifuncti	15	1352.915	52.67888	1.58E-66	15	23.44173
O00469	737	84685.1	6.69	Procollage	PLOD2	PLOD2	Procollage	8	290.4992	48.04417	2.95E-49	8	12.89009
Q02809	727	83549.5	6.94	Procollage	PLOD1	PLOD1	Procollage	19	823.104	53.06459	4.71E-68	19	30.26135
P53350	603	68254	9.19	Serine/thre	PLK1	PLK1	Serine/thre	3	78.26052	41.71326	4.77E-30	3	6.301824
O60664	434	47074.7	5.11	Perilipin-3	PLIN3	PLIN3	Perilipin-3	8	272.8579	50.87198	1.52E-59	8	28.34101
P00747	810	90568.4	7.25	Plasminog	PLG	PLG	Plasminog	1	130.8867	35.72711	3.67E-17	1	1.111111
Q7Z736	793	85316.3	7.86	Pleckstrin I	PLEKHH3	PLEKHH3	Pleckstrin I	5	179.8634	54.26017	7.33E-73	5	7.566204
Q9H8W4	249	27797.5	8.29	Pleckstrin I	PLEKHF2	PLEKHF2	Pleckstrin I	2	127.0878	50.41285	8.18E-58	2	8.433735
Q96S99	279	31194.2	8.3	Pleckstrin I	PLEKHF1	PLEKHF1	Pleckstrin I	1	337.0962	38.60095	1.06E-22	1	3.584229
Q6IQ23	1121	127133.7	9.78	Pleckstrin I	PLEKHA7	PLEKHA7	Pleckstrin I	1	81.62355	38.70196	6.43E-23	1	1.248885
Q9HAU0	1116	127463.2	7.58	Pleckstrin I	PLEKHA5	PLEKHA5	Pleckstrin I	1	197.5054	49.85336	9.85E-56	1	0.896057
Q9HB21	404	45553.2	8.78	Pleckstrin I	PLEKHA1	PLEKHA1	Pleckstrin I	3	174.2111	49.47331	2.44E-54	3	9.405941
Q15149	4684	531785.9	5.76	Plectin	PLEC	PLEC	Plectin	71	706.0461	52.83909	3.74E-67	66	17.91204
Q8IV08	490	54704.9	6.45	5-3 exonu	PLD3	PLD3	5'-3' exonu	4	509.127	46.54305	3.37E-44	4	8.163265
O14939	933	105986.1	7.68	Phospholip	PLD2	PLD2	Phospholip	7	371.3077	53.42558	1.71E-69	7	7.931404
Q13393	1074	124183.1	9.07	Phospholip	PLD1	PLD1	Phospholip	2	55.04472	37.42181	2.77E-20	2	2.327747
P16885	1265	147868.7	6.6	1-phospha	PLCG2	PLCG2	1-phospha	3	63.61169	36.32288	3.27E-18	3	2.766798
Q8N3E9	789	89257.5	6.97	1-phospha	PLCD3	PLCD3	1-phospha	16	515.2693	52.60391	3.1E-66	16	30.67174
P51178	756	85664.3	6.68	1-phospha	PLCD1	PLCD1	1-phospha	2	90.14762	45.88934	4.32E-42	2	3.571429
Q03405	335	36977.6	6.64	Urokinase	PLAUR	PLAUR	Urokinase	3	169.6341	49.79638	4.96E-45	3	12.23881
P00749	431	48523.1	8.48	Urokinase-	PLAU	PLAU	Urokinase-	4	143.3777	49.64725	5.6E-55	4	14.61717
P00750	562	62916.5	7.81	Tissue-typ	PLAT	PLAT	Tissue-typ	2	81.56682	47.47247	2.7E-47	2	4.626335
Q9Y263	795	87156.2	6.33	Phospholip	PLAA	PLAA	Phospholip	2	47.89629	46.43687	7.47E-44	2	3.27044
Q99569	1192	131867.2	9.19	Plakophilir	PKP4	PKP4	Plakophilir	13	380.8824	46.34047	1.54E-43	12	13.5906
Q9Y446	797	87081.1	9.7	Plakophilir	PKP3	PKP3	Plakophilir	25	560.2875	51.86001	2.48E-63	25	37.51568
Q99959	881	97414.1	9.73	Plakophilir	PKP2	PKP2	Plakophilir	2	205.9512	47.66374	6.03E-48	2	2.383655
Q6P5Z2	889	99419.8	8.56	Serine/thre	PKN3	PKN3	Serine/thre	1	63.25409	38.04818	1.52E-21	1	1.574803
Q16513	984	112033.5	6.22	Serine/thre	PKN2	PKN2	Serine/thre	6	241.673	49.49328	2.06E-54	6	7.825203
P14618	531	57936.4	7.94	Pyruvate k	PKM	PKM	Pyruvate k	24	4090.804	53.36788	2.94E-69	23	54.61394
Q5JRX3	1037	117411.8	6.91	Presequen	PITRM1	PITRM1	Presequen	1	116.879	31.75881	1.86E-11	1	1.639344
P48739	271	31539.9	6.87	Phosphatic	PITPNB	PITPNB	Phosphatic	5	120.1233	47.59316	1.05E-47	4	26.19926
Q9GZP4	211	24177.7	5.56	PITH doma	PITHD1	PITHD1	PITH doma	1	164.863	38.96952	1.69E-23	1	3.791469
Q9UG56	409	46671.3	9.8	Phosphatic	PISD	PISD	Phosphatic	2	148.7701	47.63202	7.73E-48	2	7.090465
O00625	290	32113.2	6.92	Pirin	PIR	PIR	Pirin	1	54.51094	37.49482	1.99E-20	1	4.137931
Q99755	562	62632.7	8.35	Phosphatic	PIP5K1A	PIP5K1A	Phosphatic	14	928.0735	54.20717	1.19E-72	5	26.69039
Q86T03	277	29469.3	9.01	Type 1 pho	PIP4P1	PIP4P1	Type 1 pho	3	98.56902	48.18137	9.87E-50	2	11.55235
Q8TBX8	421	47299.5	6.84	Phosphatic	PIP4K2C	PIP4K2C	Phosphatic	17	1406.396	50.90152	1.17E-59	17	43.46793
P78356	416	47377.6	7.37	Phosphatic	PIP4K2B	PIP4K2B	Phosphatic	12	523.7446	55.19182	1.01E-76	6	29.08654
P48426	406	46224.3	7	Phosphatic	PIP4K2A	PIP4K2A	Phosphatic	13	2421.603	48.83477	4.9E-52	13	28.57143
P12273	146	16572.3	8.18	Prolactin-i	PIP	PIP	Prolactin-i	3	58.83166	46.95453	1.48E-45	3	23.28767
Q9BXM7	581	62768.4	10.34	Serine/thre	PINK1	PINK1	Serine/thre	2	83.05588	44.88554	5.43E-36	2	7.012195
Q9Y237	131	13809.9	10.49	Peptidyl-p	PIN4	PIN4	Peptidyl-p	1	87.40636	39.19941	5.26E-24	1	9.160305
Q99570	1358	153102.1	7.17	Phosphoin	PIK3R4	PIK3R4	Phosphoin	2	50.69275	55.35824	2.16E-77	2	1.620029
O00443	1686	190677.8	8.1	Phosphatic	PIK3C2A	PIK3C2A	Phosphatic	7	147.1609	49.33144	8.03E-54	7	5.041518
Q9NWS0	290	32362.6	4.79	PIH1 doma	PIH1D1	PIH1D1	PIH1 doma	2	108.7704	43.57752	4.09E-35	2	10.34483
Q9H490	435	50051.2	7.82	Phosphatic	PIGU	PIGU	Phosphatic	1	1457.283	47.8726	1.16E-48	1	2.528736
Q92643	395	45251.4	6.09	GPI-ancho	PIGK	PIGK	GPI-ancho	1	58.08056	31.58942	2.9E-11	1	2.531646
Q13492	652	70753.9	8.1	Phosphatic	PICALM	PICALM	Phosphatic	7	236.7428	47.72563	3.7E-48	5	12.26994
O75925	651	71835.2	7.31	E3 SUMO-	PIAS1	PIAS1;PIAS	E3 SUMO-	1	105.4324	46.31957	1.8E-43	1	2.304147
P42356	2102	236828.1	7.05	Phosphatic	PI4KA	PI4KA	Phosphatic	4	79.26476	51.35443	2.18E-61	4	2.283539
Q9BTU6	479	54021.9	8.44	Phosphatic	PI4K2A	PI4K2A	Phosphatic	2	101.2802	45.84241	6.08E-42	2	5.010438
Q9P1Y6	1649	178664.1	9.28	PHD and R	PHRF1	PHRF1	PHD and R	22	2064.021	49.09555	5.68E-53	22	21.83141
Q86SQ0	1253	142156.7	7.45	Pleckstrin I	PHLDB2	PHLDB2	Pleckstrin I	5	225.4308	45.26156	4.16E-40	5	4.070231
Q8WV24	401	45016.1	10.52	Pleckstrin I	PHLDA1	PHLDA1	Pleckstrin I	1	224.2981	44.82008	9.03E-39	1	2.244389
Q8WWQ0	1821	206687.4	9.16	PH-interac	PHIP	PHIP	PH-interac	45	8316.358	51.73537	7.5E-63	45	30.86216
O43175	533	56650	6.7	D-3-phos	PHGDH	PHGDH	D-3-phos	15	2394.595	54.49599	8.04E-74	15	33.77111
Q9UPP1	1060	117863	8.99	Histone lys	PHF8	PHF8	Histone lys	11	219.1204	50.52462	3.12E-58	11	11.50943
Q8IWS0	365	41290.2	8.82	PHD finger	PHF6	PHF6	PHD finger	9	1678.081	51.89068	1.9E-63	9	29.86301
Q7RTV0	110	12405.3	8.48	PHD finger	PHF5A	PHF5A	PHD finger	4	311.027	48.12829	1.51E-49	4	48.18182
Q92576	2039	229479.7	6.95	PHD finger	PHF3	PHF3	PHD finger	25	753.2474	51.77855	5.09E-63	25	14.07553
Q9BUL5	403	43817.7	5.47	PHD finger	PHF23	PHF23	PHD finger	1	214.9601	39.61327	6.14E-25	1	3.225806
A8MW92	1017	115009.3	6.81	PHD finger	PHF20L1	PHF20L1	PHD finger	6	113.1574	50.681	7.98E-59	6	7.47296
O75151	1096	120773.9	9.72	Lysine-spe	PHF2	PHF2	Lysine-spe	7	260.8163	54.32674	4.06E-73	7	6.660584
Q96QT6	1004	109697.5	7.76	PHD finger	PHF12	PHF12	PHD finger	2	58.96722	45.83515	6.41E-42	2	2.49004
Q8WUB8	498	56050.4	6.59	PHD finger	PHF10	PHF10	PHD finger	1	135.2254	36.49302	1.6E-18	1	3.212851
O43189	567	62105.2	9.19	PHD finger	PHF1	PHF1	PHD finger	11	522.6982	48.62868	2.66E-51	11	21.51675
Q8N4B1	249	27215	9.19	Sesquiped	PHETA1	PHETA1	Sesquiped	1	116.5678	42.88154	3.63E-33	1	4.016064
Q8NDX5	983	106160.6	6.61	Polyhomeo	PHC3	PHC3	Polyhomeo	5	727.28	49.0216	1.05E-52	5	3.662258
Q8IXK0	858	90712.4	8.87	Polyhomeo	PHC2	PHC2	Polyhomeo	14	7189.188	48.98127	1.46E-52	12	18.64802
P78364	1004	105533	9.41	Polyhomeo	PHC1	PHC1	Polyhomeo	8	170.4175	52.05383	4.41E-64	8	8.366534
Q99623	299	33296.1	10.41	Prohibitin-	PHB2	PHB2	Prohibitin-	12	2848.631	53.06403	4.72E-68	12	43.81271
P35232	272	29803.8	5.46	Prohibitin	PHB1	PHB1	Prohibitin	14	3137.675	49.50541	1.86E-54	14	55.88235
Q9H814	394	44402.2	5.05	Phosphory	PHAX	PHAX	Phosphory	6	166.873	49.9842	3.23E-56	6	21.82741
Q32NB8	556	62729.7	9.11	CDP-diacy	PGS1	PGS1	CDP-diacy	1	78.64155	29.10329	7.02E-09	1	1.618705
O15173	223	23818.2	4.48	Membrane	PGRMC2	PGRMC2	Membrane	2	291.3055	51.45018	9.31E-62	2	14.79821
O00264	195	21670.9	4.3	Membrane	PGRMC1	PGRMC1	Membrane	5	409.6597	50.60985	1.49E-58	4	21.02564
P06401	933	98980	6.45	Progesterc	PGR	PGR	Progesterc	1	82.05872	38.69381	6.69E-23	1	1.071811
O95394	542	59851.5	6.21	Phosphoac	PGM3	PGM3	Phosphoac	3	144.3543	41.66521	6.35E-30	3	6.088561
Q6PCE3	622	70441.1	7.17	Glucose 1,6	PGM2L1	PGM2L1	Glucose 1,6	1	84.29317	43.09796	9.15E-34	1	1.768489
P36871	562	61448.6	6.72	Phosphogl	PGM1	PGM1	Phosphogl	2	74.54919	36.2859	3.81E-18	2	4.982206
O95336	258	27546.5	5.95	6-phospho	PGLS	PGLS	6-phospho	2	188.769	47.64119	7.2E-48	2	10.85271
P00558	417	44614.4	8.27	Phosphogl	PGK1	PGK1	Phosphogl	14	886.2679	50.74554	4.56E-59	12	38.60911
P52209	483	53139.6	7.25	6-phospho	PGD	PGD	6-phospho	7	277.3472	48.32414	3.14E-50	7	20.70393
Q96HS1	289	32004.1	8.89	Serine/thre	PGAM5	PGAM5	Serine/thre	13	4293.045	49.94687	4.45E-56	13	55.0173
P15259	253	28766	9.32	Phosphogl	PGAM2	PGAM2;PG	Phosphogl	2	201.2455	49.43891	3.26E-54	1	9.881423

P35080	140	15046.2	7.04	Profilin-2	PFN2	PFN2	Profilin-2	2	546.8046	48.90671	2.7E-52	2	20
P07737	140	15054.1	8.46	Profilin-1	PFN1	PFN1	Profilin-1	6	298.1919	51.31065	3.2E-61	6	57.85714
Q01813	784	85595.4	7.6	ATP-deper	PFKP	PFKP	ATP-deper	17	832.8444	50.70829	6.31E-59	17	23.97959
P08237	780	85181.9	8.07	ATP-deper	PFKM	PFKM	ATP-deper	8	145.4133	50.83228	2.14E-59	8	14.10256
P17858	780	85017.8	7.54	ATP-deper	PFKL	PFKL	ATP-deper	10	334.7379	54.22779	9.85E-73	7	13.84615
Q16875	520	59608.6	8.29	6-phosphc	PFKFB3	PFKFB3	6-phosphc	2	102.6338	41.35753	3.88E-29	1	4.807692
O15212	129	14582.6	9.45	Prefoldin s	PFDN6	PFDN6	Prefoldin s	1	117.3169	43.25991	3.24E-34	1	9.302326
Q9NQP4	134	15314.1	4.15	Prefoldin s	PFDN4	PFDN4	Prefoldin s	2	46.92086	33.64728	6.83E-14	2	19.40299
Q9UHV9	154	16647.7	6.55	Prefoldin s	PFDN2	PFDN2	Prefoldin s	2	160.0111	50.16397	6.9E-57	2	16.88312
O15067	1338	144733.2	5.55	Phosphoril	PFAS	PFAS	Phosphoril	3	65.07069	33.93551	2.62E-14	3	2.989537
Q13608	980	104059.8	6.29	Peroxisom	PEX6	PEX6	Peroxisom	1	77.60987	43.49043	7.23E-35	1	1.734694
P50542	639	70864	4.16	Peroxisom	PEX5	PEX5	Peroxisom	4	138.3432	37.10995	1.12E-19	4	7.981221
P56589	373	42139.4	8.28	Peroxisom	PEX3	PEX3	Peroxisom	1	116.5847	42.88329	3.59E-33	1	2.680965
O75381	377	41236.3	4.59	Peroxisom	PEX14	PEX14	Peroxisom	4	427.7031	51.52742	4.69E-62	4	16.18037
O96011	259	28430.9	10.32	Peroxisom	PEX11B	PEX11B	Peroxisom	4	113.7578	41.0606	2.16E-28	4	18.14672
O00541	588	68002	7.37	Pescadillo	PES1	PES1	Pescadillo	16	965.1635	55.30519	3.51E-77	16	25.68027
P12955	493	54547.8	5.9	Xaa-Pro di	PEPD	PEPD	Xaa-Pro di	1	48.3822	34.40026	5.28E-15	1	2.028398
Q8IZL8	1130	119698.2	3.99	Proline-, g	PELP1	PELP1	Proline-, g	15	949.378	51.2619	4.93E-61	15	19.73451
Q9BRX2	385	43358.8	6.3	Protein pe	PELO	PELO	Protein pe	6	763.5553	50.92699	9.31E-60	6	17.14286
A5PLL7	270	31134.7	6.83	Plasmanyle	PEDS1	PEDS1	Plasmanyle	1	128.3265	29.122	6.79E-09	1	5.555556
Q9BY49	303	32544.1	9.12	Peroxisom	PECR	PECR	Peroxisom	1	52.49278	30.51241	3.98E-10	1	4.620462
P30086	187	21056.6	7.65	Phosphatic	PEBP1	PEBP1	Phosphatic	1	105.2379	18.47316	1.95E-05	1	7.486631
Q96GD0	296	31697.7	6.51	Chronophi	PDXP	PDXP	Chronophi	1	32.26969	34.94898	7.26E-16	1	4.391892
Q6P996	788	86706.1	5.05	Pyridoxal-c	PDXDC1	PDXDC1	Pyridoxal-c	6	198.9526	53.65808	2.06E-70	4	12.43655
Q9NTI5	1447	164665.8	8.69	Sister chro	PDS5B	PDS5B	Sister chro	7	118.9371	46.7175	9.03E-45	7	6.219765
Q29RF7	1337	150828.6	7.99	Sister chro	PDS5A	PDS5A	Sister chro	5	156.3195	41.04752	2.33E-28	5	4.33807
Q8NCN5	879	99363.8	6.31	Pyruvate d	PDPR	PDPR	Pyruvate d	6	247.3461	49.80402	1.5E-55	6	8.191126
Q9P0J1	537	61053.6	6.66	[Pyruvate c	PDP1	PDP1	[Pyruvate c	12	649.0355	51.42419	1.17E-61	12	31.84358
Q9NR12	457	49844.3	8.49	PDZ and L	PDLIM7	PDLIM7	PDZ and L	12	1146.616	51.7025	1.01E-62	12	35.22976
Q96HC4	596	63944.6	8.26	PDZ and L	PDLIM5	PDLIM5	PDZ and L	7	351.0564	52.28613	5.43E-65	7	14.7651
Q15120	406	46938.5	8.61	[Pyruvate c	PDK3	PDK3	[Pyruvate c	3	183.0851	45.4277	1.23E-40	3	8.62069
Q15084	440	48120.9	4.7	Protein dis	PDIA6	PDIA6	Protein dis	11	1320.014	51.08746	2.27E-60	11	37.27273
Q14554	519	59594	8	Protein dis	PDIA5	PDIA5	Protein dis	8	261.11	51.0918	2.19E-60	8	19.07514
P13667	645	72931.9	4.7	Protein dis	PDIA4	PDIA4	Protein dis	22	1108.559	52.78939	5.87E-67	22	35.34884
P30101	505	56781.8	6.28	Protein dis	PDIA3	PDIA3	Protein dis	17	810.2736	54.38138	2.45E-73	17	41.18812
O00330	501	54121.8	9.09	Pyruvate d	PDHX	PDHX	Pyruvate d	3	100.5294	33.10925	3.8E-13	3	6.986028
P11177	359	39233.1	6.63	Pyruvate d	PDHB	PDHB	Pyruvate d	3	194.8952	47.10745	4.58E-46	3	9.192201
P08559	390	43295.3	8.14	Pyruvate d	PDHA1	PDHA1	Pyruvate d	5	366.333	47.06725	6.23E-46	4	13.58974
Q9HBH1	243	27013.2	9.27	Peptide de	PDF	PDF	Peptide de	2	226.0354	41.53887	1.34E-29	2	9.465021
Q6L8Q7	609	67350.9	6.54	2,5-phosp	PDE12	PDE12	2',5'-phosp	5	300.6584	50.35425	1.35E-57	5	10.67323
Q8N8D1	485	54699.4	10.58	Programm	PDCD7	PDCD7	Programm	6	319.862	51.63373	1.84E-62	6	16.08247
Q8WUM4	868	96022.3	6.46	Programm	PDCD6IP	PDCD6IP	Programm	20	1054.91	51.1767	1.04E-60	20	23.27189
O75340	191	21868.3	4.97	Programm	PDCD6	PDCD6	Programm	4	348.8438	50.72699	5.35E-59	4	22.51309
Q53EL6	469	51734.8	4.83	Programm	PDCD4	PDCD4	Programm	10	1389.767	49.95056	4.31E-56	10	23.66738
Q9BRP1	358	39416.3	4.47	Programm	PDCD2L	PDCD2L	Programm	2	248.9562	48.69836	1.5E-51	2	7.541899
Q16342	344	38591.8	5.06	Programm	PDCD2	PDCD2	Programm	4	155.9876	53.56236	4.92E-70	4	14.82558
Q14690	1871	208699.3	9.29	Protein RR	PDCD11	PDCD11	Protein RR	30	887.5978	52.44513	1.29E-65	30	18.86692
Q9BUL8	212	24701.4	8.71	Programm	PDCD10	PDCD10	Programm	3	129.9204	46.44526	7.02E-44	3	16.50943
Q13442	181	20629.9	9.53	28 kDa hea	PDAP1	PDAP1	28 kDa hea	1	120.1881	19.87486	1.19E-05	1	7.18232
P49585	367	41730.7	7.29	Choline-pl	PCYT1A	PCYT1A	Choline-pl	1	18.56859	28.47006	2.08E-08	1	4.087193
Q9UHG3	505	56639.7	6.1	Prenylcyste	PCYOX1	PCYOX1	Prenylcyste	5	317.3116	46.61445	1.97E-44	5	11.28713
Q9H6A9	2034	222036.2	6.62	Pecanex-li	PCNX3	PCNX3	Pecanex-li	1	28.50453	28.03748	4.08E-08	1	0.688299
Q8WW12	178	18924.7	7.79	PEST prote	PCNP	PCNP	PEST prote	2	231.3693	50.34557	1.46E-57	2	13.48315
P12004	261	28768.5	4.31	Proliferatir	PCNA	PCNA	Proliferatir	7	1129.586	51.62029	2.07E-62	7	33.71648
Q96MG8	357	40674.9	5.35	Protein-L-	PCMTD1	PCMTD1	Protein-L-	5	119.0761	46.69944	1.04E-44	5	14.0056
P22061	227	24636.2	7.25	Protein-L-	PCMT1	PCMT1	Protein-L-	8	1632.273	50.36263	1.26E-57	8	39.64758
Q15154	2024	228558.4	4.67	Pericentric	PCM1	PCM1	Pericentric	1	97.15289	42.00799	8.13E-31	1	0.543478
Q16822	640	70698.4	7.67	Phosphoer	PCK2	PCK2	Phosphoer	14	532.7681	48.9237	2.35E-52	13	24.53125
Q5JVF3	399	46029.5	8.68	PCI domair	PCID2	PCID2	PCI domair	4	310.1581	48.81257	5.88E-52	4	11.02757
Q9BYE7	350	39047	4.63	Polycomb	PCGF6	PCGF6	Polycomb	2	63.43308	48.08409	2.14E-49	2	10.28571
P35227	344	37787.7	8.04	Polycomb	PCGF2	PCGF2	Polycomb	5	1616.248	49.76568	3.2E-53	4	14.53488
O94913	1555	173049.3	8.76	Pre-mRNA	PCF11	PCF11	Pre-mRNA	2	115.8104	35.05152	4.96E-16	2	1.414791
P05166	539	58215.1	7.69	Propionyl-	PCCB	PCCB	Propionyl-	2	59.45378	30.43151	4.78E-10	2	5.380334
P57723	403	41481.3	8.28	Poly(rC)-b	PCBP4	PCBP4	Poly(rC)-b	1	371.0374	37.46697	2.25E-20	1	3.473945
Q15366	365	38579.7	6.77	Poly(rC)-b	PCBP2	PCBP2	Poly(rC)-b	9	3898.091	52.43776	1.38E-65	7	29.86301
Q15365	356	37497.5	7.11	Poly(rC)-b	PCBP1	PCBP1	Poly(rC)-b	8	4875.342	49.34623	7.09E-54	8	35.39326
P11498	1178	129632.6	6.83	Pyruvate c	PC	PC	Pyruvate c	5	153.1279	52.84143	3.67E-67	5	5.093379
Q96AQ6	731	80642.8	4.99	Pre-B-cell	PBXIP1	PBXIP1	Pre-B-cell	14	1107.838	52.2448	7.88E-65	14	21.88782
Q86U86	1689	192946.1	6.88	Protein po	PBRM1	PBRM1	Protein po	32	562.9595	55.95785	6.55E-80	32	22.14328
Q96KB5	322	36084.7	4.75	Lymphokir	PBK	PBK	Lymphokir	1	48.13963	34.72323	1.66E-15	1	4.037267
Q9BVG4	233	26056.6	4.43	Protein PB	PBDC1	PBDC1	Protein PB	1	119.5933	19.81173	1.22E-05	1	4.291845
Q9BUH6	204	21639.2	5.11	Protein PA	PAXX	PAXX	Protein PA	1	167.6796	50.97683	6.01E-60	1	5.882353
Q6ZW49	1069	121341	6.69	PAX-intera	PAXIP1	PAXIP1	PAX-intera	1	33.04837	41.32797	4.61E-29	1	0.748363
Q9Y5B6	917	104802.8	5.37	PAX3- and	PAXBP1	PAXBP1	PAX3- and	24	838.0635	52.50044	7.83E-66	24	34.02399
Q96IZ0	340	36567.2	5.05	PRKC apop	PAWR	PAWR	PRKC apop	2	52.1296	37.84885	3.88E-21	2	8.529412
Q9HBE1	687	74059.3	8.43	POZ-, AT h	PATZ1	PATZ1	POZ-, AT h	3	127.4355	43.8689	5.95E-36	3	5.094614
Q9NVD7	372	42243.4	5.74	Alpha-par	PARVA	PARVA	Alpha-par	2	114.6725	44.16199	8.29E-37	2	7.526882
Q7L3T8	475	53262.2	8.17	Probable p	PARS2	PARS2	Probable p	2	135.8934	40.70513	1.63E-27	2	4
Q8IXQ6	854	96342.7	7.99	Protein mc	PARP9	PARP9	Protein mc	4	104.8985	48.26904	4.89E-50	4	6.557377
Q9UKK3	1724	192592.9	5.39	Protein mc	PARP4	PARP4	Protein mc	14	335.8561	50.55539	2.39E-58	14	9.570766
Q9UGN5	583	66205.3	9.22	Poly [ADP-	PARP2	PARP2	Poly [ADP-	15	2043.765	51.83339	3.16E-63	15	31.56089
Q9H0J9	701	79063.1	8.61	Protein mc	PARP12	PARP12	Protein mc	5	105.2408	45.08168	1.44E-39	5	9.843081
P09874	1014	113082.9	9.34	Poly [ADP-	PARP1	PARP1	Poly [ADP-	69	55539.34	52.77341	6.77E-67	69	66.07495
Q86W56	976	111109.5	6.39	Poly(ADP-	PARG	PARG	Poly(ADP-	4	68.6905	42.59443	2.21E-32	4	5.020492
Q8TEW0	1356	151422	7.75	Partitionin	PARD3	PARD3	Partitionin	10	215.4556	48.4265	1.38E-50	10	8.99705
O95340	614	69500.2	8.13	Bifunction	PAPSS2	PAPSS2	Bifunction	4	463.8136	51.47573	7.41E-62	3	8.143322
O43252	624	70832.7	6.85	Bifunction	PAPSS1	PAPSS1	Bifunction	10	432.8234	51.64826	1.62E-62	10	18.58974
P51003	745	82841.8	7.4	Poly(A) po	PAPOLA	PAPOLA	Poly(A) po	6	215.0845	40.80025	9.51E-28	2	8.456376
Q9NVE7	773	85990.1	6.22	4-phosphc	PANK4	PANK4	4'-phosph	2	103.9458	38.78179	4.33E-23	2	4.527814
Q8N3R9	675	77293	6.05	Protein PA	PALS1	PALS1	Protein PA	1	48.41854	26.7554	2.18E-07	1	1.777778
Q9Y2D5	1103	122069.9	4.71	PALM2-AK	PALM2AK/	PALM2AK/	PALM2-AK	3	58.00866	47.83121	1.61E-48	3	3.717135
O75781	387	42075.6	4.64	Paralemmi	PALM	PALM	Paralemmi	2	40.10282	55.80309	3.13E-79	2	9.302326
Q8WX93	1383	150562.4	7.1	Palladin	PALLD	PALLD	Palladin	8	231.4476	49.20002	2.4E-53	8	7.809111

Q9ULE6	856	96753.7	6.51	Paladin	PALD1	PALD1	Paladin	1	127.5028	32.87912	7.68E-13	1	0.934579
O96013	591	64071.5	10.35	Serine/thre	PAK4	PAK4	Serine/thre	1	228.2203	19.443	1.39E-05	1	2.199662
Q13177	524	58042.1	5.76	Serine/thre	PAK2	PAK2	Serine/thre	2	160.6783	50.17978	6.04E-57	1	3.625954
Q9NWT1	392	43963.5	9.35	p21-activa	PAK1IP1	PAK1IP1	p21-activa	10	487.651	47.95253	6.15E-49	10	29.08163
P22234	425	47078.8	7.26	Bifunctiona	PAICS	PAICS	Bifunctiona	15	981.9037	49.74731	2.4E-55	15	35.76471
Q15102	231	25734.1	6.84	Platelet-ac	PAFAH1B3	PAFAH1B3	Platelet-ac	4	145.6944	43.04684	1.27E-33	4	18.61472
P43034	410	46637.7	7.4	Platelet-ac	PAFAH1B1	PAFAH1B1	Platelet-ac	2	27.75894	25.91942	5.13E-07	2	5.853659
Q8N7H5	531	59975.2	4.25	RNA polyn	PAF1	PAF1	RNA polyn	14	1835.176	52.74933	8.39E-67	14	28.81356
Q9Y2J8	665	75563.3	5.28	Protein-ar	PADI2	PADI2	Protein-ar	2	26944.01	41.30227	5.35E-29	2	4.06015
Q9UKS6	424	48486.5	6.09	Protein kin	PACSIN3	PACSIN3	Protein kin	8	235.3875	52.26801	6.36E-65	8	22.16981
Q86U42	306	32748.8	4.73	Polyadeny	PABPN1	PABPN1	Polyadeny	4	497.5999	51.3648	1.99E-61	4	10.13072
Q13310	644	70782.3	9.75	Polyadeny	PABPC4	PABPC4	Polyadeny	8	1148.586	55.70123	8.28E-79	7	14.44099
P11940	636	70670.4	10	Polyadeny	PABPC1	PABPC1	Polyadeny	19	1870.667	51.91983	1.46E-63	5	30.66038
P07237	508	57115.8	4.49	Protein dis	P4HB	P4HB	Protein dis	23	1737.14	51.05721	2.97E-60	23	46.65354
O15460	535	60901.4	5.43	Prolyl 4-hy	P4HA2	P4HA2	Prolyl 4-hy	11	414.6088	53.69577	1.45E-70	11	25.04673
P13674	534	61048.8	5.84	Prolyl 4-hy	P4HA1	P4HA1	Prolyl 4-hy	13	1079.92	52.81707	4.57E-67	13	30.14981
Q32P28	736	83393.2	4.79	Prolyl 3-hy	P3H1	P3H1	Prolyl 3-hy	6	133.7868	50.98314	5.69E-60	6	9.782609
Q99572	595	68584.3	8.25	P2X purinc	P2RX7	P2RX7	P2X purinc	3	90.85712	53.02807	6.59E-68	3	6.05042
O95747	527	58021.7	6.37	Serine/thre	OXSR1	OXSR1	Serine/thre	4	118.9877	54.22457	1.01E-72	4	8.918406
Q9NWU1	459	48842.4	7.72	3-oxoacyl-	OXSM	OXSM	3-oxoacyl-	1	48.66601	41.61605	8.5E-30	1	3.267974
Q15070	435	48519.3	9.89	Mitochonc	OXA1L	OXA1L	Mitochonc	2	203.0483	48.07794	2.25E-49	2	4.367816
P32242	354	37327	9.67	Homeobox	OTX1	OTX1	Homeobox	1	141.3412	30.81408	1.99E-10	1	3.389831
Q9NUU6	356	42195.6	9.68	Inactive ub	OTULINL	OTULINL	Inactive ub	1	188.3127	38.05061	1.51E-21	1	2.247191
Q96BN8	352	40262.6	5.13	Ubiquitin t	OTULIN	OTULIN	Ubiquitin t	1	65.13617	43.57362	4.2E-35	1	3.409091
Q8N6M0	293	33812.4	5.93	Deubiquiti	OTUD6B	OTUD6B	Deubiquiti	1	106.4185	37.82725	4.29E-21	1	4.43686
Q01804	1114	124043.6	6.69	OTU doma	OTUD4	OTUD4	OTU doma	4	270.1665	50.2139	4.52E-57	4	4.308797
Q5T2D3	398	45123.8	8.48	OTU doma	OTUD3	OTUD3	OTU doma	7	142.5057	49.66702	4.75E-55	7	20.85427
Q96FW1	271	31283.8	4.58	Ubiquitin t	OTUB1	OTUB1	Ubiquitin t	6	1362.832	49.22515	1.95E-53	6	29.15129
Q9NRP0	149	16829.1	9.4	Oligosacch	OSTC	OSTC	Oligosacch	1	340.2491	54.28648	5.86E-73	1	8.053691
Q96SU4	736	83184	6.11	Oxysterol-	OSBPL9	OSBPL9	Oxysterol-	7	93.36253	46.50412	4.51E-44	7	11.82065
Q9BZF1	889	101194.7	6.95	Oxysterol-	OSBPL8	OSBPL8	Oxysterol-	6	127.8412	52.99271	9.1E-68	5	8.098988
Q9BZF3	934	106305.1	6.92	Oxysterol-	OSBPL6	OSBPL6	Oxysterol-	2	121.0335	48.54063	5.45E-51	2	2.35546
Q9H4L5	887	101222.7	6.87	Oxysterol-	OSBPL3	OSBPL3	Oxysterol-	8	193.3796	57.18258	8.5E-85	8	11.27396
Q9H1P3	480	55200.9	6.31	Oxysterol-	OSBPL2	OSBPL2	Oxysterol-	1	237.5137	28.17407	3.32E-08	1	2.708333
Q9BXB4	747	83642.7	7.06	Oxysterol-	OSBPL11	OSBPL11	Oxysterol-	3	96.77708	42.94254	2.46E-33	3	5.087015
Q9BXB5	764	83969.4	8.42	Oxysterol-	OSBPL10	OSBPL10	Oxysterol-	1	19.41833	38.14183	9.77E-22	1	1.439791
P22059	807	89419.9	7.31	Oxysterol-	OSBP	OSBP	Oxysterol-	2	96.79342	32.76609	1.08E-12	2	5.700124
Q13438	667	75561	4.51	Protein OS	OS9	OS9	Protein OS	4	204.1207	38.48763	1.84E-22	4	6.446777
O43913	435	50282.6	7.81	Origin rec	ORC5	ORC5	Origin rec	5	250.7077	44.20536	6.19E-37	5	13.10345
O43929	436	50377	8.11	Origin rec	ORC4	ORC4	Origin rec	3	59.01028	51.24614	5.65E-61	3	8.256881
Q9UBD5	711	82253.3	7.65	Origin rec	ORC3	ORC3	Origin rec	6	150.7803	48.71287	1.34E-51	6	9.423347
Q13416	577	65971	6.48	Origin rec	ORC2	ORC2	Origin rec	6	235.464	50.80476	2.72E-59	6	12.99827
Q13415	861	97348.7	9.67	Origin rec	ORC1	ORC1	Origin rec	20	1011.595	51.62672	1.96E-62	20	28.80372
O60313	960	111629.8	7.99	Dynamin-l	OPA1	OPA1	Dynamin-l	10	311.1965	52.44505	1.29E-65	10	13.125
Q9NTK5	396	44743.2	7.99	Obg-like A	OLA1	OLA1	Obg-like A	5	668.6836	47.73602	3.41E-48	5	14.14141
O15294	1046	116923.5	6.68	UDP-N-ac	OGT	OGT	UDP-N-ac	17	741.2392	57.13268	1.33E-84	17	19.69407
Q9NZT2	677	73324.1	4.48	Opioid grc	OGFR	OGFR	Opioid grc	6	299.3091	44.48464	9.18E-38	6	11.66913
Q02218	1023	115934.4	6.86	2-oxogluta	OGDH	OGDH	2-oxogluta	14	691.0905	52.1129	2.6E-64	11	18.08407
O60502	916	102914.2	4.54	Protein O-	OGA	OGA	Protein O-	1	283.9726	46.80378	4.72E-45	1	1.310044
Q5SWX8	454	51102.7	5.78	Protein od	ODR4	ODR4	Protein od	2	108.061	43.63983	2.71E-35	2	6.60793
Q01968	901	104203.8	6.51	Inositol po	OCRL	OCRL	Inositol po	5	173.7128	47.00033	1.04E-45	5	5.882353
Q56VL3	154	16953.4	9.36	OCIA dom	OCIAD2	OCIAD2	OCIA dom	6	288.6977	50.87209	1.52E-59	6	38.31169
Q9NX40	245	27625.9	7.59	OCIA dom	OCIAD1	OCIAD1	OCIA dom	5	242.5064	49.21758	2.35E-46	5	28.97959
P04181	439	48534.4	7.05	Ornithine a	OAT	OAT	Ornithine a	9	289.832	54.22978	9.74E-73	9	26.42369
Q15646	514	59225.5	7.96	2-5-oligoa	OASL	OASL	2'-5'-oligo	19	4028.007	49.76856	2.02E-55	19	44.16342
Q9Y6K5	1087	121169.1	8.47	2-5-oligoa	OAS3	OAS3	2'-5'-oligo	11	381.1998	50.96019	6.96E-60	11	11.95952
Q6DKJ4	435	48391.6	4.6	Nucleored	NXN	NXN	Nucleored	4	108.3731	42.91825	2.87E-33	4	10.8046
Q9UBU9	619	70181.6	8.7	Nuclear RN	NXF1	NXF1	Nuclear RN	10	431.8069	47.57497	1.21E-47	10	20.35541
O15381	856	95049.9	6.41	Nuclear va	NVL	NVL	Nuclear va	11	217.4648	47.90617	8.88E-49	11	17.05607
Q9BXS6	441	49451.5	10.62	Nucleolar a	NUSAP1	NUSAP1	Nucleolar a	4	194.8871	51.69698	1.05E-62	4	11.56463
P52948	1817	197577.7	6.37	Nuclear pc	NUP98	NUP98	Nuclear pc	15	432.8097	46.22951	3.52E-43	15	9.411117
Q8N1F7	819	93487.4	5.44	Nuclear pc	NUP93	NUP93	Nuclear pc	14	349.0225	52.61436	2.81E-66	14	18.31502
Q99567	741	83541.1	5.46	Nuclear pc	NUP88	NUP88	Nuclear pc	4	153.6663	46.85466	3.19E-45	4	6.072874
Q9BW27	656	75018.5	5.26	Nuclear pc	NUP85	NUP85	Nuclear pc	10	522.1111	49.41714	3.91E-54	10	18.44512
P37198	522	53254.4	4.96	Nuclear pc	NUP62	NUP62	Nuclear pc	4	143.3162	53.00578	8.06E-68	4	9.195402
Q7Z3B4	507	55435.2	7.03	Nucleopor	NUP54	NUP54	Nucleopor	4	154.3755	50.393	9.7E-58	4	10.25641
Q9UKX7	468	50143.8	7.08	Nuclear pc	NUP50	NUP50	Nuclear pc	2	74.30614	47.28377	1.17E-46	2	5.982906
O15504	423	44871.2	9.73	Nucleopor	NUP42	NUP42	Nucleopor	4	224.3753	46.97007	1.32E-45	4	12.29314
Q8NFH4	326	36707.4	5.82	Nucleopor	NUP37	NUP37	Nucleopor	3	108.5201	46.41805	8.61E-44	3	13.19018
Q8NFH5	326	34773.5	9.49	Nucleopor	NUP35	NUP35	Nucleopor	2	70.75507	49.82125	1.29E-55	2	9.202454
P35658	2090	213617.6	7.52	Nuclear pc	NUP214	NUP214	Nuclear pc	5	92.13425	28.62882	1.6E-08	5	3.636364
Q8TEM1	1887	205109.5	6.8	Nuclear pc	NUP210	NUP210	Nuclear pc	12	450.1288	50.57134	2.08E-58	12	7.631161
Q92621	2012	227919.2	6.11	Nuclear pc	NUP205	NUP205	Nuclear pc	17	362.4088	49.78882	1.7E-55	17	9.343936
Q5SRE5	1749	196041	6.71	Nucleopor	NUP188	NUP188	Nucleopor	4	111.277	46.53885	3.48E-44	4	2.801601
Q12769	1436	162119.7	5.2	Nuclear pc	NUP160	NUP160	Nuclear pc	11	432.9022	52.90115	2.11E-67	11	9.261838
O75694	1391	155197.6	6.09	Nuclear pc	NUP155	NUP155	Nuclear pc	13	367.655	51.41836	1.24E-61	13	11.57441
P49790	1475	153937	8.98	Nuclear pc	NUP153	NUP153	Nuclear pc	4	97.92962	42.02759	7.22E-31	4	3.186441
Q8WUM0	1156	128977.6	4.73	Nuclear pc	NUP133	NUP133	Nuclear pc	6	247.2228	54.32254	4.19E-73	6	5.536332
P57740	925	106373.2	5.11	Nuclear pc	NUP107	NUP107	Nuclear pc	7	266.7266	52.56373	4.44E-66	7	8.432432
Q9Y6R0	609	64890.9	9.08	Numb-like	NUMBL	NUMBL	Numb-like	2	264.735	37.86369	3.62E-21	2	3.448276
P49757	651	70803.3	8.66	Protein nu	NUMB	NUMB	Protein nu	3	127.2303	42.70837	1.08E-32	2	5.529954
Q14980	2115	238257.6	5.54	Nuclear m	NUMA1	NUMA1	Nuclear m	91	4367.585	53.06813	4.56E-68	91	47.75414
Q7Z417	695	76120.9	9.17	FMR1-inte	NUFIP2	NUFIP2	FMR1-inte	20	1615.018	49.88303	7.66E-56	20	31.36691
Q9UHK0	495	56299.2	9.66	FMR1-inte	NUFIP1	NUFIP1	FMR1-inte	7	670.3939	50.49862	3.92E-58	7	14.74747
Q8WV74	236	25370	8.5	Mitochonc	NUDT8	NUDT8	Mitochonc	2	34.6636	45.37817	1.75E-40	2	9.322034
Q9NZJ9	180	20305.9	6.28	Diphospho	NUDT4	NUDT4	Diphospho	2	150.3197	44.54109	6.25E-38	1	13.33333
O43809	227	26227.1	9.18	Cleavage a	NUDT21	NUDT21	Cleavage a	12	8295.818	55.21682	8.19E-77	12	54.62555
P50583	147	16829.1	5.06	Bis(5-nucl	NUDT2	NUDT2	Bis(5'-nucl	1	360.9481	44.18798	6.96E-37	1	5.442177
Q9BRJ7	211	23338.1	9.21	Tudor-inte	NUDT16L1	NUDT16L1	Tudor-inte	8	446.9351	48.40851	1.59E-50	7	45.49763
Q96RS6	583	66755.2	4.74	NudC dom	NUDCD1	NUDCD1	NudC dom	10	463.2852	47.61311	8.98E-48	10	21.9554
Q9Y266	331	38242.7	5.03	Nuclear m	NUDC	NUDC	Nuclear m	9	474.983	56.15822	9.27E-81	9	29.30514
Q9H1E3	243	27296.2	4.71	Nuclear ub	NUCKS1	NUCKS1	Nuclear ub	1	115.1091	50.53898	2.76E-58	1	4.526749
Q9Y5Y2	271	28825.1	5.63	Cytosolic F	NUBP2	NUBP2	Cytosolic F	3	179.8959	48.52719	6.07E-51	3	12.91513

Q9Y5A7	615	70537.5	5.77	NEDD8 ult	NUB1	NUB1	NEDD8 ult	3	107.7742	42.07192	5.52E-31	3	6.341463
Q9BSD7	190	20712.9	10.15	Cancer-rel	NTPCR	NTPCR	Cancer-rel	5	324.4037	52.15367	1.8E-64	5	35.26316
O95631	604	67747.6	8.87	Netrin-1	NTN1	NTN1	Netrin-1	1	16.79125	32.44281	2.79E-12	1	1.15894
Q9BV86	223	25386.8	5.16	N-termina	NTMT1	NTMT1	N-termina	3	137.1724	48.97834	1.5E-52	3	17.04036
P78549	312	34389.3	10.23	Endonucle	NTHL1	NTHL1	Endonucle	7	1851.33	56.38855	1.09E-81	7	30.12821
P21589	574	63367.3	7.04	5-nucleoti	NT5E	NT5E	5'-nucleot	18	1841.842	51.18281	9.87E-61	18	36.06272
Q9H857	520	60718.1	6.76	5-nucleoti	NT5DC2	NT5DC2	5'-nucleot	4	277.3287	45.70505	1.65E-41	4	8.076923
Q5TFE4	455	51844.4	6.3	5-nucleoti	NT5DC1	NT5DC1	5'-nucleot	1	240.3235	45.30111	3.04E-40	1	2.417582
Q9H0P0	336	37947.8	7.15	Cytosolic 5	NT5C3A	NT5C3A	Cytosolic 5	1	147.9758	40.35566	1.13E-26	1	4.761905
P49902	561	64969.2	6.05	Cytosolic p	NT5C2	NT5C2	Cytosolic p	6	204.1333	50.94841	7.7E-60	6	13.90374
Q96P11	429	46691.2	8.73	28S rRNA (	NSUN5	NSUN5	28S rRNA (	4	157.1349	52.37536	2.42E-65	3	10.25641
Q96CB9	384	43088.2	8.26	5-methylc	NSUN4	NSUN4	5-methylc	7	1216.076	52.13316	2.17E-64	7	20.3125
Q08J23	767	86470	6.76	RNA cytos	NSUN2	NSUN2	RNA cytos	23	739.2433	51.39578	1.51E-61	23	32.20339
Q9H0G5	558	66389.7	9.36	Nuclear sp	NSRP1	NSRP1	Nuclear sp	9	270.1131	49.54093	1.38E-54	9	15.94982
Q9NXX6	385	44300.9	5.04	Non-struc	NSMCE4A	NSMCE4A	Non-struc	5	327.6729	51.33586	2.56E-61	4	13.76623
Q96MG7	304	34307.7	9.8	Non-struc	NSMCE3	NSMCE3	Non-struc	4	171.8263	34.55426	3.05E-15	4	12.82895
Q96MF7	247	27932.3	7.85	E3 SUMO-	NSMCE2	NSMCE2	E3 SUMO-	1	56.98589	39.69218	4.05E-25	1	4.048583
Q8WV22	266	30855.2	7.53	Non-struc	NSMCE1	NSMCE1	Non-struc	1	94.92857	47.32398	8.59E-47	1	3.759398
Q96IY1	281	32161.6	6.78	Kinetochor	NSL1	NSL1	Kinetochor	2	156.5423	49.59803	8.51E-55	2	11.3879
Q9UNZ2	370	40572.5	4.73	NSFL1 cofa	NSFL1C	NSFL1C	NSFL1 cofa	2	70.37849	24.69734	1.35E-06	2	7.567568
P46459	744	82593.6	6.95	Vesicle-fus	NSF	NSF	Vesicle-fus	18	913.239	51.66625	1.38E-62	18	28.22581
Q15738	373	41900	8.2	Sterol-4-a	NSDHL	NSDHL	Sterol-4-a	4	108.3658	47.15142	3.26E-46	4	15.0134
Q9BZ95	1437	161611.8	8.26	Histone-ly	NSD3	NSD3	Histone-ly	28	825.1992	52.41444	1.71E-65	28	22.82533
O96028	1365	152257	8.81	Histone-ly	NSD2	NSD2	Histone-ly	15	220.7918	48.12195	1.58E-49	15	14.79853
Q96L73	2696	296649.3	8.08	Histone-ly	NSD1	NSD1	Histone-ly	3	84.66206	46.56034	2.96E-44	3	1.29822
O95478	260	30065.4	10.98	Ribosome	NSA2	NSA2	Ribosome	8	1819.579	49.32592	8.41E-54	8	30
Q16656	503	53540.7	4.69	Nuclear re	NRF1	NRF1	Nuclear re	1	72.61915	34.84051	1.08E-15	1	2.385686
Q9H7Z3	1164	132671.7	7.74	Nuclear ex	NRDE2	NRDE2	Nuclear ex	3	106.5454	46.15022	6.35E-43	3	2.835052
O43847	1151	131699.6	4.62	Nardilysin	NRDC	NRDC	Nardilysin	6	280.1426	47.32575	8.47E-47	6	5.38662
P01111	189	21229	4.78	GTPase NF	NRAS	NRAS	GTPase NF	2	281.9076	48.83354	4.95E-52	1	11.11111
P10589	423	46155.2	8.3	COUP tran	NR2F1	NR2F1	COUP tran	1	66.33249	33.44052	1.34E-13	1	2.364066
Q86WQ0	139	15875.8	6.1	Nuclear re	NR2C2AP	NR2C2AP	Nuclear re	1	42.21364	42.82398	5.22E-33	1	8.633094
P49116	596	65413.8	6.2	Nuclear re	NR2C2	NR2C2	Nuclear re	1	67.21432	45.70273	1.68E-41	1	2.013423
P15559	274	30867.4	9.34	NAD(P)H c	NQO1	NQO1	NAD(P)H c	2	290.3988	53.06382	4.72E-68	2	8.394161
Q9Y639	398	44386.8	8.11	Neuroplas	NPTN	NPTN	Neuroplas	3	137.6148	43.09668	9.22E-34	3	10.05025
Q6UXI9	565	61906.4	8.38	Nephronec	NPNT	NPNT	Nephronec	2	63.99844	41.04927	2.3E-28	2	3.716814
O75607	178	19343.5	4.27	Nucleoplas	NPM3	NPM3	Nucleoplas	3	688.927	47.54639	1.51E-47	3	26.40449
Q86SE8	214	24152.1	4.66	Nucleoplas	NPM2	NPM2	Nucleoplas	2	46.72967	31.7002	2.17E-11	2	12.14953
P06748	294	32574.8	4.39	Nucleopho	NPM1	NPM1	Nucleopho	10	5136.492	50.40194	8.98E-58	10	34.69388
Q8TAT6	608	68119.7	6.34	Nuclear pr	NPLOC4	NPLOC4	Nuclear pr	2	119.6586	45.13123	1.01E-39	2	3.947368
P55786	919	103275.4	5.44	Puromycin	NPEPPS	NPEPPS	Puromycin	15	515.3467	56.16051	9.27E-81	9	18.82481
O15118	1278	142165.4	5	NPC intrac	NPC1	NPC1	NPC intrac	2	122.7849	29.48887	3.41E-09	2	1.956182
Q9Y314	301	33171.7	9.08	Nitric oxid	NOSIP	NOSIP	Nitric oxid	2	243.88	47.82626	1.67E-48	2	8.637874
O75052	506	56149.3	6.25	Carboxyl-t	NOS1AP	NOS1AP	Carboxyl-t	8	189.4435	56.38306	1.13E-81	8	19.16996
Q86U38	636	69437	7.29	Nucleolar p	NOP9	NOP9	Nucleolar p	1	583.3539	33.69024	5.93E-14	1	2.201258
Q9Y2X3	529	59577.6	9.4	Nucleolar p	NOP58	NOP58	Nucleolar p	19	2458.199	53.88423	2.49E-71	19	42.91115
O00567	594	66049.3	9.77	Nucleolar p	NOP56	NOP56	Nucleolar p	19	933.576	51.37275	1.86E-61	19	35.85859
Q9NZM5	478	54388.7	11.04	Ribosome	NOP53	NOP53	Ribosome	5	365.3248	49.45784	2.78E-54	5	11.08787
P46087	812	89301.1	9.83	28S rRNA (	NOP2	NOP2	28S rRNA (	18	851.3549	51.61626	2.14E-62	18	25.98522
Q9Y3C1	178	21188.2	10.52	Nucleolar p	NOP16	NOP16	Nucleolar p	7	1984.354	52.57482	4.02E-66	7	39.32584
P78316	857	97667.5	7.63	Nucleolar p	NOP14	NOP14	Nucleolar p	8	175.0782	48.88871	3.14E-52	8	12.01867
Q9NPE3	64	7705.9	10.52	H/ACA ribo	NOP10	NOP10	H/ACA ribo	4	240.4119	50.01792	2.41E-56	4	71.875
Q15233	471	54231.3	9.49	Non-POU	NONO	NONO	Non-POU	20	8381.584	52.89298	2.27E-67	20	53.07856
Q15155	1222	134323	5.58	BOS comp	NOMO1	NOMO1	BOS comp	12	373.0959	51.90507	1.67E-63	1	13.82979
Q5C9Z4	860	96256.4	8.26	Nucleolar l	NOM1	NOM1	Nucleolar l	19	1490.935	52.37901	2.35E-65	19	26.16279
Q14978	699	73602.5	10.23	Nucleolar a	NOLC1	NOLC1	Nucleolar a	21	9100.282	51.57443	3.11E-62	21	29.61373
Q5SY16	702	79321.9	9.39	Polynuclec	NOL9	NOL9	Polynuclec	11	987.3864	49.38674	5.05E-54	11	20.08547
Q9UMY1	257	29426.2	10.42	U3 small n	NOL7	NOL7	U3 small n	3	129.3215	44.24992	4.57E-37	3	7.782101
Q9H6R4	1146	127591.9	7.67	Nucleolar p	NOL6	NOL6	Nucleolar p	6	107.623	56.77576	3.06E-83	6	6.719023
Q9UGY1	213	24662.7	10.94	Nucleolar p	NOL12	NOL12	Nucleolar p	3	335.4183	46.67579	1.24E-44	3	22.53521
Q9H8H0	719	81122.9	5.98	Nucleolar p	NOL11	NOL11	Nucleolar p	5	95.8534	49.22276	1.98E-53	5	7.927677
Q9BSC4	688	80300.9	8.68	Nucleolar p	NOL10	NOL10	Nucleolar p	17	1039.498	50.71677	5.85E-59	17	24.27326
Q9BVI4	516	58467.1	7.53	Nucleolar c	NOC4L	NOC4L	Nucleolar c	3	83.91664	46.83927	3.59E-45	3	9.108527
Q8WTT2	800	92546.9	9.77	Nucleolar c	NOC3L	NOC3L	Nucleolar c	22	1868.547	52.11668	2.51E-64	22	24.5
Q9Y3T9	749	84918.6	5.31	Nucleolar c	NOC2L	NOC2L	Nucleolar c	10	316.8506	51.58715	2.77E-62	10	17.89052
Q9ULX3	412	46674.6	7.2	RNA-bind	NOB1	NOB1	RNA-bind	6	1732.709	51.20356	8.23E-61	6	20.87379
Q8NC60	698	78457.1	8.86	Nitric oxid	NOA1	NOA1	Nitric oxid	15	1069.974	50.34418	1.47E-57	15	28.79656
P30419	496	56805.9	7.91	Glycylpept	NMT1	NMT1	Glycylpept	1	159.5594	45.35421	2.08E-40	1	2.217742
Q9HAN9	279	31932.2	9.24	Nicotinam	NMNAT1	NMNAT1	Nicotinam	1	108.688	48.95494	1.82E-52	1	3.942652
Q9Y5B8	376	42491.4	6.44	Nucleoside	NME7	NME7	Nucleoside	4	79.37922	47.57288	1.23E-47	4	12.76596
Q13232	169	19014.8	7.97	Nucleoside	NME3	NME3	Nucleoside	2	144.6461	48.03528	3.17E-49	2	17.15976
P22392	152	17297.9	8.69	Nucleoside	NME2	NME2	Nucleoside	4	258.1116	52.0253	5.7E-64		31.57895
P15531	152	17148.6	6.11	Nucleoside	NME1	NME1	Nucleoside	7	460.0714	50.21598	4.44E-57	3	53.28947
Q96D46	503	57602.7	7.15	60S riboso	NMD3	NMD3	60S riboso	12	394.0515	53.38986	2.38E-69	12	26.24254
Q86UT6	975	107614.8	7.38	NLR family	NLRX1	NLRX1	NLR family	1	51.16302	43.08113	1.02E-33	1	1.025641
Q9NX02	1062	120513.9	5.96	NACHT, LF	NLRP2	NLRP2	NACHT, LF	4	228.1317	51.52193	4.93E-62	3	3.672316
Q9BYT8	704	80651.1	6.62	Neurolysin	NLN	NLN	Neurolysin	1	217.5268	49.75321	2.29E-55	1	1.420455
Q9UBE8	527	58282.4	8.19	Serine/thre	NLK	NLK	Serine/thre	3	94.55795	49.21826	2.06E-53	3	5.6926
Q9NVX2	485	53319.8	7.37	Notchless	NLE1	NLE1	Notchless	10	382.5508	51.96895	9.46E-64	10	30.51546
P30414	1462	165675	10.66	NK-tumor	NKTR	NKTR	NK-tumor	8	324.9189	42.26706	1.68E-31	8	7.729138
O15226	690	77672.4	9.09	NF-kappa-	NKRF	NKRF	NF-kappa-	31	5008.008	53.9468	1.4E-71	31	43.47826
Q6ZUT1	292	34109.6	10.43	Uncharacte	NKAPD1	NKAPD1	Uncharacte	3	164.4639	47.33653	7.81E-47	3	11.64384
Q8N5F7	415	47137.8	10.8	NF-kappa-	NKAP	NKAP	NF-kappa-	7	362.6823	52.39561	2.02E-65	4	22.16867
Q86X76	327	35896.1	7.78	Deaminate	NIT1	NIT1	Deaminate	4	209.4187	49.40331	4.39E-54	4	13.76147
Q9Y2I1	1504	166627.1	4.8	Nischarin	NISCH	NISCH	Nischarin	1	172.7963	45.38917	1.62E-40	1	0.731383
O75323	286	33742.4	9.74	Protein Nip	NIPSNAP2	NIPSNAP2	Protein Nip	2	329.8186	46.09264	9.7E-43	2	5.944056
Q9BPW8	284	33309.7	9.67	Protein Nip	NIPSNAP1	NIPSNAP1	Protein Nip	7	292.6862	46.56506	2.86E-44	6	28.16901
Q6KC79	2804	316048.4	8.01	Nipped-B-	NIPBL	NIPBL	Nipped-B-	31	1175.494	50.6779	8.2E-59	31	13.44508
Q9Y221	180	20462.5	8.73	60S riboso	NIP7	NIP7	60S riboso	2	211.7317	31.53146	3.37E-11	2	11.11111
Q9Y2I6	1382	156342.3	4.72	Ninein-like	NINL	NINL	Ninein-like	2	50.52544	25.22491	9.21E-07	2	1.447178
Q8IY84	436	49605.7	8.53	Serine/thre	NIM1K	NIM1K	Serine/thre	1	151.1417	34.27545	8.17E-15	1	3.211009
Q9BYG3	293	34222	10.55	MKI67 FHA	NIFK	NIFK	MKI67 FHA	11	702.7321	49.22253	1.99E-53	11	33.4471
Q96TA1	746	84137.2	6.11	Protein Nil	NIBAN2	NIBAN2	Protein Nil	3	127.2272	48.39908	1.71E-50	3	5.630027

Q9BZQ8	928	103133.9	4.44	Protein Nil	NIBAN1	NIBAN1	Protein Nil	5	197.9467	41.72245	4.52E-30	5	6.357759
Q9P206	1035	107093.8	10.41	NHS-like p	NHSL3	NHSL3	NHS-like p	2	104.8755	30.79329	2.09E-10	2	1.256039
Q9NX24	153	17200.9	8.35	H/ACA ribo	NHP2	NHP2	H/ACA ribo	2	1205.48	44.18207	7.24E-37	2	17.64706
Q8NBF2	726	79442.9	5.23	NHL repea	NHLRC2	NHLRC2	NHL repea	6	135.0671	45.03375	2.01E-39	6	9.504132
Q15599	337	37413.5	8.06	Na(+)/H(+	NHERF2	NHERF2	Na(+)/H(+	3	80.7186	41.4084	2.88E-29	3	10.68249
O14745	358	38868.1	5.52	Na(+)/H(+	NHERF1	NHERF1	Na(+)/H(+	5	177.1403	52.75668	7.88E-67	5	15.92179
P08138	427	45183	4.32	Tumor nec	NGFR	NGFR	Tumor nec	1	45.48815	47.25714	1.44E-46	1	2.810304
Q8NEJ9	315	35893.8	10.22	Neuroguic	NGDN	NGDN	Neuroguic	7	619.6797	53.42971	1.65E-69	7	27.93651
Q6ZNB6	911	101338.7	8.45	NF-X1-typ	NFXL1	NFXL1	NF-X1-typ	14	372.6915	50.8507	1.82E-59	14	17.12404
Q12986	1120	124393.6	8.27	Transcripti	NFX1	NFX1	Transcripti	16	749.7311	48.73116	1.15E-51	16	16.60714
Q9Y697	457	50195.2	8.45	Cysteine d	NFS1	NFS1	Cysteine d	4	93.82456	51.68874	1.13E-62	4	10.7221
Q6P4R8	1299	139000	9.85	Nuclear fac	NFRKB	NFRKB	Nuclear fac	6	101.7756	42.58819	2.3E-32	6	6.004619
Q9UBC1	381	43256.7	7.61	NF-kappa-	NFKBIL1	NFKBIL1	NF-kappa-	3	86.57572	46.07535	1.1E-42	3	10.76115
Q00653	900	96748.4	6.2	Nuclear fac	NFKB2	NFKB2	Nuclear fac	13	253.8017	51.80019	4.25E-63	13	18.33333
Q14938	502	55097.7	8.88	Nuclear fac	NFIX	NFIX	Nuclear fac	6	438.1334	50.47216	4.91E-58	4	12.749
P08651	508	55674.4	8.54	Nuclear fac	NFIC	NFIC	Nuclear fac	6	316.9975	50.86329	1.63E-59	6	11.61417
Q12857	509	55943.7	8.62	Nuclear fac	NFIA	NFIA	Nuclear fac	1	110.8935	35.91387	1.74E-17	1	2.357564
Q14494	772	84702.8	4.25	Endoplasm	NFE2L1	NFE2L1	Endoplasm	1	73.55125	33.16382	3.2E-13	1	1.554404
Q13469	925	100145	7.3	Nuclear fac	NFATC2	NFATC2	Nuclear fac	7	424.8036	54.5908	3.26E-74	7	9.945946
O95644	943	101241.7	6.99	Nuclear fac	NFATC1	NFATC1	Nuclear fac	4	138.822	52.15406	1.79E-64	4	5.408271
Q96JN8	1562	166905.6	5.67	Neuralizec	NEURL4	NEURL4	Neuralizec	1	65.57589	46.07774	1.08E-42	1	0.640205
Q99519	415	45467	5.68	Sialidase-1	NEU1	NEU1	Sialidase-1	4	193.8535	50.48515	4.39E-58	4	9.39759
Q6NW34	567	64551.2	10.44	Nucleolus	NEPRO	NEPRO	Nucleolus	4	134.9883	46.04828	1.35E-42	4	7.583774
O60524	1076	122953.5	6.28	Ribosome	NEMF	NEMF	Ribosome	4	244.3089	45.01992	2.22E-39	4	3.717472
P18615	380	43239.3	9.92	Negative e	NELFE	NELFE	Negative e	6	243.6472	51.13903	1.45E-60	6	17.10526
Q8WX92	580	65696.7	6.02	Negative e	NELFB	NELFB	Negative e	3	139.1655	51.09996	2.04E-60	3	6.551724
Q9H3P2	528	57276.2	9.55	Negative e	NELFA	NELFA	Negative e	3	62.41735	32.91389	6.91E-13	3	6.628788
Q8TD19	979	107167.7	5.5	Serine/thre	NEK9	NEK9	Serine/thre	6	181.8968	48.31615	3.34E-50	6	8.375894
Q8TDX7	302	34550.7	8.37	Serine/thre	NEK7	NEK7;NEK6	Serine/thre	1	27.04706	41.94747	1.17E-30	1	5.298013
Q969S2	332	36826.3	6.78	Endonucle	NEIL2	NEIL2	Endonucle	3	177.0845	48.57925	3.98E-51	3	14.15663
Q96FI4	390	43683.6	10.54	Endonucle	NEIL1	NEIL1	Endonucle	8	222.9994	49.89414	6.97E-56	8	26.92308
Q15843	81	9071.5	9	NEDD8	NEDD8	NEDD8	NEDD8	2	357.9032	48.99521	1.3E-52	2	17.28395
Q96PU5	975	111930.9	5.6	E3 ubiquiti	NEDD4L	NEDD4L	E3 ubiquiti	5	311.5345	50.55034	2.5E-58	5	5.74359
P46934	1319	149113.2	6.56	E3 ubiquiti	NEDD4	NEDD4	E3 ubiquiti	5	283.8486	54.90117	1.63E-75	4	4.700531
Q92692	538	57741.5	4.46	Nectin-2	NECTIN2	NECTIN2	Nectin-2	1	66.30275	44.86536	6.58E-39	1	2.973978
Q9NVZ3	263	28338.7	8.67	Adaptin ea	NECAP2	NECAP2	Adaptin ea	3	206.4125	48.01053	3.87E-49	3	16.34981
P19404	249	27391.4	8.21	NADH deh	NDUFV2	NDUFV2	NADH deh	2	195.2377	47.79554	2.13E-48	2	9.638554
P49821	464	50816.7	8.29	NADH deh	NDUFV1	NDUFV1	NADH deh	11	479.9627	50.48844	4.28E-58	11	31.46552
O00217	210	23704.8	6.27	NADH deh	NDUFS8	NDUFS8	NADH deh	4	294.1574	54.27068	6.78E-73	4	23.33333
O75251	213	23563.3	10.36	NADH deh	NDUFS7	NDUFS7	NADH deh	2	281.5875	44.87795	6.02E-39	2	10.79812
O43920	106	12517.4	9.54	NADH deh	NDUFS5	NDUFS5	NADH deh	1	151.6098	35.76013	3.22E-17	1	11.32075
O75489	264	30241.2	7.62	NADH deh	NDUFS3	NDUFS3	NADH deh	6	636.5847	52.40451	1.87E-65	6	30.30303
O75306	463	52545.3	7.61	NADH deh	NDUFS2	NDUFS2	NADH deh	6	560.9388	47.01085	9.61E-46	6	14.03888
P28331	727	79466.8	6.11	NADH-ubi	NDUFS1	NDUFS1	NADH-ubi	15	542.193	51.77493	5.26E-63	15	27.64787
Q9Y6M9	179	21830.8	8.56	NADH deh	NDUFB9	NDUFB9	NADH deh	2	207.347	49.90207	6.51E-56	2	12.84916
O95169	186	21765.7	6.79	NADH deh	NDUFB8	NDUFB8	NADH deh	1	215.215	48.40263	1.66E-50	1	5.913978
P17568	137	16401.8	9.21	NADH deh	NDUFB7	NDUFB7	NADH deh	1	220.4296	36.96691	2.1E-19	1	7.29927
O43674	189	21750.1	10.02	NADH deh	NDUFB5	NDUFB5	NADH deh	1	85.99214	28.58999	1.71E-08	1	5.820106
O95168	129	15208.4	10.24	NADH deh	NDUFB4	NDUFB4	NADH deh	2	442.3737	46.50076	4.63E-44	2	17.82946
O43676	98	11401.9	9.73	NADH deh	NDUFB3	NDUFB3	NADH deh	3	177.307	49.37926	5.37E-54	3	28.57143
O96000	172	20776.5	8.65	NADH deh	NDUFB10	NDUFB10	NADH deh	3	203.587	56.27915	3E-81	3	18.60465
O75438	58	6961.2	9.34	NADH deh	NDUFB1	NDUFB1	NADH deh	1	22.55544	36.43164	2.07E-18	1	13.7931
Q9P032	175	20266.3	9.35	NADH deh	NDUFAF4	NDUFAF4	NADH deh	1	185.7252	32.16036	6.22E-12	1	5.714286
Q8N183	169	19856.2	9.47	NADH deh	NDUFAF2	NDUFAF2	NADH deh	1	64.25259	44.18853	6.93E-37	1	9.467456
Q9Y375	327	37763.4	7.8	Complex I	NDUFAF1	NDUFAF1	Complex I	1	35.63156	45.07373	1.52E-39	1	5.810398
O14561	156	17417.1	4.53	Acyl carrie	NDUFAB1	NDUFAB1	Acyl carrie	2	224.1722	37.99678	1.94E-21	2	15.38462
Q16795	377	42509.2	10.37	NADH deh	NDUFA9	NDUFA9	NADH deh	10	557.8296	51.73506	7.51E-63	10	28.64721
P51970	172	20104.9	7.74	NADH deh	NDUFA8	NDUFA8	NADH deh	2	79.57416	39.28826	3.33E-24	2	12.2093
P56556	128	15136.5	10.64	NADH deh	NDUFA6	NDUFA6	NADH deh	4	181.287	46.02704	1.57E-42	4	41.40625
Q16718	116	13458.6	5.81	NADH deh	NDUFA5	NDUFA5	NADH deh	2	328.2339	44.56088	5.46E-38	2	16.37931
O00483	81	9369.8	9.84	Cytochrom	NDUFA4	NDUFA4	Cytochrom	2	1225.128	50.9626	6.82E-60	2	27.16049
O95167	84	9278.8	8.92	NADH deh	NDUFA3	NDUFA3	NADH deh	1	95.92738	38.74178	5.28E-23	1	13.09524
O43678	99	10921.4	10.11	NADH deh	NDUFA2	NDUFA2	NADH deh	1	110.0613	35.85734	2.19E-17	1	10.10101
Q9P0J0	144	16698.2	8.84	NADH deh	NDUFA13	NDUFA13	NADH deh	3	403.4561	45.457	9.95E-41	3	22.91667
Q9UI09	145	17114.4	10.1	NADH deh	NDUFA12	NDUFA12	NADH deh	2	160.7411	46.39644	1.01E-43	2	20.68966
Q86Y39	141	14852	8.89	NADH deh	NDUFA11	NDUFA11	NADH deh	1	213.1928	47.44857	3.25E-47	1	10.6383
O95299	355	40750.3	8.68	NADH deh	NDUFA10	NDUFA10	NADH deh	10	451.2289	52.76126	7.56E-67	10	29.57746
Q9UGV2	375	41408.4	4.98	Protein ND	NDRG3	NDRG3	Protein ND	1	337.36	46.1446	6.62E-43	1	2.666667
Q92597	394	42835.2	5.67	Protein ND	NDRG1	NDRG1	Protein ND	6	297.2404	51.32121	2.92E-61	6	26.64975
Q9NXR1	335	37720.2	4.79	Nuclear dis	NDE1	NDE1	Nuclear dis	2	560.2917	51.69919	1.03E-62	1	9.552239
Q9HCD5	579	65535.8	9.93	Nuclear re	NCOA5	NCOA5	Nuclear re	14	1630.694	49.36212	6.21E-54	14	22.79793
Q969V3	563	62973.8	6.88	BOS comp	NCLN	NCLN	BOS comp	9	436.7744	52.68895	1.45E-66	9	18.82771
P19338	710	76613.9	4.32	Nucleolin	NCL	NCL	Nucleolin	42	137850.5	51.26098	4.96E-61	42	43.94366
Q9NZQ3	722	78959.3	6.36	NCK-intera	NCKIPSD	NCKIPSD	NCK-intera	1	235.5109	29.11704	6.85E-09	1	1.66205
Q9Y2A7	1128	128788.7	6.61	Nck-assoc	NCKAP1	NCKAP1	Nck-assoc	4	120.96	48.9341	2.16E-52	4	3.900709
O43639	380	42914.8	6.95	Cytoplasm	NCK2	NCK2	Cytoplasm	2	38.88081	42.34848	1.02E-31	1	5.789474
Q6PIU2	408	45807.4	7.26	Neutral ch	NCEH1	NCEH1	Neutral ch	6	162.4416	47.54842	1.49E-47	6	16.42157
Q53F19	620	70591.8	5.42	Nuclear ca	NCBP3	NCBP3	Nuclear ca	4	396.6828	44.44306	1.22E-37	4	7.258065
P52298	156	18001	8.41	Nuclear ca	NCBP2	NCBP2	Nuclear ca	2	432.307	48.41042	1.56E-50	2	15.38462
Q09161	790	91838.6	6.4	Nuclear ca	NCBP1	NCBP1	Nuclear ca	17	1608.437	53.37059	2.87E-69	17	26.20253
P42695	1498	168889.1	7.53	Condensin	NCAPD3	NCAPD3	Condensin	3	51.07604	44.45472	1.13E-37	3	2.46996
Q9H0A0	1025	115728.8	8.39	RNA cytidi	NAT10	NAT10	RNA cytidi	18	1314.064	53.32151	4.48E-69	18	20.68293
P18440	290	33898.4	6.51	Arylamine	NAT1	NAT1	Arylamine	2	110.6751	41.17508	1.12E-28	2	6.206897
Q96I59	477	54089.6	7.26	Asparaginy	NARS2	NARS2	Asparaginy	2	306.6833	38.97937	1.61E-23	2	3.354298
O43776	548	62942.4	6.17	Asparagin	NARS1	NARS1	Asparagin	8	305.134	51.42219	1.19E-61	8	16.9708
Q6XQN6	538	57577.6	5.37	Nicotinate	NAPRT	NAPRT	Nicotinate	3	147.7243	41.62882	7.88E-30	3	6.505576
Q99733	375	42823.1	4.32	Nucleosom	NAP1L4	NAP1L4	Nucleosom	6	1126.834	52.89142	2.3E-67	5	14.4
P55209	391	45373.8	4.09	Nucleosom	NAP1L1	NAP1L1	Nucleosom	7	3262.045	49.80751	1.45E-55	7	19.94885
P43490	491	55520.8	7.18	Nicotinam	NAMPT	NAMPT	Nicotinam	12	647.5681	52.07645	3.58E-64	12	29.12424
Q9UJ70	344	37375.3	6.18	N-acetyl-D	NAGK	NAGK	N-acetyl-D	4	88.96299	48.54079	5.44E-51	4	13.0814
Q96HR8	494	53716.1	4.5	H/ACA ribo	NAF1	NAF1	H/ACA ribo	4	888.1	46.0695	1.15E-42	4	9.716599
Q13564	534	60245.8	5.06	NEDD8-ac	NAE1	NAE1	NEDD8-ac	2	221.7243	51.34778	2.31E-61	2	5.805243
Q4G0N4	442	49432.5	8.26	NAD kinas	NADK2	NADK2	NAD kinas	3	119.5232	46.18425	4.93E-43	3	9.049774

Q96RE7	527	57257.8	5.51	Nucleus ac	NACC1	NACC1	Nucleus ac	4	204.021	47.2884	1.13E-46	4	9.487666
E9PAV3	2078	205419	10.32	Nascent po	NACA	NACA	Nascent po	4	155.2839	48.19535	8.82E-50	3	2.646776
Q96AH0	204	22423.3	10.13	SOSS com	NABP1	NABP1	SOSS com	1	96.08588	22.53395	4.29E-06	1	4.411765
Q9GZZ1	169	19398.2	9.08	N-alpha-a	NAA50	NAA50	N-alpha-a	3	238.6418	51.08089	2.4E-60	3	20.11834
Q86UY6	237	27193.7	7.42	N-alpha-a	NAA40	NAA40	N-alpha-a	1	315.3548	48.54939	5.07E-51	1	5.063291
Q5VZE5	725	83638.4	7.05	N-alpha-a	NAA35	NAA35	N-alpha-a	1	204.1034	47.57114	1.25E-47	1	1.241379
Q147X3	362	39319.1	5.2	N-alpha-a	NAA30	NAA30	N-alpha-a	1	128.9295	36.49434	1.59E-18	1	3.59116
Q14CX7	972	112291.3	6.61	N-alpha-a	NAA25	NAA25	N-alpha-a	2	49.66004	35.77626	3.02E-17	2	2.160494
Q9BXJ9	866	101271.4	7.45	N-alpha-a	NAA15	NAA15	N-alpha-a	5	191.6544	43.24569	3.55E-34	5	5.542725
P41227	235	26458.3	5.39	N-alpha-a	NAA10	NAA10	N-alpha-a	1	27.20028	47.97341	5.2E-49	1	6.382979
Q92802	583	67458.5	6.36	NEDD4-bi	N4BP2L2	N4BP2L2	NEDD4-bi	2	89.05705	50.93434	8.72E-60	2	3.773585
Q9NZM1	2061	234706.8	6.06	Myoferlin	MYOF	MYOF	Myoferlin	58	1145.914	56.46694	5.05E-82	57	32.89665
Q13459	2157	243398.8	9.06	Unconvent	MYO9B	MYO9B	Unconvent	8	123.4225	47.76193	2.78E-48	8	4.636069
Q9UM54	1294	149689.8	8.72	Unconvent	MYO6	MYO6	Unconvent	54	4324.176	53.71438	1.22E-70	54	42.50386
Q9ULV0	1848	213670.7	7.19	Unconvent	MYO5B	MYO5B	Unconvent	10	179.9833	55.19489	9.91E-77	10	6.277056
Q9Y4I1	1855	215403.2	8.68	Unconvent	MYO5A	MYO5A	Unconvent	20	306.7236	54.21415	1.12E-72	15	11.53639
Q12965	1108	127061.1	9.3	Unconvent	MYO1E	MYO1E	Unconvent	28	1048.68	54.72182	9.39E-75	24	29.69314
O94832	1006	116201.3	9.87	Unconvent	MYO1D	MYO1D	Unconvent	47	3631.984	53.23563	9.75E-69	45	49.60239
O00159	1063	121680.6	9.86	Unconvent	MYO1C	MYO1C	Unconvent	34	6298.755	54.62043	2.48E-74	34	37.4412
O43795	1136	131983.9	9.87	Unconvent	MYO1B	MYO1B	Unconvent	41	4892.629	53.58647	3.98E-70	36	39.52465
Q96H55	970	109134	7.73	Unconvent	MYO19	MYO19	Unconvent	2	148.6714	51.39943	1.46E-61	2	2.268041
Q92614	2054	233112.9	6.23	Unconvent	MYO18A	MYO18A	Unconvent	36	1242.066	52.44825	1.26E-65	36	21.90847
Q9HD67	2058	237345	6.13	Unconvent	MYO10	MYO10	Unconvent	1	51.97901	28.78899	1.22E-08	1	0.728863
P24844	172	19827	4.54	Myosin reg	MYL9	MYL9	Myosin reg	2	300.1416	46.65196	1.48E-44	2	12.2093
P14649	208	22763.9	5.44	Myosin ligl	MYL6B	MYL6B	Myosin ligl	6	6504.195	50.33539	1.59E-57	3	31.25
P60660	151	16930	4.29	Myosin ligl	MYL6	MYL6	Myosin ligl	7	7578.235	49.3445	7.19E-54	7	50.33113
O14950	172	19779	4.44	Myosin reg	MYL12B	MYL12B;M	Myosin reg	9	8070.26	48.30871	3.55E-50	4	62.7907
P05976	194	21144.9	4.68	Myosin ligl	MYL1	MYL1;MYL	Myosin ligl	1	154.483	37.24602	6.1E-20	1	6.185567
P35579	1960	226530.5	5.29	Myosin-9	MYH9	MYH9	Myosin-9	112	20199.48	54.07053	4.28E-72	105	51.73469
P11055	1940	223902.9	5.55	Myosin-3	MYH3	MYH3;MYH	Myosin-3;I	4	148.8667	51.81947	3.57E-63	1	2.57732
Q7Z406	1995	227868.6	5.3	Myosin-14	MYH14	MYH14	Myosin-14	71	3896.341	54.92024	1.36E-75	71	39.84962
P35749	1972	227337.5	5.18	Myosin-11	MYH11	MYH11	Myosin-11	1	4461.578	22.16726	5.02E-06	1	0.557809
P35580	1976	228997.2	5.22	Myosin-10	MYH10	MYH10	Myosin-10	82	12107.43	51.87688	2.14E-63	61	42.15587
Q9HB07	376	42508	6.83	MYG1 exor	MYG1	MYG1	MYG1 exor	4	128.9218	50.21566	4.45E-57	4	11.70213
O75592	4678	513631.4	7.02	E3 ubiquiti	MYCBP2	MYCBP2	E3 ubiquiti	8	211.0515	48.09416	1.98E-49	8	2.116289
Q9BQG0	1328	148853.2	9.87	Myb-bindi	MYBBP1A	MYBBP1A	Myb-bindi	22	564.8589	51.87687	2.14E-63	22	19.0512
Q96S97	322	35273.1	8.2	Myeloid-a	MYADM	MYADM	Myeloid-a	3	1558.926	50.02189	2.34E-56	3	15.83851
Q14764	893	99326.2	5.15	Major vaul	MVP	MVP	Major vaul	25	2059.301	53.83069	4.06E-71	25	37.17805
Q03426	396	42450.5	6.43	Mevalonat	MVK	MVK	Mevalonat	1	55.28939	45.86386	5.19E-42	1	3.030303
P53602	400	43404.1	7.25	Diphospho	MVD	MVD	Diphospho	3	221.1712	47.28636	1.15E-46	3	8.75
Q96EY5	273	28782.7	9.22	Multivesic	MVB12A	MVB12A	Multivesic	1	249.6416	30.70654	2.55E-10	1	5.494505
Q969V5	352	39799.7	8.2	Mitochonc	MUL1	MUL1	Mitochonc	1	29.17984	20.75443	8.7E-06	1	3.409091
Q5HYI7	312	35093	7.94	Metaxin-3	MTX3	MTX3	Metaxin-3	4	107.8835	52.27939	5.76E-65	4	11.85897
O75431	263	29763	6.25	Metaxin-2	MTX2	MTX2	Metaxin-2	4	353.9636	46.99629	1.07E-45	4	16.73004
Q13505	466	51462.4	10.23	Metaxin-1	MTX1	MTX1	Metaxin-1	6	155.1581	51.81431	3.73E-63	6	15.02146
P42285	1042	117803.8	6.49	Exosome R	MTREX	MTREX	Exosome R	25	939.7122	53.11453	2.97E-68	25	30.42226
P58546	118	12894.7	5.18	Myotrophi	MTPN	MTPN	Myotrophi	1	179.9475	45.28908	3.31E-40	1	14.40678
Q9NVV4	582	66171	9.38	Poly(A) RN	MTPAP	MTPAP	Poly(A) RN	6	166.781	45.42104	1.29E-40	6	13.05842
P42345	2549	288889	7.17	Serine/thre	MTOR	MTOR	Serine/thre	3	66.49911	44.51407	7.51E-38	3	1.569243
Q9Y2Z2	717	79963.2	8.41	Protein MT	MTO1	MTO1	Protein MT	4	135.3105	44.89335	5.41E-39	4	7.531381
P03905	459	51580.3	9.67	NADH-ubi	MT-ND4	MT-ND4	NADH-ubi	1	146.4701	45.89204	4.23E-42	1	2.178649
P03897	115	13185.9	4.08	NADH-ubi	MT-ND3	MT-ND3	NADH-ubi	1	57.42828	37.03884	1.53E-19	1	13.04348
P03891	347	38960.5	10.3	NADH-ubi	MT-ND2	MT-ND2	NADH-ubi	1	63.4271	32.78324	1.03E-12	1	2.59366
P46199	727	81316.8	7.16	Translatior	MTIF2	MTIF2	Translatior	4	439.5177	51.68323	1.19E-62	4	5.914718
P13995	350	37894.8	9.12	Bifunctiona	MTHFD2	MTHFD2	Bifunctiona	6	876.9861	57.06948	2.27E-84	6	25.14286
Q6UB35	978	105789.1	8.15	Monofunct	MTHFD1L	MTHFD1L	Monofunct	17	602.075	55.1279	1.88E-76	17	21.26789
P11586	935	101530.4	7.32	C-1-tetrah	MTHFD1	MTHFD1	C-1-tetrah	24	1886.908	52.71349	1.15E-66	24	31.97861
Q6P444	385	43383.7	7.02	Mitochonc	MTFR2	MTFR2	Mitochonc	1	104.4775	34.30886	7.27E-15	1	2.077922
Q9UDX5	166	18009.5	9.63	Mitochonc	MTFP1	MTFP1	Mitochonc	1	91.3643	30.6435	2.95E-10	1	6.626506
Q96DP5	389	43831.7	10.19	Methionyl-	MTFMT	MTFMT	Methionyl-	6	200.3032	47.65103	6.66E-48	6	21.33676
Q9Y483	593	67089	8.95	Metal-resp	MTF2	MTF2	Metal-resp	12	547.7029	49.30919	9.68E-54	12	22.93423
Q7Z6M4	381	43957.1	4.43	Transcripti	MTERF4	MTERF4	Transcripti	5	375.2414	44.62431	3.52E-38	5	13.64829
Q86UE4	582	63836.5	10.06	Protein LYI	MTDH	MTDH	Protein LYI	10	726.3153	50.08405	1.38E-56	10	19.93127
P00403	227	25564.7	4.44	Cytochrom	MT-CO2	MT-CO2	Cytochrom	3	3203.167	52.41299	1.73E-65	3	17.62115
P00395	513	57040.9	6.7	Cytochrom	MT-CO1	MT-CO1	Cytochrom	1	315.2608	48.88274	3.3E-52	1	1.559454
O94964	1661	183856	6.48	Microtubu	MTCL2	MTCL2	Microtubu	5	106.8899	49.54127	1.37E-54	5	3.010235
Q9Y4B5	1905	209524.1	6.39	Microtubu	MTCL1	MTCL1	Microtubu	5	228.5338	46.58808	2.4E-44	5	3.569554
Q9Y6C9	303	33330.6	8.04	Mitochonc	MTCH2	MTCH2	Mitochonc	10	376.3908	49.7869	1.73E-55	10	47.52475
Q9NZJ7	389	41543.9	9.7	Mitochonc	MTCH1	MTCH1	Mitochonc	2	156.0169	51.91545	1.52E-63	2	5.912596
P03928	68	7991.6	10.56	ATP syntha	MT-ATP8	MT-ATP8	ATP syntha	1	52.79444	35.37211	1.47E-16	1	13.23529
P00846	226	24816.9	10.68	ATP syntha	MT-ATP6	MT-ATP6	ATP syntha	1	701.2185	45.88794	4.36E-42	1	4.424779
Q969Z3	335	38023	9.5	Mitochonc	MTARC2	MTARC2	Mitochonc	1	78.8897	33.04325	4.66E-13	1	2.089552
Q13126	283	31235.8	7.21	S-methyl-	MTAP	MTAP	S-methyl-	2	166.583	41.37143	3.58E-29	2	7.773852
Q9BTC8	594	67503	8.72	Metastasis	MTA3	MTA3	Metastasis	4	241.0568	51.24522	5.7E-61	4	7.744108
O94776	668	75022.4	10.17	Metastasis	MTA2	MTA2	Metastasis	18	860.1986	57.93139	6.81E-88	18	31.43713
Q13330	715	80785.4	9.65	Metastasis	MTA1	MTA1	Metastasis	19	1795.001	51.86933	2.28E-63	15	30.62937
P02795	61	6042.1	7.87	Metallothic	MT2A	MT2A;MT1	Metallothic	1	94.269	48.41524	1.51E-50	1	19.67213
Q9BUK6	570	61834.8	6.05	Protein mi	MSTO1	MSTO1	Protein mi	1	37.30384	38.37994	3.11E-22	1	2.105263
Q15800	293	35215.4	7.25	Methylster	MSMO1	MSMO1	Methylster	1	48.12645	40.08701	4.9E-26	1	4.778157
Q13421	630	68984.8	6.29	Mesothelir	MSLN	MSLN	Mesothelir	4	470.4353	50.41391	8.1E-58	4	7.142857
P0C860	356	40644	9.56	MSL comp	MSL3B	MSL3B;MS	MSL comp	1	35.8326	28.4282	2.23E-08	1	1.966292
Q9HCI7	577	62539.8	7.83	E3 ubiquiti	MSL2	MSL2	E3 ubiquiti	4	160.3498	51.50159	5.88E-62	4	8.492201
Q96DH6	328	35196.5	8.72	RNA-bind	MSI2	MSI2	RNA-bind	4	195.9305	53.50135	8.55E-70	3	16.46341
P52701	1360	152784.1	6.89	DNA misn	MSH6	MSH6	DNA misn	34	2646.507	52.81351	4.72E-67	34	29.41176
P43246	934	104742.3	5.49	DNA misn	MSH2	MSH2	DNA misn	14	428.4511	53.565	4.81E-70	14	18.52248
A0A1W2P(	361	39760	8.48	Myb/SANT	MSANTD7	MSANTD7	Myb/SANT	1	56.25983	43.46183	8.72E-35	1	4.709141
Q96H12	275	32362.9	8.56	Myb/SANT	MSANTD3	MSANTD3	Myb/SANT	1	88.32498	25.51556	7.3E-07	1	3.272727
Q9UKD2	239	27560.3	8.6	mRNA turr	MRTO4	MRTO4	mRNA turr	12	1494.636	51.09419	2.14E-60	12	38.49372
P82933	396	45834.5	10.03	Small ribos	MRPS9	MRPS9	Small ribos	14	2753.519	50.47763	4.68E-58	14	33.33333
Q9Y2R9	242	28133.8	10.62	Small ribos	MRPS7	MRPS7	Small ribos	16	6436.257	50.07893	1.44E-56	16	55.78512
P82932	125	14226.4	9.71	Small ribos	MRPS6	MRPS6	Small ribos	4	1657.193	49.10927	5.07E-53	4	32.8
P82675	430	48006.1	10.58	Small ribos	MRPS5	MRPS5	Small ribos	14	4827.06	50.58705	1.82E-58	14	31.16279
P82673	323	36844	8.42	Small ribos	MRPS35	MRPS35	Small ribos	12	1704.314	52.46623	1.06E-65	12	41.79567

P82930	218	25650.2	10.51	Small ribos	MRPS34	MRPS34	Small ribos	7	1551.109	54.26542	7.02E-73	7	32.11009
Q9Y291	106	12628.6	10.77	Small ribos	MRPS33	MRPS33	Small ribos	4	4489.803	50.06192	1.66E-56	4	47.16981
Q92665	395	45318.1	9.92	Small ribos	MRPS31	MRPS31	Small ribos	14	2765.095	52.79321	5.68E-67	14	42.27848
Q9NP92	439	50364.2	8.05	Large ribos	MRPS30	MRPS30	Large ribos	9	348.6379	51.18448	9.73E-61	9	23.46241
Q9Y2Q9	187	20842.6	9.61	Small ribos	MRPS28	MRPS28	Small ribos	7	3474.019	47.50814	2.04E-47	7	52.94118
Q92552	414	47610.9	6.08	Small ribos	MRPS27	MRPS27	Small ribos	14	3875.769	51.97382	9.08E-64	14	33.57488
Q9BYN8	205	24211.5	11.02	Small ribos	MRPS26	MRPS26	Small ribos	10	1238.56	48.64317	2.36E-51	10	38.53659
P82663	173	20116.3	9.14	Small ribos	MRPS25	MRPS25	Small ribos	7	2484.303	51.23866	6.04E-61	7	53.17919
Q96EL2	167	19015	9.72	Small ribos	MRPS24	MRPS24	Small ribos	5	1516.93	51.19307	9.02E-61	5	29.34132
Q9Y3D9	190	21770.5	9.33	Small ribos	MRPS23	MRPS23	Small ribos	11	9012.807	52.3066	4.49E-65	11	59.47368
P82650	360	41280	8.07	Small ribos	MRPS22	MRPS22	Small ribos	18	5560.763	51.03517	3.6E-60	18	37.77778
P82921	87	10688.4	10.38	Small ribos	MRPS21	MRPS21	Small ribos	4	439.2527	47.67342	5.58E-48	4	47.12644
Q9Y399	296	33248.8	9.59	Small ribos	MRPS2	MRPS2	Small ribos	10	1654.321	51.96188	1.01E-63	10	30.06757
Q9Y3D5	142	15849.6	10.06	Small ribos	MRPS18C	MRPS18C	Small ribos	1	135.2482	34.14689	1.28E-14	1	12.67606
Q9Y676	258	29395.4	9.74	Small ribos	MRPS18B	MRPS18B	Small ribos	6	5870.565	53.17068	1.77E-68	6	24.03101
Q9NVS2	196	22183.8	10.95	Large ribos	MRPS18A	MRPS18A	Large ribos	4	543.2772	48.23198	6.59E-50	4	22.44898
Q9Y2R5	130	14502	10.47	Small ribos	MRPS17	MRPS17	Small ribos	4	2491.926	49.60576	7.98E-55	4	37.69231
Q9Y3D3	137	15344.7	9.96	Small ribos	MRPS16	MRPS16	Small ribos	4	510.7656	49.65785	5.12E-55	4	37.22628
P82914	257	29841.9	11.16	Small ribos	MRPS15	MRPS15	Small ribos	10	830.8569	50.10282	1.17E-56	10	29.57198
O60783	128	15138.6	11.93	Small ribos	MRPS14	MRPS14	Small ribos	2	238.6251	51.50362	5.79E-62	2	17.1875
O15235	138	15172.8	11	Small ribos	MRPS12	MRPS12	Small ribos	4	1516.911	46.36777	1.25E-43	4	39.85507
P82912	194	20615.6	11.49	Small ribos	MRPS11	MRPS11	Small ribos	5	2434.138	51.55667	3.63E-62	5	22.16495
P82664	201	22999.3	8.22	Small ribos	MRPS10	MRPS10	Small ribos	7	1711.986	51.33764	2.52E-61	7	46.26866
Q9BYD2	267	30243.1	10.76	Large ribos	MRPL9	MRPL9	Large ribos	5	1459.796	47.63982	7.27E-48	5	22.09738
Q14197	206	23629.9	10.73	Large ribos	MRPL58	MRPL58	Large ribos	5	995.2961	49.05257	8.11E-53	5	29.12621
Q7Z7F7	128	15128.2	11.67	Large ribos	MRPL55	MRPL55	Large ribos	1	36.11548	43.91234	4.45E-36	1	6.25
Q6P161	138	15819	10.15	Large ribos	MRPL54	MRPL54	Large ribos	1	294.1531	42.69304	1.19E-32	1	7.246377
Q96EL3	112	12106.7	8.97	Large ribos	MRPL53	MRPL53	Large ribos	4	181.1237	49.11132	4.99E-53	4	42.85714
Q8N5N7	158	18324.8	8.06	Large ribos	MRPL50	MRPL50	Large ribos	4	643.4365	52.33321	3.54E-65	4	30.37975
Q13405	166	19197.9	9.87	Large ribos	MRPL49	MRPL49	Large ribos	7	677.4419	50.55378	2.42E-58	7	46.38554
Q96GC5	212	23934.6	9.41	Large ribos	MRPL48	MRPL48	Large ribos	2	293.2791	50.86459	1.61E-59	2	11.32075
Q9HD33	250	29450.1	11.08	Large ribos	MRPL47	MRPL47	Large ribos	8	1541.221	51.76035	5.99E-63	8	28.4
Q9H2W6	279	31704.8	7.06	Large ribos	MRPL46	MRPL46	Large ribos	5	320.6135	56.15984	9.27E-81	5	23.65591
Q9BRJ2	306	35350.7	9.42	Large ribos	MRPL45	MRPL45	Large ribos	8	1258.936	47.74236	3.25E-48	8	26.47059
Q9H9J2	332	37535	8.57	Large ribos	MRPL44	MRPL44	Large ribos	11	682.0891	53.41753	1.84E-69	11	29.81928
Q8N983	215	23430.5	8.77	Large ribos	MRPL43	MRPL43	Large ribos	4	651.2927	49.77443	1.92E-55	4	20.93023
Q8IXM3	137	15382.6	10.13	Large ribos	MRPL41	MRPL41	Large ribos	5	1159.01	48.28973	4.14E-50	5	38.68613
Q9NQ50	206	24490.1	10.26	Large ribos	MRPL40	MRPL40	Large ribos	1	85.61119	38.53203	1.48E-22	1	3.883495
Q9BYD3	311	34919	10.18	Large ribos	MRPL4	MRPL4	Large ribos	8	424.6665	47.79419	2.15E-48	8	38.58521
Q9NYK5	338	38711.4	7.72	Large ribos	MRPL39	MRPL39	Large ribos	11	832.1434	53.53176	6.49E-70	11	33.43195
Q96DV4	380	44596.1	7.59	Large ribos	MRPL38	MRPL38	Large ribos	12	7665.361	53.06466	4.71E-68	12	35
Q9BZE1	423	48117.1	8.73	Large ribos	MRPL37	MRPL37	Large ribos	15	1163.822	54.95991	9.27E-76	15	36.87943
Q9P0J6	103	11784.1	11.83	Large ribos	MRPL36	MRPL36	Large ribos	1	202.8851	52.32427	3.85E-65	1	7.76699
Q9NZE8	188	21514.2	11.86	Large ribos	MRPL35	MRPL35	Large ribos	3	415.9355	48.69634	1.53E-51	3	10.10638
Q9BQ48	92	10164.8	12.76	Large ribos	MRPL34	MRPL34	Large ribos	2	1071.036	47.95403	6.08E-49	2	23.91304
O75394	65	7619.1	11.5	Large ribos	MRPL33	MRPL33	Large ribos	3	262.2943	41.8372	2.27E-30	3	41.53846
Q9BYC8	188	21404.6	10.32	Large ribos	MRPL32	MRPL32	Large ribos	2	41.73582	42.01468	7.81E-31	2	12.23404
Q8TCC3	161	18545.7	10.73	Large ribos	MRPL30	MRPL30	Large ribos	2	107.3059	54.09309	3.45E-72	2	13.04348
P09001	348	38632.5	10.03	Large ribos	MRPL3	MRPL3	Large ribos	9	4401.478	48.45172	1.12E-50	9	32.18391
Q13084	256	30156.6	8.58	Large ribos	MRPL28	MRPL28	Large ribos	9	588.7987	51.89961	1.76E-63	9	37.89063
Q9P0M9	148	16072.7	11.04	Large ribos	MRPL27	MRPL27	Large ribos	4	1499.413	47.80541	1.97E-48	4	31.75676
Q96A35	216	24914.7	9.72	Large ribos	MRPL24	MRPL24	Large ribos	8	945.7859	51.01219	4.41E-60	8	42.59259
Q16540	153	17781.1	10.04	Large ribos	MRPL23	MRPL23	Large ribos	4	1131.501	47.63258	7.7E-48	4	29.41176
Q9NWU5	206	23640.5	10.48	Large ribos	MRPL22	MRPL22	Large ribos	6	951.9319	46.17497	5.28E-43	6	31.06796
Q7Z2W9	205	22814.4	10.55	Large ribos	MRPL21	MRPL21	Large ribos	5	1108.519	47.92895	7.42E-49	5	32.19512
Q9BYC9	149	17442.4	11.46	Large ribos	MRPL20	MRPL20	Large ribos	4	687.1398	48.86599	3.79E-52	4	34.89933
Q5T653	305	33300.6	11.84	Large ribos	MRPL2	MRPL2	Large ribos	6	673.2265	48.35196	2.5E-50	6	25.90164
P49406	292	33534.7	10.06	Large ribos	MRPL19	MRPL19	Large ribos	6	1049.176	53.4409	1.48E-69	6	20.54795
Q9H0U6	180	20576.4	9.99	Large ribos	MRPL18	MRPL18	Large ribos	3	477.3279	49.10381	5.31E-53	3	15
Q9NRX2	175	20050	10.64	Large ribos	MRPL17	MRPL17	Large ribos	5	468.0805	46.783	5.49E-45	5	30.28571
Q9NX20	251	28449	10.79	Large ribos	MRPL16	MRPL16	Large ribos	7	451.6589	49.76181	2.13E-55	7	33.86454
Q9P015	296	33419.5	10.56	Large ribos	MRPL15	MRPL15	Large ribos	16	1330.176	50.50934	3.57E-58	16	62.5
Q6P1L8	145	15947.6	10.91	Large ribos	MRPL14	MRPL14	Large ribos	2	229.838	47.77101	2.59E-48	2	13.7931
Q9BYD1	178	20691.8	9.52	Large ribos	MRPL13	MRPL13	Large ribos	9	801.0919	49.63825	6.05E-55	9	44.38202
P52815	198	21347.9	9.27	Large ribos	MRPL12	MRPL12	Large ribos	4	222.8784	46.58249	2.5E-44	4	22.22222
Q9Y3B7	192	20683	10.63	Large ribos	MRPL11	MRPL11	Large ribos	4	375.245	47.09713	4.95E-46	4	23.95833
Q7Z7H8	261	29282.3	10.13	Large ribos	MRPL10	MRPL10	Large ribos	3	329.8013	49.46864	2.54E-54	3	15.32567
Q8NDA8	1641	181247.3	6.88	Maestro he	MROH1	MROH1	Maestro he	2	63.61667	31.41355	4.57E-11	2	1.340646
Q9HC36	420	47019.6	9.05	rRNA meth	MRM3	MRM3	rRNA meth	5	890.9548	50.70504	6.49E-59	5	14.04762
Q6IN84	353	38638	8.02	rRNA meth	MRM1	MRM1	rRNA meth	2	128.6427	38.26633	5.38E-22	2	6.515581
Q9BV20	369	39149.4	6.25	Methylthic	MRI1	MRI1	Methylthic	2	68.40472	39.69677	3.95E-25	2	7.04607
Q9NV56	204	22417	5.64	MRG/MOF	MRGBP	MRGBP	MRG/MOF	1	30.78464	48.55906	4.69E-51	1	7.352941
O95297	269	29082	9.01	Myelin pro	MPZL1	MPZL1	Myelin pro	1	167.4563	45.27186	3.74E-40	1	3.717472
Q6WCQ1	1025	116532.1	6.11	Myosin ph	MPRIP	MPRIP	Myosin ph	22	358.2299	51.57112	3.2E-62	21	24.68293
Q5T2T1	576	65523.2	7.15	MAGUK p5	MPP7	MPP7	MAGUK p5	15	267.3486	53.52682	6.79E-70	15	30.72917
Q99549	860	97181.4	5.86	M-phase p	MPHOSPH	MPHOSPH	M-phase p	11	480.639	48.33434	2.89E-50	11	17.32558
Q99547	160	19023.6	4.86	M-phase p	MPHOSPH	MPHOSPH	M-phase p	2	96.30449	45.87835	4.67E-42	2	6.875
O00566	681	78863.1	4.49	U3 small n	MPHOSPH	MPHOSPH	U3 small n	7	347.9462	51.80986	3.89E-63	7	14.09692
P29372	298	32868.4	10	DNA-3-me	MPG	MPG	DNA-3-me	12	2986.728	52.24945	7.56E-65	12	54.69799
Q2M385	716	78586.5	7.7	Macrophag	MPEG1	MPEG1	Macrophag	1	159.757	38.07972	1.31E-21	1	2.653631
Q9HCE1	1003	113670.2	9.07	Helicase M	MOV10	MOV10	Helicase M	35	2623.374	53.70334	1.36E-70	35	39.88036
Q9UJG1	213	24085.9	7.9	Motile spe	MOSPD1	MOSPD1	Motile spe	3	326.4218	51.9962	7.41E-64	3	13.61502
Q15014	288	32307.6	10.3	Mortality f	MORF4L2	MORF4L2	Mortality f	1	56.19891	40.56607	3.53E-27	1	4.166667
Q9UBU8	362	41473.3	9.76	Mortality f	MORF4L1	MORF4L1	Mortality f	4	120.3598	40.80026	9.51E-28	4	14.64088
Q14149	939	107112.1	5.3	MORC fam	MORC3	MORC3	MORC fam	7	251.7681	55.05146	3.99E-76	7	9.584665
Q9Y6X9	1032	117822.1	8.56	ATPase MC	MORC2	MORC2	ATPase MC	1	95.47142	33.73222	5.16E-14	1	0.872093
Q13724	837	91916.9	9.18	Mannosyl-	MOGS	MOGS	Mannosyl-	10	302.7315	51.59969	2.49E-62	10	13.62007
O95396	460	49668.7	6.12	Adenylyltra	MOCS3	MOCS3	Adenylyltra	1	87.06587	41.89	1.66E-30	1	3.26087
Q9Y3A3	225	26032.2	5.59	MOB-like p	MOB4	MOB4	MOB-like p	1	236.0475	42.12007	4.12E-31	1	6.222222
Q7L9L4	216	25090.6	6.72	MOB kinas	MOB1B	MOB1B;MC	MOB kinas	1	77.29413	43.22809	3.97E-34	1	5.092593
P51948	309	35822.7	5.96	CDK-activa	MNAT1	MNAT1	CDK-activa	5	81.34836	48.76734	8.53E-52	5	21.0356
P22033	750	83133.8	6.92	Methylmal	MMUT	MMUT	Methylmal	6	298.7023	50.62796	1.27E-58	6	11.06667
Q9BU76	263	29411.6	10.75	Multiple m	MMTAG2	MMTAG2	Multiple m	9	1807.629	50.79203	3.04E-59	9	37.26236

Q6ZRQ5	1243	142319.2	7.13	Protein MM	MMS22L	MMS22L	Protein MM	1	57.096	43.1764	5.53E-34	1	0.965406
Q96T76	1030	113288.2	6.32	MMS19 nu	MMS19	MMS19	MMS19 nu	12	241.2987	52.4059	1.85E-65	12	15.14563
Q8N4V1	131	14685.6	9.61	ER membr	MMGT1	MMGT1	ER membr	1	63.40651	42.85547	4.28E-33	1	16.03053
Q96EY8	250	27388	8.78	Corrinoid a	MMAB	MMAB	Corrinoid a	1	127.441	44.91701	4.58E-39	1	4
P55198	1093	112046.9	8.74	Protein AF	MLLT6	MLLT6	Protein AF	1	94.20724	40.09746	4.63E-26	1	1.006404
P42568	568	63350.7	9.06	Protein AF	MLLT3	MLLT3	Protein AF	14	536.2182	49.90857	6.16E-56	14	26.40845
Q03111	559	62055.5	8.93	Protein EN	MLLT1	MLLT1	Protein EN	9	1728.094	50.09057	1.3E-56	9	20.75134
Q15773	248	28147.1	6.9	Myeloid le	MLF2	MLF2	Myeloid le	3	94.70806	49.96707	3.75E-56	3	16.53226
P58340	268	30627	10.12	Myeloid le	MLF1	MLF1	Myeloid le	1	93.85851	27.27232	1.17E-07	1	3.358209
Q14165	292	32233.6	5.09	Malectin	MLEC	MLEC	Malectin	4	840.5355	52.34423	3.21E-65	4	16.09589
Q9H000	416	46939.9	7.64	E3 ubiquiti	MKRN2	MKRN2	E3 ubiquiti	6	288.8428	51.16998	1.1E-60	6	15.14423
Q9UHC7	482	53349	4.79	E3 ubiquiti	MKRN1	MKRN1	E3 ubiquiti	2	135.5407	52.55684	4.73E-66	2	5.809129
Q9UL63	735	84767.1	6.29	Muskelin	MKLN1	MKLN1	Muskelin	4	265.7524	51.68823	1.14E-62	4	5.85034
P46013	3256	358691	10.19	Proliferatic	MKI67	MKI67	Proliferatic	67	1395.77	50.14692	7.99E-57	67	30.65111
Q8IVT2	679	75355.9	6.81	Mitotic inte	MISP	MISP	Mitotic inte	19	1451.953	52.60499	3.07E-66	19	32.98969
Q9NXC5	875	98583.6	6.72	GATOR2 c	MIOS	MIOS	GATOR2 c	2	92.36639	33.23694	2.55E-13	2	2.514286
P14174	115	12476.2	8.05	Macrophag	MIF	MIF	Macrophag	1	284.4962	49.04087	8.93E-53	1	9.565217
Q8N108	512	57982.8	4.06	Mesoderm	MIER1	MIER1	Mesoderm	1	43.89082	42.80941	5.73E-33	1	2.34375
O15344	667	75249.9	6.79	E3 ubiquiti	MID1	MID1	E3 ubiquiti	1	153.76	48.19893	8.57E-50	1	1.649175
Q8IYU8	434	49665.8	9.6	Calcium up	MICU2	MICU2	Calcium up	2	93.45166	51.77867	5.09E-63	2	6.221198
Q9BPX6	476	54350.9	8.62	Calcium up	MICU1	MICU1	Calcium up	1	47.34232	21.57378	6.39E-06	1	1.680672
O14880	152	16516.2	9.68	Glutathion	MGST3	MGST3	Glutathion	4	444.0111	49.12216	4.57E-53	4	34.21053
P10620	155	17598.4	9.71	Microsoma	MGST1	MGST1	Microsoma	2	174.1236	46.85507	3.18E-45	2	18.70968
Q9BQP7	344	39420.7	7.79	Mitochonc	MGME1	MGME1	Mitochonc	15	837.9162	50.49052	4.2E-58	15	43.89535
Q8IWI9	3065	336156.7	6.89	MAX gene	MGA	MGA	MAX gene	7	180.8899	43.32383	2.14E-34	7	2.577488
Q14728	455	48338.5	9.91	Major facil	MFSD10	MFSD10	Major facil	2	324.9977	49.18307	2.77E-53	2	4.835165
Q8IWA4	741	84158.8	6.28	Mitofusin-	MFN1	MFN1	Mitofusin-	2	81.26686	36.6137	9.62E-19	2	3.103914
Q08431	387	43104.6	8.21	Lactadheri	MFGE8	MFGE8	Lactadheri	3	182.1105	49.14524	3.78E-53	3	9.302326
Q9GZY8	342	38464.1	9.35	Mitochonc	MFF	MFF	Mitochonc	1	82.30466	41.72541	4.44E-30	1	3.216374
P55081	439	51958.1	4.64	Microfibrill	MFAP1	MFAP1	Microfibrill	9	913.0726	50.33006	1.66E-57	9	21.64009
Q86U44	580	64473.3	6.38	N6-adeno	METTL3	METTL3	N6-adeno	4	150.5197	45.21223	5.71E-40	4	8.448276
Q6P1Q9	378	43425.8	5.67	tRNA N(3)	METTL2B	METTL2B	tRNA N(3)	1	42.83735	38.49703	1.76E-22	1	3.439153
Q9H7H0	456	50733.2	9.78	Ribosome	METTL17	METTL17	Ribosome	8	295.8617	50.66821	8.91E-59	8	18.64035
A6NJ78	407	46120.6	8.85	12S rRNA	METTL15	METTL15	12S rRNA	3	33.72709	38.07643	1.33E-21	3	8.353808
Q8N6R0	699	78767.2	6.71	eEF1A lysir	METTL13	METTL13	eEF1A lysir	4	171.3808	47.20146	2.22E-46	4	6.866953
P50579	478	52891.1	5.57	Methionine	METAP2	METAP2	Methionine	5	136.963	45.71025	1.59E-41	5	13.17992
P53582	386	43214.9	7.18	Methionine	METAP1	METAP1	Methionine	3	53.61873	43.23009	3.92E-34	3	10.10363
Q7L2J0	689	74354.7	10.01	7SK snRNA	MEPCE	MEPCE	7SK snRNA	21	3559.408	49.07382	6.8E-53	21	39.76778
O00255	610	67496.2	6.52	Menin	MEN1	MEN1	Menin	4	126.7436	45.07565	1.5E-39	4	7.04918
Q9Y316	297	33732.9	7.16	Protein ME	MEMO1	MEMO1	Protein ME	5	169.3905	40.58294	3.22E-27	5	24.24242
P08582	738	80213.9	5.8	Melanotrai	MELTF	MELTF	Melanotrai	16	794.6535	53.22407	1.08E-68	16	25.33875
Q9NPJ6	270	29745.2	4.75	Mediator c	MED4	MED4	Mediator c	2	102.9142	44.0351	1.95E-36	2	7.037037
Q9Y3C7	131	15805	8.75	Mediator c	MED31	MED31	Mediator c	1	58.64359	32.67356	1.42E-12	1	10.68702
Q9ULK4	1368	156472.6	7.42	Mediator c	MED23	MED23	Mediator c	6	110.0659	48.09634	1.94E-49	6	4.97076
Q13503	144	15564.1	4.06	Mediator c	MED21	MED21	Mediator c	1	126.3107	40.37123	1.04E-26	1	9.722222
Q9NVC6	651	72889.6	7.48	Mediator c	MED17	MED17	Mediator c	5	125.8397	40.3639	1.08E-26	5	8.448541
O60244	1454	160605	8.92	Mediator c	MED14	MED14	Mediator c	4	161.7932	53.85267	3.34E-71	4	2.751032
Q71F56	2210	242599.4	5.93	Mediator c	MED13L	MED13L	Mediator c	2	87.2038	29.80197	1.83E-09	2	1.266968
Q15648	1581	168476.6	9.1	Mediator c	MED1	MED1	Mediator c	3	122.1572	26.82071	2.03E-07	3	3.478811
P51608	486	52440.3	10.69	Methyl-Cp	MECP2	MECP2	Methyl-Cp	21	1867.551	50.94274	8.1E-60	21	39.09465
Q6P9B6	456	50993.3	6.2	MTOR-ass	MEAK7	MEAK7	MTOR-ass	1	36.63552	38.58679	1.13E-22	1	2.850877
Q9HAF1	191	21634.7	9.91	Chromatin	MEAF6	MEAF6	Chromatin	6	760.6683	56.2815	2.99E-81	6	35.07853
P23368	584	65442.9	7.67	NAD-depe	ME2	ME2	NAD-depe	2	64.02272	35.77579	3.03E-17	2	3.938356
P21741	143	15585.1	10.49	Midkine	MDK	MDK	Midkine	2	89.48079	47.89611	9.62E-49	2	16.78322
P40926	338	35502.9	8.9	Malate del	MDH2	MDH2	Malate del	12	1069.579	51.03519	3.6E-60	12	44.67456
P40925	334	36425.8	7.45	Malate del	MDH1	MDH1	Malate del	5	252.9003	52.03381	5.28E-64	5	18.56287
Q14676	2089	226664.3	5.14	Mediator c	MDC1	MDC1	Mediator c	30	1144.915	52.92234	1.74E-67	30	23.74342
Q8NE86	351	39866.5	8.84	Calcium ur	MCU	MCU	Calcium ur	4	338.0809	46.77474	5.84E-45	4	12.25071
Q9ULC4	181	20555.2	9.16	Malignant	MCTS1	MCTS1	Malignant	1	520.5154	36.16076	6.39E-18	1	4.972376
Q96EZ8	462	51802.8	9.99	Microsphe	MCRS1	MCRS1	Microsphe	4	181.3727	46.98647	1.16E-45	4	12.98701
Q9BTE3	642	72979.5	5.7	Mini-chror	MCMBP	MCMBP	Mini-chror	11	189.7045	58.01179	3.48E-88	11	17.44548
P33993	719	81307.2	6.38	DNA replic	MCM7	MCM7	DNA replic	17	1132.646	52.67246	1.67E-66	17	29.90264
Q14566	821	92888.4	5.09	DNA replic	MCM6	MCM6	DNA replic	14	643.6466	55.61495	1.87E-78	14	20.95006
P33992	734	82284.7	8.5	DNA replic	MCM5	MCM5	DNA replic	21	570.3447	49.24825	1.61E-53	21	33.24251
P33991	863	96557	6.71	DNA replic	MCM4	MCM4	DNA replic	7	265.6656	51.8178	3.62E-63	7	13.20973
O60318	1980	218402.6	6.35	Germinal-c	MCM3AP	MCM3AP	Germinal-c	3	98.07843	51.79374	4.47E-63	3	2.222222
P25205	808	90979.9	5.48	DNA replic	MCM3	MCM3	DNA replic	24	1316.566	51.58607	2.8E-62	24	35.27228
P49736	904	101895.2	5.2	DNA replic	MCM2	MCM2	DNA replic	16	457.1403	52.11605	2.53E-64	16	20.90708
Q9HCC0	563	61332.7	7.75	Methylcrot	MCCC2	MCCC2	Methylcrot	7	301.6073	49.12112	4.61E-53	7	15.45293
Q96RQ3	725	80472.4	7.86	Methylcrot	MCCC1	MCCC1	Methylcrot	4	83.23154	49.65056	5.45E-55	4	7.862069
Q8IVS2	390	42961.2	8.88	Malonyl-C	MCAT	MCAT	Malonyl-C	8	809.1968	51.97946	8.63E-64	8	25.89744
P43121	646	71606.9	5.5	Cell surfac	MCAM	MCAM	Cell surfac	4	383.5783	52.40436	1.87E-65	4	7.120743
Q14703	1052	117747.7	8.98	Membrane	MBTPS1	MBTPS1	Membrane	1	37.55134	52.96752	1.15E-67	1	2.376426
Q96N66	472	52764.3	9.11	Lysophosp	MBOAT7	MBOAT7	Lysophosp	5	388.5239	53.59121	3.82E-70	5	14.61864
Q5VZF2	373	40517.3	8.45	Muscleblin	MBNL2	MBNL2	Muscleblin	4	639.9939	50.07173	1.53E-56	2	9.115282
O95983	291	32843.8	4.98	Methyl-Cp	MBD3	MBD3	Methyl-Cp	4	82.98083	47.09854	4.9E-46	4	17.18213
Q9UBB5	411	43254.3	10.76	Methyl-Cp	MBD2	MBD2	Methyl-Cp	8	1005.586	50.31741	1.85E-57	8	21.16788
Q9UIS9	605	66606.5	9.25	Methyl-Cp	MBD1	MBD1	Methyl-Cp	9	743.7924	50.21204	4.59E-57	9	16.36364
Q8IYB1	491	55799.6	7.04	Nucleotidy	MB21D2	MB21D2	Nucleotidy	17	5712.793	51.74828	6.68E-63	17	39.91853
P61244	160	18274.6	6.25	Protein ma	MAX	MAX	Protein ma	4	233.8764	51.16031	1.2E-60	4	23.125
Q7Z434	540	56527.5	5.2	Mitochonc	MAVS	MAVS	Mitochonc	4	109.6796	48.8307	5.06E-52	4	10.18519
P43243	847	94622.4	6.19	Matrin-3	MATR3	MATR3	Matrin-3	23	5820.808	53.76212	7.84E-71	23	34.59268
Q9NZL9	334	37551.5	7.39	Methionine	MAT2B	MAT2B	Methionine	5	523.5911	49.32138	8.74E-54	5	16.16766
P31153	395	43660.4	6.45	S-adenosy	MAT2A	MAT2A	S-adenosy	10	544.0048	50.02439	2.29E-56	9	31.39241
Q9Y2H9	1570	170675.2	8.6	Microtubu	MAST1	MAST1	Microtubu	2	125.2149	36.07705	9E-18	2	1.464968
Q96GW9	593	66590.3	8.14	Methionine	MARS2	MARS2	Methionine	3	54.8811	41.16105	1.21E-28	3	6.23946
P56192	900	101114.9	6.05	Methionine	MARS1	MARS1	Methionine	13	259.2072	52.69419	1.38E-66	13	22
Q96L34	752	82518.9	10.18	MAP/micro	MARK4	MARK4	MAP/micro	1	46.77964	36.02194	1.13E-17	1	1.728723
P27448	753	84427.7	10.11	MAP/micro	MARK3	MARK3	MAP/micro	6	358.6685	45.96834	2.42E-42	3	9.694555
Q7KZI7	788	87910	10.3	Serine/thre	MARK2	MARK2	Serine/thre	7	304.5784	47.19161	2.39E-46	7	10.53299
Q9Y4F3	1742	192857.5	7.9	Meiosis re	MARF1	MARF1	Meiosis re	3	72.51294	47.14743	3.36E-46	3	1.894374
P49006	195	19528.7	4.34	MARCKS-r	MARCKSL1	MARCKSL1	MARCKS-r	2	92.3196	38.02786	1.68E-21	2	14.35897
P29966	332	31554.2	4.13	Myristoyla	MARCKS	MARCKS	Myristoyla	4	96.07423	46.07476	1.11E-42	4	18.9759

Q9NX47	278	31231.4	8.83	E3 ubiquiti	MARCHF5	MARCHF5	E3 ubiquiti	1	63.52569	44.02166	2.14E-36	1	4.676259
P10636	758	78927	6.69	Microtubu	MAPT	MAPT	Microtubu	1	170.0962	46.13783	6.95E-43	1	1.583113
Q9UPY8	281	31982	5.23	Microtubu	MAPRE3	MAPRE3	Microtubu	3	337.3604	51.49625	6.16E-62	2	9.964413
Q15555	327	37031.1	5.28	Microtubu	MAPRE2	MAPRE2	Microtubu	3	40.46562	41.19983	9.7E-29	3	11.6208
Q15691	268	29998.9	4.76	Microtubu	MAPRE1	MAPRE1	Microtubu	2	270.2203	41.405	2.94E-29	2	10.44776
Q8IW41	473	54220	7.86	MAP kinas	MAPKAPK!	MAPKAPK!	MAP kinas	1	191.8483	45.82132	7.09E-42	1	1.691332
Q9BPZ7	522	59122.3	7.61	Target of r	MAPKAP1	MAPKAP1	Target of r	3	67.17003	47.86257	1.26E-48	3	6.704981
Q16659	721	82680.1	4.67	Mitogen-a	MAPK6	MAPK6	Mitogen-a	1	207.9518	23.76612	2.36E-06	1	2.080444
P31152	587	65921	5.11	Mitogen-a	MAPK4	MAPK4	Mitogen-a	1	232.9165	26.51545	2.84E-07	1	1.53322
P27361	379	43135.2	6.74	Mitogen-a	MAPK3	MAPK3	Mitogen-a	11	1008.747	49.35126	6.8E-54	11	33.24538
Q8TD08	544	59831.6	9.18	Mitogen-a	MAPK15	MAPK15	Mitogen-a	1	365.5316	41.92973	1.3E-30	1	1.470588
Q16539	360	41292.9	5.58	Mitogen-a	MAPK14	MAPK14	Mitogen-a	2	74.80681	45.09818	1.28E-39	1	5
P28482	360	41389.3	6.99	Mitogen-a	MAPK1	MAPK1	Mitogen-a	14	5097.579	52.67201	1.68E-66	11	40.27778
Q3KQU3	841	92819	10.87	MAP7 dor	MAP7D1	MAP7D1	MAP7 dor	9	330.6714	47.43482	3.63E-47	9	13.91201
Q14244	749	84051	10.33	Ensconsin	MAP7	MAP7	Ensconsin	9	324.1433	49.03249	9.56E-53	9	12.9506
O95819	1239	142099.8	7.49	Mitogen-a	MAP4K4	MAP4K4	Mitogen-a	6	178.7727	54.53781	5.44E-74	6	6.779661
Q8IVH8	894	101315.1	7.6	Mitogen-a	MAP4K3	MAP4K3	Mitogen-a	2	87.70313	48.45052	1.13E-50	2	2.237136
Q12851	820	91555.1	6.31	Mitogen-a	MAP4K2	MAP4K2	Mitogen-a	1	92.51292	35.48997	9.31E-17	1	1.585366
P27816	1152	121003.8	5.07	Microtubu	MAP4	MAP4	Microtubu	18	536.8792	51.23002	6.51E-61	18	20.22569
Q9NYL2	800	91154.3	7.95	Mitogen-a	MAP3K20	MAP3K20	Mitogen-a	8	189.0356	49.91911	5.64E-56	8	11.25
O14733	419	47484.5	9.62	Dual speci	MAP2K7	MAP2K7	Dual speci	3	91.08554	47.92607	7.59E-49	3	8.114558
P45985	399	44287.3	8.19	Dual speci	MAP2K4	MAP2K4	Dual speci	3	84.42928	46.01634	1.7E-42	3	10.52632
P46734	347	39318.1	7.5	Dual speci	MAP2K3	MAP2K3	Dual speci	6	196.569	52.59053	3.5E-66	5	21.61383
P36507	400	44423.7	6.52	Dual speci	MAP2K2	MAP2K2	Dual speci	8	230.8462	47.91597	8.22E-49	4	25.25
Q02750	393	43438.6	6.61	Dual speci	MAP2K1	MAP2K1	Dual speci	2	28.59909	27.25539	1.2E-07	2	8.396947
P11137	1827	199524.5	4.54	Microtubu	MAP2	MAP2	Microtubu	4	164.4593	53.72981	1.07E-70	4	2.572523
Q66K74	1059	112210.1	7.32	Microtubu	MAP1S	MAP1S	Microtubu	8	197.6943	52.09155	3.14E-64	8	8.876298
P46821	2468	270632	4.44	Microtubu	MAP1B	MAP1B;MA	Microtubu	1	137.5074	26.41482	3.16E-07	1	0.405186
P27338	520	58762.5	7.55	Amine oxi	MAOB	MAOB	Amine oxi	6	255.9763	56.64004	1.03E-82	6	13.84615
P21397	527	59681.3	7.96	Amine oxi	MAOA	MAOA	Amine oxi	6	188.7169	44.85207	7.22E-39	5	11.95446
Q16706	1144	131139.5	7.61	Alpha-mai	MAN2A1	MAN2A1	Alpha-mai	1	102.0062	49.45534	2.83E-54	1	1.136364
P33908	653	72967.9	6.44	Mannosyl-	MAN1A1	MAN1A1	Mannosyl-	5	143.3081	49.42322	3.72E-54	5	10.87289
Q9UDY8	824	92271.6	5.51	Mucosa-as	MALT1	MALT1	Mucosa-as	6	158.7734	50.99986	4.91E-60	6	8.373786
Q96EH3	234	26169.8	5.17	Mitochonc	MALSU1	MALSU1	Mitochonc	2	54.53997	54.47223	1.01E-73	2	10.68376
Q969L2	176	19125.1	6.21	Protein MA	MAL2	MAL2	Protein MA	1	3983.965	45.41167	1.38E-40	1	6.25
Q9BXY0	300	35368.2	5.01	Protein MA	MAK16	MAK16	Protein MA	6	1095.809	52.04789	4.65E-64	6	27.66667
Q8WWC4	291	32544.3	9.58	m-AAA pr	MAIP1	MAIP1	m-AAA pr	2	226.4348	48.22473	6.98E-50	2	8.591065
Q9H0U3	335	38036.4	10.07	Magnesiur	MAGT1	MAGT1	Magnesiur	4	153.0096	48.00474	4.05E-49	4	13.43284
Q96A72	148	17275.6	6.36	Protein ma	MAGOHB	MAGOHB	Protein ma	8	988.7035	52.56834	4.26E-66	1	61.48649
Q96QZ7	1491	164580	7.61	Membrane	MAGI1	MAGI1	Membrane	14	432.7614	48.99653	1.29E-52	14	10.86519
Q9UNF1	606	64953.5	9.93	Melanoma	MAGED2	MAGED2	Melanoma	1	91.6907	38.85586	2.99E-23	1	2.145215
O15479	319	35276.8	9.21	Melanoma	MAGEB2	MAGEB2	Melanoma	11	4432.194	50.66658	9.03E-59	11	36.67712
O15525	162	17849.4	10.76	Transcripti	MAFG	MAFG	Transcripti	2	63.43305	38.61317	9.97E-23	2	15.4321
Q9ULX9	164	17760.2	10.52	Transcripti	MAFF	MAFF	Transcripti	1	99.05572	40.91039	5.09E-28	1	9.756098
Q7L5Y9	396	45286.9	8.87	E3 ubiquiti	MAEA	MAEA	E3 ubiquiti	6	217.3219	50.61914	1.37E-58	6	15.90909
Q13257	205	23509.7	4.72	Mitotic spi	MAD2L1	MAD2L1	Mitotic spi	3	203.9584	50.25377	3.21E-57	3	17.07317
Q9P0M6	372	40057.8	10.39	Core histor	MACROH2	MACROH2	Core histor	21	10434.39	49.78091	1.82E-55	21	51.6129
O75367	369	39183.2	10.52	Core histor	MACROH2	MACROH2	Core histor	26	87267.98	51.95305	1.09E-63	25	55.28455
Q9BQ69	325	35504.7	10.01	ADP-ribos	MACROD1	MACROD1	ADP-ribos	1	359.5493	45.69308	1.8E-41	1	3.384615
Q8N5G2	664	76176.9	9.56	Macoilin	MACO1	MACO1	Macoilin	1	57.61694	37.68324	8.37E-21	1	1.807229
Q9UPN3	7388	838301.1	5.06	Microtubu	MACF1	MACF1	Microtubu	8	221.0456	42.85942	4.18E-33	1	1.407688
P20645	277	30993.1	5.64	Cation-de	M6PR	M6PR	Cation-de	1	109.1433	37.51103	1.84E-20	1	3.249097
Q9BRK4	669	72758.7	6.46	Leucine zi	LZTS2	LZTS2	Leucine zi	4	151.1603	36.89217	2.9E-19	4	6.726457
P61626	148	16536.9	9.35	Lysozyme	LYZ	LYZ	Lysozyme	1	542.8358	46.80781	4.55E-45	1	8.108108
Q5VWZ2	237	26316.2	7.95	Lysophosp	LYPLAL1	LYPLAL1	Lysophosp	1	230.7499	42.99838	1.73E-33	1	4.219409
O95372	231	24736.7	7.25	Acyl-prote	LYPLA2	LYPLA2	Acyl-prote	1	81.31763	42.60148	2.12E-32	1	7.792208
O75608	230	24669.4	6.77	Acyl-prote	LYPLA1	LYPLA1	Acyl-prote	3	285.6683	52.75388	8.08E-67	3	20.43478
O95274	346	35970.4	7.79	Ly6/PLAUF	LYPD3	LYPD3	Ly6/PLAUF	1	77.85554	47.99262	4.46E-49	1	2.890173
P07948	512	58573.6	7.13	Tyrosine-p	LYN	LYN	Tyrosine-p	12	705.2249	50.10523	1.15E-56	11	28.51563
Q9NX58	379	43633.7	10.29	Cell growtl	LYAR	LYAR	Cell growtl	17	2397.324	54.40073	2.03E-73	17	34.8285
Q86V48	1076	120273.8	8.83	Leucine zi	LUZP1	LUZP1	Leucine zi	10	285.5727	51.98222	8.42E-64	10	11.15242
O95232	432	51465.7	10.46	Luc7-like p	LUC7L3	LUC7L3	Luc7-like p	11	2759.069	53.22367	1.08E-68	11	33.10185
Q9Y383	392	46513.4	10.7	Putative RI	LUC7L2	LUC7L2	Putative RI	10	4312.579	51.44795	9.5E-62	10	21.68367
Q9NQ29	371	43727.5	10.61	Putative RI	LUC7L	LUC7L	Putative RI	10	4201.013	48.6623	2.02E-51	6	30.45822
Q96GA3	475	54854.4	4.53	Protein LT	LTV1	LTV1	Protein LT	18	5759.825	50.69242	7.21E-59	18	38.31579
P02788	710	78181.2	8.17	Lactotrans	LTF	LTF	Lactotrans	1	96.17744	28.58616	1.72E-08	1	1.267606
P09960	611	69284.6	6.1	Leukotrien	LTA4H	LTA4H	Leukotrien	6	235.4141	52.33799	3.4E-65	6	10.80196
P48449	732	83308.1	6.6	Lanosterol	LSS	LSS	Lanosterol	4	249.4877	47.2522	1.5E-46	4	6.010929
Q86X29	649	71438.1	8.01	Lipolysis-s	LSR	LSR	Lipolysis-s	8	445.4678	51.65497	1.53E-62	8	17.56549
P62310	102	11845.3	4.32	U6 snRNA	LSM3	LSM3	U6 snRNA	1	219.1434	52.04687	4.69E-64	1	11.76471
Q9BX40	385	42070.4	10.23	Protein LSI	LSM14B	LSM14B	Protein LSI	1	330.786	51.5671	3.32E-62	1	2.857143
Q8ND56	463	50529.5	10.03	Protein LSI	LSM14A	LSM14A	Protein LSI	4	190.686	49.85397	9.8E-56	4	9.287257
Q3MHD2	195	21700.6	7.87	Protein LSI	LSM12	LSM12	Protein LSI	5	692.6555	52.20078	1.17E-64	5	31.79487
P83369	360	39499.2	11.55	U7 snRNA	LSM11	LSM11	U7 snRNA	2	113.4144	42.94144	2.48E-33	2	6.388889
Q9H089	658	75224.8	6.34	Large subu	LSG1	LSG1	Large subu	28	4549.718	51.62487	1.99E-62	28	44.52888
Q9UFC0	647	70860.5	7.22	Leucine-ric	LRWD1	LRWD1	Leucine-ric	5	174.5937	56.19973	6.48E-81	5	11.59196
Q9Y608	721	82170.6	6.94	Leucine-ric	LRRFIP2	LRRFIP2	Leucine-ric	6	495.4001	51.06557	2.76E-60	5	9.015257
Q96AG4	307	34930.1	10.29	Leucine-ric	LRRC59	LRRC59	Leucine-ric	10	2103.063	53.17953	1.63E-68	10	39.08795
Q8N9N7	239	26753.9	8.6	Leucine-ric	LRRC57	LRRC57	Leucine-ric	3	83.08676	44.81509	9.35E-39	3	15.06276
Q8N1G4	583	63472.2	8.4	Leucine-ric	LRRC47	LRRC47	Leucine-ric	14	738.7043	53.54065	6.01E-70	14	29.33105
Q9Y546	428	48570.9	7.5	Leucine-ric	LRRC42	LRRC42	Leucine-ric	2	280.2407	43.78306	1.05E-35	2	4.672897
Q9BTT6	524	59241	4.66	Leucine-ric	LRRC1	LRRC1	Leucine-ric	8	283.5361	52.9181	1.81E-67	8	22.70992
P42704	1394	157903.4	6	Leucine-ric	LRPPRC	LRPPRC	Leucine-ric	36	1038.262	54.94021	1.13E-75	36	28.40746
Q5T3J3	769	84567.6	10.39	Ligand-de	LRIF1	LRIF1	Ligand-de	5	94.55978	50.05238	1.8E-56	5	8.972692
O75427	683	73449.4	8.29	Leucine-ric	LRCH4	LRCH4	Leucine-ric	2	131.1401	45.37183	1.83E-40	2	4.392387
Q96II8	777	86082.4	6.71	DISP comp	LRCH3	LRCH3	DISP comp	7	352.831	52.05653	4.3E-64	7	13.77091
Q9Y2L9	728	80874.5	5.83	Leucine-ric	LRCH1	LRCH1	Leucine-ric	8	416.5379	50.69422	7.11E-59	7	15.24725
P50851	2863	319105	5.31	Lipopolysa	LRBA	LRBA	Lipopolysa	11	290.9379	54.0526	5.08E-72	9	4.994761
Q93052	612	65745.8	7.39	Lipoma-pr	LPP	LPP	Lipoma-pr	2	48.95542	42.47823	4.55E-32	2	5.555556
Q92604	370	43089.1	9.24	Acyl-CoA:l	LPGAT1	LPGAT1	Acyl-CoA:l	2	89.92357	53.52236	7.06E-70	2	6.216216
Q7L5N7	544	60207.3	6.51	Lysophosp	LPCAT2	LPCAT2	Lysophosp	1	101.318	44.78637	1.14E-38	1	2.022059
Q8NF37	534	59150.7	5.82	Lysophosp	LPCAT1	LPCAT1	Lysophosp	6	209.3079	48.72001	1.26E-51	6	12.73408
Q9Y4K0	774	86724.3	6.35	Lysyl oxida	LOXL2	LOXL2	Lysyl oxida	2	72.29432	51.55716	3.62E-62	2	2.842377

Q86WA8	852	94615.6	7.33	Lon protea	LONP2	LONP2	Lon protea	5	80.31718	36.49488	1.59E-18	5	6.220657
P36776	959	106488.4	6.34	Lon protea	LONP1	LONP1	Lon protea	13	513.8281	51.76231	5.89E-63	13	16.68405
Q8N448	690	76003.3	6.62	Ligand of I	LNX2	LNX2	Ligand of I	1	53.30872	23.10398	3.3E-06	1	1.594203
Q8TBB1	728	80627.7	7.1	E3 ubiquiti	LNX1	LNX1	E3 ubiquiti	2	47.04673	32.987	5.53E-13	2	3.021978
Q9UIQ6	1025	117348.1	5.5	Leucyl-cys	LNPEP	LNPEP	Leucyl-cys	1	152.1512	39.57356	7.56E-25	1	0.780488
Q8WWI1	1683	192693.9	8.19	LIM domai	LMO7	LMO7	LIM domai	2	16.99381	28.87375	1.06E-08	2	1.604278
Q03252	620	69947.7	5.28	Lamin-B2	LMNB2	LMNB2	Lamin-B2	31	4560.944	56.31367	2.28E-81	28	47.25806
P20700	586	66407.7	4.82	Lamin-B1	LMNB1	LMNB1	Lamin-B1	27	2621.686	56.16175	9.27E-81	27	45.39249
P02545	664	74138.8	7.01	Prelamin-/	LMNA	LMNA	Prelamin-/	40	21171.94	51.55555	3.67E-62	40	62.1988
Q9BU23	707	79697.2	10.53	Lipase mat	LMF2	LMF2	Lipase mat	2	102.1534	25.7096	6.19E-07	2	3.253182
Q12907	356	40228.4	6.95	Vesicular i	LMAN2	LMAN2	Vesicular i	4	194.3873	55.68964	9.22E-79	4	10.95506
P49257	510	57548.7	6.76	Protein ER	LMAN1	LMAN1	Protein ER	3	337.2777	46.93539	1.72E-45	3	10.19608
Q9BRT6	129	15225.1	11.14	Protein LLI	LLPH	LLPH	Protein LLI	2	204.8802	50.08214	1.4E-56	2	10.85271
Q6P1M3	1020	113447	7.54	LLGL scribl	LLGL2	LLGL2	LLGL scribl	15	1025.889	49.6404	5.94E-55	15	18.13725
Q15334	1064	115416.9	6.25	Lethal(2) g	LLGL1	LLGL1	Lethal(2) g	12	372.0252	52.77401	6.74E-67	12	14.75564
Q8IVB5	337	36562.4	8.73	LIX1-like p	LIX1L	LIX1L	LIX1-like p	1	215.4237	36.87173	3.17E-19	1	5.341246
P38571	399	45418.7	6.91	Lysosomal	LIPA	LIPA	Lysosomal	1	172.2567	41.33841	4.34E-29	1	2.756892
Q8WXQ3	324	35482	6.22	Putative ur	LINC01599	LINC01599	Putative ur	1	92.76672	26.17407	4.03E-07	1	2.469136
Q5TKA1	542	61945.2	9.49	Protein lin	LIN9	LIN9	Protein lin	1	72.73573	43.24404	3.58E-34	1	2.767528
Q9NUP9	197	21833.8	8.7	Protein lin	LIN7C	LIN7C	Protein lin	4	240.6494	52.12872	2.26E-64	3	26.90355
P53667	647	72584.4	6.98	LIM domai	LIMK1	LIMK1	LIM domai	2	90.21146	48.26857	4.91E-50	2	3.709428
Q9UGP4	676	72189.6	6.64	LIM domai	LIMD1	LIMD1	LIM domai	1	39.80864	43.84092	7.16E-36	1	2.95858
Q9UPQ0	1083	121866.2	6.41	LIM and ca	LIMCH1	LIMCH1	LIM and ca	10	305.8076	55.65593	1.26E-78	10	10.988
Q9UHB6	759	85224.9	6.84	LIM domai	LIMA1	LIMA1	LIM domai	31	1466.274	52.76967	7.01E-67	31	45.71805
P49916	1009	112906	9.43	DNA ligase	LIG3	LIG3	DNA ligase	46	7988.099	52.33281	3.55E-65	46	43.70664
Q99538	433	49410.9	6.54	Legumain	LGMN	LGMN	Legumain	1	23.26441	40.58963	3.1E-27	1	2.540416
O00214	317	35807.8	8.46	Galectin-8	LGALS8	LGALS8	Galectin-8	8	547.2416	51.92371	1.42E-63	8	26.81388
Q08380	585	65330.3	4.9	Galectin-3	LGALS3BP	LGALS3BP	Galectin-3	11	1045.39	50.3254	1.73E-57	11	19.48718
P17931	250	26152.2	8.82	Galectin-3	LGALS3	LGALS3	Galectin-3	5	315.5725	50.4638	5.27E-58	5	25.6
P09382	135	14715.6	5.15	Galectin-1	LGALS1	LGALS1	Galectin-1	5	2786.761	49.08088	6.42E-53	5	48.88889
Q6P1Q0	360	41789.9	10.91	LETM1 dor	LETMD1	LETMD1	LETM1 dor	1	31.01925	34.66179	2.07E-15	1	3.055556
O95202	739	83353.4	6.66	Mitochonc	LETM1	LETM1	Mitochonc	4	189.0413	48.21045	7.82E-50	4	6.7659
Q8WVC0	666	75403.4	4.12	RNA polyn	LEO1	LEO1	RNA polyn	7	596.5085	49.1527	3.55E-53	7	11.56156
Q96PV6	800	88156.7	9.42	Leukocyte	LENG8	LENG8	Leukocyte	11	351.4587	52.51223	7.04E-66	11	19.375
Q9Y2U8	911	99996.5	7.58	Inner nucle	LEMD3	LEMD3	Inner nucle	5	458.0789	53.23315	9.97E-69	5	6.805708
Q8NC56	503	56974.5	9.3	LEM doma	LEMD2	LEMD2	LEM doma	11	514.374	53.05839	4.96E-68	11	21.86879
P01130	860	95375.1	4.64	Low-densi	LDLR	LDLR	Low-densi	1	95.85278	36.91694	2.61E-19	1	1.27907
P07195	334	36638.2	5.93	L-lactate d	LDHB	LDHB	L-lactate d	8	1095.898	50.76754	3.76E-59	8	32.93413
Q9BYZ2	381	41942.5	8.89	L-lactate d	LDHAL6B	LDHAL6B	L-lactate d	1	74.10398	46.75178	6.95E-45	1	4.461942
P00338	332	36688.5	8.45	L-lactate d	LDHA	LDHA	L-lactate d	7	1232.804	54.24567	8.41E-73	6	22.89157
P31025	176	19249.8	5.3	Lipocalin-1	LCN1	LCN1;LCN1	Lipocalin-1	1	156.1319	43.85709	6.44E-36	1	6.25
Q6UWP7	414	48919.8	8.8	Lysocardio	LCLAT1	LCLAT1	Lysocardio	2	167.2622	43.58328	3.94E-35	2	5.072464
Q86VQ0	697	80553.5	7.82	Lebercilin	LCA5	LCA5	Lebercilin	1	81.15987	31.81953	1.58E-11	1	1.578192
Q14739	615	70702.5	9.72	Delta(14)-s	LBR	LBR	Delta(14)-s	7	412.8655	49.75057	2.34E-55	7	12.03252
Q9GZY6	243	26550	4.41	Linker for a	LAT2	LAT2	Linker for a	2	81.1629	43.27753	2.89E-34	2	12.7572
Q14847	261	29717.1	7.07	LIM and SH	LASP1	LASP1	LIM and SH	8	553.5038	51.41565	1.26E-61	8	27.20307
Q9Y4W2	734	83064.2	4.36	Ribosomal	LAS1L	LAS1L	Ribosomal	16	800.4268	53.53946	6.06E-70	16	26.83924
Q15031	903	101975.4	8.32	Leucine--t	LARS2	LARS2	Leucine--t	5	145.4289	42.54555	2.99E-32	5	5.426357
Q9P2J5	1176	134465.2	7.31	Leucine--t	LARS1	LARS1	Leucine--t	23	2062.156	51.79678	4.36E-63	23	21.68367
Q4G0J3	582	66898.2	10.27	La-related	LARP7	LARP7	La-related	32	8486.095	50.69856	6.85E-59	32	50.68729
Q92615	738	80551.2	6.91	La-related	LARP4B	LARP4B	La-related	12	354.9262	49.87415	8.25E-56	11	17.61518
Q71RC2	724	80595.4	6.58	La-related	LARP4	LARP4	La-related	14	1161.544	50.98708	5.5E-60	14	25.55249
Q659C4	914	105321.5	7.67	La-related	LARP1B	LARP1B	La-related	19	571.315	49.15393	3.96E-53	14	22.97593
Q6PKG0	1096	123509.5	9.24	La-related	LARP1	LARP1	La-related	21	3921.815	54.93542	1.17E-75	21	21.89781
P28838	519	56165.8	8.05	Cytosol am	LAP3	LAP3	Cytosol am	5	168.5433	52.07653	3.58E-64	5	12.33141
Q9NS86	450	50853.9	7.46	LanC-like	LANCL2	LANCL2	LanC-like	12	1038.316	51.19855	8.59E-61	12	33.11111
O43813	399	45282.7	7.81	Glutathion	LANCL1	LANCL1	Glutathion	13	4753.97	49.28452	1.19E-53	13	38.59649
Q0VGL1	99	10741.2	6.52	Ragulator	LAMTOR4	LAMTOR4	Ragulator	1	741.9277	48.29675	3.91E-50	1	10.10101
Q9UHA4	124	13622.6	7.51	Ragulator	LAMTOR3	LAMTOR3	Ragulator	1	358.4312	39.89227	1.4E-25	1	12.90323
Q9Y2Q5	125	13507.4	5.04	Ragulator	LAMTOR2	LAMTOR2	Ragulator	3	117.1425	44.93488	4.04E-39	3	28.8
Q6IAA8	161	17744.6	4.79	Ragulator	LAMTOR1	LAMTOR1	Ragulator	5	313.3091	52.54584	5.24E-66	5	42.85714
P13473	410	44960.4	5.34	Lysosome-	LAMP2	LAMP2	Lysosome-	2	317.4941	50.27193	2.74E-57	2	4.878049
P11279	417	44881.9	8.92	Lysosome-	LAMP1	LAMP1	Lysosome-	2	694.8292	44.0018	2.44E-36	2	4.796163
Q13753	1193	130974.7	6.09	Laminin su	LAMC2	LAMC2	Laminin su	7	282.2598	45.24163	4.64E-40	7	7.544007
P11047	1609	177601.2	4.76	Laminin su	LAMC1	LAMC1	Laminin su	9	264.6175	54.30255	5.08E-73	9	7.271597
Q13751	1172	129571.4	7.22	Laminin su	LAMB3	LAMB3	Laminin su	8	167.0139	52.27525	5.96E-65	8	9.47099
P55268	1798	195979.2	6.5	Laminin su	LAMB2	LAMB2	Laminin su	6	258.1848	46.16465	5.7E-43	6	4.560623
P07942	1786	198036.5	4.57	Laminin su	LAMB1	LAMB1	Laminin su	3	172.3332	53.11889	2.87E-68	3	2.015677
O15230	3695	399733.5	7.01	Laminin su	LAMA5	LAMA5	Laminin su	5	110.9172	37.61567	1.14E-20	5	2.02977
Q16787	3333	366616.1	7.24	Laminin su	LAMA3	LAMA3	Laminin su	3	140.2432	44.13924	9.68E-37	3	1.020102
O00515	517	57130.4	10.42	Ladinin-1	LAD1	LAD1	Ladinin-1	5	299.6849	52.54261	5.39E-66	5	12.18569
Q53H82	288	32805.4	6.79	Endoribon	LACTB2	LACTB2	Endoribon	1	55.32621	35.59031	6.29E-17	1	3.125
P83111	547	60693.1	8.75	Serine beta	LACTB	LACTB	Serine beta	9	172.3395	47.3056	9.91E-47	9	18.64717
Q96JM7	780	88336	6.41	Lethal(3)m	L3MBTL3	L3MBTL3	Lethal(3)m	6	196.7864	48.10715	1.78E-49	6	8.589744
Q969R5	705	79109.5	6.84	Lethal(3)m	L3MBTL2	L3MBTL2	Lethal(3)m	2	202.8313	43.12602	7.64E-34	2	3.120567
Q9H9P8	463	50315.6	8.23	L-2-hydro	L2HGDH	L2HGDH	L-2-hydro	7	195.6216	48.32999	2.99E-50	7	17.92657
Q9UN81	338	40055.4	10.15	LINE-1 ret	L1RE1	L1RE1	LINE-1 ret	2	96.90638	27.03663	1.57E-07	2	6.804734
Q86UP2	1357	156274.5	5.34	Kinectin	KTN1	KTN1	Kinectin	4	64.16245	38.07485	1.34E-21	4	3.463522
Q8N6L1	136	14678.4	10.09	Keratinocy	KRTCAP2	KRTCAP2	Keratinocy	1	131.9219	42.44939	5.45E-32	1	12.5
P35527	623	62064.3	4.89	Keratin, typ	KRT9	KRT9	Keratin, typ	22	10110.44	51.03108	3.73E-60	22	38.68379
O43790	486	53500.1	5.35	Keratin, typ	KRT86	KRT86;KRT	Keratin, typ	3	137.7	46.46287	6.15E-44		6.17284
Q6KB66	452	50524.8	5.36	Keratin, typ	KRT80	KRT80	Keratin, typ	2	82.12255	43.44961	9.44E-35	2	4.867257
P05787	483	53703.8	5.26	Keratin, typ	KRT8	KRT8	Keratin, typ	29	16494.34	51.50948	5.49E-62	25	52.58799
Q8N1N4	520	56865.4	5.83	Keratin, typ	KRT78	KRT78	Keratin, typ	5	348.1677	46.00325	1.87E-42	4	10.96154
Q7Z794	578	61901.1	5.78	Keratin, typ	KRT77	KRT77	Keratin, typ	4	173.1102	47.08872	5.28E-46	4	6.920415
Q86Y46	540	58922.8	7.26	Keratin, typ	KRT73	KRT73	Keratin, typ	2	9483.321	45.34927	2.15E-40	1	4.259259
P04259	564	60066.5	8.16	Keratin, typ	KRT6B	KRT6B	Keratin, typ	5	10164.29	53.62791	2.76E-70	2	9.929078
P02538	564	60044.5	8.16	Keratin, typ	KRT6A	KRT6A	Keratin, typ	28	11268.22	52.81023	4.86E-67	2	48.93617
P13647	590	62378	7.91	Keratin, typ	KRT5	KRT5	Keratin, typ	13	1801.242	53.48583	9.79E-70	13	24.57627
Q92764	455	50360.1	4.55	Keratin, typ	KRT35	KRT35	Keratin, typ	1	133.513	46.66544	1.34E-44		2.637363
Q9C075	422	48130.8	6.52	Keratin, typ	KRT23	KRT23	Keratin, typ	3	28.3728	39.6968	3.95E-25	3	7.819905
P35908	639	65432.6	8.17	Keratin, typ	KRT2	KRT2	Keratin, typ	31	15442.98	52.3568	2.88E-65	27	53.83412
P08727	400	44105.6	4.78	Keratin, typ	KRT19	KRT19	Keratin, typ	20	8227.575	53.28899	6.01E-69	15	51

P05783	430	48057.4	5.11	Keratin, ty	KRT18	KRT18	Keratin, ty	23	5080.335	52.03777	5.09E-64	22	52.55814
Q04695	432	48105.3	4.67	Keratin, ty	KRT17	KRT17	Keratin, ty	6	187.9305	47.46646	2.83E-47	5	15.50926
P08779	473	51267.4	4.69	Keratin, ty	KRT16	KRT16	Keratin, ty	8	361.8839	50.05521	1.76E-56	8	20.93023
P19012	456	49211.4	4.42	Keratin, ty	KRT15	KRT15	Keratin, ty	1	3516.45	33.35177	1.77E-13		2.412281
P02533	472	51561.1	4.81	Keratin, ty	KRT14	KRT14	Keratin, ty	21	4627.624	50.09069	1.3E-56	6	47.24576
P13645	584	58826.9	4.88	Keratin, ty	KRT10	KRT10	Keratin, ty	24	18290.81	51.65429	1.53E-62	20	36.30137
P04264	644	66038.5	8.33	Keratin, ty	KRT1	KRT1	Keratin, ty	27	14688.83	53.48808	9.6E-70	27	40.06211
Q13601	381	43664.6	10.48	KRR1 smal	KRR1	KRR1	KRR1 smal	6	165.074	49.2356	1.78E-53	6	15.48556
Q8N9T8	703	82597.4	4.78	Protein KR	KRI1	KRI1	Protein KR	11	559.1422	54.66697	1.57E-74	11	18.49218
P01116	189	21655.7	6.76	GTPase KR	KRAS	KRAS	GTPase KR	5	324.1755	46.02407	1.61E-42	3	26.98413
Q5T749	579	64135.2	8.29	Keratinocy	KPRP	KPRP	Keratinocy	5	116.5142	44.94632	3.73E-39	5	14.16235
Q14974	876	97169.2	4.41	Importin su	KPNB1	KPNB1	Importin su	14	563.7408	53.4935	9.17E-70	14	20.54795
A9QM74	516	56937.3	5.89	Importin su	KPNA7	KPNA7	Importin su	1	568.965	46.58545	2.45E-44	1	2.131783
O60684	536	60028.9	4.62	Importin su	KPNA6	KPNA6	Importin su	9	2558.235	50.36901	1.19E-57	3	20.33582
O15131	539	60665.8	4.76	Importin su	KPNA5	KPNA5	Importin su	1	116.2739	25.03462	1.06E-06	1	1.855288
O00629	521	57886.3	4.56	Importin su	KPNA4	KPNA4	Importin su	3	133.9205	45.94964	2.77E-42	3	7.677543
O00505	521	57810.4	4.55	Importin su	KPNA3	KPNA3	Importin su	7	1338.039	50.45236	5.81E-58	5	22.07294
P52292	529	57861.4	5.05	Importin su	KPNA2	KPNA2	Importin su	9	686.2515	51.64965	1.6E-62	9	25.89792
P52294	538	60221.2	4.65	Importin su	KPNA1	KPNA1	Importin su	5	471.5359	49.66074	5E-55	5	10.78067
Q86Y97	462	52112.4	10.31	Histone-ly	KMT5C	KMT5C	Histone-ly	3	269.5715	45.90111	3.96E-42	3	7.142857
Q4FZB7	885	99187	9.05	Histone-ly	KMT5B	KMT5B	Histone-ly	3	138.5708	46.57021	2.75E-44	3	4.180791
Q9NQR1	393	42889.5	10.22	N-lysine m	KMT5A	KMT5A	N-lysine m	7	111.3859	49.36476	6.08E-54	7	21.6285
O14686	5537	593384.6	5.3	Histone-ly	KMT2D	KMT2D	Histone-ly	12	273.7892	52.64165	2.19E-66	11	2.979953
Q03164	3969	431760.5	9.56	Histone-ly	KMT2A	KMT2A	Histone-ly	27	417.2379	54.44556	1.31E-73	27	8.541194
Q9P2G9	620	68801.6	6.57	Kelch-like	KLHL8	KLHL8	Kelch-like	6	106.0995	43.32999	2.06E-34	6	11.93548
Q8IXQ5	586	65991.2	6.44	Kelch-like	KLHL7	KLHL7	Kelch-like	3	153.1364	46.82751	3.92E-45	3	6.313993
Q8N4N3	616	69895.5	6.18	Kelch-like	KLHL36	KLHL36	Kelch-like	3	188.0469	51.97112	9.28E-64	3	7.142857
Q53HC5	615	68138.9	6.46	Kelch-like	KLHL26	KLHL26	Kelch-like	2	78.73439	46.01886	1.67E-42	2	3.414634
Q6TFL4	600	68360.6	6.35	Kelch-like	KLHL24	KLHL24	Kelch-like	1	42.59942	27.06244	1.53E-07	1	2.166667
Q96M94	604	69774.2	6.37	Kelch-like	KLHL15	KLHL15	Kelch-like	2	82.76744	44.37802	1.91E-37	2	5.629139
Q53G59	568	63276.5	5.11	Kelch-like	KLHL12	KLHL12	Kelch-like	4	144.885	45.00659	2.44E-39	4	9.507042
Q8TBB5	520	57891.4	5.46	Kelch dom	KLHDC4	KLHDC4	Kelch dom	4	97.23901	36.21374	5.14E-18	4	10.19231
O43474	513	54670	8.44	Krueppel-l	KLF4	KLF4	Krueppel-l	4	337.9479	46.77194	5.97E-45	3	10.91618
Q9Y4X4	402	44239.5	10.3	Krueppel-l	KLF12	KLF12	Krueppel-l	1	102.588	46.02441	1.6E-42	1	2.985075
Q07866	573	65309.1	6.11	Kinesin ligl	KLC1	KLC1;KLC2	Kinesin ligl	1	120.3148	36.76638	5E-19	1	1.570681
P21583	273	30898.2	5.92	Kit ligand	KITLG	KITLG	Kit ligand	1	58.28609	48.17666	1.02E-49	1	3.296703
O60870	393	45373.4	9.43	DNA/RNA	KIN	KIN	DNA/RNA	4	195.6945	50.39885	9.22E-58	4	12.72265
Q9BW19	673	73747	9.35	Kinesin-lik	KIFC1	KIFC1	Kinesin-lik	8	148.0624	51.71859	8.7E-63	8	16.04755
P33176	963	109684.1	6.46	Kinesin-1 l	KIF5B	KIF5B	Kinesin-1 l	10	376.2325	52.67303	1.67E-66	5	12.35722
O14782	793	89453.6	8.67	Kinesin-lik	KIF3C	KIF3C;KIF3	Kinesin-lik	1	294.3117	44.52511	6.96E-38	1	1.387137
Q99661	725	81312.2	7.9	Kinesin-lik	KIF2C	KIF2C	Kinesin-lik	4	118.4052	45.87634	4.74E-42	3	7.862069
O00139	706	79953.9	6.66	Kinesin-lik	KIF2A	KIF2A	Kinesin-lik	13	1223.901	52.31873	4.04E-65	11	19.26346
Q02241	960	110058.2	8.7	Kinesin-lik	KIF23	KIF23	Kinesin-lik	3	63.35477	44.74413	1.54E-38	3	3.4375
Q14807	665	73261.4	10.04	Kinesin-lik	KIF22	KIF22	Kinesin-lik	4	155.0366	50.66419	9.21E-59	4	8.721805
O95235	890	100277.1	6.91	Kinesin-lik	KIF20A	KIF20A	Kinesin-lik	5	127.3307	51.82004	3.56E-63	5	7.191011
O43896	1103	122945.5	6.88	Kinesin-lik	KIF1C	KIF1C	Kinesin-lik	7	301.4528	48.50105	7.51E-51	4	6.980961
Q86Y91	852	93009.9	8.78	Kinesin-lik	KIF18B	KIF18B;KIF1	Kinesin-lik	1	169.1214	40.37641	1.01E-26	1	1.29108
Q9NQT8	1826	202787	5.68	Kinesin-lik	KIF13B	KIF13B	Kinesin-lik	4	191.0759	46.09902	9.27E-43	4	2.683461
P52732	1056	119158	5.36	Kinesin-lik	KIF11	KIF11	Kinesin-lik	1	59.87603	33.14503	3.4E-13	1	1.325758
Q9ULH0	1771	196540.2	6.6	Kinase D-i	KIDINS220	KIDINS220	Kinase D-i	5	136.9808	50.70451	6.51E-59	5	2.879729
Q8IYS2	634	69156.6	8.29	Uncharact	KIAA2013	KIAA2013	Uncharact	2	126.4631	40.78836	1.02E-27	2	5.362776
Q9BY89	1806	196709.1	8.71	Uncharact	KIAA1671	KIAA1671	Uncharact	1	33.97845	30.01915	1.17E-09	1	0.830565
Q92628	1395	154787.5	4.42	Uncharact	KIAA0232	KIAA0232	Uncharact	1	86.08561	27.35796	1.05E-07	1	1.362007
Q92945	711	73115.2	7.34	Far upstrea	KHSRP	KHSRP	Far upstrea	11	838.2086	52.66764	1.74E-66	10	20.25316
O15037	678	74533.4	7.02	Protein KH	KHNYN	KHNYN	Protein KH	2	231.1321	45.08091	1.45E-39	2	3.834808
O75525	346	38799.3	7.82	KH domair	KHDRBS3	KHDRBS3	KH domair	3	267.7026	42.56631	2.63E-32	3	8.959538
Q07666	443	48227	8.96	KH domair	KHDRBS1	KHDRBS1	KH domair	9	4272.191	55.13674	1.75E-76	6	20.99323
Q14145	624	69665.8	6.42	Kelch-like	KEAP1	KEAP1	Kelch-like	2	113.6895	48.56602	4.43E-51	2	3.365385
Q06136	332	36186.8	7.18	3-ketodihy	KDSR	KDSR	3-ketodihy	3	205.6046	48.33183	2.95E-50	3	12.3494
Q6ZMT4	941	106556	8.14	Lysine-spe	KDM7A	KDM7A	Lysine-spe	5	245.7651	51.12425	1.65E-60	5	8.076514
O15054	1643	176630.7	8.66	Lysine-spe	KDM6B	KDM6B	Lysine-spe	10	373.205	50.17944	6.06E-57	10	8.82532
O15550	1401	154175.4	7.47	Lysine-spe	KDM6A	KDM6A	Lysine-spe	1	76.26652	39.4477	1.46E-24	1	1.213419
P41229	1560	175718.6	5.29	Lysine-spe	KDM5C	KDM5C;KD	Lysine-spe	1	154.2269	44.60266	4.09E-38	1	0.705128
Q9UGL1	1544	175655.8	6.68	Lysine-spe	KDM5B	KDM5B	Lysine-spe	19	248.3636	53.5809	4.18E-70	18	14.37824
P29375	1690	192093.7	6.45	Lysine-spe	KDM5A	KDM5A	Lysine-spe	11	326.5558	44.48112	7.29E-37	11	8.461538
Q6B0I6	523	58602.3	9.5	Lysine-spe	KDM4D	KDM4D	Lysine-spe	5	572.1795	47.10715	4.59E-46	3	10.13384
Q9H3R0	1056	119980.8	6.41	Lysine-spe	KDM4C	KDM4C	Lysine-spe	11	342.906	48.47445	9.32E-51	11	13.16288
O94953	1096	121895.5	7.1	Lysine-spe	KDM4B	KDM4B	Lysine-spe	14	277.0882	53.49962	8.68E-70	13	16.14964
O75164	1064	120661.3	5.64	Lysine-spe	KDM4A	KDM4A	Lysine-spe	18	381.9486	52.6252	2.55E-66	18	19.26692
Q9Y4C1	1321	147340	8.15	Lysine-spe	KDM3A	KDM3A	Lysine-spe	3	48.07739	35.04387	5.1E-16	3	3.103709
Q8NHM5	1336	152613.4	8.68	Lysine-spe	KDM2B	KDM2B	Lysine-spe	1	97.38495	16.98297	3.24E-05	1	0.823353
Q9Y2K7	1162	132791.6	7.61	Lysine-spe	KDM2A	KDM2A	Lysine-spe	28	1343.766	50.96271	6.82E-60	27	26.50602
Q8NB78	822	92097.6	7.93	Lysine-spe	KDM1B	KDM1B	Lysine-spe	13	452.0111	52.69763	1.34E-66	13	20.92457
O60341	852	92901.9	6.47	Lysine-spe	KDM1A	KDM1A	Lysine-spe	8	271.7954	53.27744	6.66E-69	8	11.61972
O43731	214	25026.5	9.18	ER lumen p	KDELR3	KDELR3	ER lumen p	1	144.3109	38.21454	6.9E-22	1	4.205607
P33947	212	24421.7	8.94	ER lumen p	KDELR2	KDELR2	ER lumen p	2	33.36707	42.77041	7.33E-33	2	12.73585
P24390	212	24541.8	8.79	ER lumen p	KDELR1	KDELR1	ER lumen p	1	20.79833	45.80287	8.11E-42	1	5.660377
Q9NXV2	234	26092.3	6.14	BTB/POZ c	KCTD5	KCTD5	BTB/POZ c	3	593.9676	46.09473	9.56E-43	3	17.52137
Q9BQ13	255	29591	8.81	BTB/POZ c	KCTD14	KCTD14	BTB/POZ c	1	106.3327	42.16552	3.12E-31	1	3.921569
Q9H3F6	313	35431.9	6.27	BTB/POZ c	KCTD10	KCTD10	BTB/POZ c	3	181.4667	48.94656	1.95E-52	3	12.14058
O43526	872	95846.6	9.59	Potassium	KCNQ2	KCNQ2	Potassium	1	142.3577	21.33692	7E-06	1	1.720183
P51787	676	74697.9	10.39	Potassium	KCNQ1	KCNQ1	Potassium	2	26.72251	34.05741	1.73E-14	2	4.142012
Q9P0J7	381	41945	5.41	E3 ubiquiti	KCMF1	KCMF1	E3 ubiquiti	1	50.19741	49.19515	2.5E-53	1	3.674541
Q9NVX7	534	59903.4	5.57	Kelch repe	KBTBD4	KBTBD4	Kelch repe	3	44.34676	37.25364	5.9E-20	3	6.367041
Q9BVA0	655	72332.9	7.6	Katanin p8	KATNB1	KATNB1	Katanin p8	2	138.5523	40.59347	3.04E-27	2	3.816794
O95251	611	70641.8	9.12	Histone ac	KAT7	KAT7	Histone ac	20	974.657	52.11671	2.51E-64	20	33.38789
Q8WYB5	2073	231376.3	5.82	Histone ac	KAT6B	KAT6B	Histone ac	13	833.6921	53.60683	3.29E-70	7	7.187651
Q92794	2004	225026.1	5.41	Histone ac	KAT6A	KAT6A	Histone ac	25	518.6818	53.33579	3.92E-69	25	14.52096
Q15046	597	68047.5	6.31	Lysine--tR	KARS1	KARS1	Lysine--tR	11	397.3937	49.66188	4.95E-55	11	15.9129
Q63ZY3	851	91173.5	5.33	KN motif a	KANK2	KANK2	KN motif a	4	297.4021	42.43598	5.92E-32	4	5.287897
P14923	745	81744	6.05	Junction p	JUP	JUP	Junction p	24	3372.644	53.21312	1.19E-68	21	44.02685
P17535	347	35173.5	7.51	Transcripti	JUND	JUND	Transcripti	2	157.2607	46.87098	2.82E-45	2	8.357349
P17275	347	35879	9.6	Transcripti	JUNB	JUNB	Transcripti	3	241.4931	46.78899	5.25E-45	3	11.52738

P05412	331	35675.3	9.11	Transcripti	JUN	JUN	Transcripti	2	41.15375	47.19428	2.34E-46	2	8.761329
Q9Y4A0	524	59911.1	8.25	Jerky prote	JRKL	JRKL	Jerky prote	5	109.6926	41.6582	6.62E-30	5	15.83969
Q9HDC5	661	71685.5	9.78	Junctophili	JPH1	JPH1	Junctophili	7	457.8319	53.02287	6.89E-68	7	13.76702
Q6NYC1	403	46461.6	8.98	Bifunctiona	JMJD6	JMJD6	Bifunctiona	9	348.4976	51.54285	4.1E-62	9	27.29529
Q9P266	1359	148349.6	7.09	Junctional	JCAD	JCAD	Junctional	9	251.3747	53.2571	8.01E-69	9	7.873436
Q92833	1246	138733.6	9.97	Protein Jur	JARID2	JARID2	Protein Jur	1	322.0695	51.53335	4.46E-62	1	0.882825
Q96AA8	810	94933	6.01	Janus kinas	JAKMIP2	JAKMIP2	Janus kinas	1	386.9672	24.40615	1.62E-06	1	0.864198
P23458	1154	133276	7.58	Tyrosine-p	JAK1	JAK1	Tyrosine-p	3	131.0782	53.00116	8.42E-68	3	3.206239
Q8N5M9	183	21124.8	10.08	Protein jag	JAGN1	JAGN1	Protein jag	1	77.24802	47.08667	5.37E-46	1	6.557377
Q92613	823	93807.4	7.18	Protein Jac	JADE3	JADE3	Protein Jac	13	1268.058	52.28342	5.56E-65	13	14.21628
Q9NQC1	790	87464.8	4.9	E3 ubiquiti	JADE2	JADE2	E3 ubiquiti	2	100.9221	40.60265	2.89E-27	2	2.151899
Q6IE81	842	95532.6	7.97	Protein Jac	JADE1	JADE1	Protein Jac	13	489.1906	53.36118	3.11E-69	12	16.50831
Q96ST2	819	91953.7	4.31	Protein IW	IWS1	IWS1	Protein IW	11	2736.025	51.89581	1.82E-63	11	16.60562
P28290	1259	138384.6	4.85	Protein ITF	ITPRID2	ITPRID2	Protein ITF	9	501.5406	51.87619	2.15E-63	9	9.849087
Q14573	2671	304104	6.45	Inositol 1,4	ITPR3	ITPR3	Inositol 1,4	29	690.6208	54.84781	2.73E-75	24	12.65444
Q14571	2701	308061.1	6.39	Inositol 1,4	ITPR2	ITPR2	Inositol 1,4	1	50.65901	33.26308	2.35E-13	1	0.481303
Q13572	414	45620.6	6.09	Inositol-te	ITPK1	ITPK1	Inositol-te	1	280.5162	45.70859	1.61E-41	1	3.140097
Q9BY32	194	21445.5	5.34	Inosine trip	ITPA	ITPA	Inosine trip	1	44.09234	55.35197	2.25E-77	1	7.216495
Q9NQX7	267	30223.5	8.11	Integral m	ITM2C	ITM2C	Integral m	2	171.2921	51.83048	3.24E-63	2	7.490637
P19823	946	106462.7	6.85	Inter-alpha	ITIH2	ITIH2	Inter-alpha	2	105.5794	46.6962	1.06E-44	2	2.748414
P16144	1822	202165.4	5.99	Integrin be	ITGB4	ITGB4	Integrin be	20	485.6118	52.0928	3.11E-64	20	13.83095
P05556	798	88414.6	5.04	Integrin be	ITGB1	ITGB1	Integrin be	15	990.5404	54.98998	7.23E-76	15	23.68421
P06756	1048	116036.9	5.36	Integrin al	ITGAV	ITGAV	Integrin al	2	88.2607	46.81454	4.33E-45	2	2.480916
P23229	1130	126604.4	6.57	Integrin al	ITGA6	ITGA6	Integrin al	8	174.0605	47.89887	9.41E-49	8	8.40708
P26006	1051	116611.3	6.76	Integrin al	ITGA3	ITGA3	Integrin al	14	1607.2	51.21951	7.13E-61	14	15.98478
P17301	1181	129295.4	4.88	Integrin al	ITGA2	ITGA2	Integrin al	13	546.8385	51.75193	6.47E-63	13	12.3624
Q96J02	903	102802.1	6.23	E3 ubiquiti	ITCH	ITCH	E3 ubiquiti	21	1241.695	53.2096	1.22E-68	17	24.03101
Q9NPH2	558	61067.3	5.51	Inositol-3-	ISYNA1	ISYNA1	Inositol-3-	6	262.8856	49.2426	1.68E-53	6	13.08244
Q9ULR0	285	32992	4.84	Pre-mRNA	ISY1	ISY1	Pre-mRNA	3	136.9639	44.12385	1.07E-36	3	12.98246
P53990	364	39750.1	4.97	IST1 homo	IST1	IST1	IST1 homo	2	393.7335	48.41826	1.47E-50	2	5.21978
Q96AB3	205	22337	7.89	Isochorism	ISOC2	ISOC2	Isochorism	1	62.75459	44.5095	7.75E-38	1	7.317073
Q9H9L3	353	39153.7	10.65	Interferon-	ISG20L2	ISG20L2	Interferon-	1	41.93483	47.3849	5.36E-47	1	3.399433
P05161	165	17887.4	7.61	Ubiquitin-	ISG15	ISG15	Ubiquitin-	2	117.7785	47.24629	1.57E-46	2	11.51515
Q9Y4H2	1338	137332.6	8.76	Insulin rec	IRS2	IRS2	Insulin rec	6	79.57615	51.10724	1.92E-60	6	6.726457
P35568	1242	131589.8	8.66	Insulin rec	IRS1	IRS1	Insulin rec	3	146.6036	47.25212	1.5E-46	3	3.542673
Q00978	393	43696	5.52	Interferon	IRF9	IRF9	Interferon	2	106.77	40.09909	4.59E-26	2	5.597964
Q9H1B7	796	82658.1	8.3	Probable E	IRF2BPL	IRF2BPL	Probable E	2	378.1882	40.45746	6.45E-27	2	3.266332
Q8IU81	584	61687.3	8.24	Interferon	IRF2BP1	IRF2BP1	Interferon	1	49.56564	39.99074	8.25E-26	1	2.568493
Q5JU85	1488	162782.3	8.71	IQ motif ar	IQSEC2	IQSEC2	IQ motif ar	6	113.3521	49.05687	7.83E-53	5	5.510753
Q6DN90	963	108313.3	6.93	IQ motif ar	IQSEC1	IQSEC1	IQ motif ar	5	188.3082	46.40271	9.65E-44	5	6.853583
Q13576	1575	180576.3	5.33	Ras GTPas	IQGAP2	IQGAP2	Ras GTPas	12	428.7258	55.10799	2.27E-76	12	9.52381
P46940	1657	189250.4	6.43	Ras GTPas	IQGAP1	IQGAP1	Ras GTPas	39	1147.344	52.39944	1.96E-65	36	24.62281
Q96P70	1041	115961.8	4.44	Importin-9	IPO9	IPO9	Importin-9	6	210.5125	50.32522	1.73E-57	6	7.012488
O15397	1037	119937	4.8	Importin-8	IPO8	IPO8	Importin-8	1	131.2244	35.44827	1.09E-16	1	0.96432
O95373	1038	119515.5	4.44	Importin-7	IPO7	IPO7	Importin-7	12	456.2155	50.72691	5.36E-59	11	13.96917
O00410	1097	123628.9	4.57	Importin-5	IPO5	IPO5	Importin-5	12	98.93573	49.14445	3.8E-53	12	15.13218
Q8TEX9	1081	118713.8	4.6	Importin-4	IPO4	IPO4	Importin-4	6	185.483	53.2305	1.02E-68	6	6.845513
Q9NVH2	962	106832.9	8.09	Integrator	INTS7	INTS7	Integrator	1	55.62387	52.94503	1.41E-67	1	1.351351
Q9UL03	887	100389.3	8.85	Integrator	INTS6	INTS6	Integrator	3	45.47612	44.33646	2.53E-37	3	3.494927
Q96HW7	963	108169.8	6.4	Integrator	INTS4	INTS4	Integrator	2	60.87997	42.50092	3.95E-32	2	4.569055
Q68E01	1043	118068.5	5.57	Integrator	INTS3	INTS3	Integrator	3	81.62753	37.26011	5.73E-20	3	3.068073
Q9H0H0	1204	134321.9	5.94	Integrator	INTS2	INTS2	Integrator	2	102.313	43.32066	2.18E-34	2	2.159468
Q96SY0	518	57470.3	4.76	Integrator	INTS14	INTS14	Integrator	2	64.21584	42.96413	2.15E-33	2	4.247104
Q9NVM9	706	80224.4	6.69	Integrator	INTS13	INTS13	Integrator	5	149.5989	43.52802	5.66E-35	5	7.507082
Q96CB8	462	48807.1	10.43	Integrator	INTS12	INTS12	Integrator	1	66.88741	51.08342	2.36E-60	1	3.030303
Q5TA45	600	67662.1	8.14	Integrator	INTS11	INTS11	Integrator	4	248.0727	50.04844	1.86E-56	4	7.166667
Q9NVR2	710	82235.3	7.48	Integrator	INTS10	INTS10	Integrator	5	204.1794	44.44223	1.23E-37	5	10.84507
Q8N201	2190	244294.6	6.04	Integrator	INTS1	INTS1	Integrator	7	131.2451	45.27541	3.64E-40	7	3.69863
O15357	1258	138597.5	6.49	Phosphatic	INPPL1	INPPL1	Phosphatic	2	139.4513	26.54439	2.76E-07	2	2.782194
Q9BT40	448	51089.8	6.51	Inositol po	INPP5K	INPP5K	Inositol po	6	219.0345	48.3686	2.19E-50	6	14.95536
Q9Y2H2	1132	128406	7.01	Phosphatic	INPP5F	INPP5F	Phosphatic	7	218.6374	49.18426	2.74E-53	7	6.272085
Q8NBZ0	244	26477.7	8.62	INO80 con	INO80E	INO80E	INO80 con	1	143.4322	45.25555	4.2E-40	1	6.967213
Q6PI98	192	20642.3	10.68	INO80 con	INO80C	INO80C	INO80 con	3	138.948	48.38015	1.99E-50	3	13.02083
Q9C086	356	38636.8	10.26	INO80 con	INO80B	INO80B	INO80 con	4	271.076	49.83194	1.18E-55	4	15.73034
Q9ULG1	1556	176751.7	10.05	Chromatin	INO80	INO80	Chromatin	14	245.335	54.19769	1.29E-72	14	10.98972
Q8WYH8	240	27750.3	7.69	Inhibitor o	ING5	ING5	Inhibitor o	4	704.3307	47.59946	9.99E-48	3	15.41667
Q9UNL4	249	28530.2	7.71	Inhibitor o	ING4	ING4	Inhibitor o	3	84.13307	45.44165	1.11E-40	3	10.44177
Q27J81	1249	135622.7	5.03	Inverted fc	INF2	INF2	Inverted fc	5	98.84682	40.13696	3.74E-26	5	5.204163
Q9NQS7	918	105427.9	10.1	Inner centr	INCENP	INCENP	Inner centr	11	927.3812	53.60894	3.24E-70	11	15.35948
P12268	514	55804.5	6.9	Inosine-5-	IMPDH2	IMPDH2	Inosine-5'-	37	88681.6	50.47297	4.88E-58	35	64.59144
P20839	514	55405.4	6.9	Inosine-5-	IMPDH1	IMPDH1	Inosine-5'-	21	10240.02	50.6978	6.9E-59	21	45.71984
Q9P2X3	320	36476.1	4.6	Protein IM	IMPACT	IMPACT	Protein IM	4	210.1656	45.45292	1.02E-40	4	12.8125
P29218	277	30188.6	4.91	Inositol mc	IMPA1	IMPA1	Inositol mc	1	94.80035	46.48323	5.28E-44	1	3.249097
Q96G21	291	33756.3	9.9	U3 small n	IMP4	IMP4	U3 small n	4	101.6004	47.33553	7.87E-47	4	20.9622
Q9NV31	184	21849.9	9.95	U3 small n	IMP3	IMP3	U3 small n	5	304.2371	51.92279	1.43E-63	5	34.78261
Q16891	758	83677.1	6.43	MICOS cor	IMMT	IMMT	MICOS cor	25	1903.745	54.70807	1.06E-74	25	36.54354
A1L0T0	632	67867.2	8.22	2-hydroxy	ILVBL	ILVBL	2-hydroxy	5	217.8963	51.62377	2.01E-62	5	14.55696
Q9H0C8	392	42906.1	7.11	Integrin-lir	ILKAP	ILKAP	Integrin-lir	2	130.1279	47.59573	1.03E-47	2	6.377551
Q13418	452	51418.8	8.18	Integrin-lir	ILK	ILK	Integrin-lir	2	64.8505	51.94449	1.18E-63	2	5.088496
Q12906	894	95338	9.08	Interleukin	ILF3	ILF3	Interleukin	35	9516.51	51.11282	1.82E-60	30	42.17002
Q12905	390	43061.8	4.93	Interleukin	ILF2	ILF2	Interleukin	19	14961.88	51.58885	2.74E-62	19	58.20513
Q13478	541	62303.5	7.95	Interleukin	IL18R1	IL18R1	Interleukin	1	30.22295	24.7172	1.33E-06	1	1.478743
Q14116	193	22326	4.27	Interleukin	IL18	IL18	Interleukin	2	661.0465	51.66612	1.38E-62	2	8.80829
Q9Y6K9	419	48197.3	5.46	NF-kappa-	IKBKG	IKBKG	NF-kappa-	1	109.557	31.0094	1.25E-10	1	3.818616
Q70UQ0	350	39308.6	9.89	Inhibitor o	IKBIP	IKBIP	Inhibitor o	3	97.31969	43.65167	2.51E-35	3	11.71429
Q13123	557	65601.7	6.57	Protein Re	IK	IK	Protein Re	12	1106.158	50.019	2.39E-56	12	24.41652
B9A064	214	23063.1	9.05	Immunogl	IGLL5	IGLL5;IGLC	Immunogl	2	130.9213	45.5722	4.32E-41	1	14.01869
P06312	121	13379.9	4.86	Immunogl	IGKV4-1	IGKV4-1	Immunogl	1	38.61598	39.12124	7.83E-24	1	7.438017
A0A075B6	116	12783.3	4.88	Probable r	IGKV3-7	IGKV3-7;IG	Probable r	1	1367.682	43.81984	8.24E-36	1	7.758621
P01619	116	12557	4.59	Immunogl	IGKV3-20	IGKV3-20	Immunogl	2	125.686	43.83877	7.26E-36	2	21.55172
P01834	107	11765	6.5	Immunogl	IGKC	IGKC;	Immunogl	2	50.72147	22.6286	4.11E-06	2	28.97196
A0A0B4J1Y	117	12839.5	8.45	Immunogl	IGHV3-21	IGHV3-21;	Immunogl	1	160.1296	48.62867	2.66E-51	1	9.401709
A0A0C4DH	117	12820.4	9.07	Immunogl	IGHV1-18	IGHV1-18;	Immunogl	1	109.59	36.7425	5.54E-19	1	9.401709

P38935	993	109148.1	9.33	DNA-bind	IGHMBP2	IGHMBP2	DNA-bind	17	687.7677	55.65625	1.26E-78	17	19.03323
P01871	474	51923.1	6.06	Immunogl	IGHM	IGHM;	Immunogl	3	65.62498	40.36147	1.1E-26	3	9.07173
P01859	395	43805.5	6.5	Immunogl	IGHG2	IGHG2;	Immunogl	6	739.238	47.80043	2.05E-48	2	22.78481
P01857	399	43911.6	6.96	Immunogl	IGHG1	IGHG1;	Immunogl	6	935.2232	52.51994	6.56E-66	4	24.31078
P01876	398	42848.1	5.37	Immunogl	IGHA1	IGHA1	Immunogl	2	193.3481	43.49357	7.09E-35	2	6.78392
P11717	2491	274372.4	5.75	Cation-inc	IGF2R	IGF2R	Cation-inc	8	131.7292	50.00548	2.69E-56	8	3.452429
O00425	579	63704.6	9.32	Insulin-like	IGF2BP3	IGF2BP3	Insulin-like	20	4751.84	53.11444	2.97E-68	17	39.37824
Q9Y6M1	599	66121	8.82	Insulin-like	IGF2BP2	IGF2BP2	Insulin-like	16	1657.548	51.83719	3.06E-63	16	31.2187
P78318	339	39221.6	5.06	Immunogl	IGBP1	IGBP1	Immunogl	2	115.5122	38.71233	6.11E-23	2	7.079646
Q9NQC8	304	34285.4	4.13	Intraflagell	IFT46	IFT46	Intraflagell	1	62.83827	47.7287	3.61E-48	1	7.236842
Q9BW83	186	20480.3	5.07	Intraflagell	IFT27	IFT27	Intraflagell	1	120.5628	47.87035	1.18E-48	1	8.602151
Q12894	442	48047.2	6.83	Interferon-	IFRD2	IFRD2	Interferon-	3	122.5001	45.43533	1.16E-40	3	7.692308
O00458	451	50268	7.2	Interferon-	IFRD1	IFRD1	Interferon-	2	45.40454	38.06637	1.4E-21	2	5.321508
P13164	125	13964.4	8.09	Interferon-	IFITM1	IFITM1;IFIT	Interferon-	1	195.8917	50.88479	1.35E-59	1	12.8
Q13325	482	55846.2	7.44	Interferon-	IFIT5	IFIT5	Interferon-	3	62.85556	51.25799	5.1E-61	3	6.224066
P51553	393	42794	8.66	Isocitrate c	IDH3G	IDH3G	Isocitrate c	3	230.129	54.85953	2.49E-75	3	8.396947
O43837	385	42183.4	8.66	Isocitrate c	IDH3B	IDH3B	Isocitrate c	7	396.1679	50.35796	1.31E-57	7	23.11688
P50213	366	39591.4	6.93	Isocitrate c	IDH3A	IDH3A	Isocitrate c	7	472.4615	46.49171	4.96E-44	7	21.85792
P48735	452	50908.9	8.95	Isocitrate c	IDH2	IDH2	Isocitrate c	7	277.3018	51.41108	1.32E-61	7	19.9115
O75874	414	46659	7	Isocitrate c	IDH1	IDH1	Isocitrate c	10	815.9913	53.09554	3.53E-68	9	29.95169
P14735	1019	117967.5	6.59	Insulin-deg	IDE	IDE	Insulin-deg	21	2087.099	54.97831	8.08E-76	21	21.8842
O60725	284	31937.8	8.05	Protein-S-	ICMT	ICMT	Protein-S-	2	115.7086	41.66633	6.31E-30	2	3.521127
P05362	532	57824.8	8.06	Intercellula	ICAM1	ICAM1	Intercellula	5	195.0469	47.54437	1.54E-47	5	13.15789
Q9P2D0	1353	150526.6	7.75	Inhibitor o	IBTK	IBTK	Inhibitor o	8	363.8615	48.9856	1.41E-52	8	6.134516
Q5T440	356	38154.6	10.26	Putative tr	IBA57	IBA57	Putative tr	3	91.93771	38.84236	3.2E-23	3	10.11236
Q9NSE4	1012	113790.6	7.2	Isoleucine-	IARS2	IARS2	Isoleucine-	17	744.7754	52.24072	8.14E-65	17	21.14625
P41252	1262	144496.9	6.03	Isoleucine-	IARS1	IARS1	Isoleucine-	27	1501.417	52.71363	1.15E-66	27	24.24723
Q9Y4L1	999	111334.5	4.88	Hypoxia up	HYOU1	HYOU1	Hypoxia up	19	383.7574	52.61837	2.71E-66	19	23.12312
Q7Z6Z7	4374	481885.7	4.87	E3 ubiquiti	HUWE1	HUWE1	E3 ubiquiti	8	146.278	53.20768	1.24E-68	8	2.080476
O43464	458	48840.4	10.48	Serine prot	HTRA2	HTRA2	Serine prot	8	780.4425	53.52047	7.17E-70	7	20.74236
O43719	755	85852	4.02	17S U2 SnI	HTATSF1	HTATSF1	17S U2 SnI	5	201.0672	53.04381	5.69E-68	5	6.887417
Q9BUP3	242	27048.8	8.61	Oxidoredu	HTATIP2	HTATIP2	Oxidoredu	1	33.82535	39.78181	2.52E-25	1	3.305785
Q92598	858	96864.3	5.04	Heat shock	HSPH1	HSPH1	Heat shock	19	689.3336	51.18416	9.75E-61	19	27.73893
P98160	4391	468826.5	6.49	Basement	HSPG2	HSPG2	Basement	18	295.6975	50.8453	1.9E-59	18	5.351856
P61604	102	10931.6	9.49	10 kDa hea	HSPE1	HSPE1	10 kDa hea	2	69.50387	42.94793	2.38E-33	2	19.60784
P10809	573	61054.2	5.55	60 kDa hea	HSPD1	HSPD1	60 kDa hea	25	5882.313	52.02548	5.69E-64	25	60.20942
P04792	205	22782.3	6.36	Heat shock	HSPB1	HSPB1	Heat shock	7	1278.645	46.03201	1.52E-42	7	36.58537
P38646	679	73679.9	6.01	Stress-70 p	HSPA9	HSPA9	Stress-70 p	32	19594.38	52.93802	1.51E-67	32	50.66274
P11142	646	70897.6	5.16	Heat shock	HSPA8	HSPA8	Heat shock	21	9935.201	50.02409	2.29E-56	21	34.36533
P11021	654	72332.4	4.8	Endoplasm	HSPA5	HSPA5	Endoplasm	33	13123.25	51.52011	5E-62	33	45.25994
O95757	839	94511.7	5.65	Heat shock	HSPA4L	HSPA4L	Heat shock	12	733.468	54.37268	2.67E-73	9	18.71275
P34932	840	94330.2	4.82	Heat shock	HSPA4	HSPA4	Heat shock	16	598.107	52.99771	8.69E-68	16	23.92857
P54652	639	70020.4	5.42	Heat shock	HSPA2	HSPA2	Heat shock	9	4430.87	49.83538	1.15E-55	1	15.17997
P0DMV8	641	70051.6	5.31	Heat shock	HSPA1A	HSPA1A;H	Heat shock	25	17933.08	52.68227	1.54E-66	12	44.14977
P48723	471	51927.1	5.51	Heat shock	HSPA13	HSPA13	Heat shock	1	77.89661	39.09874	8.78E-24	1	2.123142
O43301	675	74977.7	6.76	Heat shock	HSPA12A	HSPA12A	Heat shock	5	211.7623	51.34027	2.46E-61	4	9.333333
P14625	803	92468.1	4.48	Endoplasm	HSP90B1	HSP90B1	Endoplasm	23	4158.387	53.61846	2.98E-70	19	29.88792
Q58FF8	381	44348.6	4.49	Putative he	HSP90AB2	HSP90AB2	Putative he	1	1464.785	42.89556	3.32E-33	1	3.937008
P08238	724	83263.5	4.68	Heat shock	HSP90AB1	HSP90AB1	Heat shock	33	16346.51	53.47905	1.04E-69	9	42.54144
P07900	732	84659	4.66	Heat shock	HSP90AA1	HSP90AA1	Heat shock	17	3313.074	50.64056	1.13E-58	11	23.08743
Q6YN16	418	45394.3	8.18	Hydroxyste	HSDL2	HSDL2	Hydroxyste	6	398.6266	41.07225	2.02E-28	6	13.8756
Q3SXM5	330	37001.4	8.9	Inactive hy	HSDL1	HSDL1	Inactive hy	7	228.2076	47.91378	8.36E-49	7	22.12121
Q92506	261	26973.6	6.5	(3R)-3-hyd	HSD17B8	HSD17B8	(3R)-3-hyd	5	147.4401	45.70589	1.64E-41	5	26.05364
P56937	341	38205.8	8.21	3-keto-ste	HSD17B7	HSD17B7	3-keto-ste	2	210.0574	44.05997	1.65E-36	2	7.917889
P51659	736	79685.7	9.21	Peroxisom	HSD17B4	HSD17B4	Peroxisom	10	451.1613	52.19589	1.22E-64	10	17.25543
Q53GQ0	312	34323.9	9.79	Very-long-	HSD17B12	HSD17B12	Very-long-	6	504.9142	49.81139	1.41E-55	6	24.35897
Q8NBQ5	300	32963.6	9.7	Estradiol 1	HSD17B11	HSD17B11	Estradiol 1	4	158.3024	46.85785	3.11E-45	4	15.33333
Q99714	261	26922.9	7.94	3-hydroxy	HSD17B10	HSD17B10	3-hydroxy	6	606.8058	49.40788	4.23E-54	6	29.88506
Q86YZ3	2850	282390.2	10.24	Hornerin	HRNR	HRNR	Hornerin	4	60.27484	46.8214	4.11E-45	4	6.561404
P01112	189	21298	4.94	GTPase HF	HRAS	HRAS	GTPase HF	1	340.4702	40.17106	3.11E-26	1	6.349206
P02790	462	51676	7.02	Hemopexi	HPX	HPX	Hemopexi	2	196.7822	44.29448	3.37E-37	2	6.926407
Q9Y251	543	61148.2	9.71	Heparanas	HPSE	HPSE	Heparanas	5	168.0611	50.29436	2.25E-57	5	13.44383
Q86YV9	775	82973.8	6.22	BLOC-2 cc	HPS6	HPS6	BLOC-2 cc	1	47.9319	37.11461	1.09E-19	1	2.064516
Q9UPZ3	1129	127447.8	5.23	BLOC-2 cc	HPS5	HPS5	BLOC-2 cc	1	38.55281	40.139	3.7E-26	1	1.151461
P00492	218	24579.2	6.67	Hypoxanth	HPRT1	HPRT1	Hypoxanth	6	230.5274	45.74174	1.27E-41	6	31.65138
Q9NWY4	346	39435.8	6.79	Histone PA	HPF1	HPF1	Histone PA	2	65.49858	44.96603	3.25E-39	2	7.803468
P37235	193	22313	4.99	Hippocalci	HPCAL1	HPCAL1	Hippocalci	5	243.9162	50.04588	1.9E-56	2	27.97927
Q5SSJ5	553	61206.5	10.36	Heterochro	HP1BP3	HP1BP3	Heterochro	29	9553.595	54.2054	1.21E-72	29	45.38879
P00738	406	45205.1	6.56	Haptoglob	HP	HP	Haptoglob	7	344.7862	55.65365	1.27E-78	3	21.18227
P13378	290	31910.4	8.66	Homeobox	HOXD8	HOXD8;HC	Homeobox	1	164.831	45.63197	2.81E-41	1	3.448276
P31271	388	39726.3	9.45	Homeobox	HOXA13	HOXA13;H	Homeobox	2	109.1943	44.80903	9.76E-39	1	4.896907
Q86YM7	354	40276.5	5.14	Homer prc	HOMER1	HOMER1	Homer prc	2	162.2489	22.90537	3.63E-06	2	4.80226
Q1KMD3	747	85104.2	4.55	Heterogen	HNRNPUL	HNRNPUL	Heterogen	26	12573.56	51.12652	1.62E-60	26	32.53012
Q9BUJ2	856	95738	6.9	Heterogen	HNRNPUL	HNRNPUL	Heterogen	24	5715.963	52.50932	7.22E-66	24	29.6729
Q00839	825	90584.1	5.8	Heterogen	HNRNPU	HNRNPU	Heterogen	36	35966.38	54.32463	4.12E-73	36	45.81818
O43390	633	70942.8	8.32	Heterogen	HNRNPR	HNRNPR	Heterogen	27	25135.58	49.96731	3.74E-56	21	37.1248
P52272	730	77515.3	9.1	Heterogen	HNRNPM	HNRNPM	Heterogen	35	22838.88	53.12124	2.82E-68	35	53.0137
Q8WVV9	542	60082.7	7.79	Heterogen	HNRNPLL	HNRNPLL	Heterogen	21	1382.11	50.30471	2.06E-57	21	48.15498
P14866	589	64132.5	8.32	Heterogen	HNRNPL	HNRNPL	Heterogen	21	19424.08	50.14417	8.18E-57	21	48.55688
P61978	463	50975.8	5.18	Heterogen	HNRNPK	HNRNPK	Heterogen	21	21390.19	52.64424	2.14E-66	21	49.02808
P31942	346	36926.4	6.87	Heterogen	HNRNPH3	HNRNPH3	Heterogen	9	10899.76	50.77685	3.47E-59	9	37.57225
P55795	449	49263.3	6.25	Heterogen	HNRNPH2	HNRNPH2	Heterogen	5	1638.394	48.68723	1.65E-51	5	14.2539
P31943	449	49229.2	6.25	Heterogen	HNRNPH1	HNRNPH1	Heterogen	14	15082.81	54.3178	4.37E-73	9	36.52561
P52597	415	45671.6	5.31	Heterogen	HNRNPF	HNRNPF	Heterogen	9	6389.469	49.72676	2.87E-55	9	28.43373
O14979	420	46437.3	9.98	Heterogeneous nuclea	HNRNPDL	HNRNPDL	Heterogen	9	1480.125	48.71792	1.28E-51	9	18.33333
Q14103	355	38434.1	8.01	Heterogen	HNRNPD	HNRNPD	Heterogen	12	7969.021	48.91129	2.6E-52	12	33.80282
P07910	306	33669.7	4.69	Heterogen	HNRNPC	HNRNPC	Heterogen	17	18254.93	51.55021	3.84E-62	11	41.17647
Q99729	332	36224.8	8.48	Heterogen	HNRNPAB	HNRNPAB	Heterogen	7	2999.22	50.04141	1.98E-56	6	18.07229
P51991	378	39595	9.31	Heterogen	HNRNPA3	HNRNPA3	Heterogen	18	17028.58	53.1394	2.38E-68	18	42.85714
P22626	353	37429.7	9.26	Heterogen	HNRNPA2	HNRNPA2	Heterogen	20	9838.852	54.27943	6.27E-73	20	56.09065
P09651	372	38746.7	9.46	Heterogen	HNRNPA1	HNRNPA1	Heterogen	17	5412.561	50.78318	3.28E-59	1	48.3871
Q13151	305	30840.6	9.66	Heterogen	HNRNPA0	HNRNPA0	Heterogen	10	1851.75	51.56833	3.28E-62	10	39.67213
P30519	316	36032.6	5.09	Heme oxyg	HMOX2	HMOX2	Heme oxyg	5	193.5451	50.66162	9.41E-59	5	22.1519

P09601	288	32818.3	8.68	Heme oxyg	HMOX1	HMOX1	Heme oxyg	1	118.1963	40.92267	4.75E-28	1	4.513889
O00479	90	9538.8	11.26	High mobi	HMGN4	HMGN4	High mobi	3	467.4871	46.99249	1.11E-45	3	24.44444
P05204	90	9392.6	10.79	Non-histo	HMGN2	HMGN2	Non-histo	4	85.99114	48.47443	9.32E-51	2	30
P05114	100	10658.8	10.4	Non-histo	HMGN1	HMGN1	Non-histo	5	423.6864	50.44106	6.41E-58	5	48
P35914	325	34359.8	8.71	Hydroxym	HMGCL	HMGCL	Hydroxym	4	102.2334	48.36188	2.31E-50	4	16.30769
P26583	209	24033.6	8.04	High mobi	HMGB2	HMGB2	High mobi	2	117.6682	40.57167	3.42E-27	2	10.04785
P09429	215	24893.6	5.42	High mobi	HMGB1	HMGB1	High mobi	3	631.7927	49.87345	8.3E-56	1	20
P52926	109	11832	11.36	High mobi	HMGA2	HMGA2	High mobi	5	5262.84	50.70259	6.62E-59	5	41.2844
P17096	107	11676	11.09	High mobi	HMGA1	HMGA1	High mobi	9	30689.66	50.97985	5.85E-60	9	53.27103
Q96FZ2	354	40574.6	8.25	Abasic site	HMCES	HMCES	Abasic site	3	77.94804	54.84858	2.73E-75	3	11.86441
Q8TCT9	377	41487.9	6.4	Minor histo	HM13	HM13	Minor histo	2	447.4088	47.62161	8.4E-48	2	5.570292
Q14527	1009	113927.7	8.84	Helicase-li	HLTF	HLTF	Helicase-li	22	831.964	53.84137	3.69E-71	22	26.56095
P01893	362	40891.6	6.25	Putative H	HLA-H	HLA-H	Putative H	1	147.3205	48.2771	4.59E-50	1	4.696133
P13747	358	40057.3	5.69	HLA class I	HLA-E	HLA-E	HLA class I	3	125.123	42.74629	8.53E-33	3	9.217877
P79483	266	29961.9	8.7	HLA class I	HLA-DRB3	HLA-DRB3	HLA class I	5	225.1938	48.44399	1.19E-50	3	23.68421
P01911	266	29966	7.83	HLA class I	HLA-DRB1	HLA-DRB1	HLA class I	1	214.8488	42.11514	4.24E-31		3.383459
P01903	254	28620.7	4.64	HLA class I	HLA-DRA	HLA-DRA	HLA class I	2	99.11435	48.43834	1.25E-50	2	11.02362
P10321	366	40648.2	5.9	HLA class I	HLA-C	HLA-C	HLA class I	6	1360.898	50.8742	1.49E-59	4	25.68306
P01889	362	40459.7	5.65	HLA class I	HLA-B	HLA-B	HLA class I	8	3367.725	56.56462	1.91E-82	1	26.79558
P04439	365	40840.4	5.85	HLA class I	HLA-A	HLA-A	HLA class I	4	366.7802	49.46063	2.71E-54	4	13.15068
Q2TB90	917	102543.9	7.12	Hexokinas	HKDC1	HKDC1	Hexokinas	3	81.8322	48.62105	2.83E-51	3	4.143948
P52789	917	102379.1	5.93	Hexokinas	HK2	HK2	Hexokinas	13	466.733	52.57998	3.85E-66	13	17.12105
P19367	917	102485.1	6.78	Hexokinas	HK1	HK1	Hexokinas	15	329.6365	51.97254	9.18E-64	13	18.75682
O75146	1068	119387.2	6.66	Huntingtin	HIP1R	HIP1R	Huntingtin	10	290.7959	49.06325	7.43E-53	10	10.95506
Q9BX68	163	17161.6	9.61	Adenosine	HINT2	HINT2	Adenosine	1	58.34604	35.79807	2.77E-17	1	9.815951
Q8IV36	788	88743.7	5.96	Protein HI[	HID1	HID1	Protein HI[	2	38.7284	40.88217	5.98E-28	2	4.060914
P31937	336	35328.5	8.26	3-hydroxy	HIBADH	HIBADH	3-hydroxy	1	67.06142	26.95952	1.73E-07	1	3.869048
O14964	777	86191.5	6.07	Hepatocyt	HGS	HGS	Hepatocyt	3	155.6269	44.31527	2.93E-37	3	4.118404
P14210	728	83133.1	7.92	Hepatocyt	HGF	HGF	Hepatocyt	1	183.2761	39.03962	1.19E-23	1	1.098901
Q96MH2	286	32418.3	6.52	Protein HE	HEXIM2	HEXIM2	Protein HE	7	530.6837	52.24741	7.7E-65	7	25.87413
O94992	359	40623.2	4.54	Protein HE	HEXIM1	HEXIM1	Protein HE	8	831.5353	50.20258	4.97E-57	7	20.89136
P07686	556	63136.8	6.75	Beta-hexo	HEXB	HEXB	Beta-hexo	3	126.9045	50.13321	9E-57	3	5.935252
Q9UII4	1024	116851.4	7.7	E3 ISG15--	HERC5	HERC5	E3 ISG15--	18	515.3428	51.6022	2.43E-62	18	22.94922
Q5GLZ8	1057	118562	6.11	Probable E	HERC4	HERC4	Probable E	3	59.22413	44.53293	6.6E-38	3	3.59508
Q9BYK8	2896	322313.1	8.02	3-5 exorib	HELZ2	HELZ2	3'-5' exoril	87	2451.753	54.83276	3.14E-75	87	36.22238
Q9NRZ9	838	97073.1	8.05	Lymphoid-	HELLS	HELLS	Lymphoid-	2	112.3159	35.48256	9.58E-17	2	3.221957
Q76N89	1606	179551.9	5.21	E3 ubiquiti	HECW1	HECW1	E3 ubiquiti	3	154.0561	43.15571	6.31E-34	3	2.241594
Q9UBI9	543	58836.2	8.63	Headcase	HECA	HECA	Headcase	3	142.3661	52.69417	1.38E-66	3	7.550645
Q6AI08	1181	128779.8	7.04	HEAT repe	HEATR6	HEATR6	HEAT repe	1	66.45676	47.46679	2.82E-47	1	1.016088
Q7Z4Q2	680	74581.8	4.75	HEAT repe	HEATR3	HEATR3	HEAT repe	9	339.8207	50.88328	1.37E-59	9	16.17647
Q9H583	2144	242367.7	6.51	HEAT repe	HEATR1	HEATR1	HEAT repe	12	392.0895	51.11625	1.77E-60	12	6.529851
Q00341	1268	141438.5	6.86	Vigilin	HDLBP	HDLBP	Vigilin	26	569.922	54.18168	1.5E-72	26	26.57729
Q9BXW7	423	46321.1	8.22	Haloacid d	HDHD5	HDHD5	Haloacid d	6	297.1971	50.16857	6.64E-57	6	17.49409
Q7Z4V5	671	74316	7.62	Hepatoma	HDGFL2	HDGFL2	Hepatoma	18	8820.851	51.70113	1.02E-62	16	30.99851
P51858	240	26788.1	4.39	Hepatoma	HDGF	HDGF	Hepatoma	1	195.5179	21.98602	5.41E-06	1	3.75
Q9UBN7	1215	131418.2	4.98	Histone de	HDAC6	HDAC6	Histone de	2	53.32006	32.25406	4.78E-12	2	2.222222
O15379	428	48847.4	4.79	Histone de	HDAC3	HDAC3	Histone de	1	163.0811	38.75273	5E-23	1	2.102804
Q92769	488	55363.9	5.75	Histone de	HDAC2	HDAC2	Histone de	6	663.6488	48.12666	1.53E-49	6	17.21311
Q13547	482	55102.6	5.16	Histone de	HDAC1	HDAC1	Histone de	9	3063.281	54.25165	7.96E-73	5	21.9917
Q9Y5Z7	792	86778.4	8.6	Host cell fa	HCFC2	HCFC2	Host cell fa	1	53.48143	42.33898	1.08E-31	1	1.641414
P51610	2035	208731	7.48	Host cell fa	HCFC1	HCFC1	Host cell fa	28	1801.823	55.28854	4.02E-77	27	17.14988
P53701	268	30601.3	6.66	Holocytoch	HCCS	HCCS	Holocytoch	3	73.60015	44.37546	1.94E-37	3	13.43284
Q9Y450	684	75472.4	6.57	HBS1-like	HBS1L	HBS1L	HBS1-like	16	627.5126	52.74944	8.39E-67	16	30.84795
O60381	514	57644.8	6.13	HMG box-	HBP1	HBP1	HMG box-	1	22.84005	36.58963	1.07E-18	1	2.918288
P02100	147	16202.7	9.1	Hemoglob	HBE1	HBE1	Hemoglob	2	412.0102	47.73839	3.35E-48	1	17.68707
O94927	633	71681.9	8.64	HAUS aug	HAUS5	HAUS5	HAUS aug	1	224.6939	35.56027	7.08E-17	1	1.105845
O14929	419	49540.2	5.41	Histone ac	HAT1	HAT1	Histone ac	5	163.8244	52.52816	6.11E-66	5	15.0358
P49590	506	56887.9	8.35	Histidine--	HARS2	HARS2	Histidine--	7	414.1662	48.39742	1.73E-50	4	14.42688
P12081	509	57410	5.56	Histidine--	HARS1	HARS1	Histidine--	2	198.7263	36.85334	3.43E-19	2	4.125737
Q16775	308	33805.6	8.23	Hydroxyac	HAGH	HAGH	Hydroxyac	6	820.2952	50.73515	4.98E-59	6	25
P55084	474	51294	9.94	Trifunction	HADHB	HADHB	Trifunction	10	1067.721	53.35272	3.35E-69	10	25.94937
P40939	763	82999	9.52	Trifunction	HADHA	HADHA	Trifunction	21	856.017	53.83762	3.82E-71	21	35.25557
Q9UJ83	578	63728.1	7.39	2-hydroxy	HACL1	HACL1	2-hydroxy	4	147.7742	47.36173	6.41E-47	4	8.650519
Q9P035	362	43159.2	9.25	Very-long-	HACD3	HACD3	Very-long	5	344.0327	50.63211	1.22E-58	5	18.78453
Q5JVS0	413	45785.1	7.58	Intracellula	HABP4	HABP4	Intracellula	6	292.0994	48.97333	1.56E-52	6	21.79177
Q14520	560	62671.3	6.52	Hyalurona	HABP2	HABP2	Hyalurona	1	116.1192	44.70638	1.99E-38	1	1.785714
P62805	103	11367.3	11.91	Histone H4	H4C1	H4C1	Histone H4	17	144871	50.39089	9.88E-58	17	70.87379
Q71DI3	136	15387.9	11.83	Histone H3	H3C15	H3C15	Histone H3	1	4362.058	45.44119	1.11E-40	1	23.52941
P68431	136	15403.9	11.71	Histone H3	H3C1	H3C1	Histone H3	12	2552.771	50.52817	3.03E-58	1	67.64706
Q5TEC6	136	15430	11.83	Histone H3	H3-7	H3-7	Histone H3	2	311.2761	43.68119	2.06E-35	2	22.05882
P84243	136	15327.7	11.83	Histone H3	H3-3A	H3-3A	Histone H3	2	644.5729	45.23373	4.9E-40	2	24.26471
P58876	126	13936.1	11	Histone H2	H2BC5	H2BC5	Histone H2	7	41247.7	49.55894	1.19E-54	1	27.77778
Q8N257	126	13908	11	Histone H2	H2BC26	H2BC26	Histone H2	1	645.6783	43.74318	1.37E-35	1	8.730159
Q6DN03	193	21471.8	11.36	Putative hi	H2BC20P	H2BC20P;I	Putative hi	1	4015.002	34.86827	9.78E-16	1	7.772021
P23527	126	13906	11	Histone H2	H2BC17	H2BC17	Histone H2	1	5731.371	51.50714	5.61E-62		8.730159
Q99879	126	13989.2	11	Histone H2	H2BC14	H2BC14	Histone H2	1	73.65549	42.36637	9.1E-32	1	8.730159
P06899	126	13904.1	11	Histone H2	H2BC11	H2BC11	Histone H2	16	106251.5	51.92444	1.41E-63		72.22222
P0C0S5	128	13552.6	11.29	Histone H2	H2AZ1	H2AZ1;H2	Histone H2	4	38665.09	50.05135	1.82E-56	4	28.125
P16104	143	15144.4	11.39	Histone H2	H2AX	H2AX	Histone H2	3	989.9423	47.85077	1.38E-48	1	23.07692
P04908	130	14135.4	11.66	Histone H2	H2AC4	H2AC4;H2	Histone H2	9	35656.89	48.5015	7.48E-51	2	43.84615
P0C0S8	130	14091.4	11.55	Histone H2	H2AC11	H2AC11;H	Histone H2	2	257.1749	51.79396	4.47E-63		23.07692
Q8IZA3	346	35813.2	11.9	Histone H1	H1-8	H1-8	Histone H1	1	198.5197	38.91233	2.24E-23	1	3.179191
P22492	207	22018.8	12.28	Histone H1	H1-6	H1-6	Histone H1	3	370.4446	50.4677	5.09E-58	3	15.45894
P16401	226	22579.9	11.69	Histone H1	H1-5	H1-5	Histone H1	1	216863.4	21.34889	6.97E-06	1	5.309735
P10412	219	21865	11.8	Histone H1	H1-4	H1-4	Histone H1	1	121721.3	49.82661	1.24E-55	1	7.305936
P16403	213	21364.6	11.71	Histone H1	H1-2	H1-2	Histone H1	13	122403.6	51.4771	7.32E-62	2	44.60094
Q92522	213	22487	11.49	Histone H1	H1-10	H1-10	Histone H1	8	9850.375	55.42561	1.18E-77	8	32.86385
Q02539	215	21841.9	11.76	Histone H1	H1-1	H1-1	Histone H1	1	168.5097	40.45668	6.48E-27	1	4.651163
P07305	194	20862.8	11.6	Histone H1	H1-0	H1-0	Histone H1	11	28607.49	47.97857	4.99E-49	11	30.92784
Q9H116	711	80491.3	7.91	GDNF-ind	GZF1	GZF1	GDNF-ind	3	134.6661	44.53035	6.72E-38	3	4.360056
P13807	737	83784.8	6.1	Glycogen [	GYS1	GYS1	Glycogen [	10	663.2822	45.62294	3E-41	9	13.29715
P46976	350	39383.4	5.19	Glycogenir	GYG1	GYG1	Glycogenir	3	114.1509	51.51632	5.17E-62	3	10
Q8N442	669	74327.2	8.87	Translatior	GUF1	GUF1	Translatior	3	67.691	49.27648	1.27E-53	3	5.082212

Q8WW33	167	19266.3	6.5	Gametocyt	GTSF1	GTSF1	Gametocyt	2	56.71967	53.68533	1.6E-70	2	12.57485
O43824	516	56897	9.97	Putative G	GTPBP6	GTPBP6	Putative G	4	94.64047	45.34649	2.19E-40	4	9.108527
Q9BZE4	634	73963.9	10.07	GTP-bindi	GTPBP4	GTPBP4	GTP-bindi	23	5307.044	51.0017	4.84E-60	23	41.95584
Q9BX10	602	65768	8.13	GTP-bindi	GTPBP2	GTPBP2	GTP-bindi	7	232.3947	49.08222	6.35E-53	7	12.29236
A4D1E9	387	42932.6	9.55	GTP-bindi	GTPBP10	GTPBP10	GTP-bindi	7	250.6525	50.37315	1.15E-57	7	19.12145
O00178	669	72453.3	8.47	GTP-bindi	GTPBP1	GTPBP1	GTP-bindi	7	569.4724	49.91449	5.86E-56	7	12.2571
Q9Y5Q8	519	59570.3	6.9	General tra	GTF3C5	GTF3C5	General tra	4	233.008	53.29216	5.84E-69	4	7.321773
Q9UKN8	822	91981.6	6.64	General tra	GTF3C4	GTF3C4	General tra	15	397.9122	50.48717	4.32E-58	15	23.60097
Q9Y5Q9	886	101271.2	4.71	General tra	GTF3C3	GTF3C3	General tra	5	340.2272	44.08703	1.38E-36	5	6.659142
Q8WUA4	911	100678.6	7.33	General tra	GTF3C2	GTF3C2	General tra	7	396.4458	51.04398	3.33E-60	7	10.75741
Q12789	2109	238872.6	7.31	General tra	GTF3C1	GTF3C1	General tra	21	615.0098	53.03246	6.32E-68	21	10.66856
P78347	998	112415.5	6.29	General tra	GTF2I	GTF2I	General tra	12	266.8259	48.94932	1.9E-52	12	14.02806
Q92759	462	52185.9	9.38	General tra	GTF2H4	GTF2H4	General tra	6	231.1996	47.29919	1.04E-46	6	18.61472
Q13888	395	44418.6	6.75	General tra	GTF2H2	GTF2H2;G1	General tra	7	294.3062	45.78608	9.17E-42	7	19.74684
P32780	548	62031.1	8.98	General tra	GTF2H1	GTF2H1	General tra	5	145.8982	48.40741	1.6E-50	5	9.671533
P13984	249	28380.1	9.74	General tra	GTF2F2	GTF2F2	General tra	3	300.1596	50.8443	1.92E-59	3	11.64659
P35269	517	58240	7.69	General tra	GTF2F1	GTF2F1	General tra	3	104.1034	43.51017	6.35E-35	3	7.930368
P29084	291	33043.4	10.34	Transcripti	GTF2E2	GTF2E2	Transcripti	4	108.494	52.39028	2.12E-65	4	16.1512
Q00403	316	34832.5	8.47	Transcripti	GTF2B	GTF2B	Transcripti	3	67.52503	28.97014	8.92E-09	3	11.70886
P52657	109	12457	6.58	Transcripti	GTF2A2	GTF2A2	Transcripti	1	110.1133	39.6206	5.91E-25	1	8.256881
P09211	210	23355.6	5.3	Glutathion	GSTP1	GSTP1	Glutathion	4	2142.729	51.34858	2.29E-61	4	26.66667
P78417	241	27565.6	6.54	Glutathion	GSTO1	GSTO1	Glutathion	6	210.4003	51.58786	2.76E-62	6	24.89627
Q9Y2Q3	226	25496.6	8.69	Glutathion	GSTK1	GSTK1	Glutathion	5	457.3678	51.86736	2.32E-63	5	30.0885
P00390	522	56256.6	8.66	Glutathion	GSR	GSR	Glutathion	11	1876.266	50.33098	1.65E-57	11	34.67433
P15170	499	55755.6	5.34	Eukaryotic	GSPT1	GSPT1	Eukaryotic	8	101.9765	50.08286	1.39E-56	2	21.04208
P06396	782	85696.9	6.19	Gelsolin	GSN	GSN	Gelsolin	21	3409.129	51.82036	3.55E-63	21	37.59591
P57764	484	52800.6	4.74	Gasdermin	GSDMD	GSDMD	Gasdermin	6	370.3918	49.80779	1.45E-55	6	20.24793
Q9BQ67	446	49418.8	4.56	Glutamate	GRWD1	GRWD1	Glutamate	4	565.2694	49.52214	1.62E-54	4	12.55605
Q12849	480	53125.8	6.11	G-rich seq	GRSF1	GRSF1	G-rich seq	14	2625.95	57.05985	2.36E-84	14	40.20833
Q8TAA5	225	25431	7.83	GrpE prote	GRPEL2	GRPEL2	GrpE prote	2	126.8287	34.02872	1.91E-14	2	10.66667
Q9HAV7	217	24278.9	8.31	GrpE prote	GRPEL1	GRPEL1	GrpE prote	2	132.6205	37.60272	1.21E-20	2	7.834101
P28799	593	63543.8	6.82	Progranuli	GRN	GRN	Progranuli	20	9383.236	52.8183	4.53E-67	20	44.51939
P43250	576	65990.5	8.06	G protein-	GRK6	GRK6	G protein-	2	72.27097	29.38342	4.17E-09	1	5.034722
P32298	578	66582.7	7.76	G protein-	GRK4	GRK4	G protein-	1	26.35043	42.18675	2.74E-31	1	2.422145
Q6ISB3	625	71104.6	6.38	Grainyhead	GRHL2	GRHL2	Grainyhead	1	85.38919	23.63754	2.53E-06	1	2.4
Q14451	532	59680.5	8.58	Growth fac	GRB7	GRB7	Growth fac	5	240.7885	51.22698	6.68E-61	5	12.40602
P62993	217	25206.2	6.25	Growth fac	GRB2	GRB2	Growth fac	9	656.6196	48.98616	1.4E-52	9	43.31797
Q8IUY3	354	40249.1	8.53	GRAM dor	GRAMD2A	GRAMD2A	GRAM dor	1	204.8487	40.40806	8.48E-27	1	2.259887
Q8TED1	209	23880.8	9.92	Probable g	GPX8	GPX8	Probable g	2	230.4852	46.76947	6.08E-45	2	10.04785
P36969	197	22174.5	8.48	Phospholip	GPX4	GPX4	Phospholip	2	251.5125	50.34065	1.52E-57	2	12.69036
P07203	203	22087.9	6.51	Glutathion	GPX1	GPX1	Glutathion	10	1198.723	50.1744	6.32E-57	10	48.76847
Q8TD30	523	57903.1	7.77	Alanine am	GPT2	GPT2	Alanine am	2	219.4386	42.68074	1.29E-32	1	3.441683
Q86YR5	675	74509.5	6.51	G-protein-	GPSM1	GPSM1	G-protein-	1	36.28535	32.77038	1.07E-12	1	1.777778
Q13098	491	55536	6.73	COP9 sign	GPS1	GPS1	COP9 sign	4	207.5681	49.71855	3.07E-55	4	9.368635
Q9NQ84	441	48192.8	8.52	G-protein	GPRC5C	GPRC5C	G-protein	2	74.43385	20.57949	9.27E-06	2	4.535147
Q8NFJ5	357	40250.7	8.26	Retinoic ac	GPRC5A	GPRC5A	Retinoic ac	5	1683.579	49.30526	1E-53	5	13.16527
Q9NPR9	543	60632	8.9	Protein GP	GPR108	GPR108	Protein GP	1	36.9263	32.34922	3.65E-12	1	1.473297
Q9HCN4	374	41739.8	4.54	GPN-loop	GPN1	GPN1	GPN-loop	1	150.3245	52.40595	1.85E-65	1	4.278075
Q92917	476	52228.4	6.01	G-patch d	GPKOW	GPKOW	G-patch d	1	56.70293	44.4849	9.17E-38	1	1.890756
P06744	558	63146.7	8.55	Glucose-6	GPI	GPI	Glucose-6	7	165.7266	49.40345	4.39E-54	7	15.94982
Q9NQX3	736	79747.6	5.09	Gephyrin	GPHN	GPHN	Gephyrin	2	106.211	47.47326	2.68E-47	2	3.26087
P43304	727	80852	7.77	Glycerol-3	GPD2	GPD2	Glycerol-3	8	427.5439	48.5244	6.21E-51	8	13.89271
Q8N335	351	38418.4	7.03	Glycerol-3	GPD1L	GPD1L	Glycerol-3	4	98.39339	46.47655	5.55E-44	4	11.68091
O75487	556	62411.6	6.65	Glypican-4	GPC4	GPC4	Glypican-4	14	1335.781	53.02676	6.66E-68	13	32.01439
P35052	558	61679.5	7.31	Glypican-1	GPC1	GPC1	Glypican-1	17	12923.58	51.36882	1.92E-61	17	40.14337
Q9HC44	474	52301.2	6.83	Vasculin-li	GPBP1L1	GPBP1L1	Vasculin-li	2	78.76199	47.41482	4.24E-47	2	8.860759
Q86WP2	473	53338.9	7.05	Vasculin	GPBP1	GPBP1	Vasculin	2	187.0954	47.97046	5.33E-49	2	6.342495
Q9UKJ3	1502	164195.9	9	G patch do	GPATCH8	GPATCH8	G patch do	14	439.8922	53.11415	2.97E-68	13	11.4514
Q5T3I0	446	50381	10.37	G patch do	GPATCH4	GPATCH4	G patch do	1	67.73994	45.77196	1.02E-41	1	3.811659
Q96I76	525	59337.5	4.65	G patch do	GPATCH3	GPATCH3	G patch do	1	72.02678	32.34879	3.65E-12	1	2.095238
Q9NWQ4	482	54258.9	8.54	G patch do	GPATCH2L	GPATCH2L	G patch do	3	136.4655	45.19681	6.36E-40	3	7.053942
Q9NW75	528	58943.2	9.63	G patch do	GPATCH2	GPATCH2	G patch do	4	107.1154	48.84304	4.58E-52	4	10.60606
Q8N954	285	33277.1	5.6	G patch do	GPATCH11	GPATCH11	G patch do	3	110.0494	49.82846	1.22E-55	3	11.22807
Q9BRR8	931	103344.1	7.05	G patch do	GPATCH1	GPATCH1	G patch do	8	306.4741	49.77747	1.87E-55	8	10.63373
Q8IXQ4	340	38141.4	5.03	GPALPP m	GPALPP1	GPALPP1	GPALPP m	1	255.1759	48.17133	1.07E-49	1	3.529412
P00505	430	47517.3	9.38	Aspartate a	GOT2	GOT2	Aspartate a	8	411.3231	48.28175	4.42E-50	8	23.02326
P17174	413	46247.1	7.01	Aspartate a	GOT1	GOT1	Aspartate a	3	186.3328	44.20642	6.14E-37	3	9.200969
O95249	250	28612.3	9.89	Golgi SNA	GOSR1	GOSR1	Golgi SNA	1	232.7631	33.67798	6.17E-14	1	4
Q9H8Y8	452	47144.8	4.46	Golgi reass	GORASP2	GORASP2	Golgi reass	2	243.3895	49.91467	5.85E-56	2	6.19469
Q9HD26	462	50519.6	5.79	Golgi-asso	GOPC	GOPC	Golgi-asso	1	221.834	42.57476	2.5E-32	1	1.948052
Q9Y3E0	138	15425.6	11.07	Vesicle tra	GOLT1B	GOLT1B	Vesicle tra	1	34.83694	47.81987	1.76E-48	1	10.14493
Q9H4A5	285	32767.1	5.55	Golgi phos	GOLPH3L	GOLPH3L	Golgi phos	1	104.4871	38.93207	2.04E-23	1	5.263158
Q9H4A6	298	33810.2	6.38	Golgi phos	GOLPH3	GOLPH3	Golgi phos	4	67.97263	51.78328	4.91E-63	3	17.11409
Q2TAP0	167	18334.4	5.83	Golgin sub	GOLGA7B	GOLGA7B	Golgin sub	1	77.40793	39.46804	1.31E-24	1	5.988024
Q7Z5G4	137	15823.9	7.13	Golgin sub	GOLGA7	GOLGA7	Golgin sub	2	148.4077	45.78324	9.36E-42	2	18.24818
Q08379	1002	113085.5	4.7	Golgin sub	GOLGA2	GOLGA2	Golgin sub	2	92.87898	37.70869	7.45E-21	2	2.195609
Q9NVN8	582	65572.7	8.62	Guanine nu	GNL3L	GNL3L	Guanine nu	10	436.4971	47.50566	2.08E-47	10	18.72852
Q9BVP2	549	61992.7	9.75	Guanine nu	GNL3	GNL3	Guanine nu	29	8705.965	51.31841	2.99E-61	28	52.45902
Q13823	731	83654.4	9.85	Nucleolar G	GNL2	GNL2	Nucleolar G	25	1290.348	54.06402	4.55E-72	25	32.01094
P36915	607	68660.3	5.58	Guanine nu	GNL1	GNL1	Guanine nu	10	1182.213	51.51438	5.26E-62	10	18.61614
A0A804HL	68	7251.3	9.55	Guanine nu	GNG5B	GNG5B;GN	Guanine nu	1	136.6012	40.92764	4.62E-28	1	39.70588
Q9UBI6	72	8006.1	9.38	Guanine nu	GNG12	GNG12	Guanine nu	3	142.8683	56.59438	1.48E-82	3	56.94444
Q9Y223	722	79273.9	6.79	Bifunctiona	GNE	GNE	Bifunctiona	5	134.7639	47.25348	1.48E-46	5	8.171745
O14775	395	43565.9	6.42	Guanine nu	GNB5	GNB5	Guanine nu	1	239.8578	44.56941	5.15E-38	1	2.78481
P62879	340	37330.6	5.85	Guanine nu	GNB2	GNB2	Guanine nu	8	4461.717	51.72314	8.35E-63	3	25.58824
Q9BYB4	327	35617.7	8.03	Guanine nu	GNB1L	GNB1L	Guanine nu	4	162.2859	48.17767	1.02E-49	4	16.81957
P62873	340	37376.6	5.85	Guanine nu	GNB1	GNB1	Guanine nu	2	1348.807	50.85122	1.81E-59	2	7.352941
P63092	394	45664.2	5.56	Guanine nu	GNAS	GNAS	Guanine nu	6	401.7671	53.68725	1.57E-70	6	13.45178
P50148	359	42141.7	5.37	Guanine nu	GNAQ	GNAQ	Guanine nu	4	371.4284	50.34823	1.42E-57	4	13.92758
P08754	354	40531.8	5.37	Guanine nu	GNAI3	GNAI3	Guanine nu	4	1288.35	46.86766	2.89E-45	4	14.40678
P04899	355	40450.5	5.2	Guanine nu	GNAI2	GNAI2	Guanine nu	13	3787.067	52.46585	1.07E-65	8	43.09859
P63096	354	40360.7	5.76	Guanine nu	GNAI1	GNAI1	Guanine nu	5	1389.164	51.27813	4.27E-61	5	20.90395
Q14344	377	44049.2	8.13	Guanine nu	GNA13	GNA13	Guanine nu	6	4401.204	51.77916	5.08E-63	5	16.44562

P29992	359	42122.9	5.38	Guanine n	GNA11	GNA11	Guanine n	7	547.5035	48.60977	3.1E-51	4	24.79109
P49915	693	76714.8	6.86	GMP synth	GMPS	GMPS	GMP synth	14	902.0305	51.63439	1.83E-62	14	22.94372
Q9Y5P6	360	39834.1	6.59	Mannose-	GMPPB	GMPPB	Mannose-	4	195.1307	48.8018	6.43E-52	4	12.5
Q96IJ6	420	46290.8	7.23	Mannose-	GMPPA	GMPPA	Mannose-	5	201.6595	45.51856	6.38E-41	5	14.52381
Q8NEA9	526	60298.5	8.25	Germ cell-	GMCL2	GMCL2;GN	Germ cell-	1	187.6651	44.39364	1.71E-37	1	2.281369
Q49A26	553	60547	9.7	Cytokine-l	GLYR1	GLYR1	Cytokine-l	8	391.183	51.77898	5.08E-63	8	18.62568
P15104	373	42064.2	6.88	Glutamine	GLUL	GLUL	Glutamine	3	78.23141	39.73225	3.28E-25	3	8.847185
P00367	558	61397.3	7.91	Glutamate	GLUD1	GLUD1	Glutamate	14	556.1042	49.17552	2.94E-53	3	27.24014
O94925	669	73460.6	7.82	Glutamina	GLS	GLS	Glutamina	6	214.3636	49.53751	1.42E-54	6	11.80867
O76003	335	37431.7	5.09	Glutaredo›	GLRX3	GLRX3	Glutaredo›	4	211.1959	51.87728	2.14E-63	4	14.62687
P35754	106	11775.7	8.21	Glutaredo›	GLRX	GLRX	Glutaredo›	1	180.9342	44.52531	6.95E-38	1	10.37736
Q9HC38	313	34793.2	5.28	Glyoxalase	GLOD4	GLOD4	Glyoxalase	2	52.42014	17.89349	2.38E-05	2	7.667732
Q04760	184	20777.5	4.92	Lactoylglut	GLO1	GLO1	Lactoylglut	2	141.6752	43.75678	1.25E-35	2	10.32609
Q9H4G4	154	17218.3	9.92	Golgi-assc	GLIPR2	GLIPR2	Golgi-assc	4	553.5044	48.37585	2.06E-50	4	36.36364
P10075	376	41144.7	8.94	Zinc finger	GLI4	GLI4	Zinc finger	1	82.62126	48.3588	2.37E-50	1	4.521277
Q92896	1179	134550.6	6.9	Golgi appa	GLG1	GLG1	Golgi appa	17	435.8405	52.83789	3.78E-67	17	17.3028
Q53GS7	698	79835.6	7.47	mRNA exp	GLE1	GLE1	mRNA exp	1	111.749	48.77115	8.27E-52	1	2.292264
P16278	677	76074.2	6.55	Beta-galac	GLB1	GLB1	Beta-galac	2	202.2187	24.26648	1.77E-06	2	3.840473
Q9Y2X7	761	84340.1	6.79	ARF GTPas	GIT1	GIT1	ARF GTPas	3	74.8215	47.72338	3.76E-48	2	4.99343
O14908	333	36048.9	6.19	PDZ doma	GIPC1	GIPC1	PDZ doma	10	629.9326	54.69537	1.2E-74	9	40.24024
Q6Y7W6	1299	150069.3	5.23	GRB10-int	GIGYF2	GIGYF2	GRB10-int	6	243.2071	58.6423	5.27E-91	6	6.235566
Q9NWU2	228	26748.3	4.63	Glucose-ir	GID8	GID8	Glucose-ir	8	259.1968	55.39575	1.59E-77	8	39.91228
Q8IVV7	300	33513.6	9.46	Glucose-ir	GID4	GID4	Glucose-ir	1	217.7975	39.5976	6.66E-25	1	3
Q9H3C7	697	79085.8	6.34	Gametoge	GGNBP2	GGNBP2	Gametoge	8	149.1639	50.98471	5.61E-60	8	13.91679
Q92820	318	35964	7.14	Gamma-g	GGH	GGH	Gamma-g	7	286.5284	49.77969	1.84E-55	7	26.41509
O75223	188	21007.6	4.79	Gamma-g	GGCT	GGCT	Gamma-g	1	66.45754	33.6185	7.5E-14	1	6.914894
Q9UJY4	613	67149.7	6.52	ADP-ribos	GGA2	GGA2	ADP-ribos	2	97.79742	29.5119	3.26E-09	2	3.588907
O94808	682	76929.9	7.4	Glutamine	GFPT2	GFPT2	Glutamine	6	569.1311	50.67132	8.67E-59	6	11.87683
Q06210	699	78805.8	7.11	Glutamine	GFPT1	GFPT1	Glutamine	16	486.9202	50.07623	1.47E-56	14	34.62089
Q969S9	779	86599.7	6.47	Ribosome-	GFM2	GFM2	Ribosome-	2	122.1741	47.98467	4.76E-49	2	3.337612
Q96RP9	751	83470.9	7.01	Elongation	GFM1	GFM1	Elongation	9	346.2004	52.10514	2.78E-64	9	14.24767
Q7L5D6	327	36504	5.11	Golgi to EF	GET4	GET4	Golgi to EF	2	95.01566	25.92474	5.11E-07	2	7.033639
O43681	348	38792.4	4.54	ATPase GE	GET3	GET3	ATPase GE	3	309.1301	43.59198	3.72E-35	3	8.045977
Q9NWZ8	242	28636.4	6.79	Gem-asso	GEMIN8	GEMIN8	Gem-asso	1	82.49926	40.48025	5.69E-27	1	3.719008
Q9H840	131	14536.5	7.51	Gem-asso	GEMIN7	GEMIN7	Gem-asso	1	52.77488	27.05457	1.54E-07	1	9.923664
Q8TEQ6	1508	168588	6.61	Gem-asso	GEMIN5	GEMIN5	Gem-asso	8	281.3966	49.92824	5.21E-56	8	6.034483
P57678	1058	120036.1	5.94	Gem-asso	GEMIN4	GEMIN4	Gem-asso	15	359.8782	51.23221	6.39E-61	15	17.29679
O14893	280	31584.8	5.24	Gem-asso	GEMIN2	GEMIN2	Gem-asso	1	78.12605	48.95905	1.76E-52	1	4.642857
P55040	296	33949.2	8.66	GTP-bindi	GEM	GEM	GTP-bindi	2	117.14	47.10424	4.69E-46	2	8.108108
Q8N9F7	314	36166.8	8.67	Lysophosp	GDPD1	GDPD1	Lysophosp	1	149.8566	49.03799	9.15E-53	1	3.821656
P31150	447	50582.3	4.75	Rab GDP c	GDI1	GDI1	Rab GDP c	5	205.0679	48.70881	1.38E-51	4	12.52796
Q99988	308	34139.8	10.05	Growth/di	GDF15	GDF15	Growth/di	7	819.7669	49.0189	1.07E-52	7	30.51948
Q9NZC3	331	37718	6.7	Glyceroph	GDE1	GDE1	Glyceroph	1	56.45145	31.86092	1.41E-11	1	2.416918
Q9NXN4	497	56224.2	5.5	Gangliosid	GDAP2	GDAP2	Gangliosid	3	76.94078	39.32476	2.76E-24	3	7.042254
Q8TB36	358	41345.3	8.55	Gangliosid	GDAP1	GDAP1	Gangliosid	3	322.7774	47.33466	7.92E-47	3	9.497207
Q9Y2T3	454	51002.6	5.45	Guanine d	GDA	GDA	Guanine d	8	497.4467	51.45718	8.76E-62	8	19.163
Q92616	2671	292706.9	7.44	Stalled ribc	GCN1	GCN1	Stalled ribc	32	482.105	51.44362	9.87E-62	32	14.48896
P48507	274	30726.7	5.91	Glutamate	GCLM	GCLM	Glutamate	3	193.9731	45.05144	1.78E-39	3	16.05839
P48506	637	72765.1	5.98	Glutamate	GCLC	GCLC	Glutamate	3	106.8961	43.95379	3.37E-36	3	6.593407
Q14397	625	68684.7	6.68	Glucokinas	GCKR	GCKR	Glucokinas	1	3558.48	23.20517	3.15E-06	1	1.6
Q92947	438	48126.7	8.15	Glutaryl-C	GCDH	GCDH	Glutaryl-C	2	139.3423	39.65696	4.88E-25	2	9.360731
O75600	419	45284.6	8.11	2-amino-3	GCAT	GCAT	2-amino-3	2	72.81528	48.57985	3.96E-51	2	5.727924
Q92538	1860	206571.9	5.5	Golgi-spec	GBF1	GBF1	Golgi-spec	2	158.3158	50.14764	7.94E-57	2	1.129032
P04062	536	59715.7	7.66	Lysosomal	GBA1	GBA1	Lysosomal	3	141.3247	32.97984	5.66E-13	3	5.970149
A0A0B4J2I	268	28142.2	8.4	Putative gl	GATD3B	GATD3B;G	Putative gl	1	38.60948	22.7016	3.98E-06	1	3.731343
O75879	557	61863.6	8.86	Glutamyl-t	GATB	GATB	Glutamyl-t	2	146.2567	45.90702	3.79E-42	2	5.02693
Q8WXI9	593	65260.2	10.38	Transcripti	GATAD2B	GATAD2B	Transcripti	5	186.1451	48.62522	2.73E-51	5	10.79258
Q86YP4	633	68062.2	10.66	Transcripti	GATAD2A	GATAD2A	Transcripti	11	280.2292	48.79797	6.64E-52	10	23.85466
Q8WUU5	269	28690.3	9.86	GATA zinc	GATAD1	GATAD1	GATA zinc	1	64.17011	42.23719	2.01E-31	1	5.947955
Q92908	595	60031.9	8.43	Transcripti	GATA6	GATA6	Transcripti	2	203.2739	48.08686	2.1E-49	2	4.705882
P22102	1010	107766.3	6.68	Trifunction	GART	GART	Trifunction	19	598.0982	53.22556	1.06E-68	19	25.24752
P41250	739	83164.8	7.04	Glycine--t	GARS1	GARS1	Glycine--t	15	1111.097	53.78227	6.5E-71	15	24.89851
Q9NY12	217	22347.9	11.49	H/ACA rib	GAR1	GAR1	H/ACA rib	4	2888.595	46.01069	1.77E-42	4	17.51152
Q14C86	1478	164978.1	4.86	GTPase-ac	GAPVD1	GAPVD1	GTPase-ac	7	205.604	48.13103	1.47E-49	7	6.7659
P04406	335	36053	8.73	Glyceralde	GAPDH	GAPDH	Glyceralde	12	11148.42	51.67963	1.23E-62	12	46.86567
Q14697	944	106873.1	6.06	Neutral alp	GANAB	GANAB	Neutral alp	18	703.6602	52.23486	8.55E-65	18	22.77542
Q9H2C0	597	67637.8	5.65	Gigaxonin	GAN	GAN	Gigaxonin	2	41.98405	43.5554	4.73E-35	2	6.197655
Q10471	571	64732.4	8.46	Polypeptid	GALNT2	GALNT2	Polypeptid	4	154.358	52.72592	1.04E-66	4	10.85814
P51570	392	42271.8	6.42	Galactokin	GALK1	GALK1	Galactokin	10	1054.562	49.5986	8.47E-55	10	30.35714
Q14376	348	38281.4	6.71	UDP-gluco	GALE	GALE	UDP-gluco	5	272.4556	52.18467	1.35E-64	5	15.22989
O14976	1311	143189.2	5.47	Cyclin-G-a	GAK	GAK	Cyclin-G-a	5	141.3619	48.31661	3.33E-50	5	4.576659
Q8TAE8	222	25383.6	10.64	Large ribos	GADD45GI	GADD45G	Large ribos	7	785.2396	51.35724	2.13E-61	7	38.28829
Q9UBS5	961	108319.4	8.3	Gamma-ar	GABBR1	GABBR1	Gamma-ar	1	165.6696	31.21682	7.49E-11	1	2.289282
P10253	952	105322.9	5.91	Lysosomal	GAA	GAA	Lysosomal	4	166.8921	51.27902	4.24E-61	4	6.092437
P11413	515	59256.3	6.84	Glucose-6	G6PD	G6PD	Glucose-6	21	1122.535	49.52255	1.61E-54	21	45.43689
Q9UN86	482	54120.9	5.26	Ras GTPas	G3BP2	G3BP2	Ras GTPas	6	254.6086	51.14252	1.41E-60	6	14.10788
Q13283	466	52164	5.21	Ras GTPas	G3BP1	G3BP1	Ras GTPas	6	274.6579	49.86417	8.98E-56	6	15.87983
Q7L622	706	80503.4	7.68	G2/M phas	G2E3	G2E3	G2/M phas	3	58.66972	34.67274	1.99E-15	3	5.240793
Q96QD9	318	35818.2	12.3	UAP56-int	FYTTD1	FYTTD1	UAP56-int	4	202.6827	47.50159	2.15E-47	4	13.83648
P51116	673	74222.7	6.07	RNA-bind	FXR2	FXR2	RNA-bind	16	768.1741	50.93195	8.91E-60	14	31.35215
P51114	621	69720.3	6.03	RNA-bind	FXR1	FXR1	RNA-bind	20	1752.854	50.28436	2.46E-57	20	38.16425
P35637	526	53426	9.59	RNA-bind	FUS	FUS	RNA-bind	7	13307.69	49.6231	6.88E-55	5	15.77947
Q96I24	572	61640.1	8.55	Far upstrea	FUBP3	FUBP3	Far upstrea	12	934.8299	51.25623	5.18E-61	12	25.34965
Q96AE4	644	67560.2	7.71	Far upstrea	FUBP1	FUBP1	Far upstrea	3	135.3526	50.20062	5.04E-57	3	5.900621
Q8IY81	847	96557.7	8.71	pre-rRNA	FTSJ3	FTSJ3	pre-rRNA	18	2733.508	52.50004	7.85E-66	18	24.4392
Q9UET6	329	36078.8	5.39	tRNA (cytic	FTSJ1	FTSJ1	tRNA (cytic	4	2228.6	46.44598	6.99E-44	4	16.41337
Q9C0B1	505	58281.5	4.88	Alpha-ketc	FTO	FTO	Alpha-ketc	5	144.4581	50.1328	9.03E-57	5	12.07921
P02792	175	20019.5	5.59	Ferritin ligl	FTL	FTL	Ferritin ligl	3	669.6925	49.84005	1.1E-55	3	17.71429
P02794	183	21225.5	5.29	Ferritin hea	FTH1	FTH1	Ferritin hea	5	450.9895	51.17905	1.02E-60	5	30.05464
Q5CZC0	6907	780600.1	6.68	Fibrous sh	FSIP2	FSIP2	Fibrous sh	2	16.02477	18.25221	2.11E-05	2	0.318517
Q9BTV5	496	55819.2	6.99	Fibronectir	FSD1	FSD1	Fibronectir	2	24.10603	34.60324	2.56E-15	2	5.443548
Q16658	493	54529.5	7.26	Fascin	FSCN1	FSCN1	Fascin	10	463.9136	54.50469	7.45E-74	10	24.54361
Q9BZ67	464	51217.5	6.16	FERM dom	FRMD8	FRMD8	FERM dom	2	82.15223	48.5346	5.71E-51	2	7.112069

Q96NE9	622	72043.3	7.48	FERM dom	FRMD6	FRMD6	FERM dom	3	166.7155	54.90385	1.6E-75	3	5.144695
Q7Z6J6	570	65064	8.46	FERM dom	FRMD5	FRMD5	FERM dom	3	51.9649	45.61711	3.12E-41	3	5.263158
Q9Y2L6	1034	118045.8	8.83	FERM dom	FRMD4B	FRMD4B	FERM dom	1	655.7277	44.02276	2.12E-36	1	0.870406
Q9P2Q2	1039	115457.3	9.21	FERM dom	FRMD4A	FRMD4A	FERM dom	1	22.06679	23.10654	3.3E-06	1	0.866218
Q14331	258	29172.1	9.54	Protein FR	FRG1	FRG1	Protein FR	8	518.5425	53.55451	5.29E-70	8	37.5969
Q70Z53	315	37547.8	8.27	Protein FR.	FRA10AC1	FRA10AC1	Protein FR.	2	57.02189	37.95006	2.42E-21	2	7.301587
Q96CU9	486	53811.2	7.88	FAD-depe	FOXRED1	FOXRED1	FAD-depe	3	131.2654	47.31573	9.16E-47	3	6.378601
Q01167	660	69061.5	10.06	Forkhead I	FOXK2	FOXK2	Forkhead I	1	264.54	33.24004	2.52E-13	1	1.515152
Q9UPW0	622	68959.9	7.09	Forkhead I	FOXJ3	FOXJ3	Forkhead I	1	18.85469	17.45075	2.77E-05	1	2.411576
Q12948	553	56788.3	8.67	Forkhead I	FOXC1	FOXC1	Forkhead I	1	104.3053	41.63739	7.49E-30	1	2.169982
P55317	472	49148.6	9.68	Forkhead I	**FOXA1**	**FOXA1**	Forkhead I	2	198.857	41.12234	1.51E-28	2	2.319236
P15408	326	35193.1	7.6	Fos-relate	FOSL2	FOSL2	Fos-relate	1	71.94738	27.48025	8.96E-08	1	3.680982
P15328	257	29818.9	8.02	Folate rece	FOLR1	FOLR1	Folate rece	2	124.7228	43.29044	2.66E-34	2	8.560311
Q53EP0	1204	132887.2	5.82	Fibronectir	FNDC3B	FNDC3B	Fibronectir	10	332.8368	49.71997	3.03E-55	10	10.46512
Q8N3X1	1017	110264.9	4.39	Formin-bi	FNBP4	FNBP4	Formin-bi	10	433.4018	53.76581	7.61E-71	10	12.0944
Q06787	632	71174	7.46	Fragile X n	FMR1	FMR1	Fragile X n	7	252.1398	53.31312	4.81E-69	7	12.65823
Q96PY5	1086	123319.8	7.43	Formin-lik	FMNL2	FMNL2	Formin-lik	6	128.294	49.96936	3.68E-56	5	6.629834
Q14254	428	47063.8	4.91	Flotillin-2	FLOT2	FLOT2	Flotillin-2	15	603.8654	56.96816	5.54E-84	15	43.2243
O75955	427	47355	7.58	Flotillin-1	FLOT1	FLOT1	Flotillin-1	13	323.6481	50.84879	1.85E-59	13	42.38876
Q14315	2725	291020.5	5.82	Filamin-C	FLNC	FLNC	Filamin-C	44	1128.768	53.82419	4.32E-71	35	19.92661
O75369	2602	278162.4	5.46	Filamin-B	FLNB	FLNB	Filamin-B	58	705.3061	56.60003	1.47E-82	58	29.3236
P21333	2647	280737.6	5.95	Filamin-A	FLNA	FLNA	Filamin-A	60	1119.166	52.74414	8.79E-67	60	31.12958
Q13045	1269	144749.9	5.91	Protein flic	FLII	FLII	Protein flic	27	636.4359	51.32952	2.71E-61	27	24.34988
Q5D862	2391	248073.3	8.35	Filaggrin-2	FLG2	FLG2	Filaggrin-2	1	111.5604	54.55064	4.83E-74	1	0.501882
Q8NFF5	587	65265	6.92	FAD synth	FLAD1	FLAD1	FAD synth	1	120.2556	52.33481	3.49E-65	1	2.214651
Q13451	457	51211.8	5.66	Peptidyl-p	FKBP5	FKBP5	Peptidyl-p	5	259.198	50.24622	3.42E-57	5	12.03501
Q02790	459	51804.2	5.11	Peptidyl-p	FKBP4	FKBP4	Peptidyl-p	5	140.9092	48.54737	5.15E-51	5	15.25054
Q6UN15	594	66525.8	5.28	Pre-mRNA	FIP1L1	FIP1L1	Pre-mRNA	7	3421.966	50.32658	1.71E-57	7	13.63636
O43427	364	41877.6	6.44	Acidic fibr	FIBP	FIBP	Acidic fibr	2	127.8865	48.98393	1.43E-52	2	6.043956
Q13643	280	31191.7	6.15	Four and a	FHL3	FHL3	Four and a	2	46.71754	32.19873	5.59E-12	2	10.71429
Q14192	279	32192.6	7.57	Four and a	FHL2	FHL2	Four and a	7	257.3784	54.6761	1.45E-74	7	27.59857
Q13642	323	36262.8	9.15	Four and a	FHL1	FHL1	Four and a	6	226.7938	49.12692	4.39E-53	6	17.64706
P07954	510	54636.6	9.17	Fumarate I	FH	FH	Fumarate I	1	59.82072	29.50511	3.3E-09	1	2.745098
P02679	453	51511.3	5.32	Fibrinogen	FGG	FGG	Fibrinogen	7	144.9485	56.00592	4.17E-80	7	16.55629
P09038	288	30769.7	11.71	Fibroblast	FGF2	FGF2	Fibroblast	2	115.7514	48.10518	1.81E-49	2	6.944444
P02675	491	55927.9	8.38	Fibrinogen	FGB	FGB	Fibrinogen	3	106.3241	50.81067	2.59E-59	3	7.535642
P02671	866	94972.5	5.87	Fibrinogen	FGA	FGA	Fibrinogen	2	44.4931	41.18473	1.06E-28	2	1.963048
P07332	822	93495.9	6.72	Tyrosine-p	FES	FES;FER	Tyrosine-p	1	125.7181	47.3504	7.01E-47	1	1.3382
Q9BQL6	677	77436	6.18	Fermitin fa	FERMT1	FERMT1	Fermitin fa	1	118.6827	39.88845	1.43E-25	1	1.920236
P39748	380	42592.6	8.92	Flap endor	FEN1	FEN1	Flap endor	4	324.2798	49.35675	6.49E-54	4	13.94737
P22830	423	47861.8	8.91	Ferrochela	FECH	FECH	Ferrochela	5	207.2653	50.90604	1.12E-59	5	16.31206
P22570	491	53836.4	8.56	NADPH:ad	FDXR	FDXR	NADPH:ad	7	431.8918	49.72087	3.01E-55	7	17.10794
P14324	419	48275	6.02	Farnesyl py	FDPS	FDPS	Farnesyl py	2	119.7808	47.0025	1.03E-45	2	4.534606
P37268	417	48114.9	6.51	Squalene s	FDFT1	FDFT1	Squalene s	1	141.0278	42.82099	5.32E-33	1	2.877698
Q0JRZ9	810	88923.6	6.84	F-BAR dor	FCHO2	FCHO2	F-BAR dor	1	141.9013	22.91587	3.61E-06	1	1.728395
Q9Y324	198	23369.4	10.25	rRNA-proc	FCF1	FCF1	rRNA-proc	3	483.724	49.68417	4.11E-55	3	17.17172
Q9UKB1	542	62090.2	7.13	F-box/WD	FBXW11	FBXW11	F-box/WD	16	622.4493	52.93976	1.48E-67	7	30.2583
Q6PIJ6	1188	133942.7	6.29	F-box only	FBXO38	FBXO38	F-box only	10	399.8643	52.57364	4.06E-66	10	10.18519
Q5XUX0	539	60663.3	7.02	F-box only	FBXO31	FBXO31	F-box only	1	182.1991	22.18108	5E-06	1	1.855288
Q9UK99	471	54559.9	4.62	F-box only	FBXO3	FBXO3	F-box only	1	23.67259	41.93792	1.24E-30	1	1.910828
Q8NEZ5	403	44508	7.04	F-box only	FBXO22	FBXO22	F-box only	8	405.3781	47.82877	1.64E-48	8	26.05459
Q9UK22	296	33327.5	4.01	F-box only	FBXO2	FBXO2	F-box only	7	407.2184	51.97536	8.95E-64	7	32.43243
Q86XK2	927	103584.4	6.99	F-box only	FBXO11	FBXO11	F-box only	4	157.904	43.21952	4.19E-34	4	5.393743
Q8N531	539	58557.4	9.63	F-box/LRR	FBXL6	FBXL6	F-box/LRR	5	325.9937	47.85793	1.3E-48	5	10.20408
Q96ME1	718	78918.5	8.31	F-box/LRR	FBXL18	FBXL18	F-box/LRR	3	87.16628	47.16639	2.9E-46	3	5.292479
P22087	321	33784.1	10.81	rRNA 2-O	FBL	FBL	rRNA 2'-O	11	3872.58	54.26898	6.86E-73	11	42.3676
P62861	133	14389.7	10.91	Ubiquitin-	FAU	FAU	Ubiquitin-	3	23483.72	50.58693	1.82E-58	3	9.022556
Q14517	4588	506268.5	4.61	Protocadh	FAT1	FAT1	Protocadh	24	436.3461	53.47543	1.08E-69	24	6.734961
Q9NYY8	710	81462.1	8.12	FAST kinas	FASTKD2	FASTKD2	FAST kinas	2	84.04382	41.59652	9.54E-30	2	3.098592
Q53R41	847	97409.7	7.79	FAST kinas	FASTKD1	FASTKD1	FAST kinas	1	19.33484	19.11503	1.55E-05	1	1.416765
P49327	2511	273424.1	6.41	Fatty acid	FASN	FASN	Fatty acid	61	1628.336	54.96153	9.21E-76	61	33.85106
Q9NSD9	589	66114.9	6.83	Phenylalar	FARSB	FARSB	Phenylalar	15	507.5862	52.66141	1.85E-66	15	28.35314
Q9Y285	508	57563.2	7.96	Phenylalar	FARSA	FARSA	Phenylalar	7	178.6781	46.12221	7.81E-43	7	20.66929
O95363	451	52356.2	7.48	Phenylalar	FARS2	FARS2	Phenylalar	3	208.1492	42.32953	1.14E-31	3	7.095344
O94887	1054	119887.5	9.05	FERM, ARH	FARP2	FARP2	FERM, ARH	10	284.644	49.65235	5.36E-55	10	11.95446
Q9Y4F1	1045	118631.9	8.27	FERM, ARH	FARP1	FARP1	FERM, ARH	10	433.3508	55.00744	6.09E-76	10	13.58852
Q8WVX9	515	59356.3	9.52	Fatty acyl-	FAR1	FAR1	Fatty acyl-	3	78.36724	43.73026	1.49E-35	3	6.796117
Q9BXW9	1451	164126.7	5.73	Fanconi an	FANCD2	FANCD2	Fanconi an	1	39.65659	50.53931	2.75E-58	1	0.964852
Q52LJ0	433	45547	8.93	Protein FA	FAM98B	FAM98B	Protein FA	7	378.9929	51.73149	7.75E-63	7	22.63279
Q8NCA5	518	55272.4	9.25	Protein FA	FAM98A	FAM98A	Protein FA	8	3470.549	53.11869	2.87E-68	7	19.49807
Q658Y4	838	93908.1	6.36	Protein FA	FAM91A1	FAM91A1	Protein FA	19	942.6255	52.65922	1.88E-66	17	24.58234
Q9UBU6	413	44122.9	7.23	Protein FA	FAM8A1	FAM8A1	Protein FA	1	135.871	48.16678	1.11E-49	1	4.116223
Q6ZRV2	1179	127121	6.97	Protein FA	FAM83H	FAM83H	Protein FA	3	121.606	43.07302	1.07E-33	3	3.901612
A6ND36	823	90834.3	6.35	Protein FA	FAM83G	FAM83G	Protein FA	1	125.5255	28.26918	2.86E-08	1	1.09356
Q8NEG4	500	55485.3	8.34	Protein FA	FAM83F	FAM83F	Protein FA	2	155.1685	47.94218	6.68E-49	2	5.6
Q5T0W9	1011	114798.3	9.37	Protein FA	FAM83B	FAM83B	Protein FA	3	77.63159	48.21413	7.59E-50	3	3.659743
Q5HYJ3	339	38707.7	9.76	Protein FA	FAM76B	FAM76B	Protein FA	14	5368.521	51.54723	3.94E-62	13	41.88791
Q8TAV0	307	35049	9.62	Protein FA	FAM76A	FAM76A	Protein FA	7	792.5856	50.29113	2.32E-57	7	25.40717
Q9NYF3	392	43090.9	8.74	Protein FA	FAM53C	FAM53C	Protein FA	1	54.07886	52.35574	2.9E-65	1	2.806122
Q14320	339	40241.3	6.82	Protein FA	FAM50A	FAM50A	Protein FA	1	263.861	47.55429	1.42E-47	1	4.129794
Q92520	227	24680.3	8.46	Protein FA	FAM3C	FAM3C	Protein FA	6	481.1037	49.59475	8.74E-55	6	30.39648
Q96D05	121	13238.3	12.08	Protein FA	FAM241B	FAM241B	Protein FA	1	138.9168	27.46182	9.19E-08	1	10.7438
Q9ULE4	1060	121042.9	6.07	Protein FA	FAM184B	FAM184B	Protein FA	1	905.3802	43.55555	4.72E-35	1	0.943396
Q9Y6X4	670	74954.1	4.24	Soluble lar	FAM169A	FAM169A	Soluble lar	1	222.0496	40.49711	5.18E-27	1	1.19403
Q96A26	154	17342.2	10.48	Protein FA	FAM162A	FAM162A	Protein FA	1	146.4418	47.20968	2.08E-46	1	7.142857
Q96EK7	910	103782.5	5.44	Constitutiv	FAM120B	FAM120B	Constitutiv	19	334.8713	53.57295	4.47E-70	19	25.16484
Q9NZB2	1118	121886.7	9.11	Constitutiv	FAM120A	FAM120A	Constitutiv	28	1466.616	53.72425	1.12E-70	26	30.14311
Q6P1L5	589	61967.3	10.49	Protein FA	FAM117B	FAM117B	Protein FA	7	321.4952	49.76214	2.13E-55	7	16.29881
Q9C073	453	48318	8.39	Protein FA	FAM117A	FAM117A	Protein FA	10	445.2368	51.9994	7.22E-64	10	26.04857
Q96PZ2	611	70195.9	8.71	Serine pro	FAM111A	FAM111A	Serine pro	2	284.8783	47.23188	1.75E-46	2	3.436989
Q96GK7	314	34596.1	8.38	Oxaloaceta	FAHD2A	FAHD2A	Oxaloaceta	1	71.219	39.19213	5.46E-24	1	3.503185
Q96CS3	445	52623	5.32	FAS-assoc	FAF2	FAF2	FAS-assoc	10	798.5412	51.49347	6.31E-62	10	31.68539
A8MUU1	101	11298.8	7.95	Putative fa	FABP5P3	FABP5P3	Putative fa	2	47.34592	46.773	5.92E-45	1	28.71287

Q01469	135	15164.4	7.05	Fatty acid-	FABP5	FABP5	Fatty acid-	1	150.0908	44.68901	2.25E-38	1	8.148148
P00734	622	70036.3	5.7	Prothromb	F2	F2	Prothromb	1	124.9341	38.28315	4.97E-22	1	1.446945
Q9Y624	299	32582.8	8.02	Junctional	F11R	F11R	Junctional	5	179.8368	51.68175	1.21E-62	5	18.7291
P15311	586	69412.3	6.17	Ezrin	EZR	EZR	Ezrin	19	1971.354	53.2454	8.92E-69	11	33.27645
Q15910	746	85362.4	7.01	Histone-ly	EZH2	EZH2	Histone-ly	14	795.24	52.30746	4.46E-65	11	22.25201
Q92800	747	85270.4	7.77	Histone-ly	EZH1	EZH1	Histone-ly	1	424.1884	23.2257	3.11E-06	1	2.677376
Q9UBQ6	330	37465.4	9.24	Exostosin-	EXTL2	EXTL2	Exostosin-	1	170.1822	50.49395	4.08E-58	1	3.333333
Q06265	439	48948.5	4.93	Exosome c	EXOSC9	EXOSC9	Exosome c	4	458.9467	48.79388	6.86E-52	4	10.25057
Q96B26	276	30039.3	4.94	Exosome c	EXOSC8	EXOSC8	Exosome c	5	332.5309	50.0898	1.31E-56	5	23.55072
Q15024	291	31821.1	4.83	Exosome c	EXOSC7	EXOSC7	Exosome c	7	212.1418	48.68359	1.7E-51	7	34.02062
Q5RKV6	272	28234.8	6.09	Exosome c	EXOSC6	EXOSC6	Exosome c	4	477.2051	45.40151	1.48E-40	4	17.27941
Q9NQT4	235	25248.6	7.66	Exosome c	EXOSC5	EXOSC5	Exosome c	3	625.0881	50.3071	2.02E-57	3	12.76596
Q9NPD3	245	26382.7	6.51	Exosome c	EXOSC4	EXOSC4	Exosome c	5	215.3409	48.17053	1.08E-49	5	22.85714
Q9NQT5	275	29571.9	8.2	Exosome c	EXOSC3	EXOSC3	Exosome c	4	132.026	45.38396	1.68E-40	4	23.63636
Q13868	293	32788.7	7.58	Exosome c	EXOSC2	EXOSC2	Exosome c	6	781.1079	53.92679	1.66E-71	6	25.25597
Q01780	885	100830.3	8.65	Exosome c	EXOSC10	EXOSC10	Exosome c	15	358.898	50.66325	9.28E-59	15	22.59887
Q9Y3B2	195	21451.6	8.36	Exosome c	EXOSC1	EXOSC1	Exosome c	4	150.9597	50.06412	1.63E-56	4	28.20513
Q9UPT5	735	83381.2	6.77	Exocyst co	EXOC7	EXOC7	Exocyst co	1	70.39262	25.92947	5.09E-07	1	2.176871
Q8TAG9	804	93721.6	6.13	Exocyst co	EXOC6	EXOC6	Exocyst co	2	37.9756	34.86504	9.89E-16	2	2.985075
O00471	708	81852.2	6.7	Exocyst co	EXOC5	EXOC5	Exocyst co	3	119.0216	40.12154	4.07E-26	3	3.954802
Q96A65	974	110497	6.45	Exocyst co	EXOC4	EXOC4	Exocyst co	4	74.69923	43.20352	4.65E-34	4	5.338809
O60645	745	85566.7	6.2	Exocyst co	EXOC3	EXOC3	Exocyst co	2	86.37924	44.25453	4.43E-37	2	2.550336
Q96KP1	924	104065.3	6.89	Exocyst co	EXOC2	EXOC2	Exocyst co	3	76.29514	28.15358	3.42E-08	3	3.571429
Q9NVH0	621	70352.1	8.43	Exonucleas	EXD2	EXD2	Exonucleas	9	393.0167	51.6793	1.23E-62	9	16.90821
Q01844	656	68478.2	9.56	RNA-bind	EWSR1	EWSR1	RNA-bind	1	674.8765	47.38734	5.26E-47	1	2.134146
Q92817	2033	231602.2	6.94	Envoplakir	EVPL	EVPL	Envoplakir	4	122.3762	38.97242	1.67E-23	4	2.115101
P41212	452	52999.4	7.46	Transcripti	ETV6	ETV6	Transcripti	8	251.3753	51.42818	1.13E-61	8	23.23009
P41162	512	57000.8	8.82	ETS transl	ETV3	ETV3	ETS transl	1	63.13441	49.96688	3.75E-56	1	3.515625
O95571	254	27872.6	6.83	Persulfide	ETHE1	ETHE1	Persulfide	4	342.1354	48.12713	1.52E-49	4	19.68504
Q16134	617	68495	7.59	Electron tr	ETFDH	ETFDH	Electron tr	1	145.86	49.04186	8.86E-53	1	1.944895
P38117	255	27843.4	8.31	Electron tr	ETFB	ETFB	Electron tr	6	703.3722	52.85574	3.21E-67	6	23.52941
P13804	333	35079.2	8.57	Electron tr	ETFA	ETFA	Electron tr	10	207.9372	51.82188	3.5E-63	10	44.44444
P62495	437	49030.5	5.41	Eukaryotic	ETF1	ETF1	Eukaryotic	7	971.4377	50.44495	6.21E-58	7	17.62014
A0FGR8	921	102356.5	9.8	Extended s	ESYT2	ESYT2	Extended s	13	383.7855	50.54792	2.55E-58	13	17.58958
Q9BSJ8	1104	122855.3	5.61	Extended s	ESYT1	ESYT1	Extended s	13	201.6358	50.9493	7.65E-60	13	14.40217
P11474	423	45509.1	6.33	Steroid ho	ESRRA	ESRRA	Steroid ho	2	62.49625	47.1861	2.49E-46	1	7.328605
Q9H6T0	727	78400.3	6.69	Epithelial s	ESRP2	ESRP2	Epithelial s	2	81.63747	36.42429	2.14E-18	2	3.576341
Q6NXG1	681	75584.7	6.67	Epithelial s	ESRP1	ESRP1	Epithelial s	9	993.8832	50.60294	1.58E-58	9	17.4743
Q9H501	851	98795.3	4.74	ESF1 homc	ESF1	ESF1	ESF1 homc	14	302.5565	47.38171	5.49E-47	14	18.21387
Q5FWF5	840	94982.2	9.73	N-acetyltra	ESCO1	ESCO1	N-acetyltra	1	177.9168	35.75592	3.28E-17	1	1.071429
Q9UJM3	462	50559.5	8.2	ERBB recep	ERRFI1	ERRFI1	ERBB recep	1	327.4075	42.81829	5.41E-33	1	3.030303
Q9BS26	406	46970.7	4.9	Endoplasm	ERP44	ERP44	Endoplasm	10	642.2906	51.35768	2.12E-61	10	27.58621
P30040	261	28993.2	7.49	Endoplasm	ERP29	ERP29	Endoplasm	3	168.5677	47.36661	6.17E-47	3	14.17625
Q96HE7	468	54392.1	5.41	ERO1-like	ERO1A	ERO1A	ERO1-like	1	154.1465	48.63842	2.45E-51	1	2.564103
Q7Z2K6	904	100230.4	7.55	Endoplasm	ERMP1	ERMP1	Endoplasm	8	576.7567	50.32954	1.67E-57	8	9.070796
O94905	339	37839.2	5.33	Erlin-2	ERLIN2	ERLIN2	Erlin-2	8	1355.477	48.36808	2.2E-50	6	26.84366
O75477	348	39170.8	8.06	Erlin-1	ERLIN1	ERLIN1	Erlin-1	9	1206.39	50.97569	6.06E-60	9	28.73563
O43414	337	37237.9	8.16	ERI1 exorib	ERI3	ERI3	ERI1 exorib	3	230.8283	48.60777	3.15E-51	3	11.27596
Q8IV48	349	40063.6	6.67	3-5 exorib	ERI1	ERI1	3'-5' exoril	9	2060.708	50.03839	2.03E-56	9	29.51289
P84090	104	12258.8	5.72	Enhancer c	ERH	ERH	Enhancer c	5	7758.313	48.15111	1.25E-49	5	36.53846
Q969X5	290	32592	7.08	Endoplasm	ERGIC1	ERGIC1	Endoplasm	6	358.981	50.36473	1.24E-57	6	21.03448
P0DP91	1061	119485.9	6.55	Chimeric E	ERCC6	ERCC6	Chimeric E	11	461.2601	49.0394	9.04E-53		10.65033
Q03468	1493	168414.9	8.2	DNA excisi	ERCC6	ERCC6	DNA excisi	4	207.3742	53.49026	9.44E-70	4	2.545211
P19447	782	89277	7.24	General tra	ERCC3	ERCC3	General tra	15	438.3561	52.5891	3.54E-66	15	26.08696
P18074	760	86908.3	7.16	General tra	ERCC2	ERCC2	General tra	2	49.90051	42.25335	1.82E-31	2	3.157895
Q96RT1	1412	158296.8	5.18	Erbin	ERBIN	ERBIN	Erbin	2	148.3002	29.79087	1.87E-09	2	1.699717
O75616	437	48349.7	9.12	GTPase Era	ERAL1	ERAL1	GTPase Era	5	445.4534	51.5306	4.56E-62	5	12.35698
Q9H6S3	715	80620	6.83	Epidermal	EPS8L2	EPS8L2	Epidermal	3	253.0936	38.28096	5.02E-22	3	4.615385
Q12929	822	91880.8	7.56	Epidermal	EPS8	EPS8	Epidermal	1	87.68504	49.72597	2.88E-55	1	1.946472
P07814	1512	170589.7	7.34	Bifunction	EPRS1	EPRS1	Bifunction	30	1048.08	53.36263	3.08E-69	30	26.52116
P58107	5088	555653.1	5.31	Epiplakin	EPPK1	EPPK1	Epiplakin	3	54.39991	50.37234	1.16E-57	3	4.579403
Q7L775	607	70368.9	6.01	EPM2A-int	EPM2AIP1	EPM2AIP1	EPM2A-int	19	1684.774	50.27103	2.76E-57	19	42.99835
P07099	455	52948.5	7.28	Epoxide hy	EPHX1	EPHX1	Epoxide hy	7	316.0626	48.48599	8.48E-51	7	14.28571
P29317	976	108265.6	6.14	Ephrin typ	EPHA2	EPHA2	Ephrin typ	14	541.0431	49.87501	8.2E-56	13	17.41803
P21709	976	108126.3	6.63	Ephrin typ	EPHA1	EPHA1	Ephrin typ	1	47.27954	37.49936	1.94E-20	1	1.536885
Q9UM22	224	25436.9	6.45	Mammalia	EPDR1	EPDR1	Mammalia	2	200.4936	37.94022	2.53E-21	2	8.928571
Q17RA5	79	8913.3	8.07	Putative ur	EPCIP-AS1	EPCIP-AS1	Putative ur	1	99.57352	24.18302	1.86E-06	1	17.72152
P16422	314	34932	7.53	Epithelial c	EPCAM	EPCAM	Epithelial c	6	1084.135	51.83441	3.13E-63	6	19.10828
Q9HCM4	733	81855.5	6.56	Band 4.1-l	EPB41L5	EPB41L5	Band 4.1-l	10	775.12	50.71782	5.8E-59	10	14.46112
Q9H329	900	99711.6	9.41	Band 4.1-l	EPB41L4B	EPB41L4B	Band 4.1-l	7	362.9459	49.19148	2.58E-53	7	10
Q9HCS5	686	79058.1	9.84	Band 4.1-l	EPB41L4A	EPB41L4A	Band 4.1-l	2	111.0881	42.68699	1.24E-32	2	2.769679
O43491	1005	112587.4	5.13	Band 4.1-l	EPB41L2	EPB41L2	Band 4.1-l	23	835.6688	52.3671	2.61E-65	21	28.85572
Q9H4G0	881	98501.9	5.31	Band 4.1-l	EPB41L1	EPB41L1	Band 4.1-l	19	1063.534	49.27298	1.31E-53	18	26.44722
P11171	864	97016.1	5.27	Protein 4.1	EPB41	EPB41	Protein 4.1	7	277.0179	49.16971	3.09E-53	7	9.837963
Q9NPA8	101	11528.5	10.08	Transcripti	ENY2	ENY2	Transcripti	3	390.73	46.43398	7.64E-44	3	34.65347
Q9Y5L3	495	53665	8.34	Ectonuclec	ENTPD2	ENTPD2	Ectonuclec	2	77.0756	36.50322	1.54E-18	2	6.262626
P22413	925	104923.6	7.14	Ectonuclec	ENPP1	ENPP1	Ectonuclec	1	12.42824	37.33843	4.03E-20	1	1.297297
P13929	434	46986.5	7.84	Beta-enola	ENO3	ENO3	Beta-enola	1	1179.483	48.42381	1.41E-50	1	3.456221
P09104	434	47268.1	4.65	Gamma-er	ENO2	ENO2	Gamma-er	2	27.87676	42.12607	3.97E-31	2	5.760369
P06733	434	47168.6	7.46	Alpha-enc	ENO1	ENO1	Alpha-enc	10	1788.329	54.14283	2.17E-72	9	33.17972
Q8NFI3	743	83986.1	6.78	Cytosolic e	ENGASE	ENGASE	Cytosolic e	4	314.2314	46.11274	8.37E-43	4	6.729475
O94919	500	55016.1	5.38	Endonucle	ENDOD1	ENDOD1	Endonucle	8	678.4763	50.32282	1.77E-57	8	20.8
Q7Z589	1322	141466.9	9.95	BRCA2-int	EMSY	EMSY	BRCA2-int	2	108.4811	38.56376	1.27E-22	2	1.966717
Q9HC35	981	108915.4	6.37	Echinoderr	EML4	EML4	Echinoderr	8	347.5423	51.58218	2.9E-62	8	9.276249
O95834	649	70678.4	6.29	Echinoderr	EML2	EML2	Echinoderr	2	88.22903	43.43459	1.04E-34	2	4.622496
Q92979	244	26719.9	9.68	Ribosomal	EMG1	EMG1	Ribosomal	7	269.0638	53.44717	1.4E-69	7	38.11475
P50402	254	28993.5	5.17	Emerin	EMD	EMD	Emerin	11	3133.032	48.80883	6.07E-52	11	49.6063
O43402	210	23772.8	6.38	ER membr	EMC8	EMC8	ER membr	3	96.56184	47.79337	2.16E-48	3	14.7619
Q9NPA0	242	26470.4	9.76	Endoplasm	EMC7	EMC7	Endoplasm	1	118.4077	38.40699	2.73E-22	1	4.132231
Q9BV81	110	12017.2	10.43	ER membr	EMC6	EMC6	ER membr	2	617.9478	50.66446	9.2E-59	2	20
Q5J8M3	183	20086.4	8.83	ER membr	EMC4	EMC4	ER membr	1	101.0844	46.21612	3.89E-43	1	7.103825
Q15006	297	34833.2	6.53	ER membr	EMC2	EMC2	ER membr	4	223.032	51.99681	7.38E-64	4	16.16162
Q8N766	993	111758.5	7.73	ER membr	EMC1	EMC1	ER membr	8	210.9614	48.74336	1.04E-51	8	10.47331

Q9H9T3	547	62258.4	9.12	Elongator	ELP3	ELP3	Elongator	7	375.9702	47.17703	2.67E-46	7	15.17367
Q6IA86	826	92499.4	5.85	Elongator	ELP2	ELP2	Elongator	2	90.15485	48.61021	3.09E-51	2	2.663438
O95163	1332	150253.1	5.8	Elongator	ELP1	ELP1	Elongator	7	163.1838	49.4203	3.81E-54	7	6.306306
Q9BW60	279	32662.5	9.9	Very long	ELOVL1	ELOVL1	Very long	2	58.49182	51.20992	7.77E-61	2	9.677419
Q15369	112	12473.1	4.45	Elongin-C	ELOC	ELOC	Elongin-C	5	243.5619	55.98124	5.21E-80	5	47.32143
Q15370	118	13132.7	4.48	Elongin-B	ELOB	ELOB	Elongin-B	6	431.0549	51.80033	4.24E-63	6	46.61017
Q14241	772	87229.3	10.21	Elongin-A	ELOA	ELOA	Elongin-A	1	50.27692	37.22887	6.59E-20	1	2.072539
Q8IZ81	293	34960.4	8.19	ELMO don	ELMOD2	ELMOD2	ELMO don	6	164.9604	45.06683	1.6E-39	6	22.5256
Q9HB65	397	45360.4	5.96	RNA polyn	ELL3	ELL3	RNA polyn	1	47.26142	32.75934	1.1E-12	1	3.274559
O00472	640	72323.8	9.4	RNA polyn	ELL2	ELL2	RNA polyn	6	264.7924	49.10092	5.44E-53	6	12.8125
P55199	621	68264	9.78	RNA polyn	ELL	ELL	RNA polyn	16	446.3358	51.14065	1.43E-60	16	34.13849
Q5R3F8	820	89686	7.67	Protein ph	ELFN2	ELFN2	Protein ph	5	163.1426	47.82011	1.75E-48	5	6.585366
Q15717	326	36091.6	9.57	ELAV-like	ELAVL1	ELAVL1	ELAV-like	11	5903.304	50.54879	2.53E-58	10	35.88957
Q9BQ52	826	92218.2	7.94	Zinc phosp	ELAC2	ELAC2	Zinc phosp	9	223.0621	50.23743	3.69E-57	9	14.04358
Q53HC9	387	43602.9	4.67	EARP and	EIPR1	EIPR1	EARP and	2	140.8493	47.18158	2.58E-46	2	6.459948
P56537	245	26598.8	4.31	Eukaryotic	EIF6	EIF6	Eukaryotic	7	1726.262	50.82028	2.38E-59	7	47.34694
O60841	1220	138825.9	5.15	Eukaryotic	EIF5B	EIF5B	Eukaryotic	27	1646.86	51.51603	5.18E-62	27	25.7377
P63241	154	16832.1	4.86	Eukaryotic	EIF5A	EIF5A	Eukaryotic	6	502.9238	49.20815	2.24E-53	2	57.79221
P55010	431	49222.4	5.26	Eukaryotic	EIF5	EIF5	Eukaryotic	5	203.9038	43.20997	4.46E-34	5	18.56148
Q15056	248	27384.8	7.37	Eukaryotic	EIF4H	EIF4H	Eukaryotic	2	158.033	41.89677	1.59E-30	2	14.51613
O43432	1585	176650.6	5.02	Eukaryotic	EIF4G3	EIF4G3	Eukaryotic	2	93.82314	40.24874	2.04E-26	2	1.198738
P78344	907	102361	7.14	Eukaryotic	EIF4G2	EIF4G2	Eukaryotic	6	292.9532	48.82616	5.25E-52	6	6.835722
Q04637	1599	175489.4	4.99	Eukaryotic	EIF4G1	EIF4G1	Eukaryotic	27	633.6	52.3395	3.35E-65	27	20.20013
O60573	245	28361.8	9.32	Eukaryotic	EIF4E2	EIF4E2	Eukaryotic	2	75.20251	34.66974	2.02E-15	2	9.795918
P06730	217	25097.1	6.04	Eukaryotic	EIF4E	EIF4E	Eukaryotic	1	502.6729	47.93071	7.31E-49	1	5.069124
P23588	611	69150.4	5.35	Eukaryotic	EIF4B	EIF4B	Eukaryotic	13	1689.091	50.58062	1.92E-58	13	22.25859
P38919	411	46870.6	6.69	Eukaryotic	EIF4A3	EIF4A3	Eukaryotic	17	7028.312	50.18082	6E-57	17	40.38929
Q14240	407	46401.9	5.13	Eukaryotic	EIF4A2	EIF4A2	Eukaryotic	4	351.3269	49.63559	6.19E-55	4	9.82801
P60842	406	46153.5	5.12	Eukaryotic	EIF4A1	EIF4A1	Eukaryotic	18	4155.219	50.3721	1.16E-57	10	45.81281
Q7L2H7	374	42502.5	5.32	Eukaryotic	EIF3M	EIF3M	Eukaryotic	11	2746.428	49.14016	3.94E-53	11	31.81818
Q9Y262	564	66726.5	6.29	Eukaryotic	EIF3L	EIF3L	Eukaryotic	22	4386.185	50.73692	4.92E-59	22	42.02128
Q9UBQ5	218	25059.4	4.55	Eukaryotic	EIF3K	EIF3K	Eukaryotic	7	3626.822	49.84186	1.09E-55	7	50.91743
O75822	258	29062.2	4.45	Eukaryotic	EIF3J	EIF3J	Eukaryotic	5	343.4717	50.24822	3.36E-57	5	20.54264
Q13347	325	36501.6	5.38	Eukaryotic	EIF3I	EIF3I	Eukaryotic	11	4144.795	50.97675	6.01E-60	11	46.46154
O15372	352	39930.1	6.52	Eukaryotic	EIF3H	EIF3H	Eukaryotic	16	2250.155	51.25233	5.36E-61	16	50.28409
O75821	320	35610.7	5.92	Eukaryotic	EIF3G	EIF3G	Eukaryotic	11	5378.781	52.88712	2.39E-67	11	42.1875
O00303	357	37563.5	5.12	Eukaryotic	EIF3F	EIF3F	Eukaryotic	9	3861.031	50.74702	4.5E-59	9	36.97479
P60228	445	52220.3	5.89	Eukaryotic	EIF3E	EIF3E	Eukaryotic	15	3581.948	53.21231	1.2E-68	15	36.17978
O15371	548	63972.3	5.85	Eukaryotic	EIF3D	EIF3D	Eukaryotic	17	4418.088	51.96173	1.01E-63	17	31.38686
B5ME19	914	105472.1	5.35	Eukaryotic	EIF3CL	EIF3CL;EIF3	Eukaryotic	30	6624.406	52.44229	1.32E-65	30	32.49453
P55884	814	92480.9	4.62	Eukaryotic	EIF3B	EIF3B	Eukaryotic	28	3716.85	54.1471	2.09E-72	28	41.15479
Q14152	1382	166567.7	6.73	Eukaryotic	EIF3A	EIF3A	Eukaryotic	55	4248.2	50.7371	4.91E-59	55	38.35022
P41091	472	51109.1	8.54	Eukaryotic	EIF2S3	EIF2S3	Eukaryotic	13	616.5939	53.55062	5.46E-70	1	35.80508
P20042	333	38388.1	5.46	Eukaryotic	EIF2S2	EIF2S2	Eukaryotic	7	509.866	47.98345	4.8E-49	7	27.32733
P05198	315	36111.8	4.73	Eukaryotic	EIF2S1	EIF2S1	Eukaryotic	10	443.4195	50.75396	4.24E-59	10	37.46032
Q13144	721	80379.2	4.72	Translatior	EIF2B5	EIF2B5	Translatior	4	293.9716	44.57705	4.89E-38	4	6.102635
Q9UI10	523	57556.7	9.87	Translatior	EIF2B4	EIF2B4	Translatior	4	368.9849	50.01117	2.56E-56	4	9.751434
Q9NR50	452	50239.8	6.41	Translatior	EIF2B3	EIF2B3	Translatior	2	57.46383	47.46103	2.95E-47	2	5.309735
Q14232	305	33711.8	7.4	Translatior	EIF2B1	EIF2B1	Translatior	2	50.17967	43.96936	3.04E-36	2	7.868852
Q9P2K8	1649	186908.9	6.22	eIF-2-alph	EIF2AK4	EIF2AK4	eIF-2-alph	7	237.7121	48.83008	5.09E-52	7	4.851425
P19525	551	62093.7	8.63	Interferon-	EIF2AK2	EIF2AK2	Interferon-	12	402.6118	51.76659	5.67E-63	12	26.1343
O60739	113	12823.6	7.51	Eukaryotic	EIF1B	EIF1B;EIF1	Eukaryotic	2	168.3448	43.80029	9.39E-36	2	26.54867
P47813	144	16460.3	4.84	Eukaryotic	EIF1AX	EIF1AX	Eukaryotic	4	220.8743	47.02692	8.5E-46	1	25
Q8N9N8	165	19053	4.89	Probable F	EIF1AD	EIF1AD	Probable F	1	198.9063	28.8072	1.18E-08	1	9.090909
Q96KQ7	1210	132369.2	5.14	Histone-ly	EHMT2	EHMT2	Histone-ly	8	694.6386	48.49762	7.72E-51	8	7.768595
Q9H9B1	1298	141464.9	5.53	Histone-ly	EHMT1	EHMT1	Histone-ly	10	237.6587	49.59619	8.64E-55	9	10.32357
Q08426	723	79494.2	9.54	Peroxisom	EHHADH	EHHADH	Peroxisom	2	129.8484	43.39706	1.33E-34	2	3.457815
Q9H223	541	61174.6	6.73	EH domair	EHD4	EHD4	EH domair	15	725.0328	53.53705	6.17E-70	13	30.31423
Q9NZN4	543	61160.9	6.42	EH domair	EHD2	EHD2	EH domair	3	189.3492	44.64401	3.08E-38	3	5.3407
Q9H4M9	534	60626.4	6.81	EH domair	EHD1	EHD1	EH domair	12	448.1397	48.6785	1.77E-51	6	26.21723
P18146	543	57506.2	8.37	Early grow	EGR1	EGR1	Early grow	1	78.04246	40.78556	1.03E-27	1	2.209945
P00533	1210	134276.2	6.67	Epidermal	EGFR	EGFR	Epidermal	7	294.8279	50.41701	7.89E-58	6	5.785124
Q9UHF1	273	29617.5	8.21	Epidermal	EGFL7	EGFL7	Epidermal	6	155.4235	49.73449	2.68E-55	6	23.07692
Q15029	972	109434.8	4.6	116 kDa U	EFTUD2	EFTUD2	116 kDa U	39	6468.993	51.70471	9.87E-63	39	47.11934
Q14156	821	92923.4	6.67	Protein EFI	EFR3A	EFR3A	Protein EFI	7	269.7421	48.46712	9.88E-51	7	10.71864
P98172	346	38006.3	9.23	Ephrin-B1	EFNB1	EFNB1	Ephrin-B1	1	24.92487	31.84814	1.46E-11	1	5.202312
Q96C19	240	26697	4.85	EF-hand d	EFHD2	EFHD2	EF-hand d	8	204.5421	53.72566	1.11E-70	6	38.33333
Q7L9B9	569	62402.1	8.57	Endonucle	EEPD1	EEPD1	Endonucle	1	257.7095	43.23373	3.83E-34	1	1.757469
P57772	596	65304.1	8.48	Selenocyst	EEFSEC	EEFSEC	Selenocyst	4	167.0647	51.6982	1.04E-62	4	8.053691
P13639	858	95337.4	6.82	Elongation	EEF2	EEF2	Elongation	33	7024.758	55.37146	1.94E-77	33	38.92774
P26641	437	50118.4	6.64	Elongation	EEF1G	EEF1G	Elongation	14	2815.992	51.66389	1.41E-62	14	25.85812
O43324	174	19810.4	8.84	Eukaryotic	EEF1E1	EEF1E1	Eukaryotic	5	481.2593	54.77165	5.75E-75	5	29.88506
P29692	281	31121.6	4.63	Elongation	EEF1D	EEF1D	Elongation	8	520.0862	48.20728	8.01E-50	8	37.72242
P24534	225	24763.5	4.26	Elongation	EEF1B2	EEF1B2	Elongation	3	249.433	46.55755	3.02E-44	2	14.66667
Q05639	463	50469.9	9.5	Elongation	EEF1A2	EEF1A2	Elongation	4	705.5453	47.39293	5.03E-47	4	12.527
P68104	462	50140.6	9.5	Elongation	EEF1A1	EEF1A1	Elongation	15	23951.48	49.99005	3.07E-56	1	47.4026
O75530	441	50197.2	7.03	Polycomb	EED	EED	Polycomb	14	1062.397	52.56088	4.56E-66	14	34.69388
Q6P2E9	1401	151659.6	5.69	Enhancer c	EDC4	EDC4	Enhancer c	45	5493.66	52.32028	3.99E-65	45	47.10921
Q96F86	508	56077.1	7.12	Enhancer c	EDC3	EDC3	Enhancer c	10	535.6383	48.89208	3.05E-52	10	26.37795
Q9H8V3	914	103503.9	7.77	Protein EC	ECT2	ECT2	Protein EC	2	270.4553	40.84735	7.28E-28	2	2.407002
Q16610	540	60673.6	6.7	Extracellul	ECM1	ECM1	Extracellul	1	78.27036	37.81326	4.58E-21	1	2.407407
O75521	394	43584.8	9.42	Enoyl-CoA	ECI2	ECI2	Enoyl-CoA	3	175.5385	47.78315	2.35E-48	3	9.137056
P30084	290	31387.1	8.19	Enoyl-CoA	ECHS1	ECHS1	Enoyl-CoA	1	126.4877	42.54099	3.08E-32	1	7.241379
Q9NTX5	307	33697.8	8.46	Ethylmalor	ECHDC1	ECHDC1	Ethylmalor	3	200.5547	48.30959	3.53E-50	3	11.40065
Q15125	230	26352.6	8.02	3-beta-hy	EBP	EBP	3-beta-hy	2	260.4707	45.34648	2.19E-40	2	11.30435
Q99848	306	34851.8	10.82	Probable r	EBNA1BP2	EBNA1BP2	Probable r	17	6389.249	49.12525	4.46E-53	17	47.38562
Q5JPH6	523	58688.1	8.98	Nondiscrin	EARS2	EARS2	Nondiscrin	2	60.78534	46.81992	4.15E-45	2	4.971319
Q56P03	285	32761.9	4.75	E2F-associ	EAPP	EAPP	E2F-associ	2	151.4504	51.17228	1.08E-60	2	8.77193
Q96JC9	268	29041.7	5.04	ELL-associ	EAF1	EAF1	ELL-associ	3	195.3779	47.92051	7.93E-49	3	10.8209
O75461	281	31843.7	5.16	Transcripti	E2F6	E2F6	Transcripti	2	143.6641	33.80168	4.1E-14	2	11.03203
Q16254	413	43959.9	4.38	Transcripti	E2F4	E2F4	Transcripti	3	91.93658	44.07361	1.51E-36	3	12.10654
Q14209	437	47505.4	4.48	Transcripti	E2F2	E2F2	Transcripti	1	37.52288	43.53325	5.47E-35	1	2.059497
Q13627	763	85583.4	8.98	Dual speci	DYRK1A	DYRK1A	Dual speci	13	992.2256	49.46853	2.54E-54	9	17.16907

Q9NP97	96	10921.5	7.54	Dynein ligl	DYNLRB1	DYNLRB1	Dynein ligl	2	92.13139	49.11952	4.67E-53	1	29.16667
P63167	89	10365.8	7.5	Dynein ligl	DYNLL1	DYNLL1;D'	Dynein ligl	2	327.0519	46.40561	9.44E-44	2	24.7191
Q8WVS4	1066	122569.7	7.32	Cytoplasm	DYNC2I1	DYNC2I1	Cytoplasm	2	77.51006	34.24527	9.08E-15	2	2.345216
O43237	492	54098.9	6.34	Cytoplasm	DYNC1LI2	DYNC1LI2	Cytoplasm	4	211.0396	48.40743	1.6E-50	4	13.41463
Q9Y6G9	523	56578.7	6.37	Cytoplasm	DYNC1LI1	DYNC1LI1	Cytoplasm	3	230.3586	43.61864	3.12E-35	3	8.413002
Q13409	638	71456.1	4.85	Cytoplasm	DYNC1I2	DYNC1I2	Cytoplasm	1	131.4766	49.30109	1.03E-53	1	2.194357
Q14204	4646	532403.4	6.34	Cytoplasm	DYNC1H1	DYNC1H1	Cytoplasm	39	460.0981	51.32656	2.78E-61	39	8.996987
Q7RTS9	669	75934.6	5.7	Dymeclin	DYM	DYM	Dymeclin	2	47.8893	47.5915	1.06E-47	2	2.690583
O14640	695	75186	7.89	Segment p	DVL1	DVL1;DVL1	Segment p	1	93.34124	52.03643	5.15E-64	1	2.158273
Q16690	384	42046.9	8.42	Dual speci	DUSP5	DUSP5	Dual speci	1	111.2343	44.5323	6.63E-38	1	3.385417
Q13115	394	42952.6	7.39	Dual speci	DUSP4	DUSP4	Dual speci	2	147.7628	50.72922	5.26E-59	2	6.091371
Q9BVJ7	150	16588	8.34	Dual speci	DUSP23	DUSP23	Dual speci	4	192.4506	47.72813	3.62E-48	4	30
Q9UNI6	340	37687	6.84	Dual speci	DUSP12	DUSP12	Dual speci	3	227.1093	48.58007	3.95E-51	3	11.17647
O75319	330	38938.6	9.8	RNA/RNP	DUSP11	DUSP11	RNA/RNP	3	123.1854	51.67687	1.25E-62	3	11.81818
Q96G46	650	72593.3	8.11	tRNA-dihy	DUS3L	DUS3L	tRNA-dihy	3	75.20066	47.25015	1.52E-46	3	5.230769
P23919	212	23819.1	8.49	Thymidyla	DTYMK	DTYMK	Thymidyla	4	129.1758	45.19689	6.36E-40	4	18.39623
Q8TDB6	740	83553.7	8.15	E3 ubiquiti	DTX3L	DTX3L	E3 ubiquiti	5	135.8407	45.32372	2.58E-40	5	8.108108
Q86UW9	622	67245.8	8.55	Probable E	DTX2	DTX2	Probable E	1	34.96719	28.92294	9.7E-09	1	2.250804
O60941	627	71355	7.98	Dystrobrev	DTNB	DTNB	Dystrobrev	6	407.3125	39.17802	5.86E-24	3	11.00478
P60981	165	18505.5	7.97	Destrin	DSTN	DSTN	Destrin	8	973.0898	46.86274	3E-45	8	48.48485
Q03001	7570	860654.9	4.89	Dystonin	DST	DST	Dystonin	3	140.3613	47.11604	4.29E-46	3	0.488771
P15924	2871	331771.2	6.78	Desmoplal	DSP	DSP	Desmoplal	35	1317.365	52.151	1.84E-64	35	13.68861
Q9H410	356	40066.9	7.05	Kinetochor	DSN1	DSN1	Kinetochor	1	57.90592	27.44107	9.44E-08	1	3.089888
Q14126	1118	122293	4.89	Desmoglei	DSG2	DSG2	Desmoglei	22	2605.659	54.35483	3.13E-73	22	26.83363
Q02413	1049	113746.7	4.66	Desmoglei	DSG1	DSG1	Desmoglei	5	167.4606	45.14211	9.39E-40	5	6.101049
Q08554	894	99986.1	5.08	Desmocoll	DSC1	DSC1	Desmocoll	5	199.021	48.66642	1.95E-51	5	7.270694
Q9NRR4	1374	159314.7	7.91	Ribonuclea	DROSHA	DROSHA	Ribonuclea	11	181.0336	54.19932	1.27E-72	11	9.534207
P55039	364	40746.1	9.2	Developme	DRG2	DRG2	Developme	1	87.14536	22.13011	5.1E-06	1	3.021978
Q9Y295	367	40541.8	9.31	Developme	DRG1	DRG1	Developme	10	332.3957	54.15014	2.04E-72	10	32.97003
Q14919	205	22349.6	4.8	Dr1-associ	DRAP1	DRAP1	Dr1-associ	2	454.7205	46.91911	1.94E-45	2	10.73171
Q01658	176	19443.5	4.4	Protein Dr	DR1	DR1	Protein Dr	2	228.4358	48.50405	7.33E-51	2	15.34091
Q14195	570	61962.8	6.45	Dihydropy	DPYSL3	DPYSL3	Dihydropy	1	71.1538	41.75888	3.64E-30	1	1.929825
Q16555	572	62293.1	6.33	Dihydropy	DPYSL2	DPYSL2	Dihydropy	13	349.3664	59.25273	1.47E-93	11	36.01399
Q9C005	99	11249.6	4.55	Protein dp	DPY30	DPY30	Protein dp	2	314.2596	40.61419	2.71E-27	2	25.25253
Q2PZI1	675	77317.8	9.18	Protein C-	DPY19L1	DPY19L1	Protein C-	1	222.5211	35.15181	3.4E-16	1	1.333333
Q9P2X0	92	10093.6	5.75	Dolichol-p	DPM3	DPM3	Dolichol-p	1	60.12199	28.30921	2.69E-08	1	10.86957
O60762	260	29634	10.02	Dolichol-p	DPM1	DPM1	Dolichol-p	7	924.705	49.47038	2.5E-54	7	31.92308
Q92784	378	43083.8	6.39	Zinc finger	DPF3	DPF3	Zinc finger	1	76.75874	44.53052	6.71E-38	1	3.703704
Q92785	391	44155	6.25	Zinc finger	DPF2	DPF2	Zinc finger	7	322.9554	47.0614	6.52E-46	7	22.76215
Q9BZ29	2069	236443.6	7.51	Dedicator	DOCK9	DOCK9	Dedicator	3	46.86884	37.68881	8.16E-21	3	1.836636
Q96N67	2140	242558.3	6.78	Dedicator	DOCK7	DOCK7	Dedicator	39	624.5361	51.64758	1.63E-62	36	20.51402
Q96HP0	2047	229555.6	6.72	Dedicator	DOCK6	DOCK6	Dedicator	13	246.6761	55.5012	5.87E-78	13	7.523205
Q5QJE6	756	84468.3	6.02	Deoxynucl	DNTTIP2	DNTTIP2	Deoxynucl	10	726.5514	50.75734	4.12E-59	10	18.78307
Q9H147	329	37013	9.37	Deoxynucl	DNTTIP1	DNTTIP1	Deoxynucl	1	211.2564	49.67176	4.56E-55	1	3.647416
Q9ULA0	485	53410	7.62	Aspartyl ar	DNPEP	DNPEP	Aspartyl ar	6	283.4619	47.3384	7.7E-47	6	16.08247
Q9UBC3	853	95750.2	8.52	DNA (cytos	DNMT3B	DNMT3B	DNA (cytos	4	117.936	48.56017	4.65E-51	4	6.096131
Q9Y6K1	912	101857.6	6.52	DNA (cytos	DNMT3A	DNMT3A	DNA (cytos	14	179.7819	47.54328	1.55E-47	13	16.33772
P26358	1616	183163.6	7.81	DNA (cytos	DNMT1	DNMT1	DNA (cytos	7	236.7457	47.56813	1.28E-47	7	4.331683
Q6XZF7	1577	177345.5	5.06	Dynamin-I	DNMBP	DNMBP	Dynamin-I	9	487.75	47.07309	5.96E-46	9	6.848446
P50570	870	98063.3	7.5	Dynamin-2	DNM2	DNM2	Dynamin-2	14	602.6425	52.08914	3.21E-64	11	18.3908
O00429	736	81876.4	6.8	Dynamin-1	DNM1L	DNM1L	Dynamin-1	8	126.5037	51.17054	1.1E-60	8	12.5
O00115	360	39580.6	8.14	Deoxyribor	DNASE2	DNASE2	Deoxyribor	2	86.55437	45.07407	1.52E-39	2	7.222222
Q8WXX5	260	29909.5	5.38	DnaJ homo	DNAJC9	DNAJC9	DnaJ homo	11	1588.994	51.31445	3.09E-61	11	40.38462
O75937	253	29841.5	9.71	DnaJ homo	DNAJC8	DNAJC8	DnaJ homo	2	159.0131	44.98251	2.89E-39	2	9.486166
Q99615	494	56440.3	6.95	DnaJ homo	DNAJC7	DNAJC7	DnaJ homo	5	191.5943	53.26372	7.54E-69	5	11.33603
Q13217	504	57579.6	6.04	DnaJ homo	DNAJC3	DNAJC3	DnaJ homo	1	38.66252	38.93002	2.06E-23	1	2.380952
Q5F1R6	531	62027.5	5.13	DnaJ homo	DNAJC21	DNAJC21	DnaJ homo	10	1058.97	50.8015	2.8E-59	10	24.67043
Q99543	621	71995.8	9.13	DnaJ homo	DNAJC2	DNAJC2	DnaJ homo	3	83.86845	41.28372	5.96E-29	3	6.924316
Q96DA6	116	12498.5	10.75	Mitochonc	DNAJC19	DNAJC19	Mitochonc	3	163.7417	38.2537	5.72E-22	3	26.72414
Q9NVM6	304	34687.1	8.95	DnaJ homo	DNAJC17	DNAJC17	DnaJ homo	4	171.104	50.22824	3.99E-57	4	18.09211
Q9Y2G8	782	90590.5	7.13	DnaJ homo	DNAJC16	DNAJC16	DnaJ homo	8	237.5774	52.72025	1.09E-66	8	9.71867
O75165	2243	254412.5	6.73	DnaJ homo	DNAJC13	DNAJC13	DnaJ homo	4	75.24731	43.40928	1.23E-34	4	2.273741
Q9NVH1	559	63277.6	8.6	DnaJ homo	DNAJC11	DNAJC11	DnaJ homo	8	281.9027	51.21822	7.21E-61	8	19.14132
Q8IXB1	793	91078.9	7.19	DnaJ homo	DNAJC10	DNAJC10	DnaJ homo	6	177.2307	53.49812	8.78E-70	6	7.818411
Q96KC8	554	63882.3	8.97	DnaJ homo	DNAJC1	DNAJC1	DnaJ homo	2	73.6919	39.71315	3.63E-25	2	5.054152
O75190	326	36086.8	9.74	DnaJ homo	DNAJB6	DNAJB6	DnaJ homo	7	1010.432	52.67933	1.58E-66	4	25.46012
O75953	348	39133.1	9.48	DnaJ homo	DNAJB5	DNAJB5	DnaJ homo	1	31.02622	37.50968	1.86E-20	1	4.022989
Q9UDY4	337	37806.5	8.78	DnaJ homo	DNAJB4	DNAJB4	DnaJ homo	3	148.8801	47.69648	4.65E-48	3	9.495549
P25686	324	35579.8	5.76	DnaJ homo	DNAJB2	DNAJB2	DnaJ homo	2	156.9747	36.80101	4.31E-19	2	7.407407
Q8TBM8	379	42515.4	8.79	DnaJ homo	DNAJB14	DNAJB14	DnaJ homo	2	152.3015	43.84758	6.86E-36	2	5.540897
Q9NXW2	375	41859.5	8.93	DnaJ homo	DNAJB12	DNAJB12	DnaJ homo	4	256.9374	51.25153	5.39E-61	4	12.26667
Q9UBS4	358	40513.7	6.08	DnaJ homo	DNAJB11	DNAJB11	DnaJ homo	4	577.4548	50.59633	1.67E-58	4	16.75978
P25685	340	38043.8	8.98	DnaJ homo	DNAJB1	DNAJB1	DnaJ homo	9	751.1006	48.58855	3.69E-51	8	27.35294
Q8WW22	397	44797.5	7.65	DnaJ homo	DNAJA4	DNAJA4	DnaJ homo	2	47.44574	36.85074	3.47E-19	2	5.541562
Q96EY1	480	52488.3	9.68	DnaJ homo	DNAJA3	DNAJA3	DnaJ homo	9	1158.714	52.27348	6.06E-65	9	23.95833
O60884	412	45745.4	6.44	DnaJ homo	DNAJA2	DNAJA2	DnaJ homo	7	760.8239	49.78571	1.75E-55	7	19.17476
P31689	397	44868	7.09	DnaJ homo	DNAJA1	DNAJA1	DnaJ homo	10	758.1544	50.43877	6.53E-58	10	28.21159
Q86Y56	855	93519.9	6.39	Dynein axo	DNAAF5	DNAAF5	Dynein axo	2	116.0231	40.21938	2.39E-26	2	2.339181
Q9NPF5	467	52992.1	10.09	DNA meth	DMAP1	DMAP1	DNA meth	4	235.4203	43.84526	6.96E-36	4	10.70664
Q9NW81	257	29267.2	6.39	Distal men	DMAC2	DMAC2	Distal men	2	49.47829	40.57548	3.35E-27	2	10.11673
P36957	453	48754.9	9.39	Dihydrolip	DLST	DLST	Dihydrolip	3	624.0854	48.85445	4.17E-52	3	7.284768
Q8TDM6	1919	213865.6	7.44	Disks large	DLG5	DLG5	Disks large	4	241.8919	45.68718	1.88E-41	4	2.344971
Q92796	817	90313.3	7.03	Disks large	DLG3	DLG3	Disks large	5	197.3939	48.65109	2.21E-51	5	7.588739
Q12959	904	100454.2	5.51	Disks large	DLG1	DLG1	Disks large	14	332.5609	51.34885	2.29E-61	11	19.13717
P09622	509	54176.9	7.95	Dihydrolip	DLD	DLD	Dihydrolip	3	152.7724	50.47149	4.94E-58	3	8.447937
O94907	266	28671.3	8.45	Dickkopf-r	DKK1	DKK1	Dickkopf-r	4	329.7188	52.81812	4.53E-67	4	15.41353
O60832	514	57673.7	10.02	H/ACA ribo	DKC1	DKC1	H/ACA ribo	24	2958.742	51.99444	7.52E-64	24	43.38521
Q9Y2L1	958	109001.7	7.14	Exosome c	DIS3	DIS3	Exosome c	10	401.5145	49.40217	4.43E-54	10	11.27349
Q8NDZ4	430	49481.3	8.64	Divergent	DIPK2A	DIPK2A	Divergent	2	58.07589	28.70365	1.41E-08	2	8.139535
Q5VUD6	431	48582.1	8.77	Divergent	DIPK1B	DIPK1B	Divergent	1	2158.629	29.5029	3.32E-09	1	2.320186
Q9Y2E4	1556	170765.9	7.39	Disco-inte	DIP2C	DIP2C	Disco-inte	8	85.83168	43.47938	7.77E-35	6	4.948586
Q9UNQ2	313	35236.1	10.71	Dimethyla	DIMT1	DIMT1	Dimethyla	8	262.5199	55.13629	1.75E-76	8	32.58786
Q9BTC0	2240	243870.3	7.96	Death-ind	DIDO1	DIDO1	Death-ind	21	571.6509	47.87465	1.14E-48	21	11.5625

Q9UPY3	1922	218679.3	5.4	Endoribon	DICER1	DICER1	Endoribon	15	286.659	47.57604	1.2E-47	15	9.833507
Q9NR28	239	27130.6	5.72	Diablo IAP	DIABLO	DIABLO	Diablo IAP	3	105.8187	46.83389	3.74E-45	3	13.80753
Q08211	1270	140957.5	6.83	ATP-deper	DHX9	DHX9	ATP-deper	49	20207.75	54.26129	7.28E-73	49	42.75591
Q14562	1220	139313.3	8.5	ATP-deper	DHX8	DHX8	ATP-deper	22	631.2262	52.82464	4.27E-67	20	23.52459
Q6P158	1386	155603	7.76	Putative A	DHX57	DHX57	Putative A	35	1695.451	52.89306	2.27E-67	35	31.52958
Q8IX18	779	88559.3	8.79	Probable A	DHX40	DHX40	Probable A	7	167.5287	45.97706	2.27E-42	7	11.16816
Q92620	1227	140501.6	6.5	Pre-mRNA	DHX38	DHX38	Pre-mRNA	28	902.7576	52.91276	1.89E-67	28	27.30236
Q8IY37	1157	129544.3	8.21	Probable A	DHX37	DHX37	Probable A	16	632.4895	49.28011	1.23E-53	16	15.81677
Q9H2U1	1008	114759.3	7.73	ATP-deper	DHX36	DHX36	ATP-deper	24	931.9381	53.00724	7.96E-68	24	28.1746
Q9H5Z1	703	78909.7	8.76	Probable A	DHX35	DHX35	Probable A	7	351.2933	52.17436	1.49E-64	7	11.23755
Q14147	1143	128118.4	7.59	Probable A	DHX34	DHX34	Probable A	2	91.94707	46.83683	3.65E-45	2	2.362205
Q7L7V1	743	84417.8	4.62	Putative pr	DHX32	DHX32	Putative pr	10	321.8884	55.48005	7.05E-78	10	14.1319
Q7L2E3	1194	133936.9	9.02	ATP-deper	DHX30	DHX30	ATP-deper	58	14274.63	53.58762	3.95E-70	58	56.36516
Q7Z478	1369	155234.3	8.22	ATP-deper	DHX29	DHX29	ATP-deper	20	592.3414	50.40932	8.43E-58	19	18.55369
O60231	1041	119262.5	6.77	Pre-mRNA	DHX16	DHX16	Pre-mRNA	13	317.5926	54.5821	3.54E-74	13	14.69741
O43143	795	90932	7.48	ATP-deper	DHX15	DHX15	ATP-deper	39	25989.54	52.88273	2.49E-67	39	47.92453
Q6IAN0	325	35118.7	10.03	Dehydroge	DHRS7B	DHRS7B	Dehydroge	2	65.01241	43.06208	1.15E-33	2	8.923077
Q9Y394	339	38298.4	8.47	Dehydroge	DHRS7	DHRS7	Dehydroge	2	102.7911	50.20813	4.75E-57	2	7.374631
P0CG22	281	30607.4	10.47	Putative de	DHRS4L1	DHRS4L1;D	Putative de	1	437.5831	42.99076	1.81E-33	1	3.202847
Q13268	280	29926.3	9.28	Dehydroge	DHRS2	DHRS2	Dehydroge	6	416.3025	47.28152	1.19E-46	6	28.92857
Q02127	395	42866.9	10.23	Dihydroor	DHODH	DHODH	Dihydroor	4	245.3188	42.93025	2.66E-33	4	12.91139
Q9UBM7	475	54489	8.82	7-dehydrc	DHCR7	DHCR7	7-dehydrc	4	363.2706	47.75065	3.04E-48	4	8.842105
Q15392	516	60100.8	8.24	Delta(24)-s	DHCR24	DHCR24	Delta(24)-s	7	477.1677	49.76427	2.09E-55	7	13.75969
Q7Z3D6	616	66436	6.78	D-glutama	DGLUCY	DGLUCY	D-glutama	1	25.66311	28.14263	3.48E-08	1	3.084416
Q13574	928	103980.1	8.1	Diacylglyce	DGKZ	DGKZ	Diacylglyce	2	175.5078	45.20388	6.05E-40	2	2.801724
P52824	942	101154	7.5	Diacylglyce	DGKQ	DGKQ	Diacylglyce	2	83.3578	46.60307	2.15E-44	2	2.441614
P23743	735	82629.6	6.71	Diacylglyce	DGKA	DGKA	Diacylglyce	5	168.796	39.48156	1.22E-24	5	10.88435
Q8WYQ5	773	86044.4	5.74	Microproc	DGCR8	DGCR8	Microproc	8	460.9362	49.61448	7.41E-55	8	13.84217
Q9BUN8	251	28800.6	9.67	Derlin-1	DERL1	DERL1	Derlin-1	1	739.1216	52.60306	3.12E-66	1	4.38247
Q9Y315	318	35230.4	9.31	Deoxyribos	DERA	DERA	Deoxyribos	1	86.58534	39.27002	3.66E-24	1	3.773585
Q8TB45	409	46293.5	8.17	DEP doma	DEPTOR	DEPTOR	DEP doma	2	184.1035	34.85242	1.04E-15	2	4.645477
O43583	198	22091.8	4.93	Density-re	DENR	DENR	Density-re	3	110.3578	47.33168	8.1E-47	3	21.21212
P35659	375	42673.9	9.01	Protein DE	DEK	DEK	Protein DE	8	1454.443	51.381	1.73E-61	8	23.73333
Q9H4E7	631	73909.8	5.89	Differentia	DEF6	DEF6	Differentia	4	189.3614	49.72213	2.98E-55	4	6.656101
O75618	318	36793.9	9.05	Death effe	DEDD	DEDD	Death effe	2	62.36338	33.94395	2.55E-14	1	5.660377
Q9NUI1	292	30777.2	9.59	Peroxisom	DECR2	DECR2	Peroxisom	2	194.9473	50.87191	1.52E-59	2	10.27397
Q16698	335	36067.4	9.84	2,4-dienoy	DECR1	DECR1	2,4-dienoy	3	106.7516	48.13962	1.38E-49	3	8.955224
Q5H9U9	1706	197611.8	8.38	Probable A	DDX60L	DDX60L	Probable A	9	155.3086	55.20946	8.76E-77	9	5.509965
Q8IY21	1712	197851.4	7.64	Probable A	DDX60	DDX60	Probable A	25	496.6169	55.4469	9.71E-78	25	17.11449
P26196	483	54416.4	8.93	Probable A	DDX6	DDX6	Probable A	13	928.5679	52.80616	5.05E-67	13	33.54037
Q9NY93	547	61588.9	9.76	Probable A	DDX56	DDX56	Probable A	8	495.4741	51.70741	9.63E-63	8	13.71115
Q8NHQ9	600	68546.3	9.84	ATP-deper	DDX55	DDX55	ATP-deper	13	533.8035	50.86112	1.66E-59	13	22
Q8TDD1	881	98594	10.7	ATP-deper	DDX54	DDX54	ATP-deper	26	2578.008	51.60594	2.35E-62	26	33.48468
Q9Y2R4	599	67465.5	10.37	Probable A	DDX52	DDX52	Probable A	6	590.0681	50.24978	3.32E-57	6	14.69115
Q9BQ39	737	82564.3	9.68	ATP-deper	DDX50	DDX50	ATP-deper	20	576.3219	52.0948	3.06E-64	20	35.27815
P17844	614	69147.6	9.21	Probable A	DDX5	DDX5	Probable A	32	53177.26	51.30008	3.52E-61	25	48.20847
Q9Y6V7	483	54225.8	9.49	Probable A	DDX49	DDX49	Probable A	3	386.3062	47.42559	3.9E-47	3	9.109731
Q9H0S4	455	50646.1	9.63	Probable A	DDX47	DDX47	Probable A	16	2053.437	52.50368	7.61E-66	16	45.93407
Q7L014	1031	117361.1	9.87	Probable A	DDX46	DDX46	Probable A	43	6071.263	54.11655	2.78E-72	43	43.06499
Q86XP3	938	102974.5	7.01	ATP-deper	DDX42	DDX42	ATP-deper	21	916.3062	52.7996	5.36E-67	21	29.53092
Q9UJV9	622	69837	6.83	Probable A	DDX41	DDX41	Probable A	29	2273.043	51.06079	2.87E-60	29	47.7492
O00571	662	73242.8	7.2	ATP-deper	DDX3X	DDX3X	ATP-deper	39	28901.64	51.60145	2.45E-62	14	58.76133
Q13838	428	48990.9	5.38	Spliceoson	DDX39B	DDX39B	Spliceoson	4	56.4575	51.3446	2.37E-61	4	10.98131
O00148	427	49129.2	5.39	ATP-deper	DDX39A	DDX39A	ATP-deper	7	2435.123	49.00275	1.22E-52	2	15.69087
Q9H8H2	851	94086.3	10.68	ATP-deper	DDX31	DDX31	ATP-deper	19	1121.111	51.06176	2.85E-60	19	26.6745
Q9NUL7	540	59580.6	11.06	Probable A	DDX28	DDX28	Probable A	12	454.3999	51.99303	7.61E-64	12	28.51852
Q96GQ7	796	89834.5	9.94	Probable A	DDX27	DDX27	Probable A	22	2057.451	57.45594	5.67E-86	22	27.88945
Q9GZR7	859	96331	9.62	ATP-deper	DDX24	DDX24	ATP-deper	27	2509.746	52.47063	1.02E-65	27	35.38999
Q9BUQ8	820	95581.6	10.21	Probable A	DDX23	DDX23	Probable A	27	3660.04	53.93461	1.55E-71	27	35.60976
Q9NR30	783	87343.9	9.92	Nucleolar I	DDX21	DDX21	Nucleolar I	29	4166.171	53.94111	1.46E-71	27	39.59132
Q9UHI6	824	92239.7	6.95	Probable A	DDX20	DDX20	Probable A	7	1158.079	47.03833	7.79E-46	7	12.01456
Q9NUU7	478	53974.6	6.53	ATP-deper	DDX19A	DDX19A;D	ATP-deper	9	345.8251	52.43881	1.37E-65	9	24.26778
Q9NVP1	670	75406.3	10.13	ATP-deper	DDX18	DDX18	ATP-deper	17	1734.455	49.54781	1.3E-54	17	30.44776
Q92841	729	80271.8	8.37	Probable A	DDX17	DDX17	Probable A	26	7438.56	51.96666	9.64E-64	26	35.93964
Q13206	875	100887.2	9.08	Probable A	DDX10	DDX10	Probable A	27	2542.794	51.90617	1.66E-63	27	38.85714
Q92499	740	82431.7	7.23	ATP-deper	DDX1	DDX1	ATP-deper	32	4772.529	52.71917	1.1E-66	32	51.08108
P39656	456	50800.3	6.52	Dolichyl-d	DDOST	DDOST	Dolichyl-d	10	2411.932	52.35278	2.97E-65	10	27.19298
Q92466	427	47863.5	9.98	DNA dama	DDB2	DDB2	DNA dama	13	5914.615	49.70906	3.33E-55	13	40.51522
Q16531	1140	126966.9	4.92	DNA dama	DDB1	DDB1	DNA dama	46	9738.896	52.08595	3.29E-64	46	44.38596
O95865	285	29643.5	5.9	Putative hy	DDAH2	DDAH2	Putative hy	1	31.00119	42.83833	4.77E-33	1	5.614035
Q9BW61	102	11835.2	9.05	DET1- and	DDA1	DDA1	DET1- and	1	115.9759	35.24717	2.37E-16	1	11.76471
Q7Z4W1	244	25912.9	8.23	L-xylulose	DCXR	DCXR	L-xylulose	6	386.4569	48.16279	1.14E-49	6	31.55738
Q9BTE7	237	27508.2	5.23	DCN1-like	DCUN1D5	DCUN1D5	DCN1-like	2	80.90003	42.05371	6.16E-31	2	8.860759
Q8IWE4	304	34291	4.77	DCN1-like	DCUN1D3	DCUN1D3	DCN1-like	7	470.5505	52.31157	4.31E-65	7	27.30263
Q9H773	170	18680.6	4.67	dCTP pyro	DCTPP1	DCTPP1	dCTP pyro	6	680.0568	53.16344	1.89E-68	6	34.70588
Q9UJW0	460	52336.6	7.37	Dynactin s	DCTN4	DCTN4	Dynactin s	5	72.52692	48.63766	2.47E-51	5	13.26087
Q13561	401	44230.7	4.86	Dynactin s	DCTN2	DCTN2	Dynactin s	3	67.27318	41.59177	9.81E-30	3	7.481297
Q14203	1278	141693.5	5.58	Dynactin s	DCTN1	DCTN1	Dynactin s	2	112.8787	35.92813	1.65E-17	2	1.877934
Q96C86	337	38608.4	6.35	m7GpppX	DCPS	DCPS	m7GpppX	2	149.2631	39.95922	9.76E-26	2	6.231454
Q8IU60	420	48422.7	8.1	m7GpppN	DCP2	DCP2	m7GpppN	4	306.3214	48.85288	4.22E-52	4	11.19048
Q8IZD4	617	67722.4	8.77	mRNA-dec	DCP1B	DCP1B	mRNA-dec	1	26.36574	35.63295	5.33E-17	1	2.593193
Q9NPI6	582	63277.9	6.2	mRNA-dec	DCP1A	DCP1A	mRNA-dec	5	139.5017	53.09931	3.42E-68	5	13.23024
P81605	110	11283.7	6.52	Dermcidin	DCD	DCD	Dermcidin	2	934.769	46.79631	4.96E-45	2	20
Q96PD2	775	85034	7.18	Discoidin,	DCBLD2	DCBLD2	Discoidin,	1	110.8087	28.81385	1.17E-08	1	1.290323
Q5TAQ9	597	66851.7	5.05	DDB1- and	DCAF8	DCAF8	DDB1- and	5	140.0649	43.58785	3.82E-35	5	9.21273
P61962	342	38925.8	5.21	DDB1- and	DCAF7	DCAF7	DDB1- and	11	2349.637	49.75156	2.32E-55	11	35.67251
Q96JK2	942	103962.2	5.53	DDB1- and	DCAF5	DCAF5	DDB1- and	2	212.3647	50.20788	4.76E-57	2	1.910828
Q8WV16	495	55693	9.55	DDB1- and	DCAF4	DCAF4	DDB1- and	2	131.21	37.93719	2.57E-21	1	4.444444
Q9NV06	445	51401.8	9.62	DDB1- and	DCAF13	DCAF13	DDB1- and	4	184.4495	52.49959	7.87E-66	4	11.23596
Q8TEB1	546	61669	6.35	DDB1- and	DCAF11	DCAF11	DDB1- and	1	132.8208	39.59774	6.66E-25	1	2.380952
Q5QP82	559	60581.4	7.53	DDB1- and	DCAF10	DCAF10	DDB1- and	1	236.4433	44.59022	4.46E-38	1	1.9678
Q9Y4B6	1507	169005.5	4.68	DDB1- and	DCAF1	DCAF1	DDB1- and	12	282.2296	53.69658	1.45E-70	12	9.223623
P11182	482	53516.7	8.75	Lipoamide	DBT	DBT	Lipoamide	4	53.18494	44.30775	3.08E-37	4	13.27801

Q9UK59	544	61554.4	5.14	Lariat debr	DBR1	DBR1	Lariat debr	3	171.1623	46.31294	1.89E-43	3	9.191176
Q9UJU6	430	48207	4.72	Drebrin-lik	DBNL	DBNL	Drebrin-lik	3	75.66019	37.9338	2.61E-21	3	9.302326
Q16643	649	71428.6	4.1	Drebrin	DBN1	DBN1	Drebrin	16	3295.995	48.9933	1.32E-52	16	23.57473
Q96EP5	407	43383.3	8.78	DAZ-assoc	DAZAP1	DAZAP1	DAZ-assoc	3	135.426	52.31549	4.15E-65	3	12.77641
Q9UER7	740	81371.7	4.51	Death dom	DAXX	DAXX	Death dom	4	917.0318	46.92307	1.89E-45	4	7.162162
Q6PI48	645	73562	8.11	Aspartate-	DARS2	DARS2	Aspartate-	3	98.09888	42.02143	7.5E-31	3	4.496124
P14868	501	57135.8	6.52	Aspartate-	DARS1	DARS1	Aspartate-	21	965.4418	56.10917	1.49E-80	21	45.10978
P51398	398	45566.1	9.22	Small ribos	DAP3	DAP3	Small ribos	21	12253.7	49.14768	3.7E-53	21	52.01005
P61803	113	12496.5	7.17	Dolichyl-d	DAD1	DAD1	Dolichyl-d	2	835.6958	53.61494	3.08E-70	2	19.46903
Q5VWQ8	1189	131623.9	8.97	Disabled h	DAB2IP	DAB2IP	Disabled h	3	493.2007	38.361	3.41E-22	2	3.027754
Q9Y4D1	1078	123472.4	7.24	Dishevelec	DAAM1	DAAM1	Dishevelec	1	62.78568	20.83151	8.46E-06	1	0.834879
O43739	400	46348.2	5.13	Cytohesin-	CYTH3	CYTH3;CY	Cytohesin-	1	156.6702	45.41441	1.35E-40	1	2.75
Q9H1C7	97	10631	3.96	Cysteine-r	CYSTM1	CYSTM1	Cysteine-r	1	201.8779	31.12528	9.39E-11	1	10.30928
A8MQ03	144	15313.3	7.08	Cysteine-r	CYSRT1	CYSRT1	Cysteine-r	1	133.1558	41.14783	1.31E-28	1	6.944444
Q9NUQ9	324	36747.7	5.94	CYFIP-rela	CYRIB	CYRIB	CYFIP-rela	7	245.5275	54.12322	2.61E-72	5	25.30864
Q16850	509	57277.8	8.72	Lanosterol	CYP51A1	CYP51A1	Lanosterol	4	122.1908	47.21055	2.07E-46	4	9.430255
Q86W10	505	59085.5	9.63	Cytochrom	CYP4Z1	CYP4Z1	Cytochrom	1	169.4873	28.72251	1.37E-08	1	1.980198
Q9HBI6	524	60145.2	6.71	Cytochrom	CYP4F11	CYP4F11	Cytochrom	3	65.36798	37.66159	9.25E-21	1	6.870229
P13584	511	58990.6	8.39	Cytochrom	CYP4B1	CYP4B1	Cytochrom	1	16.72184	24.697	1.35E-06	1	1.761252
Q07973	514	58874.7	8.99	1,25-dihyc	CYP24A1	CYP24A1	1,25-dihyc	2	142.0355	50.7289	5.27E-59	2	4.474708
Q96F07	1278	148397.2	7.33	Cytoplasm	CYFIP2	CYFIP2	Cytoplasm	3	77.54946	48.62915	2.65E-51	3	3.208138
Q7L576	1253	145181.2	6.9	Cytoplasm	CYFIP1	CYFIP1	Cytoplasm	11	378.5032	51.01429	4.33E-60	7	10.13567
P99999	105	11748.7	10.16	Cytochrom	CYCS	CYCS	Cytochrom	1	382.111	42.62311	1.85E-32	1	10.47619
P08574	325	35421.6	9.25	Cytochrom	CYC1	CYC1	Cytochrom	7	274.4739	53.39463	2.28E-69	7	23.07692
Q53TN4	286	31641	9.02	Plasma me	CYBRD1	CYBRD1	Plasma me	1	502.5627	50.45753	5.56E-58	1	3.496503
Q9BQA9	187	20773.6	6.79	Cytochrom	CYBC1	CYBC1	Cytochrom	1	128.0253	46.09846	9.3E-43	1	7.486631
P13498	195	21012.4	9.96	Cytochrom	CYBA	CYBA	Cytochrom	2	328.278	42.56857	2.59E-32	2	10.25641
P00387	301	34234.5	7.68	NADH-cyt	CYB5R3	CYB5R3	NADH-cyt	12	2267.51	50.88694	1.33E-59	12	44.18605
Q9UHQ9	305	34094.6	9.82	NADH-cyt	CYB5R1	CYB5R1	NADH-cyt	2	124.8631	50.62217	1.33E-58	2	5.901639
O43169	150	16694.4	4.61	Cytochrom	CYB5B	CYB5B	Cytochrom	4	2114.882	51.47088	7.74E-62	4	36.66667
P00167	134	15330	4.61	Cytochrom	CYB5A	CYB5A	Cytochrom	2	240.634	51.93036	1.34E-63	2	26.1194
Q9P0U4	656	75710.9	8.29	CXXC-type	CXXC1	CXXC1	CXXC-type	11	541.8721	50.79311	3.01E-59	11	17.37805
P78310	365	40029.5	7.63	Coxsackiev	CXADR	CXADR	Coxsackiev	5	236.7366	49.54525	1.33E-54	5	17.53425
Q69YN2	538	60618.6	7.26	CWF19-lik	CWF19L1	CWF19L1	CWF19-lik	9	450.4171	44.30827	3.07E-37	9	18.77323
Q6UX04	472	53846.7	5.53	Spliceoson	CWC27	CWC27	Spliceoson	4	562.2131	50.77583	3.5E-59	4	12.5
Q9NXE8	425	49647.2	10.9	Pre-mRNA	CWC25	CWC25	Pre-mRNA	2	245.4998	52.72265	1.07E-66	2	5.647059
Q9HCG8	908	105465.2	7.03	Pre-mRNA	CWC22	CWC22	Pre-mRNA	25	554.6952	51.94909	1.13E-63	25	28.4141
Q9P013	229	26624.1	5.38	Spliceoson	CWC15	CWC15	Spliceoson	2	455.2992	44.13849	9.72E-37	2	8.296943
Q96C57	262	28170.2	10.2	Protein CU	CUSTOS	CUSTOS	Protein CU	1	55.59575	46.80193	4.76E-45	1	6.10687
Q93034	780	90954.6	8.09	Cullin-5	CUL5	CUL5	Cullin-5	4	209.6521	50.20975	4.69E-57	4	4.74359
Q13620	913	103980.7	7.41	Cullin-4B	CUL4B	CUL4B	Cullin-4B	31	1590.496	51.29442	3.7E-61	24	38.1161
Q13619	759	87679.6	8.3	Cullin-4A	CUL4A	CUL4A	Cullin-4A	14	450.9783	54.12382	2.61E-72	14	23.84717
Q13618	768	88929.6	8.7	Cullin-3	CUL3	CUL3	Cullin-3	7	111.735	49.2142	2.13E-53	7	10.15625
Q13617	745	86982.4	6.91	Cullin-2	CUL2	CUL2	Cullin-2	4	187.4062	41.23704	7.82E-29	4	5.234899
Q13616	776	89677.9	8.13	Cullin-1	CUL1	CUL1	Cullin-1	10	185.5305	50.35688	1.32E-57	10	15.97938
Q9NWM3	386	42257.6	5.37	CUE doma	CUEDC1	CUEDC1	CUE doma	5	103.6372	43.07081	1.09E-33	5	13.21244
Q2VPK5	515	56106.6	6.25	Cytoplasm	CTU2	CTU2	Cytoplasm	2	72.7531	39.33555	2.61E-24	2	5.436893
Q7Z7A3	348	36449.5	9.34	Cytoplasm	CTU1	CTU1	Cytoplasm	1	66.98525	28.32333	2.63E-08	1	2.586207
Q14247	550	61585.9	5.05	Src substra	CTTN	CTTN	Src substra	16	589.5125	53.6678	1.88E-70	16	31.27273
P07339	412	44551.8	6.5	Cathepsin	CTSD	CTSD	Cathepsin	6	230.2278	51.84747	2.78E-63	6	16.74757
P53634	463	51853.5	6.99	Dipeptidyl	CTSC	CTSC	Dipeptidyl	1	163.1161	36.91144	2.67E-19	1	1.727862
P07858	339	37821.3	6.27	Cathepsin	CTSB	CTSB	Cathepsin	4	124.132	46.96323	1.39E-45	4	15.33923
P10619	480	54465.7	6.59	Lysosomal	CTSA	CTSA	Lysosomal	3	63.36018	50.03404	2.11E-56	3	7.708333
Q6PD62	1173	133501.2	6.75	RNA polyn	CTR9	CTR9	RNA polyn	19	1100.016	50.69978	6.79E-59	19	20.37511
P17812	591	66689.9	6.42	CTP syntha	CTPS1	CTPS1	CTP syntha	12	512.2571	53.21201	1.2E-68	11	22.50423
O60716	968	108169.1	6.14	Catenin de	CTNND1	CTNND1	Catenin de	18	745.1799	55.22226	7.89E-77	18	23.03719
Q8WYA6	563	65172.8	4.69	Beta-cater	CTNNBL1	CTNNBL1	Beta-cater	14	661.5521	51.67956	1.23E-62	14	24.86679
P35222	781	85495.9	5.71	Catenin be	CTNNB1	CTNNB1	Catenin be	16	682.7135	51.00032	4.89E-60	16	28.68118
P35221	906	100070.3	6.17	Catenin alp	CTNNA1	CTNNA1	Catenin alp	17	884.8113	53.72232	1.13E-70	14	25.49669
O43310	598	67586.2	6.51	CBP80/20-	CTIF	CTIF	CBP80/20-	1	185.5814	41.179	1.09E-28	1	1.839465
P32929	405	44507.6	6.69	Cystathion	CTH	CTH	Cystathion	1	225.4095	42.99815	1.73E-33	1	3.209877
Q05D32	466	52998.2	6.34	CTD small	CTDSPL2	CTDSPL2	CTD small	8	896.5414	49.3575	6.46E-54	8	19.09871
Q9Y5B0	961	104398.2	4.93	RNA polyn	CTDP1	CTDP1	RNA polyn	6	262.007	53.59644	3.64E-70	6	7.492196
P49711	727	82785	6.95	Transcripti	CTCF	CTCF	Transcripti	12	638.8359	49.34349	7.25E-54	11	16.36864
Q2NKJ3	1217	134607.1	7.91	CST compl	CTC1	CTC1	CST compl	5	332.3085	44.36811	2.04E-37	5	5.258833
P56545	445	48944.4	6.95	C-termina	CTBP2	CTBP2	C-termina	6	960.9783	47.84305	1.47E-48	6	17.97753
Q13363	440	47534.9	6.76	C-termina	CTBP1	CTBP1	C-termina	8	1290.171	45.23005	5.03E-40	6	22.5
Q12996	717	82920.8	8.28	Cleavage s	CSTF3	CSTF3	Cleavage s	6	109.8364	41.96508	1.05E-30	6	10.73919
Q05048	431	48357.2	6.56	Cleavage s	CSTF1	CSTF1	Cleavage s	7	182.0069	47.04507	7.4E-46	7	19.25754
P04080	98	11139.6	7.73	Cystatin-B	CSTB	CSTB	Cystatin-B	3	320.9046	46.2729	2.55E-43	3	45.91837
P01040	98	11006.5	5.16	Cystatin-A	CSTA	CSTA	Cystatin-A	1	82.89956	42.71985	1.01E-32	1	12.2449
Q16527	193	20953.8	8.71	Cysteine ar	CSRP2	CSRP2	Cysteine ar	7	437.6057	49.15862	3.38E-53	7	45.07772
P21291	193	20567.3	8.68	Cysteine ar	CSRP1	CSRP1	Cysteine ar	1	111.1232	43.48846	7.32E-35	1	4.663212
Q6UVK1	2322	250534.7	5.15	Chondroiti	CSPG4	CSPG4	Chondroiti	14	471.4035	51.61973	2.07E-62	14	7.579673
P67870	215	24942.3	5.29	Casein kin	CSNK2B	CSNK2B	Casein kin	5	4437.527	50.21925	4.32E-57	5	27.44186
P19784	350	41212.9	8.82	Casein kin	CSNK2A2	CSNK2A2	Casein kin	17	1884.822	48.6871	1.65E-51	17	52
P68400	391	45143.3	7.86	Casein kin	CSNK2A1	CSNK2A1	Casein kin	15	3831.59	50.12342	9.79E-57	1	43.22251
P78368	415	47456.9	9.42	Casein kin	CSNK1G2	CSNK1G2	Casein kin	9	310.9388	48.6823	1.71E-51	3	22.89157
Q9HCP0	422	48511	9.34	Casein kin	CSNK1G1	CSNK1G1	Casein kin	2	121.4867	45.57453	4.25E-41	1	5.687204
P49674	416	47314.7	10.13	Casein kin	CSNK1E	CSNK1E	Casein kin	3	306.8875	47.24341	1.6E-46	3	9.855769
P48730	415	47329.7	10.2	Casein kin	CSNK1D	CSNK1D	Casein kin	8	455.654	53.13627	2.45E-68	1	20.48193
P48729	337	38914.6	10.03	Casein kin	CSNK1A1	CSNK1A1	Casein kin	8	966.9929	50.32155	1.78E-57	4	26.70623
P41240	450	50704	7.07	Tyrosine-p	CSK	CSK	Tyrosine-p	6	192.0965	48.93255	2.18E-52	6	16.44444
P55060	971	110415.4	5.55	Exportin-2	CSE1L	CSE1L	Exportin-2	16	538.6938	50.40223	8.96E-58	16	17.40474
O75534	798	88884.1	6.17	Cold shock	CSDE1	CSDE1	Cold shock	25	1379.063	53.58026	4.2E-70	25	30.57644
O75390	466	51712	8.53	Citrate syn	CS	CS	Citrate syn	5	375.4687	49.44617	3.05E-54	5	12.66094
Q08257	329	35206.4	8.7	Quinone o	CRYZ	CRYZ	Quinone o	3	107.074	43.79649	9.62E-36	3	13.37386
Q9Y4K1	2131	231706.1	5.58	Beta/gamr	CRYBG1	CRYBG1	Beta/gamr	2	67.73789	34.42859	4.77E-15	2	1.220084
O75718	401	46561.4	5.5	Cartilage-a	CRTAP	CRTAP	Cartilage-a	6	228.6286	53.07476	4.29E-68	6	16.20948
Q9BZJ0	848	100451	8.13	Crooked n	CRNKL1	CRNKL1	Crooked n	17	542.3995	52.97914	1.03E-67	17	20.99057
P46109	303	33776.7	6.73	Crk-like pr	CRKL	CRKL	Crk-like pr	6	341.2693	51.07343	2.57E-60	6	25.08251
P52943	208	22492.6	8.85	Cysteine-r	CRIP2	CRIP2	Cysteine-r	2	177.4078	49.44757	3.03E-54	2	14.42308
Q02930	508	56918.1	8.63	Cyclic AMF	CREB5	CREB5	Cyclic AMF	1	104.3633	41.28051	6.07E-29	1	1.771654

P16220	327	35136.1	4.93	Cyclic AMF	CREB1	CREB1	Cyclic AMF	1	80.3438	40.66892	1.99E-27	1	4.281346
Q96SW2	442	50545.4	5.34	Protein cer	CRBN	CRBN	Protein cer	1	231.9013	34.97456	6.6E-16	1	2.714932
Q9H3G5	476	54163.4	5.31	Probable s	CPVL	CPVL	Probable s	8	302.4489	52.75367	8.08E-67	8	20.58824
P23786	658	73776.3	8.3	Carnitine C	CPT2	CPT2	Carnitine C	1	176.4718	44.55661	5.62E-38	1	1.671733
P50416	773	88366.9	8.84	Carnitine C	CPT1A	CPT1A	Carnitine C	11	317.5479	51.72497	8.22E-63	11	17.33506
Q8N684	471	52049.5	8.15	Cleavage a	CPSF7	CPSF7	Cleavage a	15	3605.352	49.42206	3.75E-54	15	38.21656
Q16630	551	59209.3	7.16	Cleavage a	CPSF6	CPSF6	Cleavage a	13	9841.108	51.15519	1.26E-60	13	28.31216
O95639	269	30254.6	8.36	Cleavage a	CPSF4	CPSF4	Cleavage a	8	496.6915	50.65501	9.98E-59	7	31.97026
Q9UKF6	684	77485.4	5.34	Cleavage a	CPSF3	CPSF3	Cleavage a	17	544.1884	54.59221	3.23E-74	17	28.50877
Q9P2I0	782	88485.9	4.73	Cleavage a	CPSF2	CPSF2	Cleavage a	23	1628.913	51.99537	7.46E-64	23	36.18926
Q10570	1443	160882.5	6.37	Cleavage a	CPSF1	CPSF1	Cleavage a	25	2342.305	56.31043	2.31E-81	25	21.62162
P31327	1500	164938.1	6.71	Carbamoy	CPS1	CPS1	Carbamoy	4	128.4773	51.71559	8.94E-63	4	2.933333
Q9BRF8	314	35548	6.13	Serine/thre	CPPED1	CPPED1	Serine/thre	1	56.35957	40.46684	6.13E-27	1	3.503185
P36551	454	50151.6	8.33	Oxygen-de	CPOX	CPOX	Oxygen-de	19	2748.063	51.42775	1.14E-61	19	44.27313
Q86YQ8	564	63107.1	5.75	Copine-8	CPNE8	CPNE8	Copine-8	14	524.2672	52.67059	1.7E-66	7	32.2695
Q9UBL6	633	70293.3	6.33	Copine-7	CPNE7	CPNE7	Copine-7	14	1639.115	51.86181	2.44E-63	14	25.90837
O75131	537	60130.2	5.57	Copine-3	CPNE3	CPNE3	Copine-3	19	3907.157	52.13105	2.22E-64	19	35.19553
Q96FN4	548	61189.3	5.95	Copine-2	CPNE2	CPNE2	Copine-2	13	981.5318	52.68375	1.52E-66	13	31.56934
Q99829	537	59058.3	5.62	Copine-1	CPNE1	CPNE1	Copine-1	8	634.0179	47.23554	1.7E-46	8	18.06331
P14384	443	50513.5	7.4	Carboxype	CPM	CPM	Carboxype	1	94.40535	44.57127	5.08E-38	1	2.257336
Q17RY0	729	80151.4	7.18	Cytoplasm	CPEB4	CPEB4;CPE	Cytoplasm	2	182.637	51.89631	1.81E-63	2	3.566529
O75976	1380	152929.9	5.95	Carboxype	CPD	CPD	Carboxype	3	230.2675	43.75689	1.25E-35	3	2.318841
O14548	114	12614.6	9.81	Cytochron	COX7A2L	COX7A2L	Cytochron	2	34.01157	49.02991	9.76E-53	2	18.42105
P14406	83	9395.9	10.27	Cytochron	COX7A2	COX7A2	Cytochron	1	91.17653	35.23887	2.44E-16	1	12.04819
P09669	75	8781.4	10.98	Cytochron	COX6C	COX6C	Cytochron	4	140.1813	52.64656	2.1E-66	4	24
P14854	86	10192.3	7.13	Cytochron	COX6B1	COX6B1	Cytochron	1	321.4844	49.53537	1.44E-54	1	13.95349
P12074	109	12154.8	9.7	Cytochron	COX6A1	COX6A1	Cytochron	1	225.9039	44.54103	6.25E-38	1	26.6055
P10606	129	13695.6	9	Cytochron	COX5B	COX5B	Cytochron	1	146.2026	53.57975	4.2E-70	1	10.07752
P20674	150	16762	6.78	Cytochron	COX5A	COX5A	Cytochron	3	699.8366	48.07676	2.27E-49	3	27.33333
P13073	169	19576.6	10.12	Cytochron	COX4I1	COX4I1	Cytochron	6	2208.076	51.1734	1.07E-60	6	37.27811
Q5RI15	118	13291.1	8.95	Cytochron	COX20	COX20	Cytochron	3	218.9519	45.98183	2.19E-42	3	32.20339
Q9Y6N1	276	31429.7	9.32	Cytochron	COX11	COX11	Cytochron	1	64.8595	25.45846	7.65E-07	1	5.072464
Q9UQ03	480	54953	8.39	Coronin-2	CORO2B	CORO2B	Coronin-2	1	175.0931	35.97748	1.35E-17	1	2.083333
Q92828	525	59762.8	8.15	Coronin-2	CORO2A	CORO2A	Coronin-2	4	151.9038	47.52811	1.75E-47	4	8
Q9ULV4	474	53248.6	7.09	Coronin-1	CORO1C	CORO1C	Coronin-1	11	1439.787	49.69947	3.61E-55	10	25.7384
Q9BR76	489	54234.1	5.68	Coronin-1	CORO1B	CORO1B	Coronin-1	5	136.7406	48.06535	2.49E-49	5	12.26994
Q8NI60	647	71949.5	6.98	Atypical ki	COQ8A	COQ8A	Atypical ki	5	175.8901	50.96213	6.84E-60	5	9.582689
Q9Y2Z9	468	50869.4	7.32	Ubiquinon	COQ6	COQ6	Ubiquinon	2	39.54321	40.55769	3.7E-27	2	4.91453
Q5HYK3	327	37140.2	6.94	2-methoxy	COQ5	COQ5	2-methoxy	2	52.8996	43.20964	4.47E-34	2	7.33945
P61923	177	20198	4.43	Coatomer	COPZ1	COPZ1	Coatomer	2	174.2836	50.81455	2.5E-59	2	11.86441
Q99627	209	23225.4	5.02	COP9 sign	COPS8	COPS8	COP9 sign	2	139.1164	45.6541	2.39E-41	2	13.8756
Q9H9Q2	264	29621.7	6.05	COP9 sign	COPS7B	COPS7B	COP9 sign	4	91.71134	43.72013	1.6E-35	4	21.21212
Q7L5N1	327	36163.2	5.55	COP9 sign	COPS6	COPS6	COP9 sign	2	123.3462	39.98008	8.73E-26	2	8.868502
Q92905	334	37578.6	6.52	COP9 sign	COPS5	COPS5	COP9 sign	2	101.2432	40.01653	7.18E-26	2	5.389222
Q9BT78	406	46268.4	5.64	COP9 sign	COPS4	COPS4	COP9 sign	7	239.0582	45.74489	1.24E-41	7	24.13793
Q9UNS2	423	47872.8	6.64	COP9 sign	COPS3	COPS3	COP9 sign	3	129.0582	46.64445	1.57E-44	3	8.274232
P61201	443	51596.2	5.2	COP9 sign	COPS2	COPS2	COP9 sign	4	87.85453	43.4255	1.1E-34	4	10.60948
Q9UBF2	871	97621.3	5.58	Coatomer	COPG2	COPG2	Coatomer	4	133.9849	52.78291	6.21E-67	4	5.970149
Q9Y678	874	97717.3	5.14	Coatomer	COPG1	COPG1	Coatomer	15	621.0984	52.62264	2.61E-66	14	21.39588
O14579	308	34481.6	4.75	Coatomer	COPE	COPE	Coatomer	8	837.943	52.90547	2.03E-67	8	37.98701
P35606	906	102486.4	4.93	Coatomer	COPB2	COPB2	Coatomer	20	915.5832	52.84776	3.46E-67	20	26.60044
P53618	953	107141.1	5.92	Coatomer	COPB1	COPB1	Coatomer	21	734.477	51.82831	3.3E-63	21	27.91186
P53621	1224	138344.6	7.71	Coatomer	COPA	COPA	Coatomer	46	2963.49	51.73462	7.54E-63	46	49.5915
Q86VU5	262	28808.2	8.48	Catechol C	COMTD1	COMTD1	Catechol C	2	61.73818	21.07974	7.72E-06	2	8.778626
P21964	271	30036.8	5.15	Catechol C	COMT	COMT	Catechol C	5	255.3316	49.95667	4.09E-56	5	30.62731
Q8NBJ5	622	71635.4	7.33	Procollage	COLGALT1	COLGALT1	Procollage	11	1299.621	48.47086	9.59E-51	11	19.45338
P39060	1754	178186.4	5.89	Collagen a	COL18A1	COL18A1	Collagen a	6	592.2739	53.87983	2.59E-71	6	3.81984
Q5TAT6	717	69949.7	9.82	Collagen a	COL13A1	COL13A1	Collagen a	1	151.4408	42.23454	2.05E-31	1	2.37099
Q99715	3063	333144.5	5.18	Collagen a	COL12A1	COL12A1	Collagen a	10	195.0855	52.39676	2.01E-65	10	4.048319
P38432	576	62607.7	9.57	Coilin	COIL	COIL	Coilin	10	453.7976	52.35479	2.92E-65	10	20.83333
Q14746	738	83206.7	6.59	Conserved	COG2	COG2	Conserved	2	90.52885	50.67142	8.67E-59	2	3.252033
Q8WTW3	980	108977.1	7.33	Conserved	COG1	COG1	Conserved	3	107.8662	54.17284	1.64E-72	3	3.571429
Q13057	564	62328.2	6.99	Bifunctiona	COASY	COASY	Bifunctiona	1	74.24013	23.17243	3.2E-06	1	2.12766
P78357	1384	156265.4	7.05	Contactin-	CNTNAP1	CNTNAP1	Contactin-	3	115.6856	48.27141	4.8E-50	3	2.601156
Q12860	1018	113319.4	5.69	Contactin-	CNTN1	CNTN1	Contactin-	15	361.9977	52.03845	5.07E-64	15	17.87819
P09543	421	47578.2	9.5	2,3-cyclic-	CNP	CNP	2',3'-cyclic	12	353.1662	54.71327	1.01E-74	12	26.84086
Q92600	299	33630.8	8.14	CCR4-NOT	CNOT9	CNOT9	CCR4-NOT	3	75.9386	34.58562	2.73E-15	3	11.70569
Q96LI5	555	63000.9	6.73	CCR4-NOT	CNOT6L	CNOT6L;C	CCR4-NOT	1	64.53983	44.4745	9.84E-38	1	1.801802
O75175	753	81871.3	6.13	CCR4-NOT	CNOT3	CNOT3	CCR4-NOT	1	71.3032	40.22522	2.32E-26	1	1.062417
Q9NZN8	540	59737.3	7.76	CCR4-NOT	CNOT2	CNOT2	CCR4-NOT	1	181.7425	52.09034	3.17E-64	1	2.962963
Q9H9A5	744	82309	7.84	CCR4-NOT	CNOT10	CNOT10	CCR4-NOT	1	119.8	41.72705	4.4E-30	1	1.88172
A5YKK6	2376	266936.5	7.1	CCR4-NOT	CNOT1	CNOT1	CCR4-NOT	11	186.3898	48.14992	1.27E-49	11	4.924242
Q6P4Q7	775	86606	5.95	Metal tran:	CNNM4	CNNM4	Metal tran:	1	104.6224	52.11211	2.62E-64	1	1.419355
Q8NE01	707	76118.3	5.96	Metal tran:	CNNM3	CNNM3	Metal tran:	2	29.35861	48.2749	4.67E-50	2	2.970297
Q9H8M5	875	96622.3	6.33	Metal tran:	CNNM2	CNNM2	Metal tran:	1	89.36016	36.6099	9.77E-19	1	1.257143
Q15417	329	36413.5	5.92	Calponin-3	CNN3	CNN3	Calponin-3	7	300.3828	46.02535	1.59E-42	6	24.01216
Q99439	309	33696.9	7.39	Calponin-2	CNN2	CNN2	Calponin-2	1	182.8433	49.87044	8.5E-56	1	3.559871
Q96KP4	475	52878.1	5.81	Cytosolic n	CNDP2	CNDP2	Cytosolic n	1	55.01659	44.02173	2.14E-36	1	3.578947
P62633	177	19462.5	7.74	CCHC-typ	CNBP	CNBP	CCHC-typ	6	356.1897	50.91287	1.06E-59	6	36.72316
Q8N1G2	835	95320	7.05	Cap-specif	CMTR1	CMTR1	Cap-specif	16	715.795	46.1317	7.28E-43	16	20.5988
Q9NX76	183	20418.6	4.96	CKLF-like I	CMTM6	CMTM6	CKLF-like I	1	88.9486	50.17897	6.08E-57	1	6.010929
Q9BQ75	279	31884	9.82	Protein CM	CMSS1	CMSS1	Protein CM	6	492.5894	53.03675	6.08E-68	6	22.93907
P30085	196	22222.2	5.2	UMP-CMP	CMPK1	CMPK1	UMP-CMP	2	57.66048	45.83265	6.52E-42	2	10.20408
Q96DG6	245	28048	7.2	Carboxym	CMBL	CMBL	Carboxym	1	46.5882	40.17744	3.01E-26	1	3.265306
Q8NFW8	434	48378.8	8.02	N-acylneu	CMAS	CMAS	N-acylneu	12	1333.076	49.62731	6.64E-55	12	31.10599
O75153	1309	146668.5	6.04	Clustered i	CLUH	CLUH	Clustered i	5	199.3422	46.21117	4.04E-43	5	4.430863
P10909	449	52494.2	6.21	Clusterin	CLU	CLU	Clusterin	4	166.7068	49.88739	7.38E-56	4	12.69488
P53675	1640	187028.2	5.67	Clathrin he	CLTCL1	CLTCL1	Clathrin he	14	1001.758	50.41417	8.09E-58	1	8.597561
Q00610	1675	191613	5.42	Clathrin he	CLTC	CLTC	Clathrin he	41	3771.93	53.35573	3.26E-69	41	29.61194
P09496	248	27076.5	4.15	Clathrin lig	CLTA	CLTA	Clathrin lig	4	659.0892	47.32735	8.37E-47	4	10.8871
O76031	633	69223.2	7.64	ATP-depe	CLPX	CLPX	ATP-depe	9	188.9151	55.48899	6.58E-78	9	16.27172
Q96KA5	538	62228.8	8.77	Lipid scran	CLPTM1L	CLPTM1L	Lipid scran	2	227.6069	43.56856	4.34E-35	2	4.275093
O96005	669	76096	6.25	Putative lip	CLPTM1	CLPTM1	Putative lip	2	435.7343	28.87224	1.06E-08	2	2.989537

Q16740	277	30179.8	8.21	ATP-depe	CLPP	CLPP	ATP-depe	3	94.86445	38.20849	7.1E-22	3	15.52347
Q9H078	707	78728.1	9.43	Mitochonc	CLPB	CLPB	Mitochonc	13	708.1758	50.28519	2.44E-57	13	22.63083
Q92989	425	47645.2	6.6	Polyribonu	CLP1	CLP1	Polyribonu	2	258.7584	44.92456	4.34E-39	2	3.529412
P54105	237	26215.1	3.72	Methyloso	CLNS1A	CLNS1A	Methyloso	1	511.9338	49.23845	1.74E-53	1	5.485232
O75503	358	41496.3	7.46	Bis(monoa	CLN5	CLN5	Bis(monoa	1	215.1284	17.96088	2.33E-05	1	2.513966
Q96JQ2	1002	111649.7	4.58	Calmin	CLMN	CLMN	Calmin	3	228.7587	43.0235	1.47E-33	3	4.391218
Q9HAZ1	481	57491.5	8.82	Dual speci	CLK4	CLK4	Dual speci	3	76.71063	48.04025	3.05E-49	3	8.523909
P49761	490	58587.9	9.91	Dual speci	CLK3	CLK3	Dual speci	11	1327.185	51.89346	1.85E-63	11	28.16327
P49760	499	60089.6	10	Dual speci	CLK2	CLK2	Dual speci	3	300.2158	55.45567	8.97E-78	3	7.214429
P49759	484	57290.1	9.11	Dual speci	CLK1	CLK1	Dual speci	7	257.4669	48.73417	1.12E-51	6	16.1157
Q9UDT6	1046	115836.5	6.7	CAP-Gly d	CLIP2	CLIP2	CAP-Gly d	13	419.6823	58.80244	1.26E-91	12	14.24474
Q14677	625	68259	6.35	Clathrin in	CLINT1	CLINT1	Clathrin in	7	187.8192	45.21193	5.72E-40	7	12.64
Q9Y696	253	28771.8	5.26	Chloride ir	CLIC4	CLIC4	Chloride ir	4	136.7286	48.22866	6.77E-50	4	26.08696
O00299	241	26922.5	4.82	Chloride ir	CLIC1	CLIC1	Chloride ir	8	817.2838	50.78594	3.2E-59	8	32.78008
O75596	197	22232.4	9.11	C-type lec	CLEC3A	CLEC3A	C-type lec	1	168.6309	41.0592	2.18E-28	1	10.6599
P51798	805	88678.3	8.57	H(+)/Cl(-)	CLCN7	CLCN7	H(+)/Cl(-)	1	135.7474	34.02663	1.92E-14	1	1.118012
Q8N2M8	674	77161.4	10.99	CLK4-asso	CLASRP	CLASRP	CLK4-asso	9	271.6172	54.52578	6.11E-74	9	15.57864
O75122	1294	141062.1	8.57	CLIP-assoc	CLASP2	CLASP2	CLIP-assoc	2	111.0696	49.77497	1.91E-55	2	2.318393
P12532	417	47036.3	8.47	Creatine ki	CKMT1A	CKMT1A	Creatine ki	9	757.3393	53.34368	3.65E-69	7	27.57794
P12277	381	42643.9	5.3	Creatine ki	CKB	CKB	Creatine ki	9	539.2293	49.83868	1.12E-55	8	32.8084
Q14008	2032	225492.5	7.88	Cytoskelet	CKAP5	CKAP5	Cytoskelet	5	248.6844	53.61164	3.17E-70	5	2.952756
Q07065	602	66022	5.76	Cytoskelet	CKAP4	CKAP4	Cytoskelet	17	800.6948	51.09934	2.05E-60	17	34.5515
Q8IYA6	745	83586.1	10.53	Cytoskelet	CKAP2L	CKAP2L	Cytoskelet	3	116.1106	44.79641	1.07E-38	3	4.563758
P0C7P0	127	14215.4	11.2	CDGSH iro	CISD3	CISD3	CDGSH iro	1	22.09024	29.83276	1.72E-09	1	8.661417
Q8N5K1	135	15278.1	10.25	CDGSH iro	CISD2	CISD2	CDGSH iro	4	246.3979	48.3918	1.81E-50	4	36.2963
Q9NZ45	108	12199	9.5	CDGSH iro	CISD1	CISD1	CDGSH iro	2	229.9491	48.56747	4.38E-51	2	22.22222
Q14011	172	18647.9	9.82	Cold-indu	CIRBP	CIRBP	Cold-indu	3	1059.943	48.41678	1.49E-50	3	20.93023
Q86X95	450	52313	10.57	Corepresso	CIR1	CIR1	Corepresso	1	532.3137	48.03181	3.26E-49	1	2
Q9BW66	212	24323.6	6.24	Cyclin-dep	CINP	CINP	Cyclin-dep	5	125.6122	48.2678	4.94E-50	5	25
Q6FI81	312	33581.9	5.3	Anamorsin	CIAPIN1	CIAPIN1	Anamorsin	2	79.89951	38.53512	1.46E-22	2	8.012821
Q9Y3D0	163	17662.9	4.85	Cytosolic ir	CIAO2B	CIAO2B	Cytosolic ir	1	60.60424	49.2237	1.97E-53	1	12.88344
Q9H5X1	160	18354.9	4.55	Cytosolic ir	CIAO2A	CIAO2A	Cytosolic ir	1	44.81097	31.73435	1.98E-11	1	5.625
O76071	339	37839.7	4.59	Probable c	CIAO1	CIAO1	Probable c	5	317.7444	52.422	1.59E-65	5	20.059
O15111	745	84638.9	6.72	Inhibitor o	CHUK	CHUK	Inhibitor o	6	261.0371	49.76527	2.07E-55	6	9.798658
Q9Y3Y2	248	26396.4	12.74	Chromatin	CHTOP	CHTOP	Chromatin	3	320.0923	54.34033	3.61E-73	3	14.51613
Q8WVB6	975	107381.9	7.21	Chromoso	CHTF18	CHTF18	Chromoso	3	441.2524	50.77672	3.47E-59	3	3.487179
Q86X52	802	91783.8	9.63	Chondroiti	CHSY1	CHSY1	Chondroiti	1	46.10086	25.23042	9.17E-07	1	1.246883
Q8IZ52	775	85466.4	6.92	Chondroiti	CHPF	CHPF	Chondroiti	5	227.9332	46.72709	8.39E-45	5	9.032258
Q9UHD1	332	37489.5	7.95	Cysteine ar	CHORDC1	CHORDC1	Cysteine ar	6	110.7455	45.23651	4.81E-40	6	21.08434
Q96FZ7	201	23484.7	4.98	Charged m	CHMP6	CHMP6	Charged m	1	93.02967	51.36692	1.96E-61	1	6.467662
Q9NZZ3	219	24570.5	4.43	Charged m	CHMP5	CHMP5	Charged m	2	112.8602	33.7899	4.27E-14	2	16.89498
Q9H444	224	24950	4.47	Charged m	CHMP4B	CHMP4B	Charged m	2	105.4792	50.3791	1.09E-57	2	10.71429
Q9Y3E7	222	25072.8	4.79	Charged m	CHMP3	CHMP3	Charged m	2	123.9829	47.37793	5.65E-47	2	9.459459
Q9UQN3	213	23906.4	9.47	Charged m	CHMP2B	CHMP2B	Charged m	1	151.5753	48.41239	1.54E-50	1	4.694836
O43633	222	25103.7	5.6	Charged m	CHMP2A	CHMP2A	Charged m	3	128.4141	45.70915	1.6E-41	3	15.76577
Q9HD42	196	21702.7	8.46	Charged m	CHMP1A	CHMP1A	Charged m	2	282.936	48.79473	6.81E-52	2	8.163265
Q8IWX8	916	103701.6	9.39	Calcium hc	CHERP	CHERP	Calcium hc	15	2116.732	52.53788	5.62E-66	15	19.32314
Q3L8U1	2897	326019.2	7	Chromodc	CHD9	CHD9	Chromodc	26	1444.278	51.46307	8.31E-62	23	11.14946
Q9HCK8	2581	290516.7	6.43	Chromodc	CHD8	CHD8	Chromodc	19	945.9767	54.5779	3.68E-74	12	9.647423
Q14839	1912	218003	5.63	Chromodc	CHD4	CHD4	Chromodc	43	2287.253	56.68067	7.17E-83	28	22.90795
O14647	1828	211342	8.28	Chromodc	CHD2	CHD2	Chromodc	32	2490.81	51.79529	4.42E-63	32	21.4442
Q86WJ1	897	100999.1	6.9	Chromodc	CHD1L	CHD1L	Chromodc	31	2129.167	53.13524	2.46E-68	31	39.3534
O14646	1710	196685.9	7.13	Chromodc	CHD1	CHD1	Chromodc	48	4026.682	55.03138	4.8E-76	39	28.0117
Q9NX63	227	26152.2	8.45	MICOS cor	CHCHD3	CHCHD3	MICOS cor	8	633.7632	50.86213	1.65E-59	8	29.07489
Q96JM3	812	89098.2	8.63	Chromoso	CHAMP1	CHAMP1	Chromoso	31	970.9037	51.93407	1.29E-63	31	41.37931
Q13111	956	106909	5.71	Chromatin	CHAF1A	CHAF1A	Chromatin	7	772.1117	51.43422	1.07E-61	7	9.518828
Q9P2M7	1203	137055.7	5.19	Cingulin	CGN	CGN	Cingulin	18	393.4023	51.74584	6.83E-63	18	16.87448
O15519	480	55343.6	8.11	CASP8 anc	CFLAR	CFLAR	CASP8 anc	1	619.9395	26.33878	3.42E-07	1	2.916667
Q9Y281	166	18736.5	8.17	Cofilin-2	CFL2	CFL2	Cofilin-2	2	54.67182	39.76243	2.79E-25	2	13.25301
P23528	166	18502.3	8.29	Cofilin-1	CFL1	CFL1	Cofilin-1	11	9545.492	47.93986	6.8E-49	10	53.01205
Q9Y6A4	193	22774.3	10.27	Cilia- and	CFAP20	CFAP20	Cilia- and	7	1390.349	50.78671	3.18E-59	7	35.7513
P41208	172	19738.3	4.62	Centrin-2	CETN2	CETN2	Centrin-2	7	232.134	52.27166	6.15E-65	5	51.16279
Q6ZMG9	384	44889.5	7.72	Ceramide s	CERS6	CERS6	Ceramide s	1	200.2899	52.09324	3.1E-64	1	2.083333
Q9Y6K0	416	46553.1	8.25	Choline/et	CEPT1	CEPT1	Choline/et	1	965.0766	46.35673	1.36E-43	1	2.644231
Q5JTW2	689	76395.6	8.28	Centrosom	CEP78	CEP78	Centrosom	4	66.22513	45.25231	4.29E-40	4	6.676343
Q53EZ4	464	54177.8	7	Centrosom	CEP55	CEP55	Centrosom	2	72.42786	38.88228	2.62E-23	2	4.310345
Q9Y4F5	1589	171686.2	6.83	Centrosom	CEP170B	CEP170B	Centrosom	3	47.75211	43.16339	6.02E-34	3	2.139711
Q5SW79	1584	175291.4	7.11	Centrosom	CEP170	CEP170	Centrosom	5	159.9583	41.83428	2.31E-30	4	3.787879
Q9UPN4	1083	122147.7	9.1	Centrosom	CEP131	CEP131	Centrosom	3	80.78036	47.28079	1.2E-46	3	3.693444
Q7Z7K6	275	29945.6	10.4	Centromer	CENPV	CENPV	Centromer	5	772.1521	49.07523	6.73E-53	5	25.09091
P49454	3114	357524.4	4.77	Centromer	CENPF	CENPF	Centromer	1	107.2491	42.4095	6.97E-32	1	0.32113
Q03188	943	106833	10.01	Centromer	CENPC	CENPC	Centromer	8	432.7584	48.13036	1.48E-49	8	9.225875
P07199	599	65170.7	4.2	Major cent	CENPB	CENPB	Major cent	11	786.007	51.20953	7.79E-61	11	27.04508
Q86UT8	332	37973.9	8.33	Centrosom	CENATAC	CENATAC	Centrosom	1	135.6119	41.34975	4.06E-29	1	2.710843
Q8WUJ3	1361	152996.8	7.91	Cell migrat	CEMIP	CEMIP	Cell migrat	3	80.69422	39.73527	3.23E-25	3	2.571639
Q92879	486	52062.8	8.63	CUGBP Ela	CELF1	CELF1	CUGBP Ela	2	146.4717	53.52575	6.85E-70	1	4.115226
Q03701	1054	120973	5.73	CCAAT/en	CEBPZ	CEBPZ	CCAAT/en	24	1204.289	51.65402	1.54E-62	24	24.76281
P17676	345	36105.4	8.45	CCAAT/en	CEBPB	CEBPB	CCAAT/en	3	144.0157	48.67402	1.83E-51	3	11.88406
Q9Y232	598	66481.2	10.07	Chromodc	CDYL	CDYL	Chromodc	8	221.6854	51.77099	5.45E-63	7	15.55184
Q99626	313	33519.5	10.01	Homeobox	CDX2	CDX2	Homeobox	1	14.37578	29.4033	4.02E-09	1	3.833866
Q15517	529	51606.8	8.53	Corneodes	CDSN	CDSN	Corneodes	2	366.237	36.03059	1.09E-17	2	5.10397
O95674	445	51417.5	7.1	Phosphatic	CDS2	CDS2	Phosphatic	1	68.50882	44.72651	1.73E-38	1	2.696629
Q9NXV6	580	61124.2	9.54	CDKN2A-i	CDKN2AIP	CDKN2AIP	CDKN2A-i	4	119.0379	52.72046	1.09E-66	4	8.793103
P38936	164	18119.1	8.5	Cyclin-dep	CDKN1A	CDKN1A	Cyclin-dep	1	146.087	25.91075	5.17E-07	1	7.317073
Q5VV42	579	65111	7.51	Threonylca	CDKAL1	CDKAL1	Threonylca	3	107.0886	46.16079	5.87E-43	3	6.56304
P50750	372	42777.2	9.07	Cyclin-dep	CDK9	CDK9	Cyclin-dep	12	5735.15	50.36136	1.27E-57	12	35.75269
P49336	464	53283.3	8.82	Cyclin-dep	CDK8	CDK8	Cyclin-dep	2	70.96346	37.57889	1.35E-20	1	3.663793
Q96JB5	506	56920.2	4.4	CDK5 regu	CDK5RAP3	CDK5RAP3	CDK5 regu	2	33.54934	45.55383	4.94E-41	2	5.533597
Q96SZ6	601	67687.9	8.28	Mitochonc	CDK5RAP1	CDK5RAP1	Mitochonc	2	282.4649	43.84309	7.06E-36	2	3.826955
Q00535	292	33304.1	7.75	Cyclin-dep	CDK5	CDK5	Cyclin-dep	5	189.2231	49.57332	1.05E-54	5	22.60274
P24941	298	33929.2	8.99	Cyclin-dep	CDK2	CDK2	Cyclin-dep	7	196.7731	49.23034	1.86E-53	5	31.87919
Q00537	523	59581.7	9.44	Cyclin-dep	CDK17	CDK17	Cyclin-dep	1	24.55762	29.74571	2.05E-09	1	2.294455
Q14004	1512	164921.5	10.28	Cyclin-dep	CDK13	CDK13	Cyclin-dep	5	163.6878	53.16563	1.86E-68	5	4.497354

Q9NYV4	1490	164153	10	Cyclin-dep	CDK12	CDK12	Cyclin-dep	14	255.1611	49.09035	5.93E-53	13	11.74497
P21127	795	92619.2	5.23	Cyclin-dep	CDK11B	CDK11B	Cyclin-dep	24	2855.514	51.8825	2.04E-63	2	30.18868
Q9UQ88	783	91360.9	5.04	Cyclin-dep	CDK11A	CDK11A	Cyclin-dep	2	135.6119	35.85199	2.23E-17	2	3.065134
Q15131	360	41037.7	9.08	Cyclin-dep	CDK10	CDK10	Cyclin-dep	5	1234.484	53.62066	2.94E-70	5	14.72222
P06493	297	34095.1	8.75	Cyclin-dep	CDK1	CDK1	Cyclin-dep	8	207.2231	50.53526	2.85E-58	8	31.98653
O14735	213	23538.5	8.13	CDP-diacy	CDIPT	CDIPT	CDP-diacy	1	33.32059	27.36032	1.05E-07	1	6.103286
P22223	829	91417.7	4.35	Cadherin-3	CDH3	CDH3	Cadherin-3	1	484.2202	46.63021	1.75E-44	1	1.688782
P12830	882	97455.3	4.33	Cadherin-1	CDH1	CDH1	Cadherin-1	5	555.9254	46.82446	4.01E-45	5	7.709751
Q9H5V8	836	92930.9	8.02	CUB doma	CDCP1	CDCP1	CUB doma	16	1783.869	51.6224	2.03E-62	16	22.00957
Q53HL2	280	31322.6	10.58	Borealin	CDCA8	CDCA8	Borealin	8	875.6459	50.16462	6.87E-57	8	32.85714
Q96GN5	454	52205.7	5.41	Cell divisio	CDCA7L	CDCA7L	Cell divisio	6	410.0627	49.75041	2.34E-55	5	12.77533
Q96FF9	252	27600.3	10.35	Sororin	CDCA5	CDCA5	Sororin	2	220.6165	51.6452	1.66E-62	2	11.50794
Q6P1J9	531	60576.1	10.22	Parafibrom	CDC73	CDC73	Parafibrom	24	855.197	50.99911	4.94E-60	24	46.89266
Q99459	802	92249.9	8.47	Cell divisio	CDC5L	CDC5L	Cell divisio	19	1825.354	52.77608	6.62E-67	19	31.42145
Q9H3Q1	356	37979.4	4.82	Cdc42 effe	CDC42EP4	CDC42EP4	Cdc42 effe	1	89.53319	28.13383	3.53E-08	1	3.932584
Q6DT37	1551	172457.6	6.21	Serine/thre	CDC42BPG	CDC42BPG	Serine/thre	3	90.90024	43.16138	6.09E-34	3	2.836879
Q9Y5S2	1711	194313.5	6.31	Serine/thre	CDC42BPB	CDC42BPB	Serine/thre	12	212.9344	43.79584	9.66E-36	10	8.123904
Q5VT25	1732	197305.2	6.55	Serine/thre	CDC42BPA	CDC42BPA	Serine/thre	17	364.7985	51.74081	7.14E-63	17	12.47113
P60953	191	21258.4	6.51	Cell divisio	CDC42	CDC42	Cell divisio	4	401.3704	45.51485	6.55E-41	4	23.56021
O60508	579	65520.8	7.07	Pre-mRNA	CDC40	CDC40	Pre-mRNA	13	708.1943	52.65512	1.95E-66	13	26.59758
Q16543	378	44468	4.9	Hsp90 co-	CDC37	CDC37	Hsp90 co-	9	811.4769	52.2208	9.68E-65	9	25.39683
P49427	236	26736.5	4.15	Ubiquitin-	CDC34	CDC34	Ubiquitin-	1	151.1858	42.54794	2.95E-32	1	4.661017
P30260	824	91866.4	7.03	Cell divisio	CDC27	CDC27	Cell divisio	6	155.8168	50.73555	4.97E-59	6	8.859223
Q9UJX2	597	68833.4	7.03	Cell divisio	CDC23	CDC23	Cell divisio	2	88.09575	39.97153	9.14E-26	2	3.517588
Q13042	620	71654.9	5.68	Cell divisio	CDC16	CDC16	Cell divisio	4	62.12444	22.1109	5.14E-06	4	5.645161
O75794	336	39134.4	4.39	Translation	CDC123	CDC123	Translation	4	217.2242	46.79379	5.06E-45	4	14.88095
P32320	146	16184.5	6.95	Cytidine de	CDA	CDA	Cytidine de	4	193.9347	46.1176	8.08E-43	4	36.9863
P21926	228	25415.8	7.19	CD9 antige	CD9	CD9	CD9 antige	1	1094.474	46.12825	7.47E-43	1	4.385965
P60033	236	25809.2	4.9	CD81 antig	CD81	CD81	CD81 antig	1	39.27898	36.6701	7.56E-19	1	9.745763
P13987	128	14177.2	6.47	CD59 glyco	CD59	CD59	CD59 glyco	3	473.5713	52.11766	2.5E-64	3	18.75
P08174	381	41399.8	7.64	Compleme	CD55	CD55	Compleme	5	570.4695	47.40156	4.71E-47	5	12.33596
Q08722	323	35213.3	7.27	Leukocyte	CD47	CD47	Leukocyte	1	508.6115	45.88115	4.58E-42	1	3.405573
P16070	742	81537	5	CD44 antig	CD44	CD44	CD44 antig	7	4865.86	50.7214	5.62E-59	7	9.838275
P25942	277	30618.8	5.57	Tumor nec	CD40	CD40	Tumor nec	1	153.7143	35.83487	2.39E-17	1	3.249097
O95400	341	37646.2	4.23	CD2 antige	CD2BP2	CD2BP2	CD2 antige	7	712.4837	46.09444	9.58E-43	7	29.91202
Q9Y5K6	639	71450.5	6.31	CD2-assoc	CD2AP	CD2AP	CD2-assoc	4	179.6904	42.9745	2.01E-33	4	7.355243
Q5ZPR3	534	57235	4.52	CD276 ant	CD276	CD276	CD276 ant	1	97.3042	48.80753	6.13E-52	1	5.243446
P48509	253	28294.9	7.52	CD151 ant	CD151	CD151	CD151 ant	2	756.5574	45.11763	1.12E-39	2	7.509881
Q6YHK3	1445	161687.8	5.62	CD109 ant	CD109	CD109	CD109 ant	22	450.2441	52.80021	5.34E-67	22	18.4083
P86790	482	55865.7	6.45	Vacuolar fu	CCZ1B	CCZ1B;CCZ	Vacuolar fu	2	123.5674	47.14917	3.31E-46	2	4.149378
P50990	548	59620.1	5.3	T-complex	CCT8	CCT8	T-complex	24	1006.692	54.08581	3.7E-72	24	51.09489
Q99832	543	59366.1	7.73	T-complex	CCT7	CCT7	T-complex	20	1107.435	50.42363	7.45E-58	20	45.11971
P40227	531	58023.6	6.65	T-complex	CCT6A	CCT6A	T-complex	15	1243.826	51.49852	6.04E-62	12	33.89831
P48643	541	59670.5	5.34	T-complex	CCT5	CCT5	T-complex	15	494.5387	48.5143	6.75E-51	15	28.65065
P50991	539	57923.6	7.94	T-complex	CCT4	CCT4	T-complex	17	667.5134	50.38957	9.98E-58	17	37.47681
P49368	545	60533.3	6.44	T-complex	CCT3	CCT3	T-complex	23	1726.031	50.26766	2.84E-57	23	42.38532
P78371	535	57487.6	6.42	T-complex	CCT2	CCT2	T-complex	21	568.7299	51.09419	2.14E-60	21	50.28037
O14618	274	29040.4	5.31	Copper ch	CCS	CCS	Copper ch	1	243.9178	49.94201	4.64E-56	1	4.744526
Q8N7R7	359	40704.6	5.97	Cyclin-Y-li	CCNYL1	CCNYL1	Cyclin-Y-li	1	126.1904	43.09255	9.47E-34	1	2.228412
O60583	730	81028.4	9.32	Cyclin-T2	CCNT2	CCNT2	Cyclin-T2	10	502.1335	53.84639	3.53E-71	10	15.06849
O60563	726	80684	9.14	Cyclin-T1	CCNT1	CCNT1	Cyclin-T1	22	1824.384	53.55392	5.31E-70	20	34.29752
Q8N1B3	248	28368.4	6.23	Cyclin-Q	CCNQ	CCNQ	Cyclin-Q	1	235.7632	40.72049	1.49E-27	1	5.645161
Q96S94	520	58146.8	10.89	Cyclin-L2	CCNL2	CCNL2	Cyclin-L2	3	82.86406	48.35127	2.52E-50	3	11.53846
Q9UK58	526	59633	11.34	Cyclin-L1	CCNL1	CCNL1	Cyclin-L1	4	254.0542	47.69825	4.59E-48	4	9.505703
O75909	580	64239.4	8.59	Cyclin-K	CCNK	CCNK	Cyclin-K	3	88.1213	37.89482	3.13E-21	3	7.586207
P30279	289	33066.9	4.81	G1/S-spec	CCND2	CCND2	G1/S-spec	1	26.57792	39.28447	3.39E-24	1	3.114187
O00622	381	42026.2	8.25	CCN family	CCN1	CCN1	CCN family	10	544.8787	57.86985	9.8E-88	10	27.29659
Q6ZUT6	534	57324.4	9.59	Coiled-coi	CCDC9B	CCDC9B	Coiled-coi	2	50.36523	53.21903	1.13E-68	2	5.243446
Q9Y3X0	531	59702.6	5.08	Coiled-coi	CCDC9	CCDC9	Coiled-coi	2	341.5771	47.94209	6.68E-49	2	3.578154
Q9H6F5	360	40235.3	11.07	Coiled-coi	CCDC86	CCDC86	Coiled-coi	10	798.0623	54.03593	5.91E-72	10	34.44444
Q8N4S0	544	64001.6	4.64	Coiled-coi	CCDC82	CCDC82	Coiled-coi	12	764.2552	51.28715	3.95E-61	12	29.22794
Q8N9Z2	235	26261	12.21	Coiled-coi	CCDC71L	CCDC71L	Coiled-coi	7	2044.08	47.92044	7.93E-49	7	35.31915
Q8IV32	467	49647.6	12.32	Coiled-coi	CCDC71	CCDC71	Coiled-coi	6	235.8967	49.49407	2.05E-54	6	14.77516
Q9P031	241	28669.7	10.55	Thyroid tra	CCDC59	CCDC59	Thyroid tra	9	334.8275	49.51597	1.7E-54	9	39.83402
Q8IVM0	306	35821.7	6.64	Coiled-coi	CCDC50	CCDC50	Coiled-coi	6	296.9277	50.74705	4.5E-59	6	28.10458
Q96A33	483	55873.5	4.49	PAT comp	CCDC47	CCDC47	PAT comp	10	434.6153	48.55831	4.72E-51	10	17.80538
Q86WR0	208	24478.7	6.8	Coiled-coi	CCDC25	CCDC25	Coiled-coi	1	87.75948	46.51523	4.15E-44	1	4.326923
Q5BJE1	867	102010.3	6.75	Coiled-coi	CCDC178	CCDC178	Coiled-coi	1	125.9355	20.90529	8.24E-06	1	1.038062
P0C221	793	93624.9	6.68	Coiled-coi	CCDC175	CCDC175	Coiled-coi	1	49.16648	42.04353	6.56E-31	1	1.008827
Q8IYE0	955	112805.7	8.89	Coiled-coi	CCDC146	CCDC146	Coiled-coi	1	265.6093	35.6273	5.45E-17	1	0.732984
Q6ZP82	1530	175065.4	5.7	Coiled-coi	CCDC141	CCDC141	Coiled-coi	1	60.56767	34.0928	1.54E-14	1	0.653595
Q6PK04	289	33231.2	11.59	Coiled-coi	CCDC137	CCDC137	Coiled-coi	5	549.2915	49.37972	5.36E-54	5	15.91696
Q96BQ5	260	30834	9.62	Coiled-coi	CCDC127	CCDC127	Coiled-coi	4	183.8078	41.03988	2.43E-28	4	15.38462
Q8WUD4	166	19180.6	7.52	Coiled-coi	CCDC12	CCDC12	Coiled-coi	2	225.4562	42.58578	2.33E-32	2	21.08434
Q8N163	923	102900.6	4.89	Cell cycle a	CCAR2	CCAR2	Cell cycle a	12	578.7072	52.93214	1.59E-67	12	16.25135
Q8IX12	1150	132820	5.45	Cell divisio	CCAR1	CCAR1	Cell divisio	28	3394.069	51.79252	4.52E-63	28	30.43478
Q6P1N0	951	104061.5	8.26	Coiled-coi	CC2D1A	CC2D1A	Coiled-coi	1	173.2549	43.39557	1.34E-34	1	1.366982
Q9HC52	389	43395.3	10.56	Chromobo	CBX8	CBX8	Chromobo	16	6682.268	49.35374	6.66E-54	16	45.50129
O95931	251	28340.3	10.02	Chromobo	CBX7	CBX7	Chromobo	3	331.4453	48.48936	8.25E-51	3	13.14741
O95503	412	43897.8	10.72	Chromobo	CBX6	CBX6	Chromobo	8	1141.161	51.26537	4.79E-61	7	26.45631
P45973	191	22224.8	5.57	Chromobo	CBX5	CBX5	Chromobo	11	2465.966	51.23093	6.46E-61	11	47.64398
O00257	560	61367.2	9.94	E3 SUMO-	CBX4	CBX4	E3 SUMO-	12	3062.767	57.91145	7.21E-88	12	21.60714
Q13185	183	20811.2	4.96	Chromobo	CBX3	CBX3	Chromobo	9	8705.956	50.15305	7.59E-57	7	43.1694
Q14781	532	56080.1	10.76	Chromobo	CBX2	CBX2	Chromobo	10	363.6397	53.26753	7.28E-69	10	23.68421
P83916	185	21417.6	4.57	Chromobo	CBX1	CBX1	Chromobo	7	10011.78	50.05138	1.82E-56	7	35.13514
Q8N4T8	237	25301.2	9.93	3-oxoacyl-	CBR4	CBR4	3-oxoacyl-	3	231.6614	46.46492	6.06E-44	3	21.51899
Q75N03	491	54518.8	8.38	E3 ubiquiti	CBLL1	CBLL1	E3 ubiquiti	2	139.5678	50.74761	4.48E-59	2	5.295316
P22681	906	99631.8	6.51	E3 ubiquiti	CBL	CBL	E3 ubiquiti	3	91.89516	37.88771	3.24E-21	2	5.298013
Q13951	182	21508	6.55	Core-bind	CBFB	CBFB	Core-bind	2	122.3479	52.90952	1.95E-67	2	14.28571
Q969G5	261	27700.9	6.39	Caveolae-a	CAVIN3	CAVIN3	Caveolae-a	2	209.9954	47.2086	2.1E-46	2	8.429119
Q6NZI2	390	43475.8	5.28	Caveolae-a	CAVIN1	CAVIN1	Caveolae-a	9	891.7738	52.3562	2.89E-65	9	25.12821
P51636	162	18291	4.9	Caveolin-2	CAV2	CAV2	Caveolin-2	2	259.6002	47.11364	4.37E-46	2	17.28395
Q03135	178	20471.4	5.93	Caveolin-1	CAV1	CAV1	Caveolin-1	8	1564.107	50.46065	5.41E-58	7	50

P04040	527	59755.8	7.41	Catalase	CAT	CAT	Catalase	10	150.0272	47.20263	2.2E-46	10	23.52941
P20810	708	76572	4.7	Calpastatir	CAST	CAST	Calpastatir	2	67.77274	48.35683	2.41E-50	2	4.378531
P55211	416	46280.3	5.91	Caspase-9	CASP9	CASP9	Caspase-9	1	14.34264	19.10972	1.56E-05	1	4.567308
Q14790	479	55390.5	4.73	Caspase-8	CASP8	CASP8	Caspase-8	1	43.7335	21.68757	6.11E-06	1	2.922756
P55210	303	34276.5	5.93	Caspase-7	CASP7	CASP7	Caspase-7	1	655.6879	52.91783	1.81E-67	1	2.640264
P31944	242	27679.3	5.28	Caspase-1	CASP14	CASP14	Caspase-1	3	78.65407	47.3351	7.89E-47	3	14.46281
O14936	926	105121.9	6.4	Peripheral	CASK	CASK	Peripheral	13	383.5541	51.35636	2.14E-61	13	15.65875
O15234	703	76277.5	6.44	Protein CA	CASC3	CASC3	Protein CA	4	89.80848	45.73839	1.3E-41	4	6.827881
P49589	748	85472.7	6.73	Cysteine--	CARS1	CARS1	Cysteine--	14	297.4572	53.86556	2.95E-71	14	20.32086
Q8N4J0	409	47185.3	6.25	Carnosine	CARNMT1	CARNMT1	Carnosine	1	81.37434	51.56014	3.52E-62	1	2.689487
Q5VZK9	1371	151555.3	7.93	F-actin-un	CARMIL1	CARMIL1	F-actin-un	3	100.4574	46.8816	2.59E-45	3	2.69876
Q86X55	608	65853.2	6.73	Histone-ar	CARM1	CARM1	Histone-ar	6	315.924	49.83977	1.11E-55	6	12.00658
Q9Y2V2	147	15891.9	8.35	Calcium-re	CARHSP1	CARHSP1	Calcium-re	2	73.75462	43.01737	1.53E-33	2	18.36735
Q96LW7	228	25589.2	9.37	Caspase re	CARD19	CARD19	Caspase re	4	211.9899	47.90591	8.89E-49	4	19.29825
P47756	272	30628.5	5.78	F-actin-ca	CAPZB	CAPZB	F-actin-ca	12	1431.026	52.07254	3.71E-64	12	44.11765
P47755	286	32948.9	5.65	F-actin-ca	CAPZA2	CAPZA2	F-actin-ca	5	1538.763	48.0939	1.98E-49	3	24.47552
P52907	286	32922.5	5.42	F-actin-ca	CAPZA1	CAPZA1	F-actin-ca	5	631.905	51.17589	1.05E-60	5	30.06993
Q14444	709	78365.9	4.91	Caprin-1	CAPRIN1	CAPRIN1	Caprin-1	2	139.1669	44.4493	1.17E-37	2	2.820874
P04632	268	28315.6	4.82	Calpain sm	CAPNS1	CAPNS1	Calpain sm	5	167.443	48.01867	3.62E-49	5	29.47761
Q9Y6W3	813	92651.6	7.72	Calpain-7	CAPN7	CAPN7	Calpain-7	4	265.3082	38.84477	3.16E-23	4	4.920049
Q9Y6Q1	641	74575.9	7.05	Calpain-6	CAPN6	CAPN6	Calpain-6	5	245.4371	47.875	1.14E-48	5	11.38846
O15484	640	73168.4	7.68	Calpain-5	CAPN5	CAPN5	Calpain-5	11	316.0977	51.7248	8.23E-63	11	20.78125
P17655	700	79994.5	4.6	Calpain-2	CAPN2	CAPN2	Calpain-2	7	332.9524	50.84699	1.88E-59	7	11.71429
P07384	714	81889.3	5.35	Calpain-1	CAPN1	CAPN1	Calpain-1	14	606.7517	53.3335	4E-69	14	18.62745
P40121	348	38498.3	6.12	Macrophag	CAPG	CAPG	Macrophag	2	79.2846	39.50418	1.09E-24	2	7.183908
Q01518	475	51901.1	8.2	Adenylyl c	CAP1	CAP1	Adenylyl c	6	394.008	49.76476	2.08E-55	6	15.57895
P27824	592	67567.7	4.21	Calnexin	CANX	CANX	Calnexin	18	1294.716	48.19315	8.98E-50	18	30.06757
Q86VP6	1230	136374.1	5.54	Cullin-asso	CAND1	CAND1	Cullin-asso	22	1018.029	52.95577	1.28E-67	21	22.60163
Q9P1Y5	1249	134748.1	8.5	Calmoduli	CAMSAP3	CAMSAP3	Calmoduli	4	104.1252	47.32034	8.84E-47	4	4.403523
Q08AD1	1489	168087.5	6.78	Calmoduli	CAMSAP2	CAMSAP2	Calmoduli	4	85.00608	42.8349	4.88E-33	4	4.298187
Q5T5Y3	1602	177970.2	6.7	Calmoduli	CAMSAP1	CAMSAP1	Calmoduli	3	56.41035	35.34988	1.6E-16	3	2.434457
Q13555	558	62606.7	7.89	Calcium/ca	CAMK2G	CAMK2G	Calcium/ca	5	148.5315	46.37513	1.19E-43	3	12.72401
Q13557	499	56368.9	7.26	Calcium/ca	CAMK2D	CAMK2D	Calcium/ca	8	1334.906	54.83143	3.16E-75	4	17.83567
O43852	315	37106.5	4.23	Calumenin	CALU	CALU	Calumenin	2	80.09473	25.81438	5.64E-07	2	4.761905
P27797	417	48141.2	4.04	Calreticulir	CALR	CALR	Calreticulir	9	287.5418	51.67991	1.23E-62	9	26.3789
P0DP23	149	16837.5	3.84	Calmoduli	CALM1	CALM1;CA	Calmoduli	4	371.0889	48.92233	2.38E-52	4	26.84564
Q05682	793	93230.5	5.35	Caldesmor	CALD1	CALD1	Caldesmor	4	103.9988	45.73365	1.34E-41	4	6.30517
Q13137	446	52253.6	4.66	Calcium-b	CALCOCO	CALCOCO	Calcium-b	1	210.8146	39.83739	1.87E-25	1	2.914798
P05937	261	30024.8	4.44	Calbindin	CALB1	CALB1;CAL	Calbindin;(	1	71.15295	49.96043	3.96E-56	1	4.214559
P27708	2225	242981.7	6.42	Multifuncti	CAD	CAD	Multifuncti	26	656.348	53.32067	4.51E-69	25	14.42697
Q9HB71	228	26209.8	8.59	Calcyclin-t	CACYBP	CACYBP	Calcyclin-t	5	525.157	46.52374	3.9E-44	5	21.92982
Q8WUQ7	758	88701	9.62	Splicing fa	CACTIN	CACTIN	Splicing fa	12	466.4583	50.93109	8.97E-60	12	19.92084
Q9NY47	1150	129816.1	5.54	Voltage-de	CACNA2D	CACNA2D	Voltage-de	11	540.2594	50.43641	6.66E-58	11	10.26087
Q9H8G2	361	38367.4	4.34	Caspase ac	CAAP1	CAAP1	Caspase ac	6	633.6445	55.4849	6.79E-78	6	20.77562
Q9NZ63	289	33687.8	6.71	Splicing fa	C9orf78	C9orf78	Splicing fa	1	179.0878	42.35718	9.63E-32	1	4.844291
Q6P1X6	216	23889.1	9.29	UPF0598 p	C8orf82	C8orf82	UPF0598 p	3	113.9292	49.17053	3.07E-53	3	18.05556
Q96LL4	319	36789.2	8.94	Uncharact	C8orf48	C8orf48	Uncharact	1	351.2955	46.695	1.07E-44	1	2.507837
Q9H7E9	229	24992.3	10.66	UPF0488 p	C8orf33	C8orf33	UPF0488 p	3	267.5323	44.02203	2.13E-36	3	21.83406
Q9BRJ6	194	22083.2	10.33	Uncharact	C7orf50	C7orf50	Uncharact	3	320.3868	49.02222	1.13E-52	3	19.58763
Q5TEZ5	329	38553	6.96	Uncharact	C6orf163	C6orf163	Uncharact	1	119.0232	36.89728	2.84E-19	1	3.343465
Q7Z6I8	188	20131.6	10.44	UPF0461 p	C5orf24	C5orf24	UPF0461 p	4	474.8399	55.04139	4.36E-76	4	23.93617
Q5JPI3	329	37540.7	6.45	Uncharact	C3orf38	C3orf38	Uncharact	1	95.49431	26.39551	3.23E-07	1	4.559271
P01024	1663	187146.7	6.34	Compleme	C3	C3	Compleme	9	106.692	46.42627	8.09E-44	9	7.516536
Q86YS7	1000	110446	5.42	C2 domain	C2CD5	C2CD5	C2 domain	2	142.1564	39.28656	3.36E-24	2	2.2
Q9Y426	696	75532.5	6.92	C2 domain	C2CD2	C2CD2	C2 domain	1	73.54248	42.83295	4.94E-33	1	2.873563
Q07021	282	31361.9	4.47	Compleme	C1QBP	C1QBP	Compleme	4	1098.656	51.88943	1.91E-63	4	14.89362
A1L170	272	29057.1	4.93	Uncharact	C1orf226	C1orf226	Uncharact	2	526.7673	52.1668	1.6E-64	2	11.39706
Q8IYL3	243	25976.6	6.91	UPF0688 p	C1orf174	C1orf174	UPF0688 p	1	166.6879	48.58371	3.84E-51	1	5.761317
Q9UNZ5	99	10576.5	12.16	Leydig cell	C19orf53	C19orf53	Leydig cell	1	89.69468	37.06081	1.39E-19	1	9.090909
Q8N9M1	422	44745.8	10.85	Uncharact	C19orf47	C19orf47	Uncharact	1	143.2475	45.58711	3.88E-41	1	2.369668
Q32NC0	220	24826.5	10.97	UPF0711 p	C18orf21	C18orf21	UPF0711 p	2	163.6519	45.49419	7.6E-41	2	8.636364
Q9HAS0	396	44621.1	4.7	Protein Njr	C17orf75	C17orf75	Protein Njr	5	148.649	41.85233	2.08E-30	5	15.40404
E9PRG8	123	14233.6	12.07	Uncharact	C11orf98	C11orf98	Uncharact	4	2842.113	47.91103	8.54E-49	4	35.77236
Q9H3H3	292	31430	6.26	UPF0696 p	C11orf68	C11orf68	UPF0696 p	2	164.6542	41.56002	1.18E-29	2	8.219178
Q9Y6E2	419	48162	6.65	eIF5-mimi	BZW2	BZW2	eIF5-mimi	1	293.3022	42.90947	3.04E-33	1	2.147971
Q7L1Q6	419	48042.9	5.66	eIF5-mimi	BZW1	BZW1	eIF5-mimi	7	228.1569	46.24152	3.22E-43	7	17.18377
Q13895	437	49600.9	8.3	Bystin	BYSL	BYSL	Bystin	15	1130.33	52.82645	4.2E-67	15	43.47826
P41223	144	16999.6	9	Protein BU	BUD31	BUD31	Protein BU	6	320.2068	48.03404	3.2E-49	6	39.58333
O43709	281	31880.1	8.94	18S rRNA (	BUD23	BUD23	18S rRNA (	2	47.7249	36.76054	5.13E-19	2	10.32028
Q9BRD0	619	70520.1	10.52	BUD13 hor	BUD13	BUD13	BUD13 hor	10	812.4314	47.28927	1.12E-46	10	17.60905
O43684	328	37154.5	6.84	Mitotic che	BUB3	BUB3	Mitotic che	13	3160.25	52.28907	5.3E-65	13	50.91463
Q9Y297	605	68866.1	8.01	F-box/WD	BTRC	BTRC	F-box/WD	2	126.6331	47.80749	1.94E-48	2	3.801653
Q96Q07	612	69186.9	5.57	BTB/POZ c	BTBD9	BTBD9	BTB/POZ c	4	77.7164	50.75528	4.19E-59	4	7.843137
Q9BX70	525	55930.8	5.7	BTB/POZ c	BTBD2	BTBD2;BTE	BTB/POZ c	1	282.7747	53.07645	4.23E-68	1	2.285714
Q9BSF8	475	53778.7	7.84	BTB/POZ c	BTBD10	BTBD10	BTB/POZ c	1	163.4018	39.63807	5.39E-25	1	2.105263
O14981	1849	206885.6	6.49	TATA-binc	BTAF1	BTAF1	TATA-binc	8	145.9196	53.80078	5.42E-71	8	4.488913
Q10589	180	19768.7	5.3	Bone marr	BST2	BST2	Bone marr	3	179.301	48.16016	1.17E-49	3	18.33333
Q5W0U4	402	44380.8	6.39	B box and	BSPRY	BSPRY	B box and	3	48.3	40.83462	7.83E-28	3	10.69652
P35613	385	42200.1	5.4	Basigin	BSG	BSG	Basigin	6	1218.324	48.67648	1.8E-51	6	21.03896
Q6RI45	1802	203595.9	7.89	Bromodon	BRWD3	BRWD3	Bromodon	4	139.1084	50.0108	2.56E-56	4	2.27525
Q9NSI6	2320	262933.6	8.59	Bromodon	BRWD1	BRWD1	Bromodon	33	1565.706	52.86112	3.06E-67	29	16.12069
Q9ULD4	1205	135743.7	6.55	Bromodon	BRPF3	BRPF3	Bromodon	9	152.2559	50.47057	4.97E-58	5	7.717842
P55201	1214	137497.7	7.98	Peregrin	BRPF1	BRPF1	Peregrin	22	736.3207	53.72393	1.12E-70	22	20.01647
Q5VW32	411	46475.6	7.74	BRO1 dom	BROX	BROX	BRO1 dom	1	226.592	49.95799	4.05E-56	1	2.676399
Q5PSV4	323	37628.7	4.79	Breast can	BRMS1L	BRMS1L	Breast can	3	122.5508	49.17409	2.98E-53	3	9.907121
Q8WUW1	75	8744.8	5.12	Protein BR	BRK1	BRK1	Protein BR	1	116.1752	50.98937	5.39E-60	1	14.66667
Q8TDN6	353	41401	10.6	Ribosome	BRIX1	BRIX1	Ribosome	17	1179.67	53.1361	2.45E-68	17	52.9745
Q8WY22	251	27835.2	9.81	BRI3-bindi	BRI3BP	BRI3BP	BRI3-bindi	1	61.07031	49.74821	2.39E-55	1	6.772908
Q9H8M2	597	66999.5	6.11	Bromodon	BRD9	BRD9	Bromodon	1	275.7175	51.32293	2.87E-61	1	2.847571
Q9NPI1	651	74138	6.33	Bromodon	BRD7	BRD7	Bromodon	2	109.0134	44.47365	9.9E-38	2	3.379416
O60885	1362	152218	9.8	Bromodon	BRD4	BRD4	Bromodon	23	3098.593	50.15638	7.37E-57	13	17.32746
Q15059	726	79540.9	9.99	Bromodon	BRD3	BRD3	Bromodon	16	2758.922	56.71539	5.16E-83	14	27.82369
P25440	801	88060	9.65	Bromodon	BRD2	BRD2	Bromodon	32	19316.1	51.14732	1.35E-60	32	39.95006

O95696	1058	119518.9	8.72	Bromodon	BRD1	BRD1	Bromodon	25	957.3776	52.27296	6.08E-65	25	25.23629
P46736	316	36071.7	5.82	Lys-63-sp	BRCC3	BRCC3	Lys-63-sp	3	88.34974	48.83938	4.71E-52	3	12.34177
Q6PJG6	821	88118	4.92	BRCA1-ass	BRAT1	BRAT1	BRCA1-ass	3	56.44645	50.20229	4.98E-57	3	3.775883
P15056	766	84436.1	7.58	Serine/thre	BRAF	BRAF	Serine/thre	1	178.9409	33.90989	2.85E-14	1	1.436031
Q12830	3046	338260.3	6.48	Nucleoson	BPTF	BPTF	Nucleoson	19	323.7191	54.76484	6.13E-75	19	7.846356
O95861	308	33392	5.41	3(2),5-bisp	BPNT1	BPNT1	3'(2'),5'-bis	2	92.55301	51.24707	5.61E-61	2	9.090909
Q86WA6	291	32542.3	9.59	Valacyclov	BPHL	BPHL	Valacyclov	2	31.47789	19.89231	1.18E-05	2	9.621993
Q14137	746	83628.8	6.12	Ribosome	BOP1	BOP1	Ribosome	14	791.7496	54.97504	8.3E-76	14	19.57105
Q9H3K6	86	10116.5	6.51	BolA-like p	BOLA2	BOLA2	BolA-like p	4	351.1104	48.39805	1.73E-50	4	61.62791
Q14692	1282	145806.1	6.39	Ribosome	BMS1	BMS1	Ribosome	33	1133.201	52.08887	3.21E-64	33	29.48518
Q7Z5Y6	402	44798	8.88	Bone morp	BMP8A	BMP8A	Bone morp	1	191.3779	40.15133	3.46E-26	1	2.238806
P12644	408	46554.5	8.88	Bone morp	BMP4	BMP4	Bone morp	1	62.4758	47.54571	1.52E-47	1	2.941176
Q9NSY1	1161	129170.9	6.49	BMP-2-ind	BMP2K	BMP2K	BMP-2-ind	1	155.0006	42.42738	6.24E-32	1	1.29199
P13497	986	111247.8	6.89	Bone morp	BMP1	BMP1	Bone morp	2	100.3819	43.18664	5.18E-34	1	2.332657
P35226	326	36948.6	8.8	Polycomb	BMI1	BMI1	Polycomb	6	967.4954	50.79857	2.87E-59	6	28.83436
P30043	206	22119.2	7.76	Flavin redu	BLVRB	BLVRB	Flavin redu	2	91.15031	35.00246	5.95E-16	2	11.65049
P53004	296	33428.2	6.41	Biliverdin r	BLVRA	BLVRA	Biliverdin r	5	185.6682	47.56144	1.35E-47	5	16.55405
Q13867	455	52561.9	6.2	Bleomycin	BLMH	BLMH	Bleomycin	1	312.4608	37.80327	4.8E-21	1	2.417582
P54132	1417	158998.9	7.52	RecQ-like	BLM	BLM	RecQ-like	10	221.3218	48.01464	3.74E-49	10	8.609739
O15392	142	16388.6	5.71	Baculoviral	BIRC5	BIRC5	Baculoviral	2	106.3217	41.36837	3.64E-29	2	17.60563
Q13489	604	68370.9	5.92	Baculoviral	BIRC3	BIRC3;BIRC	Baculoviral	3	135.3352	36.63486	8.79E-19	3	5.960265
Q9NQY0	253	29664.7	7.66	Bridging ir	BIN3	BIN3	Bridging ir	2	32.96393	40.13826	3.72E-26	2	7.509881
Q6AI39	1079	115083.2	6.86	BRD4-inte	BICRAL	BICRAL	BRD4-inte	2	235.1341	46.0105	1.77E-42	2	2.31696
Q7RTS1	189	20818.3	11.8	Class A ba	BHLHA15	BHLHA15	Class A ba	2	135.0187	44.47044	1.01E-37	2	9.52381
Q02338	343	38156.8	9.24	D-beta-hy	BDH1	BDH1	D-beta-hy	3	147.4538	50.0253	2.27E-56	3	10.78717
P11274	1271	142818.1	7.03	Breakpoint	BCR	BCR;ABR	Breakpoint	1	174.6266	51.30025	3.52E-61	1	0.786782
Q5H9F3	1785	190559.5	7.91	BCL-6 core	BCORL1	BCORL1	BCL-6 core	1	92.73092	30.72039	2.47E-10	1	0.616246
A2AJT9	711	83871	10.23	BCLAF1 an	BCLAF3	BCLAF3	BCLAF1 an	5	136.858	48.84726	4.42E-52	5	8.298172
Q9NYF8	920	106121.2	10.61	Bcl-2-assc	BCLAF1	BCLAF1	Bcl-2-assc	24	3243.07	53.93177	1.58E-71	24	28.26087
Q8WUZ0	217	23467.9	4.86	B-cell CLL/	BCL7C	BCL7C	B-cell CLL/	2	301.5745	46.90411	2.18E-45	1	10.13825
P41182	706	78845.7	7.98	B-cell lymp	BCL6	BCL6	B-cell lymp	2	87.26208	37.11625	1.09E-19	1	2.549575
Q9BXK5	485	52722.6	4.1	Bcl-2-like	BCL2L13	BCL2L13	Bcl-2-like	1	51.38089	45.92972	2.85E-41	1	1.85567
Q9C0K0	894	95518.5	6.53	B-cell lymp	BCL11B	BCL11B	B-cell lymp	1	21.48903	31.27067	6.55E-11	1	1.677852
O14874	412	46360	9.06	Branched-	BCKDK	BCKDK	Branched-	9	1110.549	48.26479	5.06E-50	9	31.31068
P12694	445	50470.6	8.41	2-oxoisova	BCKDHA	BCKDHA	2-oxoisova	1	313.2343	36.89357	2.89E-19	1	3.146067
Q7Z5W3	292	33199.8	6.69	RNA 5-mo	BCDIN3D	BCDIN3D	RNA 5'-mo	2	85.22231	49.07069	6.98E-53	2	6.849315
Q9P287	314	35979.2	4.24	BRCA2 and	BCCIP	BCCIP	BRCA2 and	6	544.0876	49.6775	4.34E-55	6	19.10828
O75934	225	26131.3	5.41	Pre-mRNA	BCAS2	BCAS2	Pre-mRNA	5	278.5346	48.28421	4.33E-50	5	27.11111
P56945	870	93371.1	5.41	Breast canc	BCAR1	BCAR1	Breast canc	2	17.92567	42.07076	5.56E-31	2	2.413793
P51572	246	27991.4	8.89	B-cell rece	BCAP31	BCAP31	B-cell rece	5	481.7234	48.94035	2.05E-52	5	23.57724
P50895	628	67404.2	5.61	Basal cell a	BCAM	BCAM	Basal cell a	5	136.0309	49.10502	5.26E-53	5	10.82803
Q8WY36	941	105129.2	9.23	HMG box t	BBX	BBX	HMG box t	8	410.9233	47.74833	3.1E-48	8	8.820404
Q9UIF9	1905	211195.8	6.6	Bromodon	BAZ2A	BAZ2A	Bromodon	34	1627.925	55.29841	3.73E-77	34	21.31234
Q9UIG0	1483	170901.4	8.7	Tyrosine-p	BAZ1B	BAZ1B	Tyrosine-p	62	8442.457	55.33748	2.57E-77	62	43.08833
Q9NRL2	1556	178700.7	6.55	Bromodon	BAZ1A	BAZ1A	Bromodon	18	325.726	55.39179	1.64E-77	18	14.13882
Q8IXM2	172	17900.2	7.52	Chromatin	BAP18	BAP18	Chromatin	1	135.249	41.30084	5.4E-29	1	8.139535
Q92560	729	80360.9	6.83	Ubiquitin c	BAP1	BAP1	Ubiquitin c	2	37.51937	30.1207	9.42E-10	2	3.017833
O75531	89	10058.5	5.88	Barrier-to-	BANF1	BANF1	Barrier-to-	5	1161.258	50.06249	1.66E-56	5	42.69663
Q9UHR4	511	56882.1	8.99	BAR/IMD c	BAIAP2L1	BAIAP2L1	BAR/IMD c	4	76.44796	49.09906	5.52E-53	4	9.589041
Q9UQB8	552	60867.1	9.29	BAR/IMD c	BAIAP2	BAIAP2	BAR/IMD c	2	39.62605	38.67504	7.34E-23	2	4.166667
Q8TBE0	780	84651.2	9.34	Bromo adj	BAHD1	BAHD1	Bromo adj	1	407.7508	48.852	4.25E-52	1	1.666667
Q9P281	2639	280013.2	9.02	BAH and c	BAHCC1	BAHCC1	BAH and c	1	43.26291	43.8516	6.68E-36	1	0.795756
P46379	1132	119407.9	5.36	Large proli	BAG6	BAG6	Large proli	5	124.4235	43.71559	1.64E-35	5	6.007067
Q9UL15	447	51199.2	5.9	BAG family	BAG5	BAG5	BAG family	2	102.423	36.55635	1.23E-18	2	6.040268
O95816	211	23771.7	6.68	BAG family	BAG2	BAG2	BAG family	13	1652.401	52.24158	8.08E-65	13	56.87204
Q9NXR7	383	43551.3	5.62	BRISC and	BABAM2	BABAM2	BRISC and	2	104.2885	49.81424	1.37E-55	2	7.310705
O94766	335	37121.5	8.47	Galactosylt	B3GAT3	B3GAT3	Galactosylt	2	80.1395	41.02033	2.72E-28	1	4.776119
Q96L58	329	37137.1	9.94	Beta-1,3-g	B3GALT6	B3GALT6	Beta-1,3-g	1	106.7909	41.2915	5.7E-29	1	2.735562
P61769	119	13714.4	6.51	Beta-2-mi	B2M	B2M	Beta-2-mi	1	301.7308	53.39325	2.3E-69	1	8.403361
P25311	298	34258.5	5.93	Zinc-alpha	AZGP1	AZGP1	Zinc-alpha	2	100.5321	42.73537	9.14E-33	2	9.060403
O15169	862	95634.2	6.95	Axin-1	AXIN1	AXIN1	Axin-1	1	79.57363	46.47277	5.71E-44	1	1.740139
O75366	819	92026.6	5.41	Advillin	AVIL	AVIL	Advillin	1	21.90087	21.24582	7.25E-06	1	0.976801
Q96GD4	344	39310.2	9.7	Aurora kin	AURKB	AURKB	Aurora kin	9	337.3348	56.77556	3.06E-83	9	33.72093
Q9NWT8	199	22354.1	11.4	Small ribos	AURKAIP1	AURKAIP1	Small ribos	1	7464.018	46.44523	7.02E-44	1	5.025126
Q9Y679	410	45786.5	8.76	Lipid drop	AUP1	AUP1	Lipid drop	2	92.60503	48.12627	1.53E-49	2	5.121951
Q8WWM7	1075	113372.9	8.87	Ataxin-2-l	ATXN2L	ATXN2L	Ataxin-2-l	12	1111.836	49.23819	1.74E-53	12	14.32558
Q99700	1313	140281.9	10.02	Ataxin-2	ATXN2	ATXN2	Ataxin-2	6	346.3802	47.18515	2.51E-46	6	5.331302
P46100	2492	282584.6	6.5	Transcripti	ATRX	ATRX	Transcripti	55	1311.811	52.14235	2E-64	55	23.91653
Q8N5M1	289	32772.1	7.12	ATP syntha	ATPAF2	ATPAF2	ATP syntha	1	123.27	40.60027	2.92E-27	1	3.460208
Q9UI12	483	55882.7	6.44	V-type pro	ATP6V1H	ATP6V1H	V-type pro	4	205.3604	48.29828	3.86E-50	4	12.21532
O75348	118	13757.4	9.17	V-type pro	ATP6V1G1	ATP6V1G1	V-type pro	2	83.70863	47.3815	5.5E-47	2	16.94915
Q16864	119	13370.1	5.19	V-type pro	ATP6V1F	ATP6V1F	V-type pro	2	83.73013	51.79937	4.27E-63	2	30.2521
P36543	226	26145.1	8.37	V-type pro	ATP6V1E1	ATP6V1E1	V-type pro	3	165.6784	49.28732	1.16E-53	3	19.02655
Q9Y5K8	247	28262.5	10.01	V-type pro	ATP6V1D	ATP6V1D	V-type pro	3	98.68333	48.89776	2.91E-52	3	16.19433
P21283	382	43941.2	7.58	V-type pro	ATP6V1C1	ATP6V1C1	V-type pro	4	163.3711	52.27541	5.96E-65	4	12.30366
P21281	511	56500.2	5.55	V-type pro	ATP6V1B2	ATP6V1B2	V-type pro	11	281.0341	52.96083	1.22E-67	9	31.50685
P38606	617	68303.5	5.16	V-type pro	ATP6V1A	ATP6V1A	V-type pro	13	270.6928	49.18016	2.83E-53	13	29.01135
P61421	351	40328.7	4.63	V-type pro	ATP6V0D1	ATP6V0D1	V-type pro	7	1010.29	48.77388	8.09E-52	7	18.23362
P27449	155	15735.6	8.88	V-type pro	ATP6V0C	ATP6V0C	V-type pro	1	122.5749	45.77869	9.67E-42	1	11.6129
Q93050	837	96411.9	6.39	V-type pro	ATP6V0A1	ATP6V0A1	V-type pro	12	733.8982	50.12264	9.85E-57	11	16.96535
O75787	350	39007.6	5.98	Renin rece	ATP6AP2	ATP6AP2	Renin rece	2	72.17854	47.28368	1.17E-46	2	6.285714
Q15904	470	52025.3	6.05	V-type pro	ATP6AP1	ATP6AP1	V-type pro	2	167.7324	43.8448	6.98E-36	2	5.531915
P48047	213	23277.1	10.61	ATP syntha	ATP5PO	ATP5PO	ATP syntha	8	998.2748	50.40646	8.64E-58	8	48.35681
O75947	161	18491	4.94	ATP syntha	ATP5PD	ATP5PD	ATP syntha	3	203.6499	47.74083	3.28E-48	3	17.3913
P24539	256	28908.5	9.79	ATP syntha	ATP5PB	ATP5PB	ATP syntha	5	398.4655	50.24669	3.4E-57	5	22.65625
Q96IX5	58	6457.5	10.21	ATP syntha	ATP5MK	ATP5MK	ATP syntha	2	289.521	49.87247	8.36E-56	2	43.10345
O75964	103	11428.4	10.13	ATP syntha	ATP5MG	ATP5MG	ATP syntha	4	929.7399	47.53771	1.62E-47	4	46.60194
P56134	94	10917.8	10.09	ATP syntha	ATP5MF	ATP5MF	ATP syntha	2	442.8519	53.06256	4.77E-68	2	25.53191
P56385	69	7933.1	9.82	ATP syntha	ATP5ME	ATP5ME	ATP syntha	4	253.7556	42.39948	7.42E-32	4	60.86957
P30049	168	17489.8	5.19	ATP syntha	ATP5F1D	ATP5F1D	ATP syntha	1	416.0359	49.60424	8.08E-55	1	8.333333
P36542	298	32995.7	9.71	ATP syntha	ATP5F1C	ATP5F1C	ATP syntha	8	1106.562	50.6926	7.2E-59	8	28.52349
P06576	529	56559.4	5.07	ATP syntha	ATP5F1B	ATP5F1B	ATP syntha	17	5563.891	51.05707	2.97E-60	17	44.80151
P25705	553	59750.1	9.56	ATP syntha	ATP5F1A	ATP5F1A	ATP syntha	20	7293.579	53.34883	3.47E-69	20	42.49548

P98194	919	100576.4	6.72	Calcium-tr	ATP2C1	ATP2C1	Calcium-tr	2	57.4418	44.19383	6.69E-37	2	3.04679
P20020	1220	134683.6	5.68	Plasma me	ATP2B1	ATP2B1	Plasma me	8	177.0376	47.82655	1.67E-48	6	9.344262
Q93084	999	109255.1	5.19	Sarcoplasn	ATP2A3	ATP2A3	Sarcoplasn	1	68.20602	25.44059	7.76E-07	1	1.301301
P16615	1042	114755.8	4.99	Sarcoplasn	ATP2A2	ATP2A2	Sarcoplasn	18	1283.322	53.36047	3.12E-69	12	23.89635
P54709	279	31512.3	8.52	Sodium/pc	ATP1B3	ATP1B3	Sodium/pc	7	1104.241	47.30837	9.7E-47	7	31.1828
P05026	303	35061.1	8.73	Sodium/pc	ATP1B1	ATP1B1	Sodium/pc	6	659.6364	48.58059	3.94E-51	6	22.77228
P50993	1020	112264.4	5.33	Sodium/pc	ATP1A2	ATP1A2	Sodium/pc	1	276.6752	24.84114	1.22E-06		1.078431
P05023	1023	112895	5.15	Sodium/pc	ATP1A1	ATP1A1	Sodium/pc	34	3911.757	55.04212	4.36E-76	20	35.97263
Q9HD20	1204	132953.5	8.2	Endoplasm	ATP13A1	ATP13A1	Endoplasm	9	240.2991	51.02139	4.06E-60	9	9.966777
P98196	1134	129754.6	6.58	Phospholip	ATP11A	ATP11A	Phospholip	4	96.94936	40.67898	1.88E-27	4	3.968254
Q6DD88	541	60541.5	5.37	Atlastin-3	ATL3	ATL3	Atlastin-3	6	241.5068	48.16896	1.09E-49	6	16.26617
Q8NHH9	583	66228.7	5.15	Atlastin-2	ATL2	ATL2	Atlastin-2	1	175.1566	42.69367	1.19E-32	1	1.543739
Q8WXF7	558	63543	6.09	Atlastin-1	ATL1	ATL1	Atlastin-1	1	23.38536	19.23832	1.49E-05	1	1.792115
P31939	592	64615.3	6.7	Bifunctiona	ATIC	ATIC	Bifunctiona	3	156.017	35.80293	2.72E-17	3	6.25
Q9H1Y0	275	32447	5.57	Autophagy	ATG5	ATG5	Autophagy	3	71.54226	44.24802	4.63E-37	3	14.54545
Q9Y4P1	393	44293.9	4.69	Cysteine p	ATG4B	ATG4B	Cysteine p	3	106.5016	46.78474	5.42E-45	3	11.45038
Q9NT62	314	35864.2	4.38	Ubiquitin-	ATG3	ATG3	Ubiquitin-	2	38.25277	34.3486	6.33E-15	2	7.961783
P18850	670	74584.1	8.42	Cyclic AMF	ATF6	ATF6	Cyclic AMF	1	29.93585	22.85084	3.72E-06	1	1.343284
O95260	518	59089.8	8	Arginyl-tR	ATE1	ATE1	Arginyl-tR	3	85.94412	46.94314	1.62E-45	3	5.984556
Q5T2N8	411	46379.1	9.77	ATPase far	ATAD3C	ATAD3C	ATPase far	1	117.8611	41.23177	8.06E-29	1	1.703163
Q5T9A4	648	72572.3	9.64	ATPase far	ATAD3B	ATAD3B	ATPase far	3	569.9079	48.20892	7.91E-50	3	4.012346
Q9NVI7	634	71368.6	9.41	ATPase far	ATAD3A	ATAD3A	ATPase far	18	2071.555	51.63507	1.82E-62	6	31.8612
Q9ULI0	1458	164940.3	6.82	ATPase far	ATAD2B	ATAD2B	ATPase far	13	725.5441	50.17304	6.4E-57	10	10.28807
Q6PL18	1390	158552.5	6.25	ATPase far	ATAD2	ATAD2	ATPase far	37	801.7473	53.51612	7.47E-70	37	30.57554
Q8NBU5	361	40743.7	6.9	Outer mitc	ATAD1	ATAD1	Outer mitc	6	283.1353	46.67812	1.22E-44	6	20.22161
Q8IXJ9	1541	165430.3	6.12	Polycomb	ASXL1	ASXL1	Polycomb	1	54.51208	32.92156	6.76E-13	1	0.908501
P00966	412	46530.1	8.18	Argininosu	ASS1	ASS1	Argininosu	10	2095.386	52.53183	5.93E-66	10	23.05825
Q7L266	308	32054.3	6.17	Isoaspartyl	ASRGL1	ASRGL1	Isoaspartyl	1	82.54809	49.05348	8.05E-53	1	4.87013
Q9BZE9	553	60182.5	6.61	Tether con	ASPSCR1	ASPSCR1	Tether con	1	70.93918	53.09335	3.6E-68	1	2.893309
Q6ICH7	369	41698.4	7.53	Aspartate	ASPHD2	ASPHD2	Aspartate	3	116.6898	49.04624	8.54E-53	3	8.943089
Q12797	758	85862.1	4.65	Aspartyl/a:	ASPH	ASPH	Aspartyl/a:	18	2489.503	49.68434	4.1E-55	18	22.03166
P08243	561	64369.4	6.85	Asparagine	ASNS	ASNS	Asparagine	19	4232.985	53.21114	1.21E-68	19	40.99822
O95671	621	68856.1	5.98	Probable b	ASMTL	ASMTL	Probable b	3	56.43673	42.55082	2.9E-32	3	6.924316
Q9UBL3	628	68722.4	5.42	Set1/Ash2	ASH2L	ASH2L	Set1/Ash2	10	526.836	50.90815	1.1E-59	10	15.6051
Q9NR48	2969	332787.2	9.95	Histone-ly	ASH1L	ASH1L	Histone-ly	2	57.09263	35.09104	4.28E-16	2	0.808353
Q9Y294	204	22968.5	4.03	Histone ch	ASF1A	ASF1A	Histone ch	1	108.0701	32.51715	2.25E-12	1	5.392157
Q8N3C0	2202	251458.2	7.1	Activating	ASCC3	ASCC3	Activating	89	4095.325	52.98435	9.84E-68	89	46.54859
Q9H1I8	757	86359.4	4.78	Activating	ASCC2	ASCC2	Activating	17	4035.755	50.42799	7.17E-58	17	24.57067
Q8N9N2	400	45509.1	5.23	Activating	ASCC1	ASCC1	Activating	7	1117.624	52.79007	5.84E-67	7	17.5
Q9ULH1	1129	125496.9	7.34	Arf-GAP w	ASAP1	ASAP1	Arf-GAP w	1	360.3049	27.69834	6.66E-08	1	0.88574
Q13510	395	44659.3	7.7	Acid ceram	ASAH1	ASAH1	Acid ceram	3	219.0834	47.21044	2.07E-46	3	8.35443
O00192	962	104640.9	6.8	Splicing re	ARVCF	ARVCF	Splicing re	3	94.96812	40.00406	7.68E-26	3	4.054054
P54793	590	65939.1	7.22	Arylsulfata	ARSF	ARSF	Arylsulfata	1	18.31385	28.85086	1.1E-08	1	2.881356
Q8N5I2	433	45981	7.02	Arrestin dc	ARRDC1	ARRDC1	Arrestin dc	2	249.2368	40.71275	1.56E-27	2	5.773672
P32121	409	46105.4	7.77	Beta-arres	ARRB2	ARRB2	Beta-arres	5	655.5348	45.8358	6.38E-42	5	11.98044
P49407	418	47065.3	6.09	Beta-arres	ARRB1	ARRB1	Beta-arres	1	80.36956	43.77313	1.12E-35	1	2.392344
Q9BPX5	153	16941	6.55	Actin-relat	ARPC5L	ARPC5L	Actin-relat	3	88.37804	44.48652	9.07E-38	3	28.75817
O15511	151	16320.3	5.29	Actin-relat	ARPC5	ARPC5	Actin-relat	2	192.4453	43.10313	8.85E-34	2	16.55629
P59998	168	19666.8	8.71	Actin-relat	ARPC4	ARPC4	Actin-relat	5	445.9139	44.5515	5.82E-38	5	27.97619
O15145	178	20546.5	8.83	Actin-relat	ARPC3	ARPC3	Actin-relat	3	235.7388	40.1084	4.37E-26	3	17.41573
O15144	300	34332.7	7.4	Actin-relat	ARPC2	ARPC2	Actin-relat	9	420.1302	53.56156	4.95E-70	9	29
O15143	372	40949.4	8.44	Actin-relat	ARPC1B	ARPC1B	Actin-relat	5	669.5989	49.75929	2.18E-55	5	17.74194
Q9UH62	379	42500.2	8.55	Armadillo	ARMCX3	ARMCX3	Armadillo	5	187.7821	51.22402	6.86E-61	5	16.62269
Q8IUR7	673	75508.4	6.72	Armadillo	ARMC8	ARMC8	Armadillo	8	239.2624	51.59905	2.5E-62	8	13.81872
Q9NVJ2	186	21538.8	8.63	ADP-ribos	ARL8B	ARL8B	ADP-ribos	6	588.6654	48.22178	7.15E-50	2	41.93548
Q96BM9	186	21415.7	7.93	ADP-ribos	ARL8A	ARL8A	ADP-ribos	2	158.1376	52.96466	1.18E-67	2	13.44086
O75915	188	21614.5	10.28	PRA1 fami	ARL6IP5	ARL6IP5	PRA1 fami	3	146.4265	44.74255	1.55E-38	3	14.3617
Q66PJ3	237	26374.4	11.54	ADP-ribos	ARL6IP4	ARL6IP4	ADP-ribos	8	2690.415	51.78981	4.62E-63	8	39.66245
Q15041	203	23362.6	9.79	ADP-ribos	ARL6IP1	ARL6IP1	ADP-ribos	2	103.8243	46.21263	4E-43	2	8.866995
P36405	182	20455.3	7.35	ADP-ribos	ARL3	ARL3	ADP-ribos	2	363.5327	45.42097	1.29E-40	2	11.53846
P36404	184	20877.8	6.27	ADP-ribos	ARL2	ARL2	ADP-ribos	6	891.8434	48.08079	2.2E-49	6	35.32609
Q9NXU5	204	22875.9	5.32	ADP-ribos	ARL15	ARL15	ADP-ribos	1	71.12208	23.59671	2.58E-06	1	5.882353
Q8N8R7	260	29337.3	8.13	ARL14 effe	ARL14EP	ARL14EP	ARL14 effe	1	76.09329	48.26086	5.22E-50	1	6.153846
P40616	181	20417.4	5.36	ADP-ribos	ARL1	ARL1	ADP-ribos	4	574.2346	50.62349	1.32E-58	4	27.62431
Q96B23	404	43394.7	4.51	Protein AR	ARK2N	ARK2N	Protein AR	4	528.0354	47.82468	1.69E-48	4	13.86139
O95376	493	57818.5	5.38	E3 ubiquiti	ARIH2	ARIH2	E3 ubiquiti	3	80.95579	33.37319	1.66E-13	3	5.882353
Q9Y4X5	557	64117.4	4.72	E3 ubiquiti	ARIH1	ARIH1	E3 ubiquiti	8	487.2053	50.46159	5.37E-58	8	15.61939
Q4LE39	1312	147808.2	4.76	AT-rich int	ARID4B	ARID4B	AT-rich int	8	165.4252	48.98131	1.46E-52	7	7.926829
Q68CP9	1835	197389.8	7.43	AT-rich int	ARID2	ARID2	AT-rich int	8	199.7019	48.16358	1.14E-49	8	6.485014
Q8NFD5	2319	243941.9	6.9	AT-rich int	ARID1B	ARID1B	AT-rich int	6	139.259	42.66918	1.38E-32	5	3.967227
Q14155	803	90011.2	7.1	Rho guanii	ARHGEF7	ARHGEF7	Rho guanii	4	162.7805	50.80626	2.69E-59	3	5.603985
Q92974	986	111541.3	7.27	Rho guanii	ARHGEF2	ARHGEF2	Rho guanii	11	215.4979	53.945	1.41E-71	11	12.7789
Q9NZN5	1544	173231	5.51	Rho guanii	ARHGEF12	ARHGEF12	Rho guanii	13	337.9926	52.70181	1.29E-66	13	10.10363
Q92888	912	102434.3	5.37	Rho guanii	ARHGEF1	ARHGEF1	Rho guanii	8	130.2325	52.49164	8.47E-66	8	11.62281
P52565	204	23206.9	4.74	Rho GDP-c	ARHGDIA	ARHGDIA	Rho GDP-c	1	646.7407	48.84415	4.54E-52	1	7.352941
Q52LW3	1261	142062.2	6.72	Rho GTPas	ARHGAP29	ARHGAP29	Rho GTPas	3	47.64692	51.5213	4.95E-62	3	2.616971
Q6ZUM4	889	98395.1	5.3	Rho GTPas	ARHGAP27	ARHGAP27	Rho GTPas	1	51.18843	46.83606	3.67E-45	1	1.349831
Q9UNA1	814	92234.2	6.62	Rho GTPas	ARHGAP26	ARHGAP26	Rho GTPas	1	111.819	49.81681	1.34E-55	1	1.228501
Q8IWW6	846	96253.8	7.7	Rho GTPas	ARHGAP12	ARHGAP12	Rho GTPas	1	70.5396	38.55067	1.35E-22	1	1.06383
A1A4S6	786	89373.9	7.2	Rho GTPas	ARHGAP10	ARHGAP10	Rho GTPas	4	80.56886	33.45573	1.27E-13	4	5.343511
Q07960	439	50435.3	6.24	Rho GTPas	ARHGAP1	ARHGAP1	Rho GTPas	2	96.22241	50.74427	4.61E-59	2	5.23918
Q9NWB6	273	33216	11.1	Arginine a	ARGLU1	ARGLU1	Arginine a	7	1631.627	50.03534	2.08E-56	7	19.78022
P78540	354	38577.5	6.45	Arginase-2	ARG2	ARG2	Arginase-2	3	84.19401	50.43839	6.55E-58	3	11.01695
P05089	322	34734.7	7.25	Arginase-1	ARG1	ARG1	Arginase-1	6	263.6165	52.21404	1.03E-64	6	20.49689
P53365	341	37855.5	5.91	Arfaptin-2	ARFIP2	ARFIP2	Arfaptin-2	2	95.48803	34.86987	9.72E-16	2	5.278592
P53367	373	41738	6.69	Arfaptin-1	ARFIP1	ARFIP1	Arfaptin-1	3	138.6574	43.40025	1.3E-34	3	10.45576
Q8N6H7	521	56719.9	8.13	ADP-ribos	ARFGAP2	ARFGAP2;	ADP-ribos	1	129.8115	26.74229	2.22E-07	1	1.919386
Q8N6T3	406	44667.6	5.34	ADP-ribos	ARFGAP1	ARFGAP1	ADP-ribos	3	83.76948	43.35759	1.72E-34	3	10.83744
P62330	175	20082	9.32	ADP-ribos	ARF6	ARF6	ADP-ribos	3	178.5456	48.95455	1.82E-52	3	21.14286
P84085	180	20529.5	6.79	ADP-ribos	ARF5	ARF5	ADP-ribos	4	293.9162	54.36923	2.75E-73	4	26.11111
P18085	180	20510.6	7.25	ADP-ribos	ARF4	ARF4	ADP-ribos	6	1462.198	45.89999	3.99E-42	6	31.66667
P61204	181	20600.6	7.59	ADP-ribos	ARF3	ARF3;ARF1	ADP-ribos	8	1758.001	49.67131	4.58E-55	4	43.09392
P15514	252	27895.1	7.46	Amphiregu	AREG	AREG	Amphiregu	4	70.51357	46.70086	1.03E-44	4	11.50794

P48444	511	57210	6.11	Coatomer	ARCN1	ARCN1	Coatomer	15	553.015	49.93179	5.06E-56	15	30.13699
Q8WUF8	416	47972	5.98	Cotranscri	ARB2A	ARB2A	Cotranscri	5	399.8008	48.21117	7.77E-50	5	13.94231
P10398	606	67584.8	9.3	Serine/thre	ARAF	ARAF	Serine/thre	4	234.4167	45.29816	3.1E-40	2	6.930693
O60306	1485	171293.9	6.33	RNA helica	AQR	AQR	RNA helica	22	680.6534	51.92877	1.35E-63	22	17.71044
Q7Z2E3	356	40739.8	9.68	Aprataxin	APTX	APTX	Aprataxin	6	1041.1	49.0379	9.15E-53	6	23.03371
P07741	180	19607.5	5.82	Adenine p	APRT	APRT	Adenine p	5	489.2011	51.54002	4.2E-62	5	38.33333
Q8NEU8	664	74492.7	4.59	DCC-intera	APPL2	APPL2	DCC-intera	2	119.4487	44.01647	2.21E-36	2	4.216867
P05067	770	86942.7	4.45	Amyloid-b	APP	APP	Amyloid-b	6	140.3795	51.41921	1.23E-61	6	10
Q6UXV4	268	29158.7	10.07	MICOS cor	APOOL	APOOL	MICOS cor	1	132.2155	53.40952	1.98E-69	1	7.089552
Q9BUR5	198	22284.5	9.49	MICOS cor	APOO	APOO	MICOS cor	2	207.0717	48.30457	3.67E-50	2	13.13131
Q9BQE5	337	37078.1	6.73	Apolipopro	APOL2	APOL2	Apolipopro	1	513.7536	32.0386	8.72E-12	1	2.670623
Q9HC16	384	46407.6	8.06	DNA dC->	APOBEC3C	APOBEC3C	DNA dC->	10	235.8603	50.25234	3.24E-57	10	27.86458
Q8IUX4	373	45019.8	7.24	DNA dC->	APOBEC3F	APOBEC3F	DNA dC->	11	4883.886	48.75263	9.63E-52	4	28.95442
Q9NRW3	190	22825.7	7.68	DNA dC->	APOBEC3C	APOBEC3C	DNA dC->	3	536.327	49.26795	1.36E-53	3	22.63158
Q9UH17	382	45923.9	5.94	DNA dC->	APOBEC3E	APOBEC3E	DNA dC->	6	609.9552	54.48857	9.26E-74	1	15.44503
P02652	100	11174.9	6.59	Apolipopro	APOA2	APOA2	Apolipopro	2	55.58978	42.58951	2.28E-32	2	21
P02647	267	30777.6	5.5	Apolipopro	APOA1	APOA1	Apolipopro	8	351.6447	47.73523	3.43E-48	8	30.71161
Q9HDC9	416	46479.9	6.02	Adipocyte	APMAP	APMAP	Adipocyte	10	1019.525	48.75652	9.33E-52	10	30.04808
Q06481	763	86955.2	4.44	Amyloid b	APLP2	APLP2	Amyloid b	16	1942.998	53.22494	1.07E-68	16	24.77064
Q8IW19	511	56955.4	4.72	Aprataxin	APLF	APLF	Aprataxin	5	467.7271	50.89101	1.28E-59	5	11.93738
Q9BZZ5	524	59004.1	7.42	Apoptosis	API5	API5	Apoptosis	9	873.0897	50.0804	1.42E-56	9	19.08397
P27695	318	35554.2	8.26	DNA repai	APEX1	APEX1	DNA repai	9	753.5614	52.84267	3.63E-67	9	32.38994
P13798	732	81223.9	5.16	Acylamino	APEH	APEH	Acylamino	2	63.54887	28.84096	1.12E-08	2	4.781421
Q92572	193	21731.9	5.06	AP-3 com	AP3S1	AP3S1	AP-3 com	3	667.2125	47.6567	6.37E-48	3	15.54404
P53677	418	46977	7.66	AP-3 com	AP3M2	AP3M2	AP-3 com	5	740.5967	52.37413	2.44E-65	3	15.07177
Q9Y2T2	418	46938.8	6.94	AP-3 com	AP3M1	AP3M1	AP-3 com	3	515.523	50.492	4.15E-58	3	8.61244
O14617	1153	130156.8	8.7	AP-3 com	AP3D1	AP3D1	AP-3 com	22	367.802	54.74968	7.11E-75	22	23.07025
O00203	1094	121319	5.86	AP-3 com	AP3B1	AP3B1	AP-3 com	30	1483.749	59.25273	1.47E-93	24	27.4223
P53680	142	17017.6	6.11	AP-2 com	AP2S1	AP2S1	AP-2 com	1	337.0185	36.461	1.83E-18	1	5.633803
Q96CW1	435	49654.3	10.09	AP-2 com	AP2M1	AP2M1	AP-2 com	9	417.9115	47.8711	1.17E-48	9	24.36782
P63010	937	104551.6	5.02	AP-2 com	AP2B1	AP2B1	AP-2 com	16	1112.411	53.85123	3.38E-71	7	19.74386
O94973	939	103959.4	6.96	AP-2 com	AP2A2	AP2A2	AP-2 com	3	127.347	41.68492	5.65E-30	3	3.72737
O95782	977	107544.7	7.04	AP-2 com	AP2A1	AP2A1	AP-2 com	26	936.0758	49.88435	7.57E-56	20	29.6827
Q96PC3	154	18280.1	6.78	AP-1 com	AP1S3	AP1S3	AP-1 com	1	122.3412	51.93214	1.31E-63	1	5.844156
Q9Y6Q5	423	48108.1	8.52	AP-1 com	AP1M2	AP1M2	AP-1 com	4	117.9522	47.60632	9.47E-48	4	11.34752
Q9BXS5	423	48586.5	7.34	AP-1 com	AP1M1	AP1M1	AP-1 com	7	534.2906	52.13024	2.23E-64	5	18.43972
O75843	785	87115.6	6.54	AP-1 com	AP1G2	AP1G2	AP-1 com	1	53.98183	17.80811	2.46E-05	1	2.165605
O43747	822	91350.5	6.78	AP-1 com	AP1G1	AP1G1	AP-1 com	6	170.0275	49.549	1.29E-54	6	7.542579
Q10567	949	104605.6	4.68	AP-1 com	AP1B1	AP1B1	AP-1 com	2	158.2213	40.96911	3.64E-28	2	2.107482
P20073	488	52738.9	5.32	Annexin A	ANXA7	ANXA7	Annexin A	5	203.4821	49.82757	1.23E-55	5	12.29508
P08133	673	75872.5	5.28	Annexin A	ANXA6	ANXA6	Annexin A	28	5630.808	50.91817	1.01E-59	28	45.31947
P08758	320	35936.4	4.66	Annexin A	ANXA5	ANXA5	Annexin A	7	606.854	46.51539	4.15E-44	7	25
P09525	319	35882.4	5.9	Annexin A	ANXA4	ANXA4	Annexin A	3	163.1898	47.65752	6.33E-48	3	10.65831
P12429	323	36374.9	5.71	Annexin A	ANXA3	ANXA3	Annexin A	1	60.79023	44.49154	8.76E-38	1	5.882353
P07355	339	38603.6	7.91	Annexin A	ANXA2	ANXA2	Annexin A	25	24209.91	52.57919	3.87E-66	6	63.71681
P50995	505	54389.3	7.77	Annexin A	ANXA11	ANXA11	Annexin A	5	305.9451	50.66734	8.98E-59	5	10.69307
P04083	346	38713.9	7.04	Annexin A	ANXA1	ANXA1	Annexin A	8	353.5777	51.61976	2.07E-62	8	35.83815
Q9BTT0	268	30692.2	3.49	Acidic leuc	ANP32E	ANP32E	Acidic leuc	4	193.6118	50.52746	3.05E-58	4	19.77612
O43423	234	26761.6	3.86	Putative ur	ANP32CP	ANP32CP	Putative ur	1	26.55362	17.49973	2.73E-05	1	2.991453
Q92688	251	28787.4	3.67	Acidic leuc	ANP32B	ANP32B	Acidic leuc	5	11179.18	52.49847	7.95E-66	5	20.31873
P39687	249	28585.1	3.72	Acidic leuc	ANP32A	ANP32A	Acidic leuc	8	2703.107	47.30969	9.6E-47	3	28.11245
Q9NQW6	1124	124197.6	8.17	Anillin	ANLN	ANLN	Anillin	2	217.4265	46.19292	4.63E-43	2	1.690391
Q9H8Y5	726	80925.9	8.53	tRNA endo	ANKZF1	ANKZF1	tRNA endo	7	407.9908	51.23621	6.17E-61	7	11.70799
Q9ULJ7	1429	155857.9	6.58	Ankyrin re	ANKRD50	ANKRD50	Ankyrin re	2	44.26867	31.92154	1.2E-11	2	1.819454
O15084	1053	112964.6	6.22	Serine/thre	ANKRD28	ANKRD28	Serine/thre	1	144.0374	41.55187	1.24E-29	1	1.804368
O75179	2603	274255.5	6.49	Ankyrin re	ANKRD17	ANKRD17	Ankyrin re	6	228.0632	52.53628	5.7E-66	3	2.420284
Q6UB99	2663	297910.5	7.1	Ankyrin re	ANKRD11	ANKRD11	Ankyrin re	7	175.9624	51.78053	5.02E-63	7	2.891476
Q8IWZ3	2542	269455.3	5.52	Ankyrin re	ANKHD1	ANKHD1	Ankyrin re	3	64.64471	45.80586	7.94E-42	3	1.966955
Q9P2R3	1169	128397.9	6.03	Rabankyrin	ANKFY1	ANKFY1	Rabankyrin	3	59.33028	33.48507	1.16E-13	3	2.994012
Q12955	4377	480406	6.46	Ankyrin-3	ANK3	ANK3	Ankyrin-3	4	221.7834	41.90258	1.54E-30	4	1.210875
Q9BY76	406	45213.8	9.08	Angiopoie	ANGPTL4	ANGPTL4	Angiopoie	1	143.1042	48.16878	1.09E-49	1	2.70936
Q5VTE6	544	62338.5	7.86	Protein an	ANGEL2	ANGEL2	Protein an	3	272.4201	42.6858	1.25E-32	3	7.720588
Q9UNK9	670	75274.9	4.45	Protein an	ANGEL1	ANGEL1	Protein an	1	107.734	40.87624	6.18E-28	1	2.238806
Q9UJX3	565	63132.5	5.43	Anaphase-	ANAPC7	ANAPC7	Anaphase-	3	321.3561	42.70119	1.13E-32	3	6.19469
Q9UJX4	755	85076.2	6.86	Anaphase-	ANAPC5	ANAPC5	Anaphase-	3	59.67675	46.75288	6.9E-45	3	5.165563
Q9UJX5	808	92115.5	5.21	Anaphase-	ANAPC4	ANAPC4	Anaphase-	2	95.13916	35.99619	1.25E-17	2	2.475248
Q9UM13	185	21252	9.41	Anaphase-	ANAPC10	ANAPC10	Anaphase-	1	100.3683	42.99146	1.8E-33	1	6.486486
Q9H1A4	1944	216498	6.27	Anaphase-	ANAPC1	ANAPC1	Anaphase-	5	77.95854	44.94116	3.87E-39	5	2.726337
Q01433	825	94889.3	6.54	AMP deam	AMPD2	AMPD2	AMP deam	22	1962.01	52.86147	3.05E-67	22	26.18182
Q8IY63	956	106573.5	7.1	Angiomoti	AMOTL1	AMOTL1	Angiomoti	1	27.05574	20.23489	1.05E-05	1	1.464435
Q8IY45	258	28408	7.91	Protein AM	AMN1	AMN1	Protein AM	1	165.5992	40.61123	2.75E-27	1	4.263566
Q6DCA0	310	34499	9.21	AMMECR1	AMMECR1	AMMECR1	AMMECR1	1	98.03408	50.15707	7.33E-57	1	5.483871
Q9UKV5	643	72995.1	6.36	E3 ubiquiti	AMFR	AMFR	E3 ubiquiti	1	28.40355	40.68391	1.83E-27	1	2.021773
Q86V81	257	26887.7	11.7	THO comp	ALYREF	ALYREF	THO comp	7	4472.416	48.26413	5.08E-50	7	35.40856
Q60I27	953	107747.5	6.09	ALS2 C-ter	ALS2CL	ALS2CL	ALS2 C-ter	1	143.0947	22.16709	5.02E-06	1	0.734523
P05187	535	57953.3	6.24	Alkaline ph	ALPP	ALPP;ALPG	Alkaline ph	2	185.8543	41.73315	4.24E-30	2	4.672897
Q6P6C2	394	44255.4	9.44	RNA deme	ALKBH5	ALKBH5	RNA deme	9	643.0368	56.84308	1.78E-83	9	28.4264
Q96Q83	286	33374.5	8.58	Alpha-keto	ALKBH3	ALKBH3	Alpha-keto	3	156.9714	47.83662	1.54E-48	3	13.98601
Q6NS38	261	29322.2	10.22	DNA oxida	ALKBH2	ALKBH2	DNA oxida	5	243.4542	46.96452	1.37E-45	5	26.05364
Q9Y673	324	36945.8	9.79	Dolichyl-p	ALG5	ALG5	Dolichyl-p	2	211.3654	46.59264	2.32E-44	2	7.716049
Q9H553	416	47091.1	7.05	Alpha-1,3/	ALG2	ALG2	Alpha-1,3/	3	97.70856	42.36136	9.39E-32	3	7.932692
Q9BT22	464	52517.7	7.24	Chitobiosy	ALG1	ALG1	Chitobiosy	3	100.2879	51.4535	9.05E-62	3	7.327586
P04075	364	39419.7	8.2	Fructose-b	ALDOA	ALDOA	Fructose-b	12	817.9162	50.7053	6.48E-59	11	37.36264
P49189	494	53801.5	5.61	4-trimethy	ALDH9A1	ALDH9A1	4-trimethy	4	214.939	47.41997	4.07E-47	4	10.32389
P49419	539	58486.7	8.09	Alpha-ami	ALDH7A1	ALDH7A1	Alpha-ami	5	158.2841	50.84086	1.98E-59	5	11.87384
P30038	563	61718.9	8.2	Delta-1-py	ALDH4A1	ALDH4A1	Delta-1-py	3	164.9894	44.5683	5.19E-38	3	7.104796
P51648	485	54847.4	7.99	Aldehyde	ALDH3A2	ALDH3A2	Aldehyde	6	431.3586	53.69836	1.42E-70	5	14.63918
P05091	517	56380.9	7.05	Aldehyde	ALDH2	ALDH2	Aldehyde	7	481.4842	49.75284	2.3E-55	7	15.66731
P30837	517	57249	6.99	Aldehyde	ALDH1B1	ALDH1B1	Aldehyde	5	211.8846	35.62493	5.5E-17	5	13.1528
P47895	512	56108	7.29	Retinaldeh	ALDH1A3	ALDH1A3	Retinaldeh	17	572.8535	52.38436	2.24E-65	16	37.5
P54886	795	87301.5	7.13	Delta-1-py	ALDH18A1	ALDH18A1	Delta-1-py	25	1007.785	53.07551	4.26E-68	25	37.2327
Q13740	583	65101.7	6.15	CD166 ant	ALCAM	ALCAM	CD166 ant	7	969.0768	46.00571	1.84E-42	7	12.69297
P02768	609	69365.9	6.21	Albumin	ALB	ALB	Albumin	42	5828.8	51.96684	9.64E-64	42	65.84565

P13196	640	70580.3	8.57	5-aminole	ALAS1	ALAS1	5-aminole	1	27.85647	24.20757	1.83E-06	1	2.34375
O43488	359	39588.7	7.18	Aflatoxin B	AKR7A2	AKR7A2	Aflatoxin B	4	153.8169	45.11737	1.12E-39	1	13.09192
Q9ULX6	646	71639.3	4.68	A-kinase a	AKAP8L	AKAP8L	A-kinase a	10	996.1923	53.4288	1.66E-69	10	19.96904
O43823	692	76107.7	4.77	A-kinase a	AKAP8	AKAP8	A-kinase a	10	364.144	51.62542	1.98E-62	10	16.18497
Q02040	695	80734.7	10.43	A-kinase a	AKAP17A	AKAP17A	A-kinase a	8	417.3295	49.13476	4.12E-53	8	11.22302
Q12802	2813	307547.7	4.88	A-kinase a	AKAP13	AKAP13	A-kinase a	8	448.3698	52.24406	7.93E-65	8	3.199431
Q02952	1782	191480.6	4.07	A-kinase a	AKAP12	AKAP12	A-kinase a	14	236.0484	47.79416	2.15E-48	14	11.84063
Q92667	903	97340.6	4.58	A-kinase a	AKAP1	AKAP1	A-kinase a	5	138.467	51.86102	2.46E-63	5	7.86268
Q9Y3D8	172	20061.3	4.21	Adenylate	AK6	AK6	Adenylate	1	149.0354	42.9852	1.88E-33	1	5.232558
P27144	223	25267.8	8.66	Adenylate	AK4	AK4	Adenylate	3	340.3473	46.54944	3.21E-44	3	17.48879
Q9UIJ7	227	25565.2	9.64	GTP:AMP p	AK3	AK3	GTP:AMP p	2	148.7998	30.10171	9.81E-10	2	10.57269
P54819	239	26477.4	7.97	Adenylate	AK2	AK2	Adenylate	2	138.9184	39.38913	1.98E-24	2	8.786611
P00568	194	21634.7	8.99	Adenylate	AK1	AK1	Adenylate	3	241.4568	47.57983	1.17E-47	3	19.07216
Q9NX04	203	23372.6	5.36	AFG2-inte	AIRIM	AIRIM	AFG2-inte	3	224.5879	49.11149	4.99E-53	3	22.6601
O00170	330	37663.8	6.38	AH recept	AIP	AIP	AH recept	2	183.2385	51.06273	2.83E-60	2	7.575758
Q13155	320	35348.5	8.37	Aminoacyl	AIMP2	AIMP2	Aminoacyl	4	266.0691	48.15553	1.21E-49	4	18.125
Q12904	312	34352.4	8.7	Aminoacyl	AIMP1	AIMP1	Aminoacyl	6	241.8509	47.93645	6.99E-49	6	27.5641
Q9BRQ8	373	40526.4	9.55	Ferroptosis	AIFM2	AIFM2	Ferroptosis	2	232.2874	49.18139	2.8E-53	2	7.238606
O95831	613	66900.1	9.39	Apoptosis-	AIFM1	AIFM1	Apoptosis-	16	546.2325	53.83709	3.82E-71	16	36.37847
Q9BQI0	150	17067.7	7.37	Allograft ir	AIF1L	AIF1L	Allograft ir	1	73.13206	31.89955	1.27E-11	1	6.666667
O95433	338	38274.1	5.22	Activator c	AHSA1	AHSA1	Activator c	5	842.2239	52.48122	9.3E-66	5	16.56805
P35869	848	96146.7	6.34	Aryl hydro	AHR	AHR	Aryl hydro	3	182.428	52.79849	5.41E-67	3	4.009434
Q8IVF2	5795	616624.7	4.98	Protein AH	AHNAK2	AHNAK2	Protein AH	6	117.9898	50.17063	6.53E-57	6	3.537532
Q09666	5890	629098.1	5.99	Neuroblas	AHNAK	AHNAK	Neuroblas	126	5110.76	52.95307	1.31E-67	126	38.86248
Q5TGY3	1603	168347.5	9.34	Transcripti	AHDC1	AHDC1	Transcripti	11	312.6997	54.40896	1.87E-73	11	8.296943
Q96HN2	611	66720.6	7.39	Adenosylh	AHCYL2	AHCYL2	Adenosylh	2	63.42149	49.30518	1E-53	2	4.909984
O43865	530	58950.9	6.87	S-adenosy	AHCYL1	AHCYL1	S-adenosy	12	405.4184	55.19935	9.55E-77	2	24.33962
P23526	432	47715.7	6.29	Adenosylh	AHCY	AHCY	Adenosylh	11	961.8242	51.3245	2.83E-61	11	25.46296
Q8WYP5	2266	252496	6.56	Protein EL	AHCTF1	AHCTF1	Protein EL	49	3842.053	54.96555	8.94E-76	49	24.09532
Q9UPW5	1226	138447.1	6.06	Cytosolic c	AGTPBP1	AGTPBP1	Cytosolic c	2	32.02258	30.74938	2.31E-10	2	1.386623
O00116	658	72911.2	7.37	Alkyldihyd	AGPS	AGPS	Alkyldihyd	10	312.5624	49.60516	8.02E-55	10	19.45289
Q9NUQ2	364	42071.8	9.41	1-acyl-sn-	AGPAT5	AGPAT5	1-acyl-sn-	5	375.4915	50.67312	8.55E-59	5	15.10989
Q9NRZ5	378	44020.9	8.9	1-acyl-sn-	AGPAT4	AGPAT4	1-acyl-sn-	3	195.2854	50.87961	1.42E-59	3	7.142857
O15120	278	30914.1	9.22	1-acyl-sn-	AGPAT2	AGPAT2	1-acyl-sn-	1	99.27829	36.56653	1.18E-18	1	2.877698
Q99943	283	31716.3	9.75	1-acyl-sn-	AGPAT1	AGPAT1	1-acyl-sn-	1	159.3595	30.16237	8.62E-10	1	3.180212
Q9UKV8	859	97207.5	9.54	Protein arg	AGO2	AGO2	Protein arg	8	307.9706	45.22901	5.06E-40	8	13.03842
Q9UL18	857	97213.7	9.48	Protein arg	AGO1	AGO1	Protein arg	11	552.0016	48.14099	1.36E-49	4	18.66978
P35573	1532	174762.3	6.74	Glycogen d	AGL	AGL	Glycogen d	4	67.76498	43.82113	8.17E-36	4	2.872063
Q53H12	422	47136.8	8.21	Acylglycer	AGK	AGK	Acylglycer	5	367.527	51.55106	3.81E-62	5	13.27014
Q8N302	714	80976.8	5.11	Angiogeni	AGGF1	AGGF1	Angiogeni	2	141.9356	34.67449	1.98E-15	2	2.941176
Q96P47	875	95043.2	8.05	Arf-GAP w	AGAP3	AGAP3	Arf-GAP w	5	360.3242	51.97659	8.86E-64	5	6.971429
Q99490	1192	124672.3	10.54	Arf-GAP w	AGAP2	AGAP2	Arf-GAP w	1	38.10487	45.22039	5.38E-40	1	1.426174
Q9UPQ3	857	94469.2	8.01	Arf-GAP w	AGAP1	AGAP1	Arf-GAP w	9	620.8524	50.67262	8.58E-59	7	13.53559
Q9Y4W6	797	88583	8.98	Mitochonc	AFG3L2	AFG3L2	Mitochonc	13	972.7221	50.38945	9.99E-58	13	17.06399
Q9BVQ7	753	80709.3	8.17	ATPase far	AFG2B	AFG2B	ATPase far	12	660.772	51.77783	5.12E-63	12	19.78752
Q8NB90	893	97903.4	5.33	ATPase far	AFG2A	AFG2A	ATPase far	22	1270.786	52.378	2.37E-65	21	30.34714
Q9UHB7	1163	127458.1	9.91	AF4/FMR2	AFF4	AFF4	AF4/FMR2	22	1359.465	55.7812	3.82E-79	22	24.16165
P51825	1210	131420.3	9.8	AF4/FMR2	AFF1	AFF1	AF4/FMR2	11	275.3393	47.82597	1.68E-48	11	10.33058
P55196	1824	206802.4	6.41	Afadin	AFDN	AFDN	Afadin	5	112.9946	46.5919	2.33E-44	5	3.673246
Q8N556	730	80724.2	8.95	Actin filam	AFAP1	AFAP1	Actin filam	4	137.6076	44.9234	4.38E-39	4	5.616438
Q6ZN18	517	54466.1	4.86	Zinc finger	AEBP2	AEBP2	Zinc finger	1	258.9312	56.66223	8.42E-83	1	2.321083
P30520	456	50097.1	6.52	Adenylosu	ADSS2	ADSS2	Adenylosu	1	81.17268	41.46092	2.12E-29	1	3.070175
P30566	484	54888.7	7.12	Adenylosu	ADSL	ADSL	Adenylosu	2	52.25971	39.12293	7.77E-24	2	6.198347
Q16186	407	42152.9	4.7	Proteasom	ADRM1	ADRM1	Proteasom	1	162.3089	49.1194	4.67E-53	1	3.931204
Q9NX46	363	38946.3	4.7	ADP-ribos	ADPRS	ADPRS	ADP-ribos	2	86.94939	48.22715	6.85E-50	2	6.887052
Q9BRR6	497	54088.3	6.17	ADP-depe	ADPGK	ADPGK	ADP-depe	1	39.41092	37.30433	4.7E-20	1	2.816901
Q96SZ5	270	29750.5	5.91	2-aminoet	ADO	ADO	2-aminoet	3	148.4627	46.91731	1.97E-45	3	12.59259
Q6IQ32	1131	122832.4	9.57	Activity-de	ADNP2	ADNP2	Activity-de	8	330.1057	52.27912	5.77E-65	8	8.311229
Q9H2P0	1102	123562	7.35	Activity-de	ADNP	ADNP	Activity-de	37	859.4638	54.19933	1.27E-72	37	39.01996
Q9GZN8	174	19290.9	6.84	Adipose-s	ADISSP	ADISSP	Adipose-s	2	175.8085	43.16092	6.11E-34	2	13.7931
P48960	835	91868.4	6.87	Adhesion (	ADGRE5	ADGRE5	Adhesion (	1	58.09665	26.19614	3.95E-07	1	1.556886
Q9UEY8	706	79154.1	6.26	Gamma-ad	ADD3	ADD3	Gamma-ad	1	282.7928	44.93139	4.14E-39	1	1.558074
P35611	737	80954.5	5.61	Alpha-adc	ADD1	ADD1	Alpha-adc	2	90.74281	28.26021	2.9E-08	2	2.985075
P78563	741	80762.7	9.45	Double-str	ADARB1	ADARB1	Double-str	10	398.5233	50.85493	1.75E-59	10	15.78947
P55265	1226	136065.3	8.86	Double-str	ADAR	ADAR	Double-str	26	1137.544	52.71601	1.13E-66	26	25.61175
Q13443	819	90555.3	7.55	Disintegrin	ADAM9	ADAM9	Disintegrin	2	36.03692	46.72759	8.37E-45	2	3.174603
Q9BZ11	813	87738.2	6.97	Disintegrin	ADAM33	ADAM33	Disintegrin	1	196.6992	25.79686	5.73E-07	1	1.353014
O14672	748	84141.5	7.81	Disintegrin	ADAM10	ADAM10	Disintegrin	6	234.7771	47.15563	3.16E-46	6	10.02674
Q9H981	624	70483.1	7.85	Actin-relat	ACTR8	ACTR8	Actin-relat	6	227.7677	49.89108	7.15E-56	6	11.85897
Q9GZN1	396	45809.7	4.66	Actin-relat	ACTR6	ACTR6	Actin-relat	2	92.15292	49.60015	8.36E-55	2	5.555556
Q9H9F9	607	68296.9	4.94	Actin-relat	ACTR5	ACTR5	Actin-relat	5	274.4724	52.31143	4.31E-65	5	12.35585
P61158	418	47370.8	5.69	Actin-relat	ACTR3	ACTR3	Actin-relat	8	565.887	51.26643	4.74E-61	7	20.57416
P61160	394	44760.5	6.72	Actin-relat	ACTR2	ACTR2	Actin-relat	6	328.8148	52.78498	6.11E-67	6	19.03553
P61163	376	42613.3	6.61	Alpha-cen	ACTR1A	ACTR1A	Alpha-cen	6	131.2688	48.44986	1.14E-50	3	20.21277
Q9NZ32	417	46306.6	7.42	Actin-relat	ACTR10	ACTR10	Actin-relat	1	51.22467	23.40616	2.85E-06	1	2.158273
O43707	911	104853.2	5.12	Alpha-acti	ACTN4	ACTN4	Alpha-acti	24	2826.433	54.32981	3.97E-73	23	32.71131
P12814	892	103056.7	5.07	Alpha-acti	ACTN1	ACTN1	Alpha-acti	29	2394.923	53.95762	1.26E-71	12	40.69507
O96019	429	47460.7	5.3	Actin-like	ACTL6A	ACTL6A	Actin-like	10	565.2127	50.95664	7.17E-60	9	36.13054
Q562R1	376	42002.8	5.29	Beta-actin	ACTBL2	ACTBL2	Beta-actin	1	2805.358	38.13625	1E-21	1	4.787234
P60709	375	41736.4	5.15	Actin, cyto	ACTB	ACTB;ACT(	Actin, cyto	20	59608.59	52.83133	4.01E-67	7	65.33333
P62736	377	42008.6	5.05	Actin, aorti	ACTA2	ACTA2	Actin, aorti	4	4232.441	52.38752	2.18E-65		15.11936
Q9NR19	701	78579.1	6.43	Acetyl-coe	ACSS2	ACSS2	Acetyl-coe	2	108.7377	48.50972	7E-51	2	2.853067
Q9ULC5	683	75990.1	6.91	Long-chai	ACSL5	ACSL5	Long-chai	14	430.774	53.64334	2.37E-70	12	23.27965
O60488	711	79187.4	8.51	Long-chai	ACSL4	ACSL4	Long-chai	8	392.5732	53.28067	6.47E-69	8	13.36146
O95573	720	80419.4	8.51	Fatty acid (	ACSL3	ACSL3	Fatty acid (	17	1010.563	52.59794	3.26E-66	16	30.55556
P33121	698	77942.7	7.16	Long-chai	ACSL1	ACSL1	Long-chai	4	80.33125	44.51161	7.64E-38	4	6.446991
Q4G176	576	64129.6	8.47	Malonate-	ACSF3	ACSF3	Malonate-	6	131.7506	50.55457	2.41E-58	6	12.67361
Q9NPH0	428	48886	6.44	Lysophosp	ACP6	ACP6	Lysophosp	1	52.54436	20.99987	7.95E-06	1	2.570093
P11117	423	48343.9	6.73	Lysosomal	ACP2	ACP2	Lysosomal	1	70.91756	39.69659	3.96E-25	1	2.600473
P24666	158	18042.3	6.73	Low molec	ACP1	ACP1	Low molec	3	767.9575	48.03714	3.13E-49	3	15.82278
Q15067	660	74423	8.29	Peroxisom	ACOX1	ACOX1	Peroxisom	2	122.7587	42.15124	3.41E-31	2	3.636364
Q9Y305	439	49901.3	8.82	Acyl-coen	ACOT9	ACOT9	Acyl-coen	14	728.4711	50.49923	3.9E-58	14	34.62415
O14734	319	35914	7.6	Acyl-coen	ACOT8	ACOT8	Acyl-coen	3	221.8577	52.23348	8.65E-65	3	12.85266

O00154	380	41795.8	8.66	Cytosolic a	ACOT7	ACOT7	Cytosolic a	9	2546.667	49.5902	9.09E-55	9	30
Q8WXI4	607	68491.6	8.43	Acyl-coenz	ACOT11	ACOT11	Acyl-coenz	2	213.7966	39.01141	1.37E-23	2	3.789127
Q99798	780	85424.7	7.65	Aconitate I	ACO2	ACO2	Aconitate I	4	140.5527	46.60838	2.06E-44	4	5.769231
P21399	889	98398.1	6.66	Cytoplasm	ACO1	ACO1	Cytoplasm	3	97.86398	49.78581	1.75E-55	3	4.16198
P53396	1101	120838.3	7.34	ATP-citrat	ACLY	ACLY	ATP-citrat	16	351.0869	50.27297	2.71E-57	16	17.43869
Q9UKV3	1341	151860.1	6.36	Apoptotic	ACIN1	ACIN1	Apoptotic	39	4876.519	51.31789	3E-61	39	36.98732
Q96AP0	458	48966.7	4.97	Adrenocor	ACD	ACD	Adrenocor	2	148.2831	41.11096	1.62E-28	2	6.331878
Q9H3P7	528	60593	4.73	Golgi resid	ACBD3	ACBD3	Golgi resid	7	147.899	51.56175	3.47E-62	7	19.88636
P24752	427	45199.2	9.21	Acetyl-CoA	ACAT1	ACAT1	Acetyl-CoA	5	686.1778	49.82262	1.28E-55	5	13.34895
Q15057	778	88027.9	6.78	Arf-GAP w	ACAP2	ACAP2	Arf-GAP w	3	72.29487	44.64315	3.09E-38	3	5.269923
P49748	655	70389.6	9.1	Very long-	ACADVL	ACADVL	Very long-	20	1376.896	52.22693	9.17E-65	20	35.57252
P45954	432	47485	7	Short/bran	ACADSB	ACADSB	Short/bran	8	554.8825	52.97577	1.07E-67	8	20.60185
P16219	412	44296.7	8.12	Short-chai	ACADS	ACADS	Short-chai	2	56.25594	34.12718	1.37E-14	2	5.339806
P11310	421	46588	8.51	Medium-c	ACADM	ACADM	Medium-c	5	371.9126	51.52767	4.69E-62	5	14.72684
Q9H845	621	68759.7	8.05	Complex I	ACAD9	ACAD9	Complex I	8	940.9514	53.88946	2.37E-71	8	12.07729
Q9UKU7	415	45069.4	7.91	Isobutyryl-	ACAD8	ACAD8	Isobutyryl-	10	1773.184	49.08575	6.17E-53	10	28.6747
Q709F0	780	87263.7	8.11	Acyl-CoA (	ACAD11	ACAD11	Acyl-CoA (	6	505.9562	49.11481	4.85E-53	6	9.358974
Q6JQN1	1059	118833	8.13	Acyl-CoA (	ACAD10	ACAD10	Acyl-CoA (	5	91.20609	49.54231	1.36E-54	5	7.082153
O00763	2458	276538.6	6.46	Acetyl-CoA	ACACB	ACACB	Acetyl-CoA	5	385.5679	44.54058	6.27E-38	1	2.644426
Q13085	2346	265551.7	6.32	Acetyl-CoA	ACACA	ACACA	Acetyl-CoA	9	226.7194	49.48054	2.3E-54	9	4.603581
P09110	424	44291.6	8.55	3-ketoacyl	ACAA1	ACAA1	3-ketoacyl	5	150.0725	49.67801	4.33E-55	5	13.91509
A6QL63	1104	120882.9	6.82	Ankyrin re	ABTB3	ABTB3	Ankyrin re	2	69.79295	44.72336	1.77E-38	2	1.992754
Q9ULW3	272	31078.7	10.44	Activator c	ABT1	ABT1	Activator c	7	410.8386	50.35044	1.4E-57	7	34.19118
Q15018	415	46900.4	6.15	BRISC com	ABRAXAS2	ABRAXAS2	BRISC com	4	99.43488	48.4922	8.06E-51	4	10.60241
O94929	683	77800.9	8.62	Actin-binc	ABLIM3	ABLIM3	Actin-binc	2	57.45895	36.8206	3.96E-19	2	3.806735
Q6H8Q1	611	67811.5	7.99	Actin-binc	ABLIM2	ABLIM2	Actin-binc	1	608.3931	24.44162	1.59E-06	1	1.472995
O14639	778	87686.8	8.69	Actin-binc	ABLIM1	ABLIM1	Actin-binc	3	59.54531	45.32752	2.51E-40	3	5.141388
Q9NYB9	513	55662.8	6.08	Abl interac	ABI2	ABI2	Abl interac	4	142.9239	52.53035	6.01E-66	3	9.356725
Q8IZP0	508	55080.4	7.08	Abl interac	ABI1	ABI1	Abl interac	2	211.4522	45.55541	4.88E-41	2	5.314961
Q9BV23	337	38330.4	8.54	Monoacylg	ABHD6	ABHD6	Monoacylg	1	48.30321	37.13912	9.82E-20	1	3.560831
Q96IU4	210	22345.5	6.39	Putative pr	ABHD14B	ABHD14B	Putative pr	4	164.5777	53.11778	2.89E-68	4	26.66667
Q8N2K0	398	45096.3	8.82	Lysophosp	ABHD12	ABHD12	Lysophosp	3	141.0972	46.3769	1.17E-43	3	9.547739
Q8NFV4	306	33746.6	9.56	sn-1-spec	ABHD11	ABHD11	sn-1-spec	2	106.8021	24.50921	1.52E-06	2	10.13072
Q9NUJ1	306	33932.2	8.77	Palmitoyl-	ABHD10	ABHD10	Palmitoyl-	4	664.4984	46.5645	2.87E-44	4	15.35948
Q9NUQ8	709	79743.9	6.27	ATP-bindi	ABCF3	ABCF3	ATP-bindi	7	968.2079	45.71475	1.54E-41	7	11.42454
Q9UG63	623	71289.7	7.4	ATP-bindi	ABCF2	ABCF2	ATP-bindi	15	2583.431	52.39395	2.05E-65	15	25.36116
Q8NE71	845	95925.2	6.77	ATP-bindi	ABCF1	ABCF1	ATP-bindi	13	525.2453	52.73171	9.86E-67	13	19.52663
P61221	599	67313.7	8.45	ATP-bindi	ABCE1	ABCE1	ATP-bindi	14	986.5271	49.69171	3.86E-55	14	26.71119
P28288	659	75475.3	9.75	ATP-bindi	ABCD3	ABCD3	ATP-bindi	16	1578.617	51.5308	4.56E-62	16	30.95599
P33897	745	82936.1	9.2	ATP-bindi	ABCD1	ABCD1	ATP-bindi	4	74.381	48.86122	3.94E-52	4	6.845638
O15439	1325	149525.3	8.31	ATP-bindi	ABCC4	ABCC4	ATP-bindi	1	14.93912	28.18881	3.24E-08	1	0.603774
P33527	1531	171589.5	7.11	Multidrug	ABCC1	ABCC1	Multidrug	4	92.42783	47.02433	8.67E-46	4	3.592423
Q9NY61	560	63132.3	4.56	Protein AA	AATF	AATF	Protein AA	9	380.0774	54.47316	1E-73	9	25
Q9NRN7	309	35775.6	6.79	L-aminoac	AASDHPPT	AASDHPPT	L-aminoac	6	162.4614	43.28961	2.67E-34	6	22.3301
Q5JTZ9	985	107339.5	6.2	Alanine--t	AARS2	AARS2	Alanine--t	1	193.227	47.46994	2.75E-47	1	1.522843
P49588	968	106809.5	5.18	Alanine--t	AARS1	AARS1	Alanine--t	21	673.8882	54.41019	1.86E-73	21	26.23967
Q9Y312	384	43471.8	5.83	Protein AA	AAR2	AAR2	Protein AA	2	58.34837	43.04903	1.25E-33	2	6.770833
Q13685	434	46750.6	4.04	Angio-ass	AAMP	AAMP	Angio-ass	6	186.4713	50.79851	2.87E-59	6	14.74654
Q9NRG9	546	59573.6	7.53	Aladin	AAAS	AAAS	Aladin	2	316.3484	48.40521	1.63E-50	2	4.029304
P01023	1474	163289.9	6.43	Alpha-2-n	A2M	A2M	Alpha-2-n	9	203.914	47.52447	1.8E-47	8	7.666214
